# Dinitrogen Activation with Low‐Valent Strontium

**DOI:** 10.1002/anie.202506989

**Published:** 2025-05-22

**Authors:** Michael Morasch, Timothy Vilpas, Neha Patel, Johannes Maurer, Stefan Thum, Marcel A. Schmidt, Jens Langer, Sjoerd Harder

**Affiliations:** ^1^ Inorganic and Organometallic Chemistry Universität Erlangen‐Nürnberg Egerlandstrasse 1 91058 Erlangen Germany

**Keywords:** Calcium, DFT, Low‐valent, Nitrogen activation, Strontium

## Abstract

DFT calculations on β‐diketiminate (BDI) complexes with the full series of alkaline‐earth (Ae) metals show that (BDI)AeAe(BDI) complexes of the heavier Ae metals (Ca, Sr, Ba) have long weak Ae─Ae bonds that are prone to homolytic bond cleavage. However, isolation of (BDI)Sr(μ‐N_2_)Sr(BDI) with a side‐on bridging N_2_^2−^ dianion should thermodynamically be feasible. Attempts to stabilize such a complex with the super bulky BDI* ligand failed (BDI* = HC[(Me)C = N‐DIPeP]_2_, DIPeP = 2,6‐Et_2_CH‐phenyl). First, N_2_ fixation with a Sr complex was enabled by a heterobimetallic approach. Reduction of (^DIPeP^NN)Sr with potassium gave (^DIPeP^NN)_2_Sr_2_K_2_(N_2_) (**6**‐Sr); ^DIPeP^NN = ^DIPeP^N‐Si(Me)_2_CH_2_CH_2_Si(Me)_2_‐N^DIPeP^. A similar Ca product was also isolated (**6**‐Ca). Crystal structures reveal a N_2_^2−^ anion with side‐on bonding to Ae^2+^ and end‐on coordination to K^+^. DFT calculations and Atoms‐In‐Molecules analyses show mainly ionic bonding. Both **6**‐Ae complexes are synthons for hitherto unknown (BDI*)AeAe(BDI*) (Ae = Ca, Sr) and react by releasing N_2_ and two electrons. Although surprisingly stable in benzene, the reduction of I_2_ and H_2_ is facile. Fast reaction with Teflon led to formation of crystalline [(^DIPeP^NN)SrKF]_2_ (**7**), which is labile and decomposed to KF and (^DIPeP^NN)Sr. Latter reactivity underscores potential use of **6**‐Ae complexes as very strong, hydrocarbon‐soluble reducing agents.

## Graphical abstract

Although computationally predicted, N_2_ activation with an in situ generated low‐valent β‐diketiminate (BDI) Sr^I^ precursor did not result in a stable complex. First dinitrogen fixation with Sr was achieved using a heterobimetallic approach. This highly reducing product vigorously reacts with Teflon.

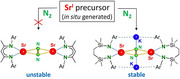

## Introduction

**Scheme 1:**
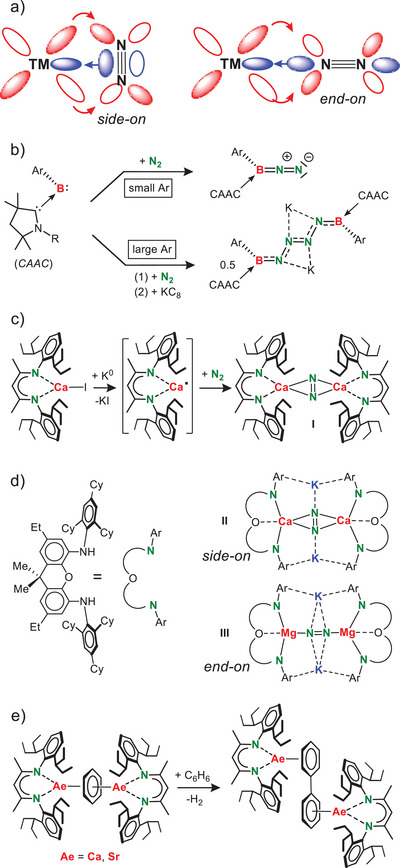
a) π‐Backbonding model for N_2_ activation with a transition metal (TM). b) Activation of N_2_ with in situ generated *CAAC*‐stabilized borylene. c) In situ generation of low‐valent Ca^I^ species for N_2_ activation to give the Ca^I^ synthon **I**. d) Alkaline‐earth metal N_2_ complexes with a super bulky NON‐ligand. e) Alkaline‐earth metal complexes with bridging C_6_H_6_^2−^ for dehydrogenative coupling of benzene.

Activation and cleavage of the dinitrogen N≡N triple bond, one of the strongest known chemical bonds, is an important step toward the syntheses of N‐containing commodity chemicals.^[^ ^1^ ^]^ This major challenge can generally be achieved by use of transition metals which can activate N_2_ by synergistic donor–acceptor bonding, either in side‐on or end‐on fashion (Scheme 1a). The conversion of N_2_ to NH_3_ by Haber–Bosch catalysis is worldwide one of the largest bulk processes. Due to enormous demand and harsh reaction conditions (400 °C–600 °C, 200–400 bar), this process requires nearly 2% of the world‐wide energy consumption.^[^ ^1^ ^]^ Modern investigations focus on N_2_ fixation under mild, homogeneous conditions by precise catalyst design. ^[^ ^2^, ^3^, ^4^, ^5^ ^]^

Although this work is traditionally based on N_2_ activation with transition metals, quite some progress has been made with lanthanide metal complexes.^[^ ^6^ ^]^ Most recently, it was shown that even *s*‐ and *p*‐block (semi‐)metals are able to activate N_2_.^[^ ^7^, ^8^ ^]^ The Braunschweig group activated N_2_ at a B center (Scheme 1b),^[^ ^9^ ^]^ showing that *d*‐orbital participation is not a strict requirement. Later work by Mézailles and coworkers demonstrated N_2_→NH_3_ conversion with in situ generated B‐centered radicals.^[^ ^10^ ^]^ Aiming to isolate complexes with a unique Ca─Ca bond, we serendipitously found N_2_ activation by low‐valent Ca^I^ radicals (Scheme 1c, **I**).^[^ ^11^ ^]^ This was followed by several examples of Mg/K and Ca/K‐mediated N_2_ fixation by Jones and coworkers (Scheme 1d, **II**‐**III**).^[^ ^12^, ^13^ ^]^ Calcium complex **I** should be regarded to consist of N_2_^2−^ ions that bridge two Ca^2+^ centers in a side‐on (*μ*‐*η* ^2^,*η* ^2^)‐fashion. Although we never achieved the isolation of a “true” Ca^I^ complex, **I** reacts as a highly reducing Ca^I^ synthon by releasing N_2_ and two electrons. In this function it is able to convert highly stable aromatic arenes like benzene into 8π‐electron, anti‐aromatic C_6_H_6_^2^ˉ (Scheme 1e) which in turn functions as a Ca^I^ synthon by release of the arene and two electrons.^[^ ^14^ ^]^ In addition, dehydrogenative benzene‐to‐benzene coupling was shown to give biphenyl complexes.^[^ ^15^ ^]^ These latter investigations showed that the Sr complexes reacted much faster and more selective than the corresponding Ca complexes. Since it is well known that group 2 metal reactivity increases steadily going down the group (Be < Mg < Ca < Sr < Ba),^[^ ^16^ ^]^ we targeted Sr‐mediated N_2_ activation and the challenging isolation of labile, highly air‐sensitive Sr^I^ synthons with strongly reducing N_2_^2−^ ions. Herein, we describe our combined computational and experimental studies.

## Results and Discussion

### Computational Studies on the Ae─Ae Bond and N_2_ Activation

Computational studies on the Ae─Ae bond strength in CpAeAeCp model systems have been reported for the full series of alkaline‐earth (Ae) metals.^[^ ^17^, ^18^ ^]^ Except for the most stable complex in this series, CpBeBeCp,^[^ ^19^ ^]^ these are fictitious species. Given that the β‐diketiminate (BDI) ligands play a prominent role for stabilization of Ae metals in low oxidation states,^[^ ^20^, ^21^, ^22^ ^]^ it is surprising that theoretical work on (BDI)AeAe(BDI) complexes is limited only to Mg and Ca.^[^ ^23^, ^24^ ^]^ In order to gain insight on the general stability of (BDI)AeAe(BDI) complexes and the thermodynamics of their reactivities with N_2_, we started this project with DFT calculations on the full series (Ae = Be, Mg, Ca, Sr, Ba).

**Figure 1:**
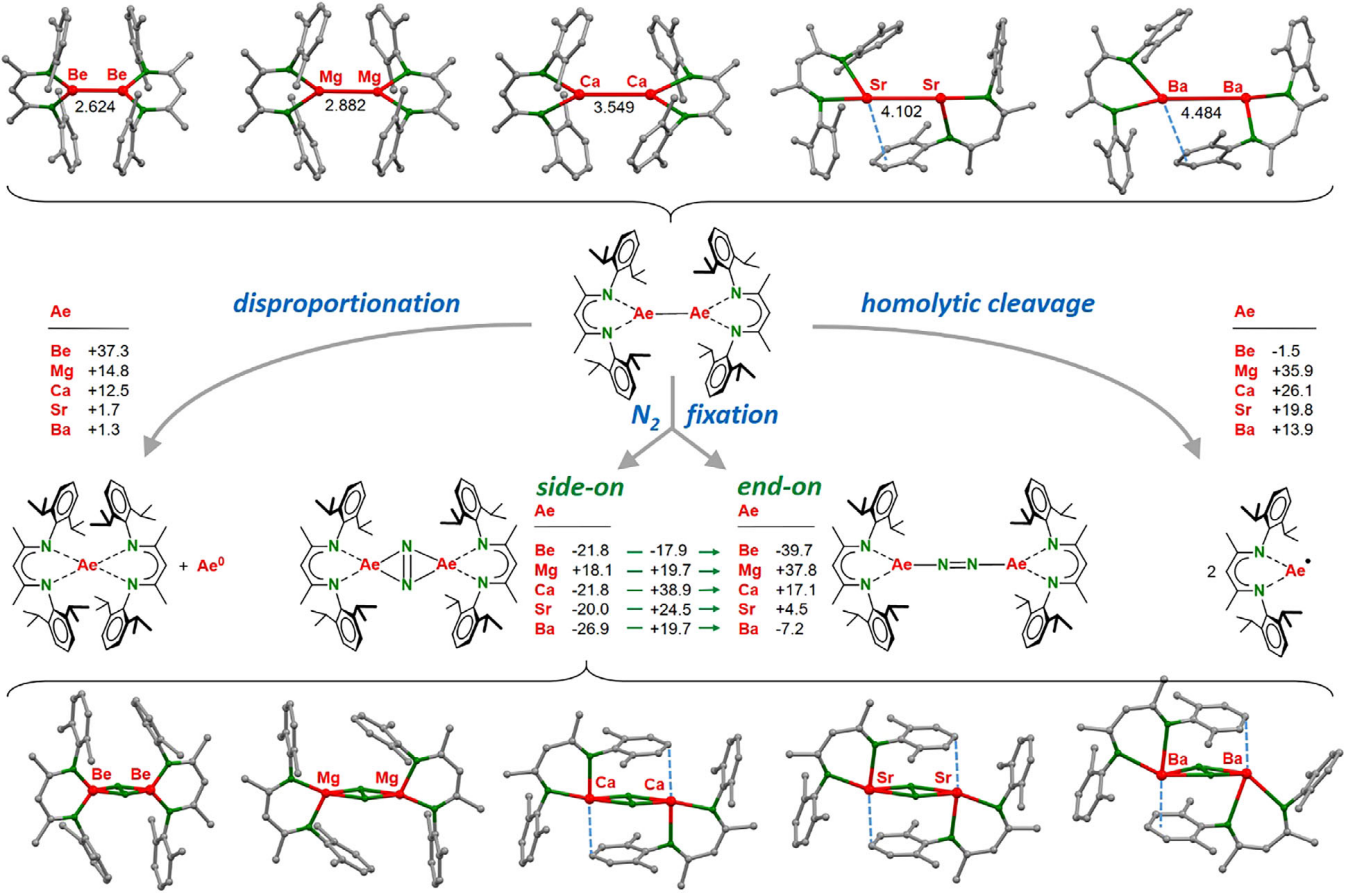
a) DFT study on the stability of (BDI)AeAe(BDI) complexes at the B3PW91/def2TZVP//def2SVP level of theory which includes corrections for dispersion. Energies are given as Δ*G*(298 K) in kcal molˉ^1^.

Using the standard BDI ligand with DIPP‐substituents (BDI = HC[(Me)C = N‐DIPP]_2_, DIPP = 2,6‐Me_2_CH‐phenyl), we modelled the complete series of (BDI)AeAe(BDI) complexes (Figure 1); details can be found in the Supporting Information. The validity of our method was confirmed by the fact that the calculated Mg─Mg bond of 2.883 Å fits the experimental value of 2.8457(8) Å quite well and NPA charges on Mg (+0.98) are in line with Mg^+I^.

The Ae─Ae bond for the heavier metals (Ca, Sr, Ba) become rather long and the heaviest complexes in the series (Sr and Ba) show Ae‐arene interactions (Figures 1 and S75). This asymmetry results in partial transfer of electron density from the ring‐stabilized Ae metal to the Ae nucleus without this stabilizing interaction. The NPA charges on the metals in (BDI)BaBa(BDI) are now +1.26 (ring‐stabilized) and + 0.69 (3‐coordinate Ba). This asymmetry is only found for the heavier complexes and only when dispersion is included. Optimization of (BDI)BaBa(BDI) without dispersion correction resulted in a symmetric dimer with 3‐coordinate Ba metals and a NPA charge of +0.94 on each Ba nucleus (Figure S76). However, the unrealistically long Ba‐Ba distance of 4.75 Å and a negative free energy for homolytic cleavage (Δ*G* = −1.8 kcal mol^−1^) indicate instability.

The BDI ligand is clearly too bulky for Be and an extremely long Be‐Be distance of 2.624 Å in (BDI)BeBe(BDI) is calculated. For comparison, the experimentally determined Be‐Be distance in CpBeBeCp is only 2.055(2) Å.^[^ ^19^ ^]^

All (BDI)AeAe(BDI) complexes are thermodynamically stable toward disproportionation into (BDI)_2_Ae and atomic Ae^0^ but this stability rapidly decreases down the group. However, if one considers the metal atomization enthalpies (kcal mol^−1^: Be 77.7, Mg 35.5, Ca 42.6, Sr 39.3, Ba 43.1), none of the complexes are stable. Disproportionation to (BDI)_2_Ae and metallic (Ae^0^)_n_ is in all cases exothermic. Their existence can only be rationalized with high kinetic barriers for disproportionation.

In agreement with experiment,^[^ ^25^ ^]^ Atoms‐In‐Molecules (AIM) analysis (Figure S84) shows a Non‐Nuclear‐Attractor (NNA) with a basin of 0.80*e* at the Mg‐Mg axis. All other (BDI)AeAe(BDI) complexes do not have NNA's on the Ae‐Ae axis but show weak Ae─Ae bond paths with low values for the electron density *ρ*(r) and Laplacian **∇^2^** *ρ*(r) in the bond‐critical‐points (*bcp*’*s)*. These values steadily decrease from Be to Ba. However, optimization of the structures without considering dispersion results in even longer Ae‐Ae distances and in this case also NNA's are observed for the Ca─Ca (basin: 0.33*e*) and Sr─Sr (basin: 0.35*e*) bonds (Figures S85–S86).

Homolytic Ae─Ae bond cleavage is slightly endergonic with bond energies rapidly decreasing down the group. Only for (BDI)BeBe(BDI) an exergonic cleavage is found. This is related to steric stress between the two oversized BDI ligands. Due to the rather low bond energies for the heavier Ae─Ae (Ca, Sr, Ba) bonds, it is plausible that these species are in equilibrium with highly reactive (BDI)Ae^●^ radicals dominating their reactivities.

Steric stress in (BDI)BeBe(BDI) is also released by reaction with N_2_ which is highly exergonic, especially for end‐on N_2_ bridging in which case BDI···BDI repulsion is reduced even further. For all other metals, side‐on N_2_ coordination is preferred. In the range of (BDI)AeAe(BDI) complexes, the Mg complex is the only complex for which N_2_ fixation is endergonic. In this light, the recently observed N_2_ activation with a low‐valent Mg complex is remarkable (**III**).^[^ ^12^ ^]^ Since for the Mg‐N_2_ complex **III** the triplet state was calculated to be 13.9 kcal mol^−1^ more stable than the singlet state, far‐fetching conclusions from this preliminary computational study can only be drawn after full evaluation of the alternative triplet states. As we reported earlier for Ca‐N_2_ complex **I**,^[^ ^11^ ^]^ the activation of N_2_ likely proceeds through reactive (BDI)Ae^●^ radicals.

The calculations in Figure 1 suggest that hypothetical (BDI)SrSr(BDI) is only weakly bound and unstable. In contrast, the corresponding Sr‐N_2_ complex is nearly as stable as the Ca‐N_2_ complex **I** and was target of further experimental investigations.

### Experimental Studies on N_2_ Activation with Sr^I^

The successful verification of N_2_ activation with an in situ formed low‐valent β‐diketiminate Ca complex (Scheme 1c) was enabled by introduction of a super bulky ligand with DIPeP‐substituents at N: HC[(Me)C = N‐DIPeP]_2_, DIPeP = 2,6‐Et_2_CH‐phenyl. This ligand, abbreviated herein as BDI*, is not only very bulky^[^ ^26^ ^]^ but its flexible alkyl arms enable dissolution of the highly reducing products in inert alkane solvents.^[^ ^11^ ^]^ Aromatic solvents would be immediately reduced to 8π‐electron, anti‐aromatic dianionic rings. (*cf*. Scheme 1e). Using the BDI* ligand, N_2_ activation studies with *in* *situ* generated low‐valent Sr^I^ can be performed in alkane solvents.

**Scheme 2:**
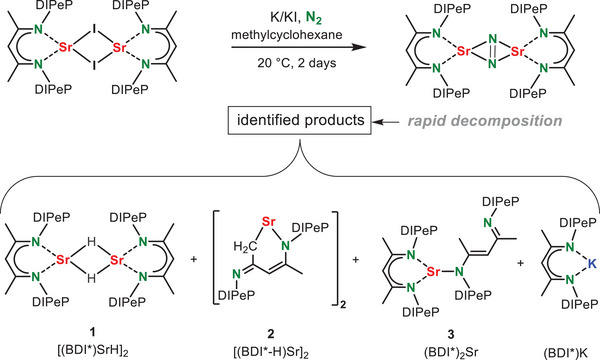
Attempted N_2_ activation with in situ prepared (BDI*)Sr^I^ led to formation of various products.

The room temperature reduction of [(BDI*)SrI]_2_ with K/KI^[^ ^27^ ^]^ under an N_2_ atmosphere in methylcyclohexane (Scheme 2) led to various products that can be conveniently assigned by their typical ^1^H NMR signals for the backbone methine group in the BDI* ligand (Figure S51). Thus, we were able to identify the products [(BDI*)SrH]_2_ (**1**), [(BDI*‐H)Sr]_2_ (**2**) and (BDI*)_2_Sr (**3**) as well as overreduction to (BDI*)K. This was confirmed by addition of THF and crystallization of the THF adducts of **2**·(THF)_2_ and **3**·(THF)_2_ which were identified in the product mixture by X‐ray diffraction (Figures S52 and S54). Compounds **1** and **2** are proposed to be formed by reductive cleavage of the C─H bond of the backbone Me group. Formation of **3** may be explained by disproportionation of (BDI*)SrSr(BDI*) as described in Figure 1. Although a solid‐state mechanochemical reduction of [(BDI*)SrI]_2_ with K/KI using the ball‐mill gave a different product distribution (Figure S53), we have not been able to identify or isolate the expected (BDI*)Sr(μ‐N_2_)Sr(BDI*) product. This stands in strong contrast with the successful isolation of the corresponding Ca complex **I**.^[^ ^11^ ^]^ Failure to isolate the target Sr complex is most likely due to its longer, more ionic, and weaker bonds which result in a much higher reactivity and lower stability.

### Heterobimetallic N_2_ Activation

**Scheme 3:**
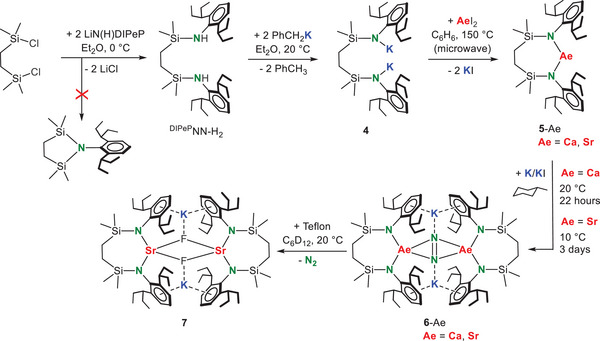
Synthesis of a new super bulky *bis*‐amine (^DIPeP^NN‐H_2_), conversion to (^DIPeP^NN)Ae (**5**‐Ae, Ae = Ca, Sr) and N_2_ activation with K/KI. The product **6**‐Sr reacts fast with Teflon to give the fluoride complex **7**.

To increase complex stability, we chose to follow a heterobimetallic approach. This concept, which is directly related to Mulvey's inverse crown ether chemistry,^[^ ^28^ ^]^ takes advantage of stabilizing anions in a crown of metal cations and was recently successfully used to stabilize N_2_^2−^ (*cf*. **II**‐**III**)^[^ ^12^, ^13^ ^]^ or the SiH_6_^2^ˉ anion.^[^ ^29^ ^]^ The essential building blocks for this chemistry are a chelating *bis*‐amide ligand, an Ae^2+^ cation and an alkali metal cation.^[^ ^12^, ^13^ ^] [^ ^29^, ^30^, ^31^, ^32^, ^33^ ^]^ Most recently, *bis*‐amide complexes of the heaviest Ae metals Sr and Ba have been reported.^[^ ^32^, ^34^ ^]^ However, these compounds were found to be notoriously insoluble in non‐donor solvents. Modifying a ligand synthesis of Hill and coworkers,^[^ ^30^ ^]^ we developed a considerably more soluble and bulkier *bis*‐amide ligand system based on the DIPeP‐substituent (^DIPeP^NN, Scheme 3). The preligand (^DIPeP^NN‐H_2_) was obtained in quantitative yield by reaction of (DIPeP)N(H)Li with a chlorosilane bridge in Et_2_O at 0 °C (crystal structure: Figure S68). Solvent choice and temperature control are essential to avoid cyclic side‐products. For comparison, reaction of this bridge with the less bulky (DIPP)N(H)Li led to considerable quantities of a cyclic side‐product,^[^ ^30^ ^]^ showing that the use of very bulky substituents is also advantageous in ligand preparation.

Deprotonation of ^DIPeP^NN‐H_2_ with benzylpotassium gave (^DIPeP^NN)K_2_ (**4**) (crystal structure of its THF adduct: Figure S69). Using salt‐metathesis, **4** could be converted to the heavier Ae metal complexes **5**‐Ae (Ae = Ca, Sr). As we aim for donor‐free products this conversion was performed in benzene. Due to the bulk of the ligand this was a challenging reaction. However, using harsh microwave conditions the complexes could be obtained in >80% isolated yields.

**Figure 2:**
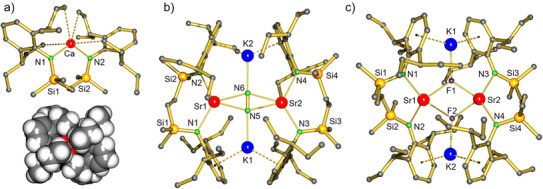
a) Crystal structure of **5**‐Ca viewed perpendicular to a non‐crystallographic *C*_2_‐axis and the space‐filling viewed along this axis. b) Crystal structure of **6**‐Sr. c) Crystal structure of **7**. Selected bond distances (Å): Sr‐N: 2.521(2)‐2.624(2) (average: 2.579), Sr‐F: 2.362(1)‐2.422(1), (average: 2.393), K1‐F1 2.491(1), K2‐F2 2.505(1). In all cases, H atoms have been omitted for clarity.

The crystal structure of **5**‐Ca (Figure 2a) shows a monomeric complex with approximate, non‐crystallographic, *C*_2_‐symmetry. Product **5**‐Ca is an odd example of a complex with a bent two‐coordinate metal center. With a N‐Ca‐N angle of 119.86(4)°, a large part of the Ca coordination sphere remains “naked”. The bulky ^DIPeP^NN‐ligand restricts dimerization and instead provides only weak secondary Ca···CH_3_CH_2_ interactions (Ca···C: 3.090(2)‐3.134(2) Å). Donor‐free **5**‐Sr could not be crystallized but the *C*_2_‐symmetric complex **5**‐Sr·(THF)_2_ is monomeric with a four‐coordinate Sr atom that shows Sr─N and Sr─O bonds of 2.466(2) Å and 2.591(2) Å, respectively (Figure S70).

Reduction of the donor‐free monomeric *bis*‐amide complexes **5**‐Ae with K/KI^[^ ^27^ ^]^ in methylcyclohexane gave the corresponding N_2_ complexes **6**‐Ae. Reductions with KC_8_ or K^0^ gave the same products but were less selective. The conversion of **5**‐Ca with K/KI was clean and the raw product is essentially pure. After crystallization **6**‐Ca was isolated in the form of orange crystals in 83% yield. Isolation of the analogue**6**‐Sr required temperature lowering to 10 °C. Temperatures below 10 °C gave very slow conversion, higher temperatures led to product decomposition. Despite temperature control, the formation of **6**‐Sr was only circa 75% selective and the product could be crystallized in a low yield of 9%. This much lower yield is not only due to instability but also to its very high solubility in alkanes.

Although **6**‐Ca and **6**‐Sr crystallize in different space groups, their structures are very similar (Figures 2b and S72–S73). The crystal structure of **6**‐Ca is highly symmetric (space group *F*ddd, *D*_2_ symmetry) with three perpendicular crystallographic *C*_2_ axes through the center of the aggregate. Complex **6**‐Sr shows no crystallographic symmetry but is close to being *D*_2_ symmetric. The Ae^2+^ and K^+^ cations and embedded N_2_^2−^ anion are coplanar, showing side‐on Ae‐N_2_‐Ae and end‐on K‐N_2_‐K bridging.

Table 1 summarizes some of the most important bond distances and angles for **6**‐Ae in comparison to other known Ae‐N_2_ complexes. The Ca‐N_2_ distances in **6**‐Ca are nearly 0.1 Å longer than those in **II**. For that, the K‐N_2_ distances in **6**‐Ca are nearly 0.1 Å shorter than those in **II**. Although the ionic radius of Sr^2+^ (1.18 Å, 6‐coordinate) is 0.18 Å larger than that of Ca^2+^ (1.00 Å, 6‐coordinate),^[^ ^35^ ^]^ the Sr‐N_2_ bonds in **6**‐Sr are only 0.12 Å longer than the Ca─N_2_ bonds in **6**‐Ca. This indicates a very tight Sr‐N_2_ interaction which results in longer K─N_2_ bonds in **6**‐Sr. The N‐N distances vary from 1.230(9)‐1.265(3) Å which is the typical range for N = N bonding in N_2_^2^^−^; for comparison the triple bond in N_2_ has a length of 1.098 Å. The small variation in N = N bond lengths and partially large standard deviation as well as different coordination geometries do not allow for clear‐cut conclusions on the degree of bond activation. Measurement of the Raman frequencies is probably a more accurate method to evaluate N_2_ activation. Unfortunately, complexes **6**‐Ae are both sensitive to laser light and decompose upon radiation (Figures S41 and S49). Although this is especially the case for **6**‐Sr, a weak N = N stretching band could be observed at 1428 cmˉ^1^ (**6**‐Ca: 1440 cmˉ^1^). The efficiency of N_2_ activation increases along the row: **III** < **6**‐Ca < **6**‐Sr < **II** < **I**. In general, N_2_ activation increases with metal size Mg < Ca < Sr. Activation is also considerably stronger in the homometallic Ca complex **I** when compared to heterobimetallic Ca/K complexes (**6**‐Ca or **III**).

**Table 1:** Selected distances (Å) and ligand bite angle (°) in Ae‐N_2_ complexes (range and average value between []) and Raman frequencies (cm^−1^) for the N = N bond.

Complex	**6**‐Ca	**6**‐Sr	**I**·(THF)_2_	**II**	**III**
Ae‐N_L_ ^a)^	2.373(3)	2.522(3)‐2.533(3) [2.526]	2.393(2), 2.396(2) [2.395]	2.342(3)‐2.354(2) [2.346]	2.047(3)‐2.069(2) [2.056]
Ae‐N_2_	2.4324(17)	2.527(2)‐ 2.565(3) [2.552]	2.301(2), 2.303(2) [2.302]	2.348(2)‐2.357(2) [2.351]	1.987(2)‐1.993(2) [1.990]
K‐N_2_	2.608(5)	2.668(3), 2.707(3) [2.688]	−	2.688(3), 2.692(3) [2.690]	2.762(3)‐2.819(3) [2.793]
N‐N	1.230(9)	1.251(4)	1.258(3)	1.265(3)	1.255(2)
N_L_‐Ae‐N_L_	112.5(2)	123.2(1), 122.4(1) [122.8]	83.9(1)	130.2(1), 130.2(1) [130.2]	140.2(1), 140.7(1) [140.5]
N‐N (Raman)	1440	1428	1375	1419	1530

^a)^ N_L_ is the N of the ^DIPeP^NN‐ligand.

**Figure 3:**
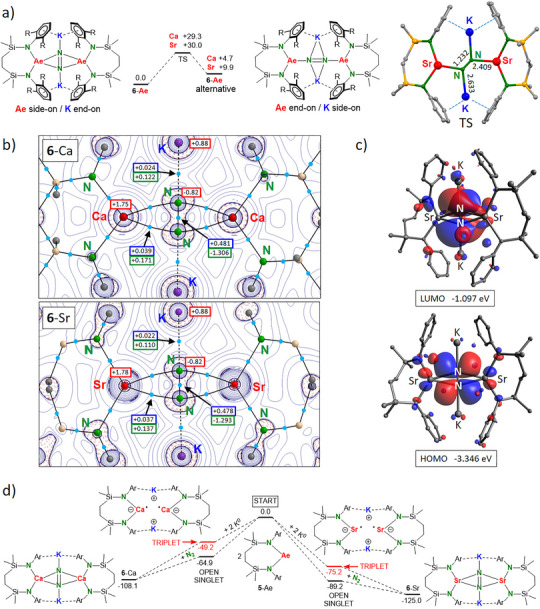
a) Energy profile for rotation of the N_2_^2^^−^ anion in **6**‐Ae (Δ*H* in kcal molˉ^1^). Right: Transition state for rotation in **6**‐Sr (distances in Å). b) Atoms‐In‐Molecules (AIM) analysis. Laplacian distributions for complexes **6**‐Ca and **6**‐Sr in the Ae(N_2_)Ae plane. Bond‐critical‐points (*bcp*’s) are shown as light‐blue spheres. The electron density, *ρ*(r) (blue boxes), and Laplacian, **∇^2^** *ρ*(r) (green boxes), in the *bcp*’s are given in atomic units. Red boxes show NPA charges. (c) HOMO and LUMO for **6**‐Sr. The CHEt_2_ substituents have been omitted for clarity. d) Computed energy profile for formation of **6**‐Ca and **6**‐Sr (Δ*H* in kcal molˉ^1^).

The computed structures of **6**‐Ae compare well to the crystal structures (Figure S90). We also located energy minima for alternative structures in which the coordination mode for N_2_^2^− switched to end‐on Ae‐N_2_‐Ae and side‐on K‐N_2_‐K bridging (Figure 3a). For **6**‐Sr this alternative structure is high in energy (Δ*H* = +9.9 kcal molˉ^1^) but for **6**‐Ca it is more stable (Δ*H* = +4.7 kcal molˉ^1^). The heterobimetallic Mg/K‐N_2_ complex **III** favors end‐on Ae‐N_2_‐Ae bonding.^[^ ^12^ ^]^ Therefore, the tendency to form end‐on complexes decreases from Mg > Ca > Sr. This may be explained by the higher electronegativity of Mg which favors more covalent σ‐bonding. It could also be rationalized by the Hard‐Soft‐Acid‐Base theory in which the smaller Mg^2+^ prefers bonding with the more compact end‐on *sp*‐hybrid orbitals whereas larger metal cations like Ca^2+^ and Sr^2+^ favor interaction with the more diffuse *p*‐orbitals.

In the transition state for N_2_ rotation, the N_2_^2^− dianion has been rotated within the Ae(N_2_)Ae plane by circa 45° and is now end‐on bridging the Ae^2+^ and K^+^ cations in μ_4_‐fashion (Figure 3a). For both, Ca and Sr, the energy barriers are relatively high (Δ*H* = +29.3 and +30.0 kcal molˉ^1^, respectively). Therefore, such rotational processes are unlikely at room temperature.

Atoms‐In‐Molecules (AIM) shows bond paths between the N_2_ nuclei and the Ae metals (Figure 3b). Small values for the electron density *ρ*(**r**) and Laplacian **∇^2^** *ρ*(**r**) in the *bcp*’s indicate ionic Ae─N_2_ bonding. This is confirmed by the calculated NPA charges in **6**‐Ca (Ca: +1.75, N_2_: −1.64) and **6**‐Sr (Sr: +1.78, N_2_: −1.66). Both complexes have similar HOMO's and LUMO's. The HOMO for **6**‐Sr clearly shows *d*‐orbital contribution of Sr (5.4% for each Sr atom) overlapping with the in‐plane N_2_ π*‐orbital (Figure 2c). The *d*‐orbital contribution for Ca in **6‐**Ca is somewhat smaller (4.0% for each Ca atom). The LUMO's are located on N_2_ and consist mainly of the N_2_ π*‐orbital perpendicular on the Ae(N_2_)Ae plane.

The energy profile for N_2_ activation shows that this is a highly exothermic process. Formation of **6**‐Sr from **5**‐Sr and K^0^ (Δ*H* = −125.0 kcal molˉ^1^) is considerably more exothermic than the formation of **6**‐Ca (Δ*H* = −108.1 kcal molˉ^1^); Figure 3d. Although there are many possible pathways for product formation, we propose the following Ae intermediates: {[(^DIPeP^NN)Ae^I^]ˉK^+^}_2_. Formation of these intermediates is also quite exothermic. The large energy differences of circa 15 kcal molˉ^1^ between the triplet and unrestricted open singlet state indicates that, despite large Ae···Ae distances, these proposed intermediates have little diradicaloid character.^[^ ^36^ ^]^

Both, **6**‐Ca and **6**‐Sr, are reasonably well soluble in alkanes. ^1^H NMR spectra of a cyclohexane‐*d*_12_ solution of **6**‐Ca show over a large temperature range (−40 °C to + 80 °C) broad resonances (Figure S39). In contrast, the ^1^H NMR spectrum of **6**‐Sr in cyclohexane‐*d*_12_ shows at 25 °C mainly sharp well‐resolved resonances corresponding to the high symmetry in the crystal structure (Figure S42). Line‐broadening of the C(H)Et_2_ signals may be attributed to dynamic anagostic C(H)Et_2_···Sr interactions. The broad ^1^H NMR signals for **6**‐Ca compared to sharp resonances for **6**‐Sr may be explained by different singlet‐triplet gaps. Although for both compounds the singlet state is most stable, both show very small singlet–triplet gaps (Δ*H* for Ca: 2.1 kcal mol^−1^, Sr 0.3 kcal mol^−1^). The differences in signal broadening are therefore likely related to differences in metal sizes and complex dynamics like the ring puckering of the seven‐membered silazane ring.

In form of pure compounds **6**‐Ca and **6**‐Sr are fully stable in benzene, even at reflux temperature. This stands in strong contrast with complex **II** which instantly decomposed when dissolved in benzene.^[^ ^13^ ^]^ Even the Mg‐N_2_ complex **III** is able to reduce benzene.^[^ ^37^ ^]^ As the N_2_^2−^ anions in **6**‐Ca and **II** or **III** are in all cases highly protected by super bulky ligands, this difference in stability is unclear.

In strong contrast, methylcyclohexane‐*d_14_* solutions of **6**‐Ca and **6**‐Sr both reacted already at −75 °C with H_2_. However, despite these controlled conditions, these reactions were unselective and well‐defined products could not be isolated. Also, reactions with I_2_ led to the formation of several products that could not be identified. During our investigations on the synthesis of **6**‐Sr, a small batch of yellow crystals was isolated. X‐ray diffraction showed a dimeric complex of composition [(^DIPeP^NN)SrKF]_2_ (**7**, Figure 1d). The presence of fluoride was confirmed by a ^19^F NMR resonance at –68.1 ppm (298 K, C_6_D_12_). The compound, which has a similar build up as a previously reported heterobimetallic Ca/K fluoride complex,^[^ ^31^ ^]^ could be considered a soluble form of K^+^Fˉ stabilized between two neutral (^DIPeP^NN)Sr fragments. However, as the ^19^F NMR signal of complex **7** slowly disappeared over time, the complex was found to be quite unstable in solution. From the appearance of ^1^H NMR signals for (^DIPeP^NN)Sr (**5**‐Sr), we assume that precipitation of KF salt is the driving force for this decomposition.

The origin of the fluoride anions in **7** is undoubtedly the Teflon‐coated stirring bars used in the synthesis of **6**‐Sr. Indeed, the Teflon‐coated stirring bars generally turned black during these experiments. For this reason, it is advisable to use glass‐coated stirring bars for this chemistry.

Additional proof for this reactivity was found in the very fast reaction of **6**‐Sr with Teflon powder. The addition of commercially available Teflon powder to a cyclohexane solution of **6**‐Sr led to immediate gas evolution and a colour change from white to black, a reaction that was complete within minutes. In addition to ^19^F NMR resonances of Teflon decomposition products, we observed a strong resonance at –68.4 ppm which is indicative for **7** (Figure S59). However, due to the poor stability of this complex we have not been able to isolate larger quantities. Similar observations were made for the reactivity of **6**‐Ca with Teflon powder. The fast reaction of Teflon with **6**‐Ca and **6**‐Sr, which are Ca^I^ and Sr^I^ synthons, contrasts with the much lower reactivity of a Mg^I^ complex which needs activation by dimethylaminopyridine (DMAP) and long reaction times to reach full conversion at room temperature.^[^ ^38^ ^]^

## Conclusion

According to DFT calculations, (BDI)SrSr(BDI) is thermodynamically not stable toward Sr^II^/(Sr^0^)_n_ disproportionation. As the Sr─Sr bond energy is smaller than 20 kcal molˉ^1^, also homolytic cleavage is facile. However, similar as found for the analogue Ca compounds, N_2_ activation to give a stable (BDI)Sr(μ‐N_2_)Sr(BDI) complex with a side‐on bridging N_2_^2^^−^ dianion should be feasible. However, while (BDI*)Ca(μ‐N_2_)Ca(BDI*) stabilized by super bulky BDI* ligands have been synthesized, all attempts to prepare the comparable Sr complex failed and only decomposition products could be isolated. This instability is likely the result of the higher ionicity of Sr compounds and the much larger size of Sr^2+^ (1.18 Å) compared to Ca^2+^ (1.00 Å). Both make the target Sr complex much more reactive than (BDI*)Ca(μ‐N_2_)Ca(BDI*).

The first example of N_2_ fixation with a Sr complex could be realized using a heterobimetallic approach. In complex **6**‐Sr the N_2_^2−^ anion is embedded in a metal crown consisting of two Sr^2+^ and two K^+^ cations. While the Sr^2+^ cations bind the N_2_^2^^−^ anion in a side‐on fashion, the K^+^ cations are coordinated end‐on. Calculations show that a structure with side‐on Sr─N_2_ bonding is 9.9 kcal molˉ^1^ more stable than alternative end‐on Sr─N_2_ bonding. The transition state between these two conformations is high (Δ*H* = 30.0 kcal molˉ^1^), indicating that at room temperature there is no free N_2_ rotation. The HOMO of **6**‐Sr mainly consists of a N_2_ π* orbital but also has considerable contributions of both Sr *d*‐orbitals. With NPA charges of + 1.78 on Sr and −1.66 on N_2_ bonding in **6**‐Sr is mainly of ionic nature. A similar synthetic approach led to isolation of the analogue Ca complex **6**‐Ca.

Preliminary reactivity studies show that both, **6**‐Ca and even **6**‐Sr, are stable in benzene under reflux conditions. Considering the facile reduction of benzene with known bulky Ae‐N_2_ complexes, it is unclear why **6**‐Ae would not react with benzene. However, both **6**‐Ae complexes react under release of N_2_ smoothly with H_2_ or I_2_ but the products of these reactions could not be isolated. The very high reactivity of **6**‐Sr as a synthon for low‐valent Sr^I^ was demonstrated by the reduction of Teflon (used as stirring bar coating) which is generally highly inert. This gave a small quantity of crystalline [(^DIPeP^NN)SrKF]_2_ (**7**) which could be seen as a molecular KF embedded between two (^DIPeP^NN)Sr compounds. Unfortunately, this complex is very labile and easily loses KF. The stirring bar as source of fluoride was confirmed by the vigorous N_2_ release when **6**‐Ae complexes are brought in contact with Teflon powder. This reactivity underscores potential use of **6**‐Ae complexes as very strong reducing agents which are hydrocarbon‐soluble and can be dosed in exact stoichiometry. We are currently targeting the most challenging isolation of even more reactive Ba‐N_2_ complexes.

## Conflict of Interests

The authors declare no conflict of interest.

## Electronic Supporting Information available

Experimental details, NMR spectra, crystallographic details including ORTEP, details for DFT calculations.

## Supporting information



Supporting Information

## Data Availability

The data that support the findings of this study are available in the Supporting Information of this article.
