# Supplementary material for: Dinitrogen Activation with Low‐Valent Strontium
Source: Angew Chem Int Ed Engl. 2025 May 22;64(28):e202506989. doi: 10.1002/anie.202506989 (PMC12232880; doi:10.1002/anie.202506989)
Supplement: Supplementary file 1 — Supporting Information [file ANIE-64-e202506989-s001.pdf]

Supporting Information

Dinitrogen Activation with Low-Valent Strontium

Michael Morasch, Timothy Vilpas, Neha Patel, Johannes Maurer, Stefan Thum, Marcel Schmidt, Jens Langer, Sjoerd Harder*

Table of Contents

1. Materials and methods 2
2. Complex synthesis and Characterization 4
3. Reactivity studies 38
4. Crystal structure determination 52
5. DFT calculations 67
6. References 165

1. Materials and methods

All experiments were conducted in dry glassware under an inert nitrogen atmosphere by applying standard Schlenk techniques or gloveboxes (MBraun) using freshly dried and degassed solvents. All solvents were degassed with nitrogen, dried over activated aluminum oxide (Innovative Technology, Pure Solv 400-4-MD, Solvent Purification System), and then stored under inert atmosphere over molecular sieves (3 Å) unless noted otherwise. Deuterated benzene (C_6D_6), cylcohexane-d_{12}, methylcyclohexane-d_{14} and THF-d_8 were purchased from Sigma Aldrich or Deutero GmbH and dried over molecular sieves (3 Å). The following compounds were synthesized according to literature procedures: benzyl potassium,[S1] (DIPeP)NH_2,[S2] [(BDI*)SrI]$_2$,[S3] CaI_2/SrI_2.[S4]

The following reagents were obtained commercially and used without further purification: *n*-butyllithium (*n*BuLi (2.5 M in hexane, Sigma Aldrich)), 1,2-bis(chlorodimethylsilyl)ethane (96%, Sigma Aldrich), poly(tetrafluoroethylene) (PTFE) powder (1 µm particle size, Sigma Aldrich).

NMR spectra were measured on Bruker Avance III HD 400 MHz and Bruker Avance III HD 600 MHz spectrometers. Chemical shifts (δ) are denoted in ppm (parts per million), coupling constants in Hz (Hertz). For describing signal multiplicities common abbreviations are used: s (singlet), d (doublet), t (triplet), q (quartet), p (quintet), sept (septet), m (multiplet) and br (broad). Spectra were referenced to the solvent residual signal. Assignments of resonance signals in the ^{1}H and ^{13}C{^{1}H}/^{13}C-APT NMR spectra were made based on two-dimensional NMR correlation (HSQC, HMBC, COSY) experiments. Elemental analysis was performed with an Hekatech Eurovector EA3000 analyzer.
The ball-mill used was an ULTRA-TURRAX® Tube Drive P control from IKA. Mechanochemical reactions were performed at room temperature in 20 mL polypropylene vessel with three stainless steel balls (diameter: 5 mm, weight: 0.52 g, type: AISI 304) without temperature control.

Reactions under microwave irradiation were carried out in a closed vessel in a Biotage Initiator+ device.

All crystal structures have been measured on a SuperNova (Agilent) diffractometer with dual Cu and Mo microfocus sources and an Atlas S2 detector. Crystallographic data have been deposited with the Cambridge Crystallographic Data Centre as supplementary publication numbers: 2424565 [(BDI*-H)Sr]$_2$(THF)$_2$ (**2**-THF), 2424566 (BDI*)$_2$Sr(THF)$_2$ (**3**-THF), 2424567 (DIPeP)N(H)Li·(THF)$_2$

2424568 (^{DIPeP}NN-H_2), 2424569 (^{DIPeP}NN)K_2(THF)$_2$ (**4**-THF), 2424570 (^{DIPeP}NN)Sr(THF)$_2$ (**5**-Sr-THF), 2424571 (^{DIPeP}NN)Ca (**5**-Ca), 2424572 [K(^{DIPeP}NN)Ca]$_2$(N_2) (**6**-Ca), 2424573 [K(^{DIPeP}NN)Sr]$_2$(N_2) (**6**-Sr), 2426832 [(^{DIPeP}NN)SrKF]$_2$ (**7**).

2. Complex synthesis and Characterization

Synthesis of (DIPeP)N(H)Li

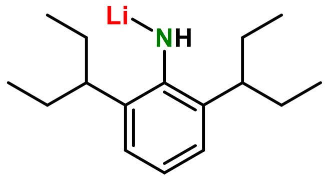

A stirred solution of (DIPeP)NH_2 (5.03 g, 21.4 mmol, 1 eq.) in hexanes (30 mL) was cooled to 0 °C and *n*BuLi in hexanes (2.5 M, 9.0 mL, 22.5 mmol, 1 eq.) was slowly added at this temperature. The suspension was stirred for 16 h allowing it to slowly reach room temperature. The light brown suspension was centrifuged and the solvent was decanted off. The remaining solids were washed with hexanes (2 x 10 mL) and dried *in vacuo* to obtain (DIPeP)N(H)Li (4.30 g, 18.0 mmol, 84%) as an off-white powder.

^{1}H-NMR (400.13 MHz, THF-d_8, 298 K): δ = 6.54-6.53 (m, 2H, *m*-C**H**$_{arom}$), 5.98 (m (br), 1H, *p*-C**H**$_{arom}$), 2.82-2.72 (m, 2H, C**H** *i*Pen), 2.73 (s, 1H, N**H**), 1.69-1.53 (m, 8H, C**H$_2$** *i*Pen), 0.86 (t, $^{3}J_{H,H}$ = 7.3 Hz, 12H, C**H$_3$** *i*Pen) ppm.
^{13}C-APT-NMR (100.62 MHz, THF-d_8, 298 K): δ = 160.6 (N-**C**), 127.9 (*o*-**C**$_{arom}$), 123.8 (*m*-**C**$_{arom}$), 108.4 (*p*-**C**$_{arom}$), 42.7 (**C**H *i*Pen), 28.2 (**C**H_2 *i*Pen), 12.7 (**C**H_3 *i*Pen) ppm.
Elemental Analysis: Calculated for $C_{20}H_{34}LiNO$ (M = 311.44 g/mol): N 4.50, C 77.13, H 11.00 %. Found: N 4.76, C 76.54, H 10.82 %.

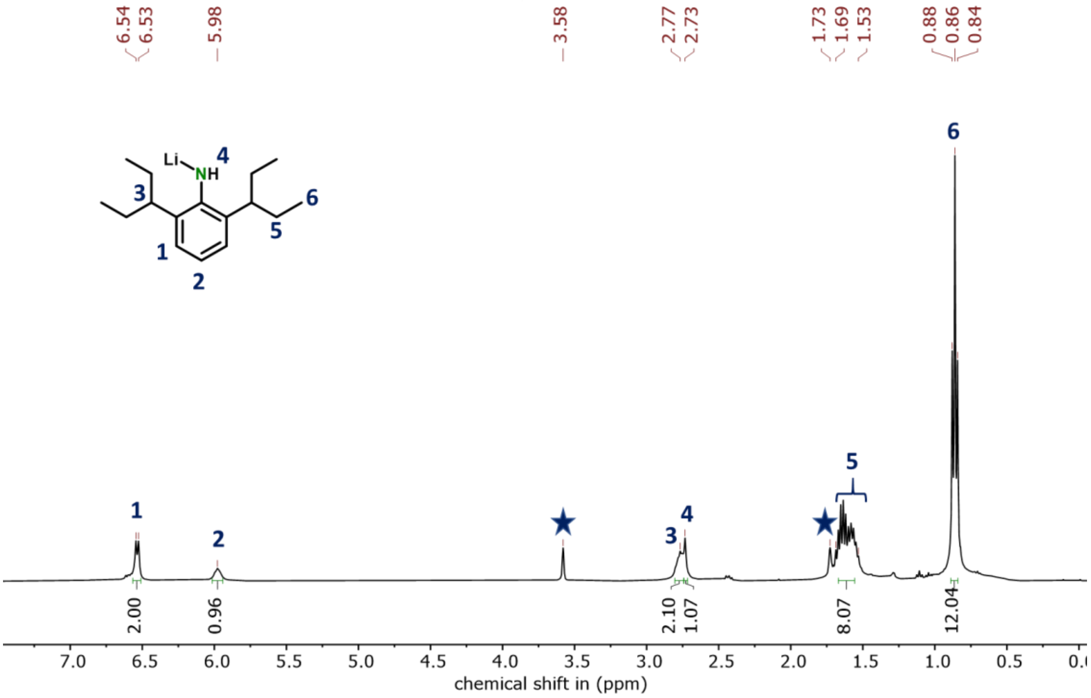

Figure S1: ^{1}H NMR (400 MHz, 298 K, THF-d_8) spectrum of (DIPeP)N(H)Li. Solvent residual signals are marked with a star.

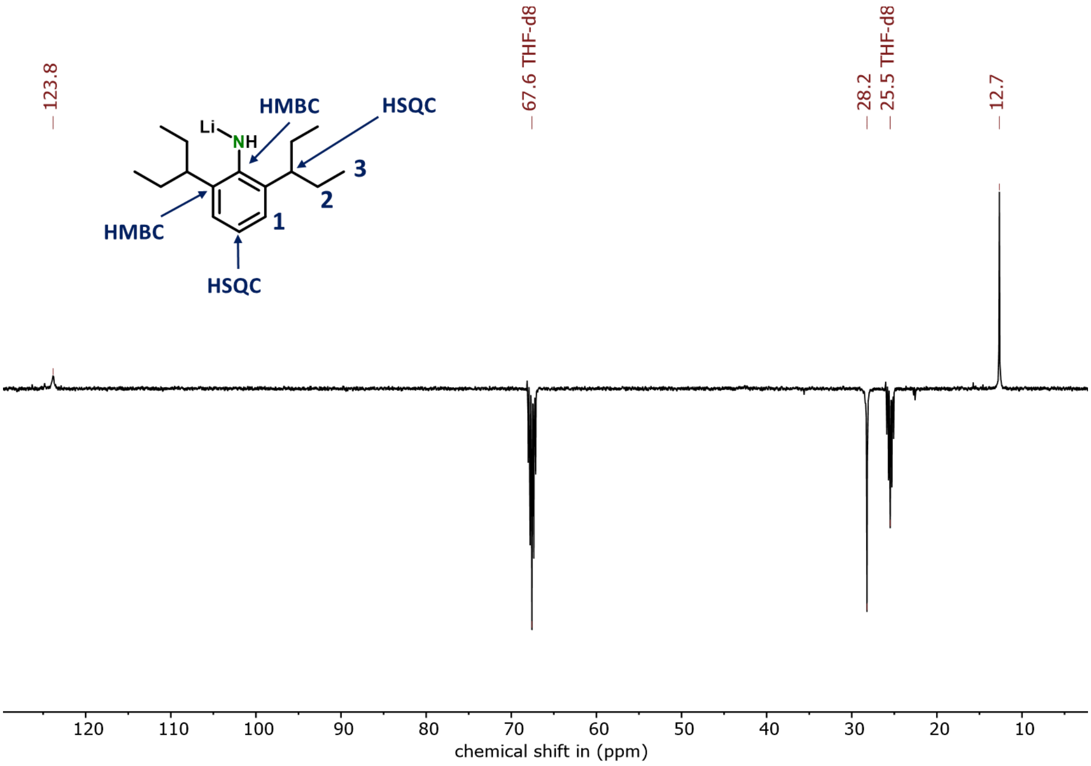

Figure S2: ^{13}C-APT NMR (101 MHz, 298 K, THF-d_8) spectrum of (DIPeP)N(H)Li.

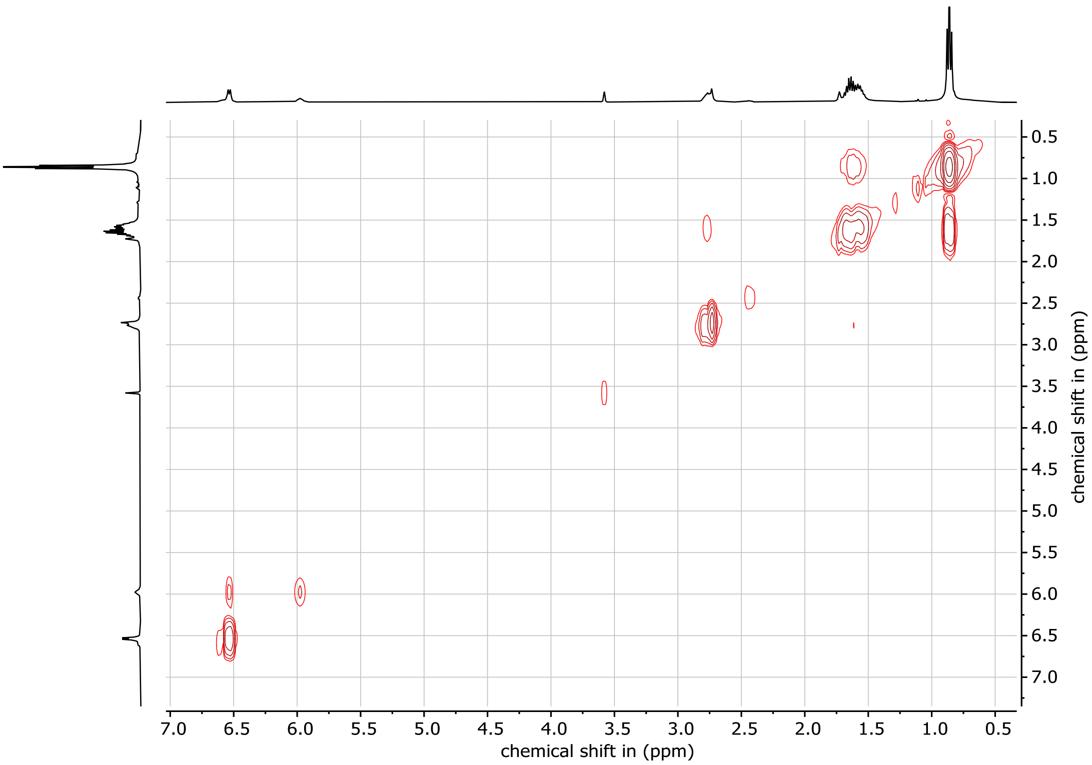

Figure S3: 2D-COSY NMR (400MHz, 298 K, THF-d_8) spectrum of (DIPeP)N(H)Li.

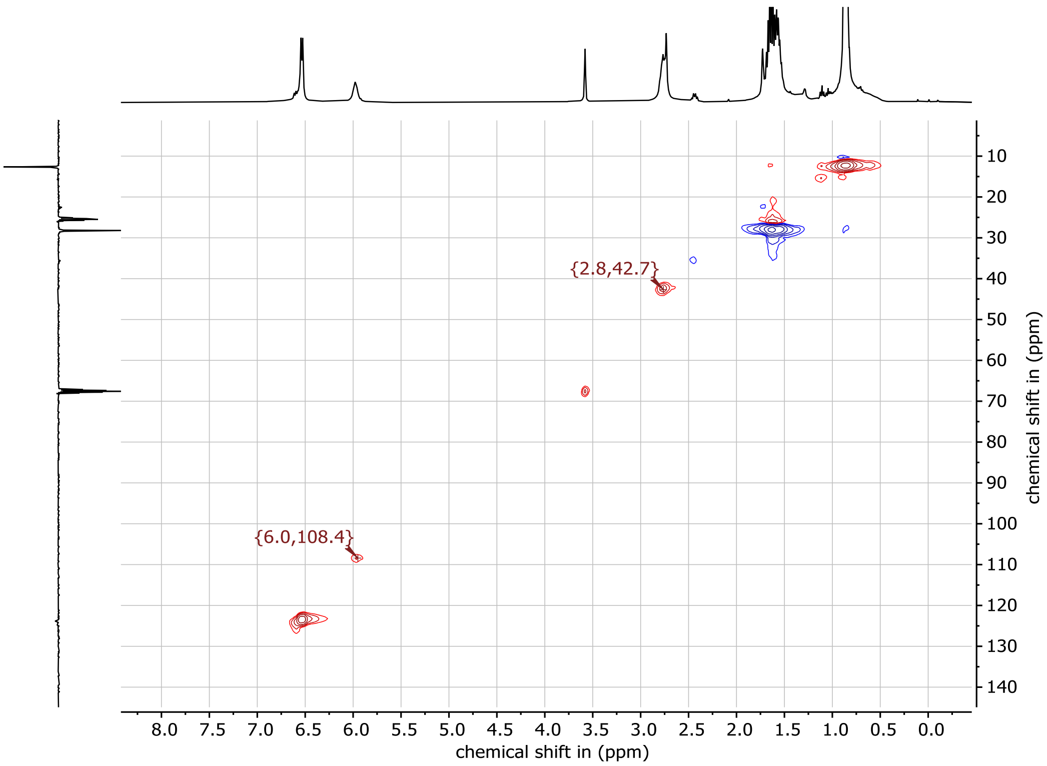

Figure S4: 2D-HSQC NMR (400/101 MHz, 298 K, THF-d_8) spectrum of (DIPeP)N(H)Li.

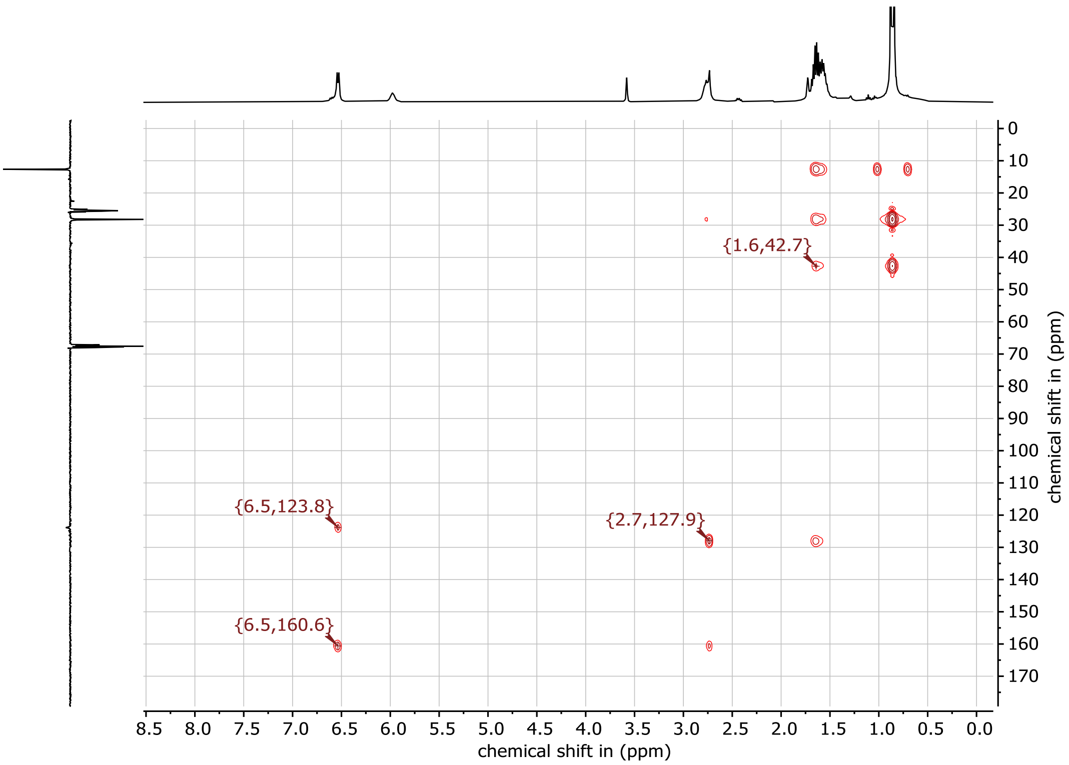

Figure S5: 2D-HMBC NMR (400/101 MHz, 298 K, THF-d_8) spectrum of (DIPeP)N(H)Li.

Synthesis of (^{DIPeP}NN-H$_2$)

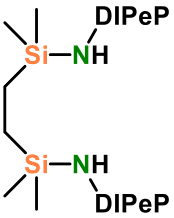

(DIPeP)N(H)Li (4.29 g, 18.0 mmol, 2 eq.) was dissolved in diethyl ether (40 mL) and cooled to 0 °C. A solution of 1,2-bis(chlorodimethylsilyl)ethane (1.93 g, 9.00 mmol, 1 eq.) in diethyl ether (11 mL) was slowly added. The resulting suspension was stirred overnight at 0 °C. The solvent was removed *in vacuo* and the residue was stripped with hexanes (6 mL). The obtained residue was dissolved in hexanes (12 mL) and then centrifuged. The clear, orange-brown liquid was decanted and the remaining solid was extracted with hexanes (2 x 4 mL). The combined liquids were dried *in vacuo*. (^{DIPeP}NN-H$_2$) was obtained as an essentially pure red brown oil (5.38 g, 8.83 mmol, 98%). Crystals suitable for single crystal X-ray diffraction were obtained by storing a concentrated solution of (^{DIPeP}NN-H$_2$) in hexanes at –30 °C.

^{1}H-NMR (400.13 MHz, C_6D_6, 298 K): δ = 7.11-7.07 (m, 2H, *p*-C**H**$_{arom}$), 7.01-6.99 (m, 4H, *m*-C**H**$_{arom}$), 3.17 (p, $^3J_{H,H}$ = 7.1 Hz, 4H, C**H** *i*Pen), 2.20 (s, 2H, N**H**), 1.77-1.65, 1.60-1.49 (two signals: m, 8H, C**H$_2$** *i*Pen), 0.91 (t, $^3J_{H,H}$ = 7.4 Hz, 24H, C**H$_3$** *i*Pen) 0.76 (s, 4H, Si-C**H$_2$**), 0.23 (s, 12H, Si-C**H$_3$**) ppm.

^{13}C-APT-NMR (100.62 MHz, C_6D_6, 298 K): δ = 142.8 (N-**C**), 142.1 (*o*-**C**$_{arom}$), 124.1 (*p*-**C**$_{arom}$), 124.0 (*m*-**C**$_{arom}$), 42.3 (**C**H *i*Pen), 29.2 (**C**H$_2$ *i*Pen), 12.5 (**C**H$_3$ *i*Pen), 10.3 (Si-**C**H$_2$), -1.2 (Si-**C**H$_3$) ppm.

Elemental Analysis: Calculated for $C_{38}H_{68}N_2Si_2$ (M = 609.15 g/mol): N 4.60, C 74.93, H 11.25 %. Found: N 4.71, C 74.99, H 11.15 %.

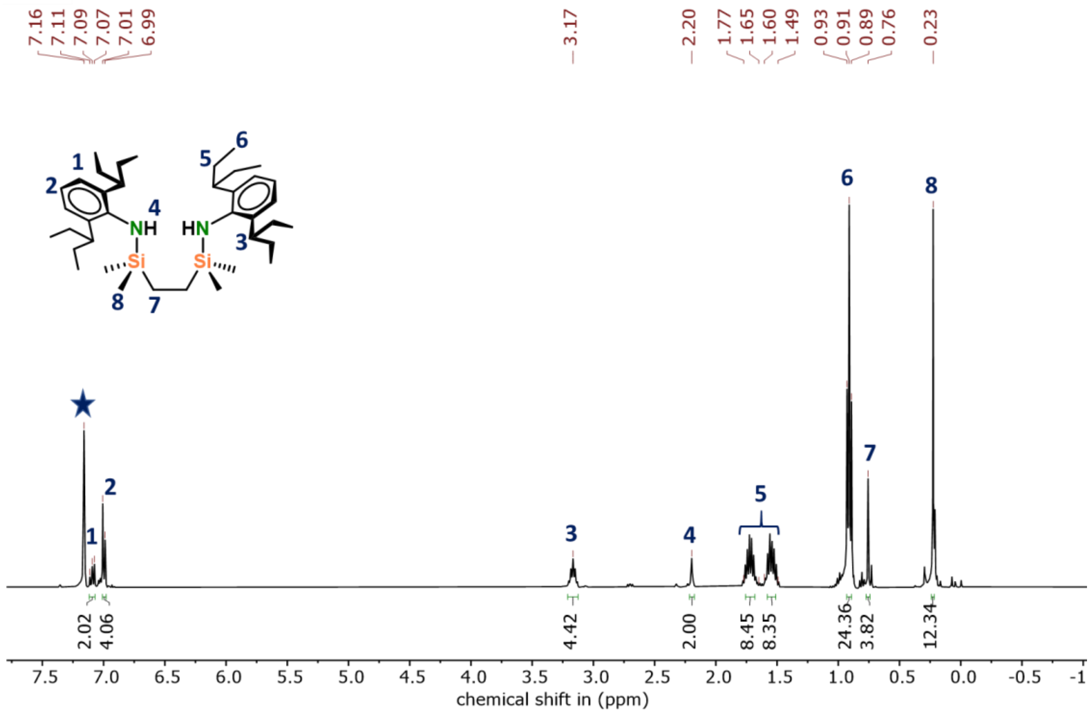

Figure S6: ^{1}H NMR (400 MHz, 298 K, C_6D_6) spectrum of (^{DIPeP}NN-H_2). Solvent residual signals are marked with a star.

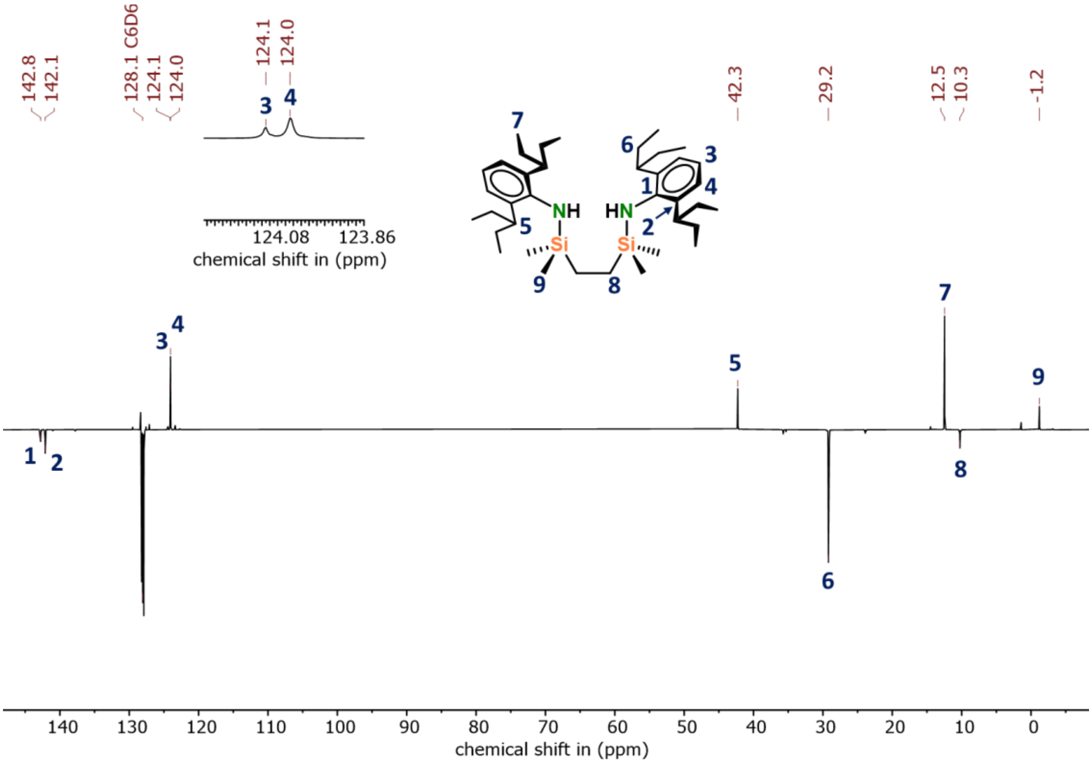

Figure S7: ^{13}C-APT NMR (151 MHz, 298 K, C_6D_6) spectrum of (^{DIPeP}NN-H_2).

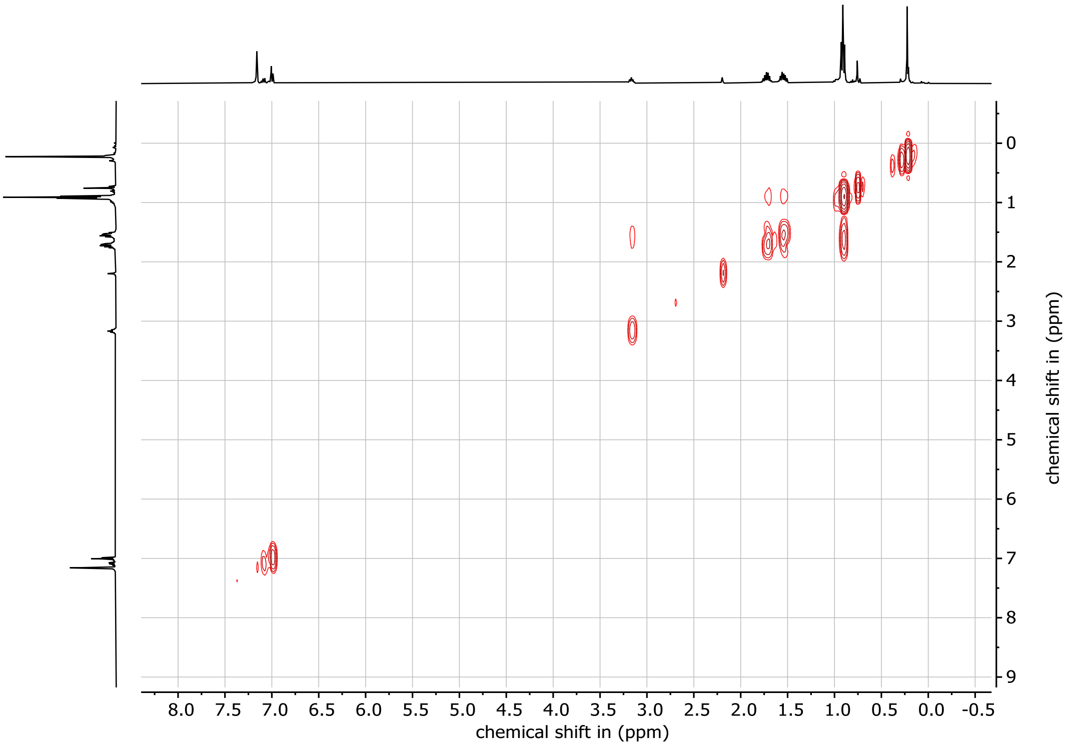

Figure S8: 2D-COSY NMR (600 MHz, 298 K, C_6D_6) spectrum of (^{DIPeP}NN-H_2).

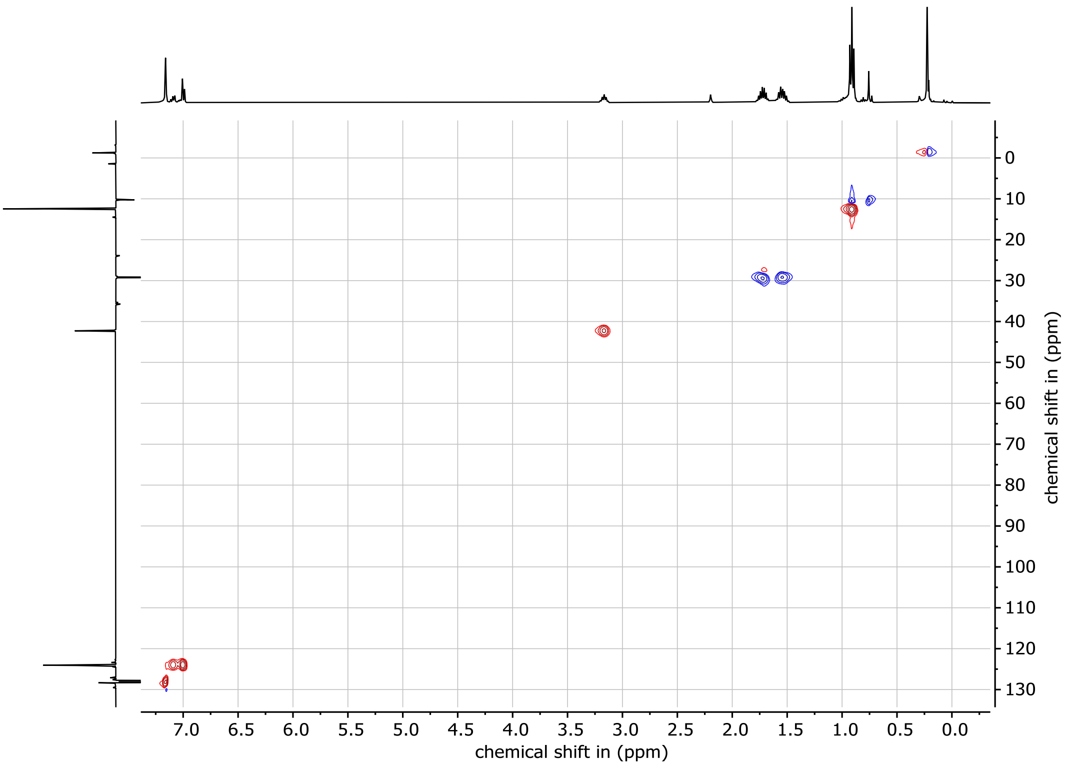

Figure S9: 2D-HSQC NMR (400/101 MHz, 298 K, C_6D_6) spectrum of (^{DIPeP}NN-H_2).

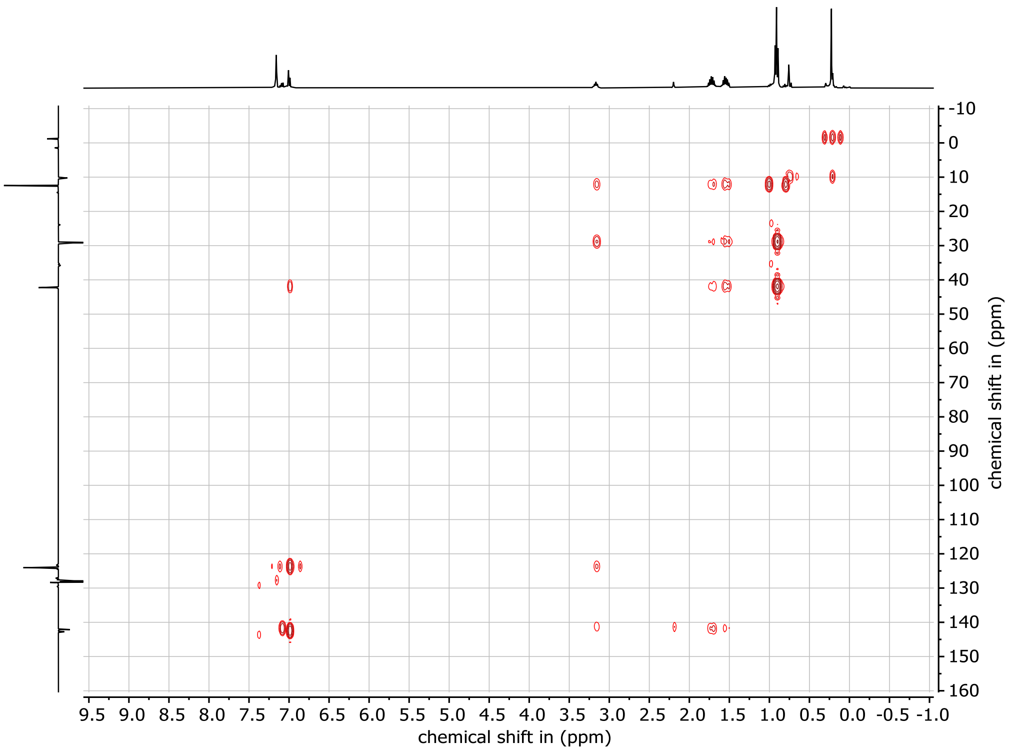

Figure S10: 2D-HMBC NMR (600/151MHz, 298 K, C_6D_6) spectrum of (^{DIPeP}NN-H_2).

Synthesis of (^{DIPeP}NN)K_2 (4)

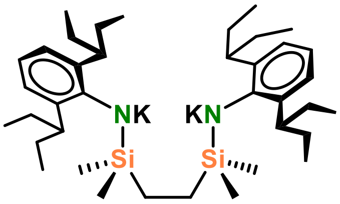

(^{DIPeP}NN-H_2) (2.00 g, 3.36 mmol) and benzyl potassium (876 mg, 6.72 mmol) were suspended in Et_2O (30 mL) and the mixture was stirred over-night. Subsequently, the solvent was removed *in vacuo* and the white solid was washed with hexane (40 mL). After drying at 55 °C for 2 h (^{DIPeP}NN)K_2 (**4**) was obtained as an essentially pure off-white powder (1.92 g, 2.76 mmol, 82%). The THF adduct (^{DIPeP}NN)K_2(THF)$_6$ was crystalized from a saturated solution of pentane and THF (10:1).

^{1}H-NMR (600.13 MHz, THF-d_8, 298 K): δ = 6.51 (d, $^3J_{H,H}$ = 7.3 Hz, 4H, *m*-C**H**$_{arom}$), 5.99 (t, $^3J_{H,H}$ = 7.3 Hz, 2H, *p*-C**H**$_{arom}$), 3.66 (p, $^3J_{H,H}$ = 7.0 Hz, 4H, C**H** *i*Pen), 1.53-1.46 (m, 8H, C**H**$_2$ *i*Pen), 1.43-1.35 (m, 8H, C**H**$_2$ *i*Pen), 0.81 (t, $^3J_{H,H}$ = 7.3 Hz, 24H, C**H**$_3$ *i*Pen), 0.66 (s, 4H, C**H**$_2$ backbone), -0.04 (s, 12H, C**H**$_3$ backbone) ppm.

^{13}C{^{1}H}-NMR (151.62 MHz, THF-d_8, 298 K): δ = 156.3 (**C**-N), 134.5 (*o*-**C**$_{arom}$), 120.3 (*m*-**C**H$_{arom}$), 106.4 (*p*-**C**H$_{arom}$), 37.3 (**C**H *i*Pen), 26.0 (**C**H$_2$ *i*Pen), 13.2 (**C**H$_2$ backbone), 9.7 (**C**H$_3$ *i*Pen), 0.9 (Si-**C**H$_3$) ppm.

^{29}SI/^{1}H-HSQC NMR (119.22 MHz, THF-d_8, 298 K): -18.8 ppm.

Elemental Analysis: Calculated for $C_{38}H_{66}N_2Si_2K_2$ (M = 685.33 g/mol): N 4.09, C 66.60, H 9.71 %. Found: N 4.27, C 66.10, H 9.45 %.

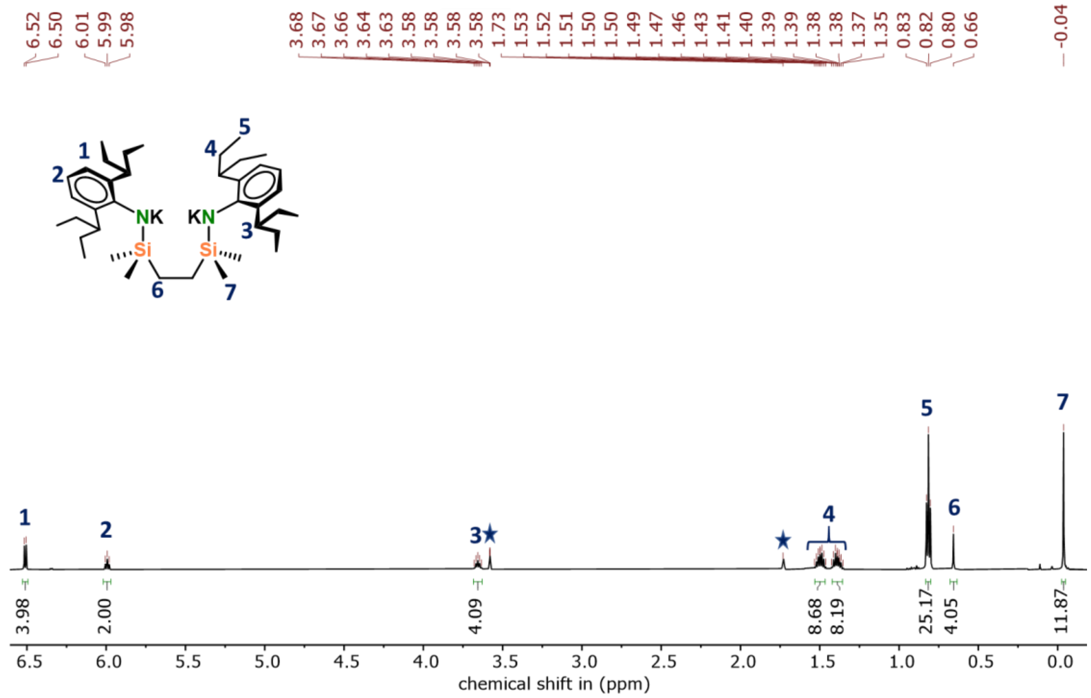

Figure S11: ^{1}H NMR (600 MHz, 298 K, THF-d_8) spectrum of (^{DIPeP}NN)K_2. Solvent residual signals are marked with a star.

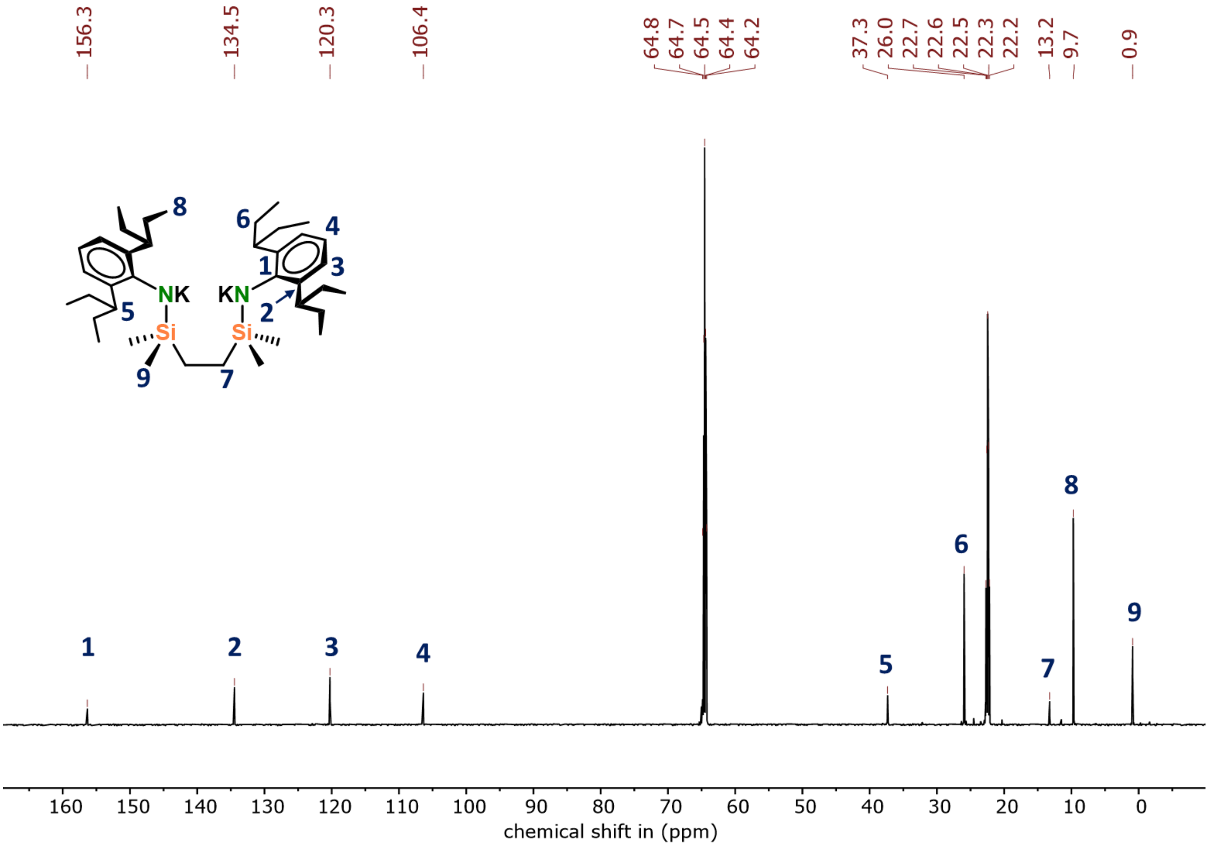

Figure S12: ^{13}C NMR (150 MHz, 298 K, THF-d_8) spectrum of (^{DIPeP}NN)K_2.

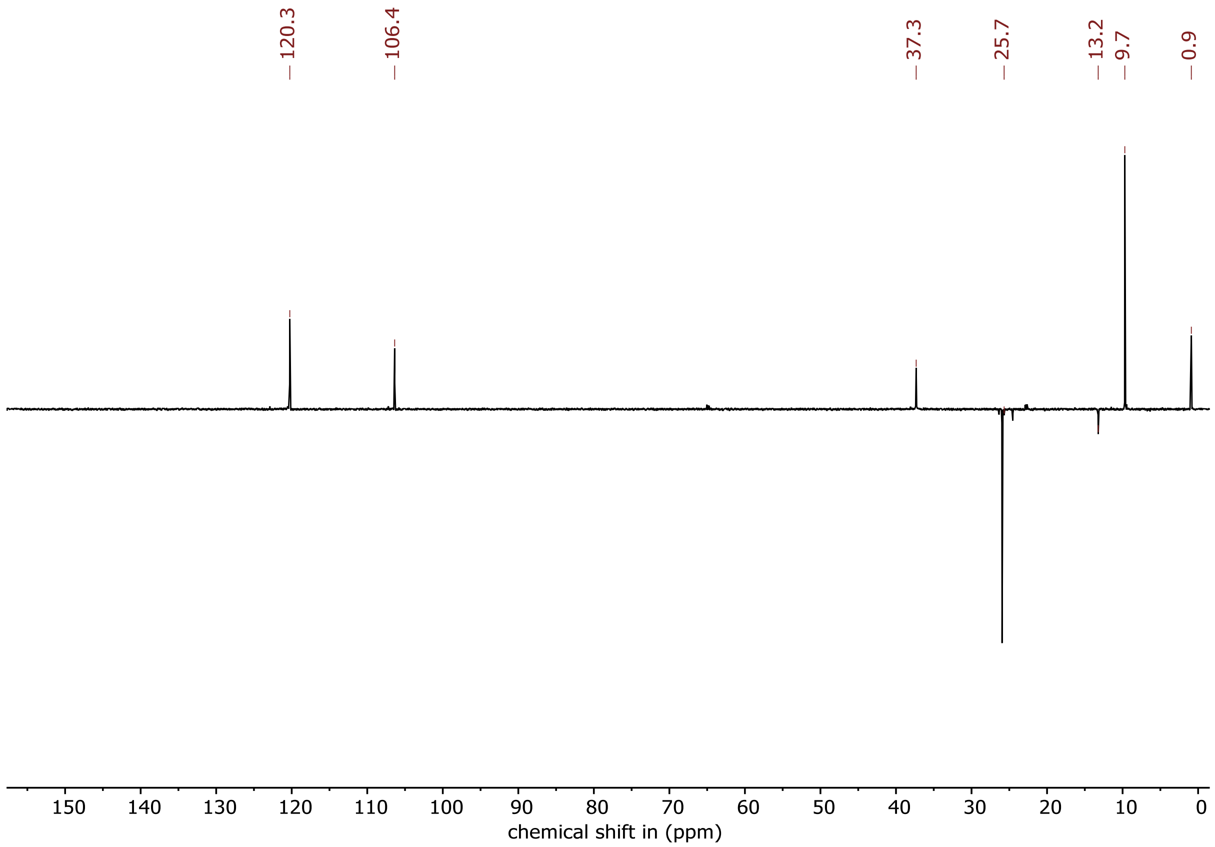

Figure S13: DEPT-135 NMR (150 MHz, 298 K, THF-d_8) spectrum of (^{DIPeP}NN)K_2.

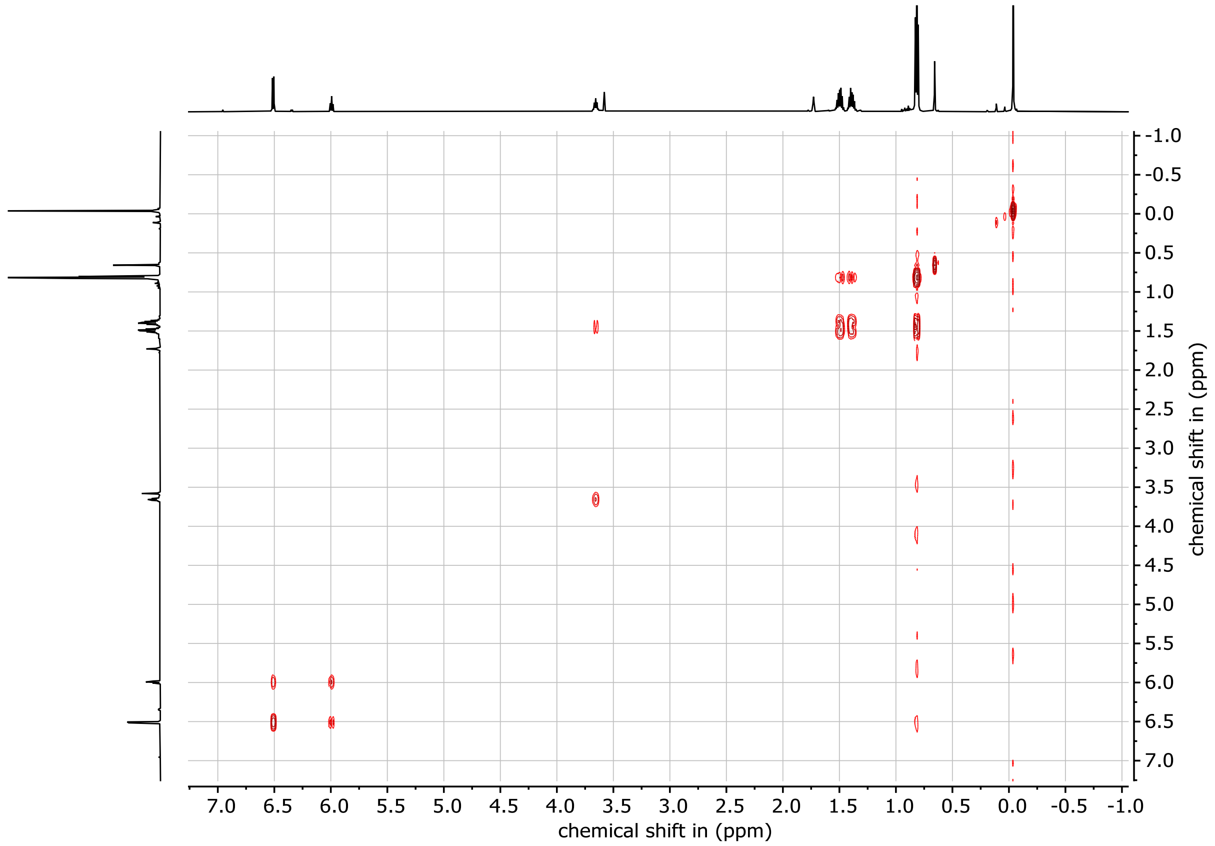

Figure S14: 2D-COSY NMR (600 MHz, 298 K, THF-d_8) spectrum of (^{DIPeP}NN)K_2.

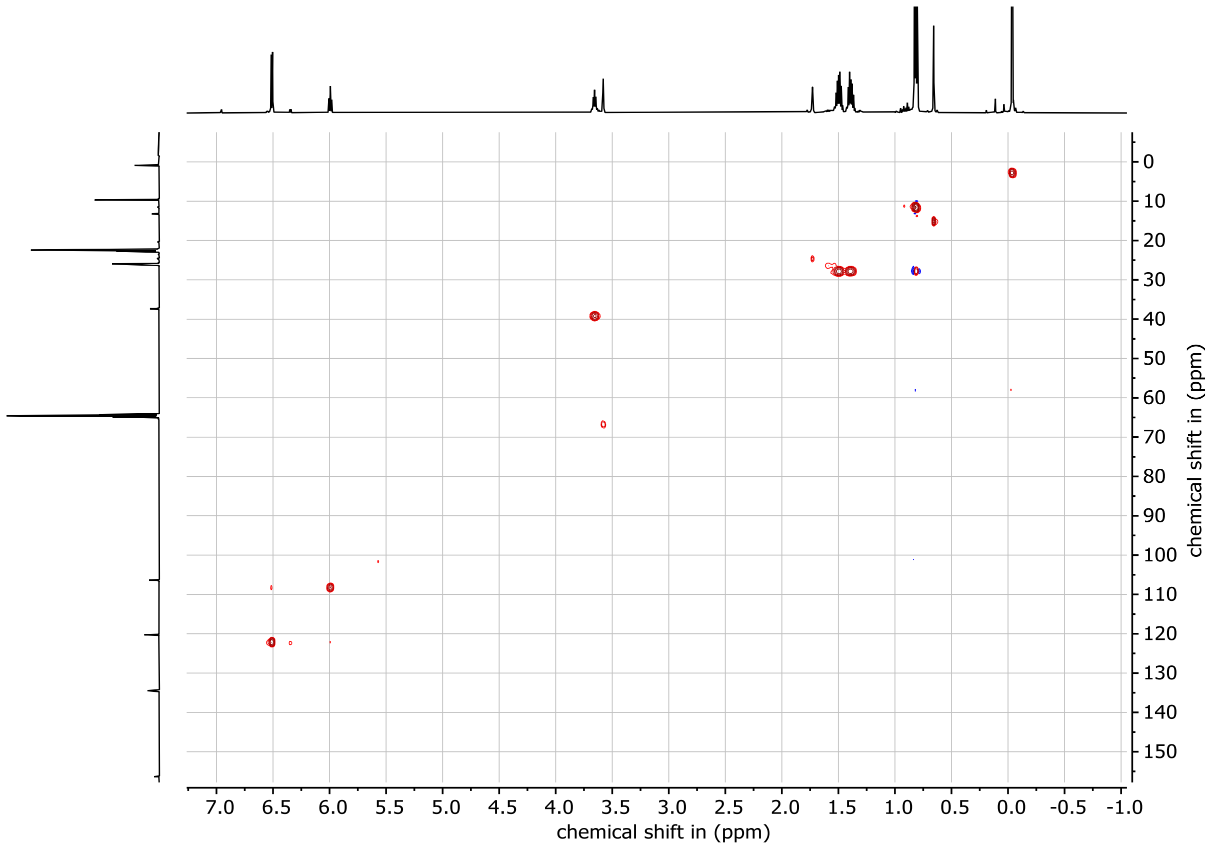

Figure S15: 2D-HSQC NMR (600/150 MHz, 298 K, THF-d_8) spectrum of (^{DIPeP}NN)K_2.

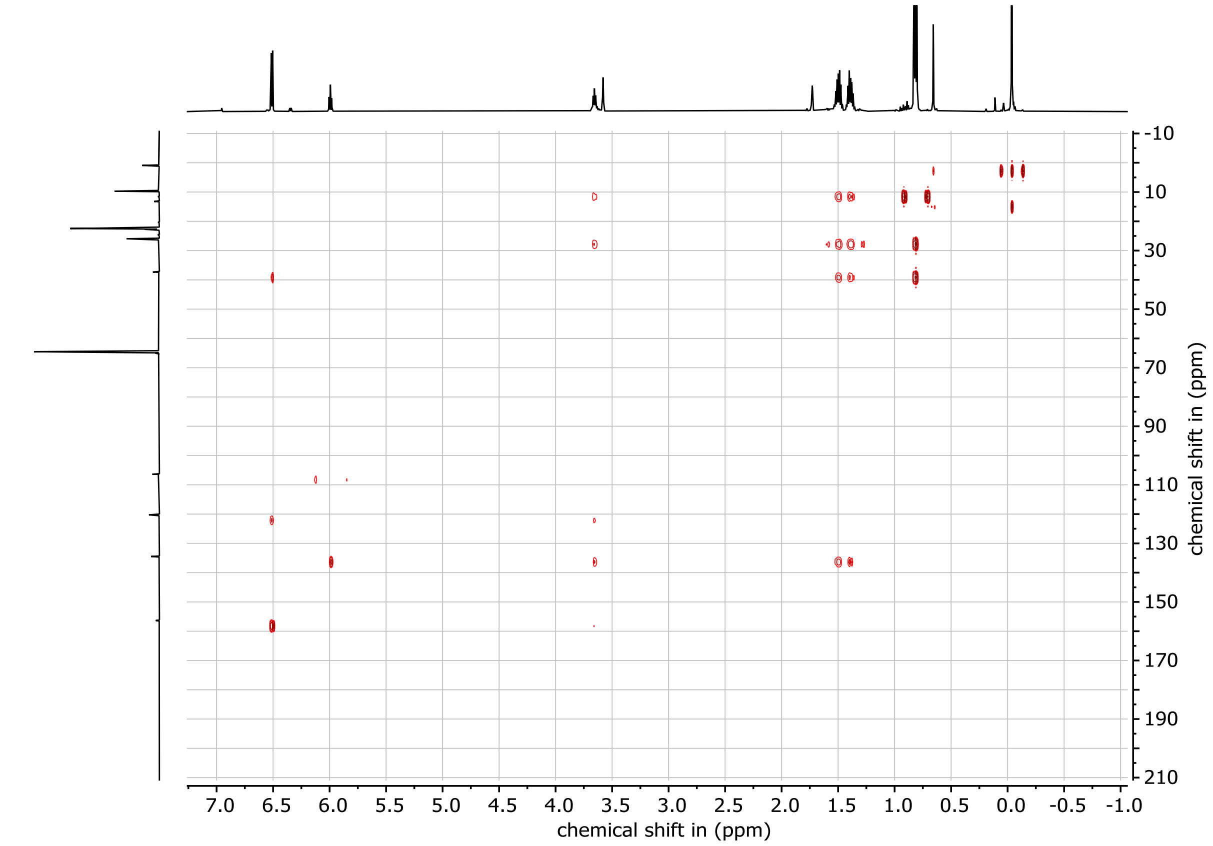

Figure S16: 2D-HMBCC NMR (600/150 MHz, 298 K, THF-d_8) spectrum of (^{DIPeP}NN)K_2.

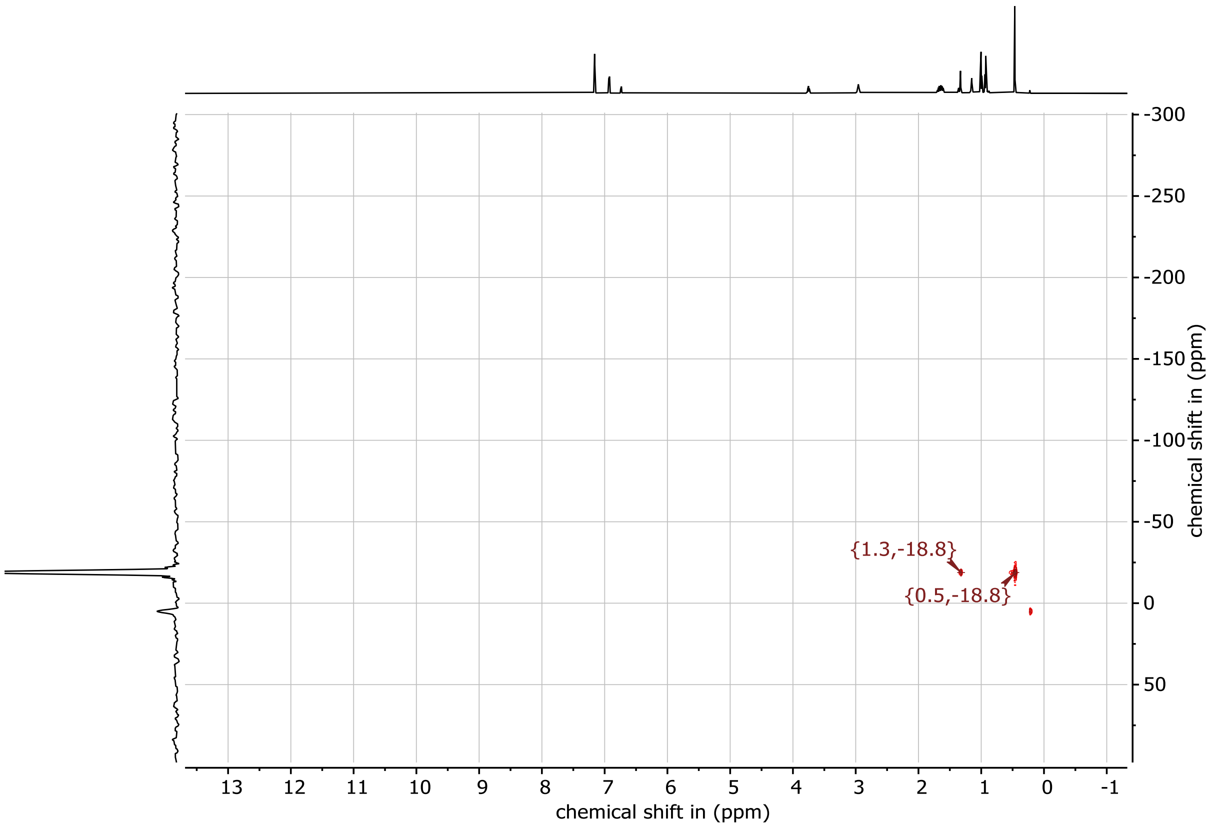

Figure S17: 2D-HMBC NMR (^{29}SI/H, 600/120 MHz, 298 K, THF-*d*$_8$) spectrum of (^{DIPeP}NN)K_2.

Synthesis of (^{DIPeP}NN)Ca (5-Ca)

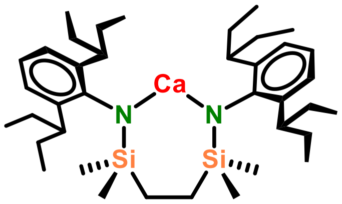

A microwave reaction vessel was charged with (^{DIPeP}NN)K_2 (1.00 g, 1.46 mmol), CaI_2 (450 mg, 1.53 mmol, 1.05 eq.) and benzene (12 mL). Subsequently, the vessel was placed in a microwave and the reaction mixture was heated to 150 °C for 1 h. After cooling to room-temperature the solvent was removed *in vacuo* and the residue was extracted with pentane (10 mL). Removing the solvent *in vacuo* gave a brown oily residue, which was stripped with pentane (4 x 6 mL) and dried under vacuum at room-temperature for 1 h to obtain (^{DIPeP}NN)Ca (**5**-Ca) as an off-white powder, which was used without further purification (Yield: 870 mg, 1.34 mmol, 92%). Crystals suitable for X-ray diffraction analysis were obtained from a saturated solution of hexanes.

^{1}H-NMR (600.13 MHz, C_6D_6, 298 K): δ = 6.98 (d, $^{3}J_{H,H}$ = 7.2 Hz, 4H, *m*-C**H**$_{arom}$), 6.89 (t, $^{3}J_{H,H}$ = 7.5 Hz, 2H, *p*-C**H**$_{arom}$), 3.55 (m, $^{3}J_{H,H}$ = 6.8 Hz, 4H, C**H** *i*Pen), 1.73-1.42 (br, 18H, C**H**$_2$ *i*Pen), 1.26 (s, 4H, C**H**$_2$ backbone), 1.01-0.85 (br, 24H, C**H**$_3$ *i*Pen), 0.40 (s, 12H, C**H**$_3$ backbone) ppm.

^{13}C{^{1}H}-NMR (151.62 MHz, C_6D_6, 298 K): δ = 150.8 (**C**-N), 138.5 (*o*-**C**$_{arom}$), 123.3 (*m*-**C**H$_{arom}$), 116.0 (*p*-**C**H$_{arom}$), 37.6 (**C**H *i*Pen), 25.8 (**C**H$_2$ *i*Pen), 11.0 (**C**H$_2$ backbone), 10.6 (**C**H$_3$ *i*Pen), 9.3 (**C**H$_3$ *i*Pen), 0.2 (Si-**C**H$_3$) ppm.

^{29}SI/^{1}H-HSQC (119.22 MHz, C_6D_6, 298 K): -13.3 ppm.

Elemental Analysis: Calculated for $C_{38}H_{66}N_2Si_2Sr$ (M = 647.21 g/mol): N 4.33, C 70.52, H 10.28 %. Found: N 4.66, C 70.68, H 10.12 %.

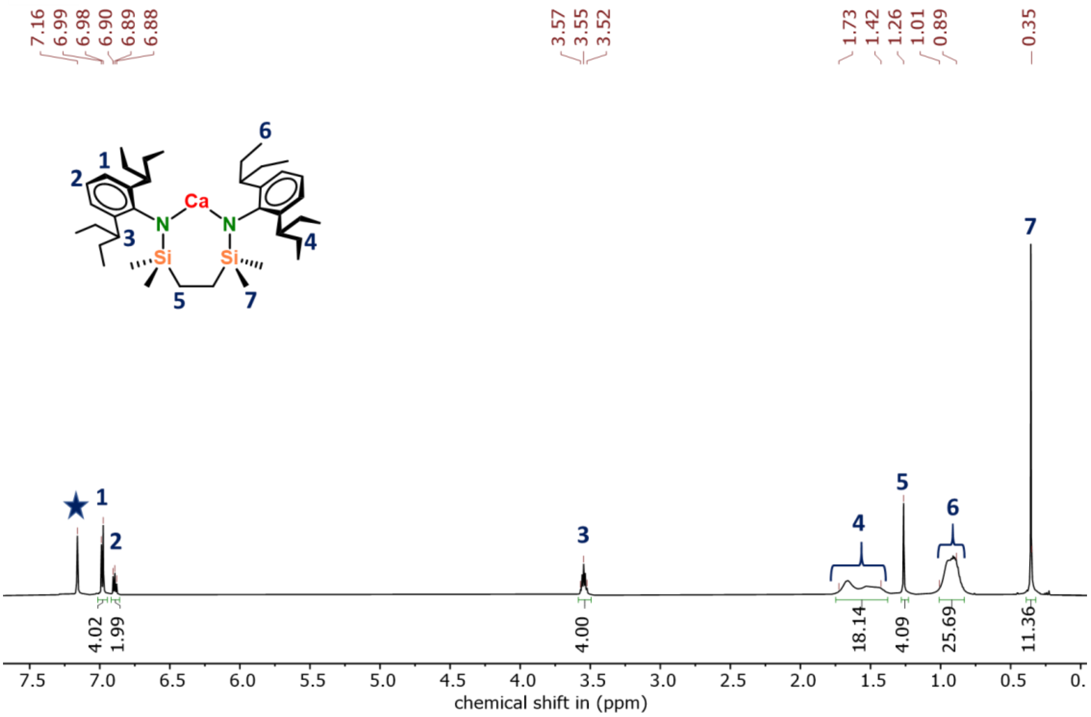

Figure S18: ^{1}H NMR (600 MHz, 298 K, C_6D_6) spectrum of (^{DIPeP}NN)Ca (**5**-Ca). Solvent residual signals are marked with a star.

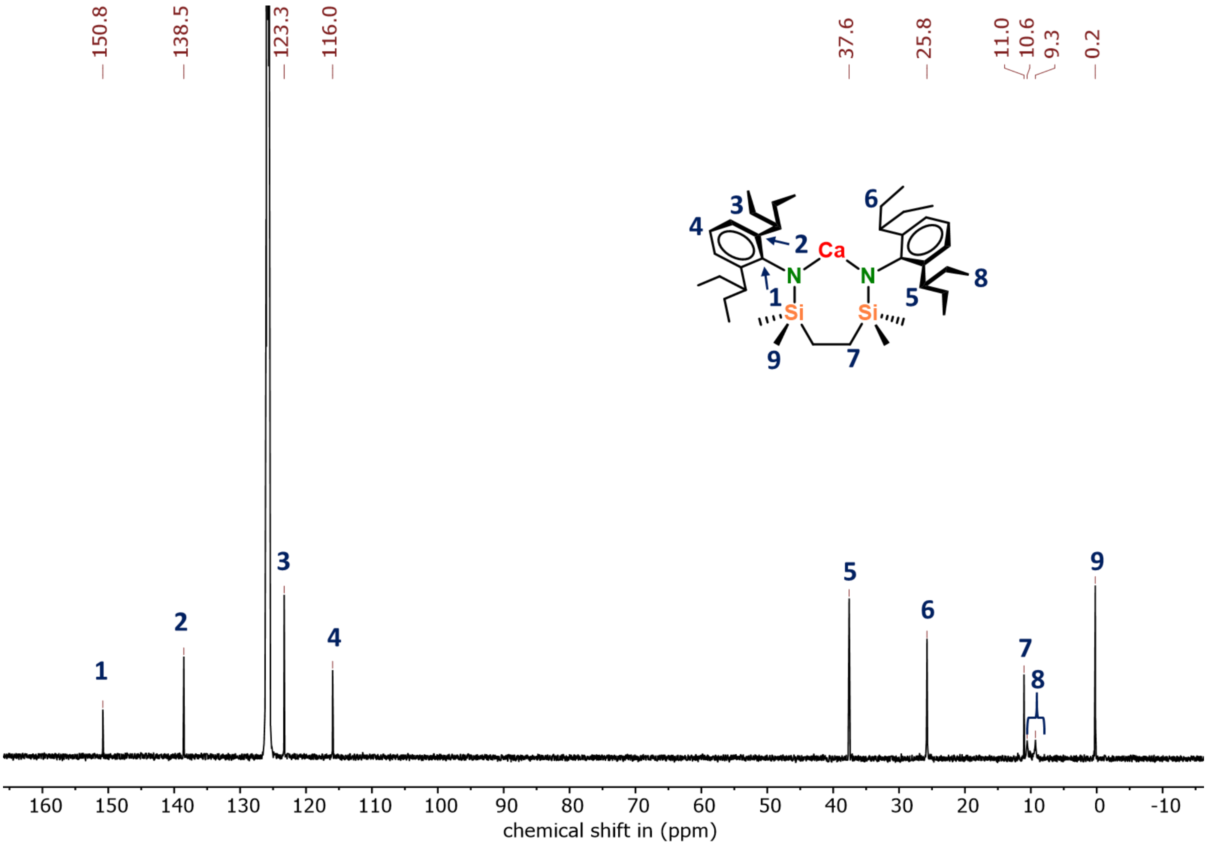

Figure S19: ^{13}C NMR (150 MHz, 298 K, C_6D_6) spectrum of (^{DIPeP}NN)Ca (**5**-Ca).

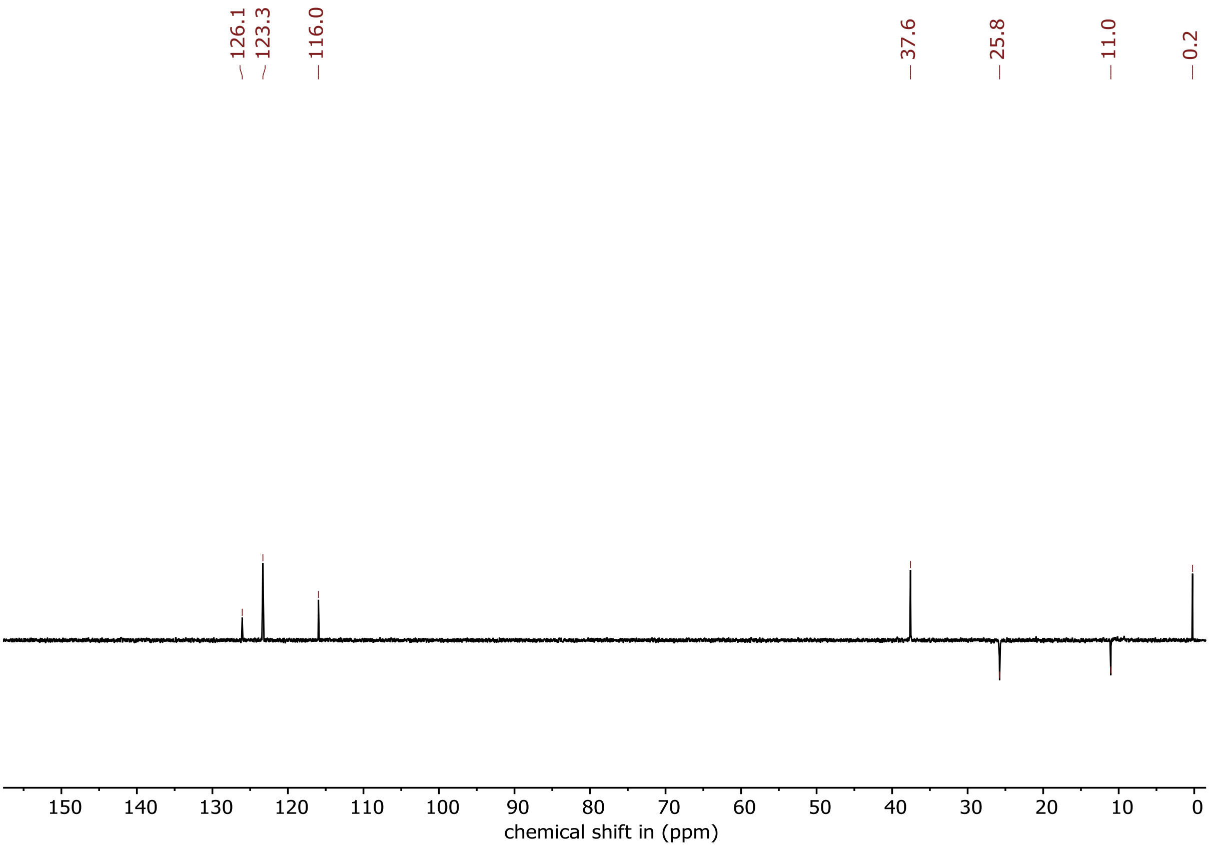

Figure S20: DEPT-135 NMR (150 MHz, 298 K, C_6D_6) spectrum of (^{DIPeP}NN)Ca (**5**-Ca).

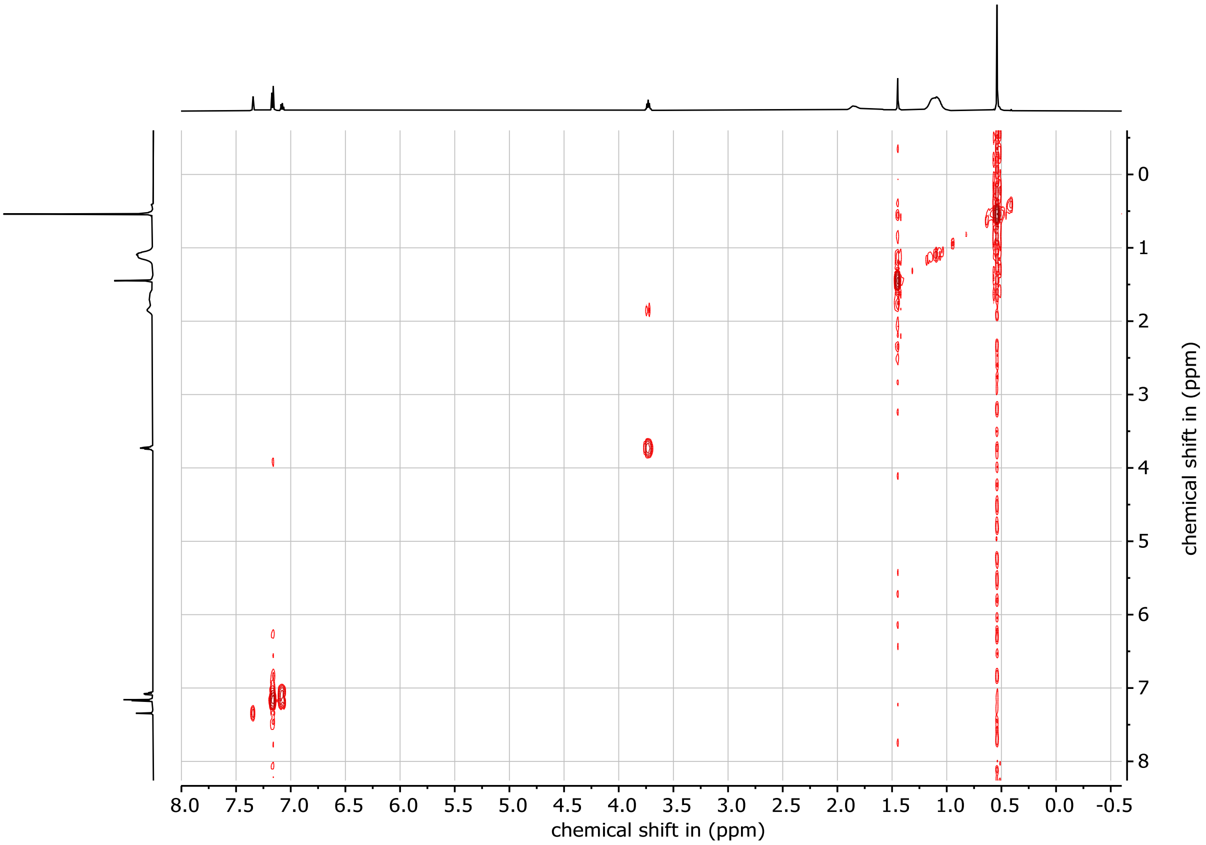

Figure S21: 2D-COSY NMR (600 MHz, 298 K, C_6D_6) spectrum of (^{DIPeP}NN)Ca (**5**-Ca).

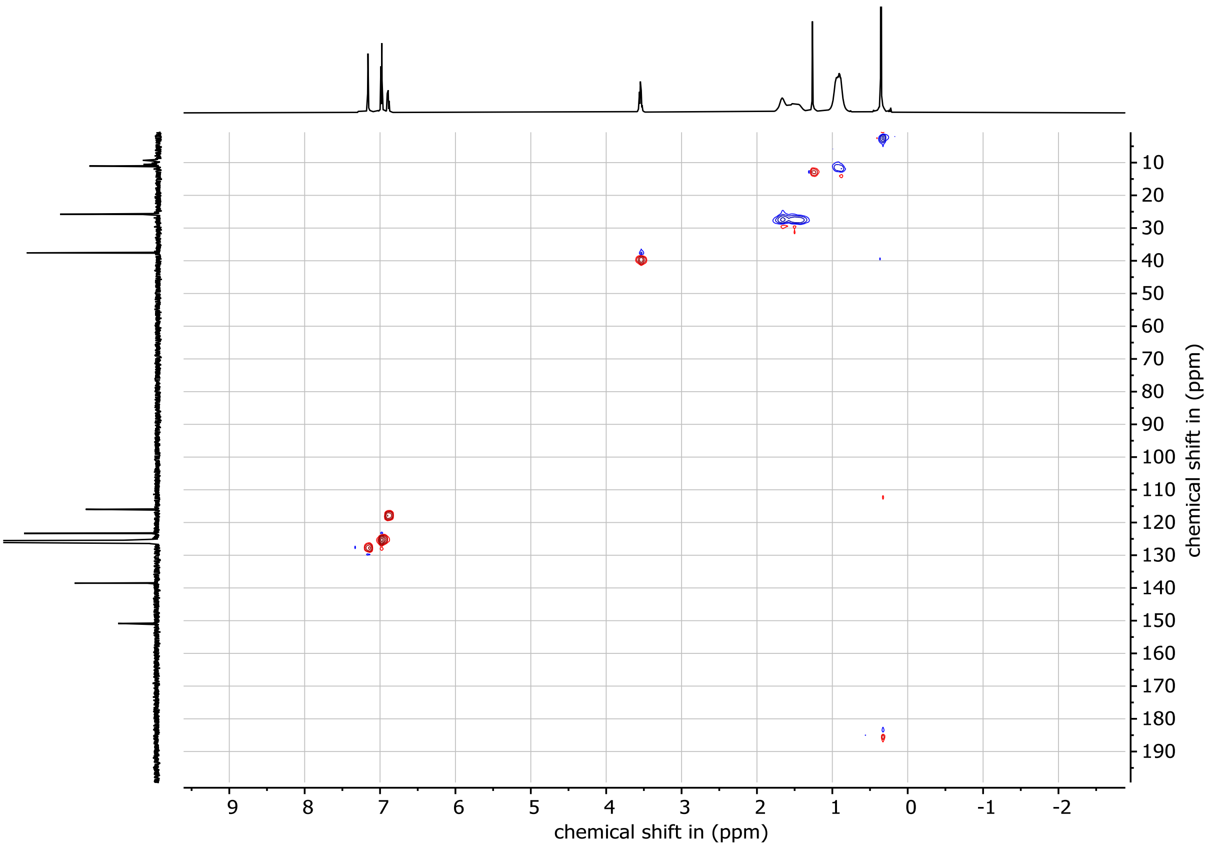

Figure S22: 2D-HSQC NMR (600/150 MHz, 298 K, C_6D_6) spectrum of ([DIPeP]NN)Ca (**5**-Ca).

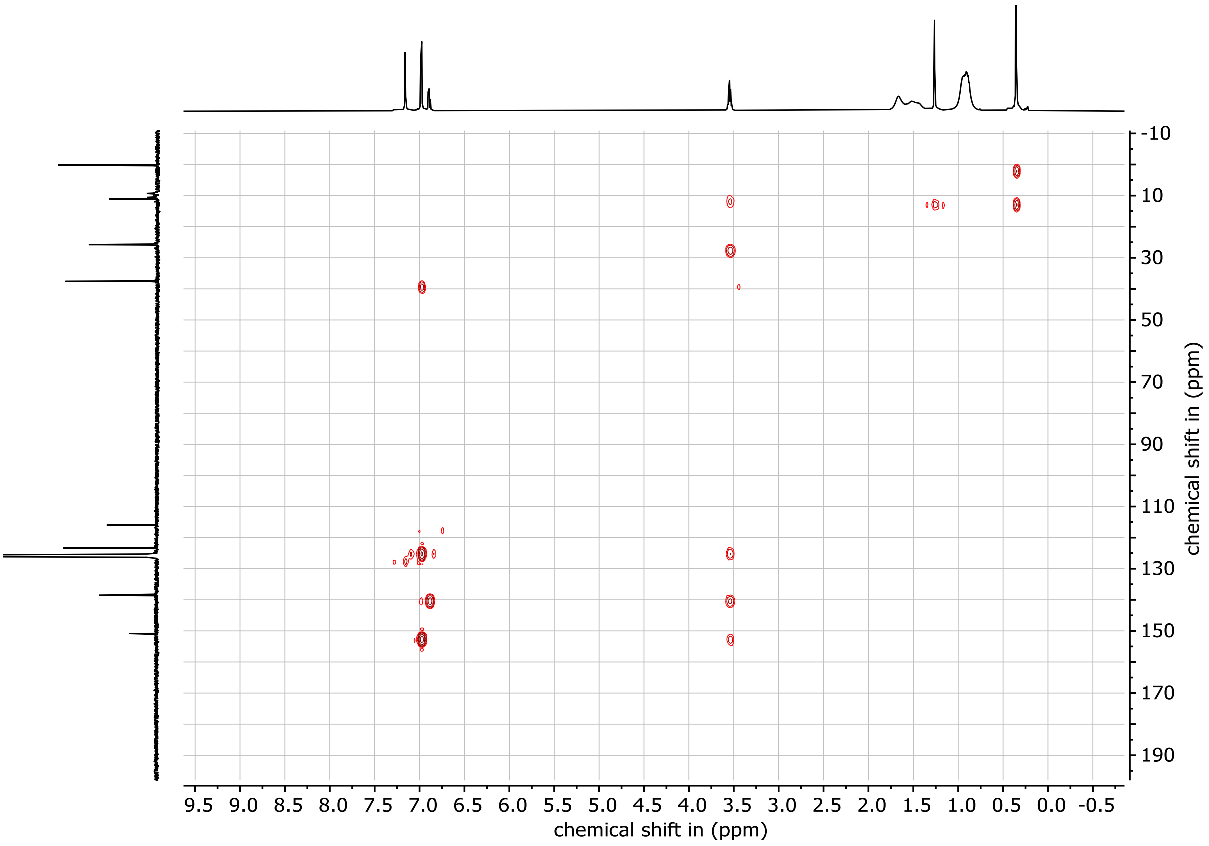

Figure S23: 2D-HMBC NMR (600/150 MHz, 298 K, C_6D_6) spectrum of ([DIPeP]NN)Ca (**5**-Ca).

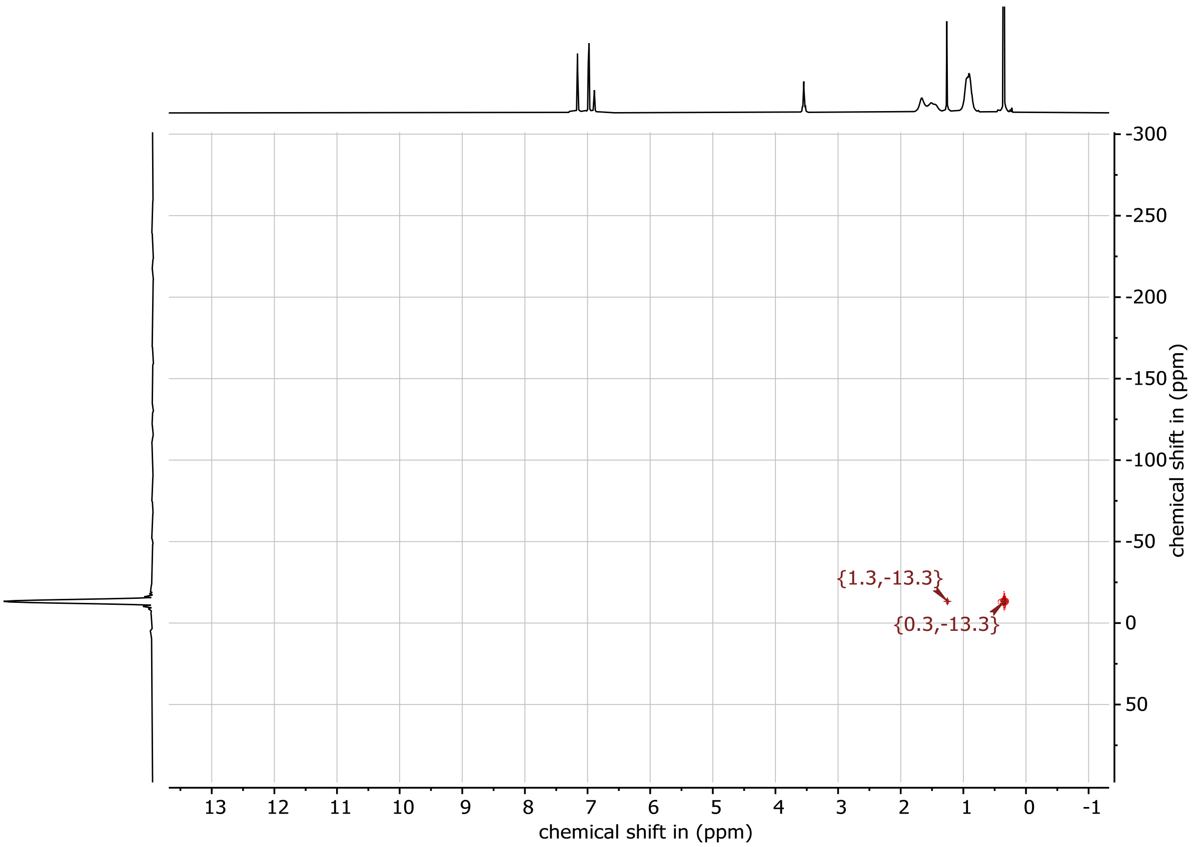

Figure S24: 2D-HMBC NMR (^{29}SI/H, 600/120 MHz, 298 K, C_6D_6) spectrum of (^{DIPeP}NN)Ca (**5**-Ca).

Synthesis of (^{DIPeP}NN)Sr (5-Sr)

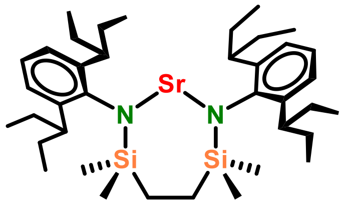

A microwave reaction vessel was charged with (^{DIPeP}NN)K_2 (1.00 g, 1.46 mmol), SrI_2 (523 mg, 1.53 mmol, 1.05 eq.) and benzene (12 mL). Subsequently, the vessel was placed in a microwave and the reaction mixture was heated to 150 °C for 1 h. After cooling to room-temperature the solvent was removed *in vacuo* and the residue was extracted with pentane (10 mL). Removing the solvent *in vacuo* gave a brown oily residue, which was stripped with pentane (4 x 6 mL) and dried under vaccum to obtain (^{DIPeP}NN)Sr (**5**-Sr) as an off-white powder, which was used without further purification (Yield: 815 mg, 1.23 mmol, 84%). The THF adduct (^{DIPeP}NN)Sr(THF)$_2$ was crystallized from a mixture of pentane (800 µL) and THF (100 µL) at -35 °C. After 2 d the mother liquor was decanted, the crystals washed with cold pentane (1 mL) and briefly dried *in vacuo* to obtain the product as colorless block-like crystals (86 mg, 0.103 mg, 28%).

^{1}H-NMR of (^{DIPeP}NN)Sr (600.13 MHz, C_6D_6, 298 K): δ = 6.97 (d, $^3J_{H,H}$ = 7.5 Hz, 4H, *m*-C**H**$_{arom}$), 6.83 (t, $^3J_{H,H}$ = 7.5 Hz, 2H, *p*-C**H**$_{arom}$), 3.60 (m, 4H, C**H** *i*Pen), 1.71-1.59 (m, 8H, C**H**$_2$ *i*Pen), 1.56-1.50 (m, 4H, C**H**$_2$ *i*Pen), 1.41-1.34 (m, 4H, C**H**$_2$ *i*Pen), 1.23 (s, 4H, C**H**$_2$ backbone), 1.01 (t, $^3J_{H,H}$ = 7.4 Hz, 12H, C**H**$_3$ *i*Pen), 0.95 (t, $^3J_{H,H}$ = 7.4 Hz, 12H, C**H**$_3$ *i*Pen), 0.40 (s, 12H, C**H**$_3$ backbone) ppm.

^{13}C{^{1}H}-NMR of (^{DIPeP}NN)Sr (151.62 MHz, C_6D_6, 298 K): δ = 152.7 (**C**-N), 139.6 (*o*-**C**$_{arom}$), 124.4 (*m*-**C**H$_{arom}$), 115.8 (*p*-**C**H$_{arom}$), 38.5 (**C**H *i*Pen), 27.0 (**C**H$_2$ *i*Pen), 12.2 (**C**H$_2$ backbone), 10.2 (**C**H$_3$ *i*Pen), 2.0 (Si-**C**H$_3$) ppm.

^{29}SI/^{1}H-HSQC (119.22 MHz, C_6D_6, 298 K): -18.8 ppm.

^{1}H-NMR of (^{DIPeP}NN)Sr(THF)$_2$ (600.13 MHz, C_6D_6, 298 K): δ = 6.93 (d, $^3J_{H,H}$ = 7.5 Hz, 4H, *m*-C**H**$_{arom}$), 6.73 (t, $^3J_{H,H}$ = 7.5 Hz, 2H, *p*-C**H**$_{arom}$), 3.75 (p, $^3J_{H,H}$ = 7.2 Hz, 4H, C**H** *i*Pen), 2.94 (s (br), 8H, THF *α*-C**H**$_2$), 1.72 – 1.57 (m, 12H, C**H**$_2$ *i*Pen), 1.38 – 1.34 (m, 4H, C**H**$_2$ *i*Pen), 1.33 (s, 4H, C**H**$_2$ backbone), 1.15 (s (br), 8H, THF ß-C**H**$_2$), 1.00 (t, $^3J_{H,H}$ = 7.4 Hz, 12H, C**H**$_3$ *i*Pen), 0.93 (t, $^3J_{H,H}$ = 7.4 Hz, 12H, C**H**$_3$ *i*Pen), 0.47 (s, 12H, C**H**$_3$ backbone) ppm.

^{13}C{^{1}H}-NMR of (^{DIPeP}NN)Sr(THF)$_2$ (151.62 MHz, C_6D_6, 298 K): 152.8 (**C**-N), 139.8 (*o*-**C**$_{arom}$), 124.4 (*m*-**C**H$_{arom}$), 116.3 (*p*-**C**H$_{arom}$), 67.7 (THF *α*-**C**H$_2$), 39.5 (**C**H *i*Pen), 28.9 (**C**H$_2$ *i*Pen), 28.3 (**C**H$_2$ *i*Pen), 25.0 (THF *ß*-**C**H$_2$), 12.9 (**C**H$_3$ *i*Pen), 11.9 (**C**H$_2$ backbone), 11.5 (**C**H$_3$ *i*Pen), 3.1 (Si-**C**H$_3$) ppm.

^{29}SI/^{1}H-HSQC (119.22 MHz, C_6D_6, 298 K): -18.8 ppm.

Elemental Analysis: Calculated for $C_{46}H_{82}N_2O_2Si_2Sr$ (M = 838.96 g/mol): N 3.34, C 65.86, H 9.85 %. Found: N 3.36, C 66.09, H 9.85 %.

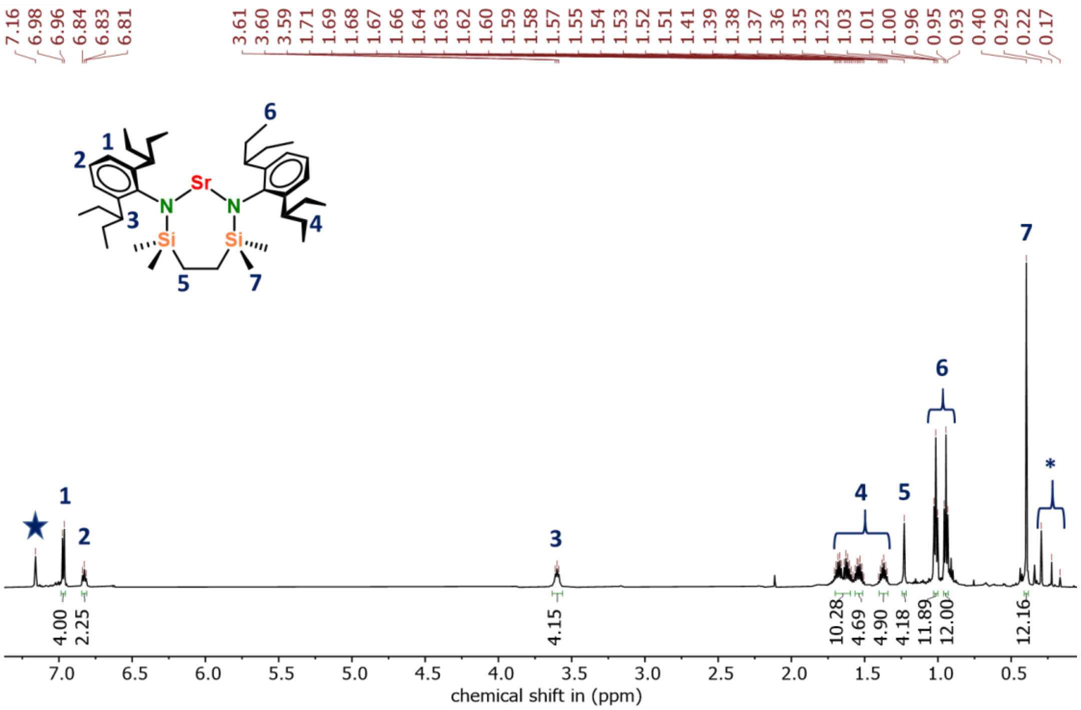

Figure S25: ^{1}H NMR (600 MHz, 298 K, C_6D_6) spectrum of (^{DIPeP}NN)Sr (**5**-Sr). Impurities are marked with an asterisk and solvent residual signals are marked with a star.

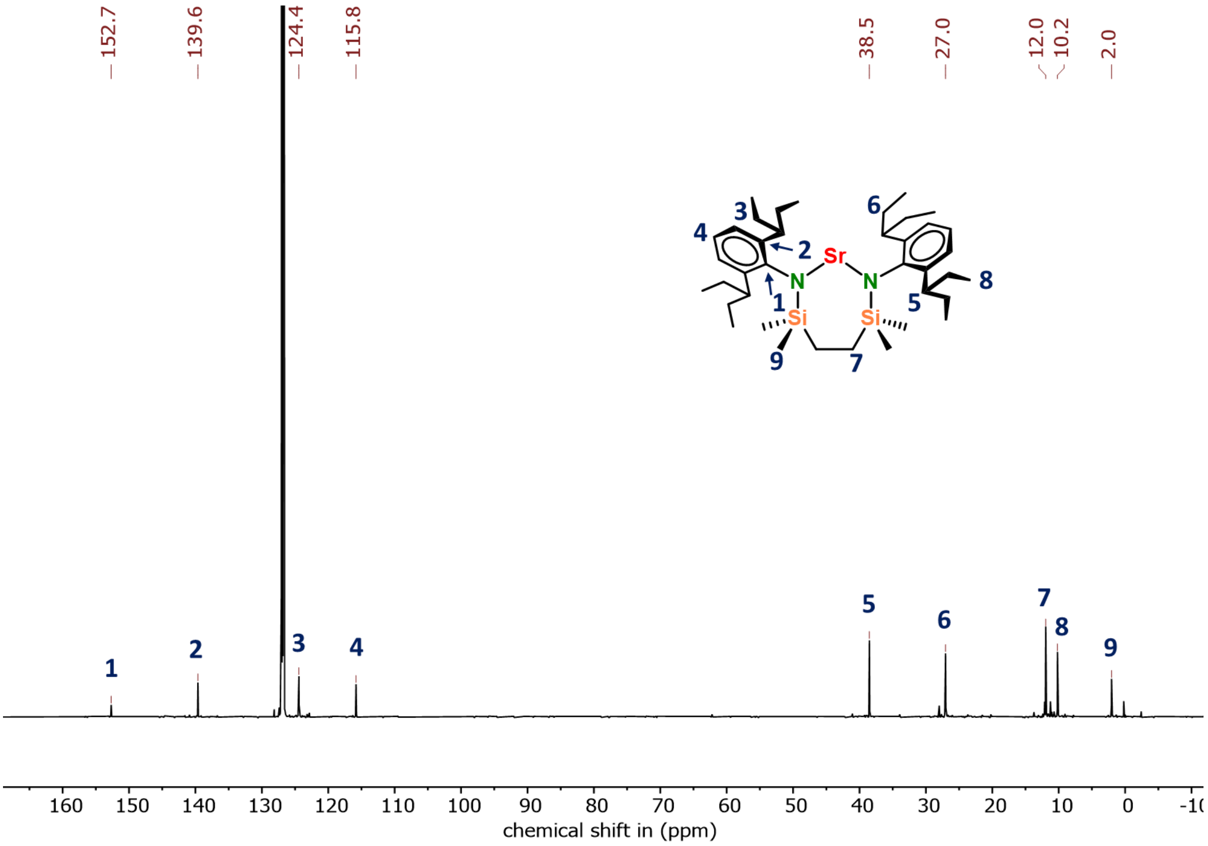

Figure S26: ^{13}C NMR (150 MHz, 298 K, C_6D_6) spectrum of (^{DIPeP}NN)Sr (**5**-Sr).

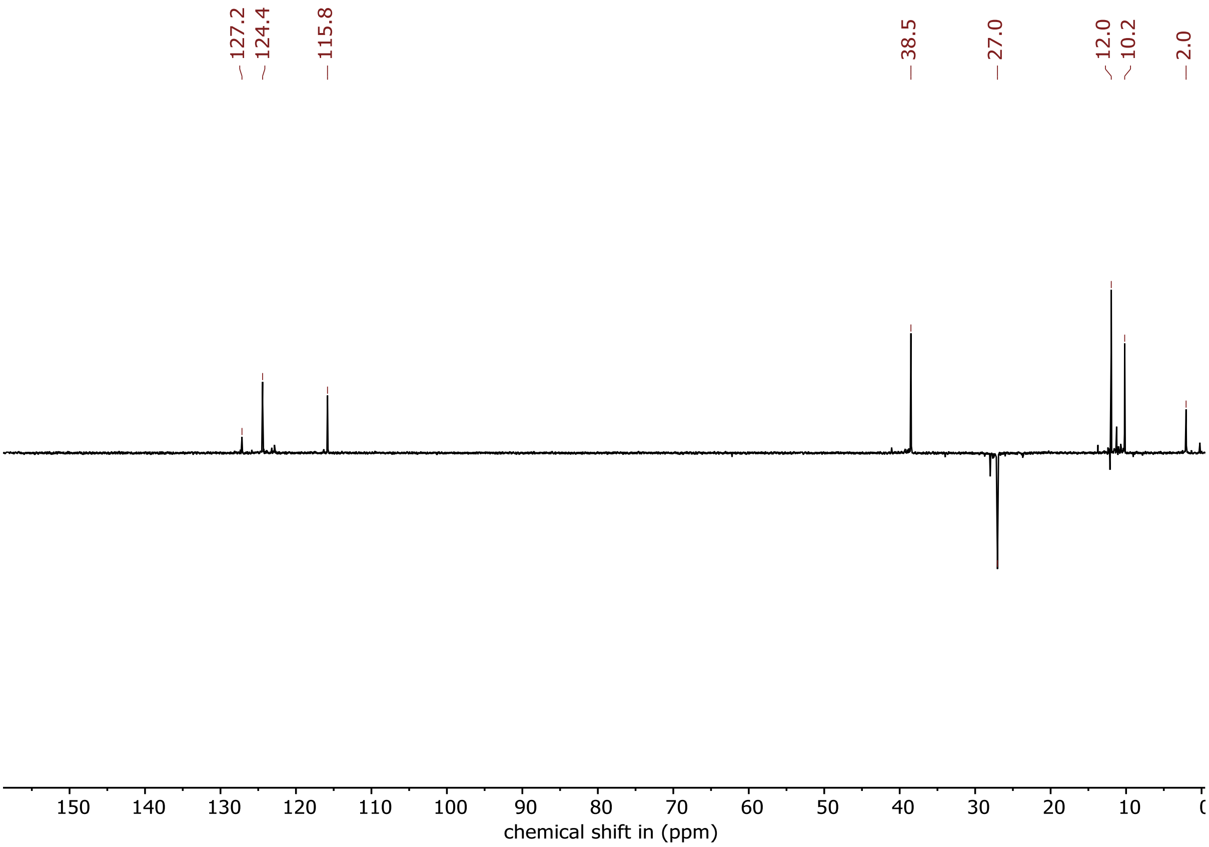

Figure S27: DEPT-135 NMR (150 MHz, 298 K, C_6D_6) spectrum of (^{DIPeP}NN)Sr (**5**-Sr).

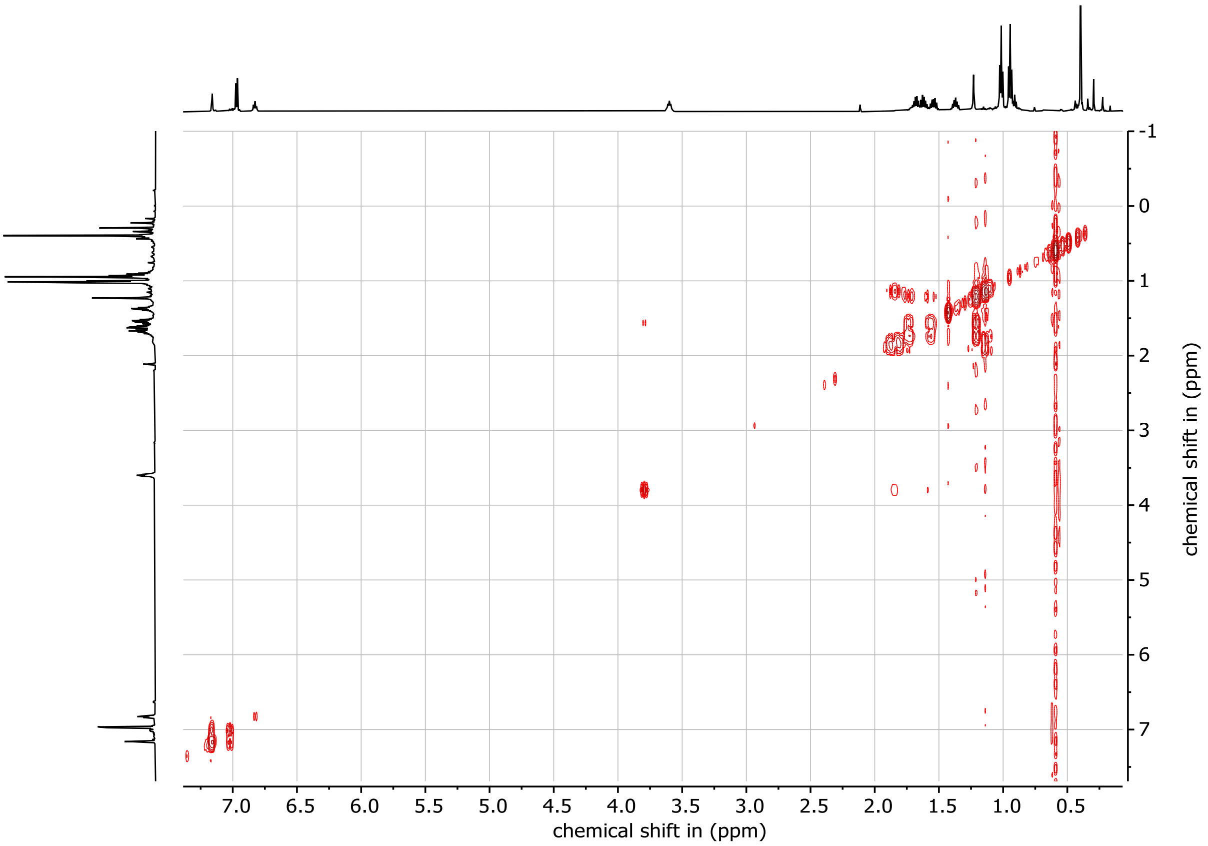

Figure S28: 2D-COSY NMR (600 MHz, 298 K, C_6D_6) spectrum of (^{DIPeP}NN)Sr (**5**-Sr).

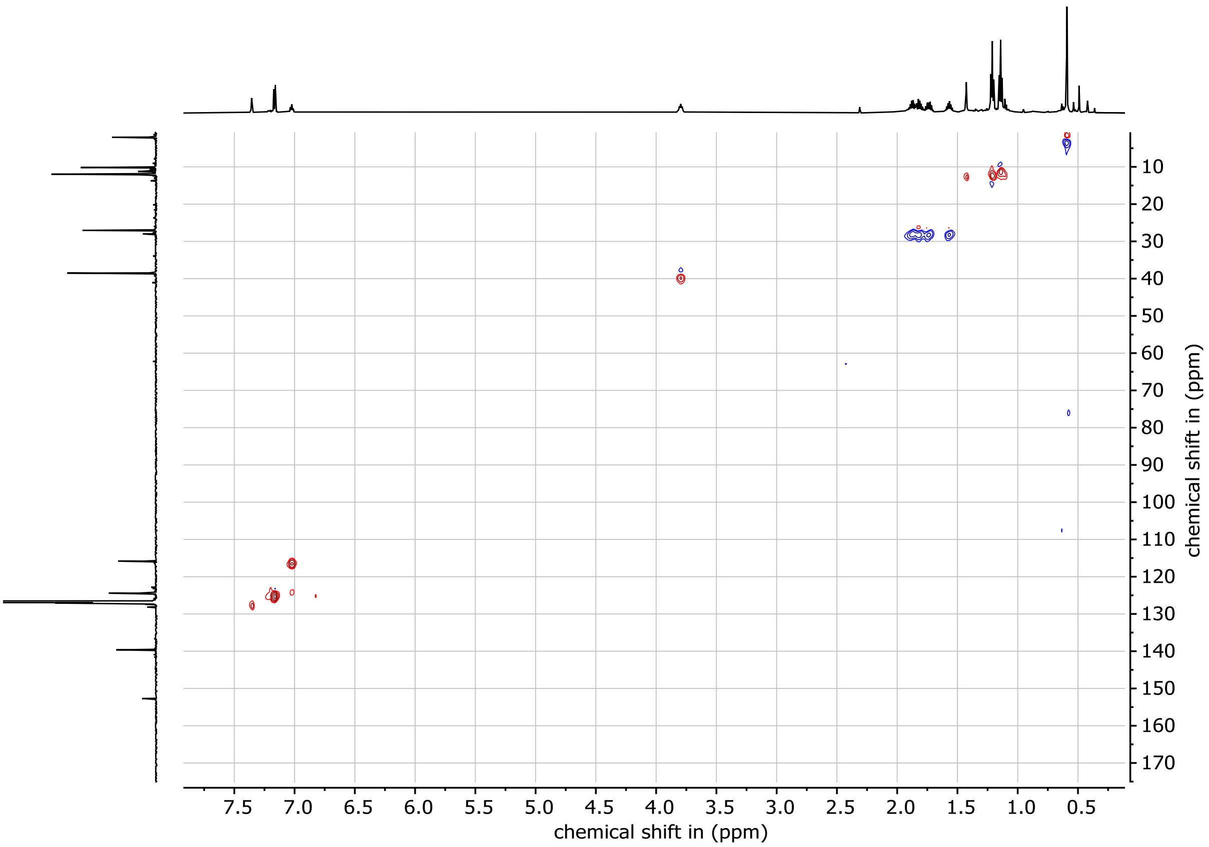

Figure S29: 2D-HSQC NMR (600/150 MHz, 298 K, C_6D_6) spectrum of (^{DIPeP}NN)Sr (**5**-Sr).

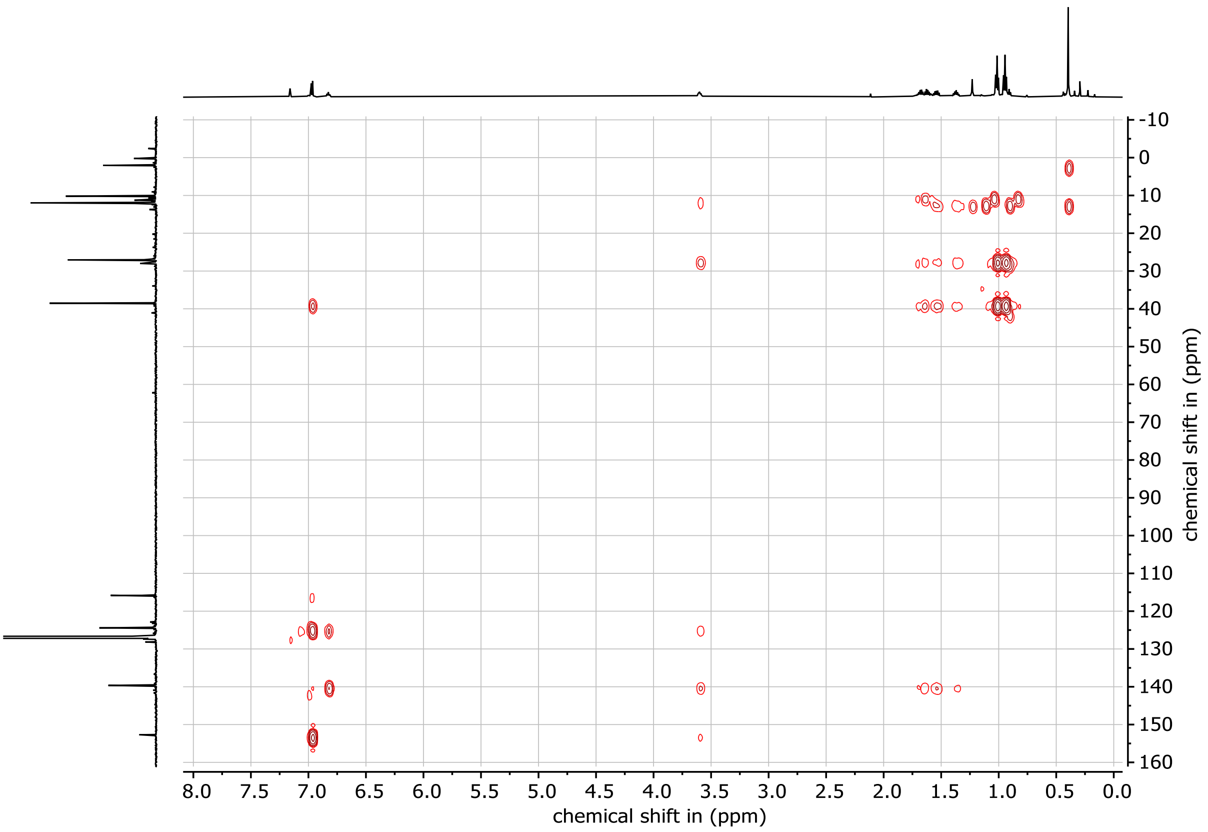

Figure S30: 2D-HMBC NMR (600/150 MHz, 298 K, C_6D_6) spectrum of (^{DIPeP}NN)Sr (**5**-Sr).

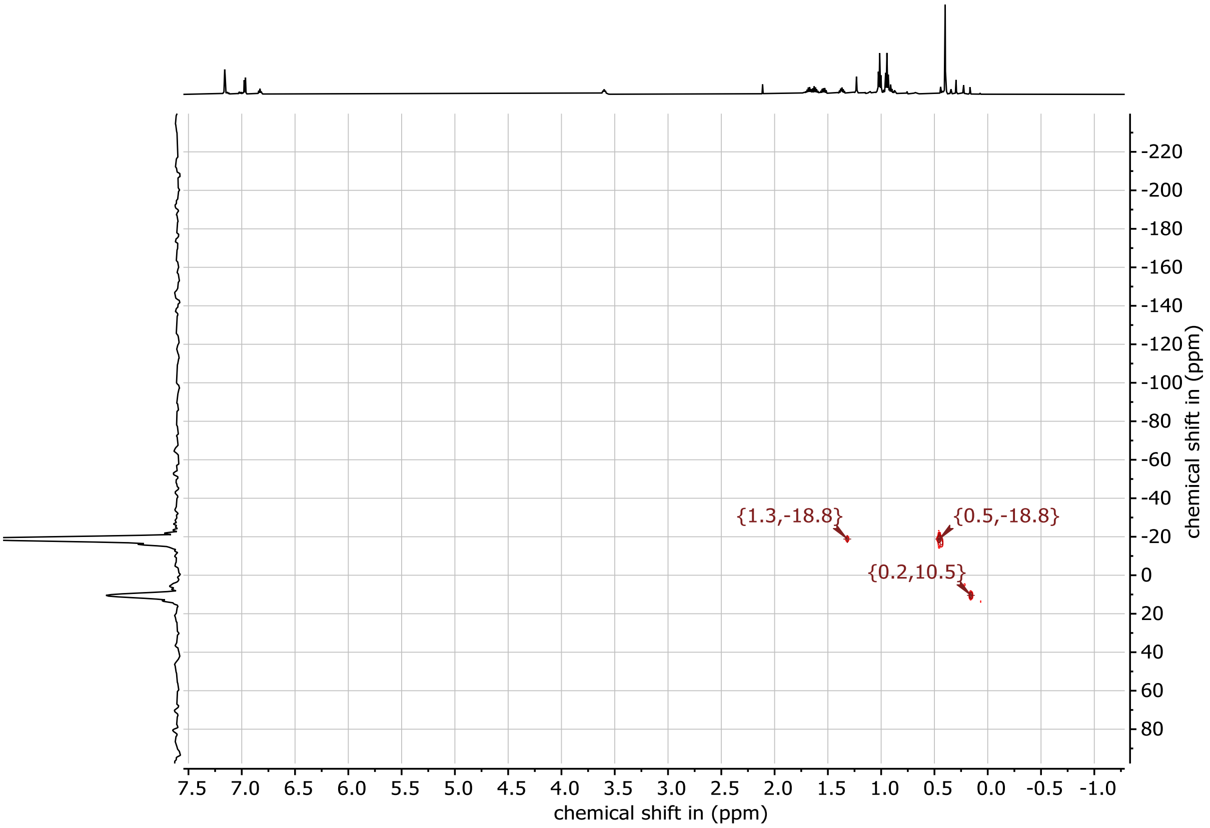

Figure S31: 2D-HMBC NMR (^{29}SI/H, 600/120 MHz, 298 K, C_6D_6) spectrum of (^{DIPeP}NN)Sr (**5**-Sr).

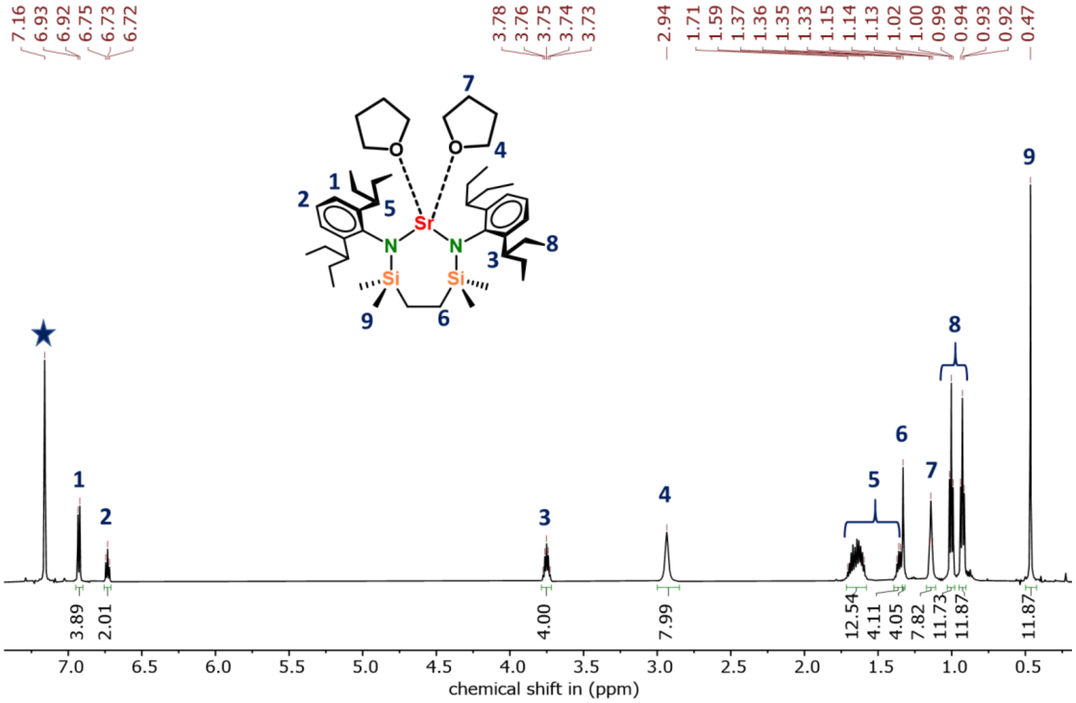

Figure S32: ^{1}H NMR (600 MHz, 298 K, C_6D_6) spectrum of (^{DIPeP}NN)Sr(THF)$_2$. Solvent residual signals are marked with a star.

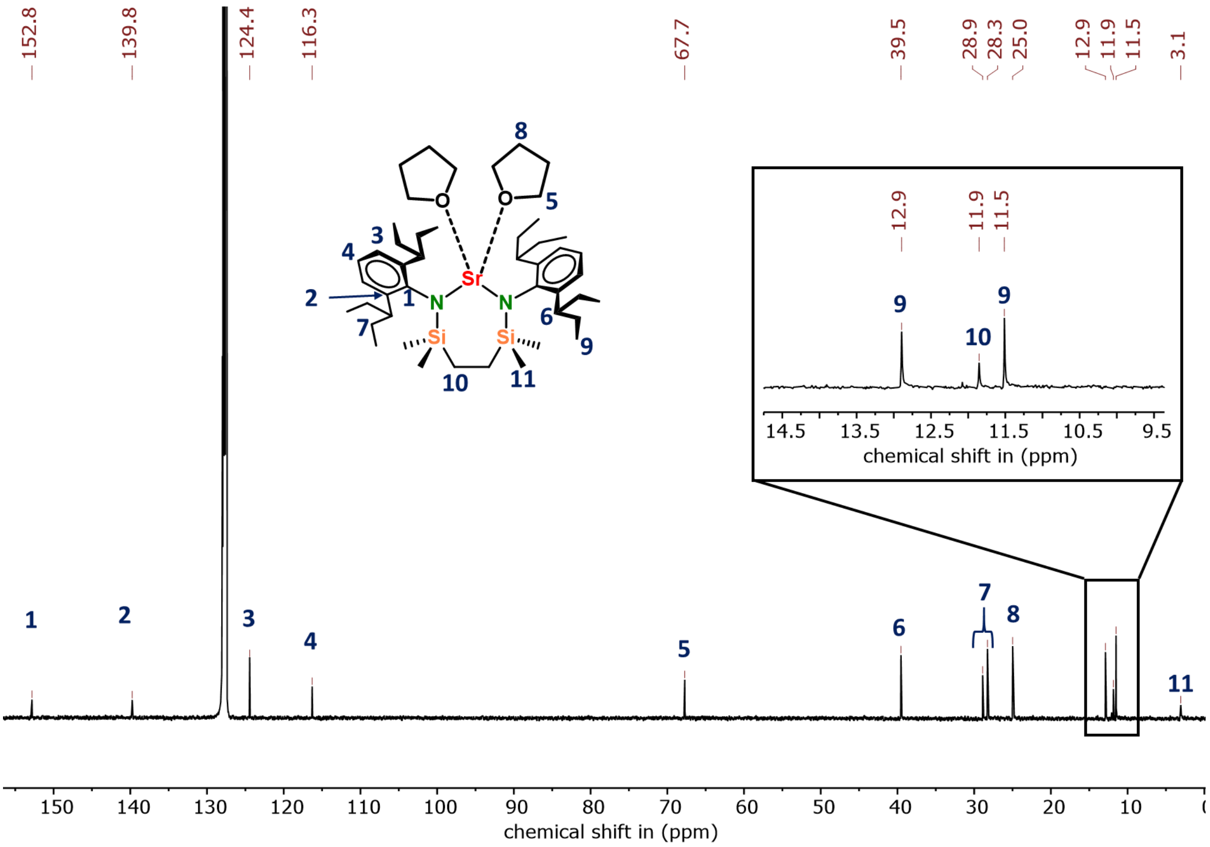

Figure S33: ^{13}C NMR (150 MHz, 298 K, C_6D_6) spectrum of (^{DIPeP}NN)Sr(THF)$_2$.

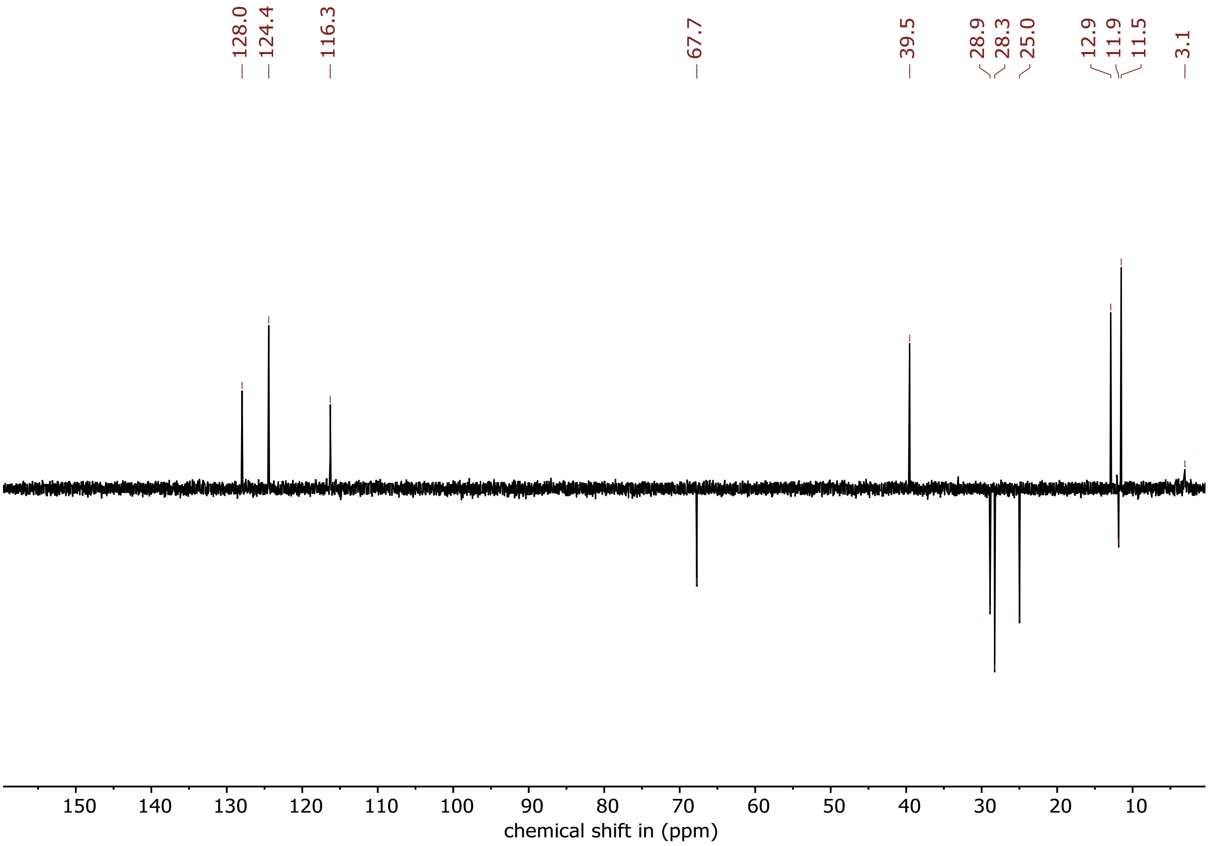

Figure S34: DEPT-135 NMR (150 MHz, 298 K, C_6D_6) spectrum of (^{DIPeP}NN)Sr(THF)$_2$.

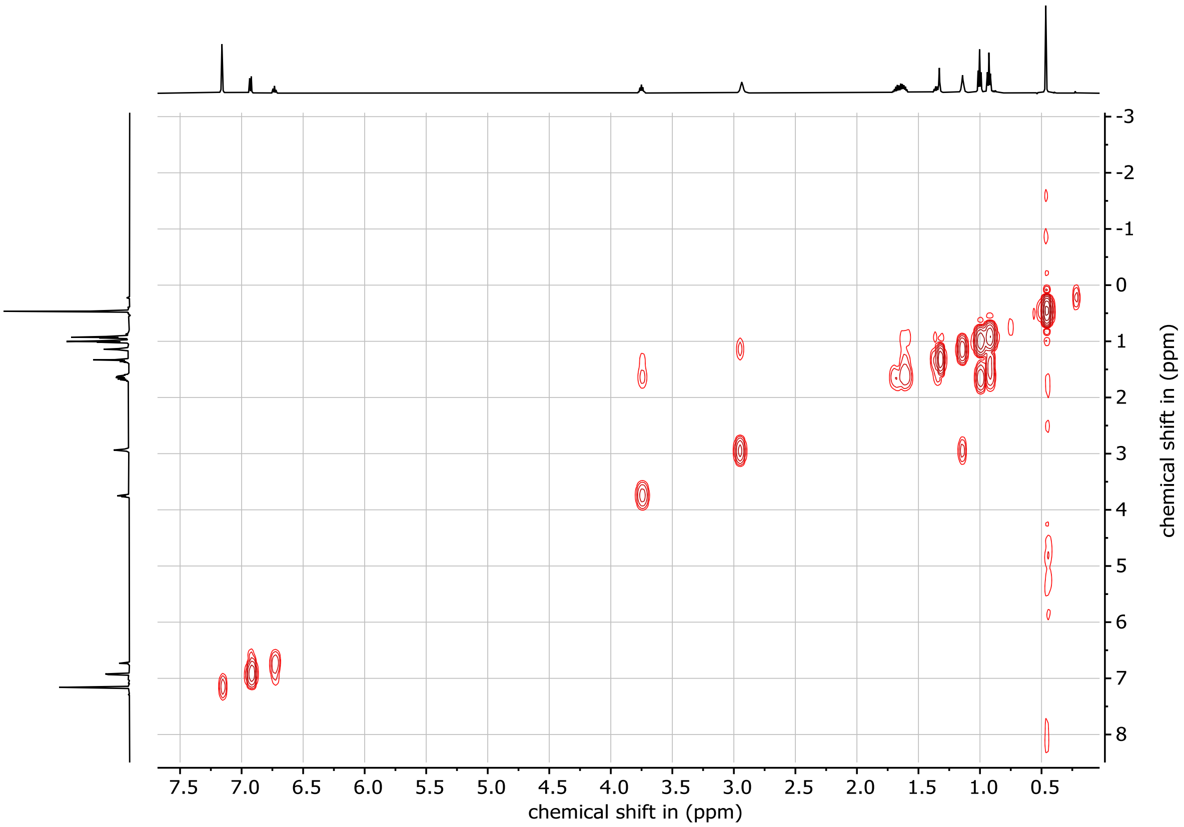

Figure S35: 2D-COSY NMR (600 MHz, 298 K, C_6D_6) spectrum of (^{DIPeP}NN)Sr(THF)$_2$.

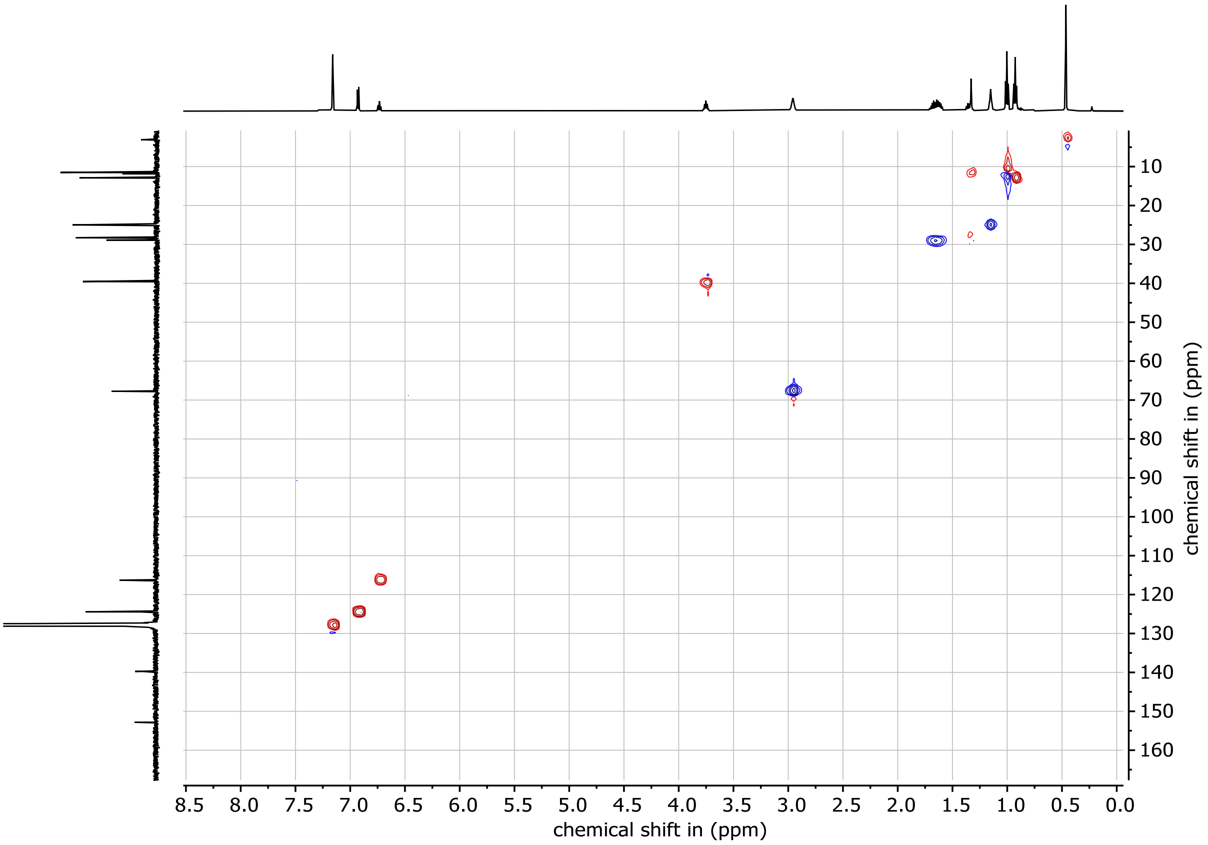

Figure S36: 2D-HSQC NMR (600/150 MHz, 298 K, C_6D_6) spectrum of (^{DIPeP}NN)Sr(THF)$_2$.

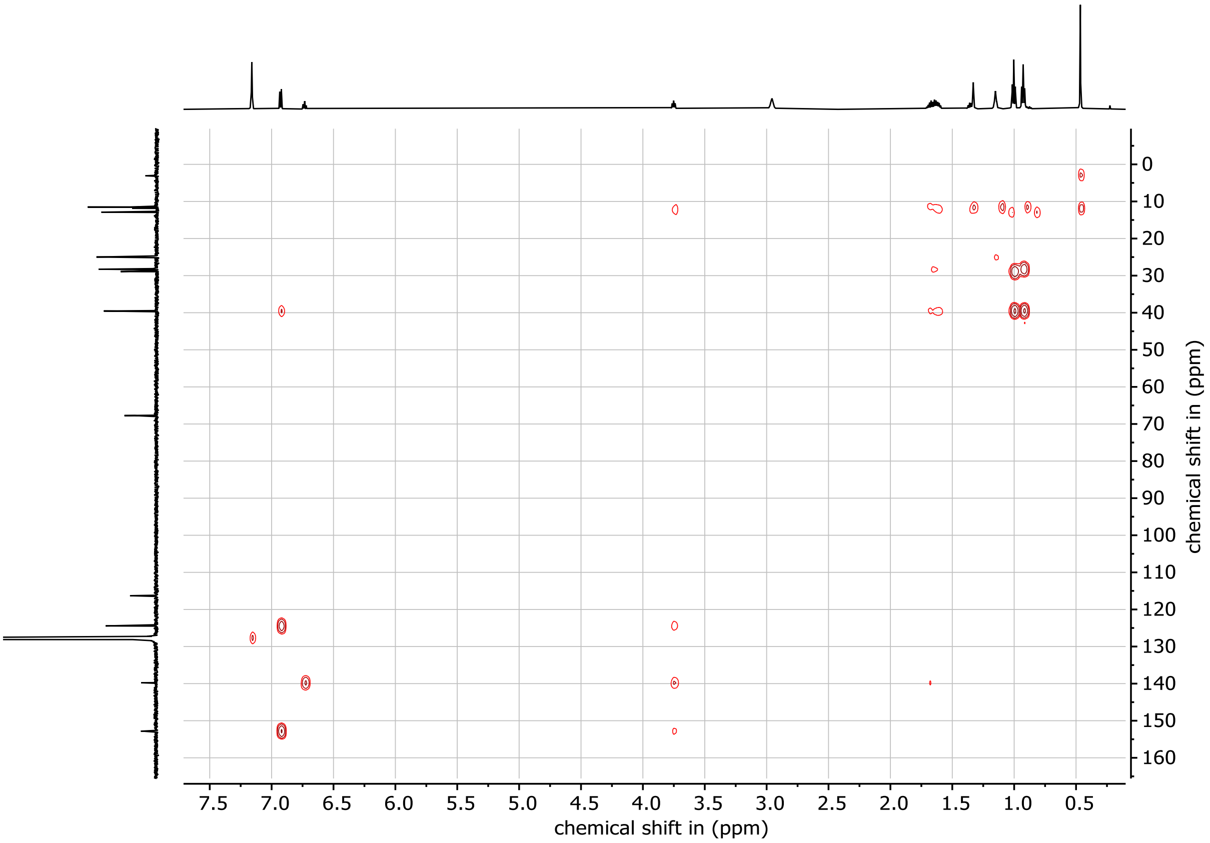

Figure S37: 2D-HMBC NMR (600/150 MHz, 298 K, C_6D_6) spectrum of (^{DIPeP}NN)Sr(THF)$_2$.

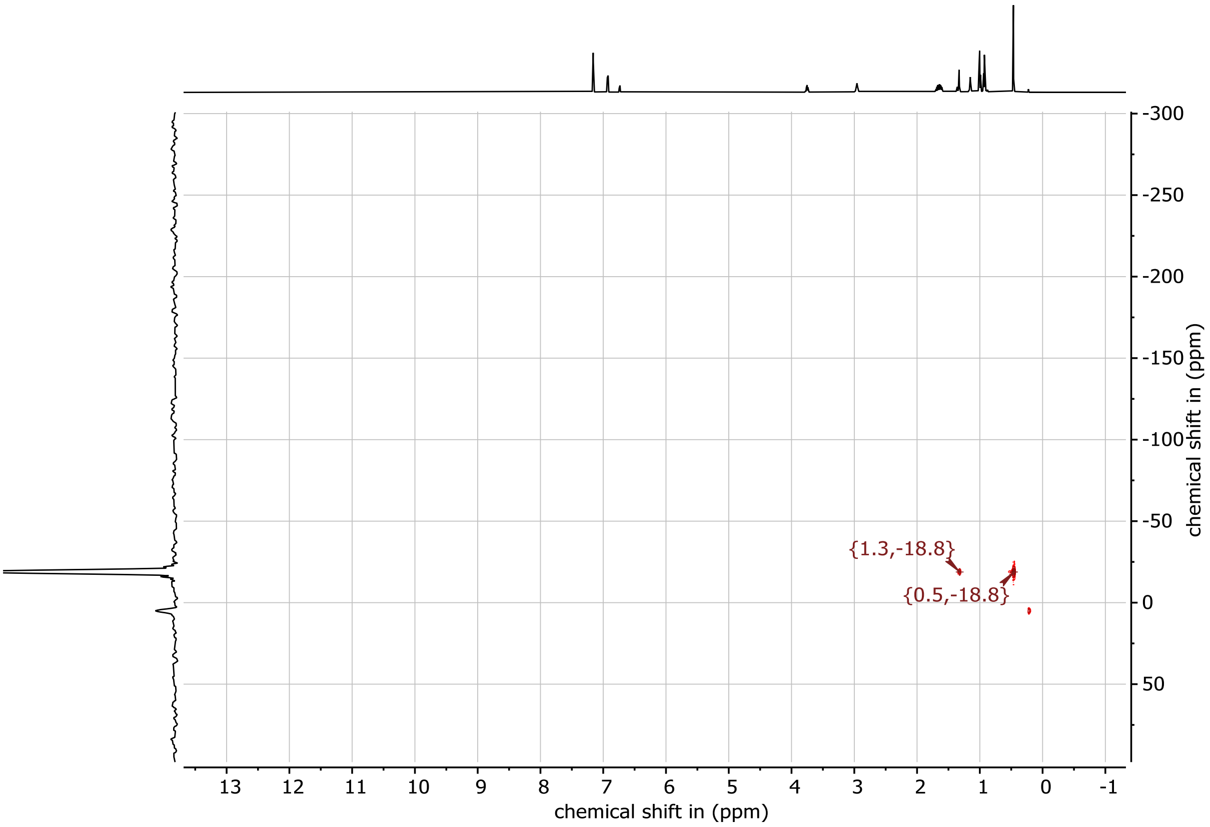

Figure S38: 2D-HMBC NMR (^{29}SI/H, 600/120 MHz, 298 K, C_6D_6) spectrum of (^{DIPeP}NN)Sr(THF)$_2$.

Synthesis of [K(^{DIPeP}NN)Ca]$_2$(N_2) (6-Ca)

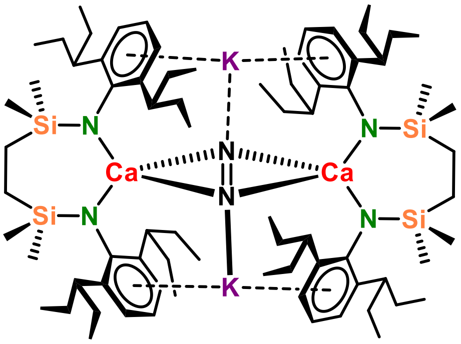

A Schlenk flask equipped with a glass stirring bar (Teflon reacts with **6**-Ca) was charged with (^{DIPeP}NN)Ca (200 mg, 0.331 mmol), K/KI (5% w/w, 730 mg, 0.993 mmol, 3 eq.) and MCH (9 mL). Stirring the suspension for 22 h led to color change from yellow to red. After filtration and removal of the solvent *in vacuo* the residue was stripped with pentane (2 x 5 mL). Drying under vacuum gave the product as an essentially pure orange powder (181 mg, 0.129 mmol, 83%). For obtaining crystals suitable for X-ray diffraction analysis, the product was dissolved in hexane (1.50 mL) and placed at -35 °C. After 3 d, orange microcrystalline solid precipitated, which was isolated by decantation of the supernatant and subsequently dissolved in MCH (400 µL) and hexane (200 µL). After one week, crystals suitable for X-ray diffraction analysis were obtained. Attempts to prepare ^{15}N-labeled compounds by storing a solution of the product under $^{15}N_2$ led to discoloration and decomposition of the complex.

^{1}H-NMR of 6-Ca: Due to broadened signals, a meaningful assignment of the peaks is not possible.

Elemental Analysis: Calculated for $C_{76}H_{132}N_6K_2Si_4Ca_2$ (M = 1400.63 g/mol): N 6.00, C 65.17, H 9.50 %. Found *(best results obtained)*: N 5.60, C 65.82, H 9.24 %.

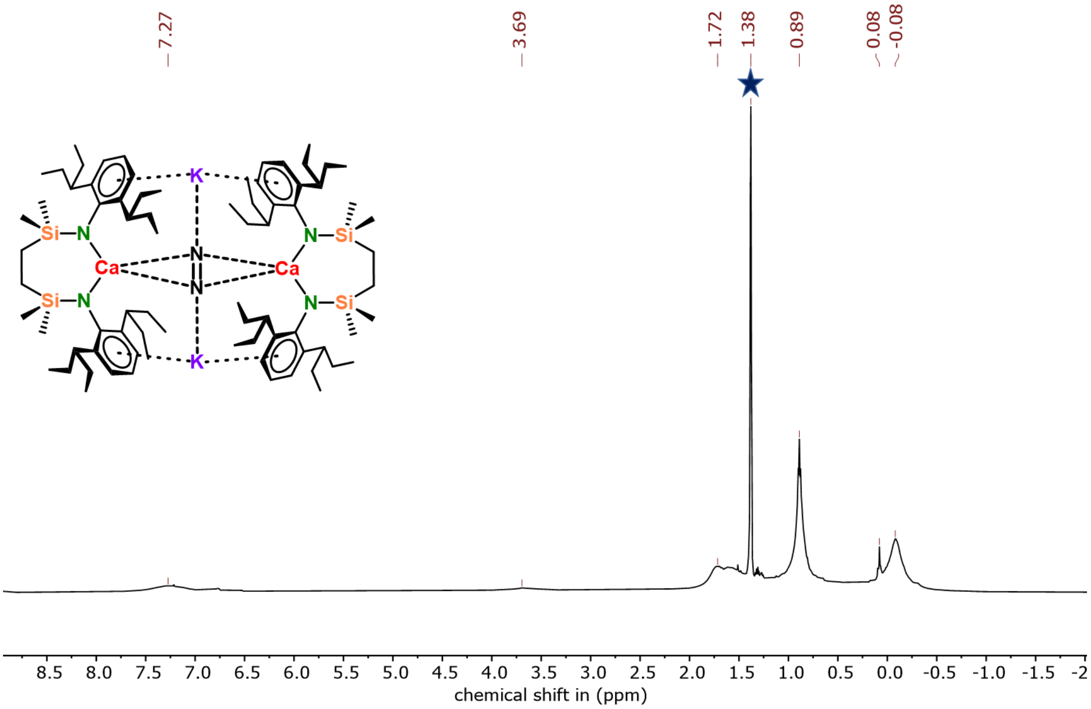

Figure S39: ^{1}H NMR (600 MHz, 298 K, C_6D_{12}) spectrum of **6**-Ca. Solvent residual signals are marked with a star.

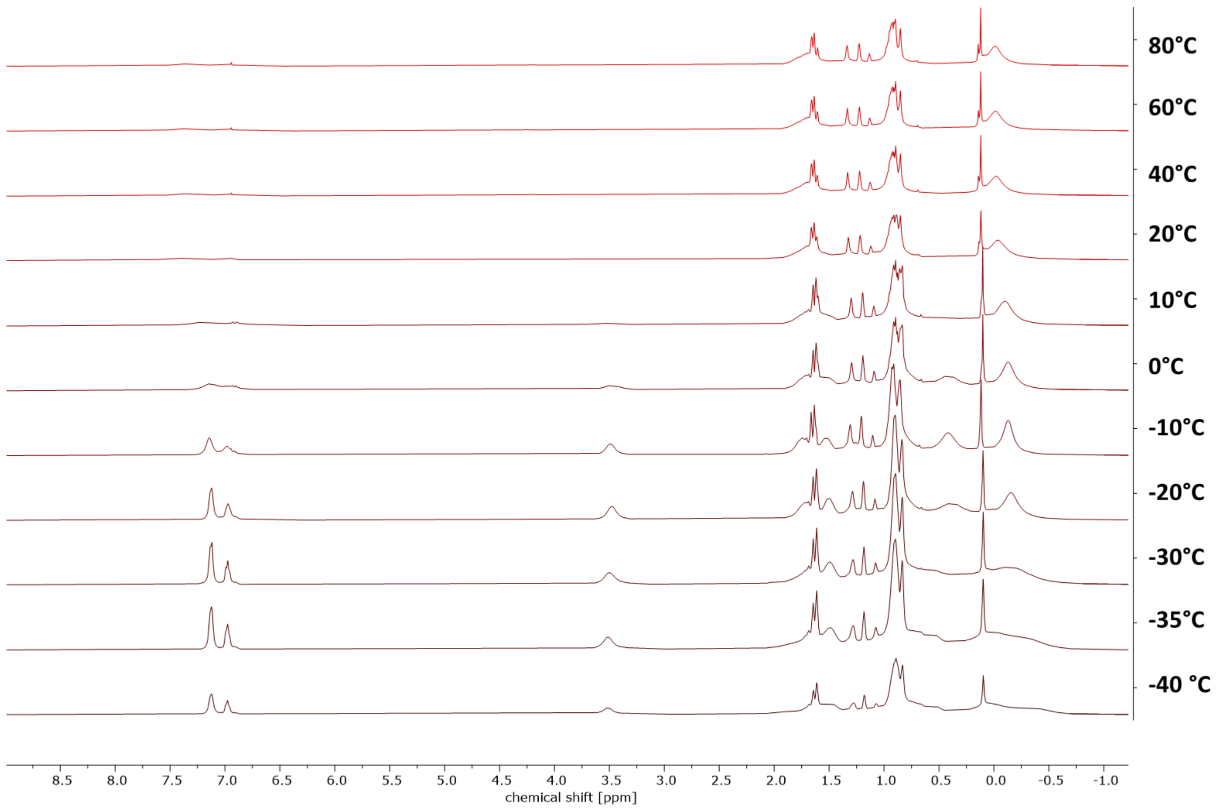

Figure S40: Temperature dependent ^{1}H NMR spectra (400.13 MHz, MCH-d_{14}) of **6**-Ca from -40 °C to 80 °C. The spectra show sharper resonance signals at temperatures below -10 °C than at higher temperatures. Decomposition at elevated temperatures up to 80 °C was not observed, which indicates significant thermal stability of the complex.

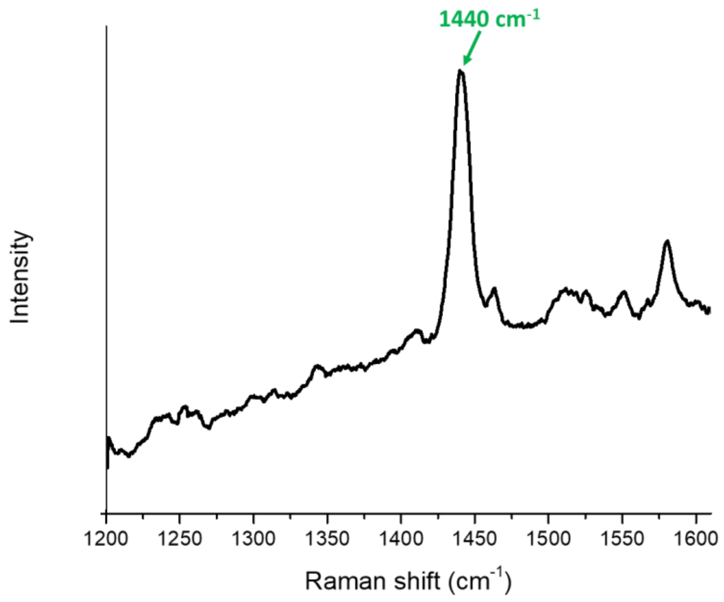

Figure S41: Raman spectrum (532 nm (0.74 mW), 1800 grat, 50 x 20s, 1200-1620 cm^{-1}) of **6**-Ca. The N-N Raman band can be observed at v = 1440 cm^{-1}. The measurement was performed at low intensity due to rapid decomposition at high laser power.

Synthesis of [K(^{DIPeP}NN)Sr]$_2$(N_2) (6-Sr)

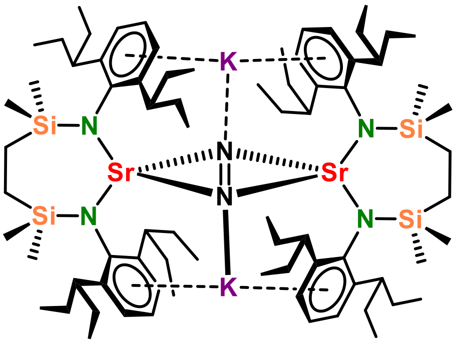

A Schlenk flask was equipped with a glass stirring bar (Teflon reacts with **6**-Sr) and charged with (^{DIPeP}NN)Sr (200 mg, 0.288 mmol), K/KI (5% w/w, 450 mg, 0.576 mmol, 2 eq.) and MCH (7 mL). The mixture was cooled to 10 °C and stirred for 3 d. Subsequently, the brown suspension was filtrated and the solvent removed *in vacuo*. After stripping with pentane (3 x 5 mL), the brown residue was washed with cold pentane (2 x 400 µL) and the remaining brown solid was briefly dried under vacuum. Crystals suitable for X-ray diffraction analysis were obtained from a solution of MCH (200 µL) and pentane (50 µL) at -35 °C. The crystals were isolated by decantation of the supernatant, washed with cold pentane (70 µL) and briefly dried *in vacuo* to obtain the product as yellow crystals (Yield: 24.0 mg, 0.016 mmol, 9%). Attempts to prepare ^{15}N-labeled compounds by storing a solution of the product under $^{15}N_2$ led to discoloration and decomposition of the complex.

^{1}H-NMR (600.13 MHz, C_7D_{14}, 298 K): δ = 7.53 (d, $^3J_{H,H}$ = 7.4 Hz, 8H, *m*-C**H**$_{arom}$), 7.34-7.31 (m, 4H, *p*-C**H**$_{arom}$), 3.88 (br, 8H, C**H** *i*Pen), 2.21-1.85 (m, 32H, C**H**$_2$ *i*Pen, *superimposed by solvent signals*), 1.25 (t, $^3J_{H,H}$ = 7.3 Hz, 32H, C**H**$_3$ *i*Pen), 0.91 (br, 16H, C**H**$_3$ *i*Pen), 0.52 (s, 8H, C**H**$_2$ backbone), 0.29 (s, 24H, C**H**$_3$ backbone) ppm.

^{13}C{^{1}H}-NMR (151.62 MHz, C_7D_{14}, 298 K): δ = 159.7 (**C**-N), 145.6 (*o*-**C**$_{arom}$), 125.3 (*m*-**C**H$_{arom}$), 116.2 (*p*-**C**H$_{arom}$), 40.0 (**C**H *i*Pen), 28.9 (**C**H$_2$ *i*Pen), 13.1 (**C**H$_3$ *i*Pen), 12.0 (**C**H$_3$ *i*Pen), 9.0 (**C**H$_2$ backbone), 1.1 (Si-**C**H$_3$) ppm.

^{29}SI/^{1}H-HSQC (119.22 MHz, C_6D_6, 298 K): -16.5 ppm.

Elemental Analysis: Calculated for $C_{76}H_{132}N_6K_2Si_4Sr_2$ + 0.8 eq. co-crystalized MCH (C_7H_{14}) (M = 1495.71): N 5.34, C 62.26, H 9.17 %. Found: N 4.95, C 61.88, H 9.18 %.

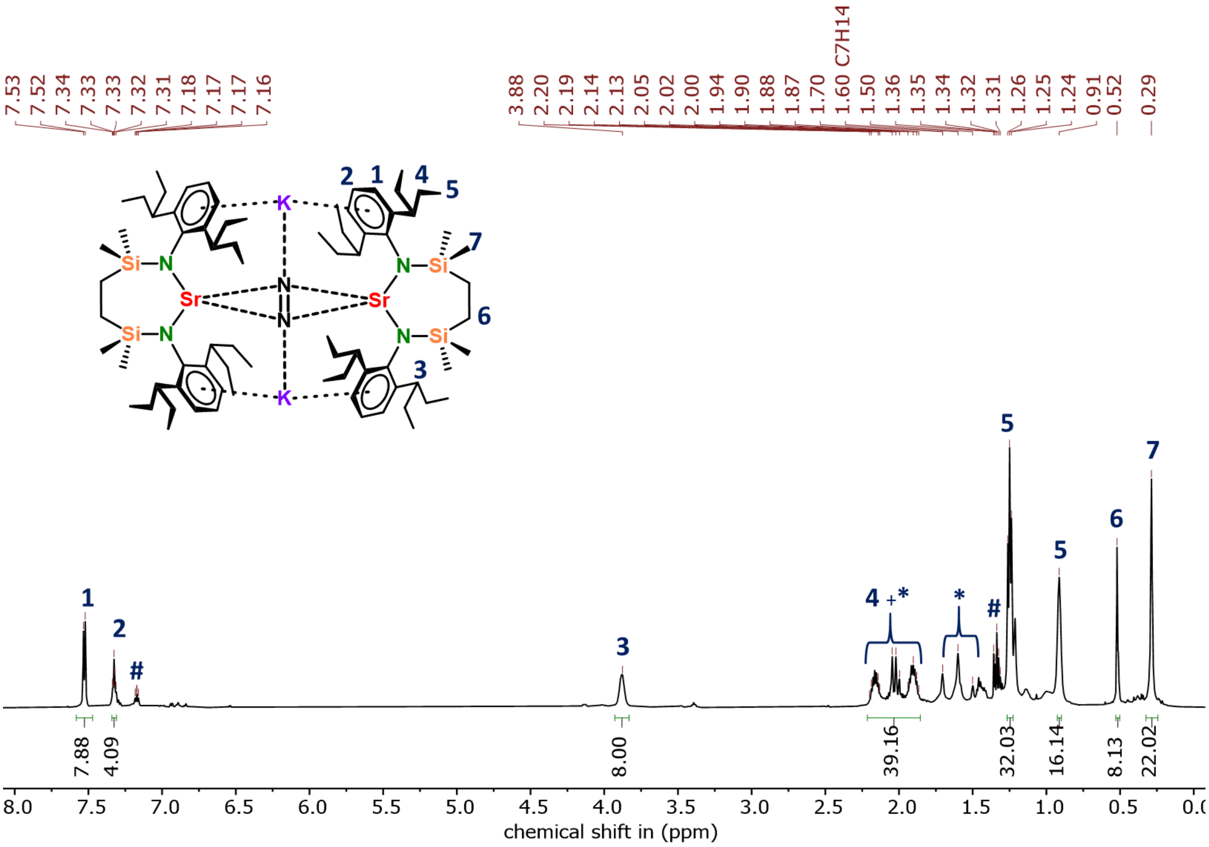

Figure S42: ^1H NMR (600 MHz, 298 K, MCH-d_{14}) spectrum of **6**-Sr. Solvent is marked with an asterisk, unknown impurities with a hashtag.

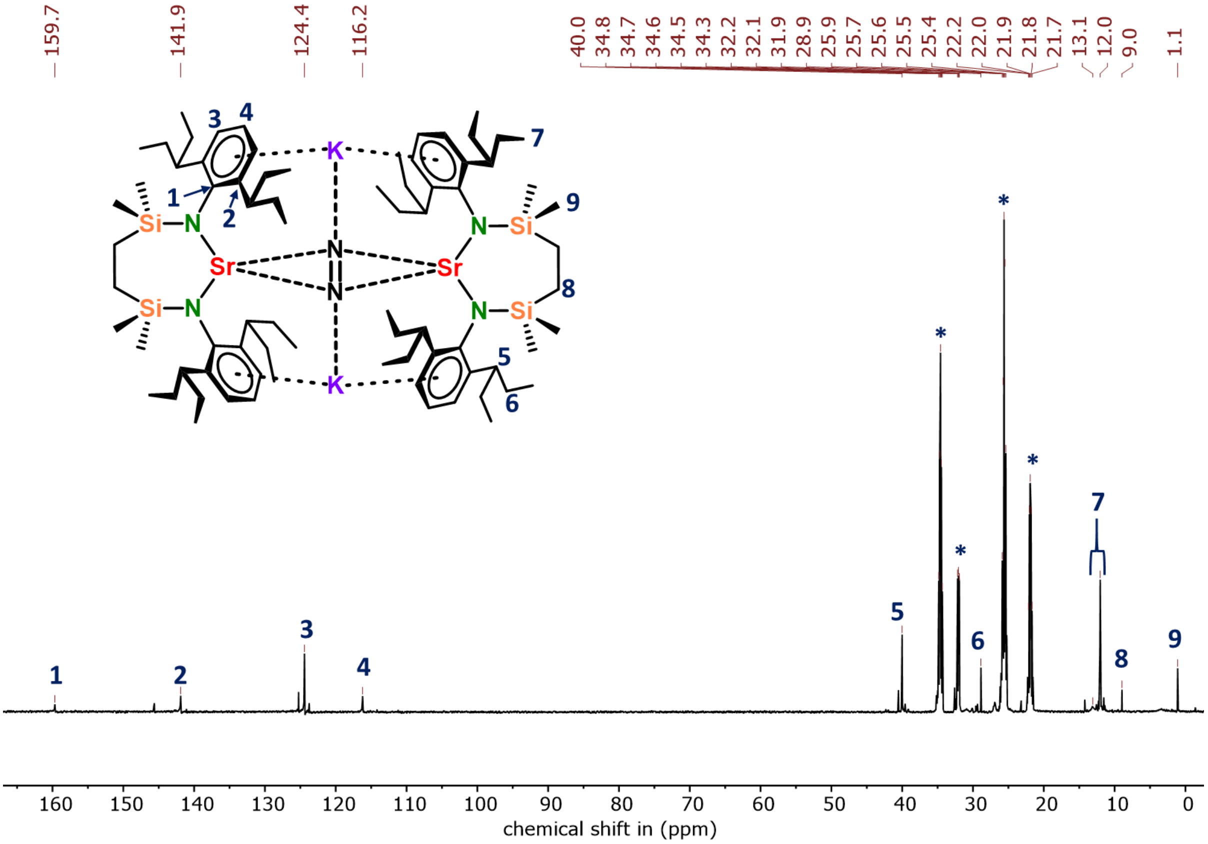

Figure S43: ^{13}C NMR (150 MHz, 298 K, MCH-d_{14}) spectrum of **6**-Sr. Solvent is marked with an asterisk.

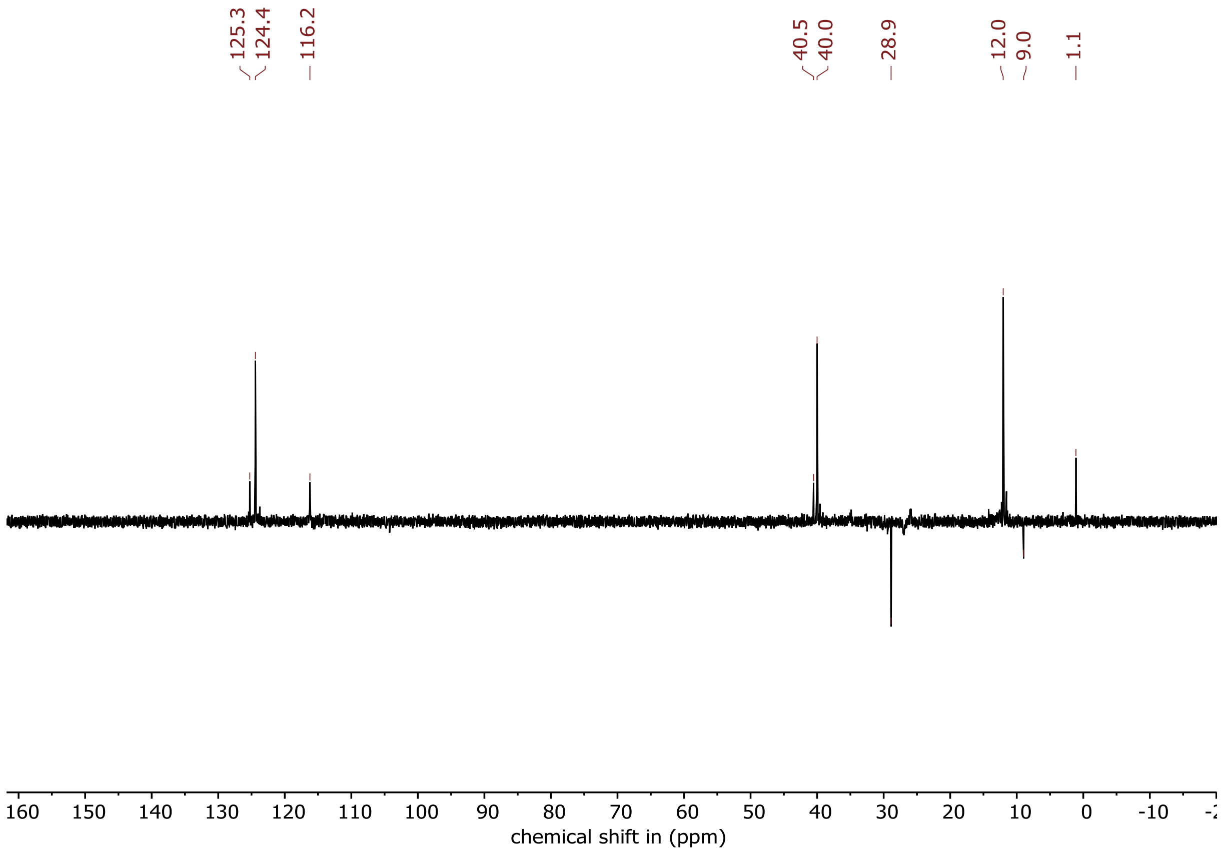

Figure S44: DEPT-135 NMR (150 MHz, 298 K, MCH-*d*$_{14}$) spectrum of **6**-Sr.

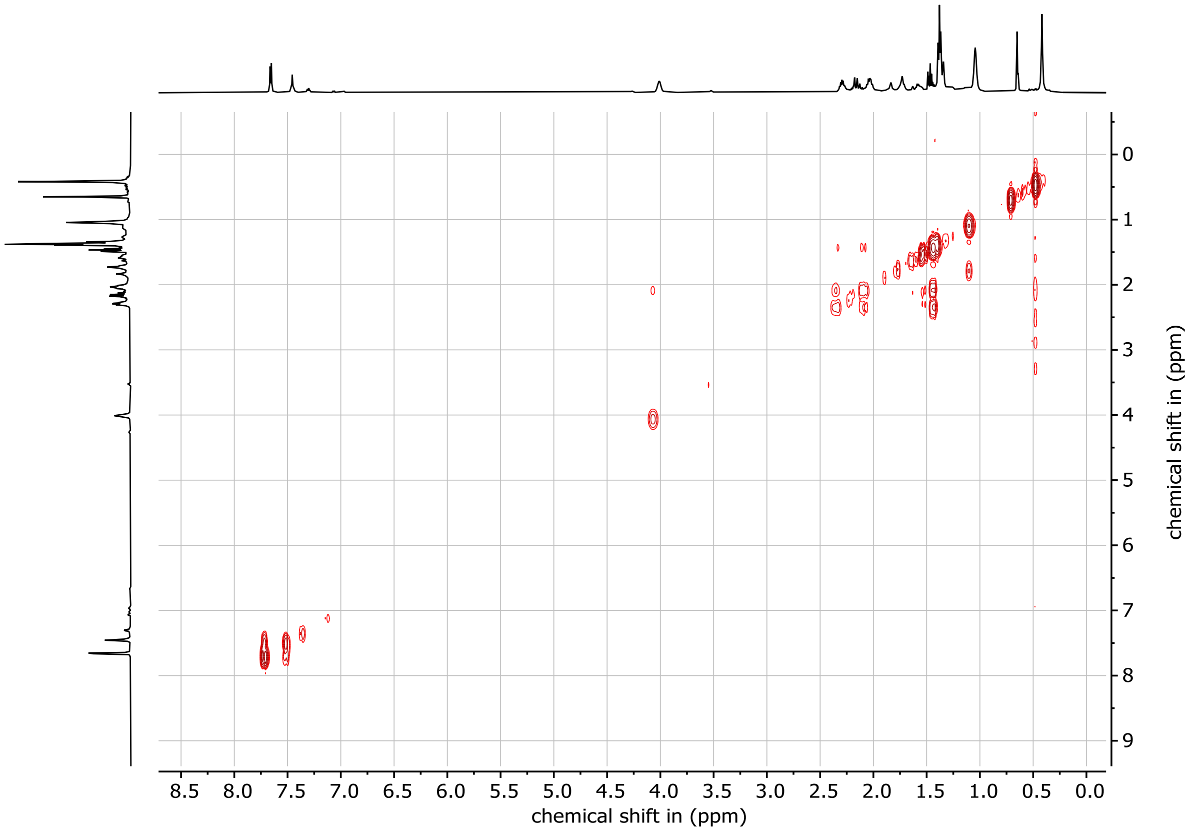

Figure S45: 2D-COSY NMR (600 MHz, 298 K, MCH-*d*$_{14}$) spectrum of **6**-Sr.

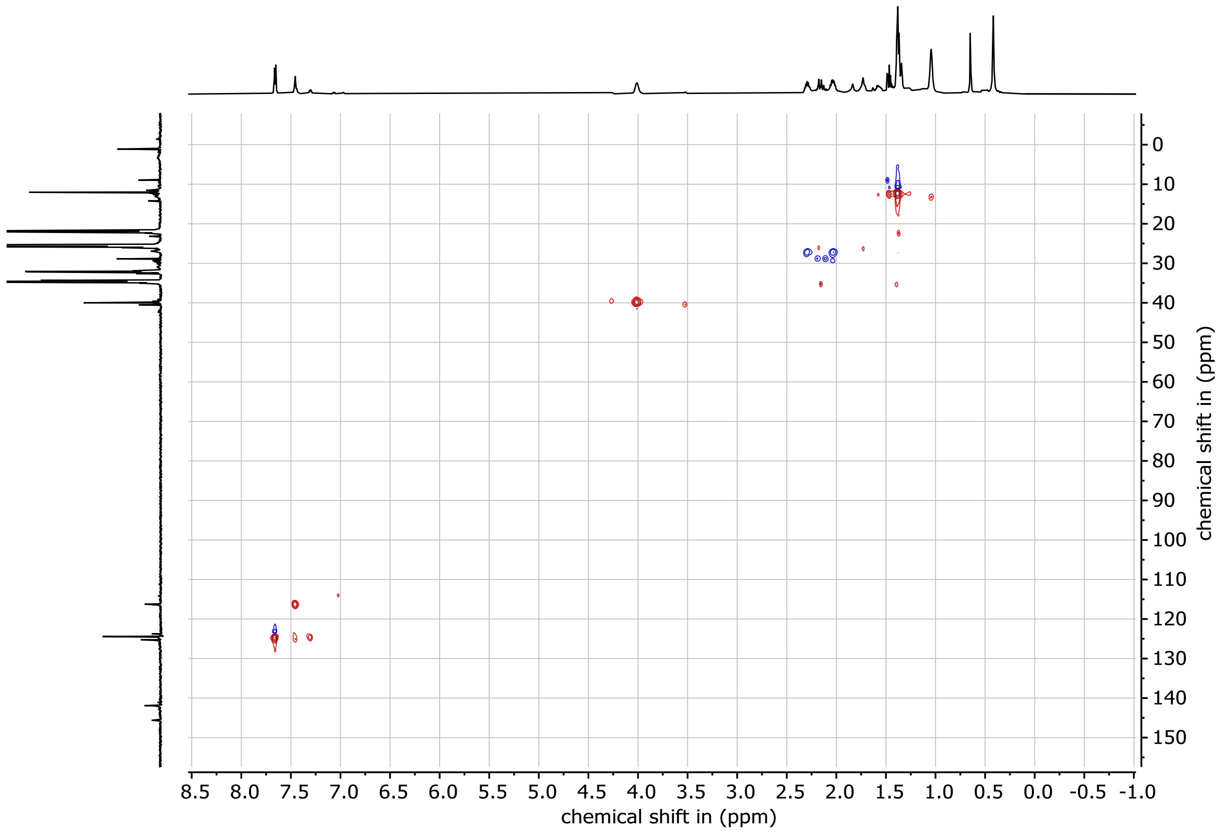

Figure S46: 2D-HSQC NMR (600/150 MHz, 298 K, MCH-d_{14}) spectrum of **6**-Sr.

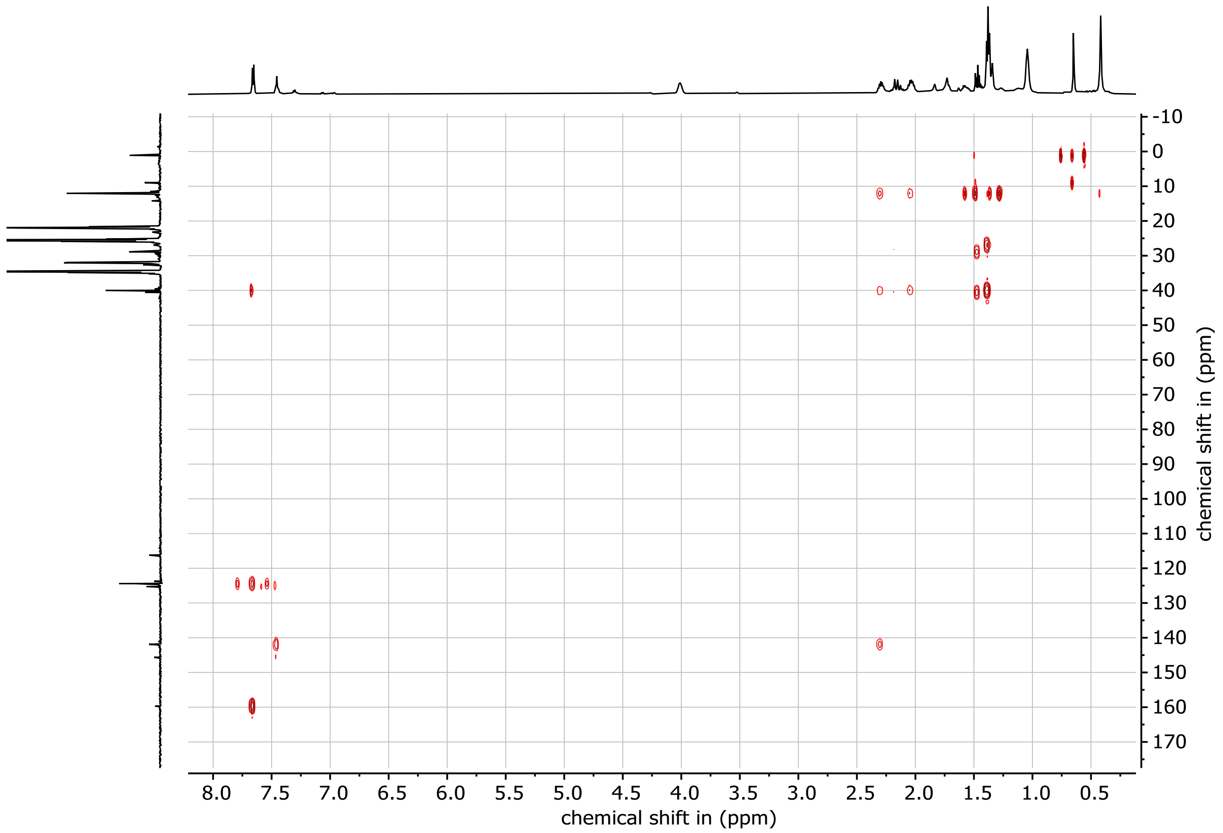

Figure S47: 2D-HSQC NMR (600/150 MHz, 298 K, MCH-d_{14}) spectrum of **6**-Sr.

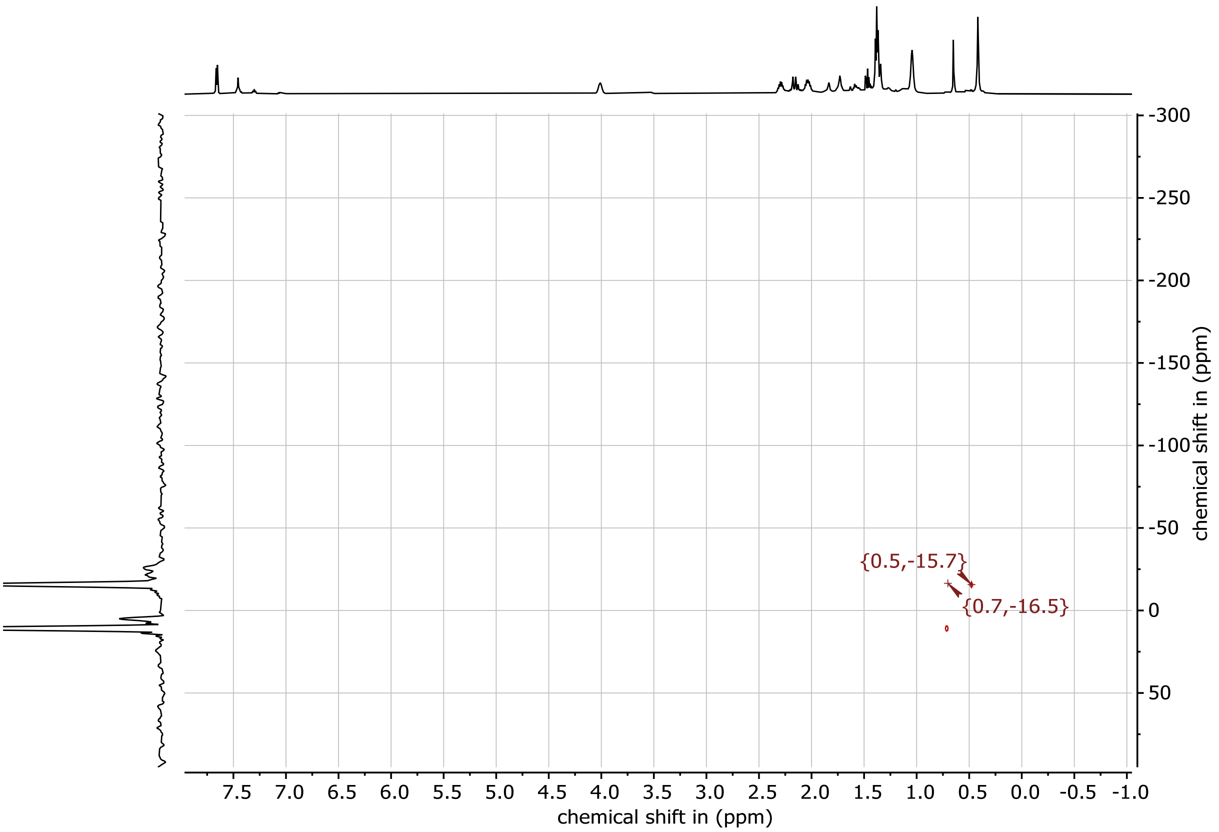

Figure S48: 2D-HMBC NMR (^{29}SI/H, 600/120 MHz, 298 K, MCH-*d14*) spectrum of **6**-Sr.

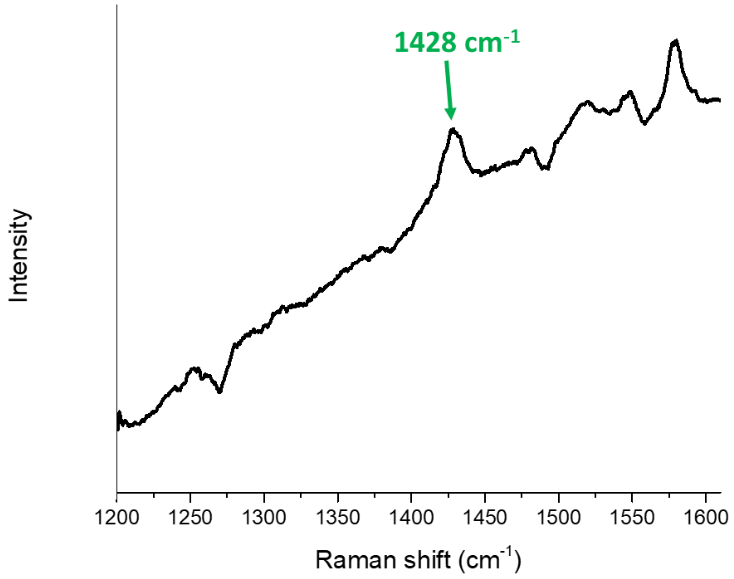

Figure S49: Raman spectrum (532 nm (0.74 mW), 1800 grat, 60 x 5s, 1200-1620 cm^{-1}) of **6**-Sr. The N-N Raman stretching band can be observed at ν = 1428 cm^{-1}. The compound decomposed significantly faster under prolonged laser irradiation than **6**-Ca.

Formation of (^{DIPeP}NN)SrKF (7) during reduction of (^{DIPeP}NN)Sr (5-Sr) with K/KI

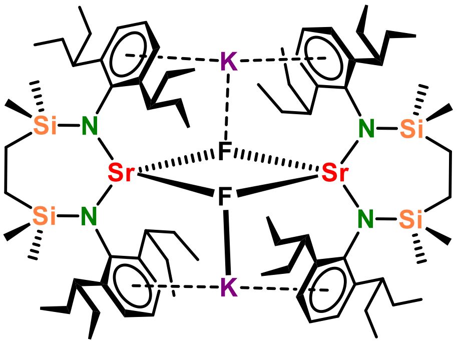

A Schlenk flask was equipped with a common Teflon-coated stirring bar and charged with (^{DIPeP}NN)Sr (200 mg, 0.288 mmol), K/KI (5% w/w, 225 mg, 0.288 mmol, 1 eq.) and MCH (7 mL) and the reaction was stirred for 3 d at room-temperature. Subsequently, the suspension was filtrated and the solvent removed *in vacuo*. After stripping with pentane (3 x 5 mL) a yellow powder was obtained. Small yellow crystals of **7** suitable for X-ray diffraction analysis were obtained from a solution of pentane (300 µL) at -35 °C but the product quantity was insufficient for further analysis.

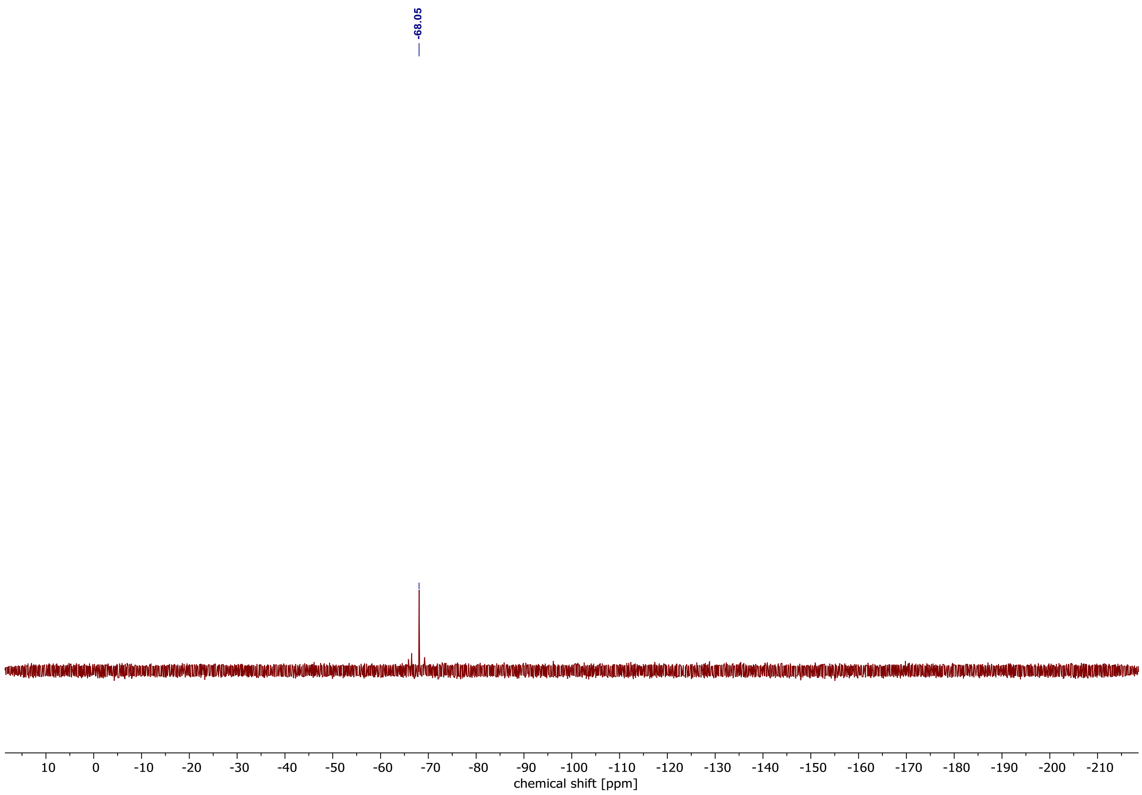

Figure S50: ^{19}F NMR (376.46 MHz, 298 K, C_6D_{12}) of the crude reaction product. The resonance signal appearing at -68.1 ppm can be attributed to complex **7**. This signal disappears over time.

3. Reactivity studies

Reduction of (BDI*)SrI in solution

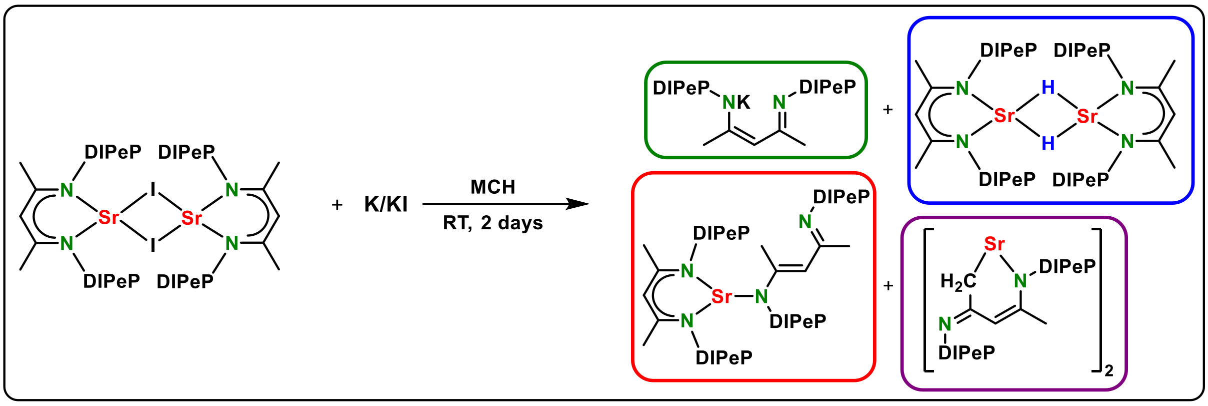

[(BDI*)SrI]$_2$ (200 mg, 0.134 mmol) and K/KI (5% w/w, 733mg, 0.938 7 eq. K) were suspended in MCH (5 mL) and stirred for 2 d at room-temperature. Afterwards the suspension was filtrated and all volatiles were removed *in vacuo*. Stripping with pentane (2 x 3 mL) and briefly drying under vacuum gave an off-white powder. ^{1}H NMR spectra (**Figure S51**) revealed the formation of several products. Dissolving the powder in a mixture of pentane/THF and storing the solution at -35 °C gave colorless crystals suitable for X-ray diffraction analysis, which revealed a mixture of the THF adduct of **2**·(THF)$_2$ and the homoleptic complex **3**·(THF)$_2$.

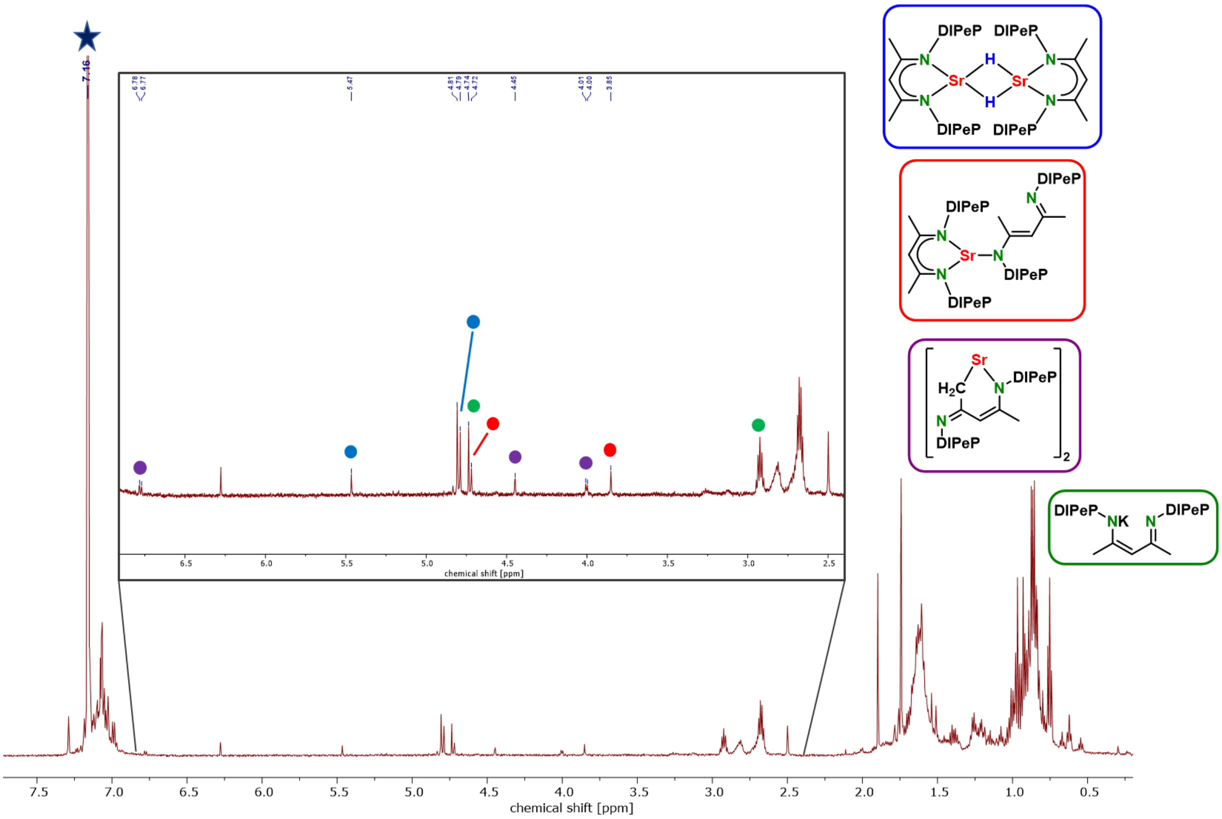

Figure S51: ^{1}H NMR (600.13 MHz, 298 K, C_6D_6) spectrum of the reaction depicted above. Similar to the Ca analogues, the formation of [(BDI*)SrH]$_2$ (**1**) and [(BDI*-H)Sr]$_2$ (**2**) is indicative of the decomposition of [(BDI*)Sr]$_2$(μ-N$_2$).[S4] Solvent residual signals are marked with a star.

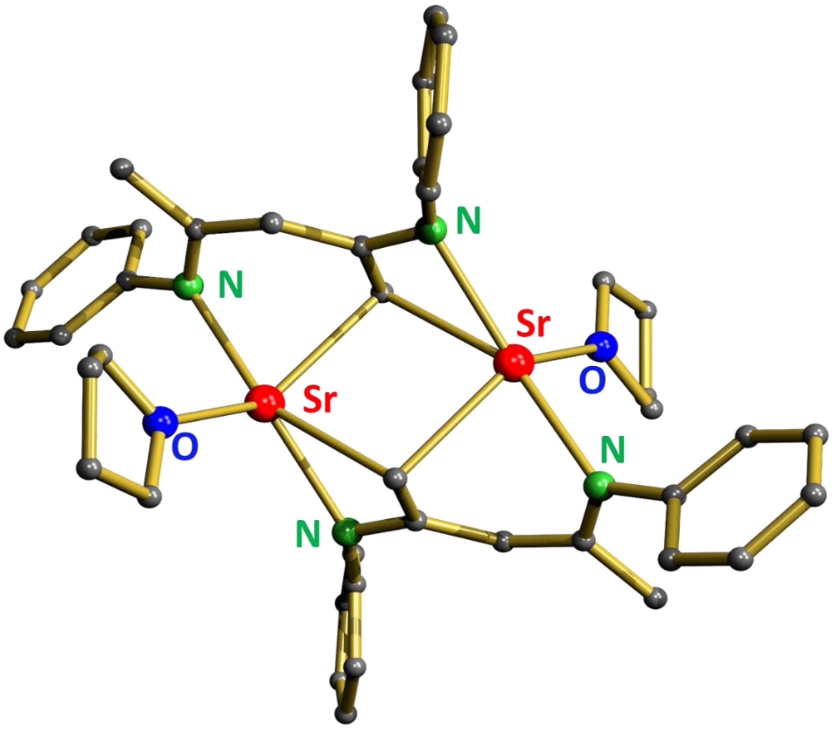

Figure S52: Crystal structure of **2**·(THF)$_2$. Hydrogen atoms and *i*-pentyl groups have been omitted for clarity.

Reduction of (BDI*)SrI *via* ball-milling

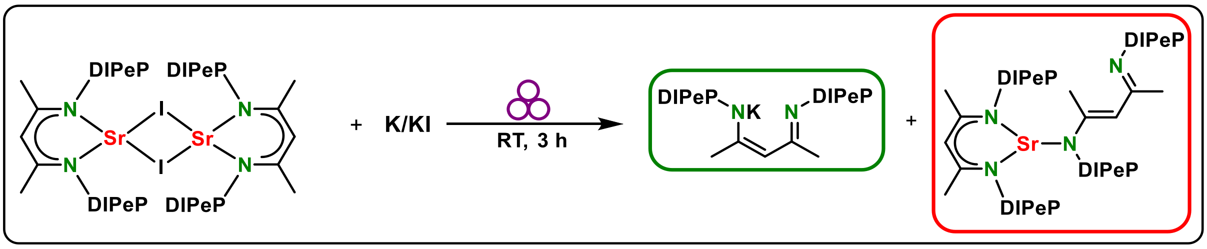

[(BDI*)SrI]$_2$ (200 mg, 0.134 mmol) was reacted with K/KI (5% w/w, 314 mg, 0.402 mmol, 3 eq. K) in a ball-mill vessel equipped with three steel balls and ground with a ball-mill speed of 3600 rpm for 3 h. The purple powder was extracted with hexane (4 mL) and the yellow solution was dried *in vacuo* to obtain a brown solid. ^{1}H NMR of the crude product showed residual [(BDI*)SrI]$_2$ as well as (BDI*)K and (BDI*)$_2$Sr (**3**). Crystallization from a mixture of pentane/THF gave colorless crystals of **3**·(THF)$_2$.

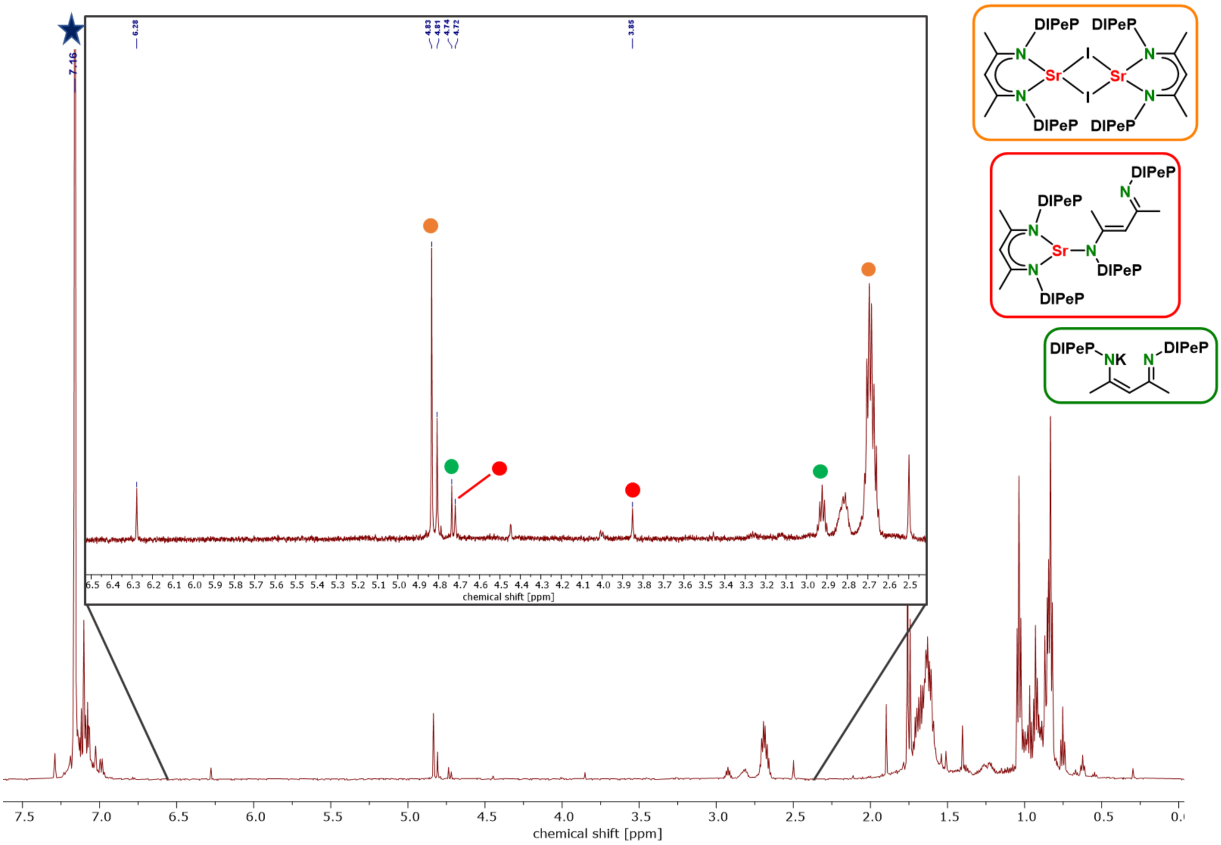

Figure S53: ^{1}H NMR (600.13 MHz, 298 K, C_6D_6) spectrum of the reaction in the ball-mill. The marked signals can be attributed to (BDI*)K and the homoleptic (BDI*)$_2$Sr species. Solvent residual signals are marked with a star.

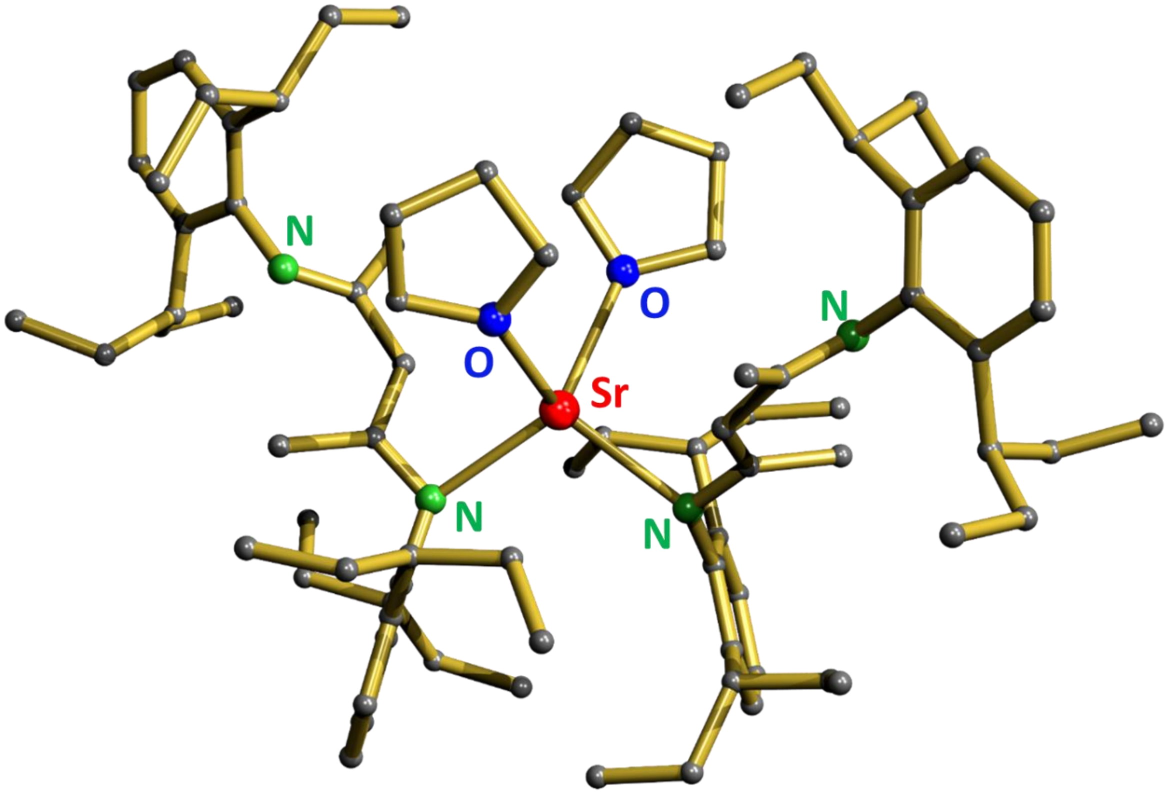

Figure S54: Crystal structure of **3**·(THF)$_2$. Hydrogen atoms have been omitted for clarity.

Reactions of 6-Ae with PTFE

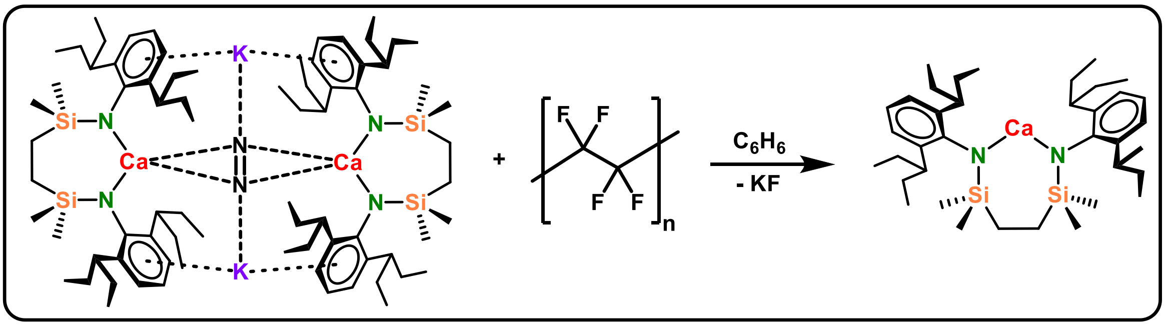

In a J-Young tube, a solution of **6**-Ca (30.0 mg, 21.4 µmol) in benzene was added to PTFE powder (23.0 mg, 200 µmol, 1 µm). Vigorous gas release and darkening of the PTFE powder was observed immediately. The reaction was stirred and the reaction progress was monitored *via* ^{1}H NMR spectroscopy. After 5 min the black suspension was filtrated. The ^{1}H NMR spectrum (**Figure S55**) of the dark yellow solution showed the selective formation of (^{DIPeP}NN)Ca as the main product. In the ^{19}F NMR spectrum (**Figure S56**) the presence of several unknown species can be seen. In addition, the black solid residue was washed with pentane (1 mL), briefly dried *in vacuo* and subsequently extracted with H_2O (800 µL). The ^{19}F NMR spectrum (**Figure S57**) of the filtrated aqueous solution shows one NMR signal, which can be attributed to KF.

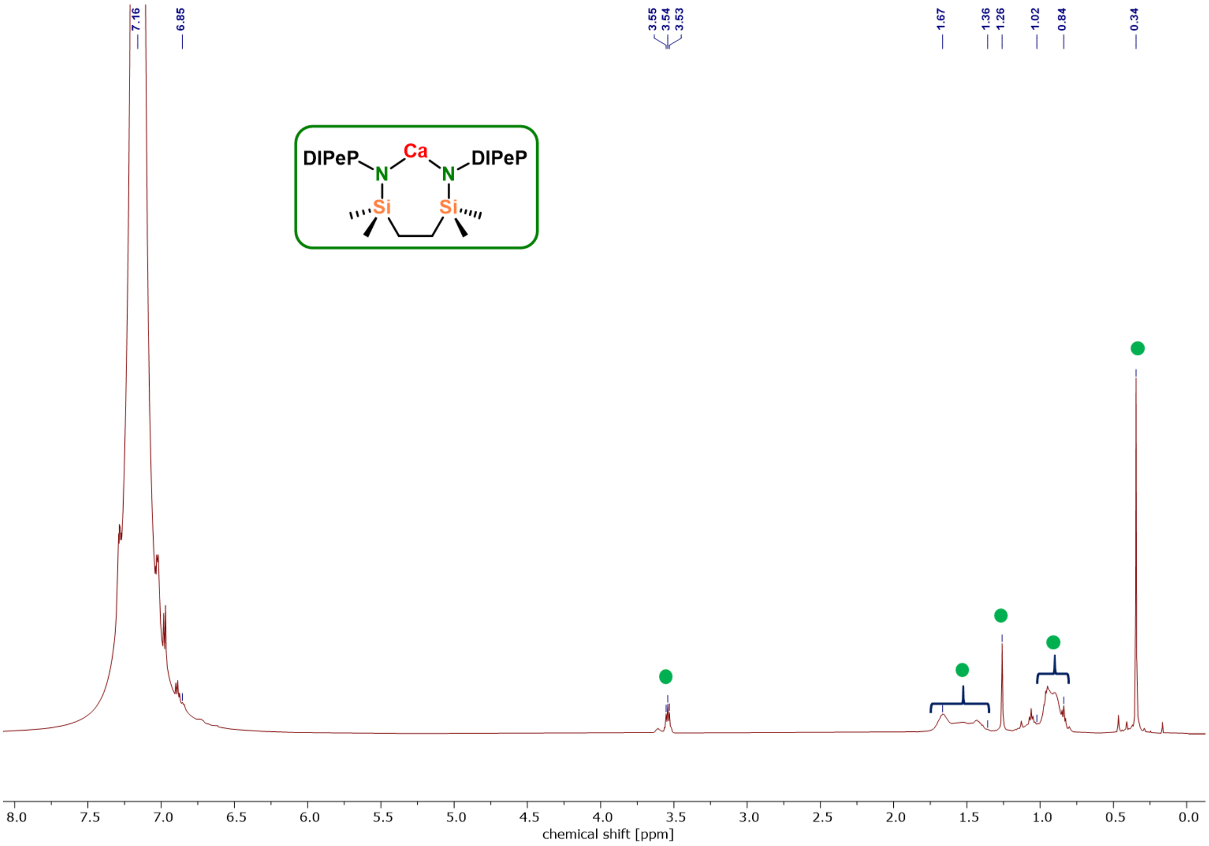

Figure S55: ^{1}H NMR (600.13 MHz, 298 K, C_6H_6) spectrum of the reaction between **6**-Ca and PTFE powder. The marked signals can be assigned to the (^{DIPeP}NN)Ca precursor complex.

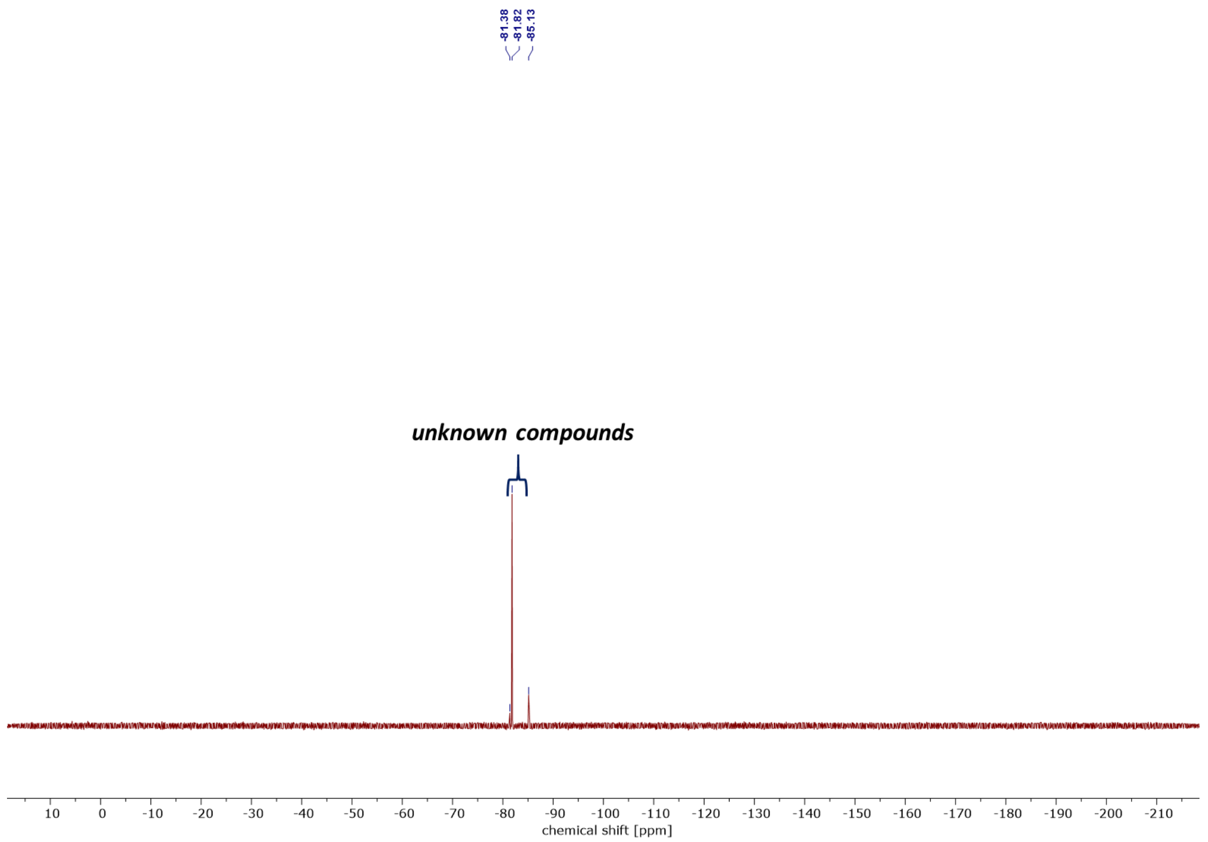

Figure S56: ^{19}F NMR (376.46 MHz, 298 K, C_6H_6) spectrum of the reaction between **6**-Ca and PTFE powder. The spectrum was measured after filtering off the black solid residue.

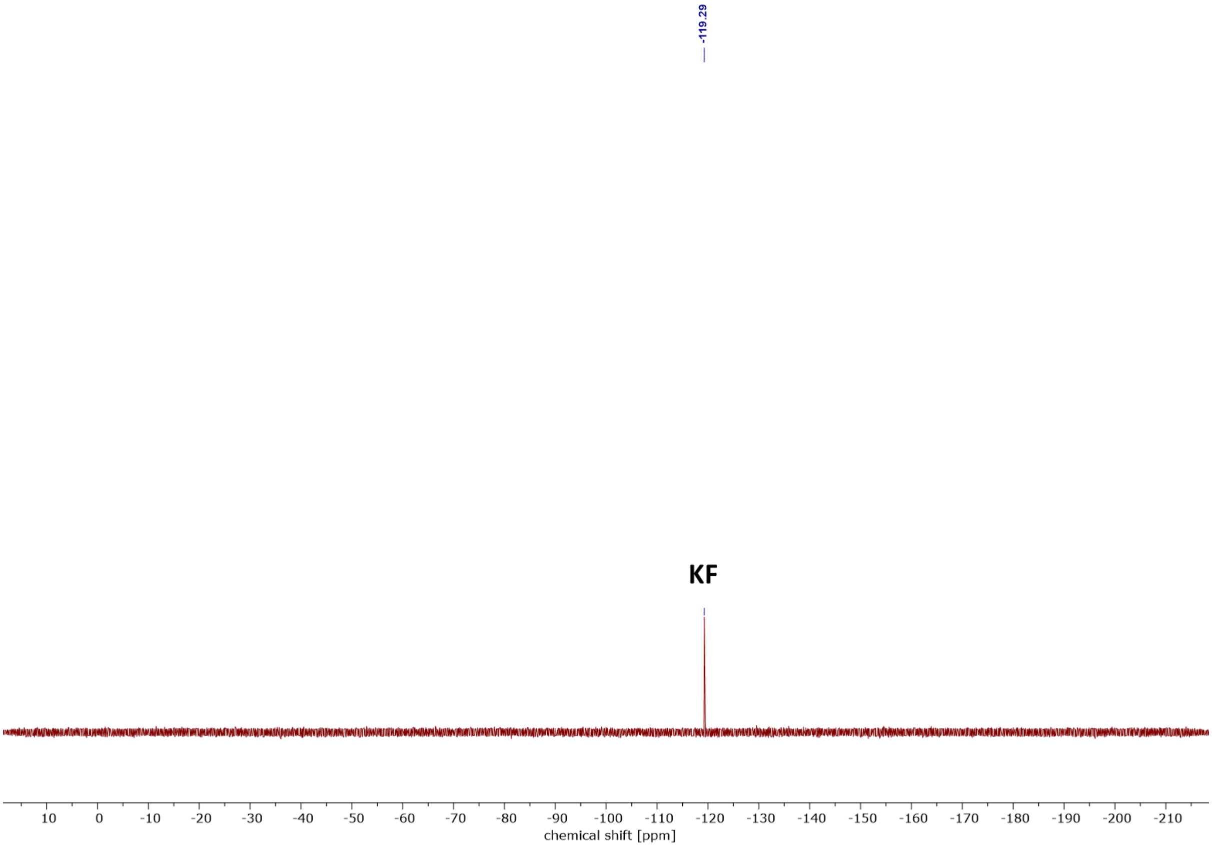

Figure S57: ^{19}F NMR (376.46 MHz, 298 K, H_2O) spectrum showing one signal, which can be attributed to KF. The spectrum was measured after the black solid residue on the filter was extracted with H_2O.

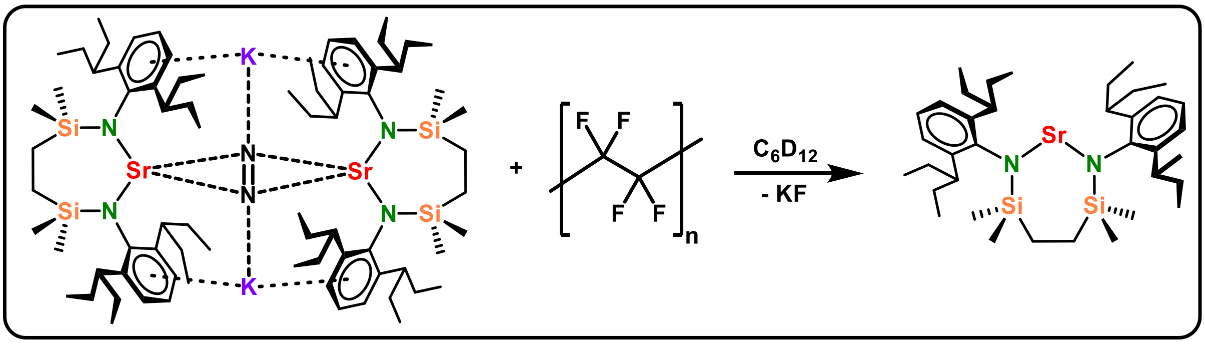

In a J-Young tube, a solution of **6**-Sr (30.0 mg, 20.1 µmol) in C_6D_{12} was added to PTFE powder (23.0 mg, 200 µmol, 1 µm). Vigorous gas release and darkening of the PTFE powder was observed immediately. After stirring for 5 min, the black suspension was filtrated and the ^{1}H NMR spectrum (**Figure S58**) of the greenish solution showed the formation of (^{DIPeP}NN)Sr and unknown byproducts. In the ^{19}F NMR spectrum (**Figure S59**) the presence of several unknown species can be observed. In addition, the black solid residue was washed again with pentane (1 mL), briefly dried *in vacuo* and subsequently extracted with H_2O (800 µL). The ^{19}F NMR spectrum (**Figure S60**) of the filtrated aqueous solution shows one NMR signal, which can be attributed to KF.

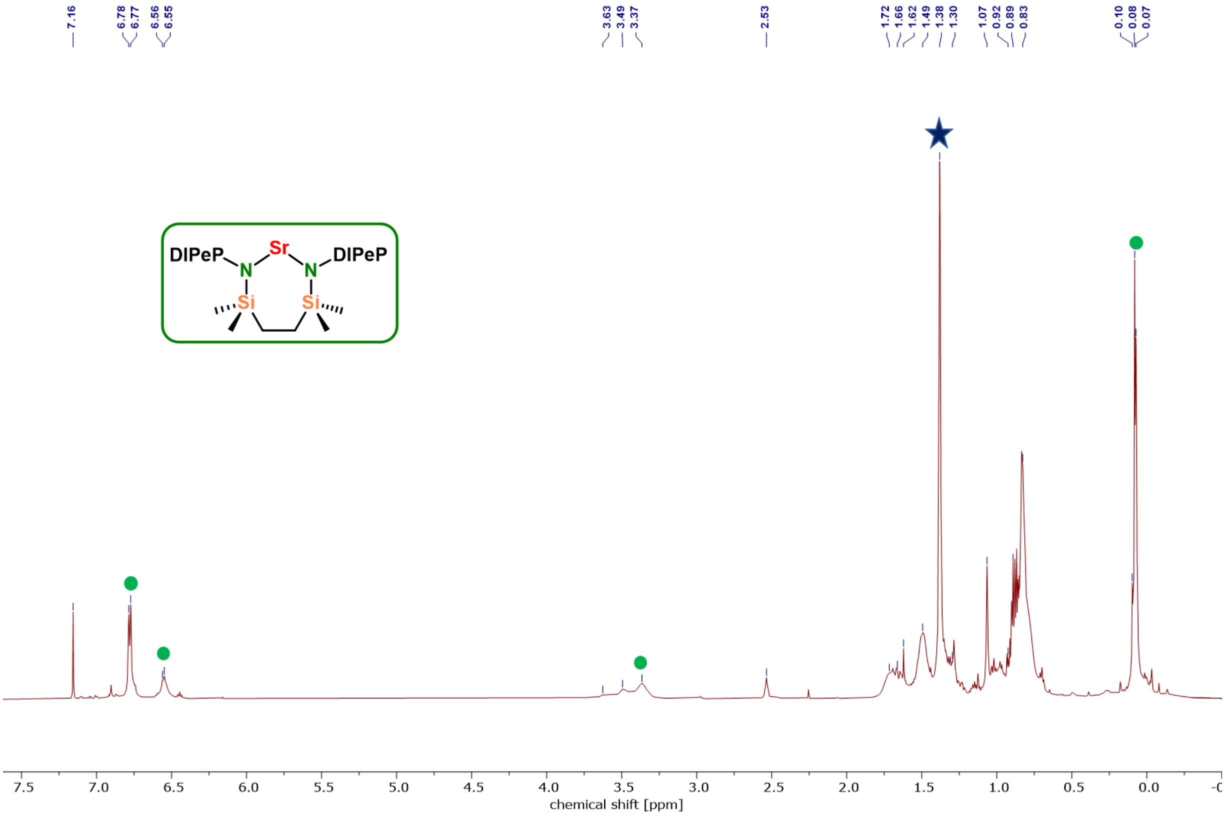

Figure S58: ^{1}H NMR (600.13 MHz, 298 K, C_6D_{12}) spectrum of the reaction between **6**-Sr and PTFE powder. The marked signals can be assigned to the (^{DIPeP}NN)Sr precursor complex. Solvent residual signals are marked with a star.

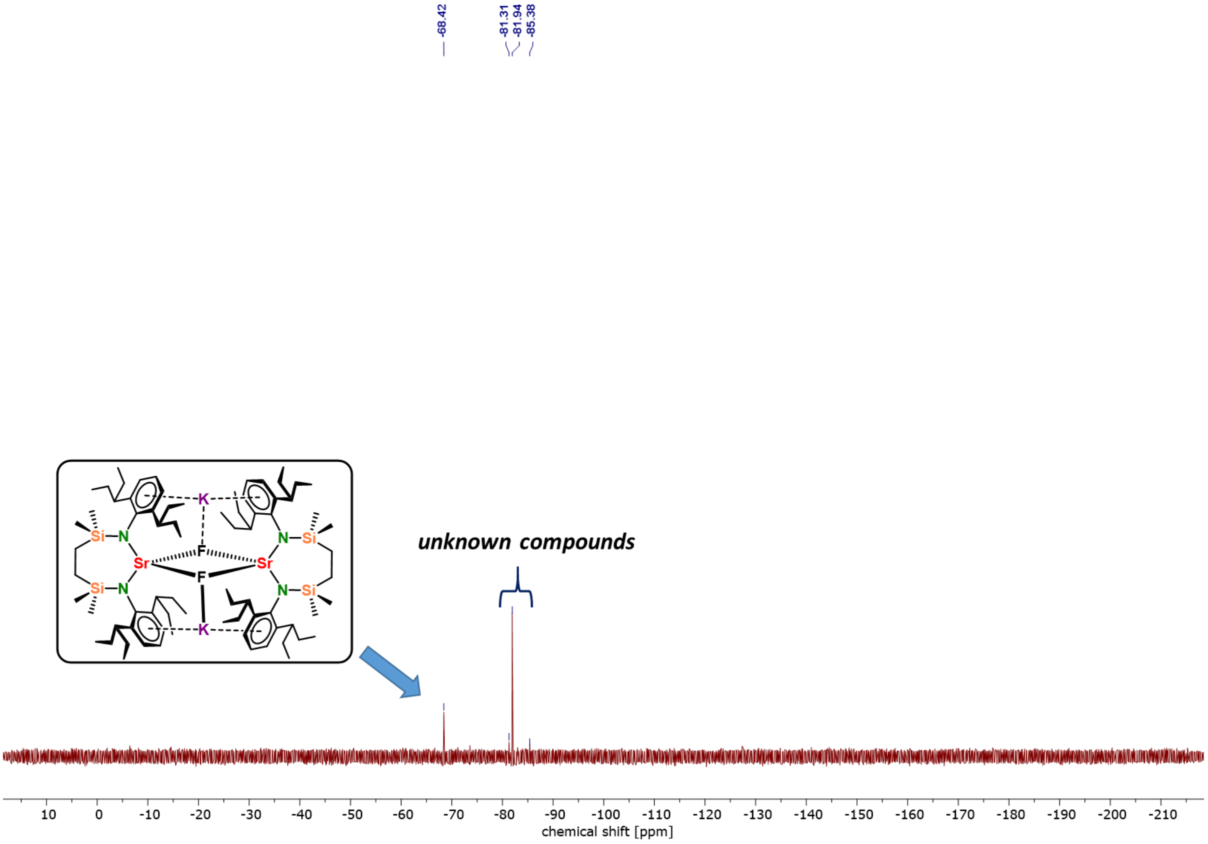

Figure S59: ^{19}F NMR (376.46 MHz, 298 K, C_6D_{12}) spectrum of the reaction between **6**-Sr and PTFE powder. The spectrum was measured after filtering off the black solid residue. The resonance signal appearing at -81.9 ppm can also be observed in **Figure S56**. The signal appearing at -68.4 ppm was also observed in **Figure S50** and can thus be assigned complex **7**. Over time, this signals disappears, which is indicative of decomposition.

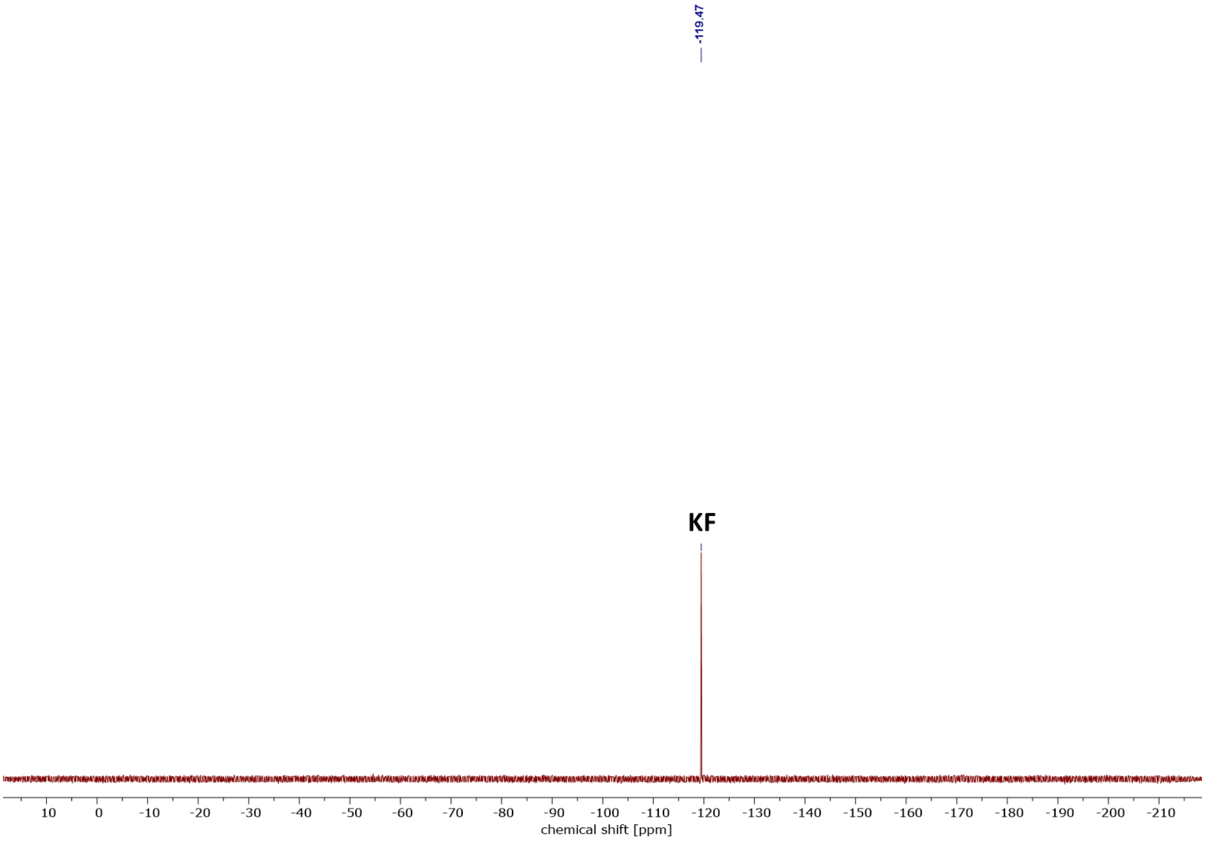

Figure S60: ^{19}F NMR (376.46 MHz, 298 K, H_2O) spectrum showing one signal, which can be attributed to KF. The spectrum was measured after the black solid residue on the filter was extracted with H_2O.

Reactivity of 6-Ca with H_2 and I_2

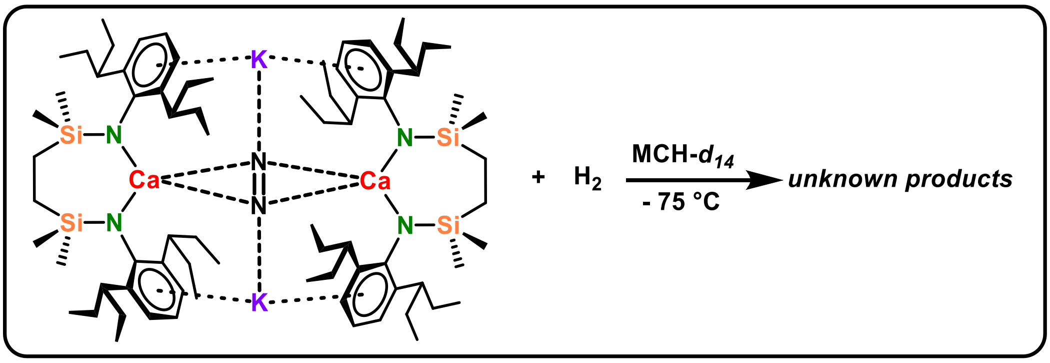

In a J-Young tube, **6**-Ca (35.0 mg, 25.0 µmol) was dissolved in methylcyclohexane-*d14* (650 µL) and the solution was degassed by freeze-pump thawing (2x). The solution was then cooled to -75 °C and pressurized with H_2 (1.5 bar). The reaction mixture was left standing at -75 °C. After 2 h, a color change from dark orange to yellow and precipitation of white solid was observed. The completion of the reaction was confirmed *via* ^{1}H NMR spectroscopy. The ^{1}H NMR spectrum (**Figure S61**) showed the formation of several unidentified products.

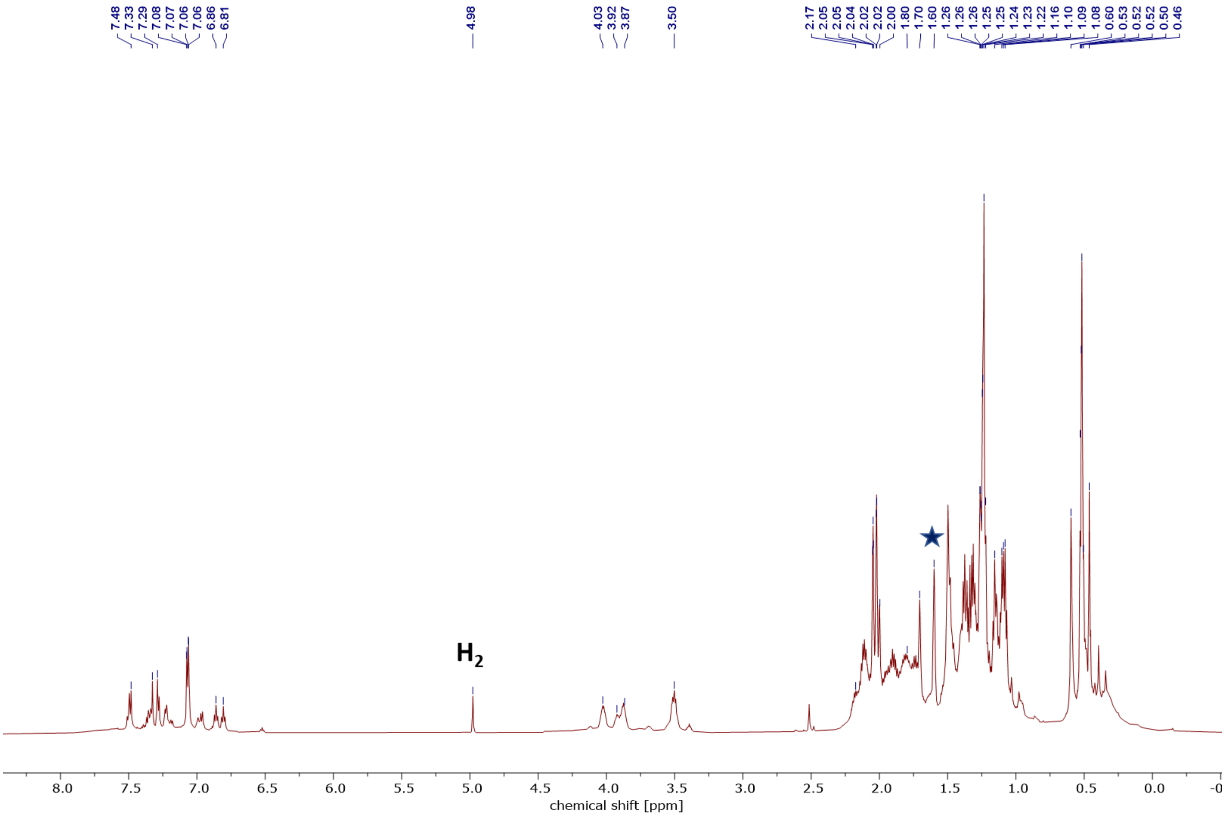

Figure S61: ^{1}H NMR spectrum (600.13 MHz, 298 K, C_7D_{14}) of the reaction between **6**-Ca and H_2. The formation of several unknown species can be observed in the spectrum. Solvent residual signals are marked with a star.

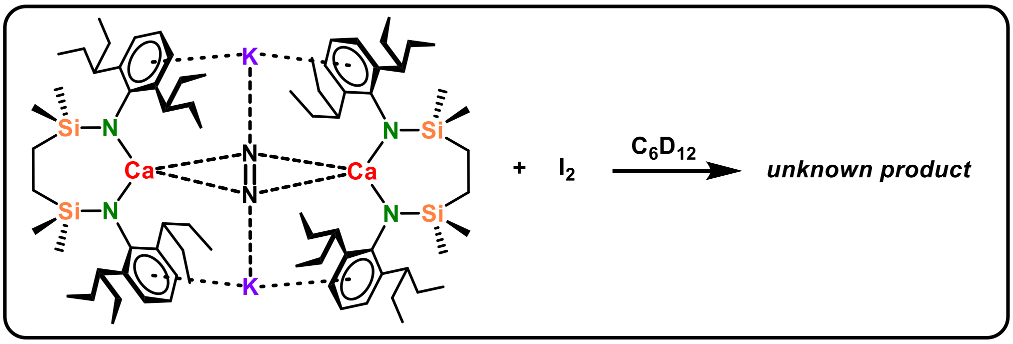

In a J-Young tube, **6**-Ca (40.0 mg, 28.6 µmol) and I_2 (7.30 mg, 28.8 µmol) were dissolved in C_6D_{12} (650 µL) upon which rapid gas release was visible. After 1 h of stirring, a color change from dark orange to yellow, as well as precipitation of solid was observed. The suspension was filtrated and the ^{1}H NMR spectrum (**Figure S62**) of the solution showed the formation of one unknown main product.

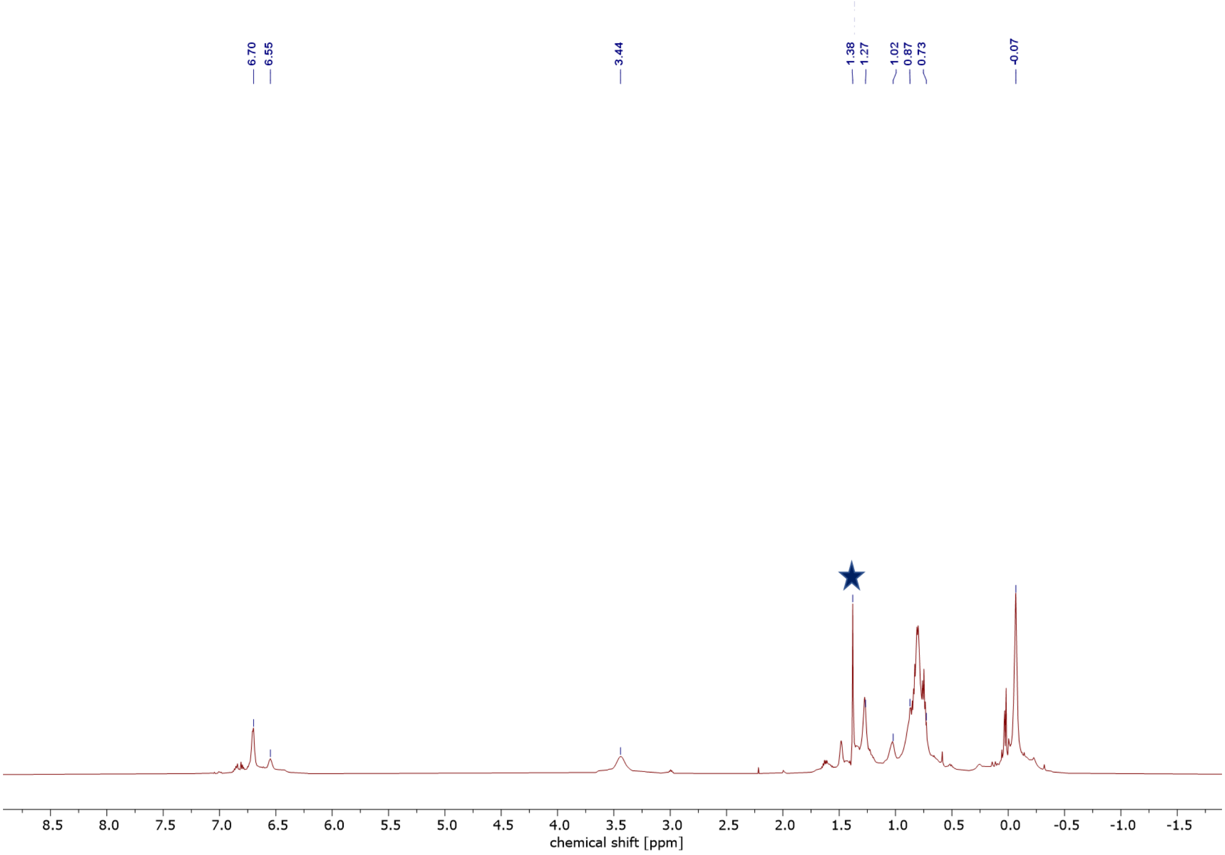

Figure S62: ^{1}H NMR (600.13 MHz, 298 K, C_6D_{12}) of the reaction between **6**-Ca and I_2. The spectrum shows the selective formation of one unknown product. Solvent residual signals are marked with a star.

Reactivity of 6-Sr with I_2 and H_2

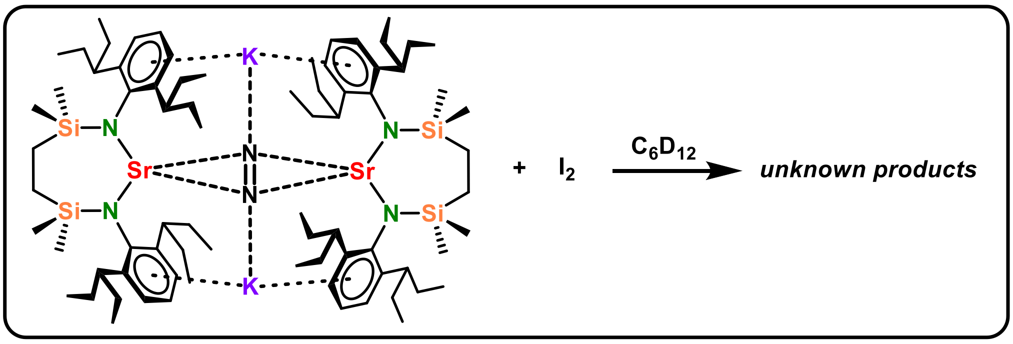

In a J-Young tube, **6**-Sr (40.0 mg, 26.7 µmol) and I_2 (6.80 mg, 26.7 mmol) were dissolved in C_6D_{12} (650 µL) upon which gas release was visible. After five minutes of stirring, the reaction mixture changed from a dark orange solution to a yellow suspension. The suspension was filtrated and the ^{1}H NMR spectrum (**Figure S63**) of the solution showed the formation of one main product with byproducts.

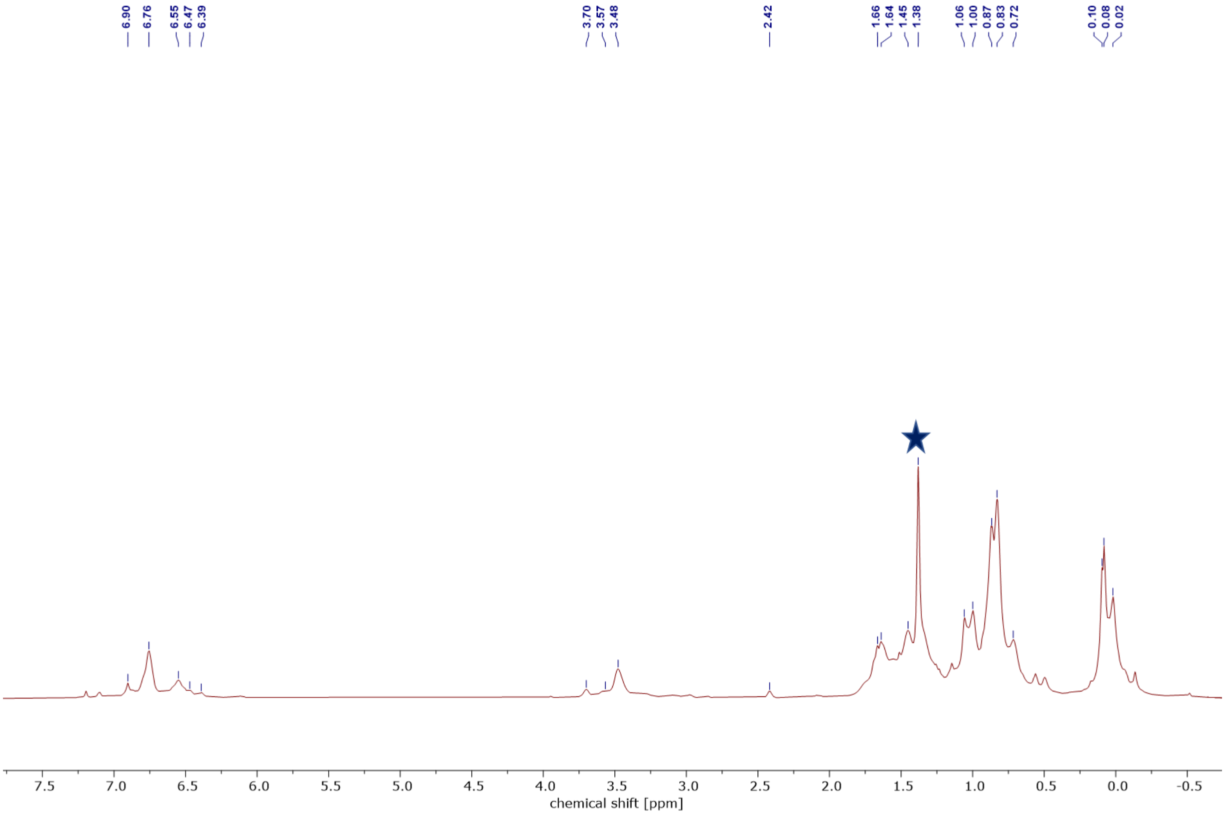

Figure S63: ^{1}H NMR (600.13 MHz, 298 K, C_6D_{12}) of the reaction between **6**-Ca and I_2. The spectrum shows the unselective formation of one main product with several byproducts. Solvent residual signals are marked with a star.

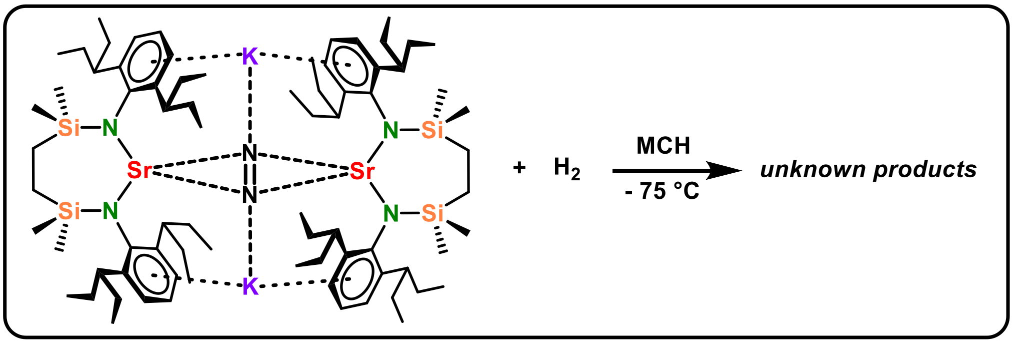

In a J-Young tube, **6**-Sr (35.0 mg, 23.4 µmol) was dissolved in methylcyclohexane-*d14* (650 µL) and the solution was degassed by freeze-pump thawing (2x). The solution was then cooled to -75 °C and subsequently pressurized with H_2 (1.5 bar). The reaction mixture was left standing at -75 °C. After 4 h the reaction mixture changed from a dark yellow solution to a brown suspension. The ^{1}H NMR spectrum (**Figure S64**) of the suspension showed the unselective formation of various unknown species.

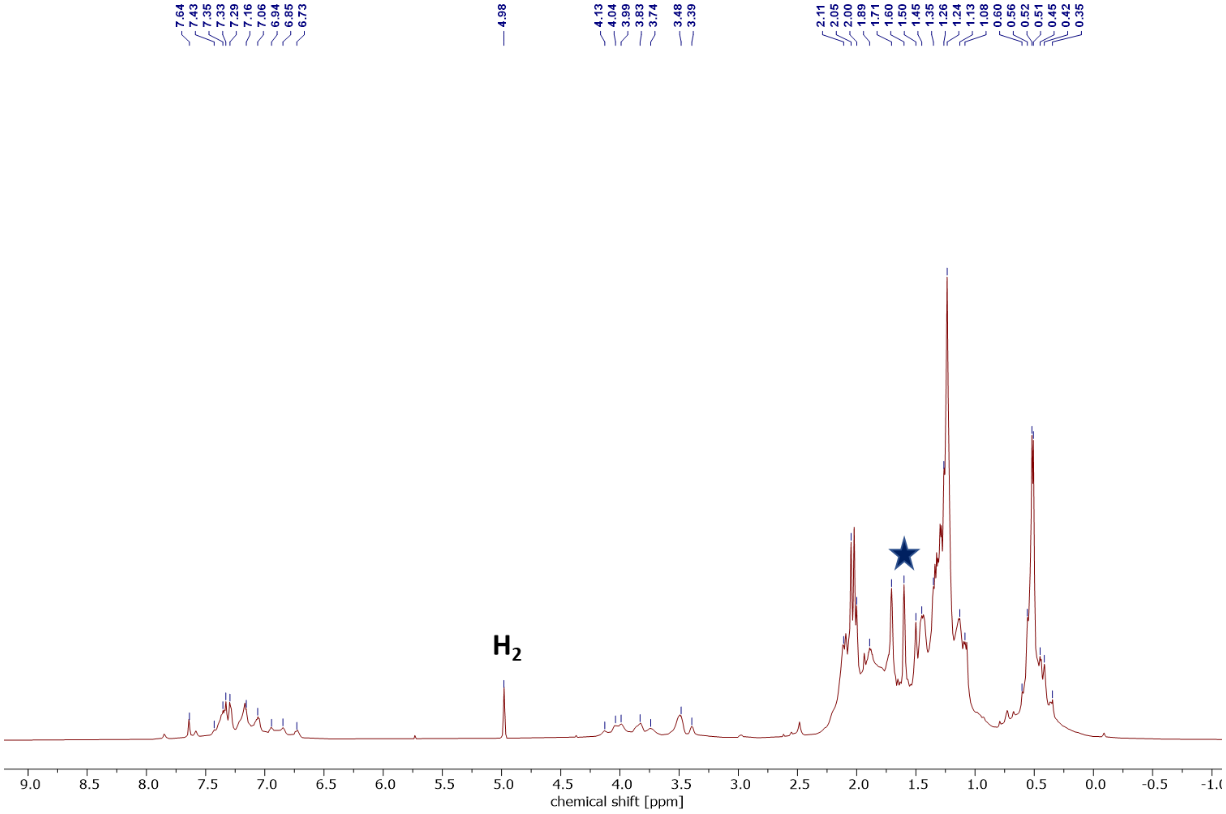

Figure S64: ^{1}H NMR spectrum (600.13 MHz, 298 K, C_7D_{14}) of the reaction between **6**-Ca and H_2. The formation of several unknown species can be observed in the spectrum. Solvent residual signals are marked with a star.

4. Crystal structure determination

Suitable single crystals of compounds **2**-**7** were embedded in protective perfluoropolyalkylether oil (viscosity 1800 cSt; ABCR GmbH) on a microscope slide and a single specimen was selected and subsequently transferred to the cold nitrogen gas stream of the diffractometer.

The intensity data was collected at 150K [K(^{DIPeP}NN)Ca]$_2$(N$_2$) or 100 K (all other compounds) using CuK_α radiation (λ = 1.54184 Å) on an Agilent SuperNova dual radiation diffractometer with microfocus X-ray sources and mirror optics. The measured data were processed with the CrysAlisPro software package.[S5] Data were corrected for Lorentz and polarization effects, and an empirical absorption correction using spherical harmonics as well as a numerical absorption correction based on gaussian integration over a multifaceted crystal model were applied. Using Olex2,[S6] the structures were solved by dual-space methods (SHELXT)[S7] and refined by full-matrix least-squares procedures on F^2 using SHELXL.[S8] All non-hydrogen atoms were refined with anisotropic displacement parameters. Most hydrogen atoms were placed in geometrically calculated positions and refined by using a riding model where each hydrogen atom was assigned a fixed isotropic displacement parameter with a value equal to 1.2U_{eq} (CH or CH$_2$) or 1.5U_{eq} (CH$_3$) of its parent C-atom.

In case of [(BDI*-H)Sr]$_2$(THF)$_2$, the asymmetric unit contained half of the molecule. The hydrogen atoms at C1 and C3 were placed in the positions indicated by a difference electron density map and refined together with isotropic displacement parameters. A minor disorder of the hydrogen atoms of a methyl group was observed and modeled. The site occupancy factors for the two alternative orientations were refined to 0.51(3) and 0.49(3).

The asymmetric unit of (BDI*)$_2$Sr(THF)$_2$ consisted of half of the molecule and disordered solvent. Although it seemed that the co-crystallized solvent was mostly *n*-pentane (a mixture of *n*-pentane, THF and *p*-xylene was used for crystallization), no satisfying disorder model was found. Therefore, the contribution of the disordered solvent to the structure factors was secured by back-Fourier transformation using the solvent mask routine[S9] of the program Olex2.[S6] The solvent accessible voids treated this way had a size of 771.7 Å^3 (9.7% of the unit cell) and contained 169.8 electrons per unit cell.

The hydrogen atom H3 noticeably deviated from the position, calculated *via* the riding model, and therefore was placed in the position indicated by a difference electron density map and refined together with isotropic displacement parameters.

The hydrogen atom at N1 in the molecular structure of (DIPeP)N(H)Li·$(THF)_2$ received the same treatment. The asymmetric unit of this compound consisted of half of the molecule, which contained a disordered THF ligand. The disorder was modeled with the help of similarity restraints (SADI, SIMU) and rigid bond restraints (RIGU),[S10] and led to site occupancy factors of 0.588(3) and 0.412(3).

Crystals of the ligand precursor (^{DIPeP}NN-H_2) contained half of the molecule per asymmetric unit. The hydrogen atom at N1 was placed in the position indicated by a difference electron density map and refined together with isotropic displacement parameters.

The molecular structure of (^{DIPeP}NN)K_2 showed signs of disorder. Three THF ligands were found to adopt two alternative positions each. The disorder was modeled with the help of similarity restraints (SADI, SIMU) and rigid bond restraints (RIGU),[S10] and led to site occupancy factors of 0.722(15)/0.278(15) (THF 1), 0.55(2)/0.45(2) (THF 2), and 0.551(14)/0.449(14) (THF 3), respectively.

The asymmetric unit of (^{DIPeP}NN)Sr$(THF)_2$ consisted of half of the molecule. The disorder of one of its 3-pentyl groups was modeled with the help of similarity restraints (SADI, SIMU) and rigid bond restraints (RIGU).[S10] The resulting site occupancy factors for the two alternative orientations were 0.763(7) and 0.237(7).

In case of [K(^{DIPeP}NN)Ca]$_2$(N_2), the asymmetric unit contained one fourth of the molecule. Disorder of the ligand backbone was observed and successfully modeled with the help of similarity restraints (SADI). This treatment led to site occupancy factors of 0.577(14) and 0.423(14) for the two alternative orientations of the moiety.

Disorder was also detected in case of [K(^{DIPeP}NN)Sr]$_2$(N_2). Here, one 3-pentyl group and the co-crystallized methylcyclohexane were affected. Similarity restraints (SADI, SIMU), rigid bond restraints (RIGU)[S10] as well as an ISOR restraint on C74a were applied during refinement of the disorder. While the site occupancy factors of the disordered 3-pentyl group were refined to 0.886(5) and 0.114(5), the occupancy of methylcyclohexane was constrained to 0.5 due to its disorder about a two-fold rotation axis.

The co-crystallized *n*-pentane within the crystal of [(^{DIPeP}NN)SrKF]$_2$ showed disorder as well. Here, the solvent molecule adopted three slightly different orientations with site occupancy factors of 0.625(3),

0.211(3) and 0.164(3). Similarity restraints (SIMU, SADI) were applied during the refinement of the disorder.

The crystal structure data of **2-7** has been deposited with the Cambridge Crystallographic Data Centre. CCDC 2424565-2424573 and 2426832 contain the supplementary crystallographic data for the complexes. This data can be obtained free of charge from The Cambridge Crystallographic Data Centre via www.ccdc.cam.ac.uk/data_request/cif.

Crystallographic and refinement data are summarized in Table S1.

Table S1. Crystal data and structure refinement for compounds **2-7**.

Compound	[(BDI*-H)Sr]$_2$(THF)$_2$ (**2**·(THF)$_2$)	(BDI*)$_2$Sr(THF)$_2$·*n*-pentane (**3**·(THF)$_2$)	(DIPeP)N(H)Li·(THF)$_2$
Identification code	hasj210916b	hasj210827b	hasj240422a
Empirical formula	$C_{82}H_{128}N_4O_2Sr_2$	$C_{82}H_{130}N_4O_2Sr$ a)	$C_{40}H_{68}Li_2N_2O_2$
Formula weight	1377.12	1291.51 a)	622.84
Temperature/K	100.0(6)	100.0(6)	99.97(13)
Crystal system	triclinic	monoclinic	triclinic
Space group	P-1	I2/a	P-1
a/Å	11.5181(4)	17.8060(3)	9.4229(4)
b/Å	13.2032(5)	17.5267(3)	9.6980(5)
c/Å	13.9772(5)	26.1483(4)	11.4686(5)
α/°	115.479(4)	90	108.973(4)
β/°	92.540(3)	102.1375(18)	91.360(3)
γ/°	97.800(3)	90	101.356(4)
Volume/Å^3	1888.54(13)	7977.9(2)	967.33(7)
Z	1	4	1
ρ_{calc}g/cm^3	1.211	1.075 a)	1.069
μ/mm^{-1}	2.211	1.266 a)	0.476
F(000)	740.0	2816.0 a)	344.0
Crystal size/mm^3	0.335 × 0.317 × 0.144	0.337 × 0.216 × 0.139	0.351 × 0.295 × 0.028
Radiation	Cu Kα (λ = 1.54184)	Cu Kα (λ = 1.54184)	Cu Kα (λ = 1.54184)
2Θ range for data collection/°	7.052 to 145.7	6.916 to 151.834	8.19 to 144.972
Index ranges	-14 ≤ h ≤ 11, -16 ≤ k ≤ 12, -16 ≤ l ≤ 17	-21 ≤ h ≤ 22, -21 ≤ k ≤ 15, -25 ≤ l ≤ 32	-11 ≤ h ≤ 11, -11 ≤ k ≤ 11, -14 ≤ l ≤ 8
Reflections collected	12260	17045	7222
Independent reflections	7204 [R_{int} = 0.0252, R_{sigma} = 0.0354]	8100 [R_{int} = 0.0262, R_{sigma} = 0.0322]	3722 [R_{int} = 0.0267, R_{sigma} = 0.0349]
Data/restraints/parameters	7204/0/428	8100/0/416	3722/289/263
Goodness-of-fit on F^2	1.056	1.041	1.046
Final R indexes [I>=2σ (I)]	R_1 = 0.0319, wR_2 = 0.0762	R_1 = 0.0415, wR_2 = 0.1088	R_1 = 0.0494, wR_2 = 0.1262
Final R indexes [all data]	R_1 = 0.0385, wR_2 = 0.0817	R_1 = 0.0443, wR_2 = 0.1119	R_1 = 0.0587, wR_2 = 0.1351
Largest diff. peak/hole / e Å^{-3}	1.16/-0.53	0.68/-0.49	0.22/-0.20
CCDC number	2424565	2424566	2424567

a) contribution of the masked disordered solvent neglected

Table S1. Crystal data and structure refinement for compounds **2-7** (continued).

Compound	(^{DIPeP}NN-H_2)	(^{DIPeP}NN)K_2(THF)$_2$ (**4**·(THF)$_2$)	(^{DIPeP}NN)Sr(THF)$_2$ (**5**-Sr)·(THF)$_2$
Identification code	hasj230809b	hasj240515a	hasj231018a
Empirical formula	$C_{38}H_{68}N_2Si_2$	$C_{62}H_{114}K_2N_2O_6Si_2$	$C_{46}H_{82}N_2O_2Si_2Sr$
Formula weight	609.12	1117.93	838.93
Temperature/K	100.00(10)	99.95(19)	99.98(10)
Crystal system	triclinic	monoclinic	tetragonal
Space group	P-1	P2$_1$/c	I4$_1$/a
a/Å	8.1382(4)	21.1311(3)	20.7535(2)
b/Å	10.3465(6)	14.3341(2)	20.7535(2)
c/Å	12.2033(7)	21.9640(3)	22.1131(2)
α/°	80.033(5)	90	90
β/°	75.827(5)	95.499(2)	90
γ/°	79.705(5)	90	90
Volume/Å^3	971.18(10)	6622.17(16)	9524.3(2)
Z	1	4	8
ρ_{calc}g/cm^3	1.041	1.121	1.170
μ/mm^{-1}	1.004	1.967	2.317
F(000)	338.0	2456.0	3632.0
Crystal size/mm^3	0.772 × 0.447 × 0.391	0.273 × 0.133 × 0.108	0.509 × 0.374 × 0.248
Radiation	Cu Kα (λ = 1.54184)	Cu Kα (λ = 1.54184)	Cu Kα (λ = 1.54184)
2Θ range for data collection/°	7.542 to 144.614	7.374 to 145.25	8.522 to 145.07
Index ranges	-9 ≤ h ≤ 4, -12 ≤ k ≤ 12, -14 ≤ l ≤ 14	-24 ≤ h ≤ 26, -16 ≤ k ≤ 17, -25 ≤ l ≤ 26	-25 ≤ h ≤ 25, -20 ≤ k ≤ 21, -27 ≤ l ≤ 27
Reflections collected	6178	36860	22879
Independent reflections	3701 [R_{int} = 0.0151, R_{sigma} = 0.0187]	12813 [R_{int} = 0.0323, R_{sigma} = 0.0329]	4620 [R_{int} = 0.0296, R_{sigma} = 0.0190]
Data/restraints/parameters	3701/0/200	12813/313/781	4620/296/294
Goodness-of-fit on F^2	1.062	1.109	1.076
Final R indexes [I>=2σ (I)]	R_1 = 0.0336, wR_2 = 0.0870	R_1 = 0.0511, wR_2 = 0.1207	R_1 = 0.0367, wR_2 = 0.0947
Final R indexes [all data]	R_1 = 0.0351, wR_2 = 0.0882	R_1 = 0.0613, wR_2 = 0.1264	R_1 = 0.0404, wR_2 = 0.0975
Largest diff. peak/hole / e Å^{-3}	0.25/-0.21	0.42/-0.29	0.53/-0.61
CCDC number	2424568	2424569	2424570

a) contribution of the masked disordered solvent neglected

Table S1. Crystal data and structure refinement for compounds **2-7** (continued).

Compound	(^{DIPeP}NN)Ca (**5**-Ca)	[K(^{DIPeP}NN)Ca]$_2$(N$_2$) (**6**-Ca)	[K(^{DIPeP}NN)Sr]$_2$(N$_2$)·0.5methylcyclohexane (**6**-Sr)
Identification code	hasj230818a	hasj240904a	hasj241128a
Empirical formula	$C_{38}H_{66}N_2Si_2Ca$	$C_{76}H_{132}Ca_2K_2N_6Si_4$	$C_{79.5}H_{139}K_2N_6Si_4Sr_2$
Formula weight	647.18	1400.59	1544.76
Temperature/K	100.00(11)	150.00(10)	100.0(6)
Crystal system	triclinic	orthorhombic	monoclinic
Space group	P-1	Fddd	I2/a
a/Å	10.4328(2)	16.0086(9)	23.2446(5)
b/Å	13.4366(3)	21.5038(12)	13.8416(2)
c/Å	14.8056(3)	48.686(2)	53.5421(7)
α/°	106.511(2)	90	90
β/°	90.633(2)	90	100.8274(17)
γ/°	100.604(2)	90	90
Volume/Å^3	1951.48(7)	16759.9(15)	16920.1(5)
Z	2	8	8
ρ_{calc}g/cm^3	1.101	1.110	1.213
μ/mm^{-1}	2.157	2.923	3.402
F(000)	712.0	6112.0	6624.0
Crystal size/mm^3	0.372 × 0.287 × 0.182	0.184 × 0.109 × 0.033	0.215 × 0.158 × 0.01
Radiation	Cu Kα (λ = 1.54184)	Cu Kα (λ = 1.54184)	Cu Kα (λ = 1.54184)
2Θ range for data collection/°	6.996 to 145.384	7.12 to 145.562	7.468 to 144.222
Index ranges	-12 ≤ h ≤ 12, -16 ≤ k ≤ 16, -18 ≤ l ≤ 18	-19 ≤ h ≤ 14, -26 ≤ k ≤ 24, -59 ≤ l ≤ 30	-25 ≤ h ≤ 28, -17 ≤ k ≤ 16, -65 ≤ l ≤ 65
Reflections collected	39376	10382	56565
Independent reflections	7600 [R_{int} = 0.0389, R_{sigma} = 0.0246]	4089 [R_{int} = 0.0520, R_{sigma} = 0.0634]	16246 [R_{int} = 0.0533, R_{sigma} = 0.0514]
Data/restraints/parameters	7600/0/400	4089/35/241	16246/338/947
Goodness-of-fit on F^2	1.065	1.063	1.012
Final R indexes [I>=2σ (I)]	R_1 = 0.0357, wR_2 = 0.1033	R_1 = 0.0695, wR_2 = 0.1743	R_1 = 0.0455, wR_2 = 0.1126
Final R indexes [all data]	R_1 = 0.0375, wR_2 = 0.1053	R_1 = 0.1045, wR_2 = 0.2131	R_1 = 0.0635, wR_2 = 0.1231
Largest diff. peak/hole / e Å^{-3}	0.52/-0.45	0.66/-0.89	1.23/-0.89
CCDC number	2424571	2424572	2424573

a) contribution of the masked disordered solvent neglected

Table S1. Crystal data and structure refinement for compounds **2-7** (continued).

Compound	[(^{DIPeP}NN)SrKF]$_2$·*n*-pentane (**7**)
Identification code	hasj240513a
Empirical formula	$C_{81}H_{144}F_2K_2N_4Si_4Sr_2$
Formula weight	1577.79
Temperature/K	99.98(13)
Crystal system	triclinic
Space group	P-1
a/Å	14.2400(3)
b/Å	14.3481(4)
c/Å	23.0561(5)
α/°	87.580(2)
β/°	74.532(2)
γ/°	70.275(2)
Volume/Å^3	4267.79(19)
Z	2
ρ_{calc}g/cm^3	1.228
μ/mm^{-1}	3.409
F(000)	1692.0
Crystal size/mm^3	0.218 × 0.196 × 0.169
Radiation	Cu Kα (λ = 1.54184)
2Θ range for data collection/°	6.846 to 145.504
Index ranges	-17 ≤ h ≤ 17, -17 ≤ k ≤ 17, -20 ≤ l ≤ 28
Reflections collected	64294
Independent reflections	16671 [R_{int} = 0.0318, R_{sigma} = 0.0261]
Data/restraints/parameters	16671/583/979
Goodness-of-fit on F^2	1.021
Final R indexes [I>=2σ (I)]	R_1 = 0.0310, wR_2 = 0.0761
Final R indexes [all data]	R_1 = 0.0345, wR_2 = 0.0786
Largest diff. peak/hole / e Å^{-3}	1.36/-0.61
CCDC number	2426832

a) contribution of the masked disordered solvent neglected

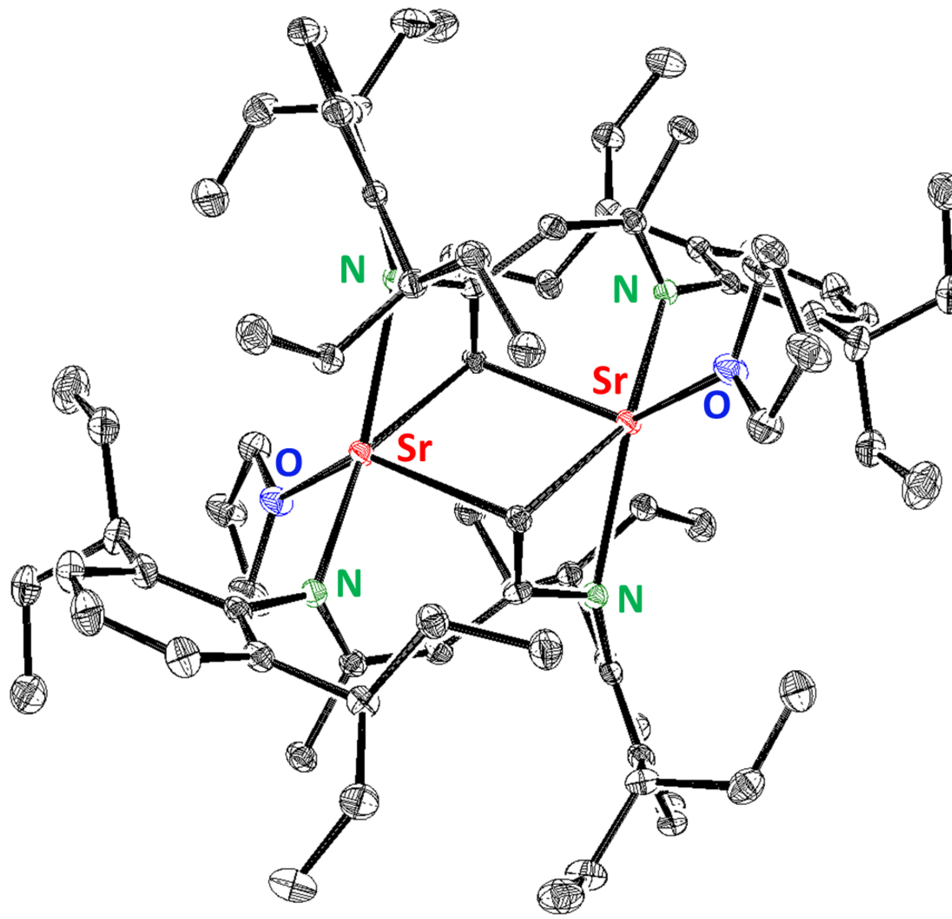

Figure S65: ORTEP representation **2**·(THF)$_2$ with atomic displacement ellipsoids set at 50% probability. Hydrogen atoms are omitted for clarity.

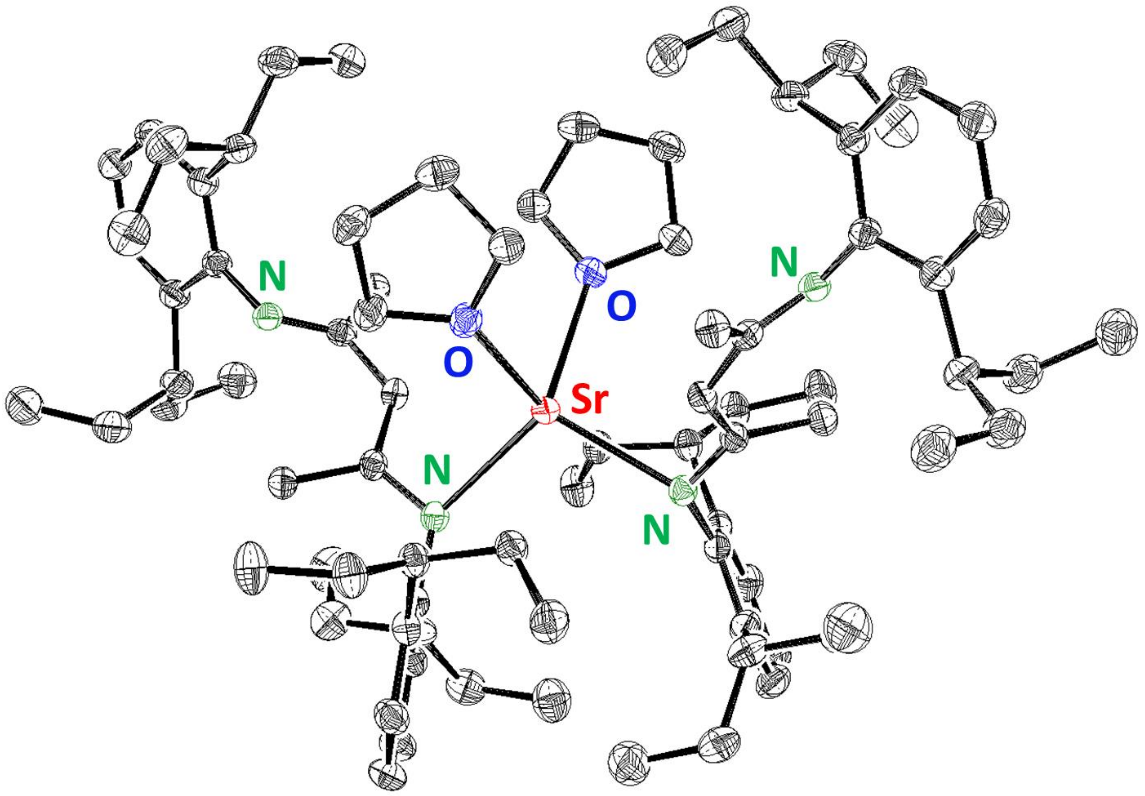

Figure S66: ORTEP representation **3**·(THF)$_2$ with atomic displacement ellipsoids set at 50% probability. Hydrogen atoms are omitted for clarity.

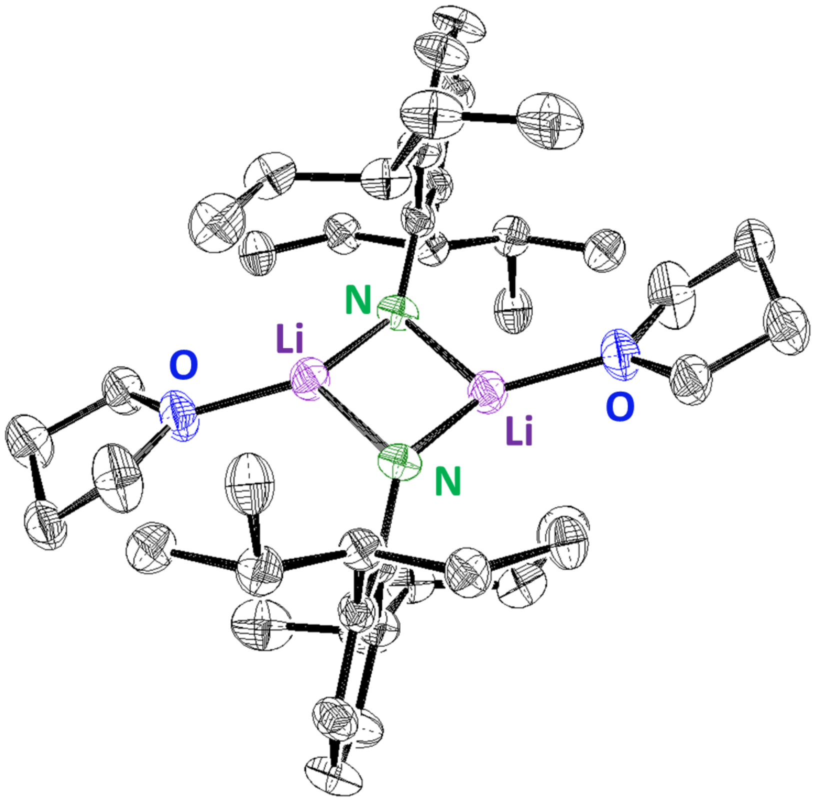

Figure S67: ORTEP representation of (DIPeP)N(H)Li·$(THF)_2$ with atomic displacement ellipsoids set at 50% probability. Hydrogen atoms are omitted for clarity.

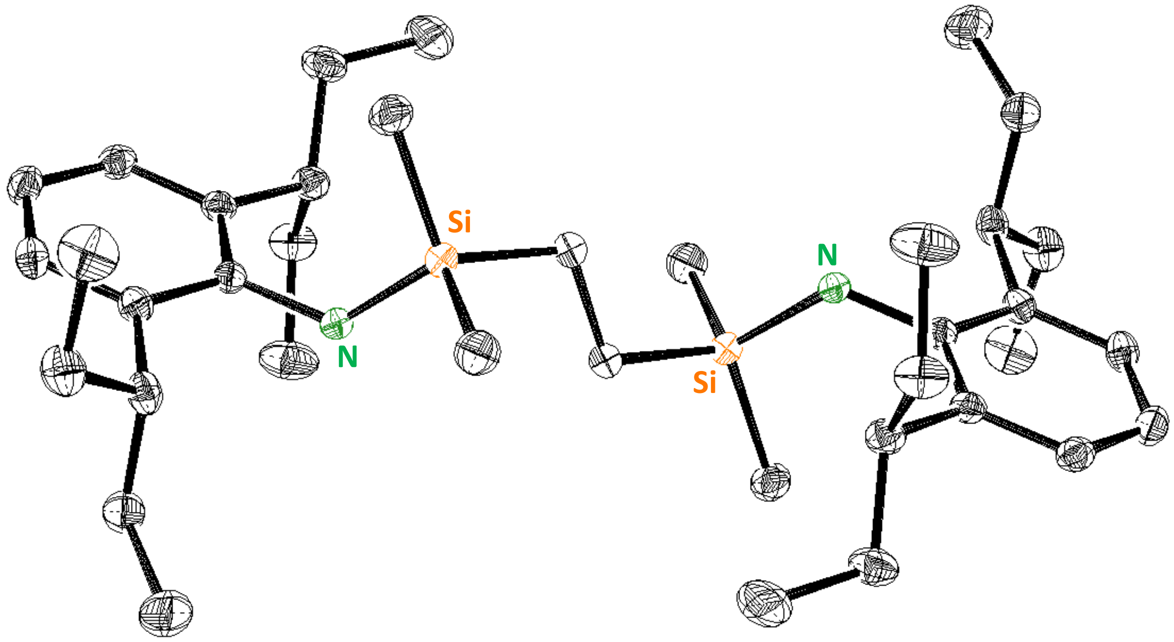

Figure S68: ORTEP representation of ($^{DIPeP}NN-H_2$) with atomic displacement ellipsoids set at 50% probability. Hydrogen atoms are omitted for clarity.

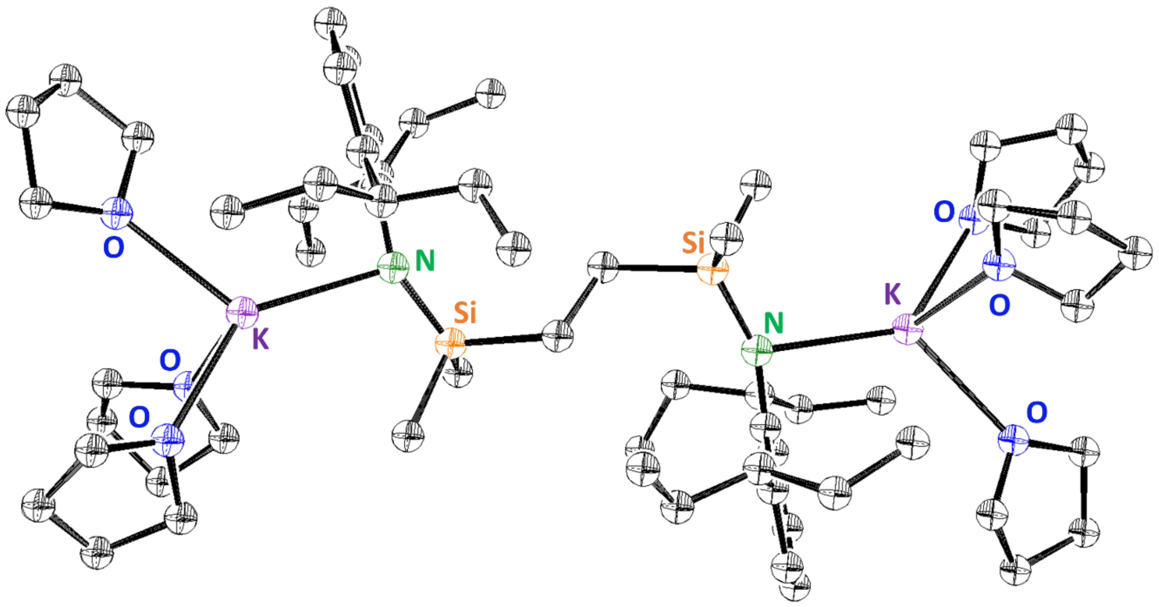

Figure S69: ORTEP representation of **4**·$(THF)_2$ with atomic displacement ellipsoids set at 50% probability. Hydrogen atoms are omitted for clarity.

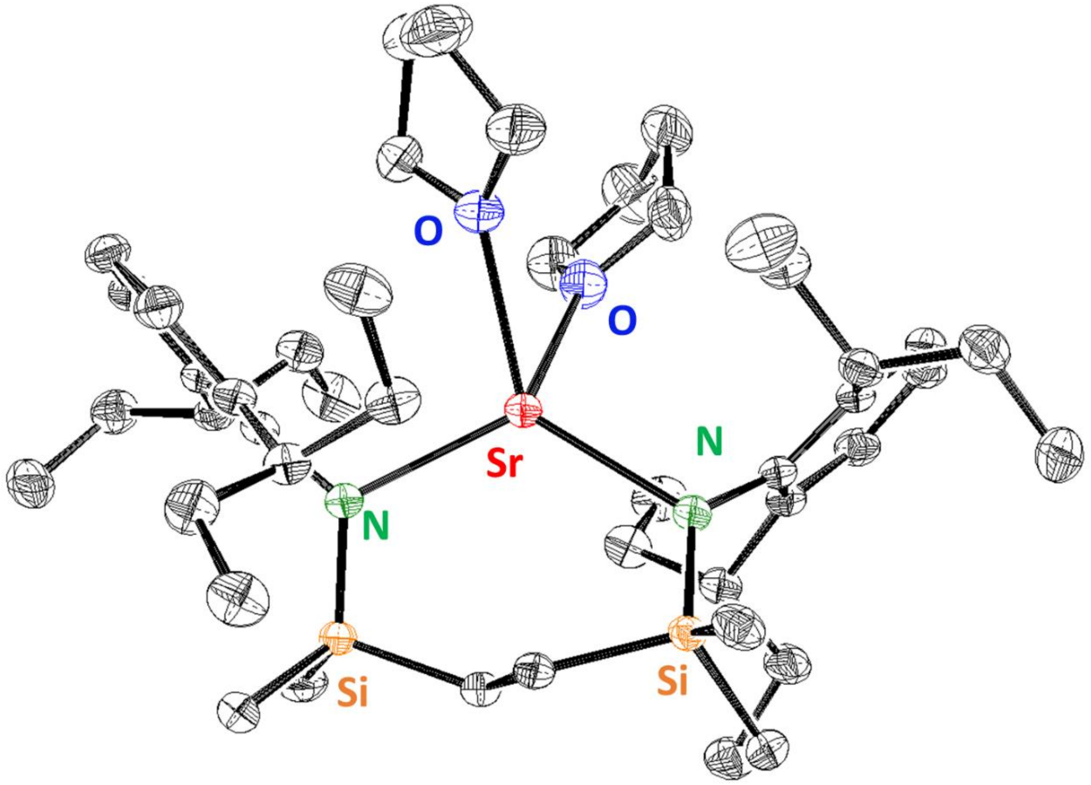

Figure S70: ORTEP representation of **5**-Sr·$(THF)_2$ with atomic displacement ellipsoids set at 50% probability. Hydrogen atoms are omitted for clarity.

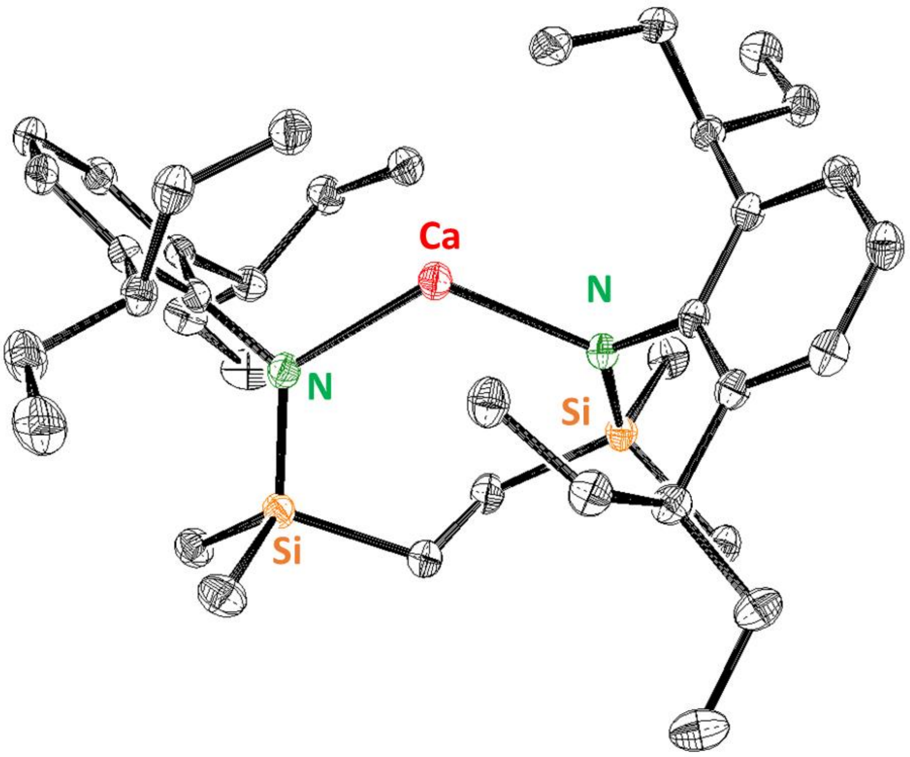

Figure S71: ORTEP representation of **5**-Ca with atomic displacement ellipsoids set at 50% probability. Hydrogen atoms are omitted for clarity.

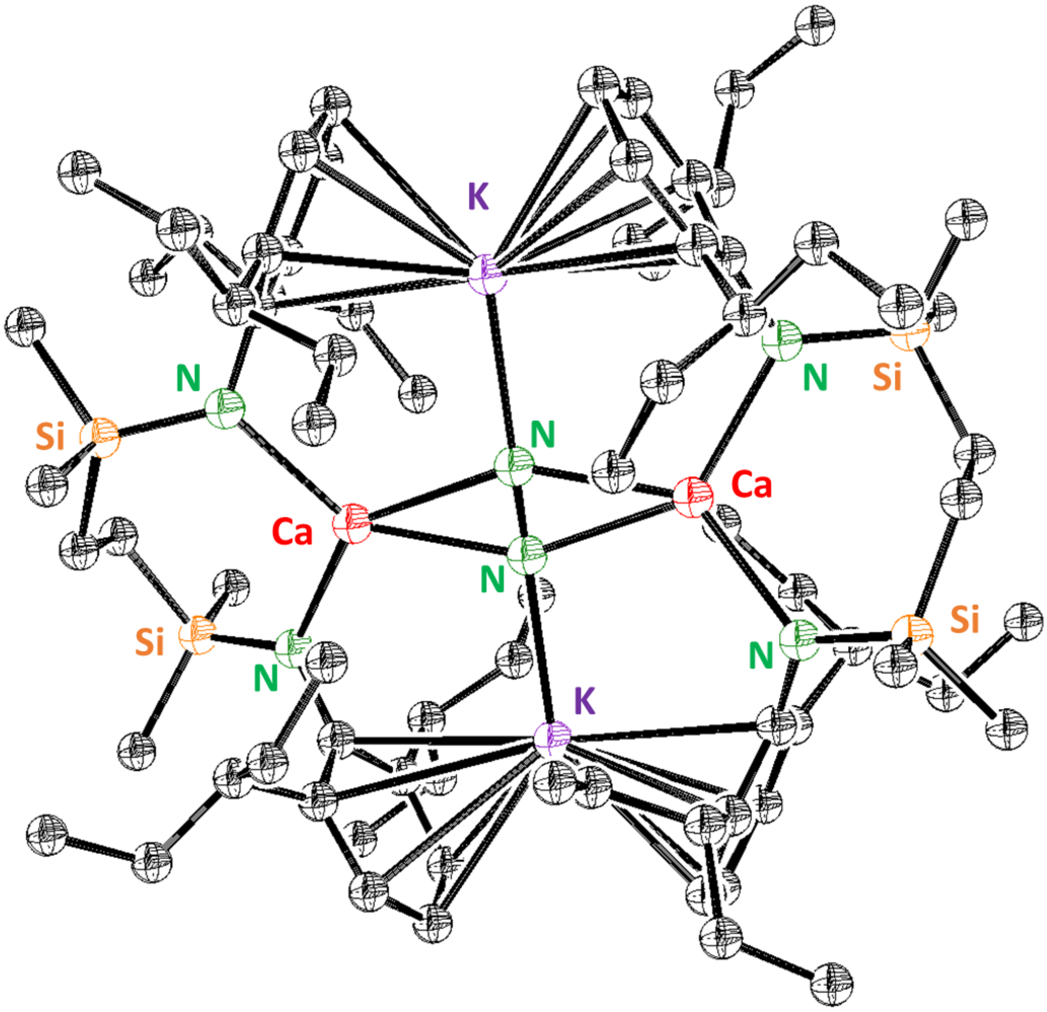

Figure S72: ORTEP representation of **6**-Ca with atomic displacement ellipsoids set at 50% probability. Hydrogen atoms are omitted for clarity.

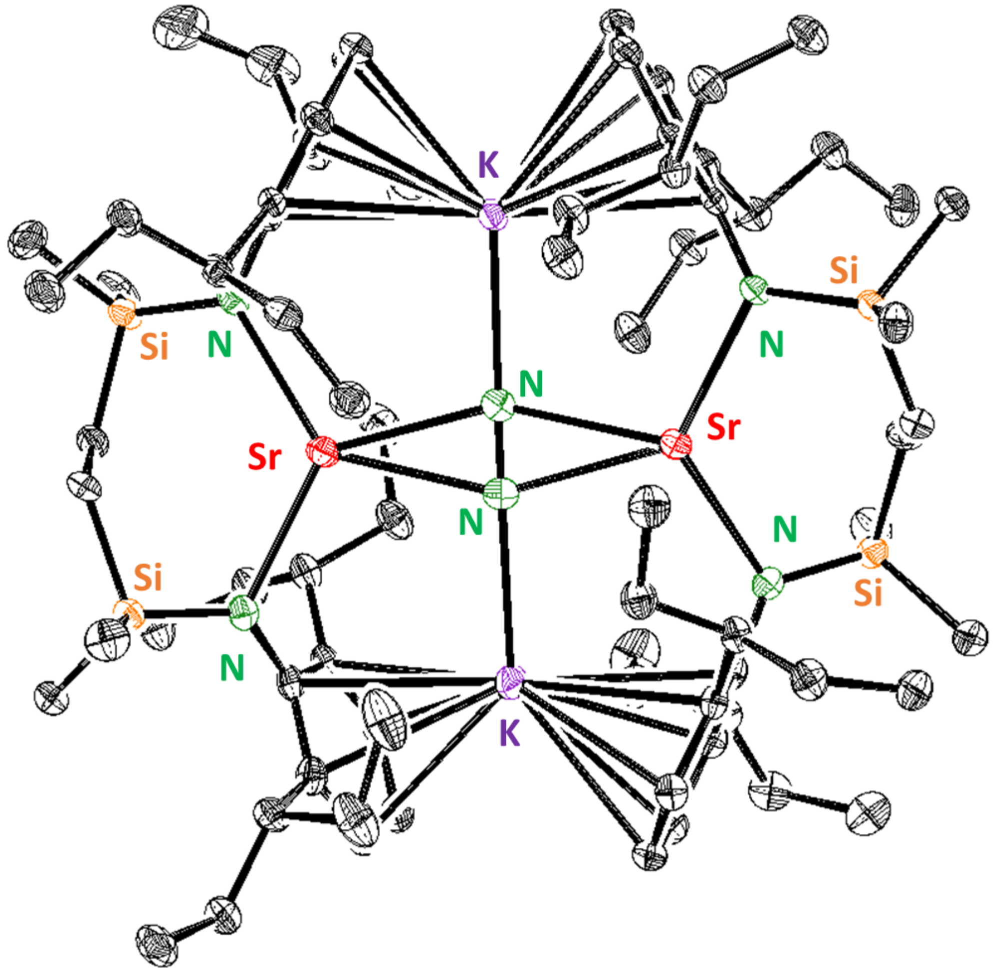

Figure S73: ORTEP representation of **6**-Sr with atomic displacement ellipsoids set at 50% probability. Hydrogen atoms are omitted for clarity.

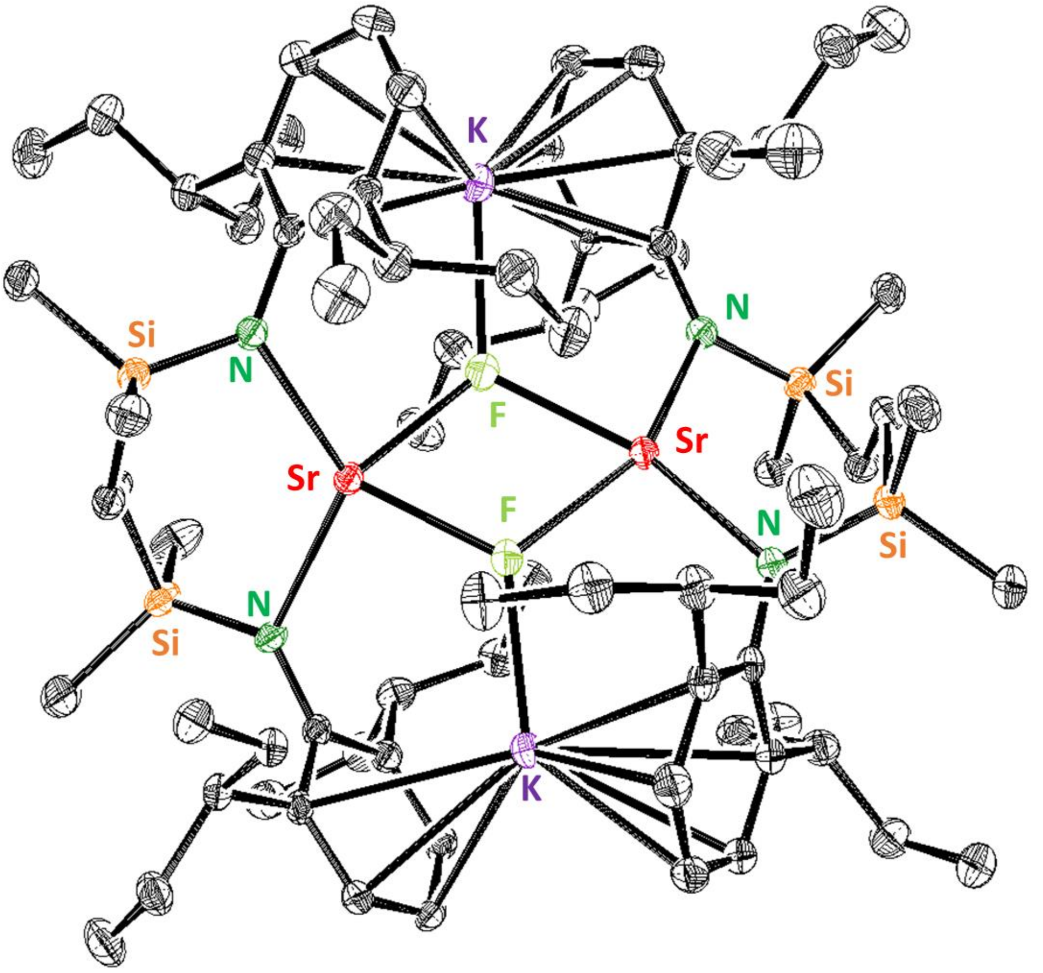

Figure S74: ORTEP representation of **7** with atomic displacement ellipsoids set at 50% probability. Hydrogen atoms are omitted for clarity

.

5. DFT calculations

All calculations were carried out using Gaussian 16A.[S11] All methods were used as implemented. All structures were fully optimized at a B3PW91/def2SVP level of theory.[S12–S15] All structures were characterized as true minima (Nimag=0) or as transition states (Nimag=1) by frequency calculations on the same level of theory. Energies were determined at a B3PW91/def2TZVP level of theory. In all cases Grimme`s third dispersion correction with Becke-Johnson damping (GD3BJ) was applied if not stated otherwise.[S16] NPA charges were calculated via NBO7 analyses.[S17] All structures were evaluated using Molecule 2.3.[S16] Topological analyses were carried out using AIMAll (v17).[S18-S19]

Calculations on (BDI)AeAe(BDI) complexes

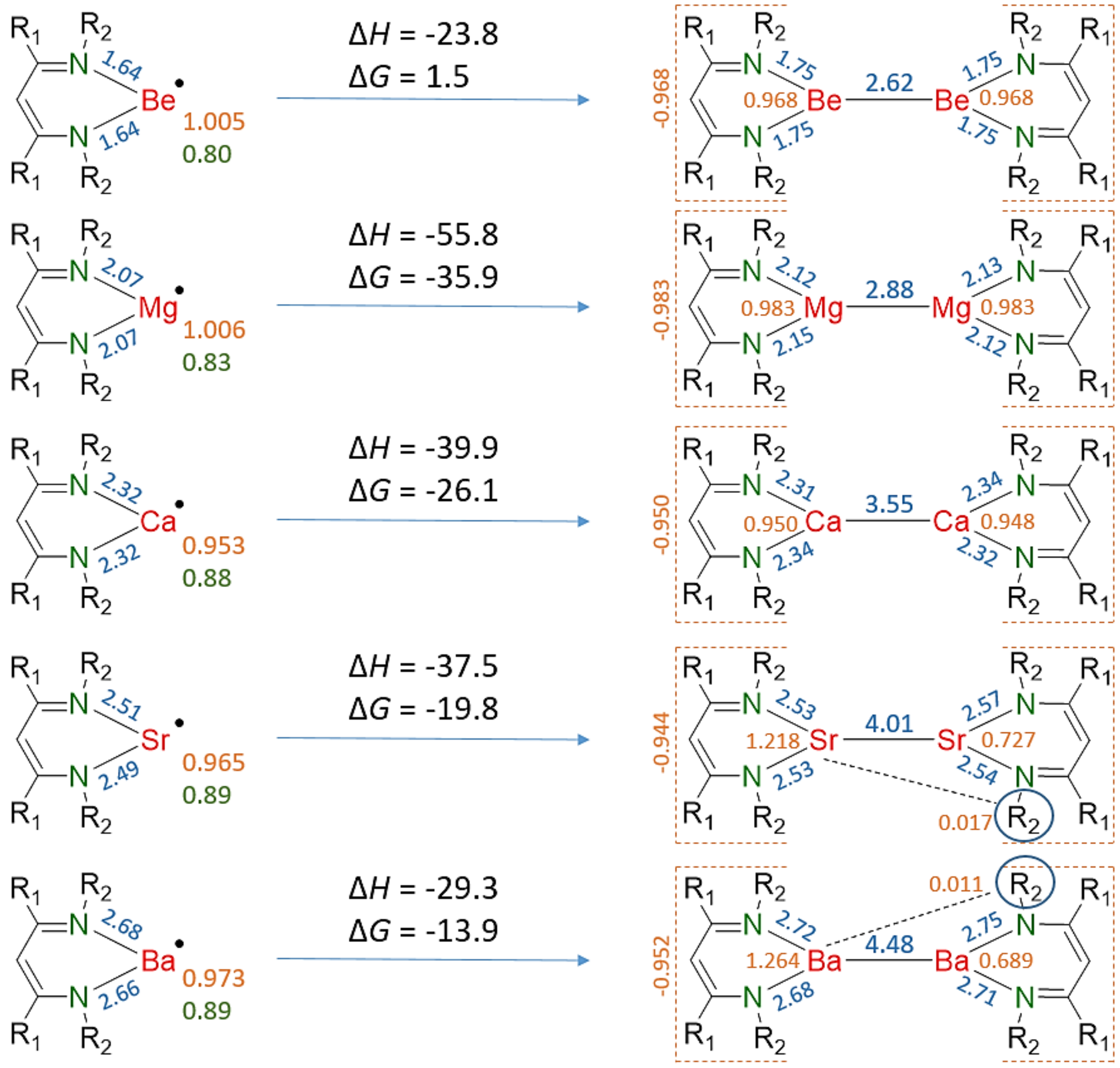

Figure S75: Reaction energies for the formation of (BDI)AeAe(BDI) from their corresponding (BDI)Ae radicals. Level of theory used B3PW91/def2TZVP//def2SVP **with Grimme's dispersion**. Energy is given in kcal/mol; Bond length in Å (blue color); NPA charger are given as number of electrons (orange color); spin density is in green color, dotted orange brackets shows collective partial NPA charger on ligand. (R_1= Me, R_2=DIPP)

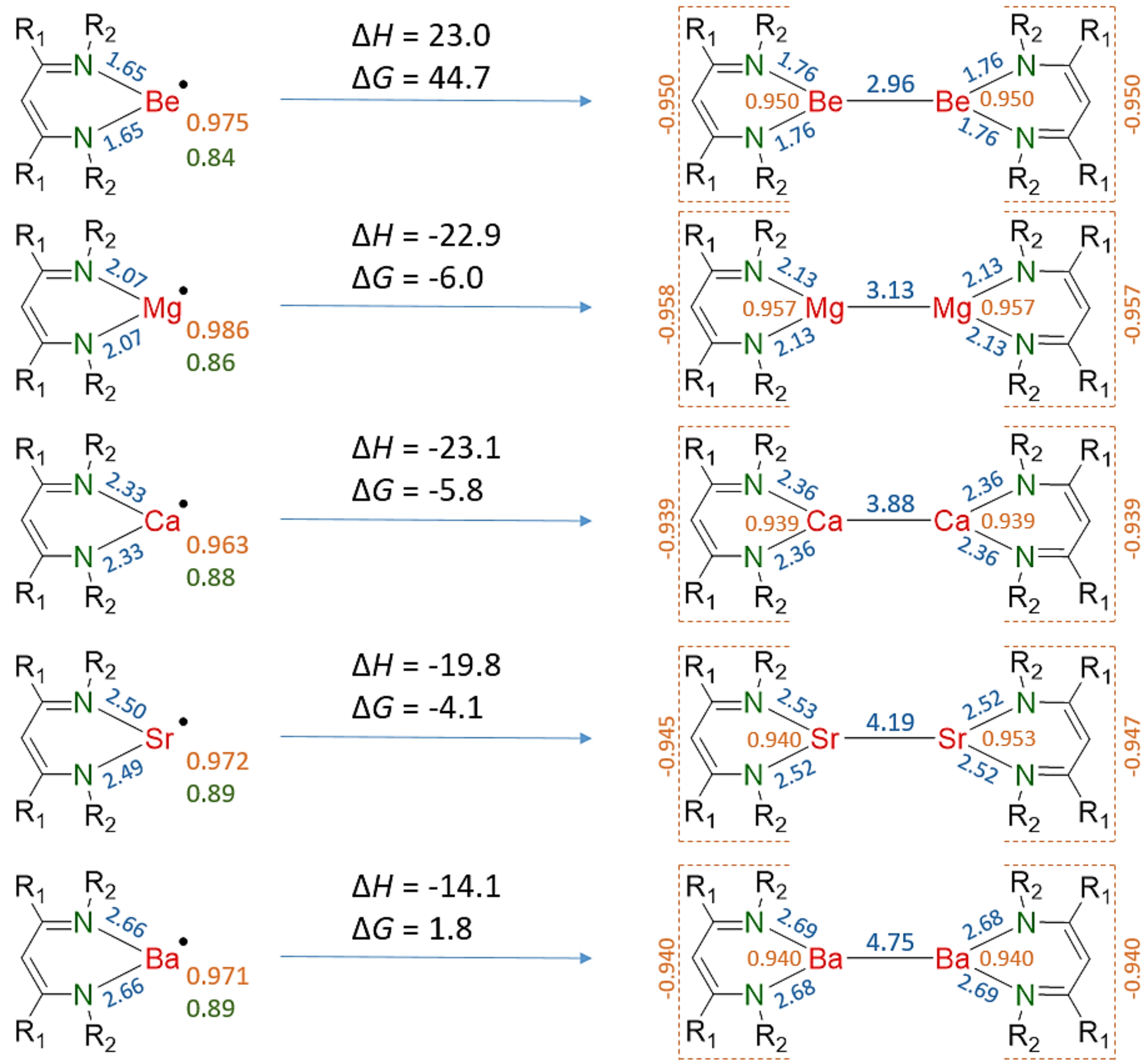

Figure S76: Reaction energies for the formation of (BDI)AeAe(BDI) from their corresponding (BDI)Ae radicals. Level of theory used B3PW91/def2TZVP//def2SVP **without Grimme's dispersion**. Energy is given in kcal/mol; Bond length in Å (blue color); NPA charger are given as number of electrons (orange color); spin density is in green color, dotted orange brackets shows collective partial NPA charger on ligand. (R_1= Me, R_2=DIPP)

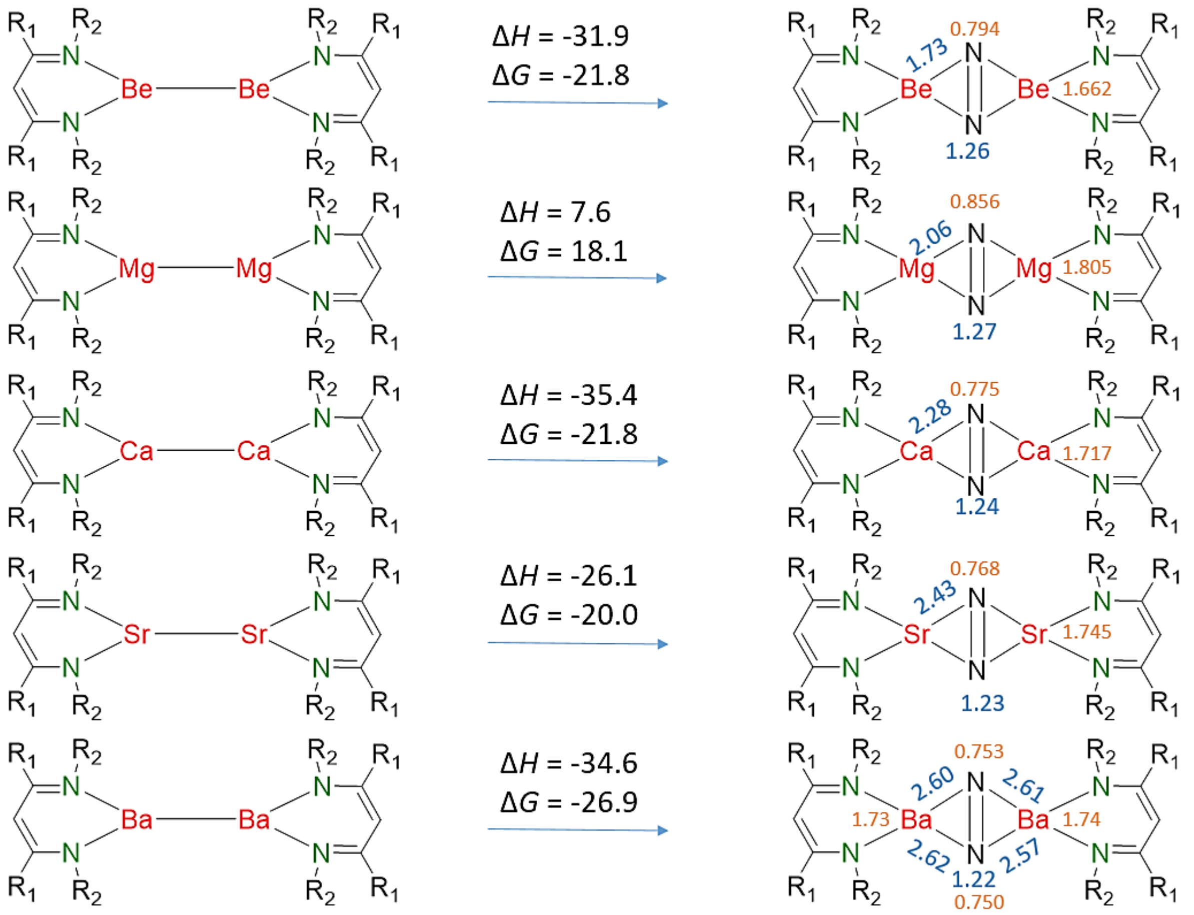

Figure S77: Reaction energies for the formation of N_2 complexes (**side-on** complexation) from (BDI)AeAe(BDI) and N_2. Level of theory used B3PW91/def2TZVP//def2SVP **with Grimme's dispersion**. Energy is given in kcal/mol; Bond length in Å (blue color); NPA charger are given as number of electrons (orange color). (R_1= Me, R_2=DIPP)

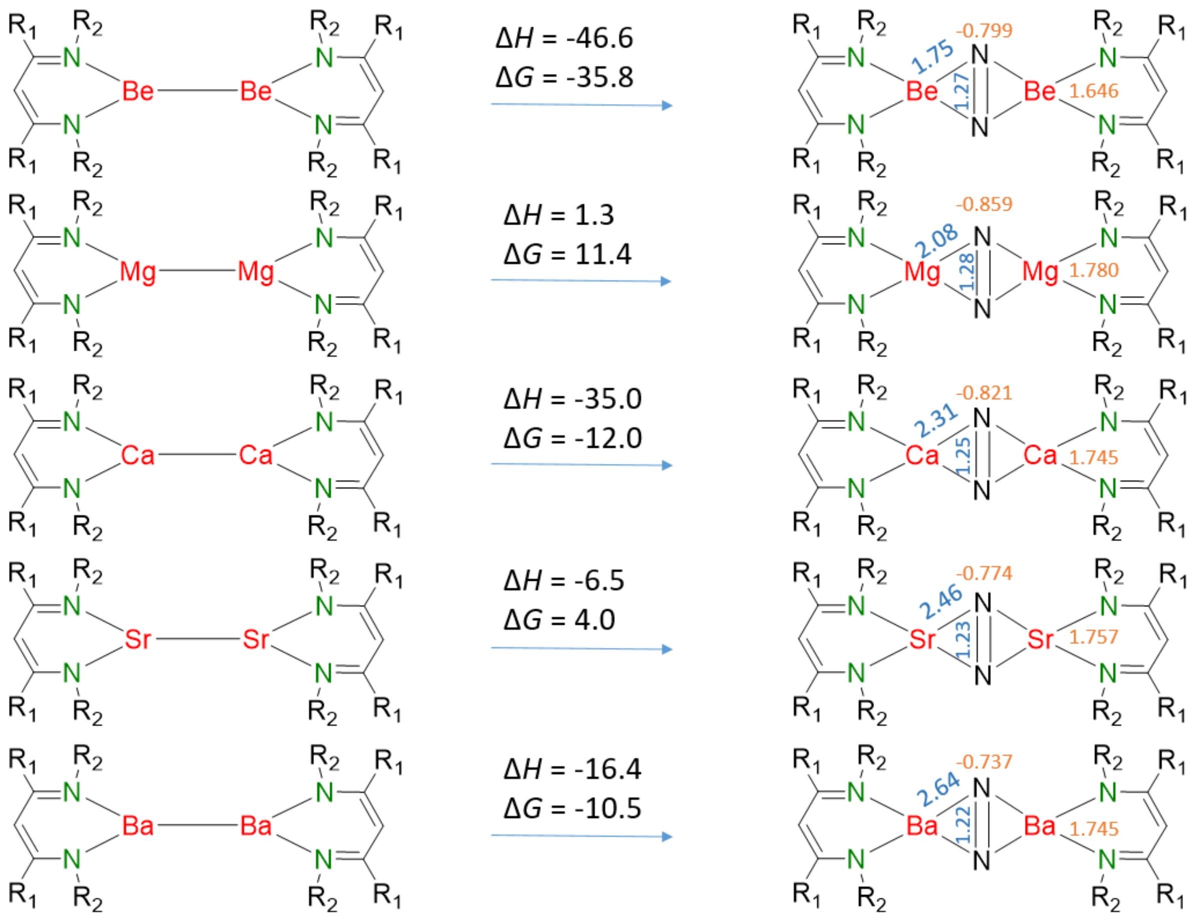

Figure S78: Reaction energies for the formation of N_2 complexes (**side-on** complexation) from (BDI)AeAe(BDI) and N_2. Level of theory used B3PW91/def2TZVP//def2SVP **without Grimme's dispersion**. Energy is given in kcal/mol; Bond length in Å (blue color); NPA charger are given as number of electrons (orange color). (R_1= Me, R_2=DIPP)

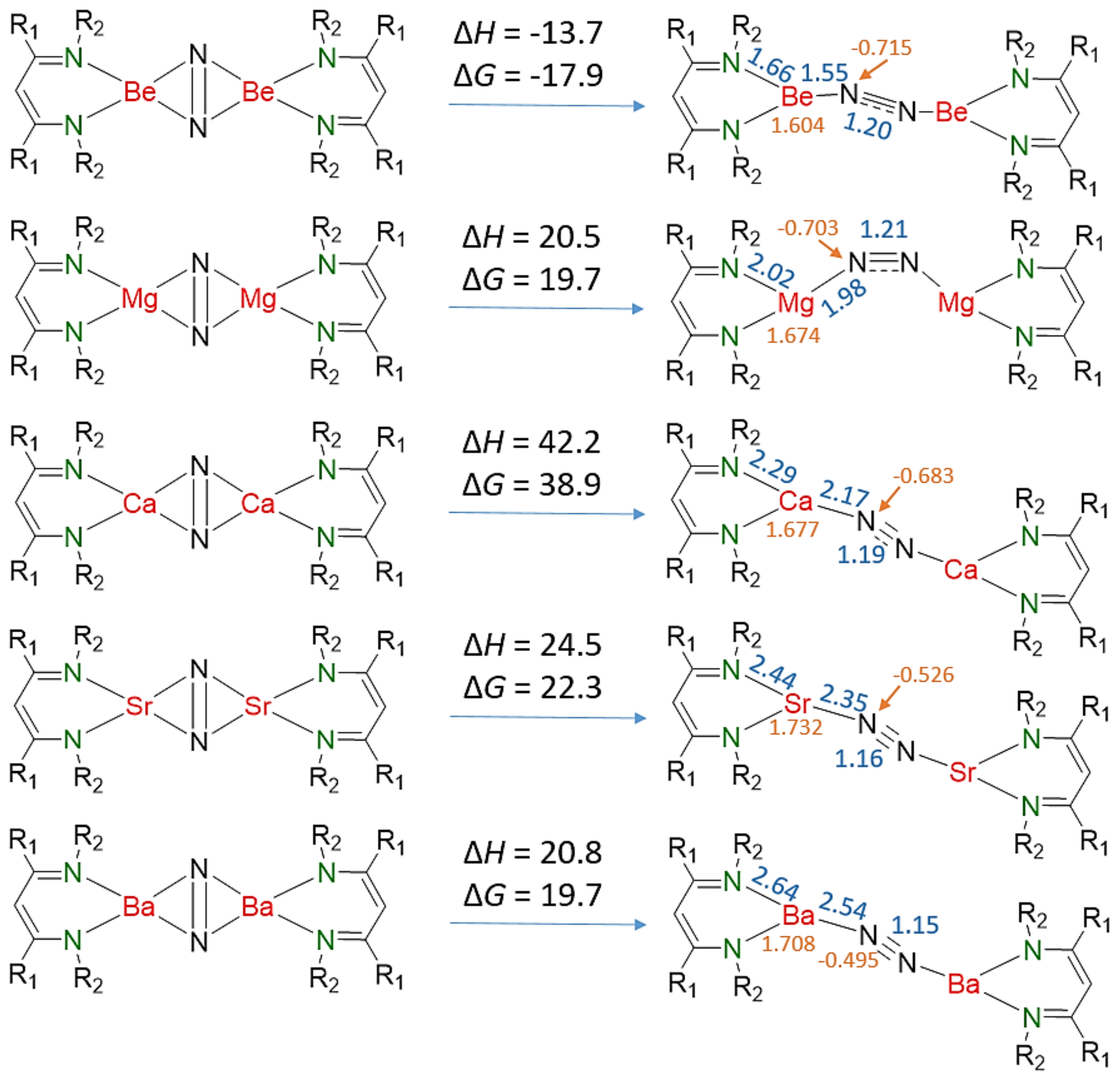

Figure S79: Energy required for the formation of N_2 complexes (**end-on** complexation) from (BDI)AeAe(BDI) and N_2. Level of theory used B3PW91/def2TZVP//def2SVP **with Grimme's dispersion**. Energy is given in kcal/mol; Bond length in Å (blue color); NPA charger are given as number of electrons (orange color). (R_1= Me, R_2=DIPP)

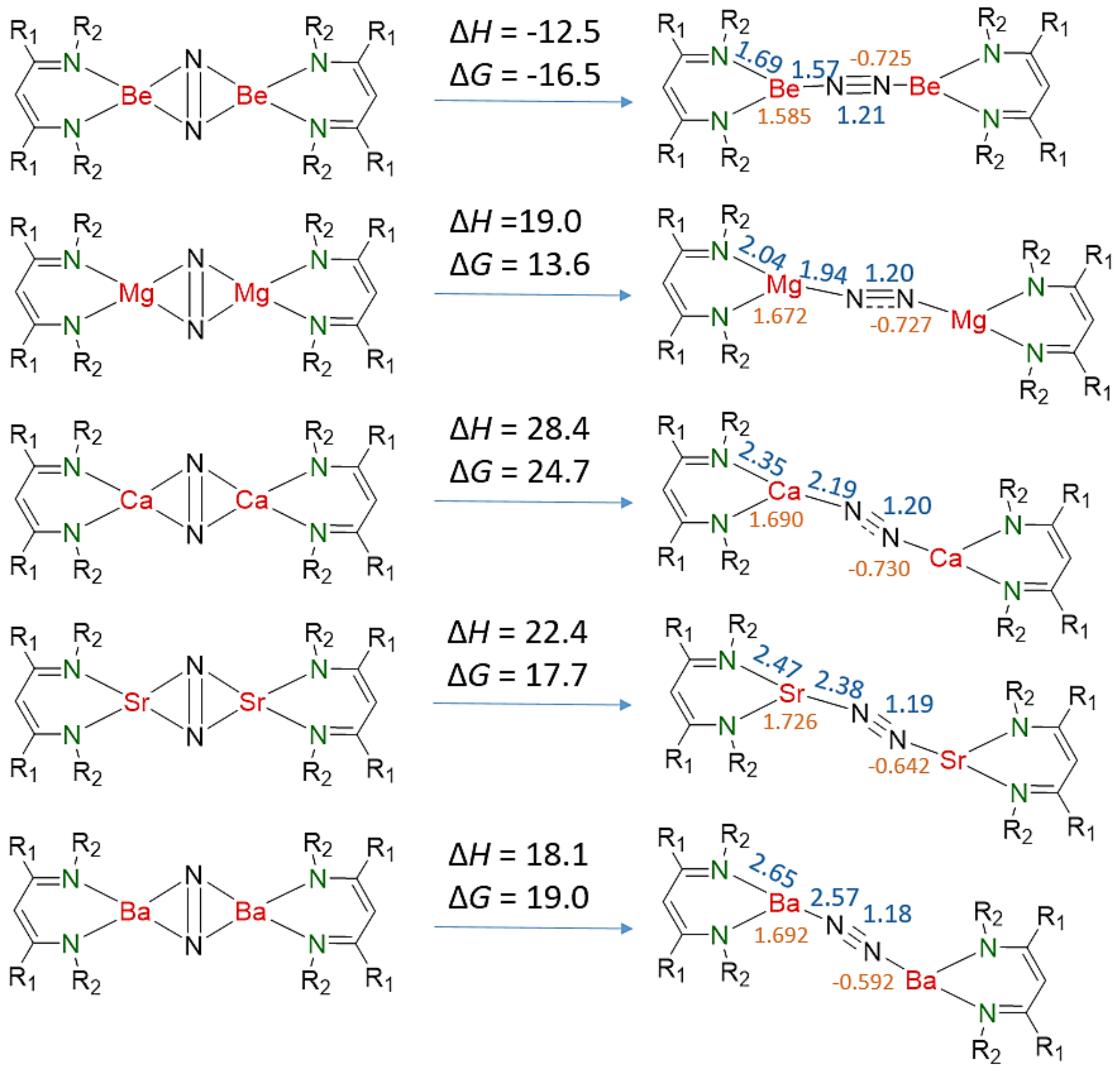

Figure S80: Energy required for the formation of N_2 complexes (**end-on** complexation) from (BDI)AeAe(BDI) and N_2. Level of theory used B3PW91/def2TZVP//def2SVP **without Grimme's dispersion**. Energy is given in kcal/mol; Bond length in Å (blue color); NPA charger are given as number of electrons (orange color). (R_1= Me, R_2=DIPP)

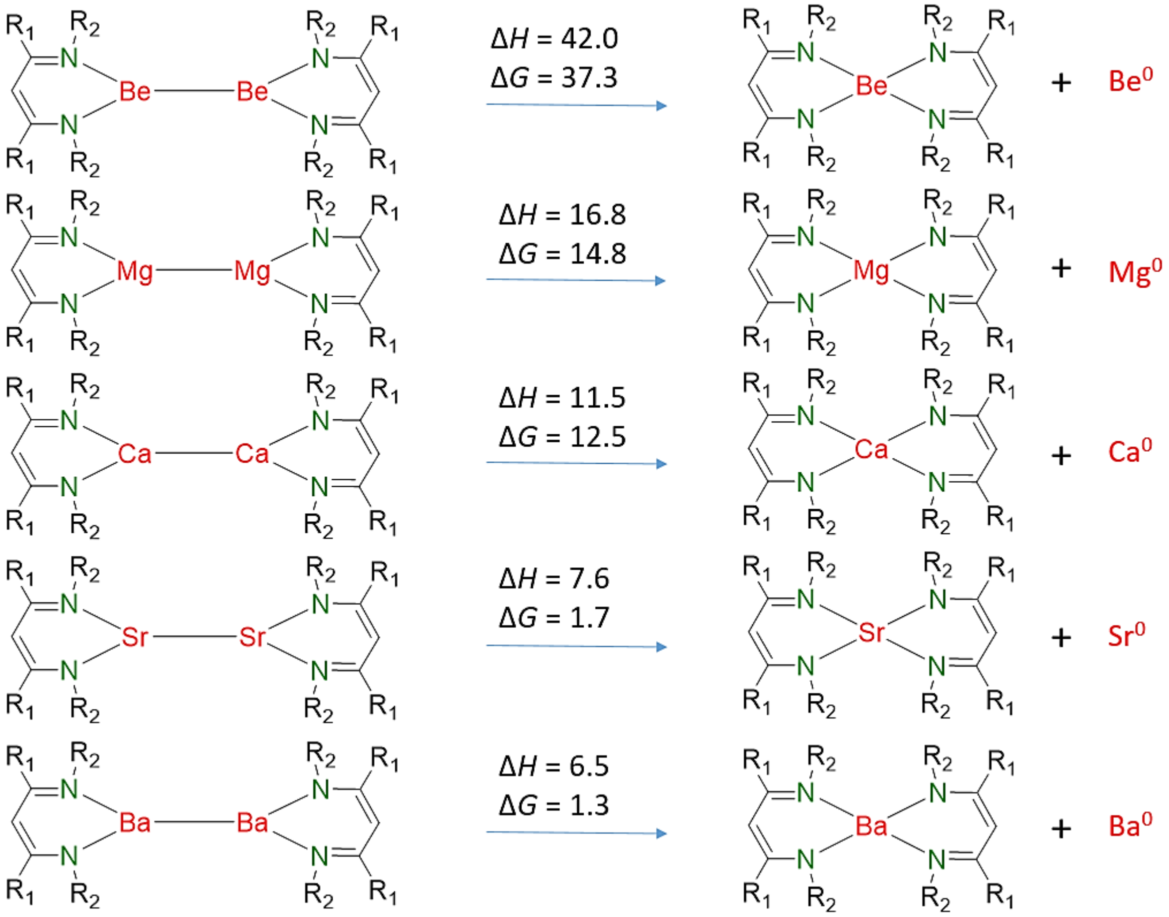

Figure S81: Reaction energies for the disproportionation of (BDI)AeAe(BDI) to give $(BDI)_2Ae$ and Ae^0. Level of theory used B3PW91/def2TZVP//def2SVP **with Grimme's dispersion**. Energy is given in kcal/mol. (R_1= Me, R_2=DIPP)

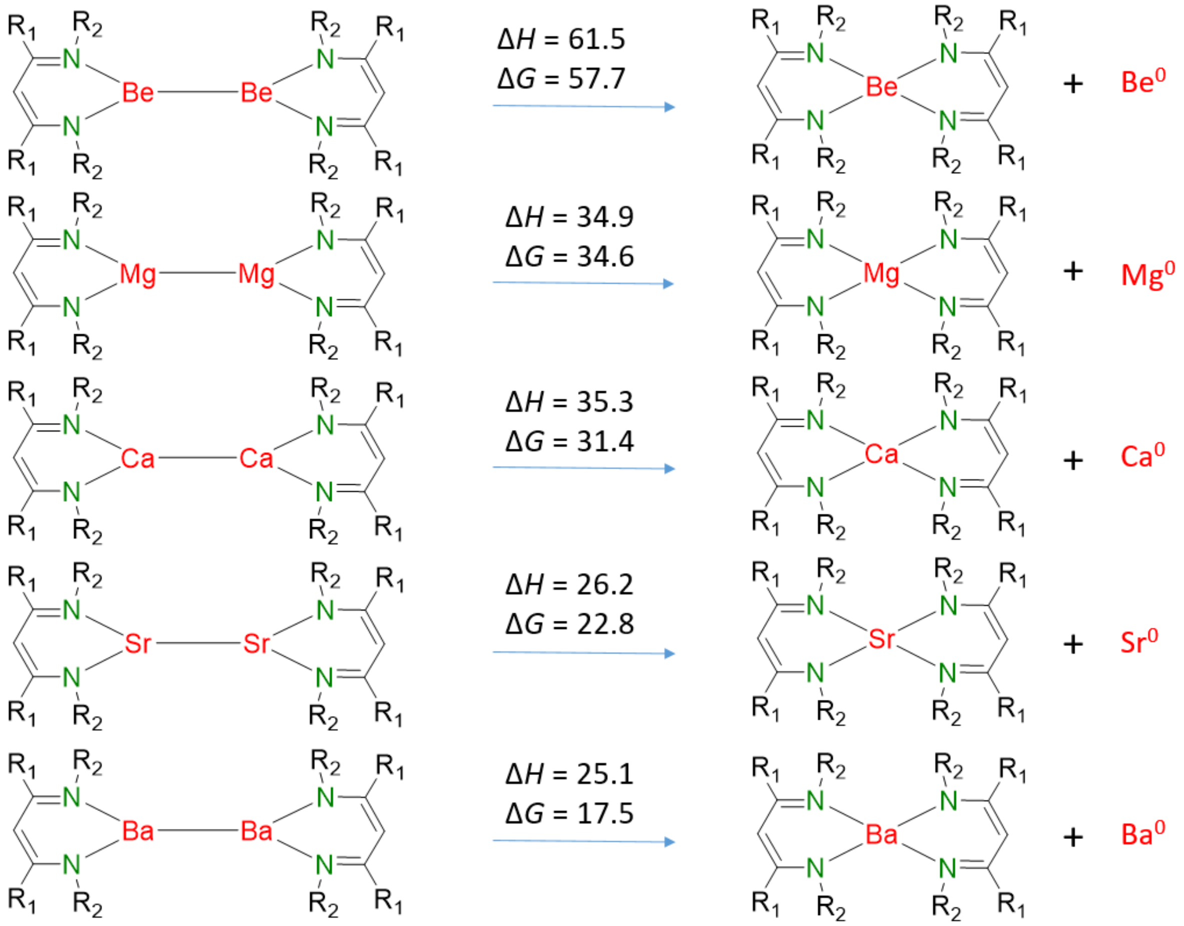

Figure S82: Reaction energies for the disproportionation of (BDI)AeAe(BDI) to give $(BDI)_2Ae$ and Ae^0. Level of theory used B3PW91/def2TZVP//def2SVP **without Grimme's dispersion**. (R_1= Me, R_2=DIPP)

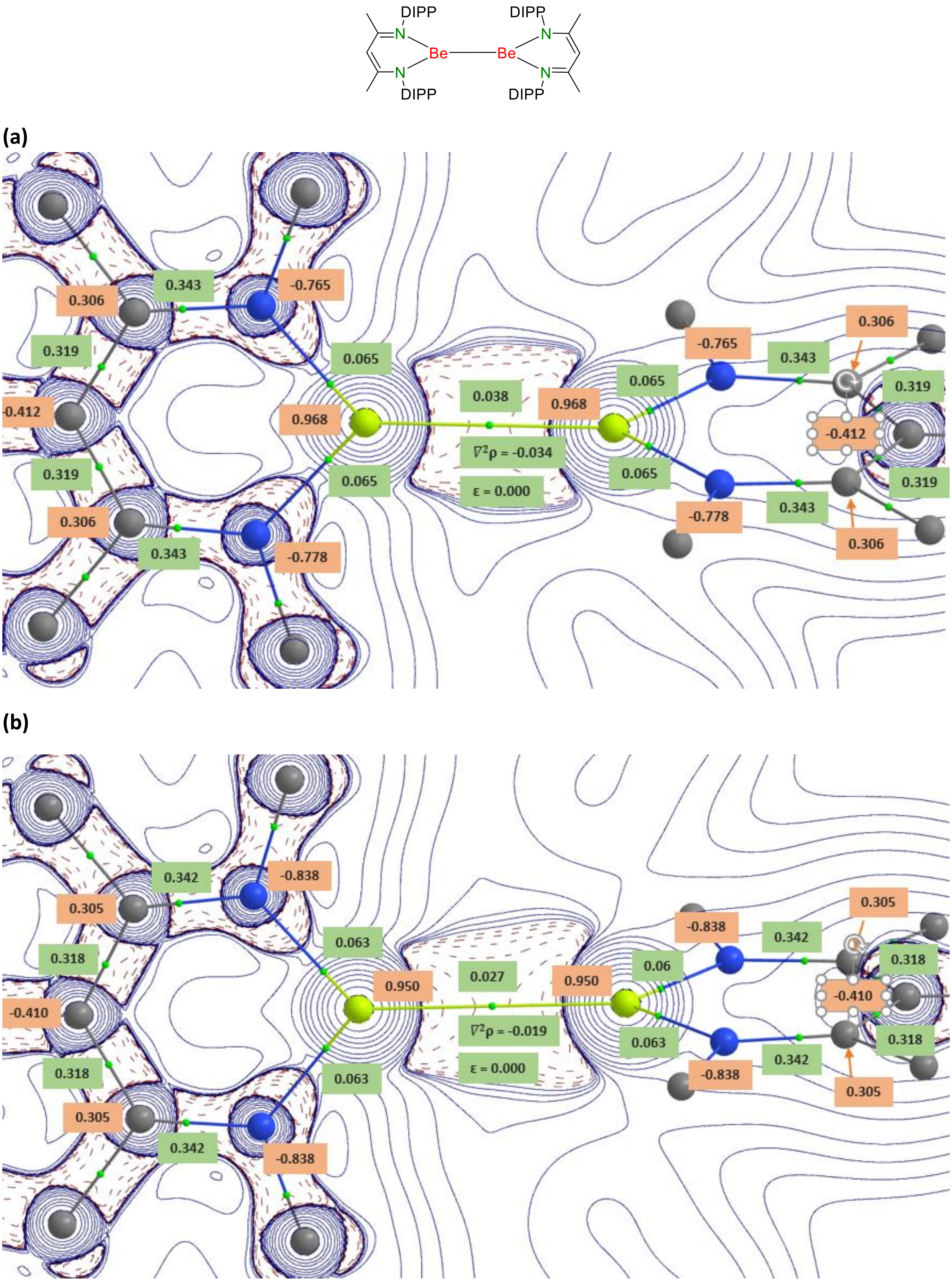

Figure S83: Atoms in Molecules (AIM) analysis of **(BDI)Be-Be(BDI)** (bond critical points are shown in fluorescent green). Level of theory used B3PW91/def2TZVP//def2SVP with (a) and without (b) Grimme's dispersion. NPA charges are shown in orange boxes; Electron density values ρ(**r**) at bond critical points (*bcp*'s) are shown in green boxes; ε shows bond elipticity; $\nabla^2\rho(\mathbf{r})$ is Laplacian of electron density.

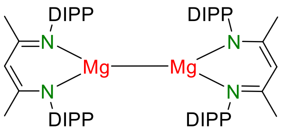

(a)

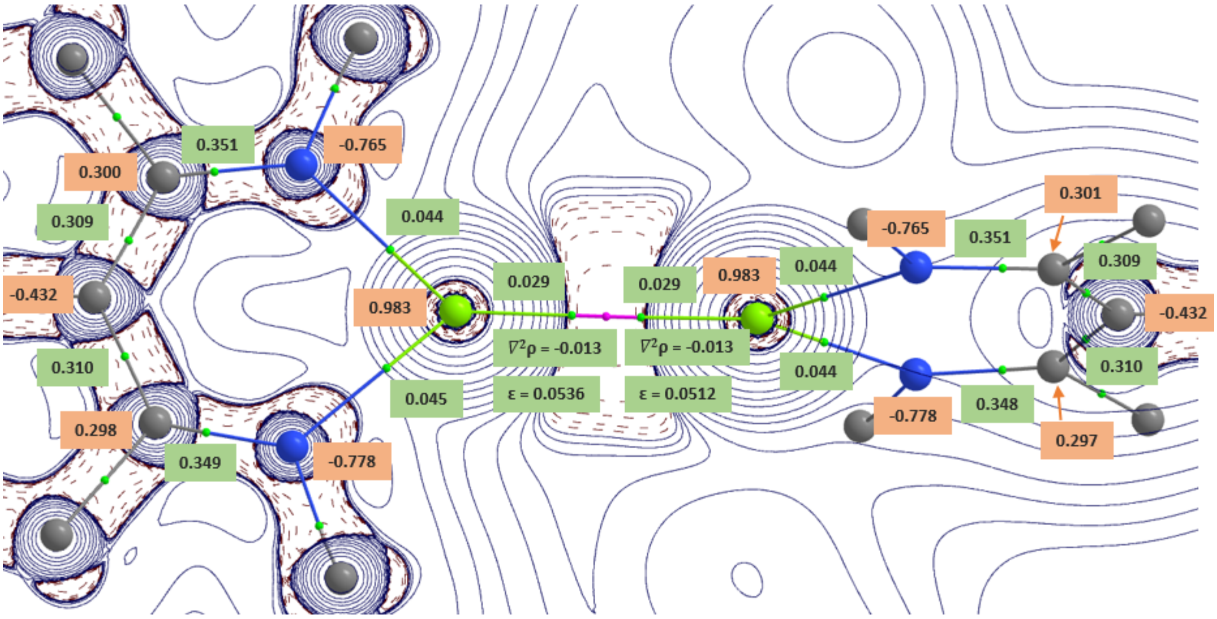

(b)

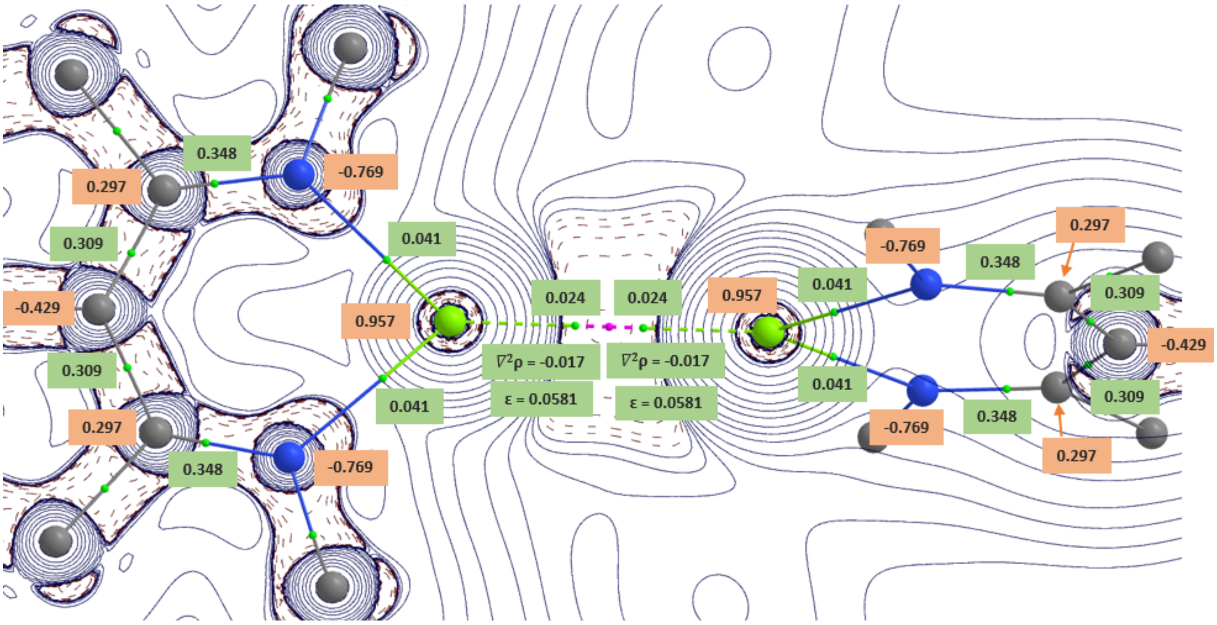

Figure S84: Atoms in Molecules (AIM) analysis of **(BDI)Mg-Mg(BDI)** (bond critical points are shown in fluorescent green). Level of theory used B3PW91/def2TZVP//def2SVP with (a) and without (b) Grimme's dispersion. NPA charges are shown in orange boxes; Electron density values ρ(**r**) at bond critical points (*bcp*'s) are shown in green boxes; ε shows bond elipticity; $\nabla^2\rho(\mathbf{r})$ is Laplacian of electron density. The total electron density in the non-nuclear attractor in (a) contributes to 0.801 *e*. The total electron density in the non-nuclear attractor in (b) contributes to 0.623 *e*.

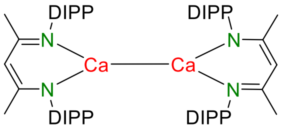

(a)

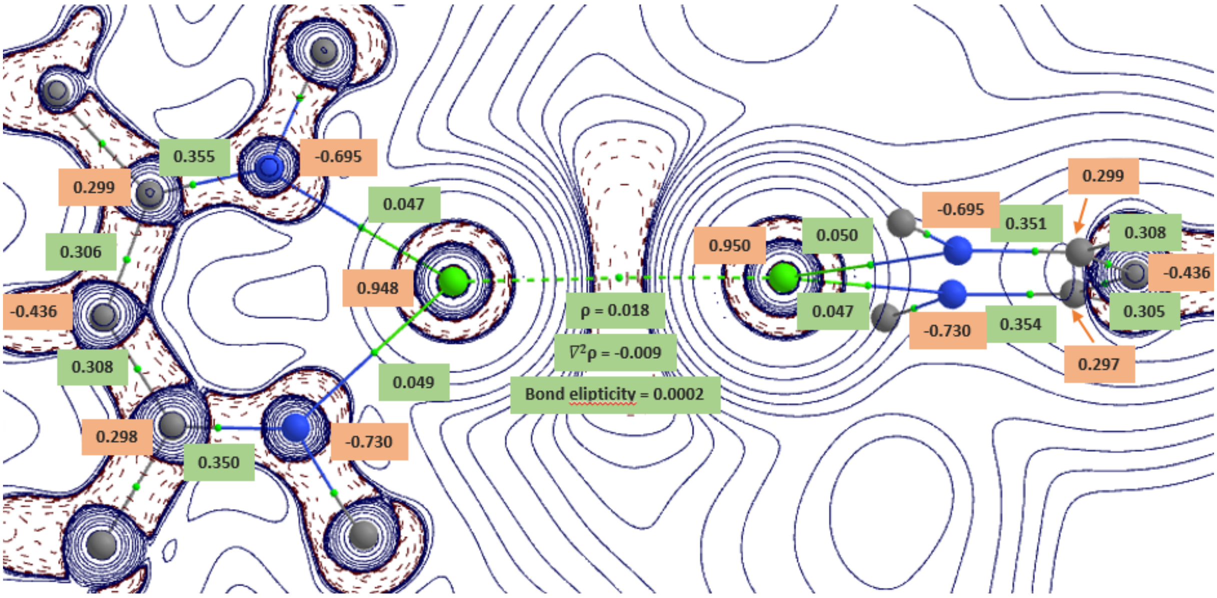

(b)

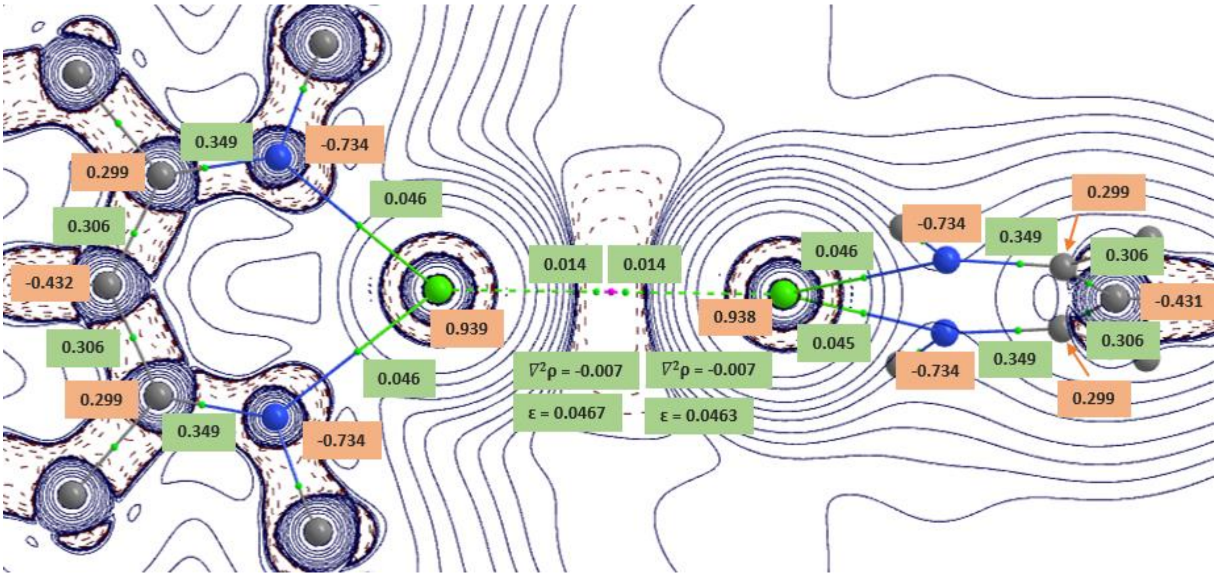

Figure S85: Atoms in Molecules (AIM) analysis of **(BDI)Ca-Ca(BDI)** (bond critical points are shown in fluorescent green). Level of theory used B3PW91/def2TZVP//def2SVP with (a) and without (b) Grimme's dispersion. NPA charges are shown in orange boxes; Electron density values ρ(**r**) at bond critical points (*bcp*'s) are shown in green boxes; ε shows bond elipticity; $\nabla^2\rho(\mathbf{r})$ is Laplacian of electron density. The total electron density in the non-nuclear attractor in (b) contributes to 0.333 *e*.

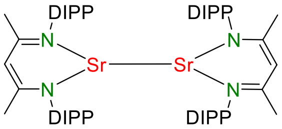

(a)

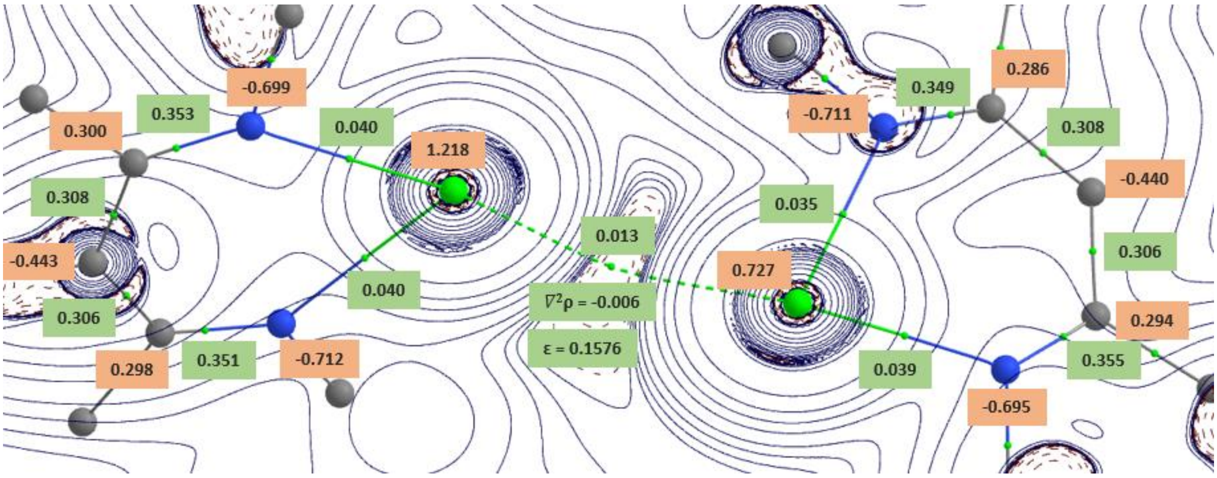

(b)

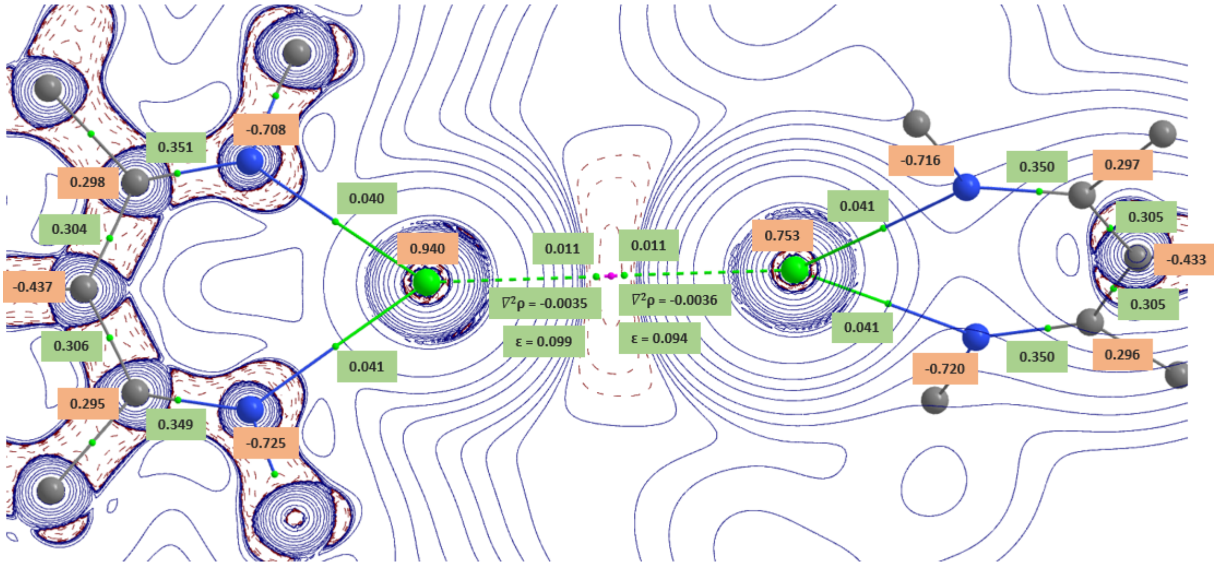

Figure S86: Atoms in Molecules (AIM) analysis of **(BDI)Sr-Sr(BDI)** (bond critical points are shown in fluorescent green). Level of theory used B3PW91/def2TZVP//def2SVP with (a) and without (b) Grimme's dispersion. NPA charges are shown in orange boxes; Electron density values ρ(**r**) at bond critical points (*bcp*'s) are shown in green boxes; ε shows bond elipticity; $\nabla^2\rho(\mathbf{r})$ is Laplacian of electron density. The total electron density in the non-nuclear attractor in (b) contributes to 0.350 *e*.

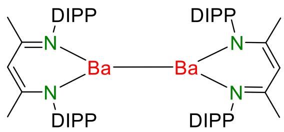

(a)

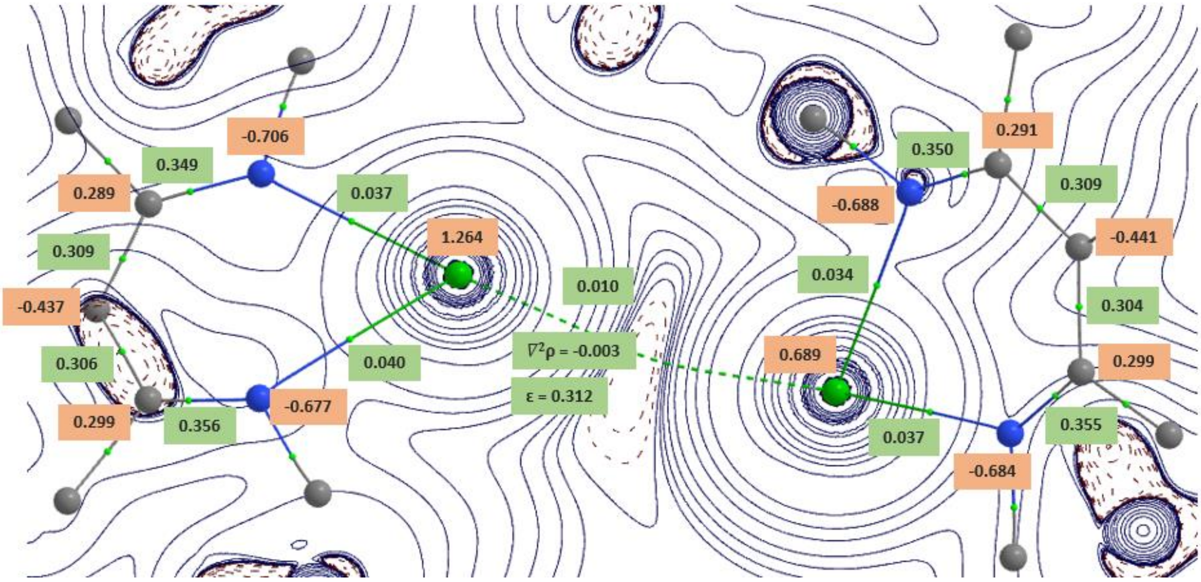

(b)

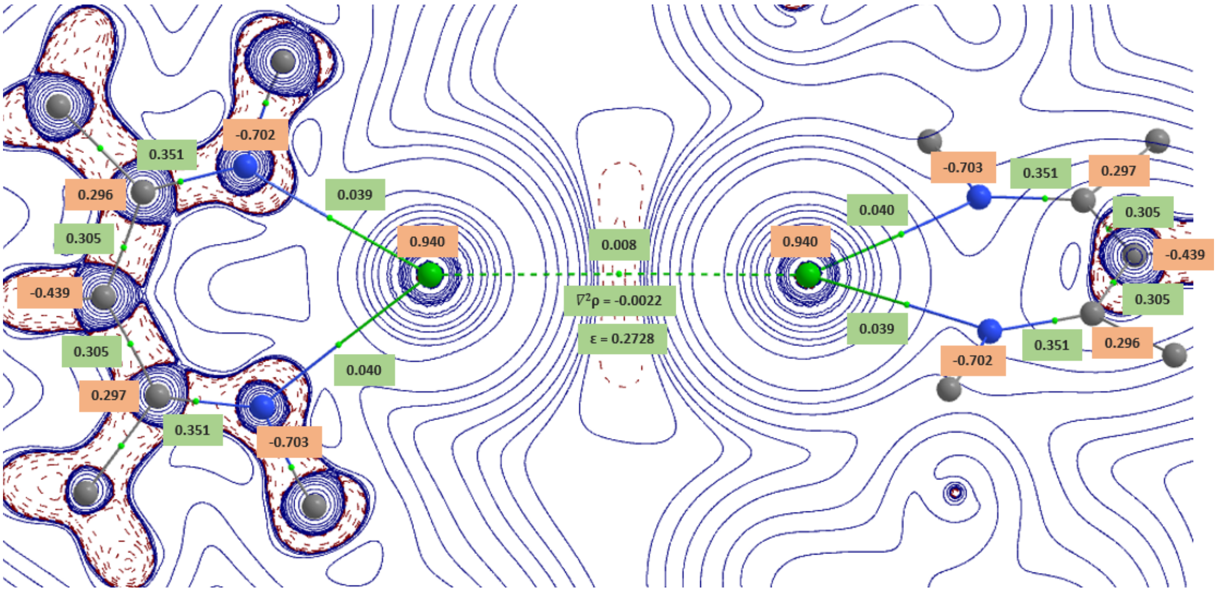

Figure S87: Atoms in Molecules (AIM) analysis of **(BDI)Ba-Ba(BDI)** (bond critical points are shown in fluorescent green). Level of theory used B3PW91/def2TZVP//def2SVP with (a) and without (b) Grimme's dispersion. NPA charges are shown in orange boxes; Electron density values ρ(**r**) at bond critical points (*bcp*'s) are shown in green boxes; ε shows bond elipticity; $\nabla^2\rho(\mathbf{r})$ is Laplacian of electron density.

Calculations on [K(^{DIPeP}NN)Ae]$_2$(N$_2$) complexes (6-Ae)

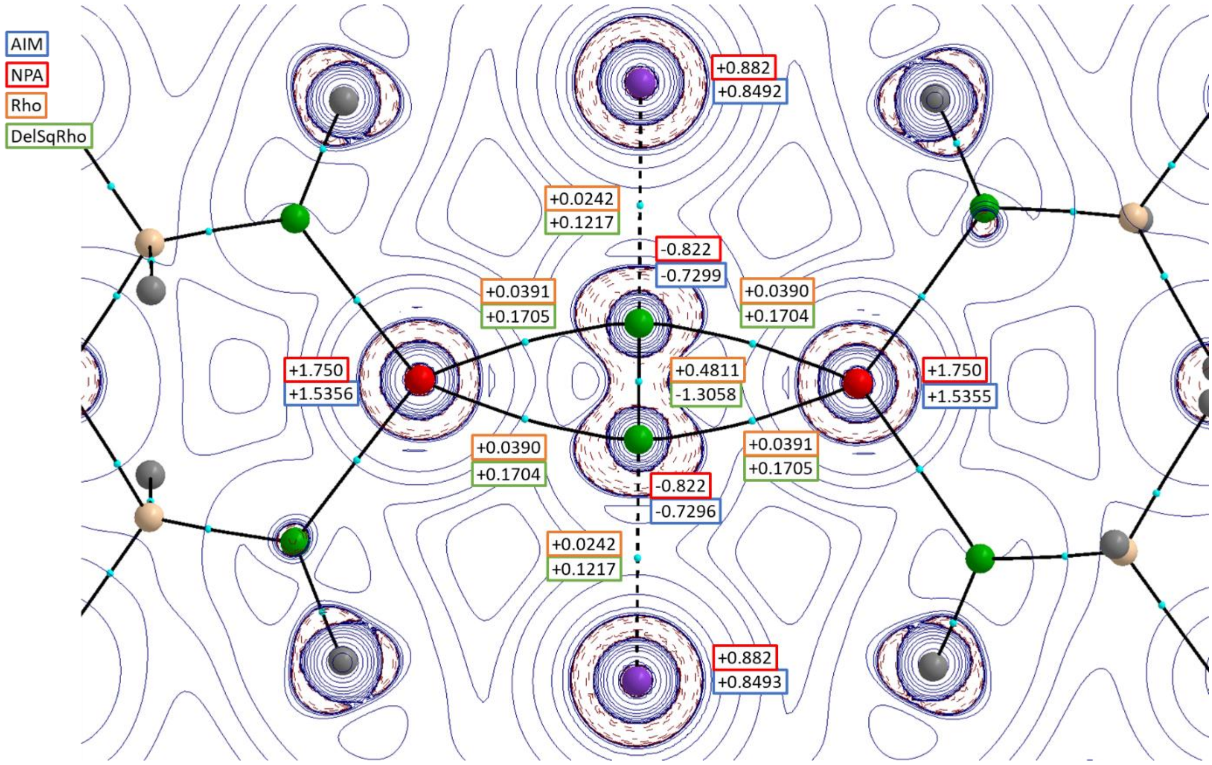

Figure S88: Laplacian distribution for complex **6**-Ca in the K-Ca-N$_2$-Ca-K plane at the B3PW91/def2TZVP+GD3BJ//B3PW91/def2SVP+GD3BJ level. Red dashed lines indicate areas of charge concentration, while blue solid lines show areas of charge depletion. Light-blue dots indicate bond critical points (*bcp*'s). Shown are the AIM charges in blue boxes, NPA charges in red boxes, the electron density ρ(**r**) in orange boxes and the Laplacian $\nabla^2\rho(\mathbf{r})$ in green boxes.

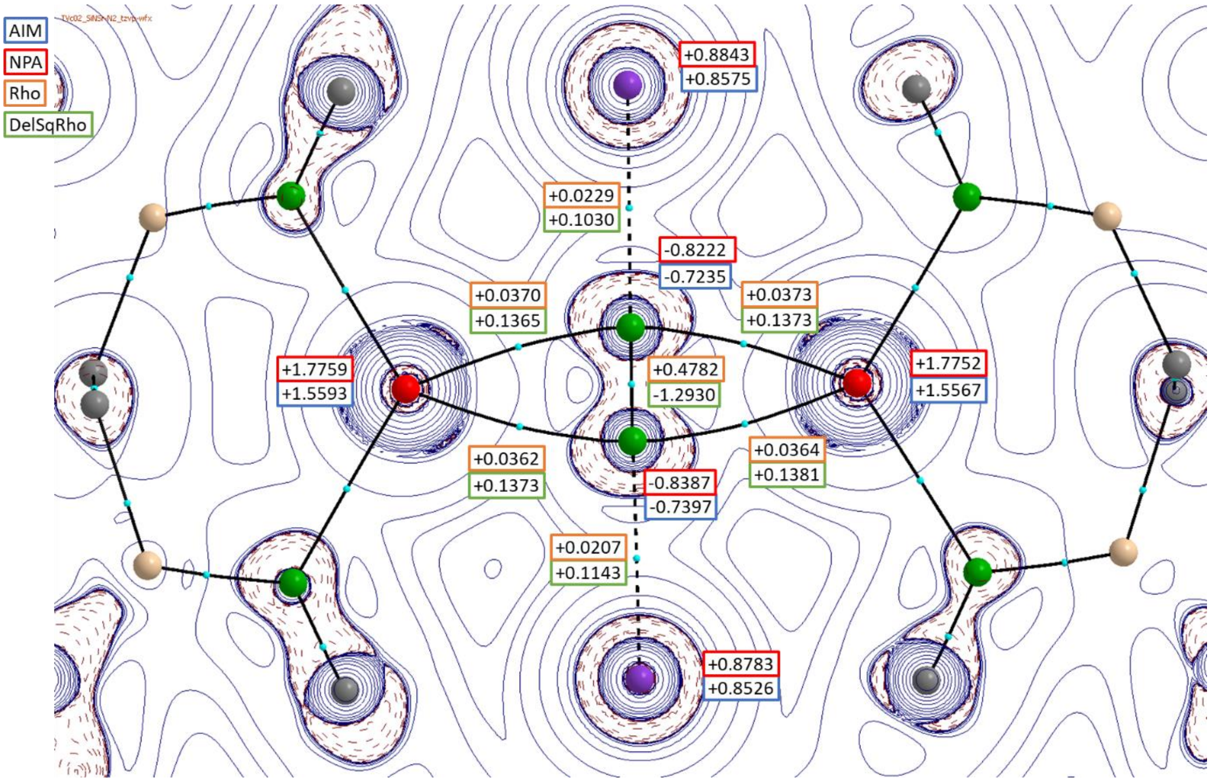

Figure S89: Laplacian distribution for complex **6**-Sr in the Sr-N_2-Sr plane at the B3PW91/def2TZVP+GD3BJ//B3PW91/def2SVP+GD3BJ level. Red dashed lines indicate areas of charge concentration, while blue solid lines show areas of charge depletion. Light-blue dots indicate bond critical points (*bcp*'s). Shown are the AIM charges in blue boxes, NPA charges in red boxes, the electron density ρ(**r**) in orange boxes and the Laplacian $\nabla^2\rho(\mathbf{r})$ in green boxes.

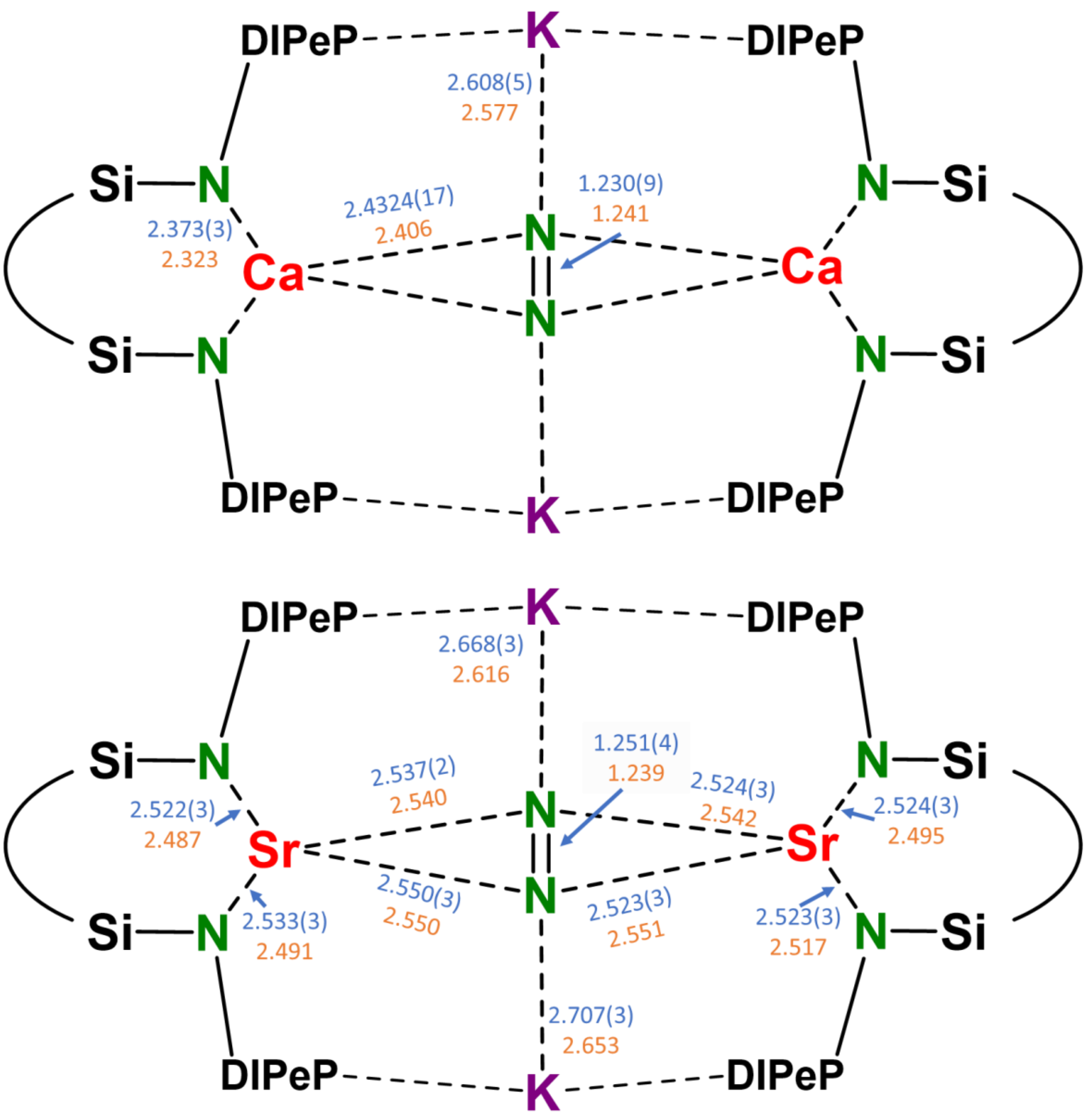

Figure S90: Comparison of selected experimentally determined distances (X-ray, blue) with calculated values (orange) for complexes **6**-Ca and **6**-Sr.

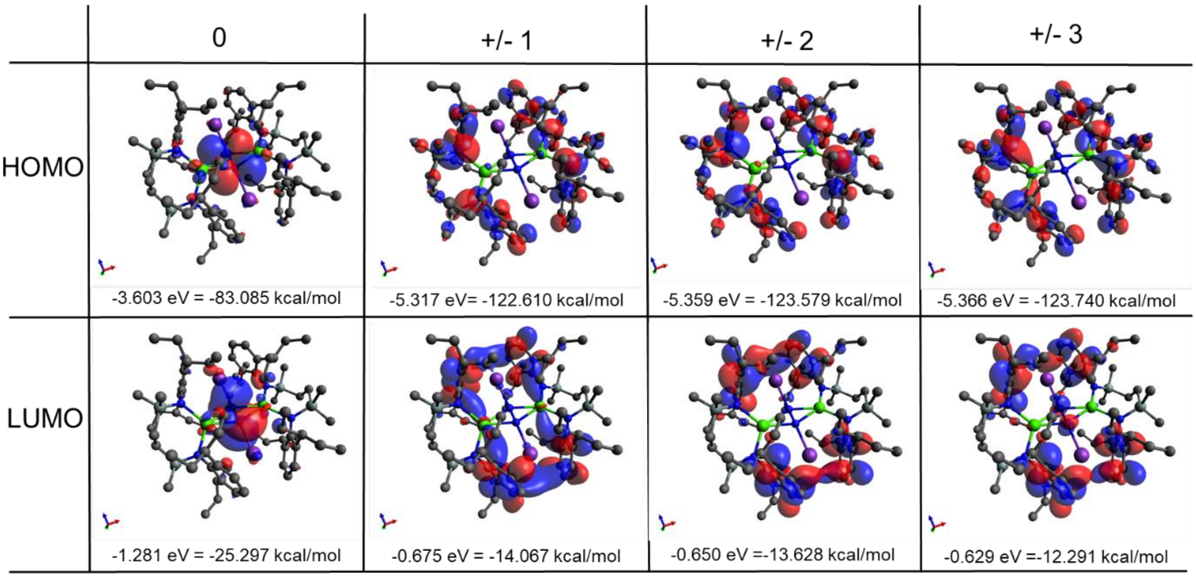

Figure S91: Selected MO´s for **6**-Ca, computed at the B3PW91-GD3BJ/def2tzvp //B3PW91-GD3BJ/def2svp level of theory.

HOMO orbital contribution (6-Ca):

N1 p=0.3905, N2 p=0.3905, Ca2 d=0.0397, Ca1 d=0.0397, K1 d=0.0148, K2 d=0.0148

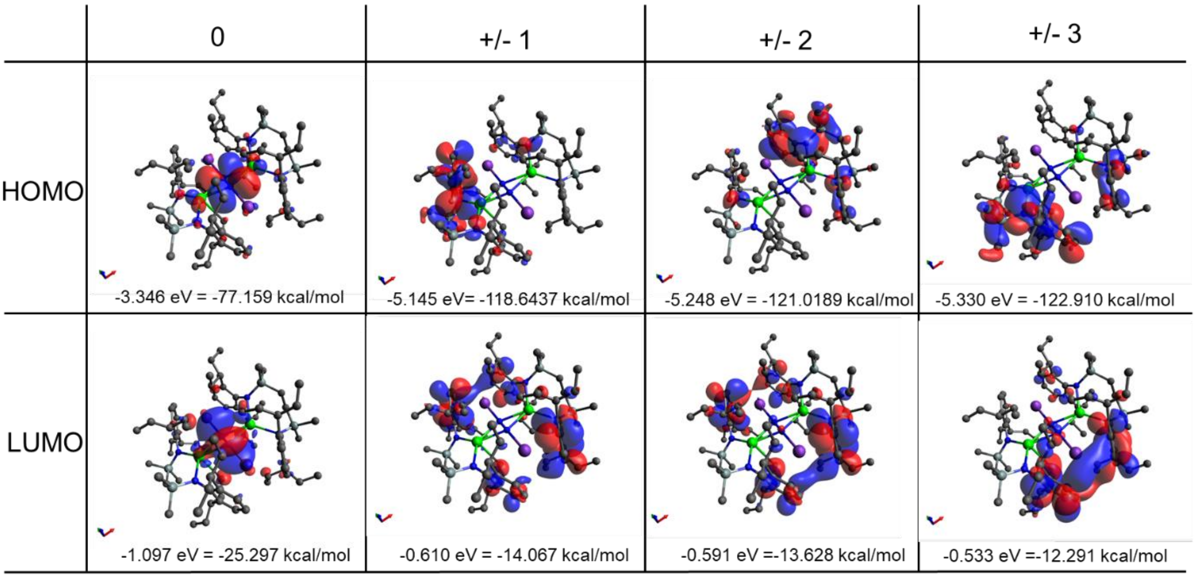

Figure S92: Selected MO´s for **6**-Sr, computed at the B3PW91-GD3BJ/def2tzvp //B3PW91-GD3BJ/def2svp level of theory.

HOMO orbital contribution (6-Sr):

N1 p=0.3937, N2 p=0.3856, Sr1 d=0.0546, Sr2 d=0.0522, Sr2 p=0.0104

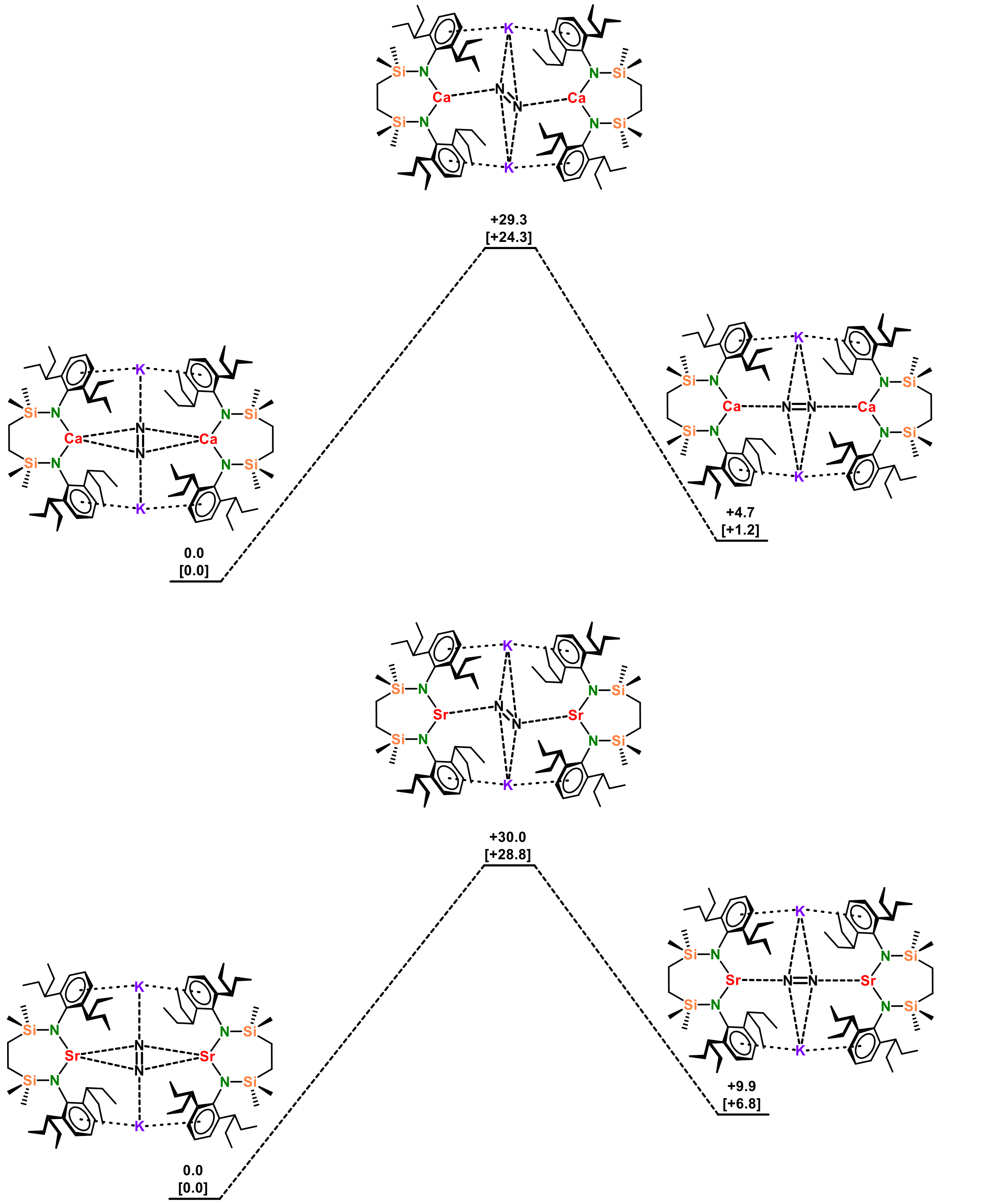

Figure S93: Energy profiles (ΔH[ΔG] in kcal/mol) for switching the N_2 binding modes between end-on and side-on in the complexes **6**-Ca and **6**-Sr.

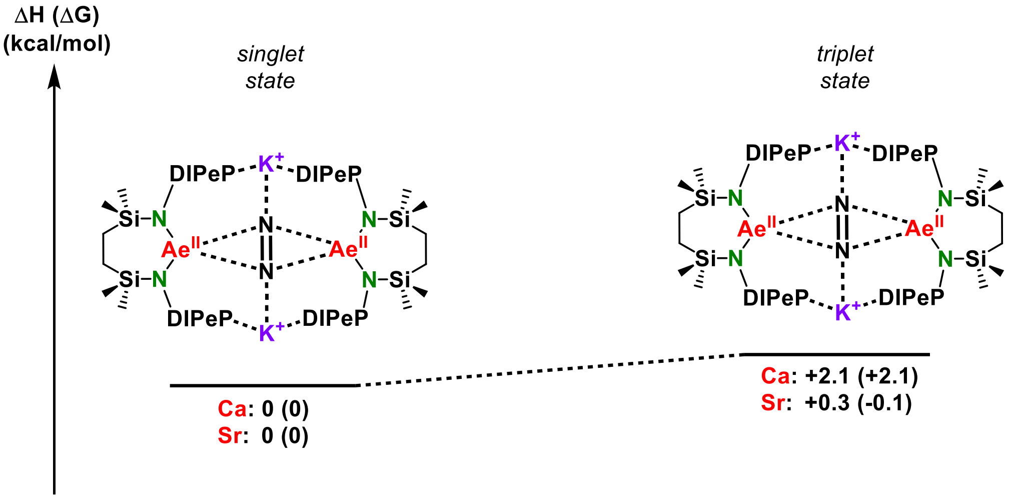

Figure S94: Energy profiles (ΔH[ΔG] in kcal/mol) for the singlet and triplet states of **6**-Ae. The triplet states were computed at the UB3PW91-GD3BJ/def2svp // UB3PW91-GD3BJ/def2tzvp level of theory.

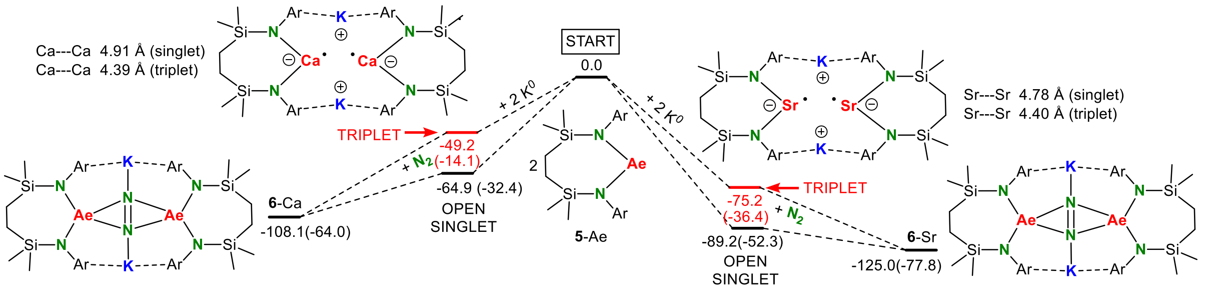

Figure S95: Computed energy profiles (ΔH(ΔG) in kcal/mol) for the formation of **6**-Ae. **5**-Ae and **6**-Ae were computed at the B3PW91-GD3BJ/def2tzvp//B3PW91-GD3BJ/def2svp level of theory. The triplet and unrestricted singlet states of the intermediate diradicaloids were computed at the UB3PW91-GD3BJ/def2svp// UB3PW91-GD3BJ/def2tzvp level of theory.

XYZ-coordinates

(BDI)Ae-Ae(BDI) Dimeric Complexes

146

(BDI)BeBe(BDI) with dispersion

Be	0.001075	0.001300	1.312198
Be	-0.001356	-0.001190	-1.311923
N	0.442834	1.265100	2.436870
N	-1.266003	0.438144	-2.436736
N	-0.437701	-1.260854	2.439606
N	1.260247	-0.442024	-2.438987
C	0.772159	2.331783	4.658795
C	-2.333729	0.762551	-4.659116
H	1.753425	2.752589	4.402864
H	-2.757601	1.742482	-4.403253
H	0.780045	2.020431	5.710239
H	-2.021843	0.771303	-5.710395
H	0.050472	3.153198	4.546095
H	-3.153094	0.038399	-4.546979
C	0.400473	1.166450	3.774204
C	-1.167896	0.393631	-3.774025
C	0.003755	0.003854	4.428506
C	-0.005320	-0.003562	-4.428100
H	0.004373	0.004751	5.516392
H	-0.006679	-0.004643	-5.515982
C	-0.393506	-1.159989	3.776608
C	1.158838	-0.399536	-3.776106
C	-0.762601	-2.324126	4.663870
C	2.322706	-0.770106	-4.663100
H	-0.037185	-3.142813	4.554633
H	3.143398	-0.047261	-4.552284
H	-1.741633	-2.749751	4.407549
H	2.745453	-1.750689	-4.407785
H	-0.773027	-2.009848	5.714420
H	2.009150	-0.778455	-5.713864
C	0.950995	2.526845	1.998465
C	-2.527822	0.946568	-1.998865
C	2.351687	2.682587	1.896680
C	-2.684173	2.347344	-1.899787
C	2.842660	3.937403	1.523761
C	-3.939149	2.838443	-1.527554
H	3.917806	4.091660	1.426086
H	-4.093792	3.913704	-1.431726
C	1.980906	5.006781	1.290446
C	-5.008192	1.976703	-1.292565
H	2.384901	5.975208	0.985349
H	-5.976726	2.380827	-0.987987
C	0.614463	4.859253	1.506161
C	-4.860127	0.609919	-1.505858
H	-0.043297	5.723968	1.396264
H	-5.724557	-0.047991	-1.394641
C	0.078399	3.627180	1.896886
C	-3.627854	0.073751	-1.895785
C	3.265213	1.555630	2.366488
C	-1.557887	3.260619	-2.371681
H	2.850128	1.250138	3.341318
H	-1.252260	2.843743	-3.345713
C	4.698226	2.005770	2.636290
C	-2.009229	4.692677	-2.644372
H	5.274328	1.174896	3.071493
H	-1.178751	5.268634	-3.080520
H	5.213263	2.303135	1.708660
H	-2.307129	5.209104	-1.717701
C	3.265855	0.291701	1.500342
C	-0.293739	3.264358	-1.505865
H	4.020468	0.344457	0.707956
H	-0.347169	4.020431	-0.714904
H	3.496475	-0.590180	2.114580
H	0.587694	3.495022	-2.120701
C	-1.381714	3.497228	2.299969
C	-3.497165	-1.386970	-2.296259
H	-1.447630	2.639344	2.985161
H	-2.639294	-1.453531	-2.981406
C	-1.895113	4.724685	3.052653
C	-4.724312	-1.902737	-3.047854
H	-2.898517	4.526423	3.460270
H	-4.525108	-2.906551	-3.453993
H	-1.234892	5.002787	3.889102
H	-5.003303	-1.244052	-3.885232
C	-2.279973	3.167383	1.118527
C	-3.166501	-2.282531	-1.113029
H	-2.298070	3.997454	0.399492
H	-3.996473	-2.299639	-0.393929
H	-1.930273	2.271618	0.589849
H	-2.271032	-1.930860	-0.585187
C	-0.945762	-2.523542	2.003879
C	2.522971	-0.949989	-2.003315
C	-0.072650	-3.623467	1.902970
C	3.623242	-0.077196	-1.903930
C	-0.608498	-4.856765	1.515928
C	4.856400	-0.613109	-1.516474
H	0.049659	-5.721279	1.406810
H	5.721244	0.044800	-1.408513
C	-1.975233	-5.005595	1.302875
C	5.004691	-1.979581	-1.301545
H	-2.379125	-5.974884	1.000408
H	5.973911	-2.383459	-0.998839
C	-2.837260	-3.936234	1.535338
C	3.934911	-2.841518	-1.532469
H	-3.912442	-4.091417	1.439569
H	4.089637	-3.916639	-1.435291
C	-2.346521	-2.680319	1.904956
C	2.679082	-2.350709	-1.902263
C	1.388054	-3.491323	2.302866
C	3.491707	1.383052	-2.305787
H	1.454621	-2.631738	2.985861
H	2.631766	1.449187	-2.988396
C	2.283117	-3.163518	1.118430
C	3.164716	2.280101	-1.122591
H	2.299550	-3.994766	0.400813
H	3.996816	2.298126	-0.405943
H	1.931551	-2.269022	0.588874
H	2.270724	1.929247	-0.591565
C	1.904081	-4.716647	3.057185
C	4.716835	1.897357	-3.061598
H	2.908245	-4.516754	3.462137
H	4.517034	2.900842	-3.468268
H	1.245895	-4.993368	3.895709
H	4.992969	1.237576	-3.899077
C	-3.259827	-1.553227	2.374939
C	1.551179	-3.264049	-2.370246

H	-2.843237	-1.246806	3.348857
H	1.243390	-2.848192	-3.344022
C	-3.262353	-0.289861	1.507961
C	0.288922	-3.266339	-1.501726
H	-4.019603	-0.342348	0.718069
H	0.343113	-4.022149	-0.710501
H	-3.490254	0.592522	2.122423
H	-0.594025	-3.496297	-2.114626
C	-4.692230	-2.003565	2.647556
C	2.001240	-4.696641	-2.642445
H	-5.268188	-1.172143	3.081912
H	1.169554	-5.272677	-3.076180
H	-5.208266	-2.302969	1.721149
H	2.300944	-5.212350	-1.715949
H	-4.736273	-2.850446	3.349569
H	2.847844	-4.741141	-3.344775
H	-2.300944	-0.112131	1.000976
H	0.110820	-2.304063	-0.996512
H	-1.983118	5.602551	2.393266
H	-5.601928	-1.990737	-2.388139
H	-3.316066	2.985041	1.440885
H	-2.983330	-3.319184	-1.433110
H	1.991304	-5.595968	2.399603
H	5.596483	1.985533	-2.404603
H	3.319985	-2.979979	1.437591
H	2.980751	3.316338	-1.443554
H	4.743569	2.854008	3.336589
H	-2.857395	4.736055	-3.344889
H	2.302948	0.112826	0.996581
H	-0.113592	2.302671	-1.000220

146

(BDI)BeBe(BDI) withOut dispersion

Be	0.030137	-0.000332	1.480914
Be	-0.029635	0.001132	-1.480985
N	-1.301838	0.086323	2.631212
N	-0.140940	-1.352648	-2.603305
N	1.406987	-0.087802	2.577285
N	0.033845	1.356546	-2.605334
C	-2.347023	0.109848	4.893678
C	-0.213853	-2.441563	-4.843329
H	-3.018735	0.950555	4.673998
H	-1.048658	-3.109230	-4.591159
H	-2.014058	0.186698	5.936228
H	-0.314780	-2.129379	-5.890234
H	-2.954123	-0.801176	4.793215
H	0.699879	-3.046019	-4.751654
C	-1.154509	0.069098	3.966280
C	-0.152944	-1.231023	-3.940839
C	0.092024	-0.001560	4.587182
C	-0.096439	0.003219	-4.587096
H	0.113675	-0.002026	5.675372
H	-0.120032	0.003897	-5.675247
C	1.312760	-0.071699	3.917127
C	-0.011490	1.236660	-3.942402
C	2.541365	-0.114864	4.795918
C	0.012025	2.448094	-4.845480
H	3.147421	0.793546	4.669434
H	-0.892878	3.057417	-4.709944
H	3.200411	-0.958591	4.550725
H	0.861521	3.110909	-4.632402
H	2.250430	-0.189019	5.851154
H	0.061860	2.136810	-5.896324
C	-2.669903	0.205944	2.214211
C	-0.250450	-2.712605	-2.157909
C	-3.233147	1.502470	2.103758
C	-1.543791	-3.274409	-2.009460
C	-4.567583	1.603068	1.694922
C	-1.634512	-4.600261	-1.571430
H	-5.028371	2.587027	1.591098
H	-2.615611	-5.059584	-1.438110
C	-5.334510	0.467136	1.443438
C	-0.492778	-5.361263	-1.328302
H	-6.372089	0.569707	1.114697
H	-0.587415	-6.392056	-0.976630
C	-4.796795	-0.794969	1.672779
C	0.763667	-4.827750	-1.595356
H	-5.431624	-1.676228	1.550944
H	1.647896	-5.459643	-1.479743
C	-3.469142	-0.953391	2.091982
C	0.911919	-3.508654	-2.044255
C	-2.446036	2.726346	2.574470
C	-2.777697	-2.498089	-2.471618
H	-1.891390	2.393882	3.466758
H	-2.463049	-1.960217	-3.380474
C	-3.338539	3.881820	3.033205
C	-3.940357	-3.400625	-2.890947
H	-2.722046	4.671319	3.491151
H	-4.739023	-2.794142	-3.346347
H	-3.878786	4.346726	2.192752
H	-4.388906	-3.924390	-2.031393
C	-1.386569	3.233656	1.593062
C	-3.267373	-1.420747	-1.500954
H	-1.837905	3.815628	0.780242
H	-3.833529	-1.857034	-0.669071
H	-0.671760	3.892279	2.110419
H	-3.936291	-0.716044	-2.019047
C	-2.953434	-2.326591	2.517297
C	2.275466	-3.000896	-2.508283
H	-2.059718	-2.152096	3.134588
H	2.087446	-2.119770	-3.139542
C	-3.965482	-3.082665	3.387561
C	3.014140	-4.029916	-3.373529
H	-3.502260	-3.990327	3.806285
H	3.912948	-3.575099	-3.819657
H	-4.334412	-2.472827	4.227357
H	2.387501	-4.415326	-4.193304
C	-2.496491	-3.201933	1.352981
C	3.173972	-2.520557	-1.371304
H	-3.343492	-3.486955	0.710534
H	3.473335	-3.354716	-0.718508
H	-1.754840	-2.687999	0.726961
H	2.672037	-1.767034	-0.749719
C	2.757646	-0.207976	2.106684
C	0.163011	2.715745	-2.163058
C	3.552014	0.950953	1.953233
C	-0.992939	3.512713	-2.001458
C	4.861772	0.792172	1.481048
C	-0.825276	4.831219	-1.557647
H	5.491683	1.673203	1.334587
H	-1.703414	5.463786	-1.405442
C	5.389190	-0.470061	1.229529
C	0.441608	5.363477	-1.342113
H	6.412729	-0.572981	0.859477
H	0.551546	6.393906	-0.993834
C	4.632463	-1.605711	1.511077

C 1.571677 4.601482 -1.631938
H 5.088438 -2.589809 1.388603
H 2.557877 5.059510 -1.537550
C 3.315551 -1.504725 1.973192
C 1.461923 3.275910 -2.066524
C 3.054684 2.324288 2.399540
C -2.374570 3.006024 -2.410066
H 2.187069 2.149967 3.053069
H -2.212438 2.126741 -3.051021
C 2.550748 3.199312 1.254550
C -3.225339 2.521430 -1.238795
H 3.370645 3.482587 0.577055
H -3.498496 3.353174 -0.571610
H 1.783053 2.685559 0.660635
H -2.697732 1.766496 -0.640756
C 4.101953 3.080027 3.227352
C -3.148873 4.036932 -3.241339
H 3.656687 3.987792 3.664900
H -4.064878 3.582622 -3.651590
H 4.505008 2.469975 4.051156
H -2.556701 4.425247 -4.085035
C 2.547577 -2.728717 2.474079
C 2.675627 2.498070 -2.577343
H 2.026544 -2.396223 3.386357
H 2.325177 1.963000 -3.474742
C 1.452804 -3.236859 1.532928
C 3.200516 1.417437 -1.628796
H 1.874327 -3.817030 0.703122
H 3.799001 1.850614 -0.818205
H 0.759051 -3.897208 2.076120
H 3.847769 0.713152 -2.174189
C 3.457683 -3.883324 2.899150
C 3.823275 3.399053 -3.039162
H 2.859830 -4.672710 3.381352
H 4.602724 2.791811 -3.525775
H 3.965248 -4.348774 2.038868
H 4.305375 3.920334 -2.196432
H 4.232348 -3.562458 3.613460
H 3.490206 4.161991 -3.760456
H 0.867629 -2.417501 1.085550
H 2.389443 0.841652 -1.154798
H -4.843819 -3.408878 2.808049
H 3.352223 -4.896702 -2.783524
H -2.039070 -4.132489 1.724747
H 4.096133 -2.069696 -1.770869
H 4.955806 3.405916 2.612182
H -3.463290 4.901712 -2.635535
H 2.110174 4.130727 1.644018
H -4.162623 2.070841 -1.601884
H -4.085102 3.562082 3.777314
H -3.633539 -4.161639 -3.625799
H -0.819773 2.414049 1.123031
H -2.439132 -0.845159 -1.057591

146

(BDI)MgMg(BDI) with dispersion

Mg -1.434392 0.125374 -0.093277
Mg 1.428171 -0.167684 0.071355
N -2.994934 -0.845781 -1.112582
N 2.789681 -1.249831 1.243939
N -2.891815 1.378706 0.730931
N 3.083243 0.589607 -0.967441
C -5.244205 -1.468382 -1.848682
C 4.894281 -2.102499 2.156612
H -4.919946 -1.601841 -2.890658
H 4.486512 -3.117229 2.268037
H -6.265764 -1.069406 -1.838178
H 5.954593 -2.167925 1.883861
H -5.256916 -2.474199 -1.400444
H 4.817258 -1.630314 3.148406
C -4.294483 -0.579416 -1.083854
C 4.111744 -1.298853 1.147362
C -4.858252 0.478114 -0.347916
C 4.849018 -0.607683 0.168842
H -5.938266 0.594569 -0.428760
H 5.928141 -0.754459 0.190988
C -4.201756 1.388563 0.497001
C 4.367745 0.278667 -0.808707
C -5.054176 2.435706 1.170053
C 5.388625 0.909265 -1.723538
H -4.721721 3.444080 0.881106
H 5.174446 0.655261 -2.772536
H -4.949574 2.382558 2.264042
H 5.345032 2.006979 -1.661323
H -6.112945 2.322918 0.907517
H 6.405759 0.579581 -1.479823
C -2.514026 -1.904450 -1.918896
C 2.132795 -1.998033 2.249780
C -2.145952 -1.640741 -3.260103
C 1.720064 -3.322453 1.967421
C -1.555810 -2.678802 -3.988472
C 0.970795 -3.991399 2.940707
H -1.259098 -2.513581 -5.024250
H 0.633033 -5.011699 2.758691
C -1.325442 -3.928857 -3.415925
C 0.639135 -3.380295 4.149151
H -0.844454 -4.715105 -4.002782
H 0.042445 -3.922544 4.886580
C -1.728343 -4.180352 -2.108872
C 1.084659 -2.091694 4.422593
H -1.569326 -5.170612 -1.674886
H 0.845466 -1.629722 5.383817
C -2.344524 -3.182702 -1.346461
C 1.843060 -1.382440 3.485739
C -2.459619 -0.287239 -3.895531
C 2.152473 -4.006786 0.671724
H -3.531922 -0.104370 -3.703827
H 3.248968 -3.885061 0.616376
C -2.284090 -0.283329 -5.412852
C 1.882767 -5.510175 0.669864
H -2.646494 0.668219 -5.829814
H 2.327329 -5.969782 -0.225512
H -1.225779 -0.382404 -5.702593
H 0.803836 -5.731654 0.646718
C -1.712292 0.904437 -3.276707
C 1.590997 -3.370670 -0.609526
H -1.832665 1.797401 -3.908174
H 1.762345 -4.032670 -1.471453
H -2.123437 1.165909 -2.292523
H 2.099020 -2.424988 -0.841891
C -2.839261 -3.467551 0.060993
C 2.361161 0.012105 3.794435
H -3.469746 -2.616510 0.357900
H 3.065475 0.276471 2.991926
C -3.704525 -4.727478 0.121980
C 3.129339 0.058065 5.116701

H	-4.140300	-4.847407	1.126302
H	3.580572	1.051460	5.267003
H	-4.529097	-4.687789	-0.606248
H	3.935528	-0.690885	5.143525
C	-1.688879	-3.542780	1.061564
C	1.244863	1.055301	3.772428
H	-1.021906	-4.386192	0.831722
H	0.528887	0.888637	4.590886
H	-1.070909	-2.630862	1.050873
H	0.673137	1.029900	2.831555
C	-2.343420	2.327207	1.630284
C	2.718858	1.535305	-1.957956
C	-1.856530	3.554792	1.133509
C	2.369333	1.088703	-3.249919
C	-1.190294	4.411062	2.016521
C	1.857028	2.019772	-4.159874
H	-0.796075	5.362210	1.650034
H	1.557380	1.689606	-5.157560
C	-1.036006	4.076679	3.359281
C	1.741857	3.364602	-3.818409
H	-0.508962	4.754688	4.034911
H	1.341229	4.080280	-4.540401
C	-1.579970	2.890090	3.848643
C	2.167028	3.803873	-2.565618
H	-1.486422	2.657511	4.911151
H	2.111014	4.867269	-2.325444
C	-2.244322	1.998783	3.001922
C	2.658414	2.905561	-1.614742
C	-2.086886	3.938029	-0.318574
C	2.585661	-0.362944	-3.641445
H	-2.833804	3.235534	-0.716624
H	3.238792	-0.804852	-2.874576
C	-0.827507	3.776868	-1.167177
C	1.286949	-1.163035	-3.631855
H	-0.059330	4.507129	-0.876020
H	0.593914	-0.795008	-4.401500
H	-0.377591	2.776645	-1.060968
H	0.762074	-1.094005	-2.665596
C	-2.664038	5.347515	-0.458440
C	3.301051	-0.496556	-4.986723
H	-2.924726	5.554211	-1.508322
H	3.551671	-1.550322	-5.185829
H	-3.571835	5.476319	0.150850
H	4.234097	0.087250	-5.009093
C	-2.883424	0.717770	3.515591
C	3.154452	3.360214	-0.250685
H	-3.724749	0.497989	2.841233
H	3.928248	2.639412	0.053939
C	-1.941186	-0.484651	3.437758
C	2.068040	3.304666	0.825193
H	-1.139262	-0.414224	4.183293
H	1.318527	4.093915	0.683584
H	-2.485152	-1.424522	3.616685
H	2.503046	3.430327	1.828097
C	-3.456899	0.859844	4.924161
C	3.808034	4.740311	-0.281661
H	-4.022002	-0.044300	5.198916
H	4.266897	4.966907	0.693202
H	-2.664687	0.986179	5.679204
H	3.075176	5.537147	-0.485330
H	-4.134865	1.723868	5.000737
H	4.592694	4.800448	-1.051525
H	-1.443464	-0.568935	2.457888
H	1.515591	2.351290	0.818844
H	-3.118300	-5.635654	-0.090367
H	2.469149	-0.135413	5.977154
H	-2.057748	-3.679371	2.089316
H	1.648692	2.072146	3.890226
H	-1.941385	6.117285	-0.144186
H	2.671377	-0.148058	-5.820669
H	-1.043492	3.932819	-2.234745
H	1.470782	-2.229515	-3.830650
H	-2.844932	-1.097762	-5.895361
H	2.311899	-6.007861	1.552379
H	-0.634956	0.722243	-3.170149
H	0.510733	-3.184509	-0.551909

146

(BDI)MgMg(BDI) withOut dispersion

Mg	-1.562963	0.002295	0.002365
Mg	1.563420	-0.000596	-0.001311
N	-3.095763	-1.052978	1.047310
N	3.099344	1.042453	1.051483
N	-3.096554	1.059858	-1.039535
N	3.093665	-1.048703	-1.057310
C	-5.358769	-1.737475	1.728198
C	5.364315	1.720663	1.731726
H	-5.194084	-2.810461	1.550303
H	5.199278	1.548800	2.805577
H	-6.405233	-1.501259	1.499267
H	6.410108	1.487709	1.496476
H	-5.189549	-1.576289	2.803039
H	5.198062	2.795167	1.565032
C	-4.411910	-0.899429	0.898787
C	4.414970	0.890215	0.897489
C	-5.014123	0.005403	0.006635
C	5.014433	-0.005940	-0.005260
H	-6.103499	0.006469	0.008099
H	6.103806	-0.007428	-0.006718
C	-4.412555	0.908892	-0.887336
C	4.410093	-0.900244	-0.906633
C	-5.360016	1.748423	-1.714641
C	5.354927	-1.732479	-1.744158
H	-5.194606	1.585435	-2.789842
H	5.185324	-2.806761	-1.579419
H	-5.192130	2.821207	-1.538742
H	5.188345	-1.557726	-2.817332
H	-6.406337	1.515059	-1.482144
H	6.401956	-1.503502	-1.510535
C	-2.629076	-2.010966	1.992522
C	2.635528	1.991309	2.007233
C	-2.385504	-3.343829	1.576121
C	2.392096	1.579014	3.341391
C	-1.777900	-4.222338	2.480970
C	1.786630	2.487300	4.217865
H	-1.568585	-5.250505	2.173849
H	1.576889	2.183142	5.246811
C	-1.448405	-3.816550	3.772027
C	1.459941	3.778100	3.809045
H	-0.966520	-4.515415	4.460747
H	0.979998	4.469658	4.506443
C	-1.770721	-2.529062	4.194366
C	1.782353	4.196364	2.520248
H	-1.555739	-2.234836	5.225173
H	1.569517	5.226893	2.223522
C	-2.379045	-1.611702	3.329349

C	2.387595	3.327471	1.604523
C	-2.830811	-3.831513	0.201053
C	2.834602	0.204093	3.832122
H	-3.493557	-3.056499	-0.211591
H	3.500633	-0.209196	3.060222
C	-3.646664	-5.127451	0.284876
C	3.644631	0.288069	5.131705
H	-4.058236	-5.386566	-0.703801
H	4.055639	-0.700482	5.392168
H	-3.031797	-5.980642	0.613586
H	3.025671	0.615955	5.982243
C	-1.673841	-3.988823	-0.788770
C	1.676733	-0.785360	3.984786
H	-2.052401	-4.231951	-1.794460
H	2.053897	-1.790473	4.232535
H	-1.069302	-3.071735	-0.870288
H	1.077971	-0.869090	3.064148
C	-2.818204	-0.241417	3.835517
C	2.825573	3.828989	0.232168
H	-3.482285	0.182496	3.067732
H	3.484228	3.056980	-0.192644
C	-3.629699	-0.338589	5.133341
C	3.644446	5.122497	0.325144
H	-4.038159	0.647957	5.405216
H	4.050421	5.391533	-0.663210
H	-4.473804	-1.039360	5.038813
H	4.490822	5.023941	1.022613
C	-1.657941	0.742971	4.000861
C	1.663223	3.999577	-0.748943
H	-0.976811	0.425275	4.804624
H	0.988210	4.808445	-0.431099
H	-1.058591	0.836144	3.081607
H	1.057533	3.084141	-0.838250
C	-2.630659	2.016017	-1.986992
C	2.625070	-1.995742	-2.012546
C	-2.385122	1.615156	-3.324221
C	2.374913	-3.331479	-1.609760
C	-1.777163	2.530507	-4.191624
C	1.765254	-4.198538	-2.524256
H	-1.565762	2.235083	-5.222804
H	1.550697	-5.228670	-2.227427
C	-1.450500	3.817500	-3.771123
C	1.440307	-3.778903	-3.811981
H	-0.968733	4.514695	-4.461618
H	0.957004	-4.469047	-4.508460
C	-1.775352	4.224922	-2.479464
C	1.768640	-2.488584	-4.220939
H	-1.562574	5.252741	-2.173624
H	1.556788	-2.183381	-5.249177
C	-2.382789	3.348579	-1.572367
C	2.378733	-1.582164	-3.345745
C	-2.828501	0.245447	-3.828348
C	2.815077	-3.834523	-0.238600
H	-3.491984	-0.176510	-3.058943
H	3.478036	-3.064971	0.183926
C	-1.670919	-0.741853	-3.995262
C	1.655304	-4.000442	0.746358
H	-0.990861	-0.426663	-4.800861
H	0.975714	-4.806310	0.430696
H	-1.069548	-0.835504	-3.077430
H	1.053849	-3.082493	0.838155
C	-3.642538	0.343138	-5.124477
C	3.628720	-5.131174	-0.333886
H	-4.053383	-0.642872	-5.394685
H	4.037383	-5.401181	0.653087
H	-4.485132	1.045552	-5.028569
H	4.472839	-5.036289	-1.034606
C	-2.823320	3.838296	-0.196486
C	2.823349	-0.208463	-3.837831
H	-3.488878	3.066117	0.217059
H	3.488457	0.205514	-3.065534
C	-1.664019	3.990341	0.791393
C	1.666653	0.781712	-3.994580
H	-0.981613	4.797115	0.484540
H	0.984764	0.473943	-4.801551
H	-2.039733	4.237997	1.797051
H	2.045232	1.785627	-4.244966
C	-3.633414	5.137999	-0.278499
C	3.635564	-0.295830	-5.135898
H	-4.042433	5.398279	0.710933
H	4.047483	0.691884	-5.398150
H	-3.014955	5.988515	-0.607365
H	3.017662	-0.625616	-5.986473
H	-4.477008	5.052784	-0.981104
H	4.477323	-1.000212	-5.047444
H	-1.065414	3.069564	0.874208
H	1.067195	0.868890	-3.074802
H	-3.012277	-0.678370	5.980351
H	3.032952	5.973009	0.666853
H	-2.032595	1.746028	4.260409
H	2.036408	4.254488	-1.753724
H	-3.026414	0.681056	-5.973160
H	3.012675	-5.979537	-0.672763
H	-2.048577	-1.744301	-4.252765
H	2.030936	-4.257324	1.749729
H	-4.488890	-5.037773	0.988578
H	4.486823	0.992309	5.046123
H	-0.996274	-4.800646	-0.484582
H	0.994254	-0.479151	4.791685

146

(BDI)CaCa(BDI) with dispersion

Ca	1.767358	-0.090234	0.035513
Ca	-1.774711	0.054201	-0.123382
N	3.478352	-1.279227	1.102776
N	-3.734536	-0.805513	-1.063235
N	3.724941	0.777848	-0.840464
N	-3.493642	1.148704	0.982927
C	5.595611	-2.161238	1.988790
C	-6.029398	-1.438855	-1.673611
H	4.946263	-2.813440	2.587374
H	-5.535783	-2.137483	-2.361897
H	6.242153	-1.584745	2.668246
H	-6.706433	-2.005406	-1.016198
H	6.258021	-2.790876	1.375054
H	-6.655851	-0.750806	-2.262256
C	4.795108	-1.232495	1.104468
C	-5.026638	-0.654900	-0.858563
C	5.529606	-0.346789	0.283598
C	-5.544245	0.248650	0.096405
H	6.615888	-0.409858	0.360355
H	-6.631379	0.304051	0.166238
C	5.021026	0.580259	-0.644300
C	-4.818680	1.107688	0.942693
C	6.005411	1.379922	-1.459654
C	-5.597603	2.034239	1.841949

H	5.822403	2.457380	-1.330694
H	-5.309696	1.879201	2.892656
H	5.868719	1.170424	-2.531589
H	-5.355712	3.082236	1.608249
H	7.044467	1.158304	-1.186010
H	-6.680750	1.890713	1.743228
C	2.681582	-2.085418	1.925583
C	-3.130561	-1.728076	-1.924615
C	2.400760	-1.690570	3.260885
C	-2.963737	-3.080080	-1.519939
C	1.462387	-2.428938	3.985627
C	-2.191541	-3.919722	-2.325850
H	1.237446	-2.152170	5.016077
H	-2.053459	-4.963315	-2.041022
C	0.786797	-3.506726	3.415627
C	-1.569536	-3.449965	-3.482082
H	0.050026	-4.060370	4.002084
H	-0.962324	-4.126539	-4.087658
C	1.039342	-3.862397	2.095677
C	-1.706385	-2.116478	-3.849496
H	0.497405	-4.698785	1.649751
H	-1.202906	-1.749919	-4.746122
C	1.976931	-3.163383	1.329279
C	-2.475267	-1.235220	-3.083260
C	3.085355	-0.468186	3.855805
C	-3.584345	-3.569361	-0.218514
H	4.157154	-0.542799	3.609725
H	-4.627925	-3.214727	-0.201946
C	2.979413	-0.391438	5.376569
C	-3.626184	-5.090440	-0.099499
H	3.594634	0.438264	5.756187
H	-4.193037	-5.383238	0.797134
H	1.944175	-0.204215	5.703736
H	-2.615816	-5.518306	0.000472
C	2.574680	0.835263	3.223262
C	-2.891597	-2.961796	1.010628
H	2.973050	1.712169	3.756561
H	-3.259969	-3.427743	1.937604
H	2.920665	0.929265	2.182627
H	-3.114963	-1.887830	1.099967
C	2.284562	-3.566422	-0.102094
C	-2.660259	0.215112	-3.494720
H	2.674973	-2.666436	-0.612046
H	-2.937556	0.774094	-2.582024
C	3.419890	-4.594232	-0.135454
C	-3.852754	0.349131	-4.447203
H	3.694763	-4.844121	-1.172532
H	-4.040003	1.405037	-4.699228
H	4.316214	-4.212702	0.373997
H	-4.767156	-0.063797	-3.997206
C	1.062259	-4.042272	-0.881001
C	-1.398819	0.858161	-4.065167
H	0.663643	-4.992066	-0.490327
H	-1.103907	0.409165	-5.026832
H	0.256390	-3.291554	-0.851649
H	-0.552031	0.761253	-3.366687
C	3.204475	1.658627	-1.806682
C	-2.772232	2.051768	1.787188
C	2.812568	2.966332	-1.423312
C	-2.289499	1.635138	3.053008
C	2.105804	3.754272	-2.336203
C	-1.410588	2.470201	3.749173
H	1.795288	4.761272	-2.045450
H	-1.030256	2.152192	4.723660
C	1.782097	3.277247	-3.603241
C	-1.008374	3.694857	3.223519
H	1.222289	3.904847	-4.300560
H	-0.317766	4.332643	3.780164
C	2.165777	1.990749	-3.972416
C	-1.482465	4.096574	1.977870
H	1.900153	1.614989	-4.964022
H	-1.156354	5.053440	1.562314
C	2.870822	1.163386	-3.094119
C	-2.354694	3.292488	1.239559
C	3.134103	3.499304	-0.038678
C	-2.699265	0.297731	3.641740
H	3.704029	2.713668	0.480796
H	-3.408788	-0.158913	2.934779
C	1.864032	3.764532	0.773202
C	-1.503570	-0.647973	3.773626
H	1.268720	4.577390	0.329174
H	-0.762162	-0.259854	4.489909
H	1.203665	2.880680	0.815535
H	-0.973993	-0.782770	2.814030
C	4.020080	4.745719	-0.101234
C	-3.424336	0.463856	4.979322
H	4.304413	5.073097	0.911337
H	-3.780929	-0.509277	5.352452
H	4.943063	4.555187	-0.669548
H	-4.293671	1.132320	4.886288
C	3.248725	-0.247859	-3.510155
C	-2.820588	3.735420	-0.136796
H	3.818965	-0.686751	-2.677091
H	-3.513540	2.963270	-0.504990
C	2.011894	-1.123311	-3.733296
C	-1.655061	3.819521	-1.127620
H	1.410870	-0.757930	-4.580648
H	-0.944701	4.612002	-0.844783
H	2.300021	-2.164567	-3.947917
H	-2.016931	4.039750	-2.144355
C	4.155563	-0.252493	-4.743039
C	-3.591609	5.056142	-0.080439
H	4.483335	-1.276477	-4.982450
H	-3.985200	5.320582	-1.074635
H	3.633205	0.144509	-5.628182
H	-2.946693	5.885101	0.252426
H	5.053263	0.363993	-4.584569
H	-4.440885	4.996822	0.616866
H	1.344464	-1.130324	-2.853419
H	-1.075074	2.880616	-1.167980
H	3.115436	-5.523731	0.371961
H	-3.663506	-0.198576	-5.384430
H	1.321678	-4.208753	-1.937372
H	-1.564242	1.931542	-4.243815
H	3.498918	5.585825	-0.587590
H	-2.759532	0.890378	5.747492
H	2.107758	4.052763	1.807634
H	-1.819742	-1.642290	4.125244
H	3.325791	-1.318235	5.858443
H	-4.108155	-5.556764	-0.972075
H	1.474874	0.895656	3.245047
H	-1.799620	-3.101204	0.973482

146

(BDI)CaCa(BDI) withOut dispersion

Ca	-1.938186	0.013208	0.008460

Ca	1.937549	-0.008919	0.000606
N	-3.746188	1.080363	-1.070164
N	3.738092	-1.105857	-1.060684
N	-3.754462	-1.036905	1.090358
N	3.763663	1.061398	1.049530
C	-6.009940	1.754646	-1.734709
C	5.996213	-1.798323	-1.725765
H	-5.848761	1.568571	-2.807274
H	5.820558	-2.868840	-1.541974
H	-5.834236	2.829664	-1.579670
H	5.825619	-1.640430	-2.801213
H	-7.056764	1.531960	-1.493929
H	7.045255	-1.570719	-1.499643
C	-5.056254	0.929253	-0.899626
C	5.049686	-0.952061	-0.903814
C	-5.650709	0.037684	0.018401
C	5.651290	-0.040885	-0.010383
H	-6.740561	0.047041	0.023152
H	6.741134	-0.050634	-0.013808
C	-5.063662	-0.865036	0.929841
C	5.071474	0.882633	0.885193
C	-6.023195	-1.679064	1.769259
C	6.038907	1.713575	1.698610
H	-5.850439	-1.500385	2.841195
H	5.880273	2.786780	1.515008
H	-5.863723	-2.755932	1.609189
H	5.874360	1.560113	2.775626
H	-7.068577	-1.440657	1.537530
H	7.082036	1.467890	1.464234
C	-3.246459	2.005583	-2.024601
C	3.229119	-2.050699	-1.991209
C	-2.937706	3.334740	-1.628430
C	2.931573	-1.646748	-3.320553
C	-2.329966	4.191020	-2.553955
C	2.314341	-2.561259	-4.181821
H	-2.086612	5.214686	-2.256027
H	2.078265	-2.256764	-5.205296
C	-2.029769	3.768670	-3.845418
C	1.994891	-3.849789	-3.764326
H	-1.552371	4.452609	-4.551901
H	1.510298	-4.547597	-4.452105
C	-2.345589	2.469425	-4.231179
C	2.300953	-4.243787	-2.465083
H	-2.113868	2.142519	-5.248728
H	2.055121	-5.259347	-2.142295
C	-2.954308	1.572302	-3.345883
C	2.918328	-3.369246	-1.563226
C	-3.254116	3.847968	-0.229368
C	3.268958	-0.250571	-3.828197
H	-3.807400	3.051236	0.291493
H	3.822971	0.261415	-3.026210
C	-4.157489	5.087263	-0.255540
C	4.180264	-0.285454	-5.061572
H	-4.443304	5.379064	0.767833
H	4.477971	0.735193	-5.350956
H	-3.651130	5.952219	-0.713557
H	3.675249	-0.738144	-5.930165
C	-1.979258	4.124450	0.576182
C	2.006145	0.571711	-4.111459
H	-2.222688	4.424799	1.608148
H	2.264257	1.600813	-4.409343
H	-1.325623	3.237333	0.628164
H	1.346760	0.630882	-3.228849
C	-3.287791	0.163482	-3.820073
C	3.243200	-3.851195	-0.154932
H	-3.829072	-0.334500	-3.000673
H	3.797886	-3.041714	0.344504
C	-4.213826	0.167234	-5.042927
C	4.148728	-5.089320	-0.159293
H	-4.509383	-0.860788	-5.307528
H	4.439252	-5.359641	0.868641
H	-5.132658	0.744701	-4.858713
H	5.070882	-4.919899	-0.735992
C	-2.023245	-0.657416	-4.101101
C	1.973833	-4.111632	0.664897
H	-1.442241	-0.226941	-4.932530
H	1.378948	-4.932900	0.233742
H	-1.352766	-0.695063	-3.225908
H	1.318790	-3.224824	0.702269
C	-3.262854	-1.976059	2.035617
C	3.279397	2.020566	1.978712
C	-2.957924	-1.556885	3.358648
C	2.992149	3.343631	1.547582
C	-2.359254	-2.469643	4.234869
C	2.400553	4.234656	2.450495
H	-2.118994	-2.154013	5.253985
H	2.173754	5.253757	2.125087
C	-2.064612	-3.770572	3.838062
C	2.095762	3.852678	3.753606
H	-1.595246	-4.467116	4.537584
H	1.631455	4.563469	4.442064
C	-2.376709	-4.178693	2.544789
C	2.390601	2.559283	4.173741
H	-2.150330	-5.203694	2.238230
H	2.156086	2.263834	5.200197
C	-2.976183	-3.306708	1.628518
C	2.982326	1.628645	3.311719
C	-3.270556	-0.147205	3.844700
C	3.315100	3.814719	0.135207
H	-3.814402	0.361990	3.033907
H	3.842632	2.990213	-0.369151
C	-1.993375	0.656700	4.116087
C	2.044630	4.108895	-0.671205
H	-1.407557	0.214009	4.937624
H	1.474841	4.943524	-0.231946
H	-1.333642	0.690899	3.232761
H	1.367488	3.238995	-0.704387
C	-4.183704	-0.145896	5.077031
C	4.253023	5.028575	0.128178
H	-4.463999	0.884280	5.349529
H	4.536421	5.292406	-0.903431
H	-5.111363	-0.711146	4.899182
H	5.178291	4.834339	0.691894
C	-3.308228	-3.805047	0.227500
C	3.295021	0.227944	3.823534
H	-3.841022	-2.990990	-0.288242
H	3.829811	-0.299821	3.018695
C	-2.043572	-4.112946	-0.583275
C	2.018389	-0.566479	4.124691
H	-1.469780	-4.939015	-0.133237
H	1.442099	-0.104011	4.942110
H	-2.299715	-4.407183	-1.613924
H	2.259160	-1.598308	4.427725
C	-4.245231	-5.019610	0.250320
C	4.219656	0.249876	5.047344
H	-4.537960	-5.301829	-0.773772

H	4.497773	-0.775300	5.340204
H	-3.762292	-5.898350	0.707286
H	3.733280	0.718868	5.917842
H	-5.165554	-4.816405	0.818981
H	5.148398	0.806588	4.849181
H	-1.367116	-3.243475	-0.638901
H	1.349353	-0.619087	3.249189
H	-3.720969	0.604384	-5.926259
H	3.641670	-5.964014	-0.597485
H	-2.279696	-1.693818	-4.374225
H	2.225095	-4.389210	1.701388
H	-3.687418	-0.593208	5.953315
H	3.775194	5.915776	0.574075
H	-2.233765	1.694603	4.397983
H	2.292543	4.383331	-1.709311
H	-5.082367	4.906943	-0.824499
H	5.098354	-0.863584	-4.875628
H	-1.385744	4.934759	0.122988
H	1.412389	0.126598	-4.926126

146

(BDI)SrSr(BDI) with dispersion

Sr	2.211953	-0.827291	-0.717010
Sr	-1.683894	0.020518	-0.271276
N	2.248597	-1.597826	1.739037
N	-3.935515	-0.464722	-1.315934
N	4.519436	-0.411476	0.253565
N	-2.816574	2.221350	-0.784226
C	2.858846	-2.220165	4.043449
C	-6.163924	-0.411189	-2.370169
H	1.930614	-2.805977	4.058776
H	-6.151126	-1.498055	-2.216980
H	2.719718	-1.364971	4.724035
H	-7.072766	0.003329	-1.908569
H	3.676038	-2.830210	4.452544
H	-6.238365	-0.213788	-3.450999
C	3.203600	-1.727306	2.652104
C	-4.923099	0.257174	-1.814293
C	4.557433	-1.401418	2.449386
C	-4.906643	1.663094	-1.878406
H	5.219925	-1.614187	3.290132
H	-5.778687	2.119637	-2.349896
C	5.155314	-0.720389	1.367345
C	-3.959616	2.569381	-1.358982
C	6.596434	-0.304657	1.566793
C	-4.349234	4.029931	-1.451818
H	6.646862	0.560694	2.246706
H	-5.027196	4.286866	-0.622329
H	7.075676	-0.015597	0.622442
H	-3.480185	4.696799	-1.387411
H	7.173567	-1.116194	2.032731
H	-4.889899	4.230354	-2.386925
C	0.899321	-1.780968	2.063692
C	-4.032852	-1.826580	-1.008544
C	0.184054	-0.793766	2.795022
C	-4.764681	-2.249403	0.137810
C	-1.193142	-0.968067	2.995897
C	-4.671233	-3.589615	0.523647
H	-1.745096	-0.232077	3.585940
H	-5.219071	-3.938530	1.400013
C	-1.869864	-2.072293	2.470359
C	-3.897276	-4.502013	-0.192296
H	-2.940180	-2.203668	2.634615
H	-3.841794	-5.543982	0.131697
C	-1.164269	-3.032179	1.743435
C	-3.211718	-4.086481	-1.329314
H	-1.708454	-3.880519	1.327885
H	-2.630991	-4.812635	-1.900164
C	0.215074	-2.917470	1.544838
C	-3.260069	-2.755908	-1.756162
C	0.875880	0.458421	3.306013
C	-5.662500	-1.268366	0.884545
H	1.958471	0.290556	3.227998
H	-6.277092	-0.769110	0.119476
C	0.549514	0.752530	4.770741
C	-6.634367	-1.944096	1.848414
H	1.157127	1.594373	5.137158
H	-7.330091	-1.199330	2.263629
H	-0.507155	1.033716	4.905135
H	-6.110839	-2.408459	2.699837
C	0.552532	1.662112	2.419693
C	-4.899537	-0.155130	1.608144
H	1.110704	2.555317	2.736258
H	-5.605111	0.547441	2.076739
H	0.808915	1.466916	1.363626
H	-4.287854	0.430777	0.907635
C	0.995271	-4.001761	0.825876
C	-2.594458	-2.308800	-3.047348
H	1.759498	-3.491237	0.207045
H	-2.267702	-1.261446	-2.902462
C	1.765057	-4.883633	1.814411
C	-3.616745	-2.289944	-4.189245
H	2.324100	-5.664618	1.275395
H	-3.154797	-1.919027	-5.117551
H	2.486222	-4.301617	2.401414
H	-4.471213	-1.643664	-3.954058
C	0.144183	-4.851917	-0.109200
C	-1.366355	-3.120719	-3.439770
H	-0.558411	-5.492963	0.446371
H	-1.616517	-4.168884	-3.669192
H	-0.440852	-4.227029	-0.797358
H	-0.602257	-3.100898	-2.647860
C	5.079203	0.389709	-0.752460
C	-1.958631	3.191777	-0.227617
C	5.104719	1.801167	-0.617146
C	-2.223884	3.742998	1.050679
C	5.518544	2.574296	-1.705548
C	-1.289202	4.613636	1.619802
H	5.537692	3.663180	-1.609905
H	-1.484603	5.037548	2.608301
C	5.900348	1.988300	-2.909262
C	-0.115212	4.945339	0.953441
H	6.217271	2.611865	-3.748444
H	0.606782	5.624596	1.412754
C	5.871251	0.602155	-3.038765
C	0.136643	4.407052	-0.306188
H	6.174306	0.145849	-3.983483
H	1.055630	4.678514	-0.825452
C	5.463663	-0.215142	-1.980118
C	-0.762545	3.529905	-0.918378
C	4.663317	2.477292	0.667959
C	-3.488617	3.397122	1.816944
H	4.464383	1.687638	1.406676
H	-4.136369	2.819486	1.142847
C	3.353353	3.238911	0.462935
C	-3.182576	2.503096	3.020041

H	3.466698	4.032271	-0.292671
H	-2.499738	3.000976	3.726535
H	2.548756	2.570369	0.119609
H	-2.704296	1.564636	2.705452
C	5.752166	3.388328	1.238988
C	-4.260709	4.645456	2.249727
H	5.448229	3.789022	2.218941
H	-5.229140	4.368009	2.695110
H	6.701264	2.847418	1.369061
H	-4.453517	5.313095	1.397305
C	5.495554	-1.728650	-2.100748
C	-0.502985	2.993097	-2.316995
H	4.768545	-2.122686	-1.366239
H	-0.823607	1.933388	-2.323656
C	5.094910	-2.244915	-3.480688
C	0.967232	3.006534	-2.718398
H	5.836896	-1.982617	-4.251076
H	1.352881	4.030175	-2.850864
H	5.014071	-3.342842	-3.468755
H	1.099519	2.477678	-3.674214
C	6.863101	-2.275071	-1.678028
C	-1.349562	3.719302	-3.367188
H	6.877717	-3.375656	-1.723098
H	-1.125680	3.330410	-4.372933
H	7.655371	-1.893907	-2.342314
H	-1.133893	4.799912	-3.364134
H	7.111389	-1.974082	-0.651014
H	-2.423764	3.583238	-3.191833
H	4.121518	-1.836917	-3.798313
H	1.591465	2.493018	-1.971506
H	1.072133	-5.380333	2.512015
H	-4.002345	-3.304553	-4.379497
H	0.786873	-5.513798	-0.709071
H	-0.905843	-2.688712	-4.340934
H	5.948556	4.248216	0.578856
H	-3.706048	5.224888	3.004588
H	3.014463	3.708792	1.399027
H	-4.102955	2.239876	3.563958
H	0.747336	-0.119077	5.413143
H	-7.231431	-2.723450	1.351635
H	-0.515772	1.920353	2.458946
H	-4.258414	-0.558433	2.404629

146

(BDI)SrSr(BDI) withOut dispersion

Sr	-2.090552	0.009762	-0.007109
Sr	2.099610	-0.041047	0.085538
N	-4.047483	1.224623	-1.045292
N	4.074077	-1.278385	-0.880794
N	-4.150304	-1.089579	0.936579
N	4.142794	1.132158	0.970421
C	-6.305706	2.035623	-1.621392
C	6.324541	-2.054752	-1.479232
H	-5.828965	2.542969	-2.470125
H	6.018427	-3.104112	-1.361570
H	-6.653876	2.813171	-0.921769
H	6.301533	-1.844844	-2.560103
H	-7.197717	1.500652	-1.976839
H	7.359857	-1.944114	-1.132822
C	-5.358337	1.090294	-0.908433
C	5.385146	-1.124294	-0.741570
C	-5.988631	0.117132	-0.098364
C	6.002283	-0.124575	0.043338
H	-7.078856	0.147455	-0.124267
H	7.092098	-0.153420	0.040000
C	-5.448327	-0.878484	0.741843
C	5.443794	0.914746	0.818719
C	-6.453015	-1.762096	1.450410
C	6.429551	1.826464	1.517104
H	-6.258489	-1.790259	2.532503
H	6.248851	2.876925	1.244148
H	-6.369890	-2.801267	1.096507
H	6.306733	1.767877	2.609340
H	-7.483097	-1.422525	1.284621
H	7.467640	1.571772	1.269706
C	-3.463546	2.226077	-1.853503
C	3.530134	-2.284173	-1.715200
C	-3.250598	3.537114	-1.345736
C	3.313867	-2.029322	-3.098429
C	-2.562464	4.464356	-2.136833
C	2.659534	-2.995488	-3.870607
H	-2.398137	5.475384	-1.753657
H	2.489817	-2.804589	-4.933953
C	-2.082310	4.129559	-3.399151
C	2.219863	-4.194547	-3.317100
H	-1.549364	4.870401	-4.000580
H	1.711941	-4.935742	-3.939406
C	-2.280088	2.841166	-3.886932
C	2.429957	-4.438908	-1.963984
H	-1.899189	2.581188	-4.877570
H	2.085037	-5.381938	-1.530956
C	-2.957320	1.873042	-3.136616
C	3.074649	-3.505088	-1.143808
C	-3.739497	3.952782	0.035701
C	3.765778	-0.730487	-3.754675
H	-4.321770	3.112533	0.443593
H	4.350346	-0.173077	-3.006828
C	-4.668547	5.171935	-0.019129
C	4.677116	-0.971845	-4.964591
H	-5.082847	5.388982	0.978599
H	5.066445	-0.016793	-5.352322
H	-4.135235	6.075809	-0.355161
H	4.138582	-1.464288	-5.790154
C	-2.570722	4.204377	0.997311
C	2.572743	0.149801	-4.148953
H	-2.938746	4.448327	2.007113
H	2.913927	1.109540	-4.569840
H	-1.910787	3.324872	1.082895
H	1.921183	0.373587	-3.287338
C	-3.196161	0.486502	-3.720948
C	3.300398	-3.834192	0.326862
H	-3.433497	-0.180789	-2.873660
H	3.734174	-2.934339	0.793523
C	-4.426875	0.477472	-4.638819
C	4.319385	-4.968213	0.504238
H	-4.621126	-0.536594	-5.024340
H	4.515203	-5.153226	1.572827
H	-5.330115	0.815691	-4.111092
H	5.280310	-4.733109	0.023013
C	-1.976627	-0.091710	-4.444605
C	1.996164	-4.163484	1.063329
H	-1.745197	0.460361	-5.369590
H	1.536191	-5.089967	0.683656
H	-1.076514	-0.074471	-3.809297
H	1.245539	-3.362384	0.956265
C	-3.689273	-2.125164	1.784150

C	3.653398	2.188831	1.774616
C	-3.337972	-1.835920	3.132602
C	3.293135	3.427419	1.174706
C	-2.768257	-2.846040	3.916941
C	2.675891	4.407387	1.961270
H	-2.501882	-2.629781	4.955387
H	2.397857	5.361625	1.504927
C	-2.534072	-4.118553	3.405845
C	2.410389	4.195430	3.310397
H	-2.085781	-4.891989	4.034726
H	1.924562	4.972624	3.905824
C	-2.873878	-4.396702	2.085119
C	2.769438	2.984433	3.895158
H	-2.687527	-5.397339	1.685094
H	2.562320	2.821725	4.956583
C	-3.449930	-3.425857	1.258104
C	3.389777	1.971003	3.155894
C	-3.588415	-0.465561	3.750038
C	3.582316	3.719525	-0.292519
H	-3.987759	0.178160	2.949743
H	4.076654	2.826298	-0.706533
C	-2.301264	0.183790	4.274041
C	2.301430	3.958118	-1.101801
H	-1.867485	-0.389863	5.109028
H	1.759269	4.851058	-0.751218
H	-1.525478	0.255401	3.493278
H	1.598496	3.110429	-1.029640
C	-4.648332	-0.524753	4.858417
C	4.552719	4.896263	-0.460271
H	-4.864733	0.484264	5.245041
H	4.811368	5.038627	-1.521929
H	-5.594174	-0.955475	4.497370
H	5.488958	4.735392	0.095333
C	-3.800001	-3.783925	-0.181096
C	3.766335	0.665203	3.845138
H	-4.332397	-2.920965	-0.610079
H	4.333120	0.065210	3.116614
C	-2.543689	-4.015611	-1.030644
C	2.528035	-0.148507	4.241936
H	-1.960455	-4.874944	-0.662292
H	1.907200	0.392473	4.974191
H	-2.809849	-4.216560	-2.081092
H	2.817410	-1.111607	4.692875
C	-4.739325	-4.993182	-0.270770
C	4.675048	0.888873	5.060452
H	-5.050134	-5.165392	-1.313749
H	5.009554	-0.075129	5.476204
H	-4.251980	-5.916293	0.082079
H	4.154041	1.429882	5.866624
H	-5.648663	-4.849800	0.332638
H	5.571056	1.471969	4.798189
H	-1.866097	-3.144685	-1.017791
H	1.880022	-0.365971	3.375523
H	-4.275183	1.144309	-5.503466
H	3.949979	-5.909433	0.065424
H	-2.165766	-1.137678	-4.733974
H	2.183680	-4.308324	2.139467
H	-4.307675	-1.139506	5.707426
H	4.111704	5.839166	-0.098397
H	-2.502260	1.202368	4.643634
H	2.535106	4.109088	-2.168075
H	-5.510682	5.010287	-0.708707
H	5.537884	-1.608099	-4.708010
H	-1.942439	5.043762	0.658256
H	1.942139	-0.343515	-4.906092

146

(BDI)BaBa(BDI) with dispersion

Ba	1.844296	0.288255	-0.075963
Ba	-2.457592	-0.462108	-1.095606
N	3.305851	2.528559	-0.126322
N	-2.632676	-2.299543	0.941633
N	4.487232	-0.113501	-0.580295
N	-4.750790	-0.295381	0.334120
C	4.857990	4.407436	-0.466489
C	-3.318477	-3.916577	2.666580
H	3.986258	5.034067	-0.237311
H	-2.260834	-4.211273	2.670043
H	5.259006	4.702847	-1.447984
H	-3.642366	-3.729460	3.700867
H	5.645325	4.616403	0.274612
H	-3.908509	-4.770220	2.295844
C	4.515090	2.933042	-0.449555
C	-3.571485	-2.711470	1.780408
C	5.565729	2.048645	-0.778864
C	-4.842689	-2.125521	1.906149
H	6.519930	2.520685	-1.016720
H	-5.502464	-2.598296	2.636022
C	5.552942	0.643307	-0.819647
C	-5.364997	-0.966627	1.287229
C	6.863335	-0.031951	-1.159200
C	-6.694480	-0.482106	1.824166
H	6.723760	-0.750733	-1.979910
H	-6.532677	0.052808	2.774015
H	7.238169	-0.613722	-0.303119
H	-7.189849	0.211878	1.132826
H	7.633042	0.694515	-1.447585
H	-7.366437	-1.325529	2.036696
C	2.200200	3.368762	0.020946
C	-1.286941	-2.634263	1.059859
C	1.541425	3.897818	-1.124757
C	-0.502045	-2.116010	2.136875
C	0.343043	4.591609	-0.930204
C	0.881970	-2.331857	2.114781
H	-0.181710	5.010599	-1.788858
H	1.491335	-1.984285	2.950476
C	-0.208594	4.760923	0.338312
C	1.510739	-2.988154	1.051648
H	-1.146807	5.306378	0.454842
H	2.591451	-3.142471	1.052682
C	0.423576	4.211951	1.448009
C	0.739562	-3.468545	-0.006126
H	-0.022070	4.336454	2.436845
H	1.236115	-3.992571	-0.823625
C	1.622652	3.505785	1.312272
C	-0.654487	-3.333832	-0.004223
C	2.147159	3.722346	-2.513631
C	-1.161945	-1.383953	3.298282
H	3.185378	4.088920	-2.454443
H	-2.005434	-2.008266	3.631201
C	1.447397	4.550951	-3.587965
C	-0.246073	-1.208702	4.507161
H	1.990471	4.463399	-4.540919
H	-0.809653	-0.765449	5.341681
H	0.418619	4.198619	-3.763646
H	0.594207	-0.529859	4.290624

C	2.223408	2.253561	-2.957475
C	-1.758851	-0.036678	2.884805
H	2.537056	2.184109	-4.010348
H	-2.211192	0.467900	3.753066
H	2.973831	1.698716	-2.373947
H	-2.541551	-0.165494	2.126399
C	2.357998	2.945698	2.517194
C	-1.509210	-3.983787	-1.076978
H	2.936615	2.073319	2.159833
H	-2.413086	-3.356276	-1.210316
C	3.395811	3.946388	3.034365
C	-2.028243	-5.338317	-0.582703
H	3.964997	3.524602	3.878137
H	-2.704462	-5.792403	-1.324301
H	4.108315	4.219529	2.244413
H	-2.577483	-5.230801	0.361722
C	1.428606	2.479597	3.634882
C	-0.822759	-4.117401	-2.432297
H	0.962090	3.327543	4.160408
H	0.018105	-4.827719	-2.396835
H	0.613757	1.854452	3.239974
H	-0.433885	-3.150330	-2.787509
C	4.612692	-1.506814	-0.489774
C	-5.283029	0.857865	-0.260886
C	4.166523	-2.333701	-1.552274
C	-5.080961	2.134870	0.321323
C	4.178154	-3.723097	-1.383120
C	-5.484906	3.271537	-0.385492
H	3.837217	-4.363403	-2.201187
H	-5.331241	4.258307	0.059374
C	4.632792	-4.303652	-0.203831
C	-6.077181	3.172530	-1.641259
H	4.636986	-5.389944	-0.089529
H	-6.383798	4.073315	-2.178017
C	5.083001	-3.487265	0.834954
C	-6.272453	1.916958	-2.211293
H	5.435768	-3.948400	1.760289
H	-6.740434	1.842435	-3.195235
C	5.075832	-2.096086	0.719528
C	-5.882881	0.749642	-1.547190
C	3.745846	-1.729290	-2.879686
C	-4.431626	2.279891	1.684141
H	3.757075	-0.635329	-2.746846
H	-4.261494	1.265947	2.071851
C	2.335860	-2.142162	-3.300803
C	-3.068337	2.965195	1.579846
H	2.255952	-3.230974	-3.447366
H	-3.167772	3.990518	1.190987
H	1.574566	-1.855169	-2.555053
H	-2.384947	2.425833	0.902288
C	4.761438	-2.059783	-3.977998
C	-5.343918	3.007676	2.674277
H	4.495225	-1.555280	-4.920031
H	-4.895345	3.016646	3.680327
H	5.775741	-1.741084	-3.696256
H	-6.329350	2.523330	2.742685
C	5.502690	-1.218646	1.884043
C	-6.156711	-0.619009	-2.146979
H	5.768283	-0.233034	1.476211
H	-5.430864	-1.315426	-1.689516
C	4.335485	-0.990439	2.848627
C	-5.968813	-0.674140	-3.661550
H	3.971635	-1.941742	3.267946
H	-6.728861	-0.082217	-4.195544
H	4.629017	-0.334263	3.682976
H	-6.055676	-1.712152	-4.018383
C	6.724497	-1.755923	2.628079
C	-7.549736	-1.117948	-1.748769
H	7.071515	-1.024512	3.374224
H	-7.734165	-2.126533	-2.151529
H	6.504054	-2.690252	3.168145
H	-8.330505	-0.445320	-2.139307
H	7.556506	-1.958380	1.936730
H	-7.661704	-1.164378	-0.657247
H	3.487887	-0.503315	2.341121
H	-4.977165	-0.297017	-3.961144
H	2.902722	4.869333	3.379135
H	-1.189762	-6.031806	-0.410429
H	1.983908	1.897495	4.386193
H	-1.535009	-4.491955	-3.183074
H	4.795690	-3.143477	-4.174407
H	-5.509103	4.055795	2.377505
H	2.051525	-1.655340	-4.246579
H	-2.578894	3.023121	2.564520
H	1.404722	5.618220	-3.322901
H	0.171167	-2.166760	4.852782
H	1.236557	1.761436	-2.878311
H	-0.987441	0.626353	2.464270

146

(BDI)BaBa(BDI) withOut dispersion

Ba	2.374623	-0.044094	-0.011880
Ba	-2.374640	0.043421	-0.011712
N	4.668473	-1.234819	0.728343
N	-4.507193	-1.387919	-0.786817
N	4.506730	1.388363	-0.786461
N	-4.668327	1.234701	0.728495
C	7.000639	-1.862295	1.236441
C	-6.742378	-2.343551	-1.212054
H	6.593103	-2.847570	1.497875
H	-6.234029	-3.011116	-1.919929
H	7.392433	-1.405074	2.159677
H	-7.121089	-2.961137	-0.381339
H	7.855625	-1.997004	0.558231
H	-7.618272	-1.898319	-1.705574
C	5.957272	-0.953294	0.613672
C	-5.821068	-1.264707	-0.673740
C	6.475986	0.182762	-0.046072
C	-6.476214	-0.182358	-0.046000
H	7.566213	0.245747	-0.053259
H	-7.566460	-0.245172	-0.053064
C	5.820634	1.265173	-0.673518
C	-5.957203	0.953519	0.613817
C	6.741708	2.344402	-1.211445
C	-7.000449	1.862751	1.236492
H	7.117533	2.963880	-0.380816
H	-7.394292	1.404444	2.158310
H	6.233984	3.010236	-1.921406
H	-6.592232	2.847160	1.500086
H	7.619364	1.899725	-1.702286
H	-7.854196	1.999524	0.557112
C	4.171354	-2.367843	1.399730
C	-3.876460	-2.483169	-1.408249
C	3.942849	-2.341423	2.805028
C	-3.579627	-3.666257	-0.673441
C	3.317233	-3.437816	3.410073

C	-2.832133	-4.676499	-1.289803
H	3.144449	-3.424411	4.489936
H	-2.607043	-5.589593	-0.731491
C	2.909112	-4.543680	2.670750
C	-2.368455	-4.546881	-2.595326
H	2.421853	-5.387977	3.164796
H	-1.786657	-5.349040	-3.056209
C	3.117833	-4.560938	1.294688
C	-2.643084	-3.382040	-3.305645
H	2.793264	-5.429640	0.716604
H	-2.273594	-3.281475	-4.329189
C	3.736304	-3.491515	0.636645
C	-3.383299	-2.338170	-2.738473
C	4.368597	-1.158028	3.664163
C	-4.062899	-3.864755	0.757305
H	4.856531	-0.430728	2.996945
H	-4.642923	-2.970021	1.031785
C	5.393326	-1.564395	4.731365
C	-4.996908	-5.075628	0.882821
H	5.755270	-0.679865	5.279954
H	-5.407126	-5.146612	1.903137
H	4.955055	-2.253481	5.471370
H	-4.465959	-6.018892	0.675658
C	3.168005	-0.453942	4.309419
C	-2.897565	-3.978271	1.748720
H	3.493176	0.432897	4.877157
H	-3.270417	-4.060551	2.782551
H	2.431065	-0.119804	3.559475
H	-2.225910	-3.104333	1.702957
C	3.996899	-3.567618	-0.862687
C	-3.707653	-1.099938	-3.565332
H	4.184238	-2.534798	-1.206207
H	-4.020771	-0.315058	-2.854340
C	5.275902	-4.362982	-1.160858
C	-4.907083	-1.352833	-4.489915
H	5.487273	-4.374900	-2.242440
H	-5.170662	-0.440498	-5.049214
H	6.149663	-3.932335	-0.651445
H	-5.795127	-1.670276	-3.924836
C	2.816187	-4.125279	-1.663372
C	-2.514192	-0.565242	-4.363502
H	2.642370	-5.192828	-1.453750
H	-2.209340	-1.257619	-5.164479
H	1.877272	-3.589975	-1.446339
H	-1.632072	-0.391087	-3.724327
C	3.876133	2.483703	-1.407938
C	-4.170947	2.367524	1.400050
C	3.579489	3.666858	-0.673170
C	-3.942545	2.340887	2.805365
C	2.832282	4.677257	-1.289611
C	-3.316769	3.437089	3.410587
H	2.607296	5.590384	-0.731309
H	-3.144132	3.423559	4.490472
C	2.368748	4.547748	-2.595198
C	-2.908298	4.542920	2.671405
H	1.787181	5.350029	-3.056160
H	-2.420873	5.387049	3.165574
C	2.643271	3.382881	-3.305506
C	-3.116925	4.560384	1.295331
H	2.273922	3.282425	-4.329112
H	-2.792097	5.429071	0.717368
C	3.383189	2.338844	-2.738247
C	-3.735656	3.491207	0.637134

C	4.062503	3.865208	0.757680
C	-4.368607	1.157464	3.664318
H	4.643066	2.970763	1.031937
H	-4.856500	0.430285	2.996941
C	2.896933	3.977617	1.748947
C	-3.168178	0.453207	4.309721
H	2.276055	4.865297	1.547061
H	-2.634027	1.117885	5.007666
H	2.226000	3.103139	1.702980
H	-2.431050	0.119212	3.559893
C	4.995682	5.076654	0.883773
C	-5.393509	1.563797	4.731341
H	5.405781	5.147452	1.904148
H	-5.755305	0.679276	5.280046
H	5.840316	5.013262	0.181750
H	-6.264621	2.067211	4.286343
C	3.707416	1.100579	-3.565116
C	-3.996372	3.567623	-0.862152
H	4.020438	0.315660	-2.854126
H	-4.184137	2.534930	-1.205800
C	2.513931	0.566021	-4.363308
C	-2.815579	4.124972	-1.662926
H	2.208938	1.258562	-5.164082
H	-2.641180	5.192360	-1.452979
H	2.774775	-0.389150	-4.846600
H	-3.011919	4.038467	-2.743496
C	4.906875	1.353324	-4.489722
C	-5.275115	4.363550	-1.159978
H	5.170360	0.440943	-5.048987
H	-5.486775	4.375569	-2.241502
H	4.676762	2.144024	-5.222613
H	-5.172709	5.408458	-0.823864
H	5.794963	1.670694	-3.924674
H	-6.148919	3.933292	-0.650310
H	1.631862	0.391610	-3.724130
H	-1.876869	3.589126	-1.446339
H	5.174050	-5.407940	-0.824727
H	-4.676903	-2.143540	-5.222777
H	3.012202	-4.038311	-2.743962
H	-2.775002	0.390060	-4.846542
H	4.464158	6.019681	0.677015
H	-4.955498	2.253121	5.471277
H	3.269547	4.060064	2.782853
H	-3.493488	-0.433745	4.877205
H	6.264351	-2.068120	4.286553
H	-5.841446	-5.011356	0.180772
H	2.633698	-1.118768	5.007102
H	-2.277389	-4.866444	1.546847

(BDI)AeN$_2$Ae(BDI) Complexes [side-on]

148

(BDI)BeN$_2$Be(BDI) with dispersion [side-on]

N	-0.102471	2.890564	-0.000250
N	-2.420010	-1.918801	-0.000010
N	-0.000013	-0.000001	0.631655
C	0.441031	4.097887	-0.000510
C	-0.389187	5.356945	-0.000403
C	-1.518607	2.807764	-0.000177
C	-2.192024	2.829126	1.240263
C	-3.586158	2.717785	1.207822
H	-4.158116	2.723054	2.135543
C	-4.269579	2.646657	-0.000151
H	-5.356078	2.575474	-0.000132
C	-3.586186	2.717683	-1.208149
H	-4.158158	2.722894	-2.135862
C	-2.192053	2.828969	-1.240622
C	-1.454127	3.241631	2.518665
H	-0.960365	4.191006	2.245902
C	-0.329955	2.331910	3.020995
H	-0.699395	1.350741	3.337242
H	0.436728	2.164738	2.258820
C	-2.398848	3.586177	3.669353
H	-2.933799	2.698526	4.041511
H	-1.822474	3.993665	4.513606
C	-1.454165	3.241332	-2.519069
H	-0.960405	4.190751	-2.246429
C	-0.329989	2.331576	-3.021332
H	0.436777	2.164506	-2.259219
H	-0.699423	1.350344	-3.337410
C	-2.398921	3.585696	-3.669779
H	-3.148627	4.337084	-3.380753
H	-1.822592	3.993136	-4.514085
C	-2.752742	-3.227723	-0.000058
C	-4.201019	-3.653244	0.000218
C	-3.585202	-1.080170	0.000110
C	-4.222724	-0.799927	-1.227480
C	-5.479054	-0.186971	-1.203976
H	-5.990859	0.029239	-2.143000
C	-6.107010	0.112395	0.000310
H	-7.098240	0.573173	0.000393
C	-5.478901	-0.187064	1.204494
H	-5.990597	0.029076	2.143594
C	-4.222556	-0.799991	1.227802
C	-3.599083	-1.259028	-2.532613
H	-3.058011	-2.193948	-2.317055
C	-2.563640	-0.249485	-3.011704
H	-3.045964	0.709570	-3.245745
H	-1.809558	-0.063495	-2.237075
C	-4.616591	-1.567371	-3.627737
H	-5.117779	-0.657010	-3.993453
H	-4.110796	-2.024558	-4.491991
C	-3.598637	-1.258975	2.532839
H	-3.057302	-2.193727	2.317199
C	-2.563428	-0.249145	3.011842
H	-1.809481	-0.062942	2.237135
H	-3.045997	0.709773	3.245941
C	-4.615933	-1.567614	3.628076
H	-5.394545	-2.265280	3.282158
H	-4.109917	-2.024663	4.492273
Be	0.771942	1.416323	-0.000089
Be	-0.771983	-1.416422	-0.000166
N	2.420043	1.918674	-0.000129
N	0.102461	-2.890636	-0.000230
N	0.000161	-0.000109	-0.631903
C	1.829082	4.268539	-0.000679
H	2.212621	5.287339	-0.000814
C	2.752770	3.227601	-0.000297
C	4.201044	3.653140	0.000007
C	3.585270	1.080101	0.000230
C	4.223066	0.799865	-1.227245
C	5.479409	0.186971	-1.203455
H	5.991458	-0.029204	-2.142353
C	6.107076	-0.112485	0.000972
H	7.098295	-0.573286	0.001250
C	5.478705	0.186945	1.205013
H	5.990168	-0.029220	2.144236
C	4.222385	0.799954	1.228041
C	3.599617	1.258872	-2.532513
H	3.058736	2.193964	-2.317188
C	2.563959	0.249502	-3.011511
H	3.046095	-0.709637	-3.245546
H	1.809927	0.063649	-2.236804
C	4.617247	1.566745	-3.627649
H	5.118308	0.656190	-3.993059
H	4.111580	2.023757	-4.492071
C	3.598334	1.259129	2.532952
H	3.056931	2.193784	2.317084
C	2.563182	0.249283	3.012021
H	1.809321	0.063030	2.237243
H	3.045770	-0.709609	3.246178
C	4.615541	1.568092	3.628185
H	5.394137	2.265705	3.282125
H	4.109442	2.025351	4.492223
C	-1.829045	-4.268656	-0.000243
H	-2.212580	-5.287456	-0.000359
C	-0.440993	-4.097974	-0.000355
C	0.389287	-5.356978	-0.000646
C	1.518588	-2.807801	-0.000133
C	2.191891	-2.829160	1.240375
C	3.586030	-2.717850	1.208044
H	4.157926	-2.723079	2.135801
C	4.269533	-2.646744	0.000115
H	5.356031	-2.575547	0.000204
C	3.586232	-2.717760	-1.207932
H	4.158296	-2.722936	-2.135586
C	2.192094	-2.829022	-1.240517
C	1.453897	-3.241590	2.518754
H	0.960425	-4.191171	2.246143
C	0.329377	-2.332033	3.020662
H	0.698186	-1.350076	3.335159
H	-0.438033	-2.166625	2.258819
C	2.398549	-3.585666	3.669643
H	2.933202	-2.697816	4.041742
H	1.822163	-3.993195	4.513865
C	1.454282	-3.241217	-2.519066
H	0.960810	-4.190878	-2.246708
C	0.329802	-2.331569	-3.020890
H	-0.437818	-2.166575	-2.259168
H	0.698598	-1.349448	-3.334891
C	2.399078	-3.585042	-3.669916
H	3.148933	-4.336366	-3.381109
H	1.822796	-3.992415	-4.514284

H	-2.933793	2.697951	-4.041834
H	-3.148485	4.337609	3.380257
H	0.158013	2.804899	3.887301
H	5.116956	0.657992	3.994237
H	2.046767	0.607032	3.916167
H	5.396094	2.264185	-3.281802
H	2.047803	0.607429	-3.915732
H	3.148427	-4.336940	3.380769
H	-0.157579	-2.804169	3.887999
H	2.933771	-2.697121	-4.041790
H	0.157870	2.804440	-3.887766
H	-0.156879	-2.803418	-3.888538
H	-2.047489	-0.607365	-3.915953
H	-5.395344	-2.264831	-3.281724
H	-5.117327	-0.657386	3.993843
H	-2.047096	-0.606894	3.916038
H	4.273320	4.746913	0.000224
H	4.736100	3.262185	0.876761
H	4.736339	3.262567	-0.876783
H	-0.139077	5.963146	0.883293
H	-0.137304	5.964530	-0.882627
H	-1.467307	5.160074	-0.001555
H	-4.273313	-4.747016	-0.000729
H	-4.735602	-3.263329	0.877748
H	-4.736753	-3.261610	-0.875805
H	0.138316	-5.963688	-0.883734
H	1.467397	-5.160015	-0.000598
H	0.138383	-5.964087	0.882195

148

(BDI)BeN$_2$Be(BDI) withOut dispersion [side-on]

N	1.282391	-2.689919	0.536597
N	1.282557	2.689820	-0.536712
N	-0.002592	0.007498	0.633187
C	1.147778	-4.023517	0.522469
C	2.256442	-4.938415	0.994678
C	2.547107	-2.250591	1.056478
C	2.662352	-1.942956	2.433274
C	3.941861	-1.689071	2.945220
H	4.061929	-1.465597	4.006715
C	5.067101	-1.705181	2.127075
H	6.054149	-1.497458	2.548863
C	4.933685	-1.990654	0.771620
H	5.824621	-2.010079	0.141704
C	3.685190	-2.292193	0.214637
C	1.446197	-1.930166	3.355981
H	0.605990	-1.550165	2.752228
C	1.614441	-0.996074	4.556459
H	2.367095	-1.370474	5.269237
H	1.908346	0.018516	4.252904
C	1.058862	-3.327651	3.864224
H	1.898376	-3.802074	4.399073
H	0.216710	-3.251161	4.570796
C	3.566504	-2.720794	-1.244070
H	2.794341	-3.504243	-1.289765
C	3.075287	-1.579303	-2.132454
H	2.112630	-1.178431	-1.785015
H	3.795833	-0.750823	-2.140207
C	4.848729	-3.331631	-1.813888
H	5.241463	-4.142252	-1.179704
H	4.651930	-3.751056	-2.812983
C	1.148114	4.023431	-0.522449
C	2.256974	4.938192	-0.994401
C	2.547198	2.250281	-1.056573
C	2.662465	1.942388	-2.433319
C	3.941909	1.687745	-2.945047
H	4.061994	1.463952	-4.006468
C	5.067081	1.703470	-2.126782
H	6.054063	1.495143	-2.548418
C	4.933663	1.989415	-0.771440
H	5.824526	2.008643	-0.141397
C	3.685242	2.291670	-0.214697
C	1.446408	1.929991	-3.356145
H	0.606041	1.550188	-2.752488
C	1.614461	0.995950	-4.556685
H	2.367398	1.370084	-5.269308
H	1.907861	-0.018805	-4.253174
C	1.059558	3.327645	-3.864306
H	1.899269	3.801832	-4.399051
H	0.217431	3.251495	-4.570945
C	3.566685	2.720458	1.243936
H	2.792268	3.501605	1.290187
C	3.079818	1.577722	2.133088
H	2.118218	1.173497	1.786582
H	3.803031	0.751553	2.140534
C	4.847785	3.335190	1.812189
H	5.237764	4.146383	1.177034
H	4.650794	3.754795	2.811174
Be	-0.002746	-1.633132	0.018830
Be	-0.002521	1.633083	-0.018700
N	-1.287136	-2.702088	-0.475375
N	-1.286792	2.702220	0.475603
N	-0.002674	-0.007500	-0.633418
C	-0.005375	-4.666634	0.062676
H	-0.006781	-5.755030	0.079545
C	-1.155371	-4.035091	-0.420405
C	-2.263459	-4.961569	-0.870896
C	-2.546740	-2.277253	-1.019601
C	-2.648506	-2.009885	-2.405750
C	-3.923021	-1.776271	-2.939121
H	-4.031907	-1.584348	-4.008030
C	-5.057116	-1.773557	-2.133187
H	-6.040433	-1.583272	-2.571580
C	-4.936980	-2.016179	-0.768089
H	-5.834482	-2.019478	-0.147220
C	-3.693363	-2.295075	-0.188698
C	-1.423020	-2.021271	-3.315270
H	-0.585929	-1.638269	-2.709400
C	-1.571353	-1.104135	-4.531316
H	-2.320765	-1.481774	-5.245759
H	-1.858800	-0.082632	-4.244743
C	-1.043635	-3.430732	-3.795208
H	-1.881204	-3.905457	-4.332848
H	-0.191277	-3.375477	-4.491463
C	-3.588192	-2.672875	1.284826
H	-2.811146	-3.448578	1.366319
C	-3.115386	-1.496027	2.136365
H	-2.148993	-1.104242	1.789052
H	-3.839471	-0.671372	2.101190
C	-4.872749	-3.272210	1.861555
H	-5.253640	-4.106649	1.251458
H	-4.684281	-3.655861	2.876500
C	-0.004953	4.666661	-0.062612
H	-0.006238	5.755058	-0.079448
C	-1.154989	4.035215	0.420491
C	-2.263053	4.961746	0.870881

C	-2.546469	2.277478	1.019733
C	-2.648392	2.010006	2.405857
C	-3.922962	1.776234	2.939027
H	-4.031985	1.584184	4.007899
C	-5.056962	1.773482	2.132944
H	-6.040313	1.583045	2.571195
C	-4.936672	2.016295	0.767900
H	-5.834088	2.019599	0.146897
C	-3.692996	2.295373	0.188721
C	-1.422991	2.021239	3.315501
H	-0.585986	1.637729	2.709816
C	-1.571776	1.104577	4.531846
H	-2.321147	1.482714	5.246065
H	-1.859476	0.083033	4.245653
C	-1.043106	3.430699	3.795048
H	-1.880491	3.905846	4.332604
H	-0.190749	3.375319	4.491292
C	-3.587720	2.673184	-1.284786
H	-2.809405	3.447571	-1.366534
C	-3.117217	1.495541	-2.136485
H	-2.151344	1.102187	-1.789504
H	-3.842624	0.672058	-2.100921
C	-4.871542	3.274674	-1.860946
H	-5.250980	4.109491	-1.250460
H	-4.682814	3.658352	-2.875835
H	5.646792	-2.581628	-1.934390
H	0.741151	-4.000042	3.056745
H	0.665570	-0.924723	5.107743
H	-5.676704	-2.523771	1.947751
H	-2.998738	-1.805361	3.187805
H	-0.744596	-4.092471	-2.971794
H	-0.616691	-1.049314	-5.074436
H	-0.743869	4.092126	2.971461
H	-0.617192	1.049661	5.075107
H	-5.676687	2.527515	-1.947129
H	2.943412	-1.930048	-3.168998
H	-3.000529	1.804587	-3.188004
H	0.665654	0.925073	-5.108133
H	0.742013	4.000083	-3.056800
H	5.648017	2.587471	1.932487
H	2.947712	1.928343	3.169648
H	-1.881163	-5.985512	-0.968570
H	-3.085272	-4.976431	-0.138925
H	-2.705302	-4.653424	-1.826733
H	1.871222	-5.956890	1.130731
H	3.071030	-4.981813	0.255751
H	2.708588	-4.599677	1.935098
H	1.871962	5.956753	-1.130403
H	3.071420	4.981346	-0.255303
H	2.709216	4.599478	-1.934784
H	-1.880797	5.985728	0.968285
H	-3.084950	4.976385	0.139003
H	-2.704792	4.653772	1.826827

148

(BDI)MgN_2Mg(BDI) with dispersion [side-on]

N	1.486974	2.938399	0.001454
N	-3.785109	-1.106371	-0.001314
N	0.000538	-0.000582	0.635425
C	2.429447	3.861183	0.002411
C	2.032098	5.313083	0.004387
C	0.105740	3.256645	0.002751
C	-0.582073	3.313024	1.233575
C	-1.979139	3.350857	1.208252
H	-2.534147	3.379603	2.148271
C	-2.678401	3.346552	0.004030
H	-3.768405	3.345275	0.004404
C	-1.980033	3.357070	-1.200701
H	-2.535828	3.390585	-2.140096
C	-0.582998	3.319455	-1.227272
C	0.176314	3.331950	2.549152
H	1.246847	3.380450	2.300886
C	-0.033832	2.054623	3.363930
H	-1.067021	1.985374	3.734060
H	0.165765	1.162768	2.753861
C	-0.164547	4.576600	3.372212
H	-1.219321	4.571453	3.690249
H	0.454594	4.623198	4.282174
C	0.174715	3.344332	-2.543150
H	1.245043	3.398298	-2.295212
C	-0.028527	2.066527	-3.358989
H	0.174811	1.175142	-2.749399
H	-1.061223	1.992402	-3.729558
C	-0.172857	4.587860	-3.364986
H	-0.011713	5.512516	-2.789628
H	0.446270	4.638712	-4.274721
C	-4.421704	-2.287659	-0.002368
C	-5.931524	-2.286223	-0.003620
C	-4.586217	0.069026	-0.000896
C	-4.979486	0.641620	-1.229975
C	-5.740566	1.814558	-1.206573
H	-6.053616	2.273597	-2.146503
C	-6.118345	2.399868	0.000034
H	-6.714712	3.315420	0.000409
C	-5.743348	1.811758	1.206169
H	-6.058577	2.268636	2.146418
C	-4.982247	0.638829	1.228616
C	-4.585549	-0.027347	-2.534357
H	-4.442888	-1.096189	-2.314106
C	-3.244401	0.504903	-3.038905
H	-3.333484	1.563760	-3.324466
H	-2.452946	0.446620	-2.275423
C	-5.657604	0.077798	-3.616965
H	-5.784336	1.112442	-3.973067
H	-5.377993	-0.531411	-4.490263
C	-4.590863	-0.032983	2.532307
H	-4.448886	-1.101555	2.310331
C	-3.249893	0.496980	3.039705
H	-2.457680	0.440176	2.276903
H	-3.338551	1.555168	3.327875
C	-5.664229	0.071168	3.613688
H	-6.641789	-0.279608	3.248704
H	-5.386333	-0.539920	4.486218
Mg	1.739232	0.912496	-0.001500
Mg	-1.739207	-0.912429	-0.000465
N	3.785103	1.106274	-0.000114
N	-1.487181	-2.938335	-0.001204
N	-0.000742	0.000843	-0.637310
C	3.805763	3.548564	0.001743
H	4.482934	4.401671	0.002457
C	4.421634	2.287624	0.000850
C	5.931460	2.286277	0.001210
C	4.586349	-0.069038	-0.000192
C	4.981613	-0.639783	-1.229496
C	5.742800	-1.812656	-1.206655
H	6.057390	-2.270248	-2.146772

C	6.118688	-2.399760	-0.000321
H	6.715125	-3.315266	-0.000361
C	5.741725	-1.813480	1.206083
H	6.055468	-2.271740	2.146159
C	4.980526	-0.640622	1.229077
C	4.589391	0.031013	-2.533452
H	4.446960	1.099644	-2.312034
C	3.248512	-0.500038	-3.039934
H	3.337653	-1.558364	-3.327439
H	2.456595	-0.443284	-2.276817
C	5.662392	-0.073232	-3.615172
H	5.789124	-1.107495	-3.972376
H	5.383793	0.537085	-4.488019
C	4.587168	0.029189	2.533205
H	4.444625	1.097940	2.312443
C	3.246023	-0.502563	3.038299
H	2.454551	-0.444746	2.274816
H	3.335076	-1.561224	3.324578
C	5.659438	-0.075565	3.615597
H	6.637126	0.276427	3.252122
H	5.380145	0.534185	4.488618
C	-3.805875	-3.548642	-0.002454
H	-4.483075	-4.401727	-0.003304
C	-2.429560	-3.861231	-0.002011
C	-2.032158	-5.313096	-0.003004
C	-0.105911	-3.256590	-0.001524
C	0.582411	-3.317543	1.228821
C	1.979454	-3.355511	1.202758
H	2.534917	-3.387700	2.142400
C	2.678226	-3.346982	-0.001742
H	3.768234	-3.345927	-0.001760
C	1.979376	-3.352860	-1.206201
H	2.534730	-3.383009	-2.145978
C	0.582325	-3.314800	-1.232042
C	-0.175578	-3.340353	2.544566
H	-1.245983	-3.392164	2.296493
C	0.030408	-2.062858	3.360194
H	1.063251	-1.990920	3.730781
H	-0.171198	-1.171115	2.750588
C	0.169339	-4.584406	3.366771
H	1.223987	-4.576041	3.685137
H	-0.449811	-4.633613	4.276584
C	-0.175660	-3.335007	-2.547829
H	-1.246237	-3.383634	-2.299821
C	0.034372	-2.058199	-3.363479
H	-0.163862	-1.165802	-2.753740
H	1.067218	-1.989920	-3.734777
C	0.165697	-4.580107	-3.369987
H	-0.000608	-5.504151	-2.795138
H	-0.453170	-4.627376	-4.280101
H	-1.227437	4.577326	-3.683503
H	0.001776	5.500962	2.797882
H	0.633699	2.039952	4.240276
H	5.786077	-1.110025	3.972271
H	2.898790	0.061841	3.917521
H	6.639873	0.278400	-3.250798
H	2.901701	0.065430	-3.918636
H	0.006118	-5.508918	2.791776
H	-0.637578	-2.050534	4.236234
H	1.220552	-4.574948	-3.687752
H	0.639544	2.055714	-4.234985
H	-0.634042	-2.043542	-4.239155
H	-2.897040	-0.058872	-3.918484
H	-6.635306	-0.274572	-3.253905
H	-5.790454	1.105265	3.971552
H	-2.903834	-0.069263	3.918202
H	6.330452	3.307286	0.001888
H	6.321974	1.750788	0.879549
H	6.322385	1.751773	-0.877551
H	2.905493	5.976258	0.006052
H	1.411928	5.539015	-0.875812
H	1.410601	5.536209	0.884335
H	-6.330584	-3.307205	-0.005903
H	-6.322910	-1.752971	0.875710
H	-6.321502	-1.749437	-0.881394
H	-2.905543	-5.976284	-0.001410
H	-1.413223	-5.537390	-0.884489
H	-1.409405	-5.537863	0.875634

148

(BDI)MgN$_2$Mg(BDI) withOut dispersion [side-on]

N	-3.377617	1.431177	0.460686
N	3.364531	1.459895	-0.461418
N	0.000018	0.000011	-0.640183
C	-4.689259	1.200360	0.388752
C	-5.651950	2.309366	0.744576
C	-2.925445	2.716003	0.889244
C	-2.682616	3.727761	-0.072812
C	-2.177258	4.958274	0.362079
H	-1.977802	5.744709	-0.369693
C	-1.929074	5.202665	1.709819
H	-1.539602	6.172723	2.029816
C	-2.186837	4.207551	2.647596
H	-1.996678	4.406433	3.705701
C	-2.686370	2.956744	2.264637
C	-2.947186	3.491247	-1.553712
H	-3.612953	2.617747	-1.629443
C	-1.652330	3.138691	-2.295519
H	-1.163319	2.248994	-1.868954
H	-1.853940	2.931143	-3.359003
C	-3.664686	4.664087	-2.229994
H	-3.027391	5.560593	-2.291871
H	-3.944168	4.396637	-3.261490
C	-2.950550	1.896081	3.325174
H	-3.489621	1.071259	2.834535
C	-1.639434	1.318343	3.872817
H	-1.021195	0.895039	3.066980
H	-1.044602	2.094721	4.380872
C	-3.842252	2.409196	4.462157
H	-4.787012	2.830762	4.085090
H	-4.092265	1.589175	5.154233
C	4.678208	1.240630	-0.390610
C	5.630844	2.358141	-0.746905
C	2.900887	2.740906	-0.889169
C	2.649475	3.749960	0.073518
C	2.133380	4.976293	-0.360561
H	1.927287	5.760555	0.371699
C	1.882881	5.219302	-1.708120
H	1.485018	6.186153	-2.027505
C	2.149024	4.226977	-2.646516
H	1.956934	4.424821	-3.704464
C	2.659406	2.980315	-2.264377
C	2.916466	3.514800	1.554189
H	3.590555	2.647630	1.629088
C	1.625147	3.149280	2.295905
H	0.889043	3.966325	2.241520

H	1.144705	2.255139	1.868897
C	3.622829	4.693811	2.231450
H	2.976980	5.584120	2.294307
H	3.905011	4.428065	3.262637
C	2.932289	1.922448	-3.325512
H	3.479342	1.102437	-2.835638
C	1.625970	1.332916	-3.872032
H	1.012429	0.903790	-3.065688
H	1.023607	2.103977	-4.379309
C	3.818173	2.443952	-4.463226
H	4.759450	2.873980	-4.086997
H	4.074936	1.626410	-5.155760
Mg	-1.974369	-0.008094	-0.000325
Mg	1.974403	0.007848	-0.000305
N	-3.364706	-1.459787	-0.461403
N	3.377868	-1.431107	0.460552
N	0.000006	-0.000066	0.638996
C	-5.272545	-0.022516	-0.001400
H	-6.361917	-0.027212	-0.001886
C	-4.678364	-1.240425	-0.390693
C	-5.631058	-2.357909	-0.746824
C	-2.901066	-2.740922	-0.888823
C	-2.649380	-3.749623	0.074142
C	-2.133248	-4.976062	-0.359627
H	-1.926912	-5.760044	0.372866
C	-1.883063	-5.219522	-1.707150
H	-1.485162	-6.186437	-2.026293
C	-2.149601	-4.227576	-2.645851
H	-1.957823	-4.425814	-3.703779
C	-2.659981	-2.980828	-2.264032
C	-2.916244	-3.514115	1.554779
H	-3.589781	-2.646502	1.629549
C	-1.624724	-3.149424	2.296554
H	-0.889187	-3.966991	2.242296
H	-1.143622	-2.255649	1.869524
C	-3.623434	-4.692615	2.232075
H	-2.978208	-5.583377	2.294935
H	-3.905427	-4.426656	3.263259
C	-2.933178	-1.923281	-3.325400
H	-3.481086	-1.103632	-2.835880
C	-1.626975	-1.332830	-3.871244
H	-1.014072	-0.903308	-3.064615
H	-1.023860	-2.103541	-4.378162
C	-3.818055	-2.445429	-4.463579
H	-4.759106	-2.876385	-4.087839
H	-4.075240	-1.627989	-5.156075
C	5.272582	0.022840	-0.001201
H	6.361952	0.027737	-0.001645
C	4.689491	-1.200187	0.388729
C	5.652259	-2.309166	0.744359
C	2.925718	-2.716006	0.888920
C	2.682577	-3.727523	-0.073289
C	2.177221	-4.958104	0.361442
H	1.977541	-5.744350	-0.370469
C	1.929366	-5.202774	1.709179
H	1.539904	-6.172871	2.029075
C	2.187460	-4.207893	2.647130
H	1.997587	-4.407037	3.705237
C	2.686981	-2.957045	2.264343
C	2.946862	-3.490684	-1.554176
H	3.612700	-2.617230	-1.629825
C	1.651922	-3.137893	-2.295731
H	0.923611	-3.961843	-2.240602
H	1.163110	-2.248078	-1.869180
C	3.664128	-4.663406	-2.230917
H	3.026716	-5.559839	-2.292899
H	3.943390	-4.395713	-3.262395
C	2.951487	-1.896598	3.325013
H	3.491076	-1.072009	2.834555
C	1.640505	-1.318264	3.872365
H	1.022653	-0.894587	3.066421
H	1.045172	-2.094454	4.380126
C	3.842653	-2.410170	4.462198
H	4.787216	-2.832376	4.085349
H	4.093050	-1.590226	5.154224
H	-3.343509	3.193141	5.054349
H	-4.584155	4.945732	-1.693264
H	-0.924148	3.962764	-2.240581
H	-3.310694	-3.224365	-5.055066
H	-1.835079	-0.535894	-4.603636
H	-4.540280	-4.982919	1.695472
H	-1.828376	-2.943028	3.359874
H	4.583681	-4.945278	-1.694455
H	1.853369	-2.930480	-3.359272
H	3.343318	-3.193720	5.054416
H	-1.840815	0.519195	4.604678
H	1.842068	-0.519319	4.604393
H	1.828860	2.943103	3.359257
H	4.539469	4.984746	1.694836
H	3.311765	3.223484	-5.054741
H	1.833931	0.535823	-4.604295
H	-6.676147	-2.042482	-0.641847
H	-5.466276	-2.697888	-1.780082
H	-5.460189	-3.236055	-0.105880
H	-6.694168	1.984657	0.639372
H	-5.490419	2.650875	1.777837
H	-5.488772	3.188945	0.103592
H	6.675950	2.042861	-0.641673
H	5.466185	2.697747	-1.780305
H	5.459789	3.236489	-0.106298
H	6.694451	-1.984428	0.638969
H	5.490936	-2.650707	1.777644
H	5.488970	-3.188727	0.103377

148

(BDI)CaN$_2$Ca(BDI) with dispersion [side-on]

N	2.172184	-2.607134	-0.000171
N	-4.430477	0.822374	-0.000193
N	0.000360	0.000183	-0.617933
C	3.128274	-3.519586	-0.000620
C	2.741791	-4.978958	-0.001247
C	0.802202	-2.942890	-0.000160
C	0.097951	-2.994178	-1.226623
C	-1.301714	-3.001044	-1.203449
H	-1.851106	-3.027967	-2.147117
C	-2.011268	-2.989528	-0.000135
H	-3.104998	-3.013548	-0.000126
C	-1.301712	-3.000700	1.203173
H	-1.851092	-3.027374	2.146854
C	0.097953	-2.993741	1.226328
C	0.832914	-3.011540	-2.553257
H	1.908506	-2.970704	-2.323618
C	0.501818	-1.787860	-3.408095
H	-0.554861	-1.789303	-3.720437
H	0.671603	-0.864094	-2.839050
C	0.565739	-4.310150	-3.319924

H	-0.495017	-4.393323	-3.606716
H	1.163809	-4.348570	-4.244360
C	0.832964	-3.010758	2.552938
H	1.908498	-2.968511	2.323240
C	0.500329	-1.787947	3.408425
H	0.668776	-0.863694	2.839779
H	-0.556256	-1.791011	3.721070
C	0.567429	-4.310100	3.318955
H	0.817081	-5.195707	2.715167
H	1.165299	-4.348104	4.243540
C	-5.120099	1.953538	-0.000222
C	-6.626990	1.882781	0.000167
C	-5.013516	-0.450849	0.000305
C	-5.214434	-1.129072	1.230482
C	-5.593815	-2.474017	1.205939
H	-5.741954	-3.007809	2.148067
C	-5.781025	-3.149091	0.001220
H	-6.070041	-4.202475	0.001581
C	-5.594367	-2.474688	-1.203958
H	-5.742940	-3.008996	-2.145725
C	-5.215000	-1.129756	-1.229409
C	-4.982019	-0.403394	2.543500
H	-4.924932	0.669891	2.309534
C	-3.641834	-0.798669	3.170511
H	-3.619550	-1.871953	3.416400
H	-2.783911	-0.608734	2.505038
C	-6.128519	-0.603683	3.534991
H	-6.201242	-1.649114	3.874424
H	-5.978875	0.020872	4.429398
C	-4.983307	-0.404747	-2.542925
H	-4.926265	0.668667	-2.309542
C	-3.643373	-0.800115	-3.170412
H	-2.785156	-0.609621	-2.505478
H	-3.620984	-1.873529	-3.415724
C	-6.130261	-0.605718	-3.533752
H	-7.096783	-0.332759	-3.084034
H	-5.981175	0.018425	-4.428539
Ca	2.184214	-0.210696	0.000305
Ca	-2.183818	0.211000	-0.000593
N	4.430640	-0.822674	-0.000298
N	-2.172232	2.607304	-0.000789
N	0.000065	0.000250	0.617979
C	4.511164	-3.220405	-0.000636
H	5.184913	-4.077270	-0.000794
C	5.120164	-1.953875	-0.000297
C	6.627057	-1.883254	0.000265
C	5.013673	0.450567	0.000518
C	5.213722	1.128769	1.230853
C	5.593020	2.473750	1.206611
H	5.740516	3.007513	2.148861
C	5.780967	3.148871	0.002042
H	6.069914	4.202273	0.002623
C	5.595081	2.474506	-1.203285
H	5.744175	3.008877	-2.144930
C	5.215798	1.129560	-1.229042
C	4.980736	0.403022	2.543732
H	4.922868	-0.670179	2.309551
C	3.640899	0.799202	3.170924
H	3.619388	1.872476	3.416922
H	2.782701	0.609958	2.505588
C	6.127469	0.602298	3.535173
H	6.201034	1.647648	3.874674
H	5.977410	-0.022232	4.429529
C	4.984597	0.404671	-2.542697
H	4.928198	-0.668825	-2.309545
C	3.644372	0.799372	-3.169994
H	2.786321	0.608067	-2.505084
H	3.621316	1.872847	-3.414967
C	6.131336	0.606560	-3.533576
H	7.098093	0.334226	-3.083978
H	5.982600	-0.017588	-4.428416
C	-4.511286	3.220160	-0.000567
H	-5.185185	4.076908	-0.000590
C	-3.128458	3.519601	-0.000761
C	-2.742204	4.979031	-0.001034
C	-0.802273	2.943142	-0.000755
C	-0.097938	2.993876	-1.227197
C	1.301724	3.000711	-1.203943
H	1.851173	3.027216	-2.147591
C	2.011188	2.989639	-0.000575
H	3.104923	3.013620	-0.000498
C	1.301562	3.001276	1.202689
H	1.850889	3.028223	2.146397
C	-0.098114	2.994424	1.225764
C	-0.832861	3.010878	-2.553856
H	-1.908438	2.969499	-2.324222
C	-0.501121	1.787459	-3.408803
H	0.555565	1.789411	-3.721116
H	-0.670509	0.863554	-2.839867
C	-0.566329	4.309714	-3.320381
H	0.494430	4.393503	-3.606986
H	-1.164291	4.347887	-4.244899
C	-0.833189	3.011904	2.552332
H	-1.908740	2.970364	2.322612
C	-0.501454	1.788839	3.407797
H	-0.670610	0.864687	2.839193
H	0.555159	1.791081	3.720352
C	-0.566813	4.311025	3.318434
H	-0.815600	5.196838	2.714587
H	-1.164886	4.349479	4.242867
H	-0.493304	-4.394867	3.605371
H	0.813955	-5.196381	-2.716448
H	1.117209	-1.768320	-4.321639
H	6.203281	1.652249	-3.872385
H	3.456123	0.233415	-4.095088
H	7.093914	0.328679	3.085696
H	3.451475	0.232674	4.095425
H	-0.815143	5.195737	-2.716853
H	-1.116464	1.767728	-4.322376
H	0.493907	4.394961	3.605138
H	1.115892	-1.768010	4.321845
H	-1.116992	1.769363	4.321244
H	-3.452690	-0.232089	4.095037
H	-7.095217	-0.330774	3.085622
H	-6.202971	-1.651329	-3.872637
H	-3.454821	-0.233997	-4.095344
H	7.086728	-2.879382	0.000137
H	6.985309	-1.327224	-0.880499
H	6.984604	-1.327783	0.881667
H	3.619203	-5.637376	0.000191
H	2.122419	-5.210920	0.877871
H	2.125545	-5.210861	-0.882600
H	-7.086744	2.878870	-0.000003
H	-6.985064	1.326742	-0.880662
H	-6.984634	1.327255	0.881495
H	-3.619714	5.637320	-0.000650

H	-2.123974	5.211124	0.878861
H	-2.124890	5.210976	-0.881621

148

(BDI)CaN$_2$Ca(BDI) withOut dispersion [side-on]

N	4.027426	1.530167	-0.000405
N	-4.027748	1.529625	0.000499
N	-0.000036	-0.000048	0.626707
C	5.333764	1.286777	-0.000365
C	6.297798	2.453635	-0.000431
C	3.534596	2.859528	-0.000504
C	3.234044	3.504204	1.229241
C	2.637141	4.769708	1.201320
H	2.398961	5.270389	2.143799
C	2.337522	5.404020	-0.000665
H	1.869316	6.391533	-0.000727
C	2.637019	4.769498	-1.202570
H	2.398751	5.270018	-2.145113
C	3.233916	3.503987	-1.230332
C	3.523750	2.834707	2.566019
H	4.134815	1.943514	2.355645
C	2.230507	2.355384	3.239438
H	1.572380	3.204107	3.486352
H	1.648265	1.678953	2.591683
C	4.333401	3.727606	3.512978
H	3.765229	4.618145	3.826398
H	4.603607	3.175415	4.427357
C	3.523477	2.834266	-2.567028
H	4.134475	1.943049	-2.356555
C	2.230148	2.354957	-3.240289
H	1.647902	1.678696	-2.592363
H	1.572086	3.203699	-3.487317
C	4.333155	3.726958	-3.514159
H	5.265136	4.077164	-3.044419
H	4.603202	3.174637	-4.428508
C	-5.334047	1.286007	0.000730
C	-6.298310	2.452671	0.001097
C	-3.535205	2.859101	0.000661
C	-3.234860	3.503910	-1.229058
C	-2.638237	4.769547	-1.201099
H	-2.400235	5.270345	-2.143562
C	-2.338670	5.403842	0.000909
H	-1.870707	6.391471	0.001006
C	-2.637889	4.769145	1.202792
H	-2.399607	5.269629	2.145352
C	-3.234506	3.503499	1.230512
C	-3.524442	2.834401	-2.565857
H	-4.135325	1.943077	-2.355497
C	-2.231093	2.355356	-3.239274
H	-1.573096	3.204205	-3.486098
H	-1.648768	1.678965	-2.591550
C	-4.334277	3.727155	-3.512794
H	-3.766303	4.617844	-3.826150
H	-4.604337	3.174947	-4.427205
C	-3.523669	2.833565	2.567187
H	-4.134473	1.942212	2.356727
C	-2.230088	2.354505	3.240144
H	-1.647880	1.678358	2.592068
H	-1.572144	3.203369	3.487059
C	-4.333401	3.725952	3.514562
H	-5.265559	4.075970	3.045034
H	-4.603140	3.173469	4.428902
Ca	2.219570	0.000055	-0.000208
Ca	-2.219589	-0.000139	-0.000331
N	4.027759	-1.529641	0.000078
N	-4.027511	-1.530153	0.000069
N	0.000012	0.000004	-0.627104
C	5.918812	0.000469	-0.000258
H	7.008884	0.000592	-0.000340
C	5.334048	-1.285972	-0.000108
C	6.298349	-2.452605	-0.000175
C	3.535244	-2.859124	0.000182
C	3.234253	-3.503589	-1.229564
C	2.637650	-4.769239	-1.201642
H	2.399153	-5.269775	-2.144120
C	2.338743	-5.403884	0.000344
H	1.870793	-6.391519	0.000410
C	2.638603	-4.769527	1.202249
H	2.400832	-5.270279	2.144794
C	3.235206	-3.503878	1.230006
C	3.523224	-2.833793	-2.566350
H	4.133900	-1.942314	-2.356041
C	2.229549	-2.355067	-3.239363
H	1.571734	-3.204082	-3.486100
H	1.647220	-1.678892	-2.591418
C	4.333092	-3.726190	-3.513604
H	3.765325	-4.617051	-3.826846
H	4.602690	-3.173817	-4.428053
C	3.524956	-2.834226	2.566696
H	4.135910	-1.942988	2.356188
C	2.231681	-2.354932	3.240080
H	1.649427	-1.678598	2.592239
H	1.573609	-3.203672	3.487084
C	4.334721	-3.726935	3.513731
H	5.266632	-4.077178	3.043880
H	4.604901	-3.174615	4.428039
C	-5.918859	-0.000415	0.000668
H	-7.008931	-0.000501	0.000884
C	-5.333849	-1.286737	0.000398
C	-6.297917	-2.453565	0.000480
C	-3.534705	-2.859528	-0.000250
C	-3.233666	-3.504232	1.229358
C	-2.636733	-4.769718	1.201168
H	-2.398176	-5.270417	2.143544
C	-2.337541	-5.403980	-0.000948
H	-1.869298	-6.391475	-0.001220
C	-2.637485	-4.769415	-1.202720
H	-2.399522	-5.269882	-2.145368
C	-3.234433	-3.503924	-1.230213
C	-3.522854	-2.834799	2.566279
H	-4.133867	-1.943508	2.356163
C	-2.229332	-2.355695	3.239320
H	-1.571208	-3.204512	3.485924
H	-1.647270	-1.679237	2.591435
C	-4.332327	-3.727683	3.513407
H	-3.764189	-4.618359	3.826500
H	-4.602123	-3.175570	4.427954
C	-3.524420	-2.834114	-2.566774
H	-4.135500	-1.943009	-2.356071
C	-2.231324	-2.354535	-3.240293
H	-1.649021	-1.678247	-2.592445
H	-1.573184	-3.203145	-3.487557
C	-4.334178	-3.726830	-3.513816
H	-5.265970	-4.077273	-3.043879
H	-4.604564	-3.174437	-4.428021
H	3.765068	4.617541	-3.827609

H	5.265291	4.077889	3.043112
H	2.449391	1.817320	4.176055
H	3.766647	-4.617497	3.827267
H	2.450532	-1.816752	4.176636
H	5.265334	-4.075944	-3.044048
H	2.447903	-1.816893	-4.176038
H	-5.264438	-4.077750	3.043822
H	-2.447855	-1.817722	4.176072
H	-3.766009	-4.617265	-3.827535
H	2.448925	1.816714	-4.176827
H	-2.450401	-1.816225	-4.176722
H	-2.449851	1.817325	-4.175937
H	-5.266258	4.077195	-3.042930
H	-3.765479	4.616648	3.827991
H	-2.448554	1.816185	4.176710
H	7.343121	-2.118230	-0.000628
H	6.134113	-3.093230	0.879871
H	6.133463	-3.093662	-0.879782
H	7.342653	2.119515	-0.000673
H	6.132901	3.094559	-0.880131
H	6.133257	3.094308	0.879526
H	-7.343091	2.118326	0.001200
H	-6.133596	3.093350	0.881013
H	-6.133863	3.093665	-0.878640
H	-7.342761	-2.119411	0.000783
H	-6.133441	-3.094255	-0.879472
H	-6.133001	-3.094483	0.880184

148

(BDI)SrN$_2$Sr(BDI) with dispersion [side-on]

N	-2.424708	2.544595	0.000612
N	4.704339	-0.730739	-0.000519
N	-0.000132	0.000053	-0.614170
C	-3.403173	3.435515	0.000351
C	-3.046586	4.904344	0.000349
C	-1.068272	2.929530	0.000833
C	-0.364384	3.022304	-1.225609
C	1.030778	3.149410	-1.203204
H	1.576410	3.218373	-2.148410
C	1.737283	3.212394	0.001223
H	2.825697	3.341457	0.001369
C	1.030527	3.148688	1.205462
H	1.575954	3.217079	2.150828
C	-0.364636	3.021567	1.227486
C	-1.095511	2.960171	-2.553484
H	-2.164546	2.840151	-2.320648
C	-0.668830	1.748198	-3.383756
H	0.383200	1.828717	-3.701931
H	-0.758967	0.825839	-2.793129
C	-0.931589	4.258941	-3.347961
H	0.119225	4.420921	-3.637858
H	-1.529957	4.229419	-4.272531
C	-1.096093	2.958798	2.555155
H	-2.165021	2.838489	2.321971
C	-0.669229	1.746796	3.385292
H	-0.758905	0.824515	2.794462
H	0.382689	1.827571	3.703769
C	-0.932871	4.257458	3.349960
H	-1.252958	5.133686	2.766296
H	-1.531375	4.227468	4.274429
C	5.395690	-1.855523	-0.000832
C	6.902963	-1.781788	-0.001057
C	5.229826	0.561357	-0.000379
C	5.372728	1.262794	1.229313
C	5.660422	2.630907	1.203295
H	5.765672	3.173638	2.146645
C	5.806263	3.318205	-0.000046
H	6.025944	4.388201	0.000081
C	5.660335	2.631212	-1.203552
H	5.765521	3.174185	-2.146770
C	5.372641	1.263106	-1.229901
C	5.165862	0.541573	2.549420
H	5.044338	-0.526342	2.313927
C	3.882419	1.004791	3.248066
H	3.923768	2.077393	3.495371
H	2.980547	0.857978	2.630748
C	6.373854	0.680516	3.477857
H	6.528210	1.724793	3.793144
H	6.236535	0.076670	4.388507
C	5.165689	0.542218	-2.550177
H	5.044295	-0.525772	-2.314963
C	3.882110	1.005496	-3.248536
H	2.980335	0.858396	-2.631149
H	3.923311	2.078174	-3.495532
C	6.373541	0.681531	-3.478739
H	7.295707	0.344652	-2.982052
H	6.236172	0.077889	-4.389516
Sr	-2.346981	-0.025590	0.000809
Sr	2.346993	0.025597	-0.000149
N	-4.704334	0.730719	0.000044
N	2.424694	-2.544589	-0.000340
N	0.000145	-0.000019	0.614915
C	-4.784537	3.122767	-0.000037
H	-5.461552	3.977239	-0.000231
C	-5.395697	1.855496	-0.000329
C	-6.902969	1.781745	-0.001072
C	-5.229809	-0.561381	-0.000538
C	-5.372921	-1.263397	1.228798
C	-5.660599	-2.631501	1.202092
H	-5.766019	-3.174673	2.145171
C	-5.806228	-3.318233	-0.001595
H	-6.025901	-4.388230	-0.002012
C	-5.660094	-2.630671	-1.204754
H	-5.765101	-3.173205	-2.148244
C	-5.372401	-1.262554	-1.230416
C	-5.166359	-0.542772	2.549277
H	-5.044639	0.525227	2.314260
C	-3.883196	-1.006442	3.248138
H	-3.924759	-2.079136	3.495008
H	-2.981116	-0.859517	2.631148
C	-6.374665	-0.681959	3.477271
H	-6.529267	-1.726359	3.792032
H	-6.237556	-0.078540	4.388237
C	-5.165153	-0.541069	-2.550319
H	-5.043963	0.526838	-2.314631
C	-3.881293	-1.003885	-3.248467
H	-2.979726	-0.856894	-2.630755
H	-3.922274	-2.076471	-3.495899
C	-6.372692	-0.680145	-3.479322
H	-7.295068	-0.343624	-2.982783
H	-6.235118	-0.076076	-4.389785
C	4.784516	-3.122787	-0.000933
H	5.461522	-3.977267	-0.001185
C	3.403149	-3.435520	-0.000681
C	3.046545	-4.904346	-0.000799
C	1.068253	-2.929506	0.000042

C	0.364122	-3.021880	-1.226291
C	-1.031036	-3.148958	-1.203659
H	-1.576856	-3.217583	-2.148780
C	-1.737305	-3.212339	0.000883
H	-2.825720	-3.341391	0.001189
C	-1.030312	-3.149042	1.205006
H	-1.575552	-3.217762	2.150458
C	0.364858	-3.021927	1.226804
C	1.095012	-2.959370	-2.554280
H	2.164082	-2.839377	-2.321590
C	0.668143	-1.747211	-3.384184
H	-0.383944	-1.827673	-3.702183
H	0.758350	-0.824985	-2.793355
C	0.931012	-4.257956	-3.349042
H	-0.119852	-4.419932	-3.638761
H	1.529192	-4.228173	-4.273726
C	1.096555	-2.959562	2.554361
H	2.165450	-2.839253	2.321030
C	0.669912	-1.747739	3.384875
H	0.759550	-0.825322	2.794257
H	-0.381954	-1.828543	3.703522
C	0.933374	-4.258406	3.348871
H	1.253258	-5.134522	2.764927
H	1.532068	-4.228699	4.273226
H	0.117832	4.419831	3.640044
H	-1.251366	5.135190	-2.764153
H	-1.283486	1.659558	-4.293760
H	-6.526631	-1.724393	-3.794905
H	-3.714903	-0.445475	-4.182389
H	-7.296516	-0.344738	2.980233
H	-3.717281	-0.448567	4.182466
H	1.250968	-5.134321	-2.765508
H	1.282631	-1.658333	-4.294279
H	-0.117283	-4.420756	3.639133
H	-1.284097	1.657781	4.295117
H	1.284946	-1.658990	4.294613
H	3.716292	0.446523	4.182121
H	7.295914	0.343647	2.980966
H	6.527733	1.725901	-3.793798
H	3.715927	0.447481	-4.182732
H	-7.367785	2.775629	-0.001100
H	-7.257297	1.225289	-0.883177
H	-7.258146	1.224909	0.880450
H	-3.937339	5.544561	0.001076
H	-2.433002	5.150146	0.879719
H	-2.434348	5.150404	-0.879898
H	7.367768	-2.775677	-0.001287
H	7.257589	-1.225006	-0.882836
H	7.257854	-1.225286	0.880792
H	3.937291	-5.544573	-0.000764
H	2.433538	-5.150331	0.878926
H	2.433724	-5.150209	-0.880692

148

(BDI)SrN$_2$Sr(BDI) withOut dispersion [side-on]

N	2.528524	-2.593481	0.000086
N	-4.832012	0.644232	0.000218
N	0.000440	-0.000124	-0.615953
C	3.546684	-3.436645	0.000119
C	3.310452	-4.935939	-0.000363
C	1.176750	-3.003037	-0.000346
C	0.474187	-3.114678	-1.230052
C	-0.918237	-3.272917	-1.204715
H	-1.461993	-3.369884	-2.148945
C	-1.622260	-3.343414	-0.001150
H	-2.704912	-3.510295	-0.001449
C	-0.918973	-3.272667	1.202819
H	-1.463313	-3.369379	2.146732
C	0.473433	-3.114400	1.228957
C	1.201180	-3.082724	-2.566457
H	2.250152	-2.825949	-2.351210
C	0.653183	-2.010500	-3.513800
H	-0.371943	-2.242631	-3.845709
H	0.628646	-1.027285	-3.020646
C	1.195153	-4.462965	-3.238427
H	0.170205	-4.778486	-3.494370
H	1.780035	-4.445611	-4.172653
C	1.199651	-3.082213	2.565785
H	2.248595	-2.824801	2.351150
C	0.650493	-2.010490	3.513019
H	0.625601	-1.027196	3.020029
H	-0.374663	-2.243288	3.844361
C	1.194102	-4.462588	3.237481
H	1.625233	-5.234982	2.583456
H	1.778367	-4.445034	4.172090
C	-5.511660	1.785162	0.000693
C	-7.025001	1.740656	0.002182
C	-5.436544	-0.623969	0.001238
C	-5.662871	-1.307331	1.231552
C	-6.092821	-2.639357	1.204233
H	-6.267214	-3.164796	2.147755
C	-6.306976	-3.310559	0.003031
H	-6.642294	-4.350769	0.003740
C	-6.093846	-2.640660	-1.199065
H	-6.269090	-3.167084	-2.141883
C	-5.663916	-1.308653	-1.228155
C	-5.444190	-0.613117	2.569429
H	-5.185619	0.433795	2.348145
C	-4.269827	-1.221803	3.346915
H	-4.457834	-2.276671	3.604729
H	-3.327801	-1.196655	2.773688
C	-6.712205	-0.602706	3.432040
H	-7.007413	-1.619304	3.738341
H	-6.552424	-0.015611	4.350655
C	-5.446693	-0.615773	-2.566967
H	-5.186878	0.431100	-2.346917
C	-4.274188	-1.226144	-3.345934
H	-3.331251	-1.201741	-2.774160
H	-4.463662	-2.280959	-3.602888
C	-6.716145	-0.604919	-3.427468
H	-7.565953	-0.160829	-2.887457
H	-6.557286	-0.019038	-4.347021
Sr	2.384229	-0.018015	0.000540
Sr	-2.384171	0.018074	-0.002187
N	4.832044	-0.644305	0.001039
N	-2.528522	2.593510	-0.001301
N	-0.000375	0.000152	0.614616
C	4.914549	-3.060017	0.000463
H	5.614726	-3.897469	0.000431
C	5.511663	-1.785247	0.000706
C	7.025007	-1.740765	0.000684
C	5.436565	0.623896	0.001015
C	5.663089	1.308035	1.230872
C	6.092983	2.640063	1.202672
H	6.267559	3.166075	2.145842
C	6.306900	3.310522	0.001017

H	6.642178	4.350744	0.001013
C	6.093573	2.639864	-1.200626
H	6.268589	3.165730	-2.143796
C	5.663680	1.307828	-1.228837
C	5.444925	0.614536	2.569204
H	5.185601	-0.432317	2.348500
C	4.271530	1.224233	3.347352
H	4.460400	2.279043	3.604771
H	3.329088	1.199597	2.774777
C	6.713615	0.603715	3.430824
H	7.009801	1.620296	3.736237
H	6.554153	0.017288	4.349924
C	5.445979	0.614226	-2.567197
H	5.186855	-0.432691	-2.346566
C	4.272578	1.223624	-3.345585
H	3.330021	1.198767	-2.773213
H	4.461253	2.278480	-3.602961
C	6.714814	0.603718	-3.428606
H	7.565337	0.160556	-2.888952
H	6.555675	0.017169	-4.347683
C	-4.914572	3.059946	-0.000059
H	-5.614773	3.897380	0.000261
C	-3.546717	3.436626	-0.000779
C	-3.310591	4.935941	-0.000890
C	-1.176756	3.003100	-0.001497
C	-0.473911	3.114182	-1.231097
C	0.918505	3.272425	-1.205530
H	1.462489	3.368909	-2.149674
C	1.622254	3.343421	-0.001846
H	2.704912	3.510251	-0.001988
C	0.918695	3.273220	1.202006
H	1.462826	3.370357	2.146001
C	-0.473719	3.115005	1.227916
C	-1.200660	3.081705	-2.567627
H	-2.249534	2.824394	-2.352506
C	-0.651910	2.009741	-3.514825
H	0.373129	2.242425	-3.846612
H	-0.626850	1.026560	-3.021615
C	-1.195334	4.461918	-3.239652
H	-0.170515	4.778076	-3.495324
H	-1.779969	4.444170	-4.174026
C	-1.200189	3.083273	2.564615
H	-2.249157	2.826075	2.349867
C	-0.651480	2.011556	3.512112
H	-0.626869	1.028173	3.019301
H	0.373694	2.244121	3.843568
C	-1.194341	4.463756	3.236097
H	-1.625052	5.236206	2.581868
H	-1.778824	4.446527	4.170576
H	0.169192	-4.778834	3.492677
H	1.625326	-5.235785	-2.584278
H	1.276988	-1.938280	-4.419605
H	7.010742	1.620359	-3.734066
H	4.095770	0.676764	-4.285431
H	7.564080	0.160244	2.891333
H	4.094386	0.677424	4.287166
H	-1.626176	5.234475	-2.585630
H	-1.275545	1.937093	-4.420713
H	-0.169384	4.779688	3.491495
H	1.273783	-1.938037	4.419159
H	-1.274921	1.939456	4.418179
H	-4.092389	-0.674617	4.286454
H	-7.563438	-0.160193	2.892969
H	-7.012989	-1.621555	-3.732058
H	-4.097648	-0.679682	-4.286062
H	7.465433	-2.745911	0.001095
H	7.398827	-1.196457	-0.880551
H	7.398847	-1.195658	0.881413
H	3.778669	-5.394754	-0.885149
H	3.780294	-5.395658	0.883077
H	2.242941	-5.190256	0.000440
H	-7.465439	2.745796	0.001112
H	-7.399703	1.194562	-0.877562
H	-7.397944	1.197326	0.884397
H	-3.780172	5.395153	-0.884742
H	-3.779185	5.395164	0.883479
H	-2.243109	5.190365	-0.001490

148

(BDI)BaN_2Ba(BDI) with dispersion [side-on]

N	-2.740883	2.583887	0.112184
N	4.887922	-0.797393	0.102092
N	0.008546	0.247264	-0.702776
C	-3.792843	3.372237	0.007432
C	-3.612299	4.866698	-0.164189
C	-1.422612	3.055039	0.003180
C	-0.830220	3.265077	-1.264632
C	0.540996	3.550689	-1.335624
H	0.996591	3.731069	-2.314149
C	1.323253	3.644625	-0.183581
H	2.381739	3.919835	-0.249773
C	0.736008	3.419125	1.062674
H	1.348136	3.490476	1.964036
C	-0.626653	3.122189	1.177234
C	-1.639094	3.148740	-2.541749
H	-2.675421	2.924729	-2.249052
C	-1.150903	1.979776	-3.398890
H	-0.127837	2.154480	-3.770503
H	-1.131773	1.054445	-2.807486
C	-1.645991	4.457264	-3.336092
H	-0.638635	4.707201	-3.706839
H	-2.309367	4.378900	-4.212259
C	-1.263587	2.904462	2.536070
H	-2.190490	2.330539	2.358219
C	-0.381237	2.104444	3.492124
H	-0.022601	1.185429	3.006815
H	0.501012	2.679025	3.815378
C	-1.705267	4.235084	3.153894
H	-2.401895	4.767946	2.491287
H	-2.208909	4.075004	4.120973
C	5.401935	-1.995092	0.303100
C	6.836974	-2.107364	0.760952
C	5.554960	0.400631	0.363931
C	5.333025	1.066475	1.602324
C	5.827144	2.363100	1.776000
H	5.657685	2.876306	2.726644
C	6.528264	3.013833	0.763049
H	6.904105	4.028052	0.916594
C	6.746090	2.359382	-0.447282
H	7.298416	2.870486	-1.240828
C	6.273808	1.061747	-0.669022
C	4.566075	0.382447	2.721697
H	4.271091	-0.610246	2.347988
C	3.285225	1.136343	3.090999
H	3.501205	2.164347	3.422833
H	2.569437	1.205257	2.255082

C	5.450123	0.169382	3.953569
H	5.753794	1.128755	4.402621
H	4.912296	-0.404877	4.724256
C	6.512242	0.376918	-2.002695
H	6.120012	-0.646969	-1.909794
C	5.741071	1.060970	-3.135838
H	4.655877	1.075816	-2.938315
H	6.061218	2.106492	-3.270047
C	8.002051	0.281687	-2.340227
H	8.563076	-0.207738	-1.530235
H	8.157319	-0.300229	-3.262315
Ba	-2.288548	-0.103045	0.465241
Ba	2.607092	0.378394	-0.635714
N	-4.865236	0.546847	0.165930
N	2.380329	-2.423471	-0.238759
N	0.286969	0.402609	0.474215
C	-5.139029	2.927362	0.004580
H	-5.889376	3.714591	-0.084034
C	-5.632313	1.611591	0.006858
C	-7.116594	1.415805	-0.197184
C	-5.282371	-0.772479	0.010489
C	-5.456677	-1.596504	1.158425
C	-5.666005	-2.969707	0.992096
H	-5.806025	-3.600213	1.874550
C	-5.703775	-3.548398	-0.273940
H	-5.868883	-4.622493	-0.385424
C	-5.520083	-2.743854	-1.397541
H	-5.534408	-3.201083	-2.390805
C	-5.303776	-1.368228	-1.283403
C	-5.427220	-0.984923	2.547484
H	-5.131353	0.068610	2.415126
C	-4.399938	-1.656275	3.463602
H	-4.658165	-2.707440	3.667474
H	-3.388351	-1.659089	3.023614
C	-6.817209	-0.994653	3.190136
H	-7.175964	-2.024690	3.347479
H	-6.803500	-0.488133	4.168299
C	-5.040258	-0.532924	-2.524077
H	-4.971602	0.516468	-2.202065
C	-3.698362	-0.898387	-3.166073
H	-2.849507	-0.766825	-2.476966
H	-3.685860	-1.949412	-3.495763
C	-6.176800	-0.637438	-3.543243
H	-7.146153	-0.381761	-3.089841
H	-6.004924	0.047013	-4.388719
C	4.676776	-3.186848	0.122518
H	5.253277	-4.104018	0.245117
C	3.292286	-3.378184	-0.094865
C	2.846294	-4.826883	-0.115189
C	1.022199	-2.795453	-0.275297
C	0.328348	-2.826110	-1.510279
C	-1.043715	-3.105856	-1.524158
H	-1.574041	-3.128730	-2.479025
C	-1.742541	-3.385494	-0.348272
H	-2.809965	-3.624645	-0.378588
C	-1.052629	-3.358702	0.868588
H	-1.592396	-3.586777	1.793136
C	0.314504	-3.056025	0.929565
C	1.061146	-2.576552	-2.814874
H	2.092634	-2.300145	-2.541083
C	0.447859	-1.420187	-3.606193
H	-0.559720	-1.670454	-3.974796
H	0.351797	-0.528137	-2.969529
C	1.148485	-3.849977	-3.660455
H	0.145999	-4.196324	-3.959621
H	1.728902	-3.670467	-4.579571
C	1.006182	-2.966298	2.278935
H	2.085678	-2.889226	2.084867
C	0.592113	-1.684583	3.006294
H	0.765284	-0.803329	2.372488
H	-0.480813	-1.710642	3.268005
C	0.769081	-4.199138	3.152225
H	1.041807	-5.128285	2.629237
H	1.369438	-4.139748	4.073536
H	-0.836002	4.890447	3.325702
H	-1.989920	5.301206	-2.720388
H	-1.801410	1.834376	-4.275948
H	-6.261917	-1.656100	-3.954335
H	-3.495542	-0.265487	-4.043196
H	-7.550947	-0.481278	2.551867
H	-4.337832	-1.136296	4.432295
H	1.633410	-4.668211	-3.108041
H	1.064412	-1.177524	-4.486976
H	-0.286274	-4.287991	3.456523
H	-0.939869	1.835508	4.402688
H	1.152921	-1.563618	3.946617
H	2.754977	0.625843	3.908781
H	6.367002	-0.381553	3.696512
H	8.447721	1.276907	-2.496921
H	5.890441	0.535416	-4.092145
H	-7.662801	2.367576	-0.197244
H	-7.305121	0.908387	-1.156172
H	-7.532079	0.763995	0.586245
H	-4.326236	5.417302	0.465215
H	-2.591714	5.187912	0.081523
H	-3.813993	5.153390	-1.208546
H	7.157001	-3.151247	0.868501
H	7.511926	-1.598961	0.055671
H	6.962741	-1.599697	1.729982
H	3.700444	-5.510268	-0.198116
H	2.296140	-5.076026	0.804767
H	2.153534	-5.014526	-0.947742

148

(BDI)BaN$_2$Ba(BDI) withOut dispersion [side-on]

N	-2.781956	-2.539607	-0.000203
N	5.122657	0.604283	0.000118
N	-0.000062	0.000025	0.608263
C	-3.808453	-3.380427	-0.000250
C	-3.538026	-4.872733	-0.000231
C	-1.450626	-3.007861	-0.000164
C	-0.752397	-3.172270	1.228823
C	0.620281	-3.451326	1.202708
H	1.154294	-3.589979	2.147940
C	1.313738	-3.590771	-0.000175
H	2.376370	-3.857177	-0.000180
C	0.620295	-3.451206	-1.203054
H	1.154316	-3.589785	-2.148293
C	-0.752383	-3.172148	-1.229156
C	-1.461379	-3.056554	2.571638
H	-2.516026	-2.826579	2.354600
C	-0.905766	-1.909904	3.425227
H	0.125936	-2.116096	3.755078
H	-0.881620	-0.966594	2.858404
C	-1.427528	-4.375152	3.356140
H	-0.398214	-4.651627	3.637600

H	-2.011170	-4.288728	4.287076
C	-1.461407	-3.056416	-2.571948
H	-2.515974	-2.826107	-2.354866
C	-0.905519	-1.910072	-3.425769
H	-0.881018	-0.966683	-2.859092
H	0.126083	-2.116639	-3.755700
C	-1.428036	-4.375161	-3.356226
H	-1.845968	-5.210769	-2.774771
H	-2.011654	-4.288686	-4.287173
C	5.790506	1.745184	0.000174
C	7.304906	1.719411	0.000216
C	5.705197	-0.667533	0.000086
C	5.907646	-1.362335	-1.230099
C	6.302326	-2.705758	-1.201141
H	6.463181	-3.235875	-2.144576
C	6.499128	-3.382250	0.000021
H	6.807417	-4.430787	-0.000003
C	6.302366	-2.705801	1.201214
H	6.463251	-3.235954	2.144625
C	5.907686	-1.362380	1.230235
C	5.722698	-0.663310	-2.570996
H	5.419164	0.372286	-2.352423
C	4.612576	-1.302130	-3.415634
H	4.851364	-2.343974	-3.683715
H	3.643336	-1.320904	-2.889725
C	7.031242	-0.603271	-3.370266
H	7.375715	-1.608932	-3.661258
H	6.897498	-0.018472	-4.294588
C	5.722759	-0.663407	2.571162
H	5.419244	0.372203	2.352633
C	4.612625	-1.302242	3.415775
H	3.643390	-1.320983	2.889858
H	4.851398	-2.344099	3.683818
C	7.031306	-0.603426	3.370432
H	7.838916	-0.134197	2.788950
H	6.897576	-0.018658	4.294776
Ba	-2.553360	0.239570	-0.000227
Ba	2.553312	-0.239538	0.000049
N	-5.122672	-0.604333	-0.000139
N	2.781999	2.539613	0.000114
N	0.000015	0.000045	-0.608450
C	-5.177000	-3.016016	-0.000255
H	-5.870716	-3.857322	-0.000309
C	-5.790491	-1.745250	-0.000142
C	-7.304892	-1.719515	-0.000030
C	-5.705239	0.667469	0.000058
C	-5.907446	1.362566	-1.229999
C	-6.302154	2.705977	-1.200803
H	-6.462839	3.236315	-2.144144
C	-6.499207	3.382175	0.000481
H	-6.807508	4.430709	0.000650
C	-6.302680	2.705437	1.201553
H	-6.463751	3.235366	2.145057
C	-5.907994	1.362014	1.230334
C	-5.722289	0.663833	-2.571020
H	-5.418499	-0.371724	-2.352612
C	-4.612343	1.303085	-3.415562
H	-4.851441	2.344882	-3.683550
H	-3.643119	1.322118	-2.889633
C	-7.030824	0.603587	-3.370291
H	-7.375589	1.609218	-3.661047
H	-6.896925	0.019030	-4.294744
C	-5.723269	0.662750	2.571138

H	-5.419991	-0.372892	2.352442
C	-4.612973	1.301168	3.415854
H	-3.643715	1.319647	2.889974
H	-4.851445	2.343074	3.683978
C	-7.031828	0.602952	3.370398
H	-7.839574	0.134080	2.788815
H	-6.898250	0.017936	4.294607
C	5.177048	3.015967	0.000216
H	5.870786	3.857255	0.000279
C	3.808511	3.380411	0.000179
C	3.538106	4.872721	0.000247
C	1.450676	3.007881	0.000060
C	0.752405	3.172188	1.229033
C	-0.620272	3.451246	1.202895
H	-1.154319	3.589828	2.148117
C	-1.313681	3.590798	-0.000006
H	-2.376314	3.857205	-0.000031
C	-0.620193	3.451349	-1.202872
H	-1.154179	3.590010	-2.148117
C	0.752485	3.172289	-1.228947
C	1.461395	3.056468	2.571843
H	2.515976	2.826197	2.354791
C	0.905522	1.910095	3.425636
H	-0.126100	2.116618	3.755525
H	0.881073	0.966708	2.858952
C	1.427952	4.375204	3.356134
H	0.398725	4.652039	3.637550
H	2.011574	4.288751	4.287079
C	1.461527	3.056622	-2.571736
H	2.516134	2.826500	-2.354659
C	0.905827	1.910135	-3.425488
H	0.881509	0.966769	-2.858765
H	-0.125820	2.116500	-3.755398
C	1.427898	4.375312	-3.356095
H	1.845627	5.211049	-2.774680
H	2.011559	4.288904	-4.287020
H	-0.398823	-4.652055	-3.637637
H	-1.845148	-5.211017	2.774833
H	-1.515094	-1.768568	4.332923
H	-7.376008	1.608652	3.661596
H	-4.462513	0.743341	4.353835
H	-7.838279	0.133999	-2.788885
H	-4.461492	0.745505	-4.353626
H	1.845834	5.210840	2.774683
H	1.514869	1.768728	4.333315
H	0.398634	4.651970	-3.637556
H	-1.514895	-1.768690	-4.333426
H	1.515207	1.768815	-4.333151
H	4.461913	-0.744406	-4.353643
H	7.838844	-0.134048	-2.788768
H	7.375760	-1.609103	3.661388
H	4.461966	-0.744550	4.353803
H	-7.734002	-2.729619	-0.000489
H	-7.684483	-1.180040	0.881499
H	-7.684618	-1.179144	-0.880949
H	-4.466822	-5.456903	-0.000913
H	-2.943224	-5.161775	-0.879441
H	-2.944489	-5.161913	0.879796
H	7.734042	2.729503	0.000275
H	7.684529	1.179434	0.881424
H	7.684574	1.179519	-0.881024
H	4.466912	5.456877	0.000427
H	2.944079	5.161901	-0.879445

H 2.943803 5.161779 0.879794

(BDI)AeN$_2$Ae(BDI) Complexes [end-on]

148
(BDI)BeN$_2$Be(BDI) with dispersion [end-on]
N -1.10171800 -2.98722200 -0.12709600
N 3.28625000 1.42048100 -0.02520900
N -0.49623600 -0.33994000 -0.02844400
C -1.93260600 -4.02030000 -0.17799100
C -1.39075200 -5.41936600 -0.26414000
C 0.30080200 -3.22737800 -0.09659900
C 0.92569800 -3.41055200 1.15465200
C 2.31550700 -3.55734100 1.16760100
H 2.83903300 -3.69691400 2.11412800
C 3.04609100 -3.55039900 -0.01663500
H 4.12911400 -3.66063900 0.01520900
C 2.39969700 -3.43189600 -1.24235500
H 2.98742700 -3.47971600 -2.15983900
C 1.01171300 -3.27931100 -1.31142800
C 0.08131300 -3.53831200 2.41589300
H -0.83696100 -4.06483000 2.11153300
C -0.37850800 -2.19851500 2.99795000
H 0.42906800 -1.71175000 3.55932500
H -0.69850700 -1.50157000 2.20993800
C 0.74322200 -4.39369200 3.49357900
H 1.62952500 -3.89740800 3.91948100
H 0.04115300 -4.56535700 4.32418500
C 0.26287500 -3.25383200 -2.63598000
H -0.71036800 -3.73288900 -2.44550000
C -0.05160400 -1.84027300 -3.13011300
H -0.45876200 -1.21632400 -2.32179700
H 0.84961600 -1.34585700 -3.51278400
C 0.95631900 -4.06904400 -3.72557600
H 1.18722100 -5.09193200 -3.38944500
H 0.31171900 -4.13759800 -4.61557400
C 3.95320400 2.58803900 -0.04295200
C 5.45416000 2.59146400 0.06732100
C 4.10962900 0.25421900 0.08429200
C 4.70686100 -0.27941300 -1.07798600
C 5.60920200 -1.33663700 -0.93711100
H 6.08806000 -1.75765700 -1.82282100
C 5.92205600 -1.85025600 0.31959100
H 6.63893500 -2.67014800 0.41190500
C 5.32110300 -1.31859600 1.45483800
H 5.57075400 -1.72380500 2.43796600
C 4.41838700 -0.25351600 1.35955800
C 4.37806100 0.30423600 -2.43838100
H 4.15979800 1.37322400 -2.29048100
C 3.10086500 -0.33623200 -2.97671000
H 3.26704500 -1.40202900 -3.19229600
H 2.28770400 -0.27022900 -2.24024600
C 5.51947500 0.20644300 -3.44658600
H 5.72090200 -0.83564700 -3.74136100
H 5.26079100 0.75540000 -4.36501900
C 3.79872600 0.34674500 2.60471600
H 3.37434300 1.32066700 2.31919300
C 2.63916000 -0.52890800 3.07059700
H 1.90789500 -0.65608800 2.26115300
H 2.99594000 -1.52880500 3.36323000
C 4.81052200 0.59436300 3.72248700
H 5.66089300 1.20112000 3.37466900
H 4.33170000 1.12872700 4.55780500
Be -1.62146400 -1.40917900 -0.06961000
Be 1.62151800 1.40915000 -0.06936500
N -3.28620600 -1.42040200 -0.02537500
N 1.10177400 2.98723600 -0.12676600
N 0.49623900 0.33993900 -0.02856400
C -3.32281600 -3.83201300 -0.14407200
H -3.95534700 -4.71734200 -0.16959000
C -3.95316300 -2.58795900 -0.04318400
C -5.45412400 -2.59141800 0.06702400
C -4.10960000 -0.25414400 0.08414100
C -4.70677100 0.27950700 -1.07816100
C -5.60916200 1.33668500 -0.93731200
H -6.08798700 1.75771300 -1.82303500
C -5.92212700 1.85024300 0.31939000
H -6.63904000 2.67010900 0.41167500
C -5.32124500 1.31856200 1.45465900
H -5.57098700 1.72371800 2.43778500
C -4.41848300 0.25351200 1.35940500
C -4.37788200 -0.30413800 -2.43853200
H -4.15968200 -1.37313600 -2.29061100
C -3.10060800 0.33626600 -2.97675500
H -3.26674400 1.40205100 -3.19244200
H -2.28753900 0.27029800 -2.24018700
C -5.51920700 -0.20629300 -3.44683600
H -5.72057500 0.83580900 -3.74160900
H -5.26045400 -0.75524000 -4.36525500
C -3.79893300 -0.34678300 2.60460700
H -3.37458300 -1.32073400 2.31912700
C -2.63934900 0.52880000 3.07056300
H -1.90801800 0.65591800 2.26116900
H -2.99609400 1.52872300 3.36315500
C -4.81080900 -0.59431900 3.72232300
H -5.66126300 -1.20092400 3.37444400
H -4.33209900 -1.12880200 4.55762800
C 3.32284700 3.83208600 -0.14378000
H 3.95535600 4.71743000 -0.16926400
C 1.93262600 4.02033400 -0.17767800
C 1.39068400 5.41936900 -0.26375800
C -0.30076100 3.22736900 -0.09648900
C -0.92589200 3.41040900 1.15466500
C -2.31570800 3.55713700 1.16738300
H -2.83940900 3.69662800 2.11382400
C -3.04608300 3.55025100 -0.01698300
H -4.12911500 3.66043600 0.01468200
C -2.39946900 3.43186400 -1.24259300
H -2.98704300 3.47970300 -2.16017700
C -1.01146400 3.27935300 -1.31144100
C -0.08171700 3.53816000 2.41604800
H 0.83650400 4.06488400 2.11187300
C 0.37826800 2.19839600 2.99804800
H -0.42926400 1.71146300 3.55933500
H 0.69843400 1.50154200 2.21002500
C -0.74395700 4.39328500 3.49373000
H -1.63025500 3.89678700 3.91939500
H -0.04207000 4.56495100 4.32448900
C -0.26245400 3.25391300 -2.63589900
H 0.71086800 3.73273700 -2.44526700
C 0.05178500 1.84035100 -3.13016900
H 0.45874000 1.21625200 -2.32186600

H -0.84950200 1.34617100 -3.51299300
C -0.95562900 4.06942400 -3.72544700
H -1.18640600 5.09229000 -3.38916200
H -0.31091200 4.13802800 -4.61535700
H 1.89905600 -3.59957400 -4.04813000
H 1.05947000 -5.37386100 3.10422000
H -1.22046300 -2.35467000 3.69021900
H -5.21515600 0.34625000 4.12863400
H -2.12630200 0.08092100 3.93532200
H -6.45453400 -0.63023900 -3.04918500
H -2.76852100 -0.15543500 -3.90412800
H -1.06030200 5.37345700 3.10445500
H 1.22015200 2.35464100 3.69038300
H -1.89840800 3.60017000 -4.04819400
H -0.78752500 -1.87674900 -3.94833900
H 0.78778800 1.87676700 -3.94832500
H 2.76888000 0.15539500 -3.90416100
H 6.45475600 0.63040700 -3.04884400
H 5.21499800 -0.34616800 4.12875300
H 2.12601400 -0.08105100 3.93530900
H -5.84000200 -3.61752200 0.06066600
H -5.77548400 -2.09293800 0.99333300
H -5.91919700 -2.02973500 -0.75579900
H -0.73942500 -5.64170200 0.59385400
H -2.20305600 -6.15539100 -0.29626800
H -0.76510100 -5.53755800 -1.16131600
H 5.84005300 3.61756200 0.06107700
H 5.77545100 2.09290100 0.99361100
H 5.91927800 2.02984500 -0.75551900
H 2.20292900 6.15545600 -0.29591600
H 0.76493100 5.53756400 -1.16085700
H 0.73940600 5.64160900 0.59430000

148

(BDI)BeN$_2$Be(BDI) withOut dispersion [end-on]

N -1.32266000 3.16387400 -0.35079300
N -1.31624600 -3.16646600 0.35112600
N -0.00066400 0.60312400 0.00022600
C -1.20804400 4.49634900 -0.31936600
C -2.38963100 5.37312900 -0.64412400
C -2.59469400 2.64268900 -0.75974700
C -3.59668500 2.41634200 0.21338300
C -4.83454300 1.92238700 -0.21295400
H -5.61966400 1.73409500 0.52157600
C -5.09253100 1.68494300 -1.56056900
H -6.06980600 1.30734800 -1.87295500
C -4.10967100 1.94805900 -2.50861700
H -4.32619200 1.78093000 -3.56710300
C -2.85006400 2.43410400 -2.13425800
C -3.34542500 2.72836800 1.68267000
H -2.61043600 3.54857600 1.71578500
C -2.71044900 1.53614000 2.40314200
H -3.38106900 0.66429000 2.38656600
H -1.76650100 1.23671200 1.92393900
C -4.59256000 3.21545700 2.42502800
H -5.33521500 2.41278600 2.55902000
H -4.31825700 3.56693600 3.43217200
C -1.81531700 2.74608600 -3.20753500
H -0.97860500 3.26348200 -2.71492000
C -1.24679400 1.46550600 -3.82506300
H -0.81378600 0.81982400 -3.04937300
H -2.02773900 0.89165700 -4.34937100
C -2.35903700 3.68194900 -4.29432600
H -2.77558000 4.60983000 -3.87252600
H -1.55577900 3.96332100 -4.99398500
C -1.19901300 -4.49869900 0.31970000
C -2.37893700 -5.37778400 0.64430000
C -2.58933900 -2.64773300 0.75981800
C -3.59154500 -2.42323500 -0.21351300
C -4.83042000 -1.93160000 0.21256400
H -5.61571100 -1.74472900 -0.52215100
C -5.08915300 -1.69472700 1.56013000
H -6.06720800 -1.31900500 1.87232900
C -4.10601000 -1.95606400 2.50838800
H -4.32309000 -1.78942700 3.56683500
C -2.84540500 -2.43969300 2.13428000
C -3.33947700 -2.73476800 -1.68276400
H -2.60249200 -3.55317700 -1.71582000
C -2.70735500 -1.54105800 -2.40328300
H -3.38002500 -0.67078700 -2.38677600
H -1.76412700 -1.23942100 -1.92404800
C -4.58544300 -3.22495000 -2.42505300
H -5.33005600 -2.42410500 -2.55911800
H -4.31032700 -3.57588900 -3.43216400
C -1.81023900 -2.74972900 3.20771200
H -0.97256400 -3.26570500 2.71524300
C -1.24400000 -1.46808200 3.82511200
H -0.81227100 -0.82163100 3.04934500
H -2.02592600 -0.89564200 4.34950000
C -2.35241000 -3.68642700 4.29455900
H -2.76725100 -4.61510100 3.87282400
H -1.54874200 -3.96627800 4.99435500
Be -0.00226000 2.17096900 0.00029100
Be 0.00232200 -2.17099100 0.00005500
N 1.31623700 3.16647200 0.35143000
N 1.32263400 -3.16391000 -0.35115400
N 0.00059100 -0.60314800 0.00056600
C -0.00527700 5.14110300 0.00070500
H -0.00638900 6.22986800 0.00089500
C 1.19883400 4.49871000 0.32047700
C 2.37860500 5.37780700 0.64559800
C 2.58943400 2.64779300 0.75988800
C 3.59150500 2.42351000 -0.21362800
C 4.83047300 1.93188100 0.21218800
H 5.61567000 1.74517700 -0.52267000
C 5.08941600 1.69481200 1.55967700
H 6.06753000 1.31907400 1.87167600
C 4.10642600 1.95600700 2.50813200
H 4.32369800 1.78924800 3.56651700
C 2.84575600 2.43966300 2.13429500
C 3.33921000 2.73533600 -1.68277700
H 2.60207000 3.55361800 -1.71554100
C 2.70725800 1.54168100 -2.40353800
H 3.38012400 0.67155100 -2.38732500
H 1.76414600 1.23969800 -1.92429100
C 4.58499600 3.22595900 -2.42509000
H 5.32976400 2.42530500 -2.55943000
H 4.30968800 3.57707800 -3.43208700
C 1.81068500 2.74951400 3.20786900
H 0.97364700 3.26693300 2.71582400
C 1.24298300 1.46766900 3.82353200
H 0.81099700 0.82252500 3.04680600
H 2.02421200 0.89391800 4.34752000
C 2.35345400 3.68436600 4.29598200
H 2.76969500 4.61295700 3.87543000
H 1.54974700 3.96447800 4.99562700

C 0.00503400 -5.14111600 -0.00030000
H 0.00603700 -6.22988100 -0.00046900
C 1.20787200 -4.49638400 -0.32011200
C 2.38941000 -5.37315100 -0.64508300
C 2.59472000 -2.64271400 -0.75988700
C 3.59661000 -2.41648700 0.21337000
C 4.83450100 -1.92243900 -0.21276600
H 5.61954500 -1.73424900 0.52187900
C 5.09262200 -1.68481000 -1.56031700
H 6.06992500 -1.30717200 -1.87255900
C 4.10987400 -1.94784100 -2.50851100
H 4.32652000 -1.78060000 -3.56695100
C 2.85025200 -2.43398000 -2.13435000
C 3.34529000 -2.72876700 1.68258500
H 2.60992800 -3.54864100 1.71555800
C 2.71092500 -1.53641100 2.40338200
H 3.38189000 -0.66481900 2.38686500
H 1.76701400 -1.23652800 1.92439400
C 4.59228800 -3.21660600 2.42468600
H 5.33534600 -2.41432100 2.55876500
H 4.31795500 -3.56819200 3.43178500
C 1.81558800 -2.74590000 -3.20771900
H 0.97929500 -3.26422000 -2.71536300
C 1.24608200 -1.46526000 -3.82420900
H 0.81292500 -0.82034800 -3.04795200
H 2.02652400 -0.89058700 -4.34836300
C 2.35975200 -3.68062000 -4.29526700
H 2.77717000 -4.60843400 -3.87417400
H 1.55652600 -3.96218100 -4.99488300
H -3.15415300 3.20352400 -4.88836200
H -5.08831500 4.04749000 1.90016000
H -2.49471800 1.78655500 3.45455000
H 3.14854800 3.20568500 4.88983800
H 0.45228200 1.70254600 4.55408500
H 5.07860400 4.05909900 -1.89996000
H 2.49075100 1.79186000 -3.45484300
H 5.08759000 -4.04874600 1.89956100
H 2.49526500 -1.78694400 3.45477800
H 3.15430700 -3.20117800 -4.88923300
H -0.45668600 1.70200700 -4.55573600
H 0.45589500 -1.70175600 -4.55479800
H -2.49101100 -1.79100600 -3.45467700
H -5.07917600 -4.05810700 -1.90006300
H -3.14844700 -3.20933600 4.88843400
H -0.45337100 -1.70310300 4.55569900
H 2.11019700 6.43745200 0.55427800
H 2.74072300 5.19206800 1.66709700
H 3.22720500 5.16822100 -0.02225900
H -3.23737200 5.16226100 0.02443600
H -2.12312500 6.43330200 -0.55338100
H -2.75215400 5.18629200 -1.66526400
H -2.11033200 -6.43744200 0.55372000
H -2.74207400 -5.19156000 1.66533200
H -3.22692300 -5.16865000 -0.02449700
H 2.12291400 -6.43333100 -0.55438000
H 2.75177000 -5.18625200 -1.66627600
H 3.23724600 -5.16231900 0.02336400

148

(BDI)MgN$_2$Mg(BDI) with dispersion [end-on]

N -3.36872700 -1.72850800 0.35476500
N 3.73438100 -1.15800700 -0.25377300
C -4.68186300 -1.71600200 0.14278500
C -5.42658100 -3.02587200 0.09065200
C -2.68609800 -2.95439700 0.58088400
C -2.76531000 -3.58503800 1.84367200
C -2.09478300 -4.79811000 2.02629100
H -2.15302000 -5.30138900 2.99365000
C -1.34117000 -5.36718600 1.00317300
H -0.83242200 -6.32112800 1.16273700
C -1.20767200 -4.69855100 -0.20900000
H -0.57531100 -5.11886400 -0.99184100
C -1.86086400 -3.48325500 -0.43702800
C -3.46989900 -2.90965100 3.00618700
H -4.18425400 -2.18418300 2.59021200
C -2.44685500 -2.11154100 3.82464200
H -1.69575200 -2.78607200 4.26622800
H -1.90729000 -1.37697300 3.20403900
C -4.25837600 -3.87734200 3.88705700
H -3.59758500 -4.56933500 4.43259700
H -4.83647900 -3.32233800 4.64193800
C -1.61695000 -2.70959500 -1.71880400
H -2.44935200 -1.99547400 -1.82948100
C -0.30982500 -1.91021000 -1.60442700
H -0.29094200 -1.25351400 -0.71189700
H 0.54938100 -2.59258700 -1.52432700
C -1.59865400 -3.58435400 -2.97055200
H -2.50886000 -4.19827400 -3.04677400
H -1.53086800 -2.95694800 -3.87300100
C 5.01435600 -0.79028200 -0.22537500
C 6.07971600 -1.84116000 -0.42021100
C 3.33144400 -2.44681100 -0.67207800
C 3.39302900 -2.78532400 -2.04666100
C 2.91489200 -4.03556800 -2.44725100
H 2.95757000 -4.31657100 -3.50118800
C 2.36226100 -4.92448900 -1.52574800
H 1.99780400 -5.89967700 -1.85814200
C 2.25465400 -4.55712400 -0.18860700
H 1.78797300 -5.23912500 0.52400300
C 2.72045500 -3.31592400 0.25938100
C 3.84976100 -1.75615300 -3.06772200
H 4.59885800 -1.10909700 -2.59008600
C 2.67490800 -0.84823200 -3.44485600
H 1.88462400 -1.42073500 -3.95436800
H 2.22605000 -0.39024100 -2.54950800
C 4.49971000 -2.36194600 -4.30874100
H 3.77779500 -2.92672300 -4.91945900
H 4.91130600 -1.56735100 -4.94979900
C 2.57852300 -2.92824900 1.71916300
H 2.68930800 -1.83338100 1.79135400
C 1.20860200 -3.27508900 2.29740500
H 0.39474100 -2.90697600 1.65777800
H 1.07464700 -4.36075900 2.41500700
C 3.71758300 -3.51949500 2.55427700
H 4.69902200 -3.19146100 2.18064500
H 3.63292500 -3.20918100 3.60762300
Mg -2.22685600 -0.06105800 0.31053500
Mg 2.22712700 0.06101500 0.31161600
N -3.73440100 1.15811100 -0.25373000
N 3.36883500 1.72856000 0.35518800
C -5.44244700 -0.53865000 -0.04516900
H -6.52045800 -0.68926300 -0.10061100
C -5.01435600 0.79036800 -0.22539700
C -6.07977700 1.84117800 -0.42033100
C -3.33139000 2.44689700 -0.67198900
C -3.39304400 2.78558100 -2.04652200

C	-2.91467500	4.03577600	-2.44701200
H	-2.95739800	4.31690100	-3.50091800
C	-2.36180800	4.92449200	-1.52546200
H	-1.99718400	5.89964200	-1.85778500
C	-2.25416100	4.55695800	-0.18836200
H	-1.78726500	5.23877000	0.52428700
C	-2.72011400	3.31578100	0.25950200
C	-3.84993300	1.75663900	-3.06774500
H	-4.59884900	1.10937600	-2.59012300
C	-2.67508100	0.84892000	-3.44537000
H	-1.88480600	1.42166700	-3.95462900
H	-2.22617400	0.39049300	-2.55025600
C	-4.50022200	2.36274500	-4.30843300
H	-3.77849100	2.92771600	-4.91918900
H	-4.91197900	1.56832400	-4.94960400
C	-2.57805200	2.92777000	1.71916800
H	-2.68868000	1.83287400	1.79106200
C	-1.20811200	3.27464400	2.29736800
H	-0.39427400	2.90683300	1.65752700
H	-1.07427200	4.36030100	2.41525200
C	-3.71713900	3.51867200	2.55449000
H	-4.69854600	3.19058000	2.18081900
H	-3.63237800	3.20809900	3.60775200
C	5.44248400	0.53870700	-0.04502400
H	6.52048800	0.68931300	-0.10052300
C	4.68192300	1.71607400	0.14298800
C	5.42655900	3.02596900	0.09047400
C	2.68605600	2.95445500	0.58083900
C	2.76513700	3.58556100	1.84340300
C	2.09446400	4.79861400	2.02557900
H	2.15260800	5.30223800	2.99276400
C	1.34080600	5.36723100	1.00223400
H	0.83190800	6.32115500	1.16144600
C	1.20744100	4.69814200	-0.20970100
H	0.57502300	5.11807000	-0.99269800
C	1.86078300	3.48284000	-0.43728100
C	3.46973800	2.91059700	3.00616000
H	4.18433300	2.18522400	2.59043100
C	2.44677600	2.11239400	3.82462900
H	1.69546800	2.78683000	4.26600400
H	1.90742900	1.37759600	3.20412200
C	4.25784500	3.87864600	3.88695600
H	3.59681100	4.57059300	4.43225900
H	4.83596600	3.32393300	4.64203800
C	1.61684900	2.70864800	-1.71873100
H	2.44923000	1.99446200	-1.82911500
C	0.30971800	1.90933500	-1.60395700
H	0.29089900	1.25298600	-0.71115400
H	-0.54947000	2.59176600	-1.52408800
C	1.59860100	3.58287900	-2.97084900
H	2.50885000	4.19670400	-3.04731000
H	1.53078300	2.95509600	-3.87303300
H	-0.72890300	-4.25966200	-2.98026200
H	-4.96294900	-4.48442900	3.29711400
H	-2.94198100	-1.56884300	4.64612200
H	-3.69434700	4.61978800	2.51995700
H	-1.08724700	2.81200200	3.28758500
H	-5.32144700	3.04568000	-4.04149200
H	-2.99977100	0.03492600	-4.11238200
H	4.96236500	4.48577900	3.29699600
H	2.94191900	1.56993900	4.64625800
H	0.72890200	4.25824900	-2.98085000
H	-0.16192200	-1.27720800	-2.49151300
H	0.16174800	1.27598400	-2.49078300
H	2.99958700	-0.03393400	-4.11150000
H	5.32100200	-3.04496700	-4.04222600
H	3.69463500	-4.62059900	2.51946000
H	1.08793000	-2.81273000	3.28777700
H	-7.07124700	1.45822700	-0.14889200
H	-5.85470500	2.72909100	0.18834600
H	-6.11623500	2.18319900	-1.46582000
H	-6.47101700	-2.88189900	-0.21082100
H	-4.93611800	-3.70847700	-0.61998700
H	-5.40778000	-3.53345000	1.06651100
H	7.07162900	-1.45744700	-0.15152500
H	5.85601300	-2.72776900	0.19089800
H	6.11409400	-2.18561700	-1.46494900
H	6.47116800	2.88192300	-0.21036000
H	4.93641400	3.70804700	-0.62089900
H	5.40713700	3.53419700	1.06597600
N	0.60347300	-0.01765700	1.44907800
N	-0.60366600	0.01680200	1.44889900

148

(BDI)MgN_2Mg(BDI) withOut dispersion [end-on]

N	-3.84560200	-1.47740400	0.51442200
N	3.84601900	-1.47676900	-0.51459000
C	-5.14657000	-1.23501800	0.66749700
C	-6.05736000	-2.36414300	1.08690300
C	-3.32116800	-2.78108900	0.76664900
C	-2.84555100	-3.10746200	2.06090100
C	-2.26695300	-4.36620700	2.26290000
H	-1.89322500	-4.62947700	3.25596600
C	-2.15323700	-5.28689700	1.22583500
H	-1.69649200	-6.26370000	1.40474800
C	-2.62047100	-4.95423800	-0.04172700
H	-2.52279700	-5.67789600	-0.85519600
C	-3.20849500	-3.70982400	-0.29739600
C	-2.91764900	-2.11948700	3.21746400
H	-3.54583000	-1.27687800	2.88981400
C	-1.52933600	-1.55076700	3.54229100
H	-0.84492700	-2.34515800	3.88161000
H	-1.07650700	-1.06837200	2.66024400
C	-3.57649900	-2.71755400	4.46585700
H	-2.97121000	-3.52792600	4.90283700
H	-3.69632000	-1.94661100	5.24380000
C	-3.67614500	-3.37237900	-1.70662600
H	-4.26737600	-2.44579900	-1.64209800
C	-2.48101600	-3.08583900	-2.62559000
H	-1.85177400	-2.27171200	-2.22961700
H	-1.83708200	-3.97397200	-2.72981000
C	-4.58646400	-4.44973600	-2.30638000
H	-5.44547000	-4.66798500	-1.65297100
H	-4.97954600	-4.12170100	-3.28196200
C	5.14701200	-1.23417000	-0.66720500
C	6.05811100	-2.36317200	-1.08626600
C	3.32190700	-2.78058300	-0.76683300
C	2.84630800	-3.10700000	-2.06107600
C	2.26804500	-4.36589000	-2.26313000
H	1.89432400	-4.62920100	-3.25618600
C	2.15466600	-5.28667600	-1.22611600
H	1.69819900	-6.26360300	-1.40506200
C	2.62185400	-4.95395800	0.04145000
H	2.52440600	-5.67768900	0.85487700
C	3.20951600	-3.70938700	0.29717700

C	2.91804200	-2.11887800	-3.21753300
H	3.54626200	-1.27627300	-2.88993300
C	1.52959500	-1.55022200	-3.54190200
H	0.84516800	-2.34461300	-3.88118100
H	1.07689100	-1.06806100	-2.65965400
C	3.57661800	-2.71669900	-4.46618300
H	2.97129600	-3.52706400	-4.90312700
H	3.69616900	-1.94563400	-5.24404500
C	3.67691200	-3.37174900	1.70644300
H	4.26837400	-2.44532200	1.64187300
C	2.48156600	-3.08470300	2.62497800
H	1.85259700	-2.27054200	2.22865100
H	1.83740800	-3.97267500	2.72919100
C	4.58679300	-4.44916600	2.30673800
H	5.44588400	-4.66787100	1.65358900
H	4.97974700	-4.12091400	3.28229600
Mg	-2.53337900	-0.02526900	-0.05908700
Mg	2.53344000	-0.02462600	0.05774600
N	-3.91034800	1.47932400	-0.14885400
N	3.91011200	1.47999400	0.14873900
C	-5.75754000	0.02303800	0.46761100
H	-6.83456800	0.03613000	0.63202000
C	-5.20300200	1.26817300	0.10106300
C	-6.16640900	2.42618200	-0.00616700
C	-3.45070600	2.78062000	-0.51326100
C	-3.37298300	3.14021100	-1.88196100
C	-2.84713700	4.39376200	-2.21672900
H	-2.78017000	4.68260600	-3.26902400
C	-2.40542200	5.27859700	-1.23757900
H	-1.99825900	6.25303300	-1.51981400
C	-2.48340500	4.91388200	0.10294900
H	-2.12885000	5.60906600	0.86828700
C	-3.00262600	3.67329700	0.49175900
C	-3.81400000	2.18972900	-2.98698700
H	-4.35237400	1.35779500	-2.50720500
C	-2.60091700	1.58875900	-3.70995500
H	-2.01241200	2.37004800	-4.21784900
H	-1.92689500	1.07340000	-3.00542900
C	-4.78111600	2.84157000	-3.98153100
H	-4.29795600	3.64438800	-4.56111600
H	-5.14959000	2.09625100	-4.70442400
C	-3.04139200	3.30160700	1.96784500
H	-3.65415300	2.39167600	2.06016300
C	-1.63754800	2.95959200	2.48565300
H	-1.18048900	2.13572700	1.91458400
H	-0.96208100	3.82662600	2.40921100
C	-3.70035500	4.38082200	2.83428600
H	-4.70557500	4.64206600	2.46829600
H	-3.80060200	4.02997700	3.87382500
C	5.75771700	0.02397700	-0.46712100
H	6.83479000	0.03723800	-0.63121700
C	5.20289200	1.26902100	-0.10067200
C	6.16609700	2.42715100	0.00699100
C	3.45018100	2.78119400	0.51316500
C	3.37235900	3.14075900	1.88186600
C	2.84631400	4.39423100	2.21660600
H	2.77925700	4.68307000	3.26889500
C	2.40447600	5.27898700	1.23743600
H	1.99715400	6.25335900	1.51966100
C	2.48251500	4.91427200	-0.10308500
H	2.12784300	5.60938500	-0.86843300
C	3.00195100	3.67376500	-0.49186900
C	3.81333200	2.19025000	2.98688400
H	4.35231100	1.35866500	2.50718100
C	2.60015400	1.58855500	3.70910100
H	2.01108900	2.36947200	4.21691800
H	1.92667100	1.07311200	3.00412300
C	4.77965600	2.84228500	3.98206100
H	4.29582500	3.64466000	4.56170000
H	5.14818400	2.09692600	4.70488400
C	3.04076800	3.30202700	-1.96793900
H	3.65370300	2.39220400	-2.06021600
C	1.63696500	2.95973500	-2.48568100
H	1.18000900	2.13592400	-1.91443300
H	0.96137400	3.82668200	-2.40937200
C	3.69953900	4.38130000	-2.83444700
H	4.70475000	4.64266100	-2.46851700
H	3.79976700	4.03044600	-3.87398400
H	-4.04947700	-5.39686900	-2.47495300
H	-4.57298900	-3.13118900	4.24529800
H	-1.59479000	-0.79944900	4.34624100
H	-3.10605600	5.30816800	2.86143600
H	-1.67604600	2.65372200	3.54381200
H	-5.65479900	3.27942800	-3.47420400
H	-2.91899800	0.85787800	-4.47119700
H	5.65334400	3.28076000	3.47527200
H	2.91818300	0.85761000	4.47030200
H	3.10512900	5.30857400	-2.86157800
H	-2.82007500	-2.79267200	-3.63257300
H	1.67547700	2.65368100	-3.54378700
H	1.59475100	-0.79873000	-4.34571200
H	4.57320100	-3.13026300	-4.24592200
H	4.04951300	-5.39609300	2.47552900
H	2.82038500	-2.79138500	3.63199700
H	-7.19529200	2.11619300	0.21318100
H	-5.88362800	3.23187600	0.68823000
H	-6.13559600	2.86645100	-1.01423700
H	-7.10090800	-2.03458700	1.15911800
H	-5.99695500	-3.19930900	0.37286900
H	-5.74688800	-2.77146000	2.06100800
H	7.10151600	-2.03326700	-1.15894200
H	5.99824000	-3.19794400	-0.37171900
H	5.74749900	-2.77118300	-2.06003200
H	7.19505100	2.11739500	-0.21234700
H	5.88323500	3.23301600	-0.68717600
H	6.13512600	2.86710100	1.01519600
N	0.59769000	-0.03613200	-0.06879100
N	-0.59760400	-0.03635800	0.07152100

148

(BDI)CaN$_2$Ca(BDI) with dispersion [end-on]

N	3.81467200	-1.53137000	-0.11068700
N	-3.81435600	-1.53179000	0.11098200
C	4.96321500	-1.67575700	0.54786600
C	5.57946300	-3.05209500	0.64083500
C	3.12471200	-2.66123500	-0.59207500
C	3.12141000	-2.93403700	-1.98137900
C	2.41784100	-4.04530300	-2.45699100
H	2.41597900	-4.26584900	-3.52728900
C	1.73057700	-4.88075800	-1.58257700
H	1.21014400	-5.76348200	-1.96174200
C	1.69142100	-4.57425600	-0.22315500
H	1.12070500	-5.21010800	0.45377600
C	2.35247900	-3.45784100	0.29110200
C	3.82997500	-1.99423500	-2.93942400
H	4.49153200	-1.36250100	-2.32421300

C	2.81492600	-1.08327200	-3.64582300
H	2.19615500	-1.66145300	-4.34993000
H	2.07906800	-0.61909700	-2.95885600
C	4.71530600	-2.71412300	-3.95547400
H	4.12481200	-3.34585700	-4.63757700
H	5.26688600	-1.98924500	-4.57460500
C	2.17427300	-3.04416700	1.74035500
H	3.06152900	-2.46454400	2.03592400
C	0.96519700	-2.10572700	1.85697800
H	1.09736600	-1.18935000	1.26269300
H	0.04652400	-2.60493400	1.51180000
C	2.04532700	-4.22317100	2.70194900
H	2.87394700	-4.93883400	2.58417500
H	2.04963100	-3.86508000	3.74290200
C	-4.96253100	-1.67616700	-0.54834200
C	-5.57861300	-3.05253300	-0.64197900
C	-3.12427800	-2.66188000	0.59172700
C	-3.12098600	-2.93548300	1.98087100
C	-2.41744600	-4.04701400	2.45588900
H	-2.41559100	-4.26813200	3.52606700
C	-1.73026000	-4.88205000	1.58100900
H	-1.20984700	-5.76500100	1.95967700
C	-1.69119600	-4.57486400	0.22173400
H	-1.12059200	-5.21044900	-0.45555500
C	-2.35219800	-3.45813600	-0.29191500
C	-3.82963900	-1.99619500	2.93934700
H	-4.49165400	-1.36464700	2.32443800
C	-2.81474600	-1.08489200	3.64556900
H	-2.19592500	-1.66284500	4.34982700
H	-2.07880800	-0.62086400	2.95857500
C	-4.71430600	-2.71655800	3.95562200
H	-4.12332700	-3.34807500	4.63750700
H	-5.26598500	-1.99196000	4.57499100
C	-2.17405300	-3.04377200	-1.74098600
H	-3.06142400	-2.46423500	-2.03636700
C	-0.96524800	-2.10494300	-1.85716200
H	-1.09790000	-1.18876800	-1.26270300
H	-0.04647900	-2.60395600	-1.51197200
C	-2.04481100	-4.22231500	-2.70310200
H	-2.87329300	-4.93820000	-2.58569700
H	-2.04914200	-3.86374500	-3.74389000
Ca	2.63138700	0.37802900	-0.69441100
Ca	-2.63245400	0.37803000	0.69588200
N	4.19375000	1.23461000	0.74764600
N	-4.19355600	1.23466600	-0.74740700
C	5.66541700	-0.62124500	1.16402700
H	6.60878100	-0.89607100	1.63613100
C	5.29340300	0.73548400	1.28659800
C	6.20412700	1.64599100	2.07162300
C	3.79129600	2.57134100	0.91550800
C	3.10941500	2.98292700	2.08858000
C	2.63429400	4.29315900	2.15989700
H	2.10373800	4.61863400	3.05831700
C	2.79461000	5.18725900	1.10146500
H	2.40880000	6.20636400	1.17969200
C	3.42905300	4.76587800	-0.06207300
H	3.53647100	5.46056300	-0.89972700
C	3.93249600	3.46599300	-0.17618500
C	2.80465700	1.99558200	3.19870000
H	3.39398400	1.08805100	3.00199400
C	1.32663300	1.58904800	3.14163600
H	0.67583100	2.45200900	3.35669300
H	1.05709900	1.20737800	2.14441000
C	3.19268900	2.51783200	4.58218900
H	2.58648500	3.39039300	4.87374000
H	3.03245400	1.74051300	5.34585800
C	4.58853100	3.00721900	-1.46736800
H	4.96110300	1.98422600	-1.28780100
C	3.58191800	2.95846100	-2.62568400
H	2.66031800	2.39452000	-2.38325200
H	3.23191100	3.96881900	-2.88814900
C	5.80653400	3.85507200	-1.83762500
H	6.54218800	3.86109200	-1.01999800
H	6.30060600	3.46279000	-2.74065600
C	-5.66459000	-0.62160400	-1.16455900
H	-6.60773100	-0.89649900	-1.63707500
C	-5.29287000	0.73527600	-1.28682500
C	-6.20360400	1.64554100	-2.07212900
C	-3.79149200	2.57153800	-0.91519300
C	-3.11000300	2.98351300	-2.08836700
C	-2.63570600	4.29403000	-2.15976100
H	-2.10546100	4.61982400	-3.05824800
C	-2.79643600	5.18805600	-1.10131900
H	-2.41130600	6.20741100	-1.17963800
C	-3.43041700	4.76628700	0.06232400
H	-3.53815400	5.46091300	0.89998600
C	-3.93304700	3.46608800	0.17650400
C	-2.80452300	1.99623600	-3.19833000
H	-3.39382800	1.08861100	-3.00201300
C	-1.32646400	1.58994000	-3.14027800
H	-0.67570100	2.45300800	-3.35501600
H	-1.05751700	1.20843200	-2.14283900
C	-3.19172200	2.51839800	-4.58207700
H	-2.58536300	3.39094300	-4.87335200
H	-3.03099000	1.74102500	-5.34558900
C	-4.58857300	3.00692200	1.46781300
H	-4.96061400	1.98371600	1.28826200
C	-3.58172000	2.95880100	2.62595900
H	-2.65981000	2.39541000	2.38341800
H	-3.23227900	3.96939100	2.88827500
C	-5.80706100	3.85397200	1.83830400
H	-6.54285200	3.85954100	1.02079800
H	-6.30072800	3.46134300	2.74140600
H	1.10005300	-4.76736200	2.55379800
H	5.44868100	-3.35976000	-3.45007000
H	3.31168400	-0.28121600	-4.21467700
H	5.52211000	4.89983800	-2.03946100
H	4.02854700	2.51258400	-3.52852200
H	4.25036700	2.82162500	4.62282100
H	1.10929200	0.80801700	3.88804500
H	-4.24938800	2.82214400	-4.62338400
H	-1.10851600	0.80886900	-3.88646600
H	-5.52327600	4.89891300	2.04013100
H	0.81397900	-1.79232100	2.90162600
H	-4.02793300	2.51275600	3.52892500
H	-3.31163100	-0.28280700	4.21428100
H	-5.44758100	-3.36247700	3.45042900
H	-1.09943900	-4.76638700	-2.55516200
H	-0.81396500	-1.79123400	-2.90171400
H	7.14679100	1.15104800	2.33629000
H	6.42299400	2.55910400	1.49699700
H	5.71154600	1.97535800	2.99952200
H	6.57879800	-3.01782600	1.09181300
H	4.94845900	-3.72358300	1.24325500
H	5.64962800	-3.51081700	-0.35765600
H	-6.57777000	-3.01816300	-1.09334500

H	-4.94739600	-3.72389100	-1.24430600
H	-5.64918600	-3.51151100	0.35637200
H	-7.14638300	1.15057000	-2.33633900
H	-6.42221100	2.55900000	-1.49796000
H	-5.71121200	1.97432100	-3.00034500
N	-0.56207100	0.79846300	0.20123100
N	0.56107500	0.79850600	-0.19959300

148

(BDI)CaN$_2$Ca(BDI) withOut dispersion [end-on]

N	-4.53366100	-1.52010700	-0.09053200
N	4.53405900	-1.51945200	0.09052300
C	-5.78944100	-1.31332800	-0.47291200
C	-6.73387600	-2.49239600	-0.55620900
C	-4.06127500	-2.81695400	0.23063800
C	-4.06285700	-3.25140900	1.58346900
C	-3.48497100	-4.48605700	1.90020500
H	-3.48206300	-4.82651500	2.93933600
C	-2.91028700	-5.28855200	0.91901500
H	-2.46285800	-6.24952300	1.18554400
C	-2.90424000	-4.85484700	-0.40358600
H	-2.44323400	-5.48392300	-1.16980700
C	-3.46953700	-3.62917600	-0.77371500
C	-4.64413000	-2.38026600	2.68869200
H	-5.19992400	-1.56574000	2.19903800
C	-3.52903900	-1.73839000	3.52647400
H	-2.94347000	-2.50172100	4.06381500
H	-2.80503400	-1.17742500	2.90756900
C	-5.63497100	-3.13049400	3.58493500
H	-5.14546600	-3.93032500	4.16336700
H	-6.09877400	-2.44205100	4.30941000
C	-3.40497100	-3.17513700	-2.22572300
H	-4.05305700	-2.29012300	-2.31853500
C	-1.98190400	-2.74150800	-2.60585600
H	-1.60255700	-1.93395700	-1.95594100
H	-1.27356900	-3.58171300	-2.52344700
C	-3.93497000	-4.23177800	-3.20144700
H	-4.95369000	-4.55556000	-2.93745200
H	-3.96410000	-3.82862000	-4.22634900
C	5.78977300	-1.31235600	0.47295300
C	6.73449600	-2.49119000	0.55630500
C	4.06200100	-2.81641700	-0.23064800
C	4.06370600	-3.25088200	-1.58347700
C	3.48610600	-4.48566300	-1.90021400
H	3.48328800	-4.82612600	-2.93934400
C	2.91159600	-5.28828700	-0.91902700
H	2.46438900	-6.24936000	-1.18555800
C	2.90543400	-4.85457800	0.40357200
H	2.44456900	-5.48375900	1.16979100
C	3.47044100	-3.62877300	0.77370100
C	4.64478800	-2.37960900	-2.68869700
H	5.20041200	-1.56496700	-2.19904000
C	3.52956100	-1.73796200	-3.52647000
H	2.94414000	-2.50141100	-4.06380400
H	2.80545400	-1.17713900	-2.90755700
C	5.63578300	-3.12962500	-3.58494900
H	5.14643900	-3.92955000	-4.16338900
H	6.09944400	-2.44108000	-4.30941700
C	3.40575900	-3.17473700	2.22570500
H	4.05367300	-2.28959800	2.31852400
C	1.98260200	-2.74137900	2.60581100
H	1.60311000	-1.93389200	1.95590100
H	1.27442600	-3.58171500	2.52337500
C	3.93594400	-4.23126700	3.20144800
H	4.95473000	-4.55485600	2.93747100
H	3.96498200	-3.82809200	4.22634600
Ca	-2.78526700	0.01997300	0.18302300
Ca	2.78528900	0.02019000	-0.18306300
N	-4.47617800	1.49116800	-0.52118800
N	4.47579400	1.49180800	0.52122100
C	-6.33105000	-0.05305000	-0.81203800
H	-7.38167400	-0.07423400	-1.10209300
C	-5.73963800	1.23068900	-0.83878300
C	-6.63978900	2.37149500	-1.26013400
C	-3.95885700	2.81019400	-0.57094000
C	-3.32850000	3.28513200	-1.75211400
C	-2.72601200	4.54824200	-1.73356800
H	-2.23528800	4.91915700	-2.63757100
C	-2.73090000	5.34002000	-0.58887800
H	-2.25236600	6.32263100	-0.59658600
C	-3.34283900	4.86678200	0.56791500
H	-3.33845500	5.48788100	1.46787500
C	-3.95997800	3.61114400	0.60289400
C	-3.26395400	2.43860200	-3.01614400
H	-3.90924800	1.56171800	-2.85198400
C	-1.83992500	1.91969300	-3.26224500
H	-1.13803100	2.75157000	-3.43556300
H	-1.45694500	1.33995400	-2.40401000
C	-3.80128700	3.17629700	-4.24789800
H	-3.17096500	4.03887300	-4.51791000
H	-3.82377100	2.50384700	-5.12043200
C	-4.58497800	3.11034900	1.89803800
H	-5.16397100	2.20743100	1.65006100
C	-3.50477600	2.70062500	2.90926100
H	-2.80106700	1.95637000	2.49513000
H	-2.89316900	3.56549800	3.21278800
C	-5.55885400	4.11610300	2.52118600
H	-6.34022600	4.41655400	1.80646700
H	-6.05668400	3.67820100	3.40118500
C	6.33105400	-0.05194100	0.81209100
H	7.38167600	-0.07286100	1.10217200
C	5.73932000	1.23165100	0.83882000
C	6.63918100	2.37268500	1.26017100
C	3.95814300	2.81070600	0.57094700
C	3.32772900	3.28553300	1.75213400
C	2.72493200	4.54849400	1.73357200
H	2.23416400	4.91932400	2.63758700
C	2.72955900	5.34022800	0.58885100
H	2.25078100	6.32272100	0.59654600
C	3.34154800	4.86709400	-0.56795800
H	3.33695700	5.48815500	-1.46794400
C	3.95900000	3.61160900	-0.60292000
C	3.26343900	2.43902300	3.01619100
H	3.90894600	1.56229600	2.85203000
C	1.83954700	1.91976400	3.26234800
H	1.13745600	2.75146900	3.43568800
H	1.45668400	1.33992900	2.40412600
C	3.80062800	3.17688400	4.24790800
H	3.17010000	4.03930900	4.51791900
H	3.82330700	2.50446200	5.12045800
C	4.58405700	3.11093000	-1.89808100
H	5.16325400	2.20814000	-1.65011500
C	3.50390800	2.70096900	-2.90926500
H	2.80036600	1.95657200	-2.49510300
H	2.89211000	3.56571000	-3.21278100
C	5.55769800	4.11688700	-2.52127200

H 6.33903600 4.41750100 -1.80658500
H 6.05558200 3.67909100 -3.40129200
H -3.29691200 -5.12982200 -3.22391900
H -6.44124800 -3.59298500 2.99533100
H -3.94135800 -1.04151800 4.27395400
H -5.04754600 5.03189600 2.85846700
H -3.95241100 2.26799900 3.81864000
H -4.82358100 3.55166600 -4.08543300
H -1.81042500 1.26676100 -4.14977400
H 4.82282400 3.55250400 4.08540200
H 1.81024200 1.26683200 4.14988400
H 5.04618100 5.03257400 -2.85852800
H -1.94888700 -2.37416100 -3.64438700
H 3.95160400 2.26842800 -3.81865400
H 3.94173200 -1.04100800 -4.27395500
H 6.44215400 -3.59195800 -2.99535200
H 3.29805700 -5.12943300 3.22392300
H 1.94949200 -2.37404600 3.64434300
H -7.65606200 2.02718000 -1.48835600
H -6.69519400 3.13496100 -0.46870600
H -6.23242900 2.87984400 -2.14750000
H -7.73955800 -2.18861100 -0.87209700
H -6.35487400 -3.24503000 -1.26466500
H -6.80835100 -2.99943100 0.41839100
H 7.74008500 -2.18715400 0.87224800
H 6.35564300 -3.24391700 1.26474200
H 6.80915200 -2.99820800 -0.41829000
H 7.65555200 2.02863400 1.48835300
H 6.69435600 3.13618700 0.46876200
H 6.23171400 2.88090200 2.14756400
N 0.59726500 0.02688500 -0.06307800
N -0.59724400 0.02688100 0.06305500

148

(BDI)SrN_2Sr(BDI) with dispersion [end-on]

N -2.82855800 2.31488400 0.07323600
N 5.16953600 -0.63911100 -0.29133000
N -0.56758300 -0.00470600 0.20457500
C -3.74466100 3.25997900 -0.07130700
C -3.33143600 4.71467700 -0.03311500
C -1.47346000 2.64927600 0.29453000
C -0.61489700 2.92883500 -0.78988500
C 0.76079400 3.09291600 -0.54295200
H 1.43060900 3.33177700 -1.37330800
C 1.27074000 3.01312900 0.75588700
H 2.33095200 3.22802800 0.94425500
C 0.41761500 2.71638400 1.82086300
H 0.82166400 2.65274300 2.83486200
C -0.95728000 2.52993600 1.61280100
C -1.13631900 2.97412600 -2.21223000
H -2.23398700 2.92953800 -2.15588400
C -0.67705800 1.74205400 -2.99581600
H 0.41485000 1.74449000 -3.13884600
H -0.92833300 0.82337700 -2.44404100
C -0.75485400 4.26591700 -2.93771600
H 0.33471700 4.34143200 -3.08376800
H -1.22175200 4.30690700 -3.93477100
C -1.87593400 2.23062800 2.78141300
H -2.85559600 1.96768800 2.34855100
C -1.39227600 1.03985900 3.61038800
H -1.18207600 0.17738400 2.95689200
H -0.46413400 1.27218900 4.15557400
C -2.09835800 3.46915600 3.65529300
H -2.51137700 4.30114800 3.06605100
H -2.79939000 3.25361300 4.47817900
C 5.76101500 -1.79138100 -0.58667900
C 7.21278500 -1.77362200 -0.99668200
C 5.76813500 0.61934800 -0.37261100
C 6.34701500 1.20275300 0.78785400
C 6.72190000 2.54926900 0.75789200
H 7.16171600 3.00389200 1.64951600
C 6.54047500 3.32599400 -0.38566700
H 6.83529600 4.37788000 -0.38801500
C 5.97853200 2.75274100 -1.52372300
H 5.83683600 3.36457400 -2.41890700
C 5.58911700 1.40858500 -1.54319300
C 6.50722500 0.37549900 2.05052000
H 6.34548500 -0.67461200 1.76600800
C 5.43541400 0.73073100 3.08653900
H 5.49564300 1.79007700 3.38236200
H 4.41524700 0.55625600 2.70395900
C 7.90773300 0.48943500 2.65370000
H 8.11417700 1.50442800 3.02874800
H 8.02149500 -0.20311100 3.50220500
C 4.96112400 0.80351300 -2.78738400
H 4.82082500 -0.26904300 -2.58423500
C 3.58039700 1.40104100 -3.07982900
H 2.85786500 1.26193500 -2.25552200
H 3.63978000 2.48551800 -3.26346900
C 5.87662900 0.92916200 -4.00721700
H 6.86768000 0.49673800 -3.80434900
H 5.44523800 0.40327500 -4.87326100
N -5.17195800 0.62470100 -0.27212500
N 2.84028000 -2.32805600 0.04016400
N 0.58853100 0.03340100 0.27240100
C -5.12493100 3.01901500 -0.29574200
H -5.74532300 3.90913100 -0.40648900
C -5.79366000 1.79066900 -0.39489600
C -7.28080800 1.79554400 -0.64973300
C -5.78911100 -0.62667600 -0.31370100
C -6.27785300 -1.21695300 0.88468600
C -6.68128000 -2.55504900 0.86687500
H -7.05283100 -3.01562600 1.78583900
C -6.61016200 -3.31853600 -0.29795600
H -6.92641700 -4.36412400 -0.28944100
C -6.12814200 -2.74121000 -1.46950800
H -6.07090800 -3.34272500 -2.38094300
C -5.71512000 -1.40416900 -1.50257700
C -6.29777200 -0.41354400 2.17315300
H -6.17963500 0.64423400 1.89716700
C -5.10712200 -0.77751400 3.06710700
H -5.11962700 -1.84463700 3.33957400
H -4.13956900 -0.57585500 2.57748200
C -7.61457900 -0.55333800 2.93729200
H -7.76258800 -1.57459200 3.32261500
H -7.63347600 0.12808300 3.80192600
C -5.17977200 -0.79134300 -2.78513200
H -4.97178100 0.26739100 -2.56540200
C -3.86448300 -1.44273500 -3.22697200
H -3.07010000 -1.38687600 -2.46091900
H -3.99837100 -2.51219700 -3.45395700
C -6.21414300 -0.83565300 -3.91220100
H -7.15442900 -0.35548000 -3.60327400
H -5.84061200 -0.31174600 -4.80597500
C 5.09281400 -3.02200200 -0.52948300
H 5.69382600 -3.90356100 -0.75696700

C	3.72593100	-3.27077200	-0.23759900
C	3.29868200	-4.72067800	-0.28784600
C	1.48460300	-2.62613500	0.29175500
C	0.57940000	-2.87908200	-0.75704100
C	-0.79349700	-2.99830300	-0.46238200
H	-1.49599800	-3.23429500	-1.26719500
C	-1.25207200	-2.90072300	0.86069800
H	-2.30734600	-3.09386100	1.09616500
C	-0.35155800	-2.63616900	1.89177000
H	-0.71846100	-2.56613600	2.91924300
C	1.01806100	-2.48408800	1.63484500
C	1.04287100	-2.91224700	-2.19973900
H	2.14211500	-2.89040600	-2.18940500
C	0.58028600	-1.65312900	-2.93705800
H	-0.51402600	-1.64083100	-3.05707800
H	0.85868900	-0.75462000	-2.36598600
C	0.60342400	-4.18067800	-2.93267100
H	-0.49280800	-4.23044100	-3.03345400
H	1.02640300	-4.21088300	-3.94954900
C	2.00196800	-2.27697500	2.77141400
H	2.96668300	-2.00501800	2.30758400
C	1.59636300	-1.14728600	3.71824200
H	1.38524600	-0.22483000	3.15194000
H	0.68108800	-1.39224000	4.27955000
C	2.25192900	-3.58371800	3.53257900
H	2.60966100	-4.37123000	2.85312900
H	3.00573000	-3.44710700	4.32530500
H	-1.15091100	3.81314600	4.10122800
H	-1.07418900	5.15499000	-2.37315200
H	-1.14584500	1.70253700	-3.99251500
H	-6.44952900	-1.87097900	-4.20636500
H	-3.46120700	-0.95292000	-4.12640000
H	-8.47384100	-0.31192100	2.29370000
H	-5.10912500	-0.18542400	3.99529900
H	0.92707100	-5.08725800	-2.39948900
H	1.02822400	-1.59104200	-3.94200700
H	1.32391500	-3.94522900	4.00459300
H	-2.14574600	0.74998500	4.36071700
H	2.38854400	-0.94764600	4.45792300
H	5.53569200	0.11454400	3.99352800
H	8.68002100	0.24597400	1.90869500
H	6.02552500	1.98137400	-4.29783200
H	3.12480600	0.93071600	-3.96449100
H	-7.67625100	2.81254700	-0.76492800
H	-7.51411900	1.21962100	-1.55925300
H	-7.81844800	1.30149600	0.17537100
H	-2.99996400	5.04108700	-1.03239900
H	-4.16860100	5.35910100	0.26620600
H	-2.48617600	4.87409100	0.65026700
H	7.59508900	-2.78062700	-1.20610800
H	7.34821100	-1.15209200	-1.89611700
H	7.83338600	-1.31684400	-0.20912000
H	4.11123100	-5.38051100	0.04648100
H	2.40645900	-4.89873100	0.32778900
H	3.04457900	-5.01061800	-1.32046900
Sr	-2.90462300	-0.22258200	0.07299400
Sr	2.93721800	0.17722400	0.28782300

148

(BDI)SrN_2Sr(BDI) withOut dispersion [end-on]

N	4.36408300	1.51199300	-0.73346700
N	-4.36519300	1.51834000	0.72800100
N	0.58910500	-0.00000100	0.11203000
C	5.40066500	1.28091600	-1.53790900
C	6.18300400	2.45314500	-2.08958000
C	3.98810100	2.81499800	-0.34264600
C	4.39377000	3.30185600	0.93312700
C	3.91803200	4.54335000	1.37624400
H	4.23088400	4.92123500	2.35423700
C	3.06461600	5.31125800	0.59194600
H	2.70608800	6.27940000	0.95091000
C	2.66775100	4.83217300	-0.65515600
H	1.98560200	5.43222400	-1.26373300
C	3.10555000	3.59626500	-1.14074900
C	5.34717800	2.51016500	1.81983700
H	5.57873200	1.57454300	1.28344800
C	4.71440200	2.14333100	3.16951300
H	4.49673700	3.03864100	3.77315700
H	3.75005700	1.61503600	3.05670600
C	6.67910300	3.24013700	2.03526500
H	6.53600500	4.18863000	2.57777300
H	7.37693800	2.62154100	2.62274500
C	2.59414700	3.09658500	-2.48436100
H	3.13249100	2.16303300	-2.70783900
C	1.09981200	2.75355400	-2.41006900
H	0.89384700	2.03474800	-1.60127000
H	0.49167400	3.65272400	-2.21900800
C	2.87249500	4.08185400	-3.62573100
H	3.94019600	4.34177000	-3.69483100
H	2.56833000	3.64828300	-4.59208400
C	-5.41171600	1.28968600	1.52009700
C	-6.19767600	2.46370300	2.06277200
C	-3.98193700	2.82047300	0.34137100
C	-4.37082600	3.30743100	-0.93958600
C	-3.88828800	4.54836700	-1.37687900
H	-4.18844500	4.92641900	-2.35877600
C	-3.04400600	5.31550700	-0.58203100
H	-2.68010000	6.28328900	-0.93652400
C	-2.66307500	4.83603200	0.66990200
H	-1.98780000	5.43535100	1.28680600
C	-3.10825600	3.60070200	1.15017700
C	-5.31364300	2.51646900	-1.83820900
H	-5.55166700	1.58046400	-1.30526400
C	-4.66495200	2.15073700	-3.18063800
H	-4.44102200	3.04651400	-3.78129000
H	-3.70151800	1.62320100	-3.05700100
C	-6.64297400	3.24655900	-2.06871000
H	-6.49337800	4.19565300	-2.60840500
H	-7.33370300	2.62859500	-2.66518500
C	-2.61360900	3.10024400	2.49975600
H	-3.15671000	2.16805500	2.71728300
C	-1.11933800	2.75356300	2.44260600
H	-0.90592800	2.03380800	1.63653800
H	-0.50706700	3.65128600	2.25797300
C	-2.90263400	4.08642900	3.63765800
H	-3.97040500	4.34908100	3.69434300
H	-2.61077600	3.65223900	4.60751700
N	4.36007900	-1.51788200	-0.73406800
N	-4.36928400	-1.51208500	0.72769200
N	-0.58289900	0.00017100	-0.08041100
C	5.84935400	-0.00469100	-1.91507700
H	6.71221800	-0.00570300	-2.58218600
C	5.39733300	-1.28928900	-1.53829700
C	6.17671200	-2.46334400	-2.09027400
C	3.98096700	-2.81999400	-0.34328200
C	3.09683400	-3.59930800	-1.14153600

C	2.65635600	-4.83430600	-0.65607200
H	1.97301500	-5.43286900	-1.26477400
C	3.05201900	-5.31430700	0.59107300
H	2.69140700	-6.28172100	0.94991200
C	3.90689500	-4.54823200	1.37556300
H	4.21877200	-4.92682300	2.35359500
C	4.38534000	-3.30772700	0.93257800
C	2.58654700	-3.09838300	-2.48510500
H	3.12692200	-2.16597300	-2.70846100
C	1.09296400	-2.75207400	-2.41071700
H	0.48288500	-3.64995300	-2.21976100
H	0.88867900	-2.03285000	-1.60185100
C	2.86272900	-4.08412400	-3.62658800
H	2.30020400	-5.02342800	-3.50079100
H	2.55949600	-3.64977200	-4.59288500
C	5.34030900	-2.51813300	1.81949100
H	5.57358700	-1.58274800	1.28341600
C	4.70841900	-2.15066000	3.16941300
H	3.74498800	-1.62062000	3.05698600
H	4.48927400	-3.04580300	3.77276700
C	6.67092200	-3.25063700	2.03445000
H	7.15486000	-3.48715100	1.07551200
H	7.36995800	-2.63359300	2.62213600
C	-5.86791400	0.00510700	1.89186300
H	-6.73847200	0.00622700	2.54889700
C	-5.41503800	-1.28060700	1.52008400
C	-6.20407100	-2.45257400	2.06272100
C	-3.98959700	-2.81550700	0.34167400
C	-3.11728000	-3.59725900	1.15047500
C	-2.67552700	-4.83410400	0.67088800
H	-2.00131100	-5.43461400	1.28779500
C	-3.05862800	-5.31365500	-0.58033000
H	-2.69740300	-6.28263000	-0.93431100
C	-3.90165500	-4.54508400	-1.37514400
H	-4.20350500	-4.92318400	-2.35650200
C	-4.38074400	-3.30259400	-0.93851200
C	-2.62057200	-3.09706200	2.49940400
H	-3.16116500	-2.16332900	2.71655400
C	-1.12543700	-2.75433800	2.44121700
H	-0.51560500	-3.65376600	2.25681900
H	-0.91049900	-2.03568700	1.63457800
C	-2.91152100	-4.08163200	3.63821600
H	-2.34979200	-5.02228300	3.51910900
H	-2.61795600	-3.64747600	4.60757300
C	-5.32195400	-2.50983100	-1.83721300
H	-5.55833200	-1.57345700	-1.30422700
C	-4.67206900	-2.14504900	-3.17932100
H	-3.70784300	-1.61908900	-3.05515500
H	-4.44929800	-3.04115900	-3.77991300
C	-6.65251100	-3.23746200	-2.06833600
H	-7.14908700	-3.47190600	-1.11536800
H	-7.34196800	-2.61799700	-2.66472200
H	2.31203100	5.02237400	-3.49982000
H	7.16358200	3.47618900	1.07648300
H	5.38263700	1.49870900	3.76268400
H	6.52610800	-4.19910600	2.57654500
H	5.37791100	-1.50746000	3.76270800
H	3.92985700	-4.34637800	-3.69574200
H	0.75029200	-2.31218100	-3.36224700
H	-3.97995200	-4.34139900	3.69574000
H	-0.79186200	-2.31512600	3.39624900
H	-6.50458000	-4.18660300	-2.60840400
H	0.75618200	2.31447800	-3.36162400
H	-5.33162200	-1.49917500	-3.78079600
H	-5.32586300	1.50602100	-3.78186000
H	-7.13887600	3.48151000	-1.11552100
H	-2.33834800	5.02549900	3.51816900
H	-0.78745800	2.31413200	3.39813000
H	7.05878700	-2.13559100	-2.65493300
H	6.50635500	-3.13346500	-1.28126500
H	5.54828800	-3.07400800	-2.75743200
H	6.51493900	3.12182600	-1.28031200
H	7.06387400	2.12331000	-2.65490600
H	5.55592100	3.06598000	-2.75600100
H	-7.08698500	2.13592900	2.61595400
H	-5.57758900	3.07368400	2.73831700
H	-6.51669300	3.13445200	1.25004400
H	-7.09072300	-2.12241000	2.61872900
H	-6.52779100	-3.12055100	1.24954700
H	-5.58461000	-3.06627000	2.73543400
Sr	2.92568800	-0.00130800	0.59123400
Sr	-2.91535600	0.00140600	-0.57973800

148

(BDI)BaN$_2$Ba(BDI) with dispersion [end-on]

N	3.07462000	-2.28096900	0.17336200
N	-5.42102500	0.53944800	-0.22239000
N	0.56355700	0.08699200	-0.18424300
C	3.97334000	-3.17914600	0.54799000
C	3.54326400	-4.59835600	0.85321100
C	1.70399400	-2.59606600	0.12511000
C	1.08831200	-2.72414400	-1.15707900
C	-0.29075500	-2.95711800	-1.22467700
H	-0.76665900	-3.09366000	-2.19909600
C	-1.06356000	-3.05069200	-0.07031700
H	-2.12592700	-3.31288800	-0.14929400
C	-0.46157800	-2.87546100	1.18557600
H	-1.06305300	-2.96053400	2.09638200
C	0.91974300	-2.65187500	1.29787300
C	1.93620800	-2.73046800	-2.41590200
H	2.92763300	-2.33357900	-2.13247500
C	1.36577700	-1.85036600	-3.52754100
H	0.40635100	-2.23760600	-3.90516100
H	1.16767500	-0.83150700	-3.15832700
C	2.18432800	-4.16109500	-2.90610200
H	1.23506600	-4.64908400	-3.18058300
H	2.84226100	-4.17223100	-3.79047500
C	1.53045600	-2.37379800	2.65765400
H	2.61793600	-2.29305600	2.51368500
C	1.04577300	-1.02439500	3.19697000
H	1.16164000	-0.23389200	2.43824700
H	-0.02019300	-1.06901400	3.46920400
C	1.26873000	-3.49310000	3.66680800
H	1.60357400	-4.46902100	3.28534100
H	1.79592600	-3.29627500	4.61397200
C	-6.01570700	1.61619700	-0.71626600
C	-7.45566600	1.51106700	-1.15792600
C	-5.97269800	-0.74114500	-0.21449700
C	-6.58847500	-1.24494900	0.96393800
C	-6.91895900	-2.60225300	1.03532500
H	-7.39123100	-2.99125500	1.94178000
C	-6.65825200	-3.46874400	-0.02464100
H	-6.92066100	-4.52652900	0.05116600
C	-6.06085300	-2.97463100	-1.18197300
H	-5.85912200	-3.65492300	-2.01413500
C	-5.71155700	-1.62456000	-1.30181000

C	-6.87568200	-0.31908900	2.13133900
H	-6.58770400	0.69258500	1.80770200
C	-6.02488500	-0.66747700	3.35614400
H	-6.23493900	-1.68570700	3.72071200
H	-4.94651700	-0.61915000	3.12663600
C	-8.36378400	-0.29605400	2.48833500
H	-8.70780700	-1.27370100	2.86215300
H	-8.56579900	0.44942500	3.27355500
C	-5.06547300	-1.11158300	-2.57890100
H	-4.88088800	-0.03556700	-2.43706100
C	-3.71519900	-1.77853100	-2.85917400
H	-2.96985300	-1.60031400	-2.06554300
H	-3.81473800	-2.87023000	-2.96678600
C	-6.00347900	-1.26614000	-3.77951600
H	-6.97427400	-0.78456100	-3.59126000
H	-5.56424900	-0.80724000	-4.67917400
N	5.43148700	-0.57227100	0.23820600
N	-3.08553800	2.29837400	-0.25313800
N	-0.53520400	-0.02143300	0.15304100
C	5.35072600	-2.91214800	0.73520000
H	5.95691500	-3.76492400	1.04542900
C	6.02077700	-1.68034500	0.65537500
C	7.48577500	-1.65108400	1.02894100
C	5.96211700	0.71067200	0.28668400
C	5.49911200	1.58140400	1.31928900
C	5.84186300	2.93789700	1.27033400
H	5.49721300	3.60881700	2.06026400
C	6.61731600	3.44757400	0.23111200
H	6.87630300	4.50871300	0.20922500
C	7.05302000	2.59823000	-0.78447500
H	7.65074800	3.00612100	-1.60432800
C	6.73174000	1.23671900	-0.78307700
C	4.68730500	1.00980400	2.47049300
H	4.17312000	0.10625500	2.09235400
C	3.62954000	1.96719900	3.01500900
H	4.07904800	2.84358700	3.50736000
H	2.95961800	2.33606700	2.22286600
C	5.60911700	0.51054000	3.58739900
H	6.19486600	1.34335500	4.00864700
H	5.02432000	0.05281200	4.40078100
C	7.16244000	0.34058600	-1.92776600
H	6.85503900	-0.68186600	-1.66350100
C	6.43371200	0.70840100	-3.22380900
H	5.33955100	0.64338000	-3.09778300
H	6.66800100	1.73662500	-3.54273000
C	8.67951500	0.34477200	-2.12389600
H	9.19971400	0.07995000	-1.19141200
H	8.97555600	-0.37945500	-2.89912100
C	-5.37099200	2.85563000	-0.84689800
H	-5.99156600	3.67996600	-1.20155800
C	-4.00119300	3.16430200	-0.65623900
C	-3.60755700	4.58740700	-0.99006600
C	-1.72157300	2.63829600	-0.19359400
C	-0.92386000	2.69714400	-1.35726600
C	0.45324800	2.93542500	-1.22996100
H	1.06488800	3.01828200	-2.13399100
C	1.03804000	3.12875300	0.03145100
H	2.09665300	3.40243100	0.11899400
C	0.25195600	3.03729800	1.17637500
H	0.71159500	3.18950400	2.15597100
C	-1.12248800	2.78279000	1.09483800
C	-1.51389800	2.40394900	-2.72278200
H	-2.60063600	2.29851100	-2.59077700
C	-0.99156100	1.06544000	-3.25280200
H	0.07370600	1.13827900	-3.52156600
H	-1.08808800	0.27747600	-2.48948900
C	-1.26610100	3.52841900	-3.72974300
H	-0.19238500	3.64198400	-3.95078400
H	-1.77604800	3.31830900	-4.68350700
C	-1.98416000	2.78259700	2.34436000
H	-2.95894400	2.34477800	2.06127400
C	-1.39291100	1.94846200	3.48083600
H	-1.13816100	0.93439200	3.13265700
H	-0.46157400	2.38903900	3.86993000
C	-2.29438400	4.21248800	2.80014600
H	-2.78347800	4.78150600	1.99643500
H	-2.96147200	4.21708900	3.67770100
H	0.19567600	-3.58174700	3.90206800
H	2.65804700	-4.76788400	-2.12102100
H	2.05523000	-1.79776600	-4.38540700
H	9.04595900	1.33478800	-2.43921400
H	6.71206100	0.02731500	-4.04352100
H	6.31285300	-0.24421700	3.21121400
H	3.00260500	1.45724900	3.76058600
H	-1.62868900	4.49640600	-3.35367000
H	-1.54053800	0.75683300	-4.15695600
H	-1.36769400	4.74339600	3.07185800
H	1.60647500	-0.73776300	4.10121700
H	-2.09530500	1.87608500	4.32658700
H	-6.21301200	0.03435300	4.18385900
H	-8.97794200	-0.04233000	1.61145500
H	-6.19735700	-2.32706300	-4.00520700
H	-3.27115700	-1.38719100	-3.78689000
H	7.73699900	-2.43573800	1.75517800
H	8.10663900	-1.81614000	0.13243500
H	7.77242800	-0.67256200	1.44081500
H	2.60705700	-4.85938700	0.34196800
H	4.32738000	-5.31244700	0.56525700
H	3.37042100	-4.72195800	1.93435200
H	-7.84138900	2.46442300	-1.54060600
H	-7.55850200	0.74936700	-1.94626600
H	-8.09357000	1.18068200	-0.32305600
H	-4.39475200	5.28927100	-0.68048200
H	-2.65938400	4.87028900	-0.51380000
H	-3.47721100	4.70418100	-2.07786200
Ba	-3.03085600	-0.24809700	0.57781400
Ba	3.06697000	0.29897500	-0.57409600

148

(BDI)BaN_2Ba(BDI) withOut dispersion [end-on]

N	-4.50469300	1.49698100	0.86503500
N	4.50435900	1.49720400	-0.86507500
N	-0.57951200	-0.00005700	-0.10414900
C	-5.42355700	1.28163700	1.79713300
C	-6.10515700	2.44980700	2.48315300
C	-4.14094000	2.76158300	0.37541900
C	-4.65469900	3.17900800	-0.88993300
C	-4.16582000	4.35841500	-1.47156200
H	-4.56286500	4.68704800	-2.43612900
C	-3.19932300	5.13091100	-0.83608500
H	-2.83324300	6.04913700	-1.30265700
C	-2.70387700	4.72493400	0.40144000
H	-1.94035600	5.33201800	0.89575100
C	-3.14653900	3.55060100	1.02095500
C	-5.78632700	2.41675200	-1.56972700
H	-5.86281600	1.43876000	-1.06384000

C	-5.54324300	2.16650600	-3.06259300
H	-5.53721300	3.10247500	-3.64311200
H	-4.57416000	1.67296000	-3.25410000
C	-7.13396300	3.12212000	-1.35853500
H	-7.13459300	4.12081600	-1.82503400
H	-7.95894300	2.54060100	-1.80171100
C	-2.55472600	3.14502700	2.36301200
H	-3.04290500	2.20358900	2.65777400
C	-1.05026700	2.86429700	2.25990200
H	-0.83728400	2.10680600	1.48957300
H	-0.48419500	3.77367400	2.00058800
C	-2.84370000	4.18582700	3.45267400
H	-3.91995800	4.39619100	3.54264500
H	-2.48381200	3.83197600	4.43252500
C	5.42312000	1.28205500	-1.79731700
C	6.10461100	2.45033300	-2.48328400
C	4.14042600	2.76174100	-0.37542700
C	4.65442700	3.17940500	0.88973700
C	4.16539800	4.35872600	1.47141100
H	4.56263500	4.68754300	2.43583700
C	3.19850200	5.13090600	0.83615600
H	2.83230700	6.04906900	1.30276300
C	2.70281700	4.72470100	-0.40119700
H	1.93899400	5.33153900	-0.89534300
C	3.14562900	3.55044500	-1.02075100
C	5.78641800	2.41747300	1.56927800
H	5.86301600	1.43947600	1.06342000
C	5.54375800	2.16721000	3.06221100
H	5.53761700	3.10319400	3.64270500
H	4.57485100	1.67340800	3.25394400
C	7.13381800	3.12318300	1.35773000
H	7.13432100	4.12187700	1.82423300
H	7.95906200	2.54187100	1.80068800
C	2.55369100	3.14471800	-2.36270800
H	3.04176000	2.20318600	-2.65735400
C	1.04920000	2.86419500	-2.25953700
H	0.83611600	2.10688600	-1.48906400
H	0.48322800	3.77369200	-2.00042200
C	2.84275900	4.18533400	-3.45252300
H	3.91904400	4.39549400	-3.54261400
H	2.48272800	3.83143600	-4.43230500
N	-4.50452300	-1.49731400	0.86487900
N	4.50476700	-1.49697100	-0.86496700
N	0.57951800	-0.00005700	0.10445400
C	-5.82995500	-0.00030100	2.22977600
H	-6.59808200	-0.00039300	3.00615200
C	-5.42343300	-1.28214800	1.79697500
C	-6.10504800	-2.45045200	2.48275200
C	-4.14050900	-2.76183200	0.37526500
C	-3.14617600	-3.55080600	1.02096600
C	-2.70325700	-4.72504400	0.40146900
H	-1.93977800	-5.33208300	0.89590100
C	-3.19837100	-5.13097700	-0.83620800
H	-2.83208400	-6.04912800	-1.30276700
C	-4.16480100	-4.35853300	-1.47184100
H	-4.56160100	-4.68713500	-2.43652000
C	-4.65394700	-3.17922600	-0.89022700
C	-2.55463000	-3.14521500	2.36313400
H	-3.04300500	-2.20388000	2.65789400
C	-1.05020100	-2.86421600	2.26022500
H	-0.48394200	-3.77350600	2.00100900
H	-0.83723500	-2.10671900	1.48990200
C	-2.84354000	-4.18612400	3.45270600
H	-2.33629700	-5.14198800	3.24286600
H	-2.48379700	-3.83227900	4.43261200
C	-5.78557400	-2.41711100	-1.57017700
H	-5.86233500	-1.43917000	-1.06423000
C	-5.54226400	-2.16671100	-3.06298400
H	-4.57322000	-1.67300900	-3.25429000
H	-5.53597700	-3.10264100	-3.64356300
C	-7.13313200	-3.12272100	-1.35929200
H	-7.34978700	-3.25456900	-0.28946100
H	-7.95812700	-2.54128900	-1.80255500
C	5.82964000	0.00021700	-2.23013300
H	6.59763800	0.00029000	-3.00664400
C	5.42344400	-1.28170000	-1.79724700
C	6.10523100	-2.44985800	-2.48313400
C	4.14095300	-2.76148500	-0.37520500
C	3.14647400	-3.55051500	-1.02061600
C	2.70373200	-4.72472200	-0.40092300
H	1.94016700	-5.33183200	-0.89513600
C	3.19913500	-5.13053800	0.83667600
H	2.83297200	-6.04866000	1.30338900
C	4.16569000	-4.35802200	1.47203600
H	4.56270800	-4.68653100	2.43665600
C	4.65467800	-3.17875300	0.89020800
C	2.55467900	-3.14508500	-2.36272300
H	3.04275600	-2.20360600	-2.65752200
C	1.05017500	-2.86454100	-2.25971900
H	0.48418700	-3.77398400	-2.00045500
H	0.83704600	-2.10707200	-1.48940800
C	2.84386100	-4.18590600	-3.45231100
H	2.33703200	-5.14196700	-3.24237000
H	2.48384400	-3.83223000	-4.43217700
C	5.78646400	-2.41653900	1.56978800
H	5.86293700	-1.43857700	1.06384100
C	5.54367000	-2.16620500	3.06268400
H	4.57467000	-1.67255500	3.25435100
H	5.53764900	-3.10214800	3.64324500
C	7.13400300	-3.12202200	1.35837400
H	7.35024800	-3.25384500	0.28845600
H	7.95912200	-2.54052600	1.80132400
H	-2.33661700	5.14177900	3.24285100
H	-7.35045500	3.25382700	-0.28865500
H	-6.33423200	1.52787600	-3.48664700
H	-7.13352300	-4.12137000	-1.82589000
H	-6.33327900	-1.52817700	-3.48713400
H	-3.91977300	-4.39665900	3.54257100
H	-0.66086600	-2.49798200	3.22494300
H	3.92016800	-4.39599100	-3.54231200
H	0.66067300	-2.49844800	-3.22442100
H	7.13465800	-4.12067800	1.82495700
H	-0.66073400	2.49815900	3.22457600
H	6.33478900	-1.52762700	3.48657000
H	6.33501700	1.52879800	3.48609100
H	7.34999100	3.25495400	0.28779200
H	2.33586700	5.14140000	-3.24275300
H	0.65963300	2.49791700	-3.22414500
H	-7.16520200	-2.23251200	2.67629000
H	-6.03314500	-3.37408300	1.89210800
H	-5.63206100	-2.64540800	3.45962200
H	-6.03353600	3.37348700	1.89255300
H	-7.16522100	2.23171400	2.67699700
H	-5.63189700	2.64474600	3.45989600
H	7.16507600	2.23289200	-2.67571900
H	5.63239800	2.64425300	-3.46073100

H 6.03165200 3.37432700 -1.89334800
H 7.16581300 -2.23240700 -2.67492600
H 6.03189500 -3.37398800 -1.89346500
H 5.63357900 -2.64348500 -3.46091000
Ba 3.06029000 -0.00011100 0.77892600
Ba -3.06024800 0.00003800 -0.77880500

(BDI)$_2$Ae Homoleptic Complexes

145
(BDI)$_2$Be with dispersion
Be 0.0000300 -0.2683620 -0.0000540
N 0.5422930 0.6499960 -1.4406550
N -1.3055970 -1.3672720 -0.6976860
N -0.5424550 0.6498740 1.4406520
N 1.3056200 -1.3671250 0.6975310
C 0.9365520 0.6507230 -3.9179280
C 0.4604770 0.0314170 -2.6210320
C -0.1181070 -1.2363860 -2.7861320
C -1.0019960 -1.8593190 -1.9102620
C -1.5972990 -3.1598300 -2.3989330
C 1.0437600 1.9989190 -1.5434910
C 2.4422770 2.2407420 -1.6292600
C 2.8746740 3.5176160 -2.0025510
C 1.9797810 4.5528910 -2.2491740
C 0.6214610 4.3198040 -2.1070660
C 0.1314300 3.0486210 -1.7751920
C 3.4832990 1.1653060 -1.3617870
C -1.3695200 2.8381260 -1.8295640
C -1.8113690 2.5966520 -3.2807620
C -2.1415590 4.0218450 -1.2502940
C -2.6313720 -1.7342270 -0.2779650
C -3.7357940 -1.1577210 -0.9723020
C -5.0342210 -1.5542540 -0.6396410
C -5.2783290 -2.5108680 0.3388890
C -4.2002320 -3.0957670 0.9856770
C -2.8797330 -2.7346800 0.6884560
C -3.5714280 -0.2004910 -2.1484870
C -4.4835360 1.0238980 -2.0665170
C -3.8339830 -0.9120370 -3.4822440
C -1.7745190 -3.5252360 1.3534750
C -1.9618720 -3.6075760 2.8695260
C -1.6794960 -4.9404470 0.7744520
C -0.9367160 0.6501520 3.9179800
C -0.4606410 0.0311540 2.6209300
C 0.1179190 -1.2366980 2.7858670
C 1.0019280 -1.8594160 1.9099990
C 1.5974010 -3.1599520 2.3984350
C -1.0437790 1.9988460 1.5435400
C -2.4422460 2.2408530 1.6293430
C -2.8744940 3.5177940 2.0025970
C -1.9794810 4.5529970 2.2490750
C -0.6211910 4.3197370 2.1068990
C -0.1313310 3.0484590 1.7751360
C -3.4833890 1.1655580 1.3618530
C -3.6107640 0.0853390 2.4403050
C -4.8849840 1.7306960 1.1299200
C 1.3695560 2.8377600 1.8297110
C 1.8110170 2.5964680 3.2810660
C 2.1418830 4.0212730 1.2503870
C 2.6314260 -1.7339880 0.2779090
C 2.8798890 -2.7344460 -0.6884590
C 4.2004130 -3.0957080 -0.9853630
C 5.2784250 -2.5109090 -0.3383510
C 5.0342060 -1.5542090 0.6400760
C 3.7357510 -1.1575360 0.9724480
C 1.7747300 -3.5247700 -1.3538230
C 1.6794850 -4.9401200 -0.7751790
C 1.9623580 -3.6066360 -2.8698640
C 3.5712220 -0.2002310 2.1485440
C 3.8328790 -0.9118950 3.4824260
C 4.4838880 1.0237660 2.0669990
C 4.8849770 1.7302310 -1.1298440
C 3.6105850 0.0850590 -2.4402440
H 1.5866320 1.5196840 -3.7905540
H 0.0050240 -1.6977570 -3.7631390
H 0.0685240 0.9654090 -4.5130880
H 1.4681330 -0.1099340 -4.5052500
H -0.9875920 -3.9802270 -1.9890790
H 3.9418040 3.7117970 -2.1017130
H 2.3459620 5.5410980 -2.5373790
H -0.0848820 5.1302040 -2.2924370
H -1.6061890 1.9451600 -1.2316030
H -2.8919830 2.7540680 -3.3880360
H -2.6267320 -3.3261450 -2.0652660
H -1.5521950 -3.2348060 -3.4920460
H -2.1370230 4.8834200 -1.9367580
H -1.7248030 4.3450320 -0.2899960
H -3.1936850 3.7476870 -1.0889560
H -5.8774500 -1.1186480 -1.1798160
H -6.3012080 -2.8098330 0.5798540
H -4.3806450 -3.8723930 1.7318050
H -2.5303260 0.1478020 -2.1489720
H -4.1554100 1.7238870 -1.2927230
H -5.5276890 0.7519870 -1.8560680
H -4.4839430 1.5594300 -3.0251480
H -3.2044730 -1.7962280 -3.6227290
H -3.6401520 -0.2252660 -4.3209390
H -4.8853110 -1.2359690 -3.5488850
H -0.8394770 -2.9991540 1.1305510
H -2.1593560 -2.6210430 3.3071820
H -1.0650510 -4.0241380 3.3518460
H -2.8031780 -4.2685120 3.1313000
H -2.6147650 -5.4979330 0.9427650
H -0.8642580 -5.5047860 1.2544500
H -1.4901180 -4.9297700 -0.3053210
H -0.0685960 0.9621560 4.5144120
H -1.5848540 1.5206090 3.7908720
H -1.4705520 -0.1099980 4.5039200
H -0.0052130 -1.6981900 3.7628210
H 2.6271420 -3.3256480 2.0654210
H 1.5515750 -3.2354880 3.4914840
H 0.9883780 -3.9804240 1.9877100
H -3.9416010 3.7120550 2.1018460
H -2.3455220 5.5412690 2.5372300
H 0.0852580 5.1300800 2.2921140
H -3.1601090 0.6527820 0.4447410
H -3.8065780 0.5322390 3.4270180
H -4.4526550 -0.5745780 2.1901590
H -2.7246590 -0.5517640 2.5049860
H -4.9028890 2.5700150 0.4211070
H -5.5358900 0.9376450 0.7361560

H -5.3318090 2.0802950 2.0745190
H 1.6062120 1.9446710 1.2319290
H 1.3040620 3.3000180 3.9594510
H 2.8916660 2.7535040 3.3885620
H 1.5903880 1.5766660 3.6182460
H 3.1939590 3.7468570 1.0891510
H 1.7252850 4.3444400 0.2900100
H 2.1374850 4.8829230 1.9367570
H 4.3809110 -3.8723950 -1.7314090
H 6.3013230 -2.8100020 -0.5790750
H 5.8773700 -1.1186280 1.1803730
H 0.8396950 -2.9986540 -1.1309420
H 1.4900420 -4.9296870 0.3045910
H 2.6146940 -5.4976790 -0.9435730
H 0.8642090 -5.5042360 -1.2553730
H 1.0656230 -4.0230130 -3.3525080
H 2.8037270 -4.2674670 -3.1317040
H 2.1599380 -2.6199250 -3.3070710
H 2.5302520 0.1484700 2.1485690
H 3.6392780 -0.2249460 4.3210280
H 4.8839550 -1.2365730 3.5493820
H 3.2026670 -1.7956100 3.6227960
H 4.1563720 1.7240640 1.2932230
H 4.4841720 1.5591840 3.0256940
H 5.5280160 0.7514370 1.8569350
H -1.3043260 3.2998540 -3.9594500
H -1.5912340 1.5766630 -3.6177410
H 3.1599820 0.6525720 -0.4446690
H 4.9030010 2.5693560 -0.4208310
H 5.3318120 2.0799500 -2.0743880
H 5.5357800 0.9370170 -0.7362270
H 4.4521810 -0.5751460 -2.1898750
H 3.8068430 0.5319100 -3.4268900
H 2.7242870 -0.5517440 -2.5052710

145

(BDI)$_2$Be withOut dispersion

Be 0.0000150 0.2492060 -0.0000410
N -0.5406430 -0.6768900 -1.5025580
N 1.3512380 1.3972640 -0.7061180
N 0.5405420 -0.6770920 1.5024910
N -1.3509980 1.3974520 0.7062990
C -0.8650990 -0.5896200 -3.9909430
C -0.4533620 -0.0024520 -2.6540990
C 0.0977340 1.2865120 -2.7679100
C 1.0044780 1.9006900 -1.9029470
C 1.5765340 3.2094350 -2.4110830
C -1.0488270 -2.0266280 -1.6839980
C -2.4556880 -2.2557190 -1.7709320
C -2.9087430 -3.5179310 -2.1749880
C -2.0354280 -4.5581020 -2.4684890
C -0.6736640 -4.3390550 -2.3485420
C -0.1559240 -3.0881250 -1.9769270
C -3.5073300 -1.1852990 -1.4898170
C 1.3607430 -2.9557050 -2.0052790
C 1.8848330 -2.9600270 -3.4504780
C 2.0466500 -4.0792300 -1.2204490
C 2.6959910 1.7706860 -0.3189220
C 3.7925180 1.2178430 -1.0555260
C 5.1014530 1.5865730 -0.7221360
C 5.3743230 2.4959050 0.2916190
C 4.3099090 3.0751740 0.9651900
C 2.9773570 2.7504370 0.6713060
C 3.6356280 0.3136810 -2.2805610
C 4.4930340 -0.9531340 -2.2136690
C 3.9840590 1.0670280 -3.5757910
C 1.9098090 3.5828510 1.3627160
C 2.1183210 3.6720320 2.8790950
C 1.8544980 5.0046100 0.7819950
C 0.8649540 -0.5901580 3.9908270
C 0.4533840 -0.0027140 2.6540610
C -0.0974260 1.2863790 2.7680010
C -1.0041160 1.9007760 1.9031450
C -1.5758610 3.2096320 2.4113460
C 1.0485490 -2.0269050 1.6838900
C 2.4553830 -2.2561870 1.7708750
C 2.9082590 -3.5185110 2.1747720
C 2.0348030 -4.5586010 2.4681310
C 0.6730730 -4.3393510 2.3482050
C 0.1555030 -3.0883130 1.9767090
C 3.5072150 -1.1859230 1.4899230
C 3.7379610 -0.1988720 2.6442140
C 4.8742950 -1.7728480 1.1174420
C -1.3611400 -2.9557070 2.0051140
C -1.8851920 -2.9601460 3.4503250
C -2.0471770 -4.0790920 1.2201860
C -2.6957260 1.7709640 0.3190980
C -2.9770140 2.7507250 -0.6711490
C -4.3095360 3.0754950 -0.9651250
C -5.3740100 2.4962630 -0.2916230
C -5.1012300 1.5868990 0.7221260
C -3.7923310 1.2181030 1.0555790
C -1.9094290 3.5830700 -1.3625680
C -1.8539960 5.0048050 -0.7817940
C -2.1180670 3.6723280 -2.8789230
C -3.6356050 0.3138310 2.2805490
C -3.9837200 1.0671980 3.5758520
C -4.4934420 -0.9526880 2.2136260
C -4.8745370 -1.7719780 -1.1173960
C -3.7378210 -0.1980500 -2.6439870
H -1.6035520 -1.3936310 -3.9301430
H -0.0449750 1.7751580 -3.7297160
H 0.0246450 -0.9998030 -4.4915780
H -1.2544120 0.2064160 -4.6400390
H 0.9479230 4.0274840 -2.0227610
H -3.9805070 -3.6942340 -2.2661820
H -2.4184750 -5.5324750 -2.7825110
H 0.0194910 -5.1518050 -2.5759570
H 1.6260890 -1.9970660 -1.5343750
H 2.9810100 -3.0397360 -3.4646650
H 2.6010330 3.4042030 -2.0771080
H 1.5387460 3.2655020 -3.5062530
H 1.9577620 -5.0484630 -1.7370760
H 1.6253720 -4.1916330 -0.2140370
H 3.1226120 -3.8719230 -1.1182620
H 5.9323070 1.1636180 -1.2921790
H 6.4045220 2.7697980 0.5334770
H 4.5123700 3.8281790 1.7302840
H 2.5838140 0.0026900 -2.3436730
H 4.1726700 -1.6236960 -1.4085420
H 5.5587900 -0.7256400 -2.0589100
H 4.4216610 -1.5048480 -3.1619220
H 3.4043590 1.9890180 -3.7019220
H 3.7918360 0.4276460 -4.4522990
H 5.0513540 1.3423600 -3.5951280
H 0.9485850 3.0925990 1.1653320

H 2.2737690 2.6835840 3.3308490
H 1.2452530 4.1357560 3.3641390
H 2.9892910 4.2981820 3.1310350
H 2.8069600 5.5350480 0.9445380
H 1.0612050 5.5943620 1.2699040
H 1.6571750 5.0063330 -0.2975390
H -0.0248400 -1.0005320 4.4912210
H 1.6034460 -1.3941310 3.9299250
H 1.2541420 0.2057320 4.6401740
H 0.0454330 1.7749100 3.7298420
H -2.6003970 3.4045450 2.0775760
H -1.5378420 3.2657410 3.5065060
H -0.9472150 4.0275760 2.0228590
H 3.9799990 -3.6949650 2.2659710
H 2.4177110 -5.5330630 2.7820440
H -0.0201890 -5.1520330 2.5755390
H 3.1423270 -0.5973720 0.6348700
H 3.9730490 -0.7309870 3.5805240
H 4.5897480 0.4537960 2.4023490
H 2.8787530 0.4573370 2.8194180
H 4.8088490 -2.5802120 0.3740340
H 5.5139140 -0.9796440 0.7045690
H 5.3946070 -2.1760620 2.0015050
H -1.6263970 -1.9969940 1.5343110
H -1.4862170 -3.8223750 4.0079950
H -2.9813570 -3.0400110 3.4645200
H -1.6118550 -2.0509700 4.0025550
H -3.1231110 -3.8716450 1.1179880
H -1.6258960 -4.1914820 0.2137730
H -1.9584270 -5.0483720 1.7367510
H -4.5119200 3.8284960 -1.7302460
H -6.4041840 2.7701910 -0.5335480
H -5.9321320 1.1639400 1.2920970
H -0.9482260 3.0927420 -1.1652620
H -1.6566680 5.0064700 0.2977420
H -2.8064160 5.5353260 -0.9443090
H -1.0606570 5.5945110 -1.2696830
H -1.2450390 4.1360630 -3.3640260
H -2.9890610 4.2984890 -3.1307550
H -2.2735640 2.6839000 -3.3307070
H -2.5838850 0.0025050 2.3435530
H -3.7916490 0.4276920 4.4523020
H -5.0509290 1.3428570 3.5952700
H -3.4037250 1.9889990 3.7020210
H -4.1733440 -1.6233160 1.4084500
H -4.4222230 -1.5044750 3.1618490
H -5.5591250 -0.7248150 2.0589130
H 1.4859910 -3.8222980 -4.0081770
H 1.6113640 -2.0508790 -4.0026860
H -3.1423210 -0.5969360 -0.6346900
H -4.8092280 -2.5795370 -0.3741870
H -5.3950110 -2.1748550 -2.0015150
H -5.5139470 -0.9787240 -0.7042950
H -4.5894640 0.4547950 -2.4020830
H -3.9729940 -0.7299960 -3.5803720
H -2.8784410 0.4579670 -2.8190830

145

$(BDI)_2Mg$ with Dispersion

Mg -0.217618 0.220363 0.648073
N -0.436587 -1.871715 0.775424
N -1.519609 0.581331 2.270060
N -0.544265 1.411797 -1.059994
N 1.741459 0.949408 0.931715
C -0.827695 -3.882709 2.181577
C -0.724035 -2.387403 1.966871
C -0.993654 -1.630921 3.130215
C -1.438363 -0.310866 3.268239
C -1.819305 0.089293 4.676609
C -0.352673 -2.790652 -0.316369
C 0.877345 -3.420244 -0.637974
C 0.868622 -4.422112 -1.614738
C -0.295671 -4.778409 -2.289186
C -1.477928 -4.108837 -2.008705
C -1.529340 -3.112322 -1.025821
C 2.182632 -3.046841 0.049170
C -2.865147 -2.460894 -0.721697
C -3.772116 -3.428502 0.044886
C -3.551856 -1.964474 -1.992595
C -2.490238 1.611438 2.412001
C -3.867025 1.257756 2.405234
C -4.827552 2.263286 2.550429
C -4.466641 3.597710 2.694863
C -3.118889 3.936448 2.699227
C -2.118738 2.967706 2.562560
C -4.351447 -0.179906 2.284105
C -5.287297 -0.367705 1.091576
C -5.041671 -0.649008 3.569732
C -0.669387 3.384262 2.705631
C -0.364464 4.738231 2.068246
C -0.281526 3.405266 4.186914
C -0.089861 3.514777 -2.302945
C 0.126642 2.555816 -1.151366
C 1.114767 2.987110 -0.236436
C 1.908286 2.250623 0.650268
C 3.002973 3.037877 1.335569
C -1.390959 1.074991 -2.161436
C -2.742182 1.506009 -2.191682
C -3.479813 1.278903 -3.358827
C -2.932826 0.617596 -4.454646
C -1.630250 0.145050 -4.385168
C -0.841308 0.362669 -3.248543
C -3.395324 2.207670 -1.010022
C -2.920711 3.648998 -0.797155
C -4.921003 2.222219 -1.094276
C 0.595392 -0.124074 -3.257578
C 1.457401 0.765979 -4.158312
C 0.692824 -1.586744 -3.686734
C 2.901912 0.231448 1.333993
C 3.045114 -0.283398 2.643187
C 4.213556 -0.977134 2.977474
C 5.236485 -1.168201 2.056784
C 5.093136 -0.657808 0.771748
C 3.943489 0.037548 0.385686
C 2.028773 0.000361 3.730614
C 2.454701 1.238212 4.525857
C 1.819842 -1.178386 4.679607
C 3.884807 0.592667 -1.030461
C 4.923940 1.699557 -1.241169
C 4.067268 -0.499566 -2.082564
C 3.416279 -3.544786 -0.702597
C 2.288656 -3.529414 1.499540
H -0.452733 -4.471479 1.338924
H -1.023818 -2.217214 4.048350
H -1.879744 -4.155405 2.350712
H -0.275955 -4.164440 3.089250

H	-0.896540	0.375990	5.207344
H	1.797031	-4.936320	-1.861688
H	-0.271990	-5.565156	-3.046872
H	-2.388249	-4.369122	-2.552881
H	-2.671927	-1.589237	-0.076882
H	-4.772393	-2.997716	0.189063
H	-2.497078	0.949428	4.704765
H	-2.266375	-0.745508	5.230857
H	-3.887073	-2.799579	-2.627517
H	-2.879774	-1.335390	-2.588157
H	-4.442583	-1.369208	-1.744410
H	-5.885839	1.990421	2.554519
H	-5.231565	4.369641	2.807408
H	-2.832892	4.982187	2.824557
H	-3.477447	-0.824218	2.119457
H	-4.761095	-0.201279	0.144320
H	-6.142640	0.323334	1.128809
H	-5.693310	-1.389297	1.080524
H	-4.409333	-0.502956	4.454708
H	-5.292166	-1.719451	3.502920
H	-5.980392	-0.097434	3.739342
H	-0.052910	2.621908	2.207576
H	-0.717791	4.787038	1.030059
H	0.719333	4.923368	2.064242
H	-0.828622	5.565156	2.628529
H	-0.922190	4.109621	4.741328
H	0.764221	3.721293	4.318435
H	-0.396838	2.415822	4.645179
H	0.797589	3.511558	-2.952674
H	-0.963587	3.271137	-2.914983
H	-0.197332	4.537796	-1.916280
H	1.418904	4.025921	-0.362280
H	3.834735	2.406921	1.667765
H	3.387637	3.843672	0.698030
H	2.567830	3.509147	2.232234
H	-4.512550	1.622097	-3.413986
H	-3.532826	0.455173	-5.353105
H	-1.205964	-0.395753	-5.233690
H	-3.109612	1.642133	-0.107323
H	-3.009580	4.234557	-1.725588
H	-3.537972	4.128376	-0.025088
H	-1.884724	3.695631	-0.447724
H	-5.344332	1.236391	-1.329440
H	-5.335257	2.549318	-0.130919
H	-5.270286	2.931050	-1.862318
H	0.980715	-0.045451	-2.228824
H	1.030050	0.820741	-5.172045
H	2.476248	0.364850	-4.246729
H	1.530067	1.790478	-3.770967
H	1.715855	-1.964270	-3.544795
H	0.014766	-2.219248	-3.101596
H	0.444713	-1.716586	-4.751856
H	4.329157	-1.366371	3.990635
H	6.142640	-1.708599	2.340517
H	5.898482	-0.797627	0.046318
H	1.069802	0.225936	3.241124
H	2.529025	2.123041	3.882399
H	3.441705	1.075732	4.987769
H	1.736127	1.460327	5.329162
H	0.973032	-0.982428	5.353105
H	2.702567	-1.348467	5.315968
H	1.607428	-2.106256	4.133414
H	2.890827	1.033676	-1.184336
H	4.803760	2.154890	-2.236801
H	5.948086	1.297503	-1.179560
H	4.836810	2.496164	-0.491611
H	3.232449	-1.209830	-2.066194
H	4.117993	-0.057546	-3.087817
H	4.997076	-1.067353	-1.929122
H	-3.891040	-4.371716	-0.511429
H	-3.364004	-3.673810	1.033975
H	2.222431	-1.944985	0.077238
H	3.384699	-3.312635	-1.775692
H	3.532703	-4.635496	-0.597101
H	4.314427	-3.074879	-0.279253
H	3.304544	-3.344125	1.874454
H	2.083520	-4.608976	1.571870
H	1.603860	-2.994324	2.164410

145

$(BDI)_2Mg$ withOut dispersion

Mg	0.0000490	-0.2891310	-0.0001310
N	0.4700690	0.7099730	-1.8700720
N	-1.5965830	-1.4723540	-0.8757360
N	-0.4700040	0.7100840	1.8699500
N	1.5962190	-1.4723400	0.8756310
C	0.5169860	0.5390700	-4.3506600
C	0.2234370	-0.0045170	-2.9650210
C	-0.3856130	-1.2843640	-2.9899860
C	-1.2822730	-1.9059560	-2.1069850
C	-1.9049400	-3.1757300	-2.6597170
C	1.0047550	2.0289220	-2.0798420
C	2.4137480	2.2255960	-2.1435550
C	2.8975640	3.5073200	-2.4346200
C	2.0448820	4.5872220	-2.6377450
C	0.6749540	4.3908940	-2.5487790
C	0.1308010	3.1262350	-2.2841900
C	3.4165860	1.0951280	-1.9318220
C	-1.3863350	3.0051240	-2.2718610
C	-1.9875130	3.3439510	-3.6435750
C	-2.0108130	3.8902430	-1.1882100
C	-2.8949950	-1.8336900	-0.3904470
C	-4.0510510	-1.3045130	-1.0402870
C	-5.3209560	-1.6247450	-0.5450830
C	-5.4897080	-2.4548600	0.5559280
C	-4.3644540	-2.9973880	1.1638620
C	-3.0691760	-2.7162730	0.7093750
C	-3.9967660	-0.4372010	-2.2962680
C	-4.6106520	0.9484700	-2.0794550
C	-4.6818810	-1.1251340	-3.4874700
C	-1.9115590	-3.4737080	1.3411730
C	-2.0419710	-3.6262600	2.8590850
C	-1.7665360	-4.8592530	0.6942070
C	-0.5166390	0.5393950	4.3505450
C	-0.2234230	-0.0043450	2.9649000
C	0.3852960	-1.2844150	2.9898820
C	1.2815370	-1.9063040	2.1067450
C	1.9029680	-3.1771250	2.6583550
C	-1.0046080	2.0290930	2.0796190
C	-2.4136010	2.2257940	2.1434000
C	-2.8973630	3.5075950	2.4341990
C	-2.0446390	4.5875170	2.6370430
C	-0.6747230	4.3911350	2.5480890
C	-0.1306050	3.1264140	2.2837230
C	-3.4164860	1.0952930	1.9320630

C -3.6345540 0.2291090 3.1815160
C -4.7852470 1.5917170 1.4547130
C 1.3865300 3.0052760 2.2714860
C 1.9875810 3.3443640 3.6431870
C 2.0110640 3.8902320 1.1877420
C 2.8948240 -1.8335930 0.3907250
C 3.0696760 -2.7163440 -0.7088440
C 4.3652490 -2.9975110 -1.1625090
C 5.4901410 -2.4548610 -0.5540480
C 5.3207220 -1.6243720 0.5465910
C 4.0505290 -1.3040000 1.0409250
C 1.9126200 -3.4739220 -1.3415300
C 1.7688940 -4.8604840 -0.6964360
C 2.0432580 -3.6244250 -2.8596410
C 3.9954370 -0.4361910 2.2965450
C 4.6775280 -1.1248110 3.4890830
C 4.6117540 0.9485190 2.0804330
C 4.7852610 1.5915870 -1.4542560
C 3.6348860 0.2287280 -3.1810790
H 1.2723850 1.3316950 -4.3663620
H -0.3020920 -1.7868720 -3.9538800
H -0.4125610 0.9563630 -4.7694430
H 0.8295190 -0.2729320 -5.0215650
H -1.1685020 -3.9901200 -2.5585650
H 3.9742060 3.6684190 -2.5010090
H 2.4510170 5.5774480 -2.8596720
H 0.0012000 5.2374310 -2.7037920
H -1.6374250 1.9586030 -2.0409290
H -3.0798500 3.2082690 -3.6328070
H -2.8110020 -3.4783190 -2.1236920
H -2.1356980 -3.0833230 -3.7292400
H -1.8218290 4.9581270 -1.3825050
H -1.6123450 3.6575210 -0.1914860
H -3.1031940 3.7550410 -1.1575460
H -6.2032280 -1.2163190 -1.0454870
H -6.4903240 -2.6917210 0.9266370
H -4.4949140 -3.6787760 2.0070920
H -2.9427470 -0.2850340 -2.5647120
H -4.0727600 1.5099830 -1.3051100
H -5.6675250 0.8855760 -1.7752590
H -4.5729120 1.5332250 -3.0116790
H -4.2942310 -2.1380690 -3.6630430
H -4.5305260 -0.5400630 -4.4089510
H -5.7692150 -1.2122260 -3.3301480
H -0.9909800 -2.9071750 1.1343350
H -2.2244480 -2.6615180 3.3516100
H -1.1194480 -4.0517220 3.2821470
H -2.8601740 -4.3109300 3.1342660
H -2.6793640 -5.4574750 0.8494680
H -0.9229530 -5.4142680 1.1346220
H -1.5950280 -4.7904210 -0.3880030
H 0.4130960 0.9564170 4.7691780
H -1.2718030 1.3322450 4.3662990
H -0.8293270 -0.2724780 5.0215300
H 0.3014460 -1.7869690 3.9537030
H 2.8125900 -3.4762860 2.1264230
H 2.1271930 -3.0886150 3.7295700
H 1.1685680 -3.9923670 2.5493400
H -3.9739960 3.6687440 2.5006100
H -2.4507400 5.5777990 2.8587790
H -0.0009440 5.2376830 2.7029410
H -3.0046740 0.4367790 1.1490610
H -3.9380290 0.8489700 4.0413220
H -4.4329230 -0.5039830 2.9909260
H -2.7394840 -0.3359090 3.4653940
H -4.7080150 2.3154600 0.6311790
H -5.3849890 0.7395420 1.1045940
H -5.3494650 2.0722520 2.2706080
H 1.6376570 1.9587240 2.0407380
H 1.7910370 4.3932870 3.9171890
H 3.0799260 3.2087700 3.6325220
H 1.5760120 2.7141010 4.4448740
H 3.1034450 3.7550140 1.1571580
H 1.6126510 3.6573490 0.1910360
H 1.8220680 4.9581470 1.3818530
H 4.4961960 -3.6790740 -2.0055340
H 6.4909760 -2.6918180 -0.9241020
H 6.2026950 -1.2157430 1.0473610
H 0.9915430 -2.9084540 -1.1339330
H 1.5981920 -4.7932900 0.3860020
H 2.6819790 -5.4579260 -0.8531790
H 0.9253370 -5.4153900 -1.1370350
H 1.1213160 -4.0506010 -3.2832680
H 2.8623740 -4.3076810 -3.1356100
H 2.2244880 -2.6588540 -3.3509720
H 2.9411880 -0.2824270 2.5631360
H 4.5263540 -0.5387390 4.4099590
H 5.7648490 -1.2144640 3.3330900
H 4.2873670 -2.1367150 3.6649710
H 4.0759160 1.5107060 1.3051520
H 4.5733990 1.5334760 3.0125020
H 5.6690270 0.8840940 1.7779550
H -1.7910790 4.3928500 -3.9177490
H -1.5759620 2.7136020 -4.4452030
H 3.0046100 0.4367560 -1.1487890
H 4.7078670 2.3155790 -0.6309520
H 5.3497280 2.0718620 -2.2701320
H 5.3848570 0.7394750 -1.1037310
H 4.4331190 -0.5044320 -2.9901850
H 3.9386500 0.8484370 -4.0408960
H 2.7398170 -0.3362050 -3.4651180

145

$(BDI)_2Ca$ with dispersion

Ca -0.000560 -0.635898 -0.000674
N 1.128758 -1.750094 -1.820391
N 2.144524 0.305379 0.309419
C 2.315054 -2.261745 -1.466580
C 3.210277 -1.704270 -0.533396
H 4.138402 -2.267347 -0.426896
C 3.219908 -0.446971 0.119056
C 2.799708 -3.558554 -2.086900
H 2.859063 -4.320797 -1.293504
H 3.809911 -3.455342 -2.505933
H 2.127647 -3.924698 -2.871187
C 4.564795 0.041332 0.599948
H 5.359929 -0.679929 0.377303
H 4.552911 0.242666 1.679798
H 4.807816 0.998438 0.113882
C 2.304178 1.605041 0.862452
C 2.333871 2.723900 -0.001192
C 2.473113 4.002074 0.550430
H 2.495555 4.869367 -0.114024
C 2.587452 4.187855 1.923879
H 2.702403 5.192715 2.337034
C 2.535949 3.083293 2.767835

H	2.598758	3.228926	3.849075
C	2.383348	1.788196	2.263304
C	2.227839	2.571640	-1.505898
H	2.096877	1.498553	-1.716846
C	3.515297	3.027794	-2.196438
H	4.381999	2.440763	-1.856531
H	3.727158	4.087898	-1.985187
H	3.435495	2.915191	-3.287416
C	1.005195	3.312893	-2.047774
H	0.898047	3.155795	-3.131338
H	1.081934	4.398031	-1.877090
H	0.078606	2.971640	-1.565007
C	2.218365	0.633912	3.231642
H	2.192373	-0.296203	2.645525
C	0.875648	0.762020	3.947337
H	0.722538	-0.063248	4.653756
H	0.045728	0.747322	3.227604
H	0.815354	1.709809	4.505006
C	3.358578	0.510696	4.242158
H	3.212501	-0.385260	4.865047
H	3.397899	1.379502	4.918042
H	4.340025	0.429475	3.750857
C	0.648018	-2.037799	-3.118556
C	-0.632328	-2.620529	-3.305842
C	-1.102311	-2.841992	-4.603392
H	-2.084587	-3.292514	-4.747644
C	-0.337385	-2.517408	-5.719952
H	-0.719852	-2.705824	-6.725677
C	0.913587	-1.943185	-5.535540
H	1.510301	-1.668198	-6.408765
C	1.419760	-1.679020	-4.258449
C	-1.451764	-3.094114	-2.121572
H	-1.375218	-2.339564	-1.320306
C	-0.879817	-4.402876	-1.574778
H	-1.435984	-4.736188	-0.685653
H	0.174388	-4.289178	-1.294938
H	-0.946315	-5.193521	-2.338522
C	-2.940720	-3.242482	-2.412877
H	-3.476340	-3.488634	-1.486985
H	-3.135101	-4.052026	-3.133188
H	-3.373152	-2.314089	-2.814446
C	2.754289	-0.953759	-4.169129
H	3.038901	-0.871315	-3.111899
C	2.640062	0.472745	-4.713657
H	3.609728	0.989266	-4.644824
H	1.901090	1.058401	-4.152331
H	2.333108	0.474184	-5.771477
C	3.872242	-1.707313	-4.896618
H	4.846930	-1.232026	-4.704164
H	3.716428	-1.703489	-5.987205
H	3.934303	-2.757144	-4.578245
N	-1.129421	-1.748439	1.820290
C	-2.315235	-2.260642	1.465734
C	-3.210158	-1.703653	0.531983
H	-4.137866	-2.267279	0.424901
C	-3.220171	-0.446455	-0.120663
N	-2.145157	0.306458	-0.310735
C	-2.304869	1.605927	-0.863945
C	-2.333026	2.724969	-0.000510
C	-2.471633	4.003126	-0.552326
H	-2.492944	4.870585	0.111947
C	-2.586698	4.188660	-1.925753
H	-2.701010	5.193521	-2.339082

C	-2.536787	3.083849	-2.769509
H	-2.600199	3.229302	-3.850736
C	-2.385013	1.788742	-2.264777
C	-2.221417	0.633963	-3.232782
H	-2.196804	-0.296050	-2.646408
C	-0.878353	0.759922	-3.948140
H	-0.726109	-0.066007	-4.653989
H	-0.048574	0.744508	-3.228265
H	-0.816679	1.707317	-4.506335
C	-3.361545	0.512086	-4.243567
H	-3.216827	-0.384429	-4.865980
H	-3.399210	1.380583	-4.919928
H	-4.343285	0.432777	-3.752532
C	-2.226566	2.572726	1.504176
H	-2.093724	1.499820	1.714924
C	-3.515125	3.026296	2.194445
H	-4.380517	2.437401	1.854431
H	-3.729086	4.085913	1.982837
H	-3.435361	2.914022	3.285471
C	-1.005477	3.316057	2.046585
H	-0.898457	3.158740	3.130132
H	-1.084108	4.401137	1.876375
H	-0.078133	2.976547	1.564038
C	-4.565238	0.041225	-0.601742
H	-5.359929	-0.680785	-0.379962
H	-4.553257	0.243634	-1.681353
H	-4.809066	0.997670	-0.114748
C	-2.799159	-3.558154	2.085156
H	-2.854938	-4.320640	1.291716
H	-3.810622	-3.456723	2.501568
H	-2.128329	-3.922956	2.871110
C	-0.648972	-2.036292	3.118421
C	0.631979	-2.617712	3.305872
C	1.101561	-2.839564	4.603468
H	2.084289	-3.288976	4.747950
C	0.335768	-2.516501	5.719915
H	0.718015	-2.705162	6.725677
C	-0.915654	-1.943352	5.535340
H	-1.513045	-1.669493	6.408453
C	-1.421543	-1.678971	4.258165
C	-2.756575	-0.954676	4.168558
H	-3.040938	-0.872231	3.111264
C	-2.643352	0.471891	4.713169
H	-3.613186	0.988021	4.643666
H	-1.904238	1.057795	4.152264
H	-2.337063	0.473499	5.771191
C	-3.874298	-1.709106	4.895446
H	-4.849170	-1.234170	4.703038
H	-3.718767	-1.705815	5.986072
H	-3.935811	-2.758795	4.576436
C	1.452938	-3.087782	2.121241
H	1.380099	-2.328959	1.323642
C	0.879896	-4.392662	1.566553
H	1.437995	-4.722553	0.677403
H	-0.173234	-4.275470	1.284194
H	0.942675	-5.187242	2.326514
C	2.941021	-3.240321	2.414774
H	3.477782	-3.483425	1.488734
H	3.132419	-4.053383	3.131920
H	3.374550	-2.314537	2.821161

145

$(BDI)_2Ca$ withOut dispersion

Ca 0.0000710 -0.3462410 -0.0001310
N 1.9999260 -1.4865460 -0.8128800
N 0.0176630 0.7314890 -2.1465370
C 1.7562120 -1.9559930 -2.0409470
C 0.9267790 -1.3622060 -3.0184550
H 0.9004590 -1.9345650 -3.9466980
C 0.3353540 -0.0761910 -3.1507010
C 2.3913720 -3.2567140 -2.5091490
H 1.5998560 -4.0180530 -2.6045070
H 2.8492680 -3.1498540 -3.5022690
H 3.1467470 -3.6344190 -1.8106290
C 0.1013620 0.3665730 -4.5804390
H 0.3785380 -0.4173150 -5.2954580
H 0.9418170 0.6593020 -4.7571530
H 0.7093210 1.2593180 -4.7937200
C 0.4780720 2.0367950 -2.4454670
C 0.3976240 3.1515580 -2.3847770
C 0.1100800 4.4314600 -2.6413060
H 0.5649010 5.2902360 -2.5956130
C 1.4472940 4.6335910 -2.9612000
H 1.8235800 5.6403180 -3.1601600
C 2.3002300 3.5372530 -3.0260540
H 3.3527280 3.6936820 -3.2766690
C 1.8461730 2.2370080 -2.7755530
C 1.8829610 3.0053560 -2.0916600
H 2.0660050 1.9461820 -1.8523400
C 2.7257080 3.3543840 -3.3261170
H 2.4667900 2.7228730 -4.1896130
H 2.5777120 4.4038890 -3.6281350
H 3.7981700 3.2175860 -3.1182970
C 2.3156310 3.8422140 -0.8833190
H 3.3792860 3.6718680 -0.6544150
H 2.1928540 4.9210690 -1.0707840
H 1.7332150 3.5952950 0.0165410
C 2.8534740 1.0972170 -2.8366940
H 2.2978240 0.1504950 -2.7576280
C 3.8111070 1.1607710 -1.6445700
H 4.5051080 0.3072760 -1.6478240
H 3.2615760 1.1388360 -0.6926250
H 4.4062900 2.0882350 -1.6636400
C 3.6446840 1.0584520 -4.1502760
H 4.2835430 0.1618850 -4.1823350
H 4.3065850 1.9328730 -4.2541230
H 2.9886080 1.0377040 -5.0341270
C 3.2195940 -1.8548920 -0.1819630
C 3.2241240 -2.6081660 1.0248400
C 4.4425700 -2.8907830 1.6541220
H 4.4442330 -3.4730910 2.5774630
C 5.6560560 -2.4693110 1.1217600
H 6.5957890 -2.7058860 1.6271890
C 5.6531760 -1.7516890 -0.0674940
H 6.6052120 -1.4235110 -0.4935140
C 4.4649050 -1.4281230 -0.7336310
C 1.9526360 -3.2171360 1.5972330
H 1.1063850 -2.5676640 1.3154760
C 1.6932950 -4.5967290 0.9761010
H 0.7628660 -5.0391910 1.3659520
H 1.6086940 -4.5412710 -0.1174100
H 2.5201950 -5.2858020 1.2128950
C 1.9472560 -3.3196140 3.1234770
H 0.9580840 -3.6441920 3.4772580
H 2.6773250 -4.0596500 3.4876790
H 2.1734920 -2.3547020 3.6006090
C 4.5890190 -0.6241750 -2.0255350
H 3.5780930 -0.4423930 -2.4166560
C 5.2382560 0.7460640 -1.7927110
H 5.3022500 1.3049220 -2.7396420
H 4.6662350 1.3530240 -1.0781100
H 6.2633760 0.6481250 -1.4008460
C 5.3755630 -1.3888930 -3.1010630
H 5.3567650 -0.8408080 -4.0568860
H 6.4328560 -1.5099720 -2.8143170
H 4.9681920 -2.3938190 -3.2787020
N 2.0000250 -1.4870270 0.8121210
C 1.7565010 -1.9571100 2.0399820
C 0.9270780 -1.3639360 3.0178620
H 0.9009400 -1.9367430 3.9458340
C 0.3354760 -0.0780750 3.1507680
N 0.0176210 0.7300570 2.1470200
C 0.4782380 2.0351610 2.4466320
C 0.3973510 3.1500370 2.3864730
C 0.1104630 4.4297630 2.6436580
H 0.5644380 5.2886240 2.5983810
C 1.4476890 4.6316140 2.9636800
H 1.8240620 5.6382070 3.1631560
C 2.3005290 3.5351690 3.0279760
H 3.3530400 3.6913750 3.2786740
C 1.8463580 2.2350900 2.7768200
C 2.8535720 1.0951900 2.8373880
H 2.2978410 0.1485520 2.7578780
C 3.8111850 1.1592430 1.6452750
H 4.5051240 0.3056940 1.6481080
H 3.2616290 1.1377960 0.6933360
H 4.4064400 2.0866510 1.6647750
C 3.6448180 1.0557380 4.1509310
H 4.2836200 0.1591150 4.1825420
H 4.3067780 1.9300680 4.2551750
H 2.9887720 1.0346090 5.0347940
C 1.8826910 3.0041120 2.0932250
H 2.0658040 1.9450810 1.8533190
C 2.7254590 3.3525190 3.3278440
H 2.4666420 2.7204950 4.1909950
H 2.5773690 4.4018390 3.6304600
H 3.7979250 3.2159430 3.1199030
C 2.3152740 3.8416620 0.8853290
H 3.3789220 3.6714770 0.6562770
H 2.1924800 4.9204080 1.0734090
H 1.7328200 3.5952360 -0.0146410
C 0.1015300 0.3639600 4.5807380
H 0.3787230 -0.4202970 5.2953470
H 0.9416350 0.6566150 4.7576340
H 0.7095110 1.2565860 4.7944570
C 2.3919550 -3.2579330 2.5074930
H 1.6005730 -4.0194230 2.6027440
H 2.8500870 -3.1514080 3.5005410
H 3.1472180 -3.6352400 1.8086350
C 3.2196750 -1.8548960 0.1809010
C 3.2241870 -2.6075180 -1.0263160
C 4.4426090 -2.8896210 -1.6558750
H 4.4442640 -3.4714200 -2.5795370
C 5.6560930 -2.4682900 -1.1233940
H 6.5958070 -2.7044530 -1.6290500
C 5.6532370 -1.7513650 0.0662790
H 6.6052720 -1.4233330 0.4924100
C 4.4649870 -1.4283280 0.7327120
C 4.5891200 -0.6251620 2.0251010

H	3.5782100	-0.4438050	2.4164630
C	5.2380750	0.7453430	1.7930510
H	5.3020880	1.3036240	2.7403210
H	4.6658480	1.3526390	1.0789020
H	6.2631600	0.6478320	1.4009890
C	5.3759210	-1.3904160	3.1000610
H	5.3571040	-0.8429490	4.0562370
H	6.4332130	-1.5111010	2.8131420
H	4.9687660	-2.3955390	3.2770910
C	1.9527260	-3.2164220	-1.5988490
H	1.1064140	-2.5671650	-1.3167800
C	1.6936050	-4.5962800	-0.9782110
H	0.7632070	-5.0387190	-1.3681600
H	1.6090710	-4.5412360	0.1153260
H	2.5205800	-5.2851540	-1.2153190
C	1.9472200	-3.3183330	-3.1251300
H	0.9580530	-3.6428960	-3.4789420
H	2.6773350	-4.0581550	-3.4896720
H	2.1733000	-2.3532200	-3.6019280

145

$(BDI)_2Sr$ with dispersion

Sr	0.019037	-0.399004	0.101510
N	2.331159	0.565888	0.281534
N	1.273895	-1.536988	-1.822017
C	4.752134	0.309703	0.530974
C	3.399125	-0.188284	0.080330
C	3.374221	-1.455078	-0.555407
C	2.468149	-2.028512	-1.471569
C	2.965533	-3.319030	-2.098453
C	2.453019	1.853032	0.855071
C	2.528056	2.006948	2.261116
C	2.568108	3.296708	2.798139
C	2.522800	4.422257	1.980157
C	2.425481	4.262597	0.601554
C	2.388645	2.991646	0.018874
C	2.467608	0.808378	3.188284
C	3.566220	0.801773	4.250724
C	1.086628	0.723931	3.836582
C	2.259051	2.852658	-1.485079
C	0.928813	3.429541	-1.971894
C	3.446942	3.482878	-2.214093
C	0.768403	-1.874524	-3.095504
C	1.500866	-1.518017	-4.261049
C	0.973418	-1.824531	-5.519633
C	-0.258475	-2.451022	-5.659020
C	-0.988677	-2.769916	-4.517261
C	-0.503452	-2.492170	-3.236171
C	-1.318567	-2.891116	-2.018107
C	-2.779415	-3.208404	-2.320213
C	-0.673296	-4.055457	-1.266698
C	2.802259	-0.731778	-4.206530
C	2.577676	0.713286	-4.660551
C	3.917701	-1.384732	-5.027000
H	4.300373	-2.022223	-0.446325
H	2.373440	5.145445	-0.040721
H	2.621173	3.422163	3.882477
H	2.596769	-0.095932	2.575037
H	3.141416	-0.686429	-3.163498
H	2.551509	5.422596	2.418535
H	2.262654	1.774656	-1.709146
H	1.835677	1.217890	-4.028529
H	1.537334	-1.546747	-6.413486
H	3.234463	-4.024870	-1.297329
H	3.523630	-0.130361	4.835024
H	4.979169	1.261368	0.025821
H	3.873277	-3.152938	-2.696062
H	-1.960236	-3.250857	-4.630354
H	5.548022	-0.408973	0.301988
H	-0.656413	-2.680209	-6.650188
H	3.453398	1.638043	4.958451
H	4.051092	-2.444134	-4.763827
H	-2.877585	-4.115545	-2.936472
H	0.997270	-0.164035	4.475769
H	0.886284	1.615476	4.450985
H	3.516649	1.286078	-4.608335
H	0.286392	0.669044	3.083054
H	2.213490	0.753253	-5.699553
H	2.209979	-3.785766	-2.741330
H	-1.234038	-4.282641	-0.349110
H	-3.318711	-3.378260	-1.379314
H	0.804416	3.279788	-3.054693
H	-3.278755	-2.379713	-2.843958
H	4.568181	0.880247	3.802004
H	0.861478	4.510902	-1.776092
H	-1.354528	-2.023605	-1.335398
H	3.352615	3.347234	-3.301974
H	0.363737	-3.830882	-0.988032
H	0.070269	2.960305	-1.468843
H	-0.665341	-4.957062	-1.898758
H	3.709361	-1.338114	-6.107716
H	3.511584	4.565118	-2.018471
H	4.761611	0.525860	1.608790
H	4.874510	-0.866418	-4.857595
H	4.397560	3.029103	-1.894963
N	-2.322663	0.436687	-0.266608
C	-3.407298	-0.304212	-0.092848
C	-4.736367	0.204922	-0.599916
H	-4.974116	1.164672	-0.115470
H	-5.548022	-0.503352	-0.395653
H	-4.700490	0.408570	-1.678922
C	-3.436019	-1.554045	0.572879
C	-2.561982	-2.136865	1.513845
N	-1.353177	-1.684505	1.850343
C	-0.828077	-2.004629	3.120980
C	-1.464935	-1.509867	4.292501
C	-0.895773	-1.774102	5.541474
C	0.291603	-2.487906	5.665509
C	0.919697	-2.958993	4.517167
C	0.378822	-2.737531	3.246052
C	1.033127	-3.393495	2.045687
C	2.557897	-3.348425	2.078925
H	2.962886	-3.968649	2.893137
H	2.971926	-3.731544	1.136419
H	2.930844	-2.323060	2.209203
C	0.534912	-4.836097	1.912356
H	0.953173	-5.322458	1.017666
H	-0.560915	-4.874039	1.844954
H	0.835349	-5.422596	2.794981
H	0.695504	-2.863413	1.138984
H	1.845355	-3.530051	4.611588
H	0.725566	-2.676098	6.650188
H	-1.388752	-1.394414	6.440142
C	-2.728230	-0.666175	4.241328
C	-2.474980	0.742743	4.782379
H	-1.694851	1.257437	4.206467

H -3.393614 1.346582 4.729279
H -2.151970 0.719004 5.835124
C -3.887292 -1.328482 4.991974
H -4.067066 -2.353512 4.639487
H -3.685988 -1.381702 6.073986
H -4.816263 -0.752371 4.856809
H -3.027877 -0.560573 3.189791
C -3.106080 -3.393275 2.167843
H -3.118294 -4.200165 1.416690
H -4.141144 -3.257764 2.510238
H -2.491949 -3.721776 3.014377
H -4.385580 -2.082489 0.475796
C -2.438305 1.731006 -0.832290
C -2.484307 1.910116 -2.236479
C -2.574087 3.207494 -2.749697
C -2.590719 4.319085 -1.913250
C -2.498411 4.137947 -0.537316
C -2.421553 2.858803 0.022822
C -2.301104 2.708221 1.526997
C -0.989726 3.318306 2.027228
H -0.863906 3.160362 3.108737
H -0.954192 4.403244 1.844432
H -0.113410 2.885362 1.521199
C -3.511007 3.302583 2.249911
H -3.418295 3.173037 3.338171
H -3.608125 4.381675 2.051453
H -4.444999 2.818552 1.926473
H -2.280784 1.628679 1.744282
H -2.484983 5.010294 0.120971
H -2.657265 5.325023 -2.334294
H -2.613045 3.349495 -3.832641
C -2.337580 0.744033 -3.194790
C -3.457848 0.654788 -4.231001
H -3.329146 -0.247723 -4.848450
H -3.452196 1.521768 -4.910369
H -4.451941 0.608918 -3.761224
C -0.974100 0.821458 -3.880755
H -0.826792 -0.018177 -4.571997
H -0.872676 1.758741 -4.449799
H -0.158238 0.793855 -3.144735
H -2.358955 -0.181356 -2.600870

145

(BDI)$_2$Sr withOu dispersion

Sr 0.0135930 -0.3153260 -0.0223140
N -0.2828490 0.7705200 -2.3089380
N -2.1570960 -1.5008100 -0.8194320
C -0.5798320 0.4266260 -4.7255150
C -0.6958110 -0.0283680 -3.2840560
C -1.2913930 -1.3082670 -3.1151030
C -2.0414290 -1.9188290 -2.0805810
C -2.7717660 -3.1709090 -2.5460770
C 0.2144010 2.0669630 -2.6193000
C 1.5627900 2.2478770 -3.0337800
C 2.0333670 3.5454460 -3.2661440
C 1.2140390 4.6580530 -3.1050590
C -0.1043070 4.4757230 -2.7030680
C -0.6256190 3.1996550 -2.4566040
C 2.5260160 1.0812680 -3.2056350
C 3.2270980 1.0731870 -4.5696950
C 3.5614520 1.0569450 -2.0776250
C -2.0861620 3.0686380 -2.0524150
C -2.4053930 3.8640900 -0.7819570
C -3.0204420 3.4751120 -3.2000300
C -3.2857370 -1.8966980 -0.0598220
C -4.5913560 -1.4557050 -0.4283870
C -5.6799950 -1.7922920 0.3854210
C -5.5253680 -2.5415170 1.5448930
C -4.2530690 -2.9794750 1.8984740
C -3.1296380 -2.6797810 1.1192990
C -1.7865230 -3.2852920 1.5040210
C -1.6152980 -3.5256970 3.0046680
C -1.5311530 -4.5845380 0.7280740
C -4.8758200 -0.6116090 -1.6675660
C -5.3935470 0.7850240 -1.3009900
C -5.8658370 -1.3010040 -2.6179460
H -1.3590220 -1.8625630 -4.0529660
H -0.7519310 5.3481490 -2.5805810
H 3.0705200 3.6874520 -3.5815990
H 1.9409900 0.1518210 -3.1331380
H -3.9322560 -0.4724790 -2.2139020
H 1.6019740 5.6624460 -3.2930450
H -2.2676390 2.0037550 -1.8385680
H -4.6750460 1.3337300 -0.6770850
H -6.6791360 -1.4500610 0.1019710
H -2.0327470 -3.9006500 -2.9133440
H 3.8336350 0.1606550 -4.6818460
H -1.2474700 1.2868510 -4.8913400
H -3.4430690 -2.9513700 -3.3888580
H -4.1342100 -3.5832120 2.7998700
H -0.8664570 -0.3675080 -5.4255600
H -6.3892460 -2.7891000 2.1668860
H 3.9074740 1.9319590 -4.6842740
H -5.5520180 -2.3236310 -2.8719190
H -2.2649220 -4.3389940 3.3647710
H 4.2191520 0.1784000 -2.1584740
H 4.1919830 1.9603980 -2.1032560
H -5.5770930 1.3788130 -2.2105880
H 3.0834670 1.0208020 -1.0868820
H -6.3437410 0.7307450 -0.7454690
H -3.3582590 -3.6404430 -1.7476390
H -0.5535290 -5.0171020 0.9931550
H -0.5785180 -3.8190320 3.2234510
H -3.4498280 3.7001170 -0.4731320
H -1.8376560 -2.6235100 3.5931870
H 2.5109620 1.1076710 -5.4050180
H -2.2789690 4.9475380 -0.9372760
H -0.9945080 -2.5749050 1.2081660
H -4.0747730 3.3370040 -2.9131630
H -1.5465820 -4.4200690 -0.3578740
H -1.7560500 3.5762240 0.0586290
H -2.3065790 -5.3304560 0.9652040
H -6.8723000 -1.3678760 -2.1739990
H -2.8862090 4.5351590 -3.4703920
H 0.4319700 0.7740710 -4.9719810
H -5.9611760 -0.7313680 -3.5564050
H -2.8368180 2.8776500 -4.1059550
N 0.2603770 0.7825810 2.2735440
C 0.6384520 -0.0246040 3.2526420
C 0.5179310 0.4289910 4.6937750
H 1.2519530 1.2287200 4.8825200
H 0.7224170 -0.3899150 5.3942070
H -0.4669940 0.8582710 4.9175110
C 1.2081650 -1.3208920 3.0884240
C 1.9692800 -1.9331000 2.0671500
N 2.1618060 -1.4651050 0.8298560

C 3.3167760 -1.8850650 0.1171400
C 4.5763890 -1.2979820 0.4425730
C 5.7138730 -1.6813040 -0.2749110
C 5.6464540 -2.6055440 -1.3134830
C 4.4172730 -3.1667520 -1.6359170
C 3.2491400 -2.8333110 -0.9364010
C 1.9661110 -3.5626510 -1.3062580
C 1.5999490 -3.3856650 -2.7832180
H 2.3590410 -3.8376190 -3.4411250
H 0.6381180 -3.8711910 -3.0083420
H 1.5054370 -2.3254990 -3.0584530
C 2.0503400 -5.0546490 -0.9539430
H 1.0957910 -5.5628240 -1.1639030
H 2.2945400 -5.2097510 0.1069380
H 2.8312110 -5.5566070 -1.5475600
H 1.1532000 -3.1329830 -0.6958980
H 4.3627370 -3.9018120 -2.4437040
H 6.5472010 -2.8880880 -1.8643890
H 6.6797900 -1.2387550 -0.0185240
C 4.7244220 -0.2699110 1.5588470
C 5.5759490 0.9367990 1.1472930
H 5.2086680 1.4014740 0.2214300
H 5.5608380 1.7003570 1.9405200
H 6.6315730 0.6639040 0.9890770
C 5.2959760 -0.8918670 2.8411970
H 4.6686000 -1.7088480 3.2217900
H 6.3052060 -1.2996060 2.6647310
H 5.3758160 -0.1344850 3.6377720
H 3.7129090 0.0980630 1.7887670
C 2.5908850 -3.2622780 2.4667820
H 1.9301310 -4.0783140 2.1285610
H 2.7040740 -3.3570340 3.5543780
H 3.5660390 -3.4245210 1.9902200
H 1.2253200 -1.8878400 4.0194620
C -0.2291610 2.0840760 2.5778660
C -1.5819710 2.2746090 2.9729270
C -2.0447340 3.5756650 3.2010880
C -1.2144270 4.6819500 3.0526330
C 0.1078190 4.4895040 2.6690500
C 0.6225320 3.2093030 2.4297880
C 2.0876740 3.0673260 2.0479010
C 2.4236290 3.8309340 0.7621750
H 3.4711570 3.6572180 0.4700400
H 2.2955390 4.9178840 0.8894750
H 1.7832990 3.5233780 -0.0784970
C 3.0070980 3.4992420 3.1980800
H 4.0651170 3.3503630 2.9306940
H 2.8746750 4.5662990 3.4398680
H 2.8074080 2.9258320 4.1161000
H 2.2707040 1.9980010 1.8609950
H 0.7643530 5.3565940 2.5564000
H -1.5970650 5.6892030 3.2360410
H -3.0851090 3.7255710 3.5017470
C -2.5582580 1.1160510 3.1253660
C -3.2851460 1.1125060 4.4757550
H -3.9041870 0.2066970 4.5727530
H -3.9579200 1.9785020 4.5804770
H -2.5844750 1.1356470 5.3245350
C -3.5701370 1.1040560 1.9762210
H -4.2376120 0.2317710 2.0417810
H -4.1918750 2.0137800 1.9862640
H -3.0677570 1.0624140 0.9981250
H -1.9821270 0.1803680 3.0619570

145

(BDI)$_2$Ba with dispersion

Ba 0.424818 -0.044081 -0.308196
N 1.807377 2.283912 -0.073147
N 1.691937 0.101837 2.134964
N -1.922420 -0.321050 -1.634671
N 0.535941 -1.857363 -2.401048
C 3.878670 3.579188 0.101107
C 2.995400 2.420397 0.514153
C 3.513334 1.597557 1.532429
C 2.883730 0.633463 2.355780
C 3.677785 0.213969 3.576113
C 1.297234 3.402199 -0.769214
C 0.695456 4.455914 -0.033610
C 0.212073 5.572963 -0.718778
C 0.284698 5.658656 -2.106843
C 0.827203 4.600170 -2.827653
C 1.337435 3.468890 -2.181902
C 0.514972 4.357546 1.470499
C 1.134927 5.538138 2.218548
C -0.969181 4.201733 1.813109
C 1.906717 2.328169 -3.000242
C 3.013803 2.780196 -3.952726
C 0.797275 1.592454 -3.756240
C 1.116681 -0.749088 3.101406
C 1.477500 -2.115769 3.175008
C 0.845919 -2.936942 4.114593
C -0.128584 -2.434266 4.969492
C -0.497920 -1.093841 4.877510
C 0.101247 -0.236079 3.950268
C -0.299861 1.225251 3.857226
C -1.702264 1.499284 4.391766
C 0.721095 2.135584 4.548921
C 2.476962 -2.712184 2.204464
C 1.752081 -3.541378 1.146473
C 3.563422 -3.545234 2.884413
C -3.725175 -0.666421 -3.276534
C -2.278031 -0.704561 -2.838773
C -1.349602 -1.185611 -3.794592
C -0.081947 -1.750241 -3.581221
C 0.625742 -2.272715 -4.815729
C -2.784870 0.130169 -0.631178
C -3.346116 1.432288 -0.658412
C -4.016974 1.895960 0.478326
C -4.151476 1.108886 1.617885
C -3.620791 -0.179735 1.630833
C -2.939911 -0.689257 0.523029
C -3.230183 2.321540 -1.881508
C -2.369443 3.551043 -1.606180
C -4.606212 2.723149 -2.419375
C -2.449581 -2.126028 0.486049
C -3.521725 -3.014858 -0.153815
C -2.003470 -2.669670 1.838854
C 1.513249 -2.862648 -2.264909
C 2.892899 -2.554422 -2.230304
C 3.823793 -3.585704 -2.058076
C 3.416526 -4.910269 -1.938875
C 2.054442 -5.209335 -1.946904
C 1.089608 -4.208646 -2.078905
C -0.389704 -4.548438 -1.986480
C -0.686183 -5.547257 -0.866817
C -0.964561 -5.057483 -3.311693

C 3.373211 -1.126306 -2.388540
C 4.315360 -0.958254 -3.581269
C 4.020860 -0.601444 -1.105729
H 3.949478 3.641793 -0.995061
H 3.446580 4.534927 0.433007
H 4.886647 3.489785 0.524572
H 4.525531 1.855764 1.844123
H 3.026291 0.084289 4.451207
H 4.461400 0.943200 3.815916
H 4.162312 -0.758490 3.395939
H -0.245335 6.389471 -0.153789
H -0.098742 6.540294 -2.625812
H 0.862524 4.654623 -3.918828
H 1.027694 3.444297 1.803310
H 1.046744 5.395591 3.306905
H 0.636849 6.488631 1.968481
H 2.203379 5.648360 1.980879
H -1.113045 4.126891 2.901116
H -1.554644 5.064609 1.457676
H -1.400285 3.300203 1.352490
H 2.365555 1.627896 -2.284125
H 3.831327 3.276849 -3.409068
H 3.434728 1.919828 -4.494706
H 2.634977 3.488761 -4.705707
H -0.005300 1.229400 -3.095084
H 1.191070 0.721366 -4.299808
H 0.314798 2.256347 -4.490619
H 1.120160 -3.993264 4.172558
H -0.611097 -3.087657 5.700151
H -1.274463 -0.710182 5.540817
H -0.282901 1.486319 2.785026
H -2.441690 0.816501 3.952543
H -1.746925 1.399731 5.488037
H -2.009776 2.525862 4.146555
H 0.385857 3.183792 4.519944
H 0.841857 1.848747 5.605798
H 1.704250 2.091073 4.063567
H 2.974514 -1.879165 1.686845
H 1.263589 -4.415523 1.604220
H 2.441110 -3.899627 0.370424
H 0.955590 -2.971228 0.643330
H 4.081332 -2.973956 3.669220
H 3.147216 -4.450622 3.353495
H 4.311011 -3.874128 2.146060
H -4.021473 -1.638596 -3.696632
H -4.395928 -0.418192 -2.444082
H -3.865946 0.085130 -4.069258
H -1.731271 -1.267316 -4.813657
H -0.023549 -2.261103 -5.700151
H 1.016665 -3.288673 -4.670049
H 1.500396 -1.633393 -5.021199
H -4.442518 2.902773 0.469497
H -4.671976 1.497659 2.495782
H -3.742645 -0.804796 2.517639
H -2.717979 1.743803 -2.663158
H -2.262090 4.166059 -2.511402
H -1.357694 3.273579 -1.281242
H -2.806010 4.185496 -0.819767
H -4.503278 3.287616 -3.359480
H -5.142069 3.368699 -1.705507
H -5.240271 1.846051 -2.614283
H -1.589252 -2.163199 -0.207103
H -4.447395 -2.990691 0.442829
H -3.179397 -4.058218 -0.216616
H -3.764200 -2.673537 -1.170009
H -1.263378 -2.018594 2.327072
H -1.548535 -3.663294 1.719327
H -2.847922 -2.783520 2.535715
H 4.890383 -3.346120 -2.035047
H 4.155161 -5.707176 -1.824996
H 1.736220 -6.246924 -1.821990
H -0.905424 -3.604978 -1.747803
H -1.772390 -5.685976 -0.755834
H -0.285079 -5.205268 0.097201
H -0.258163 -6.540294 -1.075362
H -0.892649 -4.303321 -4.105066
H -2.029502 -5.314440 -3.195392
H -0.432668 -5.962184 -3.649030
H 2.476914 -0.522238 -2.605026
H 3.841546 -1.297650 -4.514081
H 4.604321 0.096639 -3.703794
H 5.240271 -1.540708 -3.446626
H 4.937695 -1.162590 -0.866406
H 4.289709 0.461501 -1.191438
H 3.367773 -0.698953 -0.224142

145

$(BDI)_2$Ba withOut dispersion

Ba 0.0237190 -0.298309 -0.0597440
N -2.3278600 -1.252214 -1.1520700
N -0.5098630 1.247732 -2.2679500
N 0.5122620 0.302437 2.5712100
N 2.3644840 -1.589474 0.6856720
C -3.0435930 -2.589449 -3.0850480
C -2.2620510 -1.440519 -2.4718290
C -1.5543140 -0.650433 -3.4049860
C -0.9614880 0.645963 -3.3523940
C -0.8936660 1.321530 -4.7118570
C -3.3849650 -1.856961 -0.4289180
C -4.6810730 -1.261959 -0.4407140
C -5.6946980 -1.816823 0.3472280
C -5.4740610 -2.935914 1.1451180
C -4.2151480 -3.525021 1.1439920
C -3.1686230 -3.015733 0.3644020
C -5.0063890 -0.031138 -1.2797480
C -6.0968960 -0.311748 -2.3229810
C -5.4106760 1.162396 -0.4057600
C -1.8380230 -3.753111 0.3731030
C -1.9773260 -5.201007 -0.1155960
C -1.1679860 -3.716752 1.7508400
C 0.0189250 2.561117 -2.3102620
C 1.3455500 2.800801 -2.7625930
C 1.8557700 4.103432 -2.7060710
C 1.0947050 5.163725 -2.2245630
C -0.2056060 4.924938 -1.7902600
C -0.7632310 3.641716 -1.8223060
C -2.2127470 3.441226 -1.4092130
C -2.5498710 4.092487 -0.0652340
C -3.1565980 3.933831 -2.5152860
C 2.2350250 1.685366 -3.2939660
C 3.3870150 1.390077 -2.3290960
C 2.7723230 1.973556 -4.7011390
C 0.9704810 -0.367251 4.9089460
C 1.0310980 -0.580723 3.4057920
C 1.6916270 -1.786060 3.0361050
C 2.3668960 -2.204785 1.8696670

C	3.1359550	-3.507469	2.0326970
C	-0.0712460	1.510345	3.0243080
C	-1.3936830	1.536891	3.5475420
C	-1.9615280	2.769974	3.8904830
C	-1.2615780	3.962657	3.7386530
C	0.0355950	3.930824	3.2356600
C	0.6490260	2.726356	2.8730940
C	-2.2147780	0.269493	3.7419040
C	-3.3846900	0.207591	2.7555360
C	-2.7147610	0.105301	5.1824920
C	2.0947170	2.729946	2.4013220
C	3.0466840	2.894260	3.5945300
C	2.3765470	3.780043	1.3234750
C	3.4032070	-1.843835	-0.2408220
C	3.1949710	-2.661925	-1.3835150
C	4.2228810	-2.806617	-2.3247330
C	5.4561680	-2.188451	-2.1549850
C	5.6694970	-1.406063	-1.0228400
C	4.6702080	-1.210194	-0.0643820
C	4.9738270	-0.344378	1.1532590
C	5.6130810	0.999564	0.7857600
C	5.8594500	-1.082382	2.1676490
C	1.9121170	-3.454607	-1.5854620
C	2.1677180	-4.964811	-1.4805570
C	1.2091780	-3.121362	-2.9053080
H	-2.8761760	-3.520046	-2.5235100
H	-4.1252830	-2.395375	-3.0399320
H	-2.7699620	-2.752524	-4.1351600
H	-1.6636070	-1.011856	-4.4295110
H	-0.4735050	2.333064	-4.6623910
H	-1.9007640	1.380705	-5.1516950
H	-0.2799150	0.720782	-5.4014900
H	-6.6871520	-1.357063	0.3380650
H	-6.2804360	-3.350178	1.7556750
H	-4.0417650	-4.415630	1.7549030
H	-4.0912010	0.251669	-1.8198670
H	-6.2548530	0.570126	-2.9645600
H	-7.0621160	-0.549253	-1.8467560
H	-5.8366610	-1.156946	-2.9772700
H	-5.6134330	2.048261	-1.0283150
H	-6.3252860	0.950808	0.1715630
H	-4.6200800	1.425819	0.3106290
H	-1.1810330	-3.237814	-0.3486120
H	-2.4372790	-5.251361	-1.1138830
H	-0.9925930	-5.691816	-0.1690020
H	-2.6045980	-5.796591	0.5664660
H	-1.0191430	-2.690936	2.1224300
H	-0.1810950	-4.204005	1.7287750
H	-1.7789940	-4.240915	2.5029320
H	2.8759760	4.291706	-3.0517460
H	1.5122390	6.173238	-2.1914320
H	-0.8084890	5.759576	-1.4232130
H	-2.3681490	2.355403	-1.3065700
H	-1.8814690	3.746890	0.7380730
H	-2.4755670	5.191041	-0.1079580
H	-3.5832350	3.852143	0.2293210
H	-4.2091140	3.749541	-2.2470680
H	-3.0382440	5.017008	-2.6830100
H	-2.9569230	3.427055	-3.4714550
H	1.6228810	0.773310	-3.3611010
H	4.0660930	2.253766	-2.2451570
H	3.9794290	0.524185	-2.6619460
H	3.0232730	1.167039	-1.3140930
H	1.9613290	2.188461	-5.4125360
H	3.4560630	2.837487	-4.7063600
H	3.3381850	1.107959	-5.0810240
H	1.9824620	-0.416735	5.3381780
H	0.5207830	0.594877	5.1820920
H	0.3844320	-1.171975	5.3806510
H	1.8667150	-2.445678	3.8889280
H	3.4745710	-3.660626	3.0659380
H	4.0009940	-3.567494	1.3600770
H	2.4692900	-4.349138	1.7782030
H	-2.9785660	2.794991	4.2914920
H	-1.7240870	4.913510	4.0151920
H	0.5908570	4.866156	3.1280930
H	-1.5591140	-0.587120	3.5254260
H	-3.9333990	-0.743039	2.8408130
H	-3.0451750	0.292943	1.7121220
H	-4.0995110	1.027210	2.9318950
H	-3.2255270	-0.863321	5.3040750
H	-3.4379660	0.890820	5.4542730
H	-1.8901020	0.148199	5.9092710
H	2.2905220	1.736919	1.9657660
H	2.8911660	3.865241	4.0929340
H	4.0982090	2.848847	3.2689400
H	2.8904520	2.106678	4.3468890
H	1.6968630	3.683151	0.4629070
H	3.4079010	3.679556	0.9509600
H	2.2735220	4.806818	1.7095760
H	4.0584530	-3.438288	-3.2024720
H	6.2500330	-2.321923	-2.8943440
H	6.6426250	-0.927685	-0.8828940
H	4.0115450	-0.132007	1.6430670
H	5.7321810	1.623812	1.6853730
H	5.0020020	1.559250	0.0637440
H	6.6165450	0.874767	0.3480670
H	5.4050490	-2.023984	2.5047300
H	6.0348240	-0.457326	3.0582900
H	6.8424550	-1.325012	1.7308840
H	1.2367020	-3.189725	-0.7536070
H	2.6517490	-5.227994	-0.5288040
H	1.2245310	-5.529515	-1.5544740
H	2.8256850	-5.312824	-2.2929600
H	1.8307120	-3.400205	-3.7710440
H	0.2580910	-3.669422	-2.9949230
H	0.979070	-2.049061	-3.0047350

(BDI)Ae$^{\bullet}$ Radicals

73

(BDI)Be$^{\bullet}$ with dispersion

Be	-0.000001	0.000077	-0.452184
N	-1.327118	0.043465	0.510655
N	1.327118	-0.043445	0.510644
C	-2.489545	0.181037	2.668160
H	-3.144196	-0.687050	2.499147
H	-2.257052	0.250203	3.737703
H	-3.070431	1.064715	2.365032
C	-1.238979	0.073118	1.845089
C	0.000004	-0.000124	2.496865
H	0.000006	-0.000184	3.585838
C	1.238983	-0.073290	1.845074
C	2.489550	-0.181400	2.668115
H	3.070185	-1.065279	2.365083
H	3.144428	0.686485	2.498953
H	2.257084	-0.250378	3.737675
C	-2.619038	0.035739	-0.100803
C	-3.292232	-1.196080	-0.249488
C	-4.548696	-1.192697	-0.860455
H	-5.087583	-2.133142	-0.989611
C	-5.124085	-0.006630	-1.312988
H	-6.108105	-0.023458	-1.787804
C	-4.441669	1.195742	-1.165196
H	-4.893478	2.120981	-1.531256
C	-3.180937	1.241171	-0.558053
C	-2.624524	-2.490412	0.179037
H	-1.959903	-2.255693	1.025205
C	-3.604091	-3.559602	0.656133
H	-3.055621	-4.421860	1.065530
H	-4.232287	-3.939200	-0.164945
C	-1.734775	-3.011311	-0.953941
H	-2.342776	-3.282420	-1.831525
H	-1.168224	-3.900989	-0.636448
C	-2.436604	2.557361	-0.441849
H	-1.525956	2.367224	0.146541
C	-3.249009	3.622268	0.296625
H	-2.655157	4.540601	0.427029
H	-3.559289	3.274431	1.294068
C	-1.991685	3.038922	-1.825237
H	-2.859396	3.248960	-2.471018
H	-1.377374	2.270851	-2.320000
C	2.619032	-0.035702	-0.100829
C	3.180947	-1.241133	-0.558067
C	4.441674	-1.195697	-1.165211
H	4.893498	-2.120932	-1.531263
C	5.124071	0.006685	-1.313033
H	6.108083	0.023519	-1.787864
C	4.548678	1.192745	-0.860496
H	5.087561	2.133195	-0.989642
C	3.292221	1.196119	-0.249505
C	2.436611	-2.557319	-0.441857
H	1.526100	-2.367243	0.146768
C	1.991369	-3.038656	-1.825222
H	2.858948	-3.248643	-2.471198
H	1.377018	-2.270470	-2.319761
C	3.249136	-3.622370	0.296274
H	2.655242	-4.540670	0.426740
H	3.559721	-3.274666	1.293668
C	2.624555	2.490446	0.179111
H	1.959897	2.255672	1.025233
C	1.734871	3.011519	-0.953840
H	2.342908	3.282689	-1.831378
H	1.168361	3.901194	-0.636265
C	3.604163	3.559534	0.656353
H	3.055725	4.421781	1.065815
H	4.232412	3.939180	-0.164664
H	4.273410	3.176288	1.442366
H	1.015362	2.240773	-1.280300
H	-4.160806	3.896232	-0.257369
H	-1.395797	3.961790	-1.747446
H	4.160755	-3.896363	-0.257998
H	1.395439	-3.961497	-1.747454
H	-4.273392	-3.176476	1.442160
H	-1.015303	-2.240493	-1.280295

73

(BDI)Be$^{\bullet}$ withOut dispersion

Be	-0.000001	0.000034	-0.446909
N	1.345246	-0.000002	0.509319
N	-1.345246	0.000053	0.509321
C	2.489440	0.000226	2.692912
H	3.112117	0.879988	2.471900
H	2.240628	0.000178	3.761437
H	3.112431	-0.879306	2.471877
C	1.247508	0.000063	1.845167
C	0.000001	0.000002	2.489623
H	0.000002	-0.000021	3.579049
C	-1.247507	-0.000040	1.845169
C	-2.489438	-0.000254	2.692915
H	-3.112450	0.879268	2.471905
H	-3.112093	-0.880027	2.471884
H	-2.240622	-0.000228	3.761439
C	2.642357	-0.000051	-0.107919
C	3.264387	1.230357	-0.422799
C	4.520811	1.202046	-1.039899
H	5.013289	2.144325	-1.293998
C	5.151459	-0.000241	-1.343552
H	6.132759	-0.000322	-1.825133
C	4.520632	-1.202440	-1.039886
H	5.013003	-2.144789	-1.293940
C	3.264197	-1.230554	-0.422813
C	2.589083	2.569232	-0.162278
H	1.691439	2.373785	0.443939
C	3.473125	3.539639	0.628793
H	2.917147	4.461018	0.865013
H	4.368714	3.837594	0.060487
C	2.115234	3.194943	-1.480737
H	2.966647	3.424430	-2.141972
H	1.570305	4.134766	-1.295558
C	2.588665	-2.569318	-0.162306
H	1.690891	-2.373674	0.443653
C	3.472402	-3.539742	0.629077
H	2.916253	-4.461015	0.865298
H	3.815066	-3.101108	1.579342
C	2.115051	-3.195112	-1.480823
H	2.966589	-3.424800	-2.141822
H	1.444428	-2.509048	-2.021326
C	-2.642357	0.000071	-0.107916
C	-3.264253	1.230549	-0.422808
C	-4.520687	1.202378	-1.039881
H	-5.013100	2.144704	-1.293934

C -5.151459 0.000152 -1.343552
H -6.132757 0.000188 -1.825135
C -4.520756 -1.202109 -1.039903
H -5.013191 -2.144409 -1.294006
C -3.264331 -1.230361 -0.422802
C -2.588790 2.569349 -0.162306
H -1.690995 2.373758 0.443637
C -2.115237 3.195171 -1.480832
H -2.966801 3.424809 -2.141815
H -1.444584 2.509146 -2.021346
C -3.472571 3.539721 0.629089
H -2.916471 4.461025 0.865305
H -3.815198 3.101067 1.579358
C -2.588959 -2.569203 -0.162277
H -1.691334 -2.373705 0.443953
C -2.115052 -3.194881 -1.480731
H -2.966440 -3.424413 -2.141982
H -1.570076 -4.134677 -1.295545
C -3.472956 -3.539661 0.628780
H -2.916927 -4.461007 0.865009
H -4.368518 -3.837670 0.060459
H -3.815803 -3.101125 1.579026
H -1.444237 -2.508809 -2.020991
H 4.368068 -3.837873 0.060981
H 1.569955 -4.134845 -1.295672
H -4.368261 3.837805 0.061005
H -1.570192 4.134936 -1.295690
H 3.815929 3.101084 1.579046
H 1.444392 2.508908 -2.021012

73

(BDI)Mg• with dispersion

Mg -0.000034 -0.000031 -1.010978
N -1.476080 -0.000067 0.434643
N 1.476055 0.000010 0.434599
C -2.467110 -0.000564 2.667988
H -3.099945 -0.880105 2.475450
H -2.167690 -0.000197 3.722834
H -3.100924 0.878170 2.475057
C -1.271225 -0.000181 1.750115
C 0.000016 -0.000054 2.354228
H 0.000033 -0.000063 3.443542
C 1.271238 0.000094 1.750076
C 2.467157 0.000485 2.667904
H 3.100991 -0.878224 2.474918
H 3.099957 0.880051 2.475365
H 2.167779 0.000078 3.722760
C -2.806918 -0.000074 -0.066058
C -3.444586 -1.228282 -0.348099
C -4.716313 -1.204304 -0.929398
H -5.219370 -2.146301 -1.159868
C -5.350844 0.000035 -1.221120
H -6.343784 0.000099 -1.677152
C -4.716455 1.204326 -0.928828
H -5.219657 2.146350 -1.158872
C -3.444747 1.228205 -0.347516
C -2.745469 -2.546181 -0.067275
H -1.918500 -2.333494 0.627095
C -3.655990 -3.573021 0.605766
H -3.081913 -4.469259 0.888161
H -4.467623 -3.904038 -0.061039
C -2.126336 -3.106940 -1.351002
H -2.904692 -3.337903 -2.095636
H -1.557290 -4.028510 -1.150096
C -2.745761 2.546023 -0.066001
H -1.919198 2.333156 0.628795
C -3.656602 3.572771 0.606745
H -3.082640 4.468965 0.889521
H -4.119852 3.163371 1.517559
C -2.125901 3.107070 -1.349244
H -2.903876 3.338398 -2.094162
H -1.439189 2.379504 -1.815810
C 2.806883 0.000071 -0.066132
C 3.444784 -1.228177 -0.347573
C 4.716505 -1.204230 -0.928855
H 5.219766 -2.146226 -1.158880
C 5.350834 0.000096 -1.221135
H 6.343787 0.000084 -1.677139
C 4.716243 1.204402 -0.929405
H 5.219266 2.146425 -1.159841
C 3.444503 1.228311 -0.348132
C 2.745885 -2.546037 -0.066023
H 1.919285 -2.333202 0.628740
C 2.126110 -3.107190 -1.349261
H 2.904129 -3.338501 -2.094138
H 1.439366 -2.379696 -1.815894
C 3.656778 -3.572690 0.606795
H 3.082871 -4.468911 0.889595
H 4.119975 -3.163217 1.517601
C 2.745330 2.546170 -0.067253
H 1.918340 2.333405 0.627068
C 2.126230 3.106993 -1.350968
H 2.904608 3.338041 -2.095551
H 1.557138 4.028527 -1.150019
C 3.655784 3.573000 0.605891
H 3.081661 4.469198 0.888321
H 4.467430 3.904092 -0.060861
H 4.118601 3.163661 1.516952
H 1.439651 2.379399 -1.817688
H -4.467924 3.903890 -0.060384
H -1.556774 4.028465 -1.147775
H 4.468140 -3.903784 -0.060299
H 1.557039 -4.028615 -1.147767
H -4.118827 -3.163722 1.516834
H -1.439709 -2.379337 -1.817637

73

(BDI)Mg• withOut dispersion

Mg -0.000000 0.000020 -1.000497
N 1.504273 -0.000023 0.426503
N -1.504273 0.000008 0.426503
C 2.458458 -0.000059 2.686456
H 3.096717 0.879529 2.513353
H 2.134628 -0.000094 3.734456
H 3.096743 -0.879617 2.513302
C 1.280761 -0.000046 1.740502
C -0.000000 -0.000046 2.332579
H -0.000000 -0.000070 3.422278
C -1.280761 -0.000019 1.740502
C -2.458458 -0.000058 2.686457
H -3.096748 0.879504 2.513344
H -3.096711 -0.879642 2.513313
H -2.134627 -0.000070 3.734457
C 2.839187 -0.000023 -0.077213
C 3.481916 1.229596 -0.368597
C 4.757383 1.201698 -0.944982

H	5.260454	2.144231	-1.177289
C	5.396913	-0.000012	-1.232571
H	6.392396	-0.000008	-1.683962
C	4.757354	-1.201729	-0.945069
H	5.260401	-2.144257	-1.177448
C	3.481888	-1.229638	-0.368683
C	2.809252	2.569928	-0.103658
H	1.896165	2.368360	0.477349
C	3.680266	3.515021	0.731976
H	3.125058	4.434141	0.978666
H	4.591407	3.819983	0.192797
C	2.376792	3.237559	-1.415960
H	3.246246	3.470138	-2.052006
H	1.840762	4.180522	-1.220617
C	2.809185	-2.569974	-0.103860
H	1.896118	-2.368434	0.477189
C	3.680187	-3.515183	0.731655
H	3.124951	-4.434308	0.978267
H	3.996449	-3.048992	1.677588
C	2.376670	-3.237455	-1.416219
H	3.246101	-3.470000	-2.052309
H	1.709461	-2.583058	-2.001461
C	-2.839188	0.000031	-0.077213
C	-3.481878	1.229656	-0.368662
C	-4.757343	1.201765	-0.945052
H	-5.260382	2.144301	-1.177419
C	-5.396913	0.000059	-1.232571
H	-6.392395	0.000070	-1.683962
C	-4.757394	-1.201661	-0.944999
H	-5.260473	-2.144186	-1.177318
C	-3.481926	-1.229578	-0.368616
C	-2.809161	2.569982	-0.103829
H	-1.896101	2.368430	0.477225
C	-2.376630	3.237465	-1.416182
H	-3.246054	3.470022	-2.052277
H	-1.709424	2.583064	-2.001421
C	-3.680160	3.515198	0.731684
H	-3.124915	4.434315	0.978304
H	-3.996434	3.049007	1.677613
C	-2.809275	-2.569919	-0.103690
H	-1.896183	-2.368364	0.477315
C	-2.376830	-3.237548	-1.415998
H	-3.246290	-3.470114	-2.052040
H	-1.840808	-4.180517	-1.220663
C	-3.680295	-3.515008	0.731945
H	-3.125095	-4.434135	0.978627
H	-4.591442	-3.819957	0.192770
H	-3.996503	-3.048710	1.677843
H	-1.709607	-2.583238	-2.001322
H	4.591302	-3.820125	0.192421
H	1.840610	-4.180419	-1.220960
H	-4.591267	3.820152	0.192445
H	-1.840562	4.180422	-1.220915
H	3.996485	3.048722	1.677870
H	1.709573	2.583246	-2.001284

73

(BDI)Ca• with dispersion

Ca	0.013304	-0.411637	-1.294944
N	-1.433872	-0.312691	0.515385
N	1.452851	-0.114456	0.493716
C	-2.427224	-0.154663	2.757423
H	-3.330592	-0.611666	2.332449
H	-2.656488	0.908120	2.936556
H	-2.208879	-0.612737	3.731328
C	-1.242638	-0.271275	1.827522
C	0.029536	-0.272484	2.431964
H	0.037201	-0.285534	3.522201
C	1.286619	-0.152849	1.810149
C	2.490579	-0.073850	2.718276
H	3.261034	0.594741	2.310010
H	2.951759	-1.069914	2.813890
H	2.211894	0.264310	3.724443
C	-2.720747	-0.156798	-0.041968
C	-3.292783	1.133289	-0.162525
C	-4.532620	1.259804	-0.794684
H	-4.986124	2.249324	-0.892643
C	-5.195174	0.150405	-1.312232
H	-6.164195	0.269548	-1.802588
C	-4.611153	-1.109979	-1.218018
H	-5.129976	-1.972750	-1.639969
C	-3.372632	-1.288238	-0.594518
C	-2.552060	2.367464	0.320761
H	-1.703338	2.028771	0.932580
C	-3.420308	3.275551	1.192424
H	-2.821278	4.101574	1.606485
H	-4.245450	3.726002	0.618546
C	-1.967231	3.137841	-0.866963
H	-1.370559	3.998094	-0.525113
H	-1.312903	2.495945	-1.481375
C	-2.748025	-2.666394	-0.467262
H	-1.651373	-2.521631	-0.431609
C	-3.131294	-3.327971	0.859487
H	-2.663322	-4.320910	0.950490
H	-2.806832	-2.723772	1.717452
C	-3.046505	-3.581745	-1.651499
H	-4.107264	-3.875893	-1.688015
H	-2.793193	-3.096367	-2.606749
C	2.715050	0.136867	-0.085933
C	2.967370	1.437084	-0.596874
C	4.181062	1.679010	-1.245864
H	4.394004	2.675937	-1.636164
C	5.124033	0.666923	-1.408406
H	6.067179	0.874346	-1.919393
C	4.853749	-0.612188	-0.932339
H	5.589500	-1.406525	-1.081056
C	3.655125	-0.903468	-0.273880
C	1.942645	2.542251	-0.404114
H	0.948821	2.058312	-0.381969
C	1.929252	3.565231	-1.536290
H	2.844210	4.178096	-1.548756
H	1.833100	3.076882	-2.518360
C	2.104697	3.222987	0.957989
H	1.347186	4.011605	1.091194
H	1.989013	2.505974	1.781826
C	3.360656	-2.320735	0.181321
H	2.452318	-2.283848	0.801110
C	3.050871	-3.219505	-1.019685
H	3.916227	-3.290800	-1.697789
H	2.786418	-4.238061	-0.693837
C	4.486699	-2.908185	1.033927
H	4.195973	-3.891746	1.434793
H	5.407585	-3.053432	0.447412
H	4.735174	-2.252312	1.881930
H	2.205867	-2.825123	-1.609945

H	-4.223473	-3.456577	0.927415
H	-2.456279	-4.507529	-1.573581
H	3.100551	3.686238	1.045206
H	1.080435	4.254711	-1.415182
H	-3.865330	2.721398	2.032539
H	-2.762836	3.513681	-1.529700

73

(BDI)Ca• withOut dispersion

Ca	0.000018	-0.000582	-1.292124
N	-1.505920	-0.000126	0.483820
N	1.505919	-0.000190	0.483842
C	-2.456921	-0.000095	2.744803
H	-3.095186	-0.879883	2.573377
H	-3.095335	0.879532	2.573120
H	-2.133088	0.000074	3.792879
C	-1.280992	-0.000155	1.794830
C	-0.000009	-0.000244	2.387077
H	-0.000015	-0.000305	3.476960
C	1.280982	-0.000220	1.794850
C	2.456888	-0.000264	2.744851
H	3.095300	0.879387	2.573289
H	3.095158	-0.880029	2.573337
H	2.133029	-0.000212	3.792919
C	-2.829430	0.000024	-0.034126
C	-3.468044	1.230646	-0.348127
C	-4.724216	1.201590	-0.965067
H	-5.221167	2.144298	-1.210326
C	-5.354142	0.000359	-1.274585
H	-6.334182	0.000492	-1.758430
C	-4.724543	-1.201029	-0.965055
H	-5.221746	-2.143610	-1.210295
C	-3.468369	-1.230419	-0.348109
C	-2.823652	2.575820	-0.037271
H	-1.881286	2.370773	0.494467
C	-3.692879	3.436063	0.888696
H	-3.165662	4.363858	1.162896
H	-4.639488	3.726737	0.405429
C	-2.471734	3.343222	-1.318187
H	-1.955008	4.287279	-1.081233
H	-1.810323	2.754884	-1.977196
C	-2.824385	-2.575786	-0.037218
H	-1.881912	-2.371004	0.494439
C	-3.693835	-3.435670	0.888875
H	-3.166905	-4.363613	1.163129
H	-3.947766	-2.905708	1.819561
C	-2.472827	-3.343407	-1.318097
H	-3.374315	-3.591576	-1.901365
H	-1.811226	-2.755365	-1.977181
C	2.829421	0.000010	-0.034125
C	3.468015	1.230647	-0.348062
C	4.724188	1.201635	-0.965023
H	5.221124	2.144364	-1.210232
C	5.354115	0.000434	-1.274622
H	6.334153	0.000596	-1.758473
C	4.724521	-1.200982	-0.965162
H	5.221726	-2.143544	-1.210473
C	3.468360	-1.230416	-0.348208
C	2.823644	2.575819	-0.037130
H	1.881226	2.370756	0.494513
C	2.471854	3.343362	-1.317993
H	3.373264	3.591857	-1.901241
H	1.810457	2.755112	-1.977091
C	3.692843	3.435921	0.888995
H	3.165667	4.363726	1.163242
H	3.946875	2.906017	1.819686
C	2.824378	-2.575791	-0.037373
H	1.881866	-2.371027	0.494227
C	2.472910	-3.343429	-1.318268
H	3.374442	-3.591637	-1.901452
H	1.956488	-4.287647	-1.081293
C	3.693781	-3.435672	0.888774
H	3.166854	-4.363634	1.162966
H	4.640550	-3.726035	0.405632
H	3.947633	-2.905725	1.819490
H	1.811377	-2.755393	-1.977422
H	-4.640563	-3.726073	0.405680
H	-1.956444	-4.287641	-1.081102
H	4.639513	3.726567	0.405831
H	1.955159	4.287419	-1.080970
H	-3.947021	2.906259	1.819415
H	-3.373099	3.591686	-1.901520

73

(BDI)Sr• with dispersion

Sr	0.061870	-0.462834	-1.308193
N	1.487029	-0.011555	0.706423
N	-1.417010	0.028172	0.634975
C	2.451308	0.446120	2.926795
H	3.415175	0.399688	2.403774
H	2.381151	1.407915	3.456842
H	2.432715	-0.342771	3.694890
C	1.282386	0.264354	1.982060
C	0.000748	0.390881	2.553823
H	-0.021248	0.606757	3.623262
C	-1.253657	0.317504	1.917848
C	-2.456501	0.638961	2.775547
H	-2.634124	1.726549	2.769597
H	-3.369818	0.159240	2.399464
H	-2.291096	0.339158	3.819056
C	2.739015	0.050772	0.079222
C	3.308286	1.311029	-0.255222
C	4.465123	1.328486	-1.038297
H	4.920161	2.283161	-1.305552
C	5.054710	0.150251	-1.494426
H	5.954704	0.193563	-2.111991
C	4.493664	-1.077640	-1.162226
H	4.964292	-1.996895	-1.516121
C	3.338276	-1.152664	-0.376890
C	2.657826	2.599791	0.228654
H	2.409598	2.456960	1.291973
C	3.578105	3.815139	0.150005
H	3.099249	4.680932	0.631875
H	3.792009	4.101460	-0.892070
C	1.335004	2.884361	-0.491722
H	0.921581	3.856308	-0.180905
H	0.579095	2.126710	-0.235522
C	2.765689	-2.490567	0.057262
H	1.682438	-2.339552	0.227767
C	3.350609	-2.905032	1.411603
H	2.899718	-3.846382	1.763517
H	3.172590	-2.134833	2.173985
C	2.926840	-3.600279	-0.978134
H	3.981494	-3.887606	-1.112177
H	2.529788	-3.298374	-1.960736

C	-2.688459	-0.027125	0.034013
C	-3.325953	1.150651	-0.428969
C	-4.533136	1.033533	-1.124283
H	-5.033248	1.936801	-1.483308
C	-5.106633	-0.209583	-1.374229
H	-6.050170	-0.279533	-1.920410
C	-4.465266	-1.365538	-0.936161
H	-4.914984	-2.338505	-1.143811
C	-3.256410	-1.301275	-0.237427
C	-2.703542	2.517435	-0.211691
H	-1.826389	2.377784	0.437174
C	-2.203960	3.107916	-1.533438
H	-3.036362	3.279755	-2.234139
H	-1.492902	2.431137	-2.036682
C	-3.657688	3.482755	0.494829
H	-3.148181	4.430647	0.729006
H	-4.037429	3.056261	1.435108
C	-2.574890	-2.560452	0.269617
H	-1.495847	-2.329708	0.350236
C	-2.727346	-3.757180	-0.666250
H	-3.765432	-4.123298	-0.701546
H	-2.101112	-4.593493	-0.319345
C	-3.039043	-2.902922	1.688746
H	-2.524955	-3.802790	2.061899
H	-4.123694	-3.096253	1.705489
H	-2.831161	-2.081713	2.387828
H	-2.420746	-3.506595	-1.694329
H	4.439294	-3.054536	1.331894
H	2.383841	-4.502194	-0.656512
H	-4.528783	3.724611	-0.134472
H	-1.693070	4.069589	-1.369323
H	4.538146	3.634135	0.656711
H	1.471889	2.909204	-1.584810

73

(BDI)Sr• withOut dispersion

Sr	-0.008182	0.053471	-1.382396
N	-1.519466	0.004790	0.610005
N	1.508553	-0.035700	0.595242
C	-2.437457	-0.118617	2.898274
H	-3.400382	0.131478	2.435037
H	-2.514946	-1.136817	3.312879
H	-2.261271	0.559222	3.746085
C	-1.287604	-0.051899	1.912900
C	-0.000053	-0.079025	2.497185
H	0.000960	-0.116894	3.587578
C	1.278983	-0.074967	1.904614
C	2.454548	-0.119700	2.856095
H	3.081335	-1.002131	2.658249
H	3.104579	0.756423	2.713233
H	2.131063	-0.148133	3.904021
C	-2.821304	0.028810	0.056946
C	-3.493985	-1.185277	-0.254821
C	-4.724174	-1.124580	-0.920183
H	-5.248491	-2.054153	-1.158839
C	-5.296049	0.090753	-1.283520
H	-6.257820	0.114378	-1.802055
C	-4.631236	1.275888	-0.983602
H	-5.084080	2.229193	-1.268563
C	-3.396939	1.274473	-0.323068
C	-2.919961	-2.543848	0.125536
H	-1.959883	-2.361040	0.632969
C	-3.827389	-3.289702	1.112616
H	-3.354881	-4.229101	1.442029
H	-4.794998	-3.550533	0.654085
C	-2.629232	-3.410593	-1.106118
H	-2.161038	-4.363595	-0.811390
H	-1.946076	-2.904756	-1.809345
C	-2.724853	2.598150	0.019018
H	-1.698229	2.361594	0.345461
C	-3.422929	3.286528	1.200178
H	-2.898057	4.214859	1.478016
H	-3.453320	2.637720	2.087733
C	-2.623704	3.546034	-1.180848
H	-3.613796	3.882287	-1.527766
H	-2.118237	3.068518	-2.036594
C	2.822781	-0.030384	0.066962
C	3.444560	-1.254474	-0.309709
C	4.686344	-1.214540	-0.954915
H	5.168152	-2.152676	-1.244450
C	5.322246	-0.009299	-1.235503
H	6.290571	-0.001055	-1.742221
C	4.712717	1.185447	-0.865503
H	5.215217	2.131663	-1.085042
C	3.471756	1.204607	-0.217907
C	2.803055	-2.605951	-0.019664
H	1.850581	-2.407740	0.496906
C	2.480545	-3.377801	-1.305513
H	3.393043	-3.623918	-1.872115
H	1.827045	-2.798135	-1.979897
C	3.665124	-3.462178	0.917497
H	3.144423	-4.397763	1.177601
H	3.897531	-2.935072	1.855104
C	2.859990	2.544620	0.172543
H	1.906139	2.329692	0.679757
C	2.546776	3.413181	-1.052577
H	3.461087	3.679792	-1.606853
H	2.054107	4.351801	-0.751517
C	3.744286	3.314318	1.162565
H	3.243252	4.238144	1.493597
H	4.704653	3.604938	0.706985
H	3.972280	2.716456	2.057844
H	1.876908	2.897480	-1.762018
H	-4.462319	3.551839	0.946212
H	-2.050055	4.447338	-0.911914
H	4.623277	-3.737344	0.447859
H	1.965914	-4.324632	-1.075336
H	-4.038386	-2.684209	2.006560
H	-3.549611	-3.650500	-1.662452

73

(BDI)Ba• with dispersion

Ba	0.057252	-0.612313	-1.332600
N	1.498320	0.025737	0.831556
N	-1.433526	0.069414	0.757963
C	2.444888	0.605727	3.029204
H	3.409926	0.574409	2.506911
H	2.348236	1.574990	3.540799
H	2.448854	-0.168978	3.812162
C	1.281366	0.369442	2.088039
C	-0.001918	0.522144	2.648241
H	-0.024473	0.794710	3.704957
C	-1.259175	0.432556	2.017570
C	-2.449100	0.828177	2.864589
H	-2.507749	1.926408	2.929490
H	-3.395795	0.468155	2.441318

H	-2.343654	0.448562	3.890547
C	2.738097	0.101074	0.189707
C	3.269962	1.364584	-0.198435
C	4.402002	1.385032	-1.017312
H	4.824803	2.342282	-1.325363
C	5.005781	0.209091	-1.459917
H	5.882927	0.255615	-2.109215
C	4.489266	-1.021415	-1.069004
H	4.975593	-1.939178	-1.404860
C	3.363747	-1.100851	-0.240992
C	2.613335	2.654042	0.274999
H	2.391186	2.527671	1.345704
C	3.516842	3.878718	0.155003
H	3.038506	4.746197	0.634378
H	3.701851	4.151426	-0.896229
C	1.271287	2.910264	-0.418043
H	0.842172	3.871223	-0.094179
H	0.540371	2.131716	-0.155082
C	2.855438	-2.437303	0.271614
H	1.766692	-2.330327	0.442519
C	3.465640	-2.744006	1.643426
H	3.061282	-3.683629	2.051824
H	3.253720	-1.940475	2.360960
C	3.067878	-3.598702	-0.695213
H	4.134743	-3.845111	-0.813272
H	2.659407	-3.379771	-1.695348
C	-2.690416	0.024601	0.134507
C	-3.275021	1.195724	-0.413640
C	-4.452556	1.076034	-1.158846
H	-4.910145	1.974682	-1.580856
C	-5.049257	-0.161290	-1.379036
H	-5.966375	-0.232744	-1.968218
C	-4.465817	-1.310648	-0.850656
H	-4.936892	-2.279439	-1.027639
C	-3.291538	-1.245305	-0.094885
C	-2.647714	2.562064	-0.207618
H	-1.762541	2.422365	0.429987
C	-2.169053	3.166090	-1.530298
H	-3.009720	3.340779	-2.220386
H	-1.456859	2.500464	-2.045911
C	-3.599404	3.518164	0.516684
H	-3.094547	4.469804	0.745982
H	-3.962081	3.086224	1.460692
C	-2.686944	-2.492711	0.526753
H	-1.599611	-2.311574	0.620941
C	-2.879662	-3.754636	-0.310487
H	-3.934406	-4.069901	-0.344926
H	-2.306034	-4.588211	0.122732
C	-3.206090	-2.694874	1.953969
H	-2.744542	-3.582222	2.415153
H	-4.298604	-2.838128	1.951739
H	-2.980326	-1.828394	2.589355
H	-2.534676	-3.611850	-1.347241
H	4.559842	-2.845934	1.564436
H	2.565150	-4.502671	-0.319084
H	-4.481107	3.752239	-0.100888
H	-1.664369	4.130243	-1.362264
H	4.491070	3.717775	0.641109
H	1.387331	2.943326	-1.513326

73

(BDI)Ba• withOut dispersion

Ba	-0.000116	-0.267496	-1.421727
N	1.511387	0.046390	0.745893
N	-1.511345	0.046337	0.745984
C	2.436766	0.402575	3.006798
H	3.408847	0.192529	2.542494
H	2.453972	1.438206	3.382310
H	2.311894	-0.251892	3.882216
C	1.283122	0.221395	2.038368
C	0.000043	0.276355	2.625140
H	0.000084	0.414846	3.708639
C	-1.283063	0.221359	2.038453
C	-2.436701	0.402577	3.006868
H	-2.454022	1.438270	3.382209
H	-3.408761	0.192381	2.542581
H	-2.311773	-0.251741	3.882386
C	2.792725	0.025717	0.161105
C	3.419251	1.235363	-0.257077
C	4.616560	1.163057	-0.979413
H	5.103805	2.089061	-1.297442
C	5.202191	-0.057414	-1.300953
H	6.136216	-0.088938	-1.867508
C	4.587010	-1.238226	-0.895315
H	5.053336	-2.194980	-1.143286
C	3.388844	-1.227443	-0.170911
C	2.839932	2.602189	0.084876
H	1.893807	2.430008	0.621132
C	3.767415	3.387671	1.022330
H	3.296732	4.334937	1.331294
H	4.720365	3.638183	0.528513
C	2.513449	3.427816	-1.165474
H	2.050494	4.388188	-0.886953
H	1.810205	2.900439	-1.831999
C	2.784213	-2.540154	0.312215
H	1.735163	-2.329569	0.583637
C	3.483591	-3.028209	1.589171
H	3.009479	-3.948194	1.967713
H	3.442764	-2.274361	2.388228
C	2.779041	-3.641413	-0.752044
H	3.796729	-3.974927	-1.010656
H	2.289880	-3.311548	-1.683552
C	-2.792645	0.025699	0.161125
C	-3.419136	1.235365	-0.257065
C	-4.616257	1.163086	-0.979719
H	-5.103455	2.089105	-1.297773
C	-5.201764	-0.057379	-1.301518
H	-6.135622	-0.088890	-1.868347
C	-4.586677	-1.238205	-0.895777
H	-5.052940	-2.194953	-1.143891
C	-3.388692	-1.227447	-0.171070
C	-2.840009	2.602169	0.085257
H	-1.894027	2.429996	0.621759
C	-2.513228	3.428048	-1.164832
H	-3.416520	3.654363	-1.754022
H	-1.809686	2.900891	-1.831225
C	-3.767819	3.387411	1.022612
H	-3.297194	4.334551	1.332049
H	-4.007668	2.815114	1.930768
C	-2.784239	-2.540153	0.312296
H	-1.735240	-2.329588	0.583913
C	-2.778937	-3.641543	-0.751833
H	-3.796598	-3.975055	-1.010550
H	-2.233257	-4.524940	-0.384159
C	-3.483937	-3.028012	1.589153

H	-3.010002	-3.948011	1.967887
H	-4.545934	-3.250844	1.394604
H	-3.443206	-2.274094	2.388153
H	-2.289626	-3.311813	-1.683312
H	4.545608	-3.251132	1.394833
H	2.233279	-4.524838	-0.384559
H	-4.720567	3.638148	0.528519
H	-2.050494	4.388442	-0.886015
H	4.006897	2.815667	1.930764
H	3.416915	3.654169	-1.754378

6-Ae Side-On

222

$[K(^{DIPeP}NN)Ca]_2(N_2)$ (6-Ca) [side-on]

Ca	-0.03334500	2.32432600	0.02892600
K	3.19783800	0.05055800	-0.01663800
Si	-1.68359300	5.15251200	-1.43454600
N	-1.84354800	3.62233000	-0.63500100
N	0.62065700	0.00833000	0.03151500
C	-3.04662800	3.10186700	-0.20780800
C	-4.03554600	2.59829300	-1.12419600
C	-5.22347700	2.03404900	-0.65293400
H	-5.95678900	1.66015600	-1.37173000
C	-5.48551300	1.90373300	0.71182700
H	-6.41244900	1.44463000	1.06164700
C	-4.54182500	2.38749300	1.61544300
H	-4.74885900	2.31957700	2.68749400
C	-3.35583100	3.00016300	1.19256800
C	-3.73651700	2.59202800	-2.60780100
H	-3.05177000	3.42692600	-2.79331300
C	-4.95931100	2.79526200	-3.50466800
H	-4.62464600	2.77294500	-4.55395500
H	-5.65075800	1.93897400	-3.40502600
C	-5.69950500	4.10201400	-3.25614300
H	-6.55743800	4.21159100	-3.93710000
H	-5.03474700	4.96513500	-3.41533700
H	-6.07744300	4.16635900	-2.22478300
C	-2.95031800	1.31861300	-2.96871600
H	-2.10932300	1.23293800	-2.25822200
H	-3.59837300	0.43463300	-2.81290200
C	-2.39343000	1.29090100	-4.38518200
H	-3.19059800	1.26537800	-5.14232200
H	-1.76556200	0.40304600	-4.54828200
H	-1.77651600	2.18114700	-4.58145400
C	-2.49558300	3.68549400	2.23815800
H	-1.62639200	4.09922200	1.70327400
C	-3.27989500	4.88008200	2.81486400
H	-3.70105400	5.44024800	1.96475300
H	-4.15037200	4.49673300	3.37574200
C	-2.47922700	5.82962900	3.69560900
H	-3.10507800	6.67290400	4.02555600
H	-1.61788500	6.24791500	3.15494300
H	-2.09236600	5.33942500	4.60195400
C	-1.96955900	2.74799700	3.33114700
H	-2.79815400	2.14932100	3.74623200
H	-1.58486700	3.34880400	4.16976600
C	-0.86290800	1.82834500	2.84697900
H	-1.19558700	1.19714400	2.00556700
H	-0.51569800	1.13838900	3.62687900
H	0.02866100	2.41094300	2.54955500
C	-0.17697400	6.11381100	-0.81048500
H	0.72423000	5.74594400	-1.32525000
H	-0.32569000	7.13421500	-1.20766100
C	-1.39411800	5.10498200	-3.31672200
H	-0.64372200	4.34452400	-3.58457800
H	-0.99523700	6.08072700	-3.63934400
H	-2.29881200	4.90977000	-3.91143200
C	-3.21101400	6.20144000	-1.08095700
H	-4.13825800	5.63376700	-1.24871300
H	-3.23748700	7.10338200	-1.71269200
H	-3.20625800	6.52454000	-0.02787000
Si	1.56684800	5.23999300	1.34133600
N	1.76004100	3.67751900	0.61250400
C	2.98561700	3.17493300	0.22491900
C	3.94982500	2.68689700	1.17372300
C	5.16597400	2.14858400	0.74489700
H	5.87931600	1.78797400	1.49053800
C	5.48179600	2.03203100	-0.60930600
H	6.43140100	1.59644000	-0.92696800
C	4.56006800	2.49775300	-1.54507800
H	4.80569300	2.43675600	-2.60938400
C	3.34605000	3.08202800	-1.16340000
C	3.60799600	2.67252500	2.64726000
H	2.83690300	3.43660700	2.79461400
C	4.78272100	3.01343600	3.56811100
H	4.41661500	3.01864900	4.60719300
H	5.53923700	2.20842100	3.53219500
C	5.43416100	4.35682800	3.27149400
H	6.25018900	4.56959200	3.97897800
H	4.70238000	5.17536900	3.34993500
H	5.85249700	4.38975000	2.25444900
C	2.94361500	1.33339300	3.01282300
H	2.10514500	1.17326400	2.31178800
H	3.66234100	0.50842800	2.84571800
C	2.40882400	1.25751700	4.43621100
H	3.21706000	1.27810800	5.18163600
H	1.84189000	0.32979100	4.60001300
H	1.73893300	2.10499000	4.64998100
C	2.49558300	3.72955800	-2.23908900
H	1.62359100	4.15840000	-1.72195500
C	3.27605800	4.89824800	-2.86987800
H	3.73957600	5.47209700	-2.05172800
H	4.11673200	4.49146800	-3.45932100
C	2.44913000	5.83802400	-3.73703000
H	3.07328500	6.65371400	-4.13294000
H	1.63065900	6.29579400	-3.16219000
H	1.99726300	5.32649600	-4.60060300
C	1.97692300	2.74565800	-3.29472300
H	2.81237700	2.13836100	-3.68360400
H	1.59030700	3.31014300	-4.15767100
C	0.87691200	1.83536800	-2.77945000
H	1.21243700	1.23646900	-1.91607000
H	0.53689800	1.11574300	-3.53526200
H	-0.01852100	2.42085000	-2.50159100
C	0.02076400	6.13073400	0.70836900
H	-0.86904300	5.74444900	1.22887000
H	0.13688300	7.16283800	1.08584600
C	1.33156100	5.26102000	3.23091300
H	0.61093900	4.49126900	3.55019900
H	0.91937900	6.23792100	3.53268200
H	2.26007100	5.10894800	3.80072100
C	3.05021400	6.31931600	0.90097600
H	3.99835300	5.78458200	1.06048800
H	3.06955400	7.24489900	1.49779200

H	3.00312200	6.60216400	-0.16254800
N	-0.62065700	-0.00833000	0.03151500
K	-3.19783800	-0.05055800	-0.01663800
Ca	0.03334500	-2.32432600	0.02892600
Si	1.68359300	-5.15251200	-1.43454600
N	1.84354800	-3.62233000	-0.63500100
C	3.04662800	-3.10186700	-0.20780800
C	4.03554600	-2.59829300	-1.12419600
C	5.22347700	-2.03404900	-0.65293400
H	5.95678900	-1.66015600	-1.37173000
C	5.48551300	-1.90373300	0.71182700
H	6.41244900	-1.44463000	1.06164700
C	4.54182500	-2.38749300	1.61544300
H	4.74885900	-2.31957700	2.68749400
C	3.35583100	-3.00016300	1.19256800
C	3.73651700	-2.59202800	-2.60780100
H	3.05177000	-3.42692600	-2.79331300
C	4.95931100	-2.79526200	-3.50466800
H	4.62464600	-2.77294500	-4.55395500
H	5.65075800	-1.93897400	-3.40502600
C	5.69950500	-4.10201400	-3.25614300
H	6.55743800	-4.21159100	-3.93710000
H	5.03474700	-4.96513500	-3.41533700
H	6.07744300	-4.16635900	-2.22478300
C	2.95031800	-1.31861300	-2.96871600
H	2.10932300	-1.23293800	-2.25822200
H	3.59837300	-0.43463300	-2.81290200
C	2.39343000	-1.29090100	-4.38518200
H	3.19059800	-1.26537800	-5.14232200
H	1.76556200	-0.40304600	-4.54828200
H	1.77651600	-2.18114700	-4.58145400
C	2.49558300	-3.68549400	2.23815800
H	1.62639200	-4.09922200	1.70327400
C	3.27989500	-4.88008200	2.81486400
H	3.70105400	-5.44024800	1.96475300
H	4.15037200	-4.49673300	3.37574200
C	2.47922700	-5.82962900	3.69560900
H	3.10507800	-6.67290400	4.02555600
H	1.61788500	-6.24791500	3.15494300
H	2.09236600	-5.33942500	4.60195400
C	1.96955900	-2.74799700	3.33114700
H	2.79815400	-2.14932100	3.74623200
H	1.58486700	-3.34880400	4.16976600
C	0.86290800	-1.82834500	2.84697900
H	1.19558700	-1.19714400	2.00556700
H	0.51569800	-1.13838900	3.62687900
H	-0.02866100	-2.41094300	2.54955500
C	0.17697400	-6.11381100	-0.81048500
H	-0.72423000	-5.74594400	-1.32525000
H	0.32569000	-7.13421500	-1.20766100
C	1.39411800	-5.10498200	-3.31672200
H	0.64372200	-4.34452400	-3.58457800
H	0.99523700	-6.08072700	-3.63934400
H	2.29881200	-4.90977000	-3.91143200
C	3.21101400	-6.20144000	-1.08095700
H	4.13825800	-5.63376700	-1.24871300
H	3.23748700	-7.10338200	-1.71269200
H	3.20625800	-6.52454000	-0.02787000
Si	-1.56684800	-5.23999300	1.34133600
N	-1.76004100	-3.67751900	0.61250400
C	-2.98561700	-3.17493300	0.22491900
C	-3.94982500	-2.68689700	1.17372300
C	-5.16597400	-2.14858400	0.74489700
H	-5.87931600	-1.78797400	1.49053800
C	-5.48179600	-2.03203100	-0.60930600
H	-6.43140100	-1.59644000	-0.92696800
C	-4.56006800	-2.49775300	-1.54507800
H	-4.80569300	-2.43675600	-2.60938400
C	-3.34605000	-3.08202800	-1.16340000
C	-3.60799600	-2.67252500	2.64726000
H	-2.83690300	-3.43660700	2.79461400
C	-4.78272100	-3.01343600	3.56811100
H	-4.41661500	-3.01864900	4.60719300
H	-5.53923700	-2.20842100	3.53219500
C	-5.43416100	-4.35682800	3.27149400
H	-6.25018900	-4.56959200	3.97897800
H	-4.70238000	-5.17536900	3.34993500
H	-5.85249700	-4.38975000	2.25444900
C	-2.94361500	-1.33339300	3.01282300
H	-2.10514500	-1.17326400	2.31178800
H	-3.66234100	-0.50842800	2.84571800
C	-2.40882400	-1.25751700	4.43621100
H	-3.21706000	-1.27810800	5.18163600
H	-1.84189000	-0.32979100	4.60001300
H	-1.73893300	-2.10499000	4.64998100
C	-2.49558300	-3.72955800	-2.23908900
H	-1.62359100	-4.15840000	-1.72195500
C	-3.27605800	-4.89824800	-2.86987800
H	-3.73957600	-5.47209700	-2.05172800
H	-4.11673200	-4.49146800	-3.45932100
C	-2.44913000	-5.83802400	-3.73703000
H	-3.07328500	-6.65371400	-4.13294000
H	-1.63065900	-6.29579400	-3.16219000
H	-1.99726300	-5.32649600	-4.60060300
C	-1.97692300	-2.74565800	-3.29472300
H	-2.81237700	-2.13836100	-3.68360400
H	-1.59030700	-3.31014300	-4.15767100
C	-0.87691200	-1.83536800	-2.77945000
H	-1.21243700	-1.23646900	-1.91607000
H	-0.53689800	-1.11574300	-3.53526200
H	0.01852100	-2.42085000	-2.50159100
C	-0.02076400	-6.13073400	0.70836900
H	0.86904300	-5.74444900	1.22887000
H	-0.13688300	-7.16283800	1.08584600
C	-1.33156100	-5.26102000	3.23091300
H	-0.61093900	-4.49126900	3.55019900
H	-0.91937900	-6.23792100	3.53268200
H	-2.26007100	-5.10894800	3.80072100
C	-3.05021400	-6.31931600	0.90097600
H	-3.99835300	-5.78458200	1.06048800
H	-3.06955400	-7.24489900	1.49779200
H	-3.00312200	-6.60216400	-0.16254800

222

[K(^{DIPeP}NN)Sr]$_2$(N$_2$) (6-Sr) [side-on]

C	4.13486600	6.11364900	3.00830300
C	5.27763700	1.66498600	3.02834500
C	6.07490500	-2.40110900	3.45156800
C	6.32363800	3.43488900	0.75257900
C	2.88243700	5.29579000	3.29351800
C	1.66647200	2.56124700	4.22468700
C	5.05744600	-3.30857000	2.77310800
C	5.97865300	0.34150000	0.50269400
C	2.74830400	4.05419300	2.41248000
C	1.56930700	3.15501800	2.82646000
C	6.59187800	-2.96199600	-0.69913400

C	5.90261200	0.03437600	-0.99985900
C	2.92447900	-1.99741900	3.20554400
C	2.64568400	4.33225200	0.92879000
C	3.87031900	-2.56193000	2.13736900
C	2.03036800	5.49600300	0.46322700
C	3.08537000	3.31694800	0.00907600
C	5.37887200	3.64780400	-3.05261800
C	2.00123600	-0.90219900	2.70005100
C	3.18290600	-3.44766100	1.11905100
C	1.79860600	5.71684300	-0.89651600
C	2.81692500	3.55557400	-1.38380400
C	2.56864700	-4.62269900	1.56639100
C	5.49122500	-1.81765100	-3.31346600
C	-0.95781200	-3.14492200	4.52339200
C	2.19081900	4.73339400	-1.80331600
C	4.06757100	3.05697000	-3.54626800
C	3.25343400	-3.14814500	-0.28687300
C	3.18702700	2.51308500	-2.41665900
C	-1.94509100	0.12708400	3.56850900
C	-1.77370000	1.48348100	2.89847400
C	-1.87959400	5.54850600	1.18494600
C	-2.15220700	4.44373200	1.99066300
C	2.02856000	-5.55630800	0.68266200
C	-1.09048500	-3.45237900	3.03814000
C	4.37693500	-5.68471800	-3.12446600
C	2.68799100	-4.12199500	-1.17847100
C	-3.95606200	2.58349900	3.62274900
C	-3.07340000	2.16502300	2.44031100
C	2.10563300	-5.29276600	-0.68506200
C	-2.28523600	5.50098900	-0.14893500
C	-2.79310500	3.30847000	1.48992100
C	1.92981900	1.80920900	-2.95590500
C	2.65307200	-3.84788600	-2.66537300
C	2.16534700	0.49703400	-3.69339500
C	-5.29173200	3.17066100	3.19209400
C	-2.89475900	-5.21986200	3.42509600
C	3.04578900	-5.05870200	-3.51906000
C	-2.46005900	-4.00094400	2.60113800
C	-2.93072500	4.38721700	-0.69567200
C	-3.20043200	3.22412200	0.10848500
C	1.28231600	-3.26461200	-3.05185800
C	-1.83832000	-5.40746300	0.61866600
C	-2.47060300	-4.26106100	1.11041900
C	-2.11126800	4.11421400	-3.06691700
C	-4.20934400	-5.83991300	2.97059300
C	-5.34076900	-2.03834900	3.25364500
C	-1.72065000	2.64331700	-3.08977000
C	-3.31304100	4.42755300	-2.15852200
C	-3.99574400	5.74595000	-2.55543200
C	-1.76957300	-5.68312300	-0.74697800
C	1.12676400	-2.90139500	-4.52202600
C	-3.08571300	-3.31975600	0.21716800
C	-2.37591900	-4.79122200	-1.63201300
C	-1.96703300	-1.08882700	-2.79021200
C	-5.83322000	-0.21728400	0.94336400
C	-3.03561500	-3.64024300	-1.18645100
C	-4.84545100	5.63093300	-3.81400200
C	-6.50915600	3.16673800	-0.68660300
C	-5.89945300	0.11108700	-0.55414100
C	-2.86641700	-2.20632400	-3.28837700
C	-3.77664700	-2.79653600	-2.20289500
C	-6.43078800	-3.23163700	0.64924500
C	-5.29268200	1.56071400	-3.02084000
C	-4.94475700	-3.59056800	-2.81491700
C	-6.00522000	-2.72083600	-3.47699200
H	4.20265000	6.99127900	3.66935600
H	5.04067600	5.50700700	3.16554100
H	6.26291800	1.24666900	3.29337600
H	7.28272400	3.37722400	1.29127400
H	6.89678900	-2.98600000	3.89205800
H	5.13107800	2.56911500	3.63775900
H	2.90186800	4.97254100	4.34663900
H	5.62896700	-1.80275700	4.26119200
H	6.51652200	-1.69542200	2.73127300
H	4.15900100	6.47183800	1.96814300
H	5.82603600	4.37543300	1.03648500
H	4.52028000	0.92476500	3.33339400
H	1.62144500	3.33192500	5.00845700
H	7.02396700	0.31420300	0.85563400
H	1.98047000	5.92752100	3.20285200
H	2.61125500	2.01200400	4.35220000
H	6.53530000	3.48666300	-0.32544100
H	3.65417000	3.45391600	2.56031100
H	4.67232900	-4.05134500	3.49460500
H	0.84378400	1.85578200	4.41489800
H	3.52007700	-1.59484900	4.04036900
H	6.77257300	-2.75159700	0.36576900
H	5.55866100	-3.89138900	1.98495700
H	5.48545200	-0.45820600	1.08307900
H	6.87454500	0.21169300	-1.49049300
H	7.55902900	-2.91826700	-1.22471700
H	4.28765500	-1.71844300	1.56710000
H	0.63125700	3.73222900	2.73065300
H	1.70226200	6.24888700	1.18344400
H	5.20097600	4.46846000	-2.34161500
H	2.57550800	-0.00150800	2.41688800
H	1.51551300	2.32705200	2.09805300
H	6.20522300	-3.99071000	-0.77730800
H	5.98035100	2.88524600	-2.53444200
H	2.32480100	-2.81846000	3.63400200
H	5.21859000	0.73339100	-1.51507500
H	5.98091400	4.04159900	-3.88528600
H	1.28806900	-0.56964500	3.46707000
H	-0.01583500	-2.61660700	4.73602300
H	6.48261500	-1.44605000	-3.62251300
H	2.53430000	-4.82461600	2.64113600
H	-1.24396600	2.16626000	3.58202200
H	1.39754000	-1.23240300	1.83782700
H	-1.37470200	6.42854600	1.58769700
H	-0.97092400	-0.30281000	3.83793400
H	3.80754300	1.78066300	-1.87320000
H	-1.86936100	4.46901300	3.04725000
H	1.31757900	6.63542100	-1.23853200
H	-0.96240300	-4.06088500	5.13210300
H	-1.78625300	-2.51018800	4.87384900
H	-2.54145900	0.19605700	4.48946200
H	5.38078100	-2.83419600	-3.71862900
H	3.50125200	3.81236300	-4.11960600
H	-3.40520700	3.30813100	4.24831500
H	4.73480300	-1.18060300	-3.79889300
H	-1.08569600	1.38243100	2.03653500
H	5.19033100	-4.94395500	-3.16318200
H	4.27894000	2.23987700	-4.25606500
H	2.01211900	4.88815700	-2.87158500
H	4.34940900	-6.08095800	-2.09875600
H	-0.29662800	-4.16464100	2.74548300

H	1.56373800	-6.47318400	1.05046500
H	-0.90878700	-2.52089100	2.47232600
H	-4.13292300	1.70417700	4.26419600
H	-2.45612900	-0.61195100	2.92502800
H	4.64559500	-6.51108600	-3.80021100
H	-2.08368000	6.36078300	-0.79341500
H	3.38620200	-3.05288600	-2.84961900
H	2.67993600	-0.26162100	-3.07566400
H	1.22061200	1.63130500	-2.12333100
H	-2.98751400	-4.91244600	4.47943000
H	-2.09755500	-5.98573100	3.41209800
H	-5.89701500	3.46945900	4.06107700
H	1.39486500	2.51062400	-3.61768400
H	2.79092500	0.63207300	-4.58737900
H	1.68316000	-6.01293800	-1.39090000
H	-3.68375400	1.44993000	1.86323200
H	-5.14853600	4.05575000	2.55427600
H	-3.18436400	-3.19595500	2.77777800
H	-1.25520400	4.74082100	-2.75362500
H	-1.37799100	-6.10270200	1.32644000
H	3.09765100	-4.73936100	-4.57253100
H	-4.61168600	-1.35760700	3.72129100
H	2.24706100	-5.82218800	-3.48408800
H	1.12412600	-2.35443700	-2.44405100
H	-5.87374000	2.43717200	2.61355000
H	1.21349700	0.05367500	-4.01808600
H	-1.54754800	2.23512700	-2.08197400
H	-3.23600500	6.53594800	-2.69185400
H	-4.50430500	-6.67307800	3.62678100
H	-0.81661400	2.47163100	-3.69100600
H	-5.17821300	-3.04153200	3.67373100
H	1.95455700	-2.26004800	-4.86240300
H	0.48521400	-3.97695900	-2.76761700
H	-4.62998200	6.08157300	-1.71868100
H	-1.26618600	-6.58076300	-1.11199500
H	-6.34603700	-1.70916200	3.56605600
H	-5.02409100	-5.10018700	2.98410200
H	-1.35706100	-1.40535500	-1.92747800
H	-5.18474300	0.50267200	1.47512400
H	-2.34241300	4.42869500	-4.09850400
H	-4.13964300	-6.22589000	1.94299300
H	-4.04354000	3.62696100	-2.31983400
H	1.11481300	-3.79412600	-5.16426600
H	-2.53003600	2.03857900	-3.52001100
H	0.18527900	-2.36037200	-4.70040500
H	-6.13153800	4.15916000	-0.97796400
H	-1.26216700	-0.73630800	-3.55671500
H	-6.67871500	3.18630900	0.40061200
H	-2.56587500	-0.20358800	-2.51182000
H	-6.81847100	-0.08254900	1.42089700
H	-5.28578300	6.60083400	-4.09188600
H	-4.25965300	5.27800200	-4.67760500
H	-2.35443500	-5.00697200	-2.70465300
H	-2.25048600	-3.00990000	-3.72648400
H	-4.21882200	-1.96455900	-1.63420700
H	-5.31109400	-0.62386400	-1.13390000
H	-5.67342000	4.91986000	-3.66550300
H	-6.00842300	-4.24739800	0.70535400
H	-7.47894500	3.00998800	-1.18493700
H	-4.56362700	0.78638800	-3.31183000
H	-7.38857000	-3.22747200	1.19344600
H	-5.08472000	2.45879100	-3.61966900
H	-6.92509400	-0.01833400	-0.94002300
H	-3.48611300	-1.81923600	-4.11305500
H	-6.63928700	-3.01361800	-0.40906800
H	-5.41088600	-4.18738400	-2.01589400
H	-6.28752400	1.18877900	-3.31802500
H	-4.54619300	-4.32172300	-3.54092000
H	-6.45072400	-2.02237900	-2.75165400
H	-5.59625700	-2.11636000	-4.30160600
H	-6.81918600	-3.33353200	-3.89382400
K	-0.07564900	3.32616300	-0.10639300
K	0.12708600	-3.16569900	0.05538300
N	3.66666200	2.14722400	0.42329200
N	3.78573300	-1.96395100	-0.74053300
N	-0.01691600	0.67775100	-0.12194800
N	0.01873200	-0.56362200	-0.04939900
N	-3.75286300	2.07227600	-0.37672800
N	-3.65392800	-2.14768200	0.66464100
Si	5.22222900	1.95858500	1.14681300
Si	5.35647200	-1.73364300	-1.41878700
Si	-5.22500800	-1.96712300	1.35732700
Si	-5.27916800	1.81000600	-1.13168900
Sr	2.46140600	0.09321600	-0.30394800
Sr	-2.45928800	-0.01971300	0.15864100

6-Ae End-On

222

[K(^{DIPeP}NN)Ca]$_2$(N$_2$) (6-Ca) [end-on]

Ca	-2.88023800	-0.00151800	0.00067400
K	0.00051500	2.66037600	0.00084300
Si	-5.54046700	-1.80190600	-1.30220100
N	-3.95152900	-1.91821000	-0.63649400
N	-0.61141900	-0.00247800	-0.00134800
C	-3.28965200	-3.08048100	-0.29010100
C	-2.55759800	-3.84764100	-1.25518700
C	-1.85022900	-4.98980500	-0.86712000
H	-1.30058000	-5.55608400	-1.62400100
C	-1.82557300	-5.42413900	0.45886500
H	-1.26885500	-6.31978300	0.74203500
C	-2.53400000	-4.69211700	1.41130700
H	-2.53376900	-5.02837400	2.45269100
C	-3.26626500	-3.54893200	1.06817100
C	-2.52515600	-3.40331000	-2.70137500
H	-3.34995200	-2.68851000	-2.81709700
C	-2.75186100	-4.55246800	-3.69099200
H	-2.83125200	-4.12758000	-4.70474700
H	-1.86263600	-5.20888500	-3.71731700
C	-3.99630200	-5.38171000	-3.40215200
H	-4.15175500	-6.14989100	-4.17507600
H	-4.89684500	-4.74930000	-3.37408700
H	-3.92762000	-5.88923300	-2.42884700
C	-1.23659900	-2.61538000	-2.99485600
H	-1.18799500	-1.77269500	-2.28074300
H	-0.35852600	-3.25956700	-2.79541900
C	-1.13266800	-2.05790600	-4.40805800
H	-1.00169400	-2.85325500	-5.15618600
H	-0.27414500	-1.37626100	-4.50371400
H	-2.04056600	-1.49623400	-4.67833900
C	-4.07412400	-2.85201100	2.14236300
H	-4.61613500	-2.04363000	1.62952300
C	-5.13112400	-3.79168300	2.74752000
H	-5.55186200	-4.40568900	1.93591400
H	-4.64156300	-4.50162700	3.43795100
C	-6.26022700	-3.06343300	3.46483900
H	-6.99460700	-3.77256700	3.87652400
H	-6.79530500	-2.39061800	2.77608900
H	-5.89491700	-2.44865300	4.30229000
C	-3.19431700	-2.21613000	3.22809800
H	-2.50500100	-2.97909000	3.62938500
H	-3.82683200	-1.90507200	4.07513000
C	-2.39651800	-1.01291700	2.75386500
H	-1.76429400	-1.25980100	1.88118600
H	-1.71058200	-0.63847000	3.52480600
H	-3.06090300	-0.16359700	2.50922400
C	-6.27049800	-0.14132200	-0.75720400
H	-5.71909300	0.64117900	-1.30925100
H	-7.29180400	-0.08673300	-1.17079200
C	-5.63647900	-1.72092200	-3.19800200
H	-4.95038700	-0.95317300	-3.59230600
H	-6.65756700	-1.43756400	-3.50365800
H	-5.39511600	-2.67202700	-3.69453700
C	-6.62709400	-3.22361900	-0.71193000
H	-6.12448400	-4.18951600	-0.88062200
H	-7.59316700	-3.24226100	-1.24078900
H	-6.83017300	-3.13576000	0.36609800
Si	-5.53962000	1.80112300	1.30079200
N	-3.95012000	1.91661400	0.63611600
C	-3.28810100	3.07942500	0.29138400
C	-2.55501900	3.84455900	1.25725000
C	-1.84744600	4.98713100	0.87065900
H	-1.29669200	5.55162000	1.62807000
C	-1.82404100	5.42414700	-0.45443200
H	-1.26719700	6.32009300	-0.73636600
C	-2.53366600	4.69423000	-1.40758600
H	-2.53437500	5.03245700	-2.44832800
C	-3.26583200	3.55053300	-1.06596900
C	-2.52224800	3.39866500	2.70294900
H	-3.34575500	2.68215500	2.81758400
C	-2.75167500	4.54674300	3.69333700
H	-2.83258500	4.12079100	4.70652700
H	-1.86291200	5.20369700	3.72183000
C	-3.99601700	5.37567300	3.40318500
H	-4.15343100	6.14256400	4.17699400
H	-4.89618400	4.74288500	3.37228400
H	-3.92561300	5.88475100	2.43082100
C	-1.23215100	2.61332200	2.99671900
H	-1.18210100	1.76997700	2.28348000
H	-0.35528600	3.25884200	2.79635400
C	-1.12683900	2.05748500	4.41047900
H	-0.99655400	2.85385700	5.15763100
H	-0.26739800	1.37706900	4.50656000
H	-2.03388100	1.49495800	4.68187500
C	-4.07398600	2.85528400	-2.14097200
H	-4.61714700	2.04723700	-1.62888200
C	-5.12943900	3.79629900	-2.74669600
H	-5.54972900	4.41108000	-1.93544000
H	-4.63877500	4.50541200	-3.43720100
C	-6.25918300	3.06924600	-3.46423300
H	-6.99242400	3.77913900	-3.87664200
H	-6.79546500	2.39744500	-2.77542300
H	-5.89424700	2.45356200	-4.30118600
C	-3.19405400	2.21890100	-3.22633000
H	-2.50332500	2.98124200	-3.62638100
H	-3.82621300	1.90937900	-4.07420000
C	-2.39841700	1.01419100	-2.75237500
H	-1.76669500	1.25916000	-1.87880000
H	-1.71224300	0.63945100	-3.52296600
H	-3.06426400	0.16568400	-2.50893700
C	-6.27155300	0.14197500	0.75382800
H	-5.72271300	-0.64169500	1.30674400
H	-7.29372200	0.08930500	1.16553900
C	-5.63605800	1.71781800	3.19645600
H	-4.95100400	0.94858600	3.58967900
H	-6.65750200	1.43546700	3.50184200
H	-5.39327800	2.66790700	3.69422300
C	-6.62432200	3.22478300	0.71170300
H	-6.12024400	4.18979900	0.88106000
H	-7.59039800	3.24450800	1.24049200
H	-6.82743100	3.13801600	-0.36640700
N	0.61289100	-0.00228800	-0.00281200
K	0.00166100	-2.66490900	-0.00240400
Ca	2.88163800	-0.00046300	-0.00284100
Si	5.53864800	1.80308600	-1.30409600
N	3.94981900	1.91891800	-0.63802300
C	3.28773900	3.08144100	-0.29270700
C	2.55393400	3.84665600	-1.25794800
C	1.84660000	4.98919800	-0.87074500
H	1.29572600	5.55391900	-1.62787000
C	1.82413100	5.42603900	0.45440300

H	1.26762400	6.32201800	0.73692300
C	2.53433200	4.69591300	1.40700300
H	2.53564200	5.03401800	2.44778800
C	3.26631400	3.55230200	1.06476600
C	2.51980500	3.40020400	-2.70347500
H	3.34506900	2.68592600	-2.81932500
C	2.74425900	4.54821600	-3.69495800
H	2.82315500	4.12196500	-4.70817800
H	1.85425400	5.20357600	-3.72138700
C	3.98801400	5.37933400	-3.40856400
H	4.14208900	6.14620500	-4.18306200
H	4.88924400	4.74793100	-3.37994700
H	3.91953600	5.88861900	-2.43617100
C	1.23147800	2.61084500	-2.99422600
H	1.18492000	1.76847700	-2.27957400
H	0.35313700	3.25428700	-2.79365300
C	1.12580900	2.05256600	-4.40698800
H	0.99207800	2.84738200	-5.15519000
H	0.26830100	1.36942300	-4.50078300
H	2.03416100	1.49234500	-4.67875900
C	4.07509700	2.85683300	2.13915200
H	4.61793000	2.04886700	1.62658500
C	5.13088100	3.79759500	2.74459700
H	5.55117700	4.41217100	1.93318300
H	4.64054400	4.50691800	3.43511200
C	6.26054700	3.07030900	3.46201800
H	6.99419200	3.78003600	3.87399500
H	6.79637900	2.39809400	2.77326100
H	5.89562400	2.45502600	4.29926900
C	3.19545100	2.22027000	3.22461800
H	2.50539100	2.98280100	3.62546200
H	3.82776900	1.90975900	4.07200800
C	2.39878400	1.01643300	2.75014400
H	1.76879000	1.26207400	1.87552600
H	1.71102700	0.64302000	3.51996700
H	3.06370500	0.16692500	2.50766500
C	6.26890200	0.14271500	-0.75856700
H	5.71722800	-0.64019100	-1.30983800
H	7.29002700	0.08759000	-1.17250000
C	5.63433600	1.72171500	-3.19986600
H	4.94906600	0.95308200	-3.59387800
H	6.65568500	1.43957500	-3.50577800
H	5.39163400	2.67243700	-3.69647200
C	6.62490400	3.22527600	-0.71435400
H	6.12210100	4.19094000	-0.88384800
H	7.59124900	3.24387700	-1.24270400
H	6.82741600	3.13813700	0.36383700
Si	5.54145200	-1.79950400	1.30082700
N	3.95210200	-1.91715600	0.63635400
C	3.29025000	-3.08026900	0.29281600
C	2.55715400	-3.84442400	1.25942900
C	1.84937800	-4.98723000	0.87392000
H	1.29872400	-5.55102000	1.63193200
C	1.82564400	-5.42532000	-0.45081500
H	1.26863800	-6.32142500	-0.73193300
C	2.53541600	-4.69645700	-1.40471100
H	2.53591600	-5.03563000	-2.44514600
C	3.26783800	-3.55261400	-1.06414100
C	2.52370100	-3.39650800	2.70444900
H	3.34734000	-2.68006600	2.81860800
C	2.75215400	-4.54321000	3.69666000
H	2.83231600	-4.11584300	4.70931000
H	1.86328600	-5.20001300	3.72538000
C	3.99663600	-5.37264600	3.40856400
H	4.15347900	-6.13848200	4.18353200
H	4.89685500	-4.73993800	3.37740800
H	3.92688200	-5.88305600	2.43684900
C	1.23356900	-2.61048700	2.99604000
H	1.18369800	-1.76849600	2.28120000
H	0.35677200	-3.25632100	2.79637400
C	1.12757200	-2.05211800	4.40877600
H	0.99624400	-2.84707600	5.15725300
H	0.26851800	-1.37098200	4.50301300
H	2.03476800	-1.48966200	4.67982200
C	4.07650900	-2.85873800	-2.13965800
H	4.61927500	-2.04989100	-1.62839600
C	5.13257200	-3.80052100	-2.74311200
H	5.55291500	-4.41329800	-1.93037600
H	4.64242500	-4.51137100	-3.43219200
C	6.26218000	-3.07450400	-3.46190600
H	6.99632500	-3.78493700	-3.87176600
H	6.79740000	-2.40030100	-2.77460900
H	5.89733800	-2.46152700	-4.30088200
C	3.19726500	-2.22403700	-3.22654400
H	2.50690200	-2.98700000	-3.62603900
H	3.82996800	-1.91560800	-4.07439900
C	2.40113000	-1.01868300	-2.75491100
H	1.76900400	-1.26230800	-1.88123600
H	1.71531200	-0.64525300	-3.52647200
H	3.06675100	-0.16970200	-2.51248900
C	6.27049600	-0.13950400	0.75268100
H	5.71981900	0.64339400	1.30489500
H	7.29248800	-0.08414500	1.16445000
C	5.63824200	-1.71523000	3.19638900
H	4.95365700	-0.94552000	3.58947700
H	6.65993200	-1.43320600	3.50128200
H	5.39525300	-2.66499500	3.69467000
C	6.62804800	-3.22206300	0.71249800
H	6.12484800	-4.18759200	0.88157600
H	7.59373200	-3.24077300	1.24204600
H	6.83195000	-3.13517700	-0.36545300

222

[K(^{DIPeP}NN)Sr]$_2$(N$_2$) (6-Sr) [end-on]

C	4.03224900	6.34633700	2.38565900
C	5.69319400	2.36448400	2.69804000
C	6.30104500	-2.23401400	3.78861000
C	6.61240600	3.54730200	0.03676200
C	2.88331600	5.47432400	2.87441000
C	1.80439600	2.83563100	4.11755600
C	5.25453300	-3.14487300	3.16001200
C	6.11927300	0.48648700	0.46174300
C	2.75364400	4.15902300	2.10508500
C	1.65014000	3.24055900	2.65868700
C	7.01944500	-2.89594900	-0.16645700
C	6.10857300	-0.05362100	-0.97994500
C	3.21364500	-1.63623600	3.38598100
C	2.55479400	4.32545600	0.61440800
C	4.13529200	-2.39367900	2.41951300
C	1.74556400	5.35216400	0.12109500
C	3.12946500	3.35535000	-0.27478600
C	5.18624200	3.86354100	-3.56821000
C	2.34639400	-0.58643500	2.71000800
C	3.38014200	-3.32216300	1.49059300
C	1.45633200	5.47506600	-1.23963200
C	2.79076400	3.47825900	-1.66803600

C	2.63039600	-4.36605900	2.04262800
C	5.95083400	-2.31942300	-2.96737800
C	-1.39306100	-3.67874100	4.31997200
C	1.97830000	4.52488900	-2.11757400
C	3.95057000	3.04171400	-3.89770600
C	3.47605200	-3.16976400	0.06234800
C	3.27394200	2.44092600	-2.66127000
C	-2.19453400	-0.26990800	3.58947700
C	-1.94155000	1.10746600	2.99098300
C	-1.77430300	5.32371300	1.69030500
C	-2.11050100	4.17078100	2.39893600
C	1.96379700	-5.29800800	1.24551800
C	-1.38398100	-3.76306400	2.80009400
C	4.38804800	-6.04273600	-2.40616600
C	2.78141300	-4.13495500	-0.74023500
C	-3.99765800	2.30938500	3.91903300
C	-3.19631200	1.93586300	2.66575100
C	2.05034400	-5.16570900	-0.14118400
C	-2.22138900	5.44092100	0.37431400
C	-2.85378600	3.14133800	1.81465700
C	2.12267800	1.49249800	-3.04960600
C	2.81813400	-4.02738600	-2.24969500
C	2.53518600	0.18827800	-3.72073400
C	-5.30972200	3.00602000	3.59085100
C	-3.14604200	-5.61496700	2.73328600
C	3.13395000	-5.36125500	-2.93714700
C	-2.68613700	-4.26783400	2.15827000
C	-2.97468000	4.44056600	-0.25099500
C	-3.30466500	3.22760200	0.44747900
C	1.51826600	-3.38669300	-2.76552400
C	-1.76590800	-5.28444400	0.05495900
C	-2.56134100	-4.30044200	0.65109200
C	-2.22084300	4.43498000	-2.64867900
C	-4.38560000	-6.18219400	2.05440900
C	-5.76110600	-2.54453100	2.90049300
C	-1.86932200	2.96420200	-2.83552400
C	-3.40098300	4.65982100	-1.68683100
C	-4.06249300	6.03075900	-1.89983300
C	-1.61691600	-5.37293500	-1.32932700
C	1.42149700	-3.21921300	-4.27538400
C	-3.25924000	-3.33672300	-0.15094500
C	-2.29392200	-4.44917800	-2.12690900
C	-2.11319700	-0.74267400	-2.85676600
C	-6.03587100	-0.33732400	0.88630000
C	-3.10664400	-3.45229000	-1.57864200
C	-4.93052300	6.09850300	-3.14924800
C	-6.73762000	3.25440300	-0.24994900
C	-6.05075900	0.21108200	-0.55027600
C	-2.99536000	-1.79466800	-3.50851500
C	-3.88614200	-2.55007400	-2.51365000
C	-6.82257100	-3.22436900	0.10719200
C	-5.52767300	2.00543000	-2.79347900
C	-5.00333500	-3.33847100	-3.21850900
C	-6.07764000	-2.46433900	-3.85191700
H	4.12724500	7.26349000	2.98696300
H	4.98928400	5.80415700	2.44953300
H	6.70118800	2.01305400	2.97528500
H	7.60985800	3.55108500	0.50433500
H	7.10232900	-2.81792500	4.26673900
H	5.58117800	3.38383800	3.09714700
H	3.04026200	5.24060900	3.93978400
H	5.87379800	-1.57427300	4.55961700
H	6.76822700	-1.58675100	3.02915800
H	3.89664500	6.64241000	1.33474400
H	6.17227600	4.54997800	0.16078000
H	4.96538800	1.71767000	3.21504500
H	1.71321800	3.69559000	4.79736400
H	7.13792900	0.41881800	0.87903600
H	1.93277500	6.03654700	2.83657800
H	2.78620800	2.37102000	4.29873200
H	6.73620200	3.37221400	-1.04173400
H	3.69445000	3.61259800	2.23426400
H	4.80917500	-3.79893600	3.93087800
H	1.03110800	2.10817000	4.40899300
H	3.82003100	-1.14495700	4.16355000
H	7.09944200	-2.53507300	0.86951100
H	5.74432800	-3.82053600	2.44153800
H	5.53721500	-0.17904000	1.13056900
H	7.08225800	0.13157000	-1.46347200
H	8.01444900	-2.82134800	-0.63370000
H	4.62884000	-1.65609000	1.76787600
H	0.66924300	3.72884500	2.51607500
H	1.31637800	6.07260500	0.82120000
H	4.94913800	4.67042700	-2.85860500
H	2.95572000	0.26657700	2.35880200
H	1.65321600	2.32240000	2.04394400
H	6.73965900	-3.96126600	-0.12951900
H	5.96169900	3.23454700	-3.10473400
H	2.57139800	-2.35934400	3.91694900
H	5.40995500	0.51897600	-1.62586700
H	5.62022000	4.31831200	-4.47140500
H	1.60023000	-0.15453200	3.39170400
H	-0.48088900	-3.18736500	4.69297400
H	6.95273400	-2.00340700	-3.30296200
H	2.57814100	-4.46245400	3.13155800
H	-1.30255700	1.69397800	3.67239200
H	1.78411000	-1.00763700	1.85796500
H	-1.18833500	6.11836200	2.15691300
H	-1.24727500	-0.79750900	3.76738300
H	4.05244100	1.86567700	-2.12804100
H	-1.79305500	4.07041700	3.44138300
H	0.83141700	6.29252100	-1.60514200
H	-1.44001800	-4.67463700	4.78441900
H	-2.25858400	-3.10221900	4.68281100
H	-2.72633300	-0.21332200	4.55021100
H	5.85406300	-3.39436200	-3.18186300
H	3.21974500	3.65861600	-4.44954300
H	-3.37820000	2.94882800	4.57204300
H	5.20910400	-1.78888300	-3.58632000
H	-1.32835300	0.99901700	2.07438900
H	5.26248300	-5.37810100	-2.48452000
H	4.22765600	2.22560600	-4.58553100
H	1.74767300	4.59976000	-3.18441800
H	4.28171100	-6.31326300	-1.34534100
H	-0.53926600	-4.39686200	2.47571900
H	1.39757500	-6.11679600	1.69496700
H	-1.19141000	-2.75022900	2.40283500
H	-4.20424400	1.39490500	4.49950900
H	-2.80437600	-0.91767800	2.93362300
H	4.61431300	-6.96049900	-2.97031300
H	-1.96976300	6.34152200	-0.19261200
H	3.62877200	-3.32517300	-2.48644100
H	3.18945300	-0.44063700	-3.08903700
H	1.50798200	1.26673400	-2.15395600
H	-3.34866000	-5.48767500	3.80936800
H	-2.32083200	-6.34801600	2.67158900

H	-5.86154500	3.27679200	4.50348900
H	1.42902500	2.03934400	-3.71101800
H	3.09314200	0.36233900	-4.65204400
H	1.53453600	-5.88852400	-0.77894100
H	-3.89417500	1.33363400	2.05751500
H	-5.13572700	3.92427900	3.01005300
H	-3.45953400	-3.52186100	2.38412700
H	-1.34816800	4.99034100	-2.26349300
H	-1.25136600	-6.00727800	0.69456300
H	3.25372000	-5.17800500	-4.01730200
H	-5.03753700	-1.96109700	3.49303500
H	2.27015900	-6.04550600	-2.84887600
H	1.43112400	-2.38922300	-2.29420600
H	-5.95877800	2.35254600	2.98702800
H	1.65533100	-0.41999400	-3.96985600
H	-1.91712200	2.41519700	-1.88198900
H	-3.28770000	6.81651700	-1.94323000
H	-4.71121500	-7.11760800	2.53471100
H	-0.87163800	2.82720400	-3.28049100
H	-5.61988300	-3.60554000	3.15419200
H	2.30490300	-2.70036100	-4.67952800
H	0.65392600	-3.98195100	-2.41958600
H	-4.67768900	6.26590300	-1.01582200
H	-0.99751700	-6.15259700	-1.77803700
H	-6.77224500	-2.25604600	3.23319500
H	-5.22552500	-5.47243100	2.10275700
H	-1.53443200	-1.15757700	-2.01411700
H	-5.36794300	0.26199500	1.53807800
H	-2.44763900	4.87644400	-3.63356400
H	-4.20014000	-6.39273400	0.99079000
H	-4.15471500	3.89846000	-1.91855200
H	1.34398300	-4.18874500	-4.78901200
H	-2.59833500	2.46596800	-3.49021700
H	0.52988400	-2.63405600	-4.54990500
H	-6.38175300	4.28112800	-0.43102900
H	-1.38388600	-0.30622200	-3.55476300
H	-6.87786800	3.14168300	0.83574400
H	-2.71588900	0.11002800	-2.49589100
H	-7.02176200	-0.19814500	1.36040000
H	-5.35023100	7.10598000	-3.29338100
H	-4.36422100	5.84622400	-4.05982600
H	-2.19961400	-4.51671600	-3.21500100
H	-2.36642200	-2.51625100	-4.05643600
H	-4.38983400	-1.81146200	-1.86957300
H	-5.43859100	-0.43141600	-1.21574000
H	-5.77480300	5.39426500	-3.08122800
H	-6.49483000	-4.27535600	0.06382100
H	-7.71668300	3.14034300	-0.74169400
H	-4.76640700	1.31467100	-3.19267200
H	-7.81450300	-3.19583700	0.58589200
H	-5.36765200	2.98539800	-3.26475800
H	-7.05924400	0.10781400	-0.98529200
H	-3.62606300	-1.30854700	-4.26976900
H	-6.93114000	-2.86541400	-0.92715400
H	-5.46789600	-4.00523700	-2.47558800
H	-6.50979400	1.63358400	-3.13024100
H	-4.55477500	-4.00301000	-3.97846500
H	-6.54576600	-1.80848900	-3.10052900
H	-5.67665100	-1.81566100	-4.64602300
H	-6.87431900	-3.07618000	-4.30194200
K	-0.05188900	2.67502100	-0.05406500
K	0.09028400	-2.70189200	0.06166900
N	3.89584500	2.31001100	0.17167600
N	4.14313500	-2.10899000	-0.49941500
N	0.63905300	-0.00899400	-0.13619400
N	-0.57450100	-0.03710200	0.01482000
N	-3.95555600	2.16772400	-0.12725000
N	-3.98254100	-2.31115600	0.40634200
Si	5.48912700	2.24956000	0.80974300
Si	5.72555900	-1.91932800	-1.12706400
Si	-5.56616900	-2.18138300	1.04823600
Si	-5.48515900	2.00302400	-0.88945000
Sr	3.03453200	0.06554500	-0.34366500
Sr	-2.97342700	-0.08845200	0.18129200

222

[K(^{DIPeP}NN)Ca]$_2$(N$_2$) (6-Ca) [TS]

Ca	-2.72731700	-0.12741400	-0.11327400
Ca	2.72733300	0.12765700	-0.11299800
K	0.23572800	-3.12967600	0.09417400
K	-0.23523700	3.12977300	0.09514800
Si	-5.16288000	-2.15649000	-1.46962500
Si	5.16283400	2.15616900	-1.47058300
Si	-5.56017300	1.23086900	1.37254100
Si	5.56047500	-1.23074600	1.37190400
N	-3.57760900	-2.17625400	-0.76968600
N	0.47234100	-0.41524900	-0.08462100
N	-4.01285200	1.60620300	0.70003300
N	-0.47254300	0.41562900	-0.08484800
N	3.57780400	2.17613700	-0.77011700
N	4.01289700	-1.60599900	0.69992400
C	-3.45870000	-5.62627700	-3.79083600
C	-2.25533200	-4.70407100	-3.93900400
C	-0.97623700	-1.92730800	-4.41624300
C	-5.26394900	-1.99246800	-3.36203900
C	-6.56192200	2.83152700	-3.23009100
C	-2.18710000	-3.59913600	-2.88187700
C	-0.98733100	-2.65734900	-3.08027000
C	0.97578400	1.92681200	-4.41577100
C	-3.41062300	2.33712600	-3.12009500
C	-5.50436500	3.62846400	-2.47760500
C	-6.11987800	-3.69553300	-0.95613700
C	6.56094100	-2.83302000	-3.23047400
C	3.40974900	-2.33782400	-3.11965300
C	-2.48707000	1.18116400	-2.77461100
C	-2.21133100	-4.10438200	-1.45759600
C	-4.33044400	2.77235500	-1.97106100
C	5.50335900	-3.62957700	-2.47762400
C	2.48664800	-1.18151600	-2.77412800
C	-1.51557300	-5.26562400	-1.11149100
C	-6.05195700	-0.60064900	-0.86164700
C	0.98713000	2.65696900	-3.07986300
C	2.25442700	4.70390000	-3.93912900
C	3.02771100	-4.72567700	-1.08625400
C	5.26332000	1.99228800	-3.36303600
C	4.32984200	-2.77306100	-1.97084200
C	-2.90019900	-3.33840100	-0.45838700
C	3.45774900	5.62625700	-3.79151500
C	2.42485500	-5.46563000	-0.06902400
C	2.18678300	3.59899900	-2.88193300
C	-3.02826800	4.72535700	-1.08737000
C	-3.59317600	3.46693500	-0.84674000
C	3.59274600	-3.46721200	-0.84614900
C	-1.44697300	-5.71319400	0.20902400
C	-6.13039100	-0.42890500	0.66217800
C	-2.82284800	-3.81566200	0.89220800
C	-2.42531500	5.46572400	-0.07049500
C	-6.81106400	2.57982200	0.96200800
C	-2.09823700	-4.97529300	1.19518700
C	2.39952200	-4.92904200	1.21916600
C	-3.53802600	2.88219200	0.46450500
C	2.21140200	4.10428800	-1.45767800
C	3.53786300	-2.88200200	0.46490100
C	6.05183000	0.60017000	-0.86289900
C	1.51576400	5.26554000	-1.11140900
C	-3.58624800	-3.12841900	2.00336800
C	-4.59150500	-4.08711000	2.66443100
C	6.12023900	3.69503000	-0.95730000
C	2.93574900	-3.66876700	1.50133900
C	2.90050900	3.33829200	-0.45863600
C	-2.39978400	4.92962100	1.21789200
C	-2.93587900	3.66940000	1.50059600
C	-5.70504400	-3.37980800	3.42571200
C	6.81098600	-2.58008000	0.96147000
C	6.13088300	0.42870900	0.66092300
C	1.44750000	5.71312400	0.20912400
C	-5.59893700	0.91566900	3.24727900
C	-2.66656300	-2.46790000	3.04022400
C	-1.98198000	-1.20597100	2.54445300
C	2.85057300	-3.11889300	2.90712800
C	2.82343800	3.81551800	0.89199600
C	1.45779500	-2.52369100	3.17108200
C	-2.85060000	3.12002300	2.90657800
C	4.63229500	-4.77054300	3.72583200
C	3.27239700	-4.13282300	3.97745000
C	2.09895200	4.97519000	1.19514200
C	-4.63211500	4.77197400	3.72502200
C	-1.45793200	2.52460200	3.17062400
C	1.98230100	1.20626900	2.54477800
C	3.58698100	3.12819400	2.00301700
C	-3.27214800	4.13438100	3.97659000
C	5.59971200	-0.91501900	3.24653400
C	1.30469000	-1.85026000	4.52828600
C	2.66739400	2.46806500	3.04020800
C	4.59267300	4.08673300	2.66363800
C	-1.30495400	1.85133700	4.52792400
C	5.70608000	3.37932700	3.42501300
H	-3.49546300	-6.37002900	-4.60149800
H	-2.28972500	-4.22968400	-4.93291400
H	-0.79114200	-2.60723000	-5.26079100
H	-4.39962500	-5.05514900	-3.81953400
H	-4.99979700	-2.91126500	-3.90546200
H	-6.15179700	2.33099700	-4.12122100
H	-1.94198100	-1.43001900	-4.59721900
H	-6.29651100	-1.72775000	-3.64500900
H	-7.38432300	3.47980100	-3.56938100
H	-1.32164700	-5.29580200	-3.93234900
H	-0.19376100	-1.15513600	-4.44136500
H	-4.60682200	-1.18611300	-3.72720000
H	-3.44002700	-6.16950100	-2.83449400
H	-4.02604300	2.04505600	-3.98621300
H	-6.99596800	2.04903900	-2.58761000
H	0.79021300	2.60660200	-5.26032100
H	-7.09653500	-3.75548500	-1.46182500
H	-3.07784600	-2.97116500	-2.99803000
H	0.19351800	1.15441900	-4.44054900
H	6.15069500	-2.33238400	-4.12149000
H	-5.11967500	4.44297500	-3.11710700
H	2.80853200	-3.20150500	-3.45330500
H	1.94160300	1.42977000	-4.59703100
H	4.02495300	-2.04612500	-3.98605000
H	-0.04837700	-3.23147400	-2.96358400
H	-1.81923900	0.92288600	-3.60665200
H	-2.80975400	3.20089300	-3.45414600
H	-5.54880400	-4.60531400	-1.20120700
H	7.38302200	-3.48157500	-3.57000300
H	5.11825400	-4.44402900	-3.11695300
H	1.81866500	-0.92318300	-3.60603100
H	-0.99995300	-5.83040400	-1.89253100
H	2.28846000	4.22947500	-4.93303200

H	3.07833000	-5.14607100	-2.09520900
H	-7.05848900	-0.61263400	-1.31337800
H	-3.06772200	0.26359300	-2.56808900
H	-0.99796200	-1.90820100	-2.26887300
H	-5.97053300	4.12301300	-1.61095500
H	-5.55832300	0.26472400	-1.33947200
H	4.60599200	1.18605100	-3.72809000
H	4.99912800	2.91117500	-3.90628900
H	-6.29573200	-3.69446800	0.13063300
H	-4.76223600	1.86544300	-1.52338900
H	-3.07907300	5.14539200	-2.09646800
H	6.99544400	-2.05064900	-2.58815900
H	6.29577100	1.72747900	-3.64631900
H	1.99706100	-6.44959900	-0.27223600
H	3.49406800	6.36999100	-4.60221400
H	1.32067000	5.29551100	-3.93210800
H	0.04810900	3.23093500	-2.96294500
H	3.06762600	-0.26409400	-2.56788400
H	-1.82418600	1.41691100	-1.92432200
H	1.82391000	-1.41689000	-1.92362800
H	5.96962300	-4.12419000	-1.61106000
H	4.39873000	5.05523900	-3.82061100
H	0.99811200	1.90791400	-2.26839600
H	3.07761000	2.97120300	-2.99838500
H	-7.03990200	2.57787500	-0.11464400
H	4.76200500	-1.86616200	-1.52350400
H	-0.89711600	-6.62178400	0.46237900
H	-7.15966600	-0.59011400	1.02580600
H	-5.54068100	-1.20671500	1.17752000
H	3.43943500	6.16950800	-2.83518100
H	-5.03300800	-4.72131400	1.87942500
H	5.55776600	-0.26516300	-1.34035400
H	-1.99762400	6.44965300	-0.27412900
H	0.99995300	5.83032800	-1.89231500
H	-7.75528200	2.43885300	1.51144300
H	7.05816100	0.61184700	-1.31508600
H	7.09666800	3.75495700	-1.46343100
H	-4.16859400	-2.33360700	1.51438400
H	-6.40994200	3.57363600	1.21652900
H	1.94430600	-5.50670600	2.02898800
H	7.03944100	-2.57862200	-0.11526300
H	5.54914600	4.60490600	-1.20197700
H	-2.06085300	-5.32016400	2.23262400
H	-6.30223300	-2.74595400	2.75208300
H	-4.05393900	-4.77660200	3.33967900
H	6.40979600	-3.57372200	1.21654700
H	-6.38814300	-4.10325500	3.89643200
H	-1.37743900	-1.37351700	1.63757800
H	4.64016600	-5.35080800	2.79142400
H	6.29659700	3.69378600	0.12938800
H	-2.72047400	-0.40366700	2.36319100
H	-6.56076400	0.44990500	3.51996200
H	-1.94454000	5.50764000	2.02744900
H	-4.64021500	5.35184000	2.79036700
H	0.69403700	-3.31846000	3.06957700
H	1.24813700	-1.78207400	2.37969000
H	7.75542300	-2.43903600	1.51050800
H	0.89774000	6.62173700	0.46261000
H	7.16037500	0.58963600	1.02406800
H	-1.24847000	1.78276400	2.37938400
H	-3.55126900	2.27678400	2.94479700
H	-4.80129700	0.21265600	3.53901000
H	2.50228000	-4.92006500	4.07499500
H	-1.90815500	-3.19804300	3.37248300
H	1.37772300	1.37381700	1.63793300
H	-5.31640800	-2.72938100	4.22483300
H	5.54167500	1.20684600	1.17634900
H	-5.41909100	4.00733100	3.63882500
H	3.55110800	-2.27552000	2.94497000
H	-5.48616800	1.82295100	3.85829000
H	-3.25258000	-2.21492000	3.93844300
H	4.16900400	2.33316600	1.51398700
H	2.72046700	0.40365000	2.36356200
H	4.91355000	-5.44711900	4.54733300
H	5.41927000	-4.00595700	3.63912900
H	-1.28073900	-0.79596100	3.28221300
H	-0.69401000	3.31920600	3.06900300
H	-4.91319500	5.44889300	4.54630100
H	5.03427900	4.72051100	1.87834600
H	-2.50201300	4.92167000	4.07362700
H	1.28100600	0.79665300	3.28270200
H	2.06180500	5.32006200	2.23258800
H	3.29155000	-3.62112800	4.95329700
H	0.34244000	-1.32290000	4.60824000
H	5.48661300	-1.82207700	3.85781500
H	4.80244600	-0.21155200	3.53819100
H	-2.10671700	1.11474500	4.69413500
H	6.56179400	-0.44962000	3.51893900
H	-3.29106800	3.62308800	4.95265100
H	1.34568300	-2.57450700	5.35481700
H	2.10634800	-1.11354200	4.69444600
H	6.30286700	2.74490700	2.75156200
H	1.90928800	3.19846200	3.37259900
H	-0.34278100	1.32385300	4.60797100
H	4.05543300	4.77664700	3.33871300
H	3.25357500	2.21500700	3.93829500
H	-1.34587800	2.57569300	5.35436100
H	6.38957500	4.10272500	3.89523300
H	5.31736100	2.72945000	4.22453900

222

[K(^{DIPeP}NN)Sr]$_2$(N$_2$) (6-Sr) [TS]

C	2.15988000	7.04766000	2.43207200
C	4.57423000	3.26078900	2.94931100
C	6.31150800	-0.62224400	3.81255600
C	5.16150500	4.96012400	0.46235300
C	1.20241300	5.93486300	2.83629900
C	0.77737500	3.09625900	4.08376700
C	5.61830800	-1.78799800	3.12058700
C	5.69289200	1.87978400	0.61185400
C	1.45001500	4.61781000	2.10118200
C	0.56691600	3.46959000	2.62349800
C	7.52691700	-1.06073600	-0.16102800
C	5.85640200	1.44954000	-0.85437200
C	3.19058700	-1.05117300	3.38461300
C	1.30292900	4.69294700	0.59712300
C	4.31159000	-1.40946900	2.39781900
C	0.35752500	5.54402400	0.02131200
C	2.06240000	3.78384100	-0.21891700
C	4.17798400	4.54112500	-3.29997100
C	2.06967800	-0.20922300	2.79299900
C	3.95014300	-2.49893700	1.40577000
C	0.08802300	5.52966000	-1.34896100
C	1.74892000	3.76481700	-1.62268200
C	3.50565300	-3.73228800	1.89604600

C	6.35876500	-0.62947000	-2.96349300
C	-0.19492800	-3.12396600	4.29559700
C	0.78343600	4.62738200	-2.15209100
C	3.14423300	3.51276700	-3.73125000
C	4.19139200	-2.31848200	-0.00406700
C	2.46444200	2.80790800	-2.55195000
C	-2.35880100	-0.29925900	3.78761400
C	-2.43039700	1.08047900	3.14920500
C	-3.23641100	5.00606300	1.31483800
C	-3.29751700	3.86590500	2.11412400
C	3.28472400	-4.82371600	1.05589800
C	-0.28108800	-3.43687700	2.80808100
C	6.36360300	-4.57747600	-2.21468700
C	3.94567100	-3.45109900	-0.85193000
C	-4.84514400	1.71146700	3.65963600
C	-3.79989700	1.46265200	2.56325000
C	3.51319700	-4.66593600	-0.31225500
C	-3.62621900	4.89366700	-0.01994200
C	-3.70349200	2.62947200	1.60165500
C	1.53468600	1.67319900	-3.01699100
C	4.13583400	-3.32735000	-2.34721700
C	2.23379800	0.47667400	-3.64926700
C	-6.24708800	1.91962500	3.10887100
C	-1.34278700	-5.72407300	3.23726800
C	5.02524600	-4.43397900	-2.92677300
C	-1.38897000	-4.43075000	2.41542300
C	-4.04364700	3.67979100	-0.57730200
C	-4.06834200	2.47867100	0.21400900
C	2.77501500	-3.22200100	-3.05573500
C	-0.43916400	-5.59001500	0.40020700
C	-1.34675300	-4.66331300	0.92190900
C	-3.24428700	3.75986800	-2.96692900
C	-2.38958600	-6.75379500	2.83272000
C	-4.81005300	-3.53154200	3.02213800
C	-2.50779200	2.43435900	-3.11880200
C	-4.46348400	3.67327400	-2.03184100
C	-5.50346900	4.76332200	-2.33740800
C	-0.29335500	-5.78363200	-0.97359300
C	2.84153900	-3.00229700	-4.56076600
C	-2.18695600	-3.88952300	0.05348000
C	-1.08696000	-5.02740600	-1.83523900
C	-1.77644400	-1.13957100	-2.68006300
C	-5.66918700	-1.66653500	0.83726100
C	-2.02459600	-4.10376200	-1.35865300
C	-6.31099400	4.49321800	-3.59982700
C	-7.30744900	1.48443200	-0.65190200
C	-5.78270500	-1.25418300	-0.63863500
C	-2.17389700	-2.46555300	-3.30810700
C	-2.92433500	-3.41235200	-2.35870700
C	-5.46929300	-4.70356500	0.27457800
C	-5.63414800	0.44714300	-3.00808600
C	-3.77443100	-4.43630800	-3.13182100
C	-5.01851700	-3.83605400	-3.77332600
H	1.96217900	7.97400300	2.99298900
H	3.20345500	6.75513800	2.62917000
H	5.62647200	3.16639200	3.26542800
H	6.07798200	5.24330200	1.00384100
H	7.24739200	-0.94606800	4.29288500
H	4.16516100	4.15709900	3.43873900
H	1.30561500	5.75176600	3.91788100
H	5.68481100	-0.16451100	4.59349400
H	6.56461700	0.17053000	3.09129800
H	2.08344800	7.27979900	1.35911900
H	4.40945700	5.74822800	0.62483500
H	4.03557200	2.38460800	3.34584600
H	0.47074900	3.90104000	4.76852000
H	6.67294000	2.14112100	1.04614100
H	0.15498700	6.25778000	2.69728400
H	1.83611400	2.87308300	4.28529600
H	5.38742200	4.93611000	-0.61356300
H	2.48583800	4.32324700	2.30556400
H	5.40539800	-2.59348300	3.84594700
H	0.19246200	2.20237700	4.34694600
H	3.62610000	-0.50321300	4.23511700
H	7.49015500	-0.73198700	0.88858800
H	6.30128600	-2.22729200	2.37689000
H	5.36981200	1.01985000	1.23007700
H	6.72884200	1.94669400	-1.31058600
H	8.46831400	-0.69545300	-0.60179900
H	4.55349700	-0.51429700	1.80241300
H	-0.49409300	3.73445200	2.46217500
H	-0.20757800	6.22298600	0.66321800
H	3.72765200	5.31198700	-2.65671900
H	2.43032300	0.80257700	2.52996400
H	0.77338300	2.57751000	2.00641300
H	7.55230100	-2.16213500	-0.16589400
H	4.98758800	4.06365800	-2.72666900
H	2.77217700	-1.97683900	3.81259000
H	5.00764800	1.80248200	-1.47244600
H	4.63242900	5.04300600	-4.16744000
H	1.25242400	-0.04358100	3.50861700
H	0.48744500	-2.28207800	4.48438300
H	7.20461100	0.01572400	-3.25439300
H	3.34854100	-3.84868100	2.97240800
H	-2.14081200	1.84387200	3.89043900
H	1.60204800	-0.67222300	1.90043200
H	-2.90995300	5.96361300	1.72597000
H	-1.35937000	-0.48378800	4.20405500
H	3.28314000	2.37824600	-1.94873500
H	-3.03248200	3.93853600	3.17310700
H	-0.65265200	6.20715000	-1.77810400
H	0.17936600	-3.98304500	4.87165700
H	-1.18093600	-2.85578100	4.70529400
H	-3.08850500	-0.42343900	4.60111600
H	6.61312000	-1.65852500	-3.25782600
H	2.37485400	3.98799800	-4.36521800
H	-4.53600000	2.58724000	4.25662800
H	5.48397000	-0.31741100	-3.55712000
H	-1.64896600	1.14121000	2.36509100
H	6.92639000	-3.63156300	-2.23464400
H	3.62795900	2.75370400	-4.36783500
H	0.57889300	4.59985200	-3.22625600
H	6.22935700	-4.85322900	-1.15811900
H	0.69605900	-3.82348400	2.46629800
H	2.95018300	-5.78142900	1.46015700
H	-0.45822200	-2.49747500	2.25237800
H	-4.84626100	0.85539900	4.35498900
H	-2.55230500	-1.11614000	3.06827400
H	6.98959300	-5.34812900	-2.68993600
H	-3.60087100	5.78238100	-0.65667300
H	4.64216800	-2.36827900	-2.51081500
H	2.98539300	0.02196000	-2.97831100
H	0.92311300	1.30829900	-2.17067000
H	-1.48660600	-5.46523100	4.29855900
H	-0.33402200	-6.17289000	3.18128000
H	-6.96960500	2.12445000	3.91311700

H	0.79936600	2.08760100	-3.72802000
H	2.76520300	0.74592600	-4.57373900
H	3.34805800	-5.51472500	-0.98254100
H	-4.20552800	0.61943300	1.97763200
H	-6.27322400	2.76299400	2.40246200
H	-2.34281500	-3.92999400	2.62614700
H	-2.56278600	4.53881000	-2.58256500
H	0.18286000	-6.16747100	1.08863800
H	5.20418300	-4.21860800	-3.99283600
H	-4.29957900	-2.73703500	3.58948700
H	4.48587800	-5.39872800	-2.90693000
H	2.23135700	-2.36748400	-2.61325900
H	-6.59162400	1.02505900	2.56736600
H	1.50921100	-0.31257100	-3.89299300
H	-2.43549400	1.89200700	-2.16300400
H	-5.00285400	5.74436300	-2.41864000
H	-2.35787500	-7.63522800	3.49136400
H	-1.49612600	2.56331800	-3.53168200
H	-4.39543100	-4.49567600	3.35079900
H	3.47780900	-2.13702500	-4.80472100
H	2.17561600	-4.12767600	-2.84367000
H	-6.18828800	4.84782800	-1.47771300
H	0.42046400	-6.51268600	-1.36304700
H	-5.87453700	-3.51413700	3.31019100
H	-3.40439200	-6.33101000	2.89053500
H	-1.05041200	-1.23849500	-1.85277200
H	-5.25243100	-0.84457300	1.45232700
H	-3.55936200	4.10789400	-3.96468300
H	-2.24243000	-7.09835300	1.79859900
H	-4.94442800	2.70753100	-2.22341300
H	3.25208000	-3.87608700	-5.08781000
H	-3.05576400	1.75449700	-3.78594500
H	1.84001800	-2.80942300	-4.97538500
H	-7.22551300	2.56076500	-0.87097000
H	-1.28735300	-0.47118800	-3.40411900
H	-7.51899200	1.38211800	0.42261200
H	-2.68018600	-0.59805300	-2.34646700
H	-6.66929100	-1.81997100	1.27662200
H	-7.01188200	5.31504400	-3.81251000
H	-5.66605800	4.37511000	-4.48508700
H	-0.99230800	-5.17356700	-2.91526800
H	-1.27765000	-2.97559300	-3.70347400
H	-3.62137800	-2.80822500	-1.75970700
H	-4.97905300	-1.73121100	-1.23231300
H	-6.90289000	3.56983800	-3.49852500
H	-4.82466200	-5.59522300	0.33620100
H	-8.16736000	1.08691500	-1.21413800
H	-4.68412300	-0.01588700	-3.32010200
H	-6.44388500	-4.95477000	0.72241200
H	-5.74095900	1.39469800	-3.55477900
H	-6.70077200	-1.67282300	-1.08470000
H	-2.81178100	-2.25974600	-4.18298800
H	-5.62890100	-4.47934800	-0.79061200
H	-4.07346300	-5.23622100	-2.43630800
H	-6.44504000	-0.22044500	-3.34436500
H	-3.15136000	-4.92527200	-3.90192300
H	-5.68300800	-3.39986600	-3.01099500
H	-4.77372600	-3.03369500	-4.48667000
H	-5.59355900	-4.59794700	-4.32153100
K	-0.95958800	2.80393600	-0.23274200
K	0.89684900	-3.02146600	-0.37935400
N	2.99115600	2.92213700	0.30215700
N	4.56059200	-1.09725300	-0.50569900
N	0.54364500	-0.44567100	-0.29448600
N	-0.43844900	0.20714200	0.06037500
N	-4.34750400	1.23611700	-0.29127000
N	-3.06907100	-2.95115600	0.54073600
Si	4.50182400	3.29749600	1.04662000
Si	6.02302100	-0.42929600	-1.10608600
Si	-4.65565400	-3.24897000	1.15214500
Si	-5.71534000	0.58878600	-1.11106700
Sr	2.76229900	0.50252400	-0.14915500
Sr	-2.71123900	-0.52969400	0.30196900

Triplet states for 6-Ae

222

$[K(^{DIPeP}NN)Ca]_2(N_2)$ (6-Ca) TRIPLET

Ca	-0.028750	2.362740	0.029464
K	3.184294	0.037263	-0.012227
Si	-1.670075	5.192912	-1.425362
N	-1.814014	3.652552	-0.639132
N	0.630565	0.005676	0.033613
C	-3.014295	3.115104	-0.221034
C	-3.995825	2.613702	-1.145491
C	-5.187940	2.051403	-0.683002
H	-5.918518	1.683846	-1.407992
C	-5.455954	1.913572	0.679924
H	-6.386522	1.456781	1.023131
C	-4.511615	2.380728	1.590874
H	-4.720789	2.301403	2.661579
C	-3.321636	2.993118	1.177307
C	-3.690464	2.603363	-2.627578
H	-2.965686	3.405130	-2.805271
C	-4.900673	2.867745	-3.526462
H	-4.559792	2.856553	-4.573951
H	-5.620378	2.032577	-3.449049
C	-5.601618	4.191142	-3.254538
H	-6.447462	4.343729	-3.942299
H	-4.909024	5.036831	-3.384725
H	-5.990193	4.242234	-2.226369
C	-2.966141	1.294871	-2.993342
H	-2.120296	1.167437	-2.295067
H	-3.650887	0.441426	-2.825185
C	-2.431047	1.237377	-4.417345
H	-3.238908	1.226441	-5.163546
H	-1.829868	0.330651	-4.577861
H	-1.791999	2.107892	-4.631889
C	-2.462463	3.664135	2.233272
H	-1.593172	4.089105	1.706899
C	-3.250765	4.850910	2.822120
H	-3.679349	5.415101	1.978421
H	-4.115904	4.458274	3.384814
C	-2.450941	5.798379	3.705699
H	-3.081971	6.631408	4.051435
H	-1.599252	6.231578	3.161619
H	-2.050005	5.301855	4.602387
C	-1.937865	2.715569	3.318443
H	-2.761999	2.089653	3.700891
H	-1.592578	3.311084	4.177792
C	-0.792388	1.832124	2.854440
H	-1.079917	1.161542	2.023926
H	-0.428863	1.170366	3.651598
H	0.081516	2.444095	2.562605
C	-0.156911	6.143230	-0.804117
H	0.742875	5.769389	-1.317274
H	-0.297571	7.165386	-1.199471
C	-1.410970	5.164276	-3.311390
H	-0.673168	4.399240	-3.600473
H	-1.008957	6.140036	-3.630164
H	-2.328105	4.984088	-3.891502
C	-3.191595	6.235452	-1.030793
H	-4.120060	5.661609	-1.166809
H	-3.243011	7.133675	-1.666236
H	-3.155727	6.564639	0.019804
Si	1.580188	5.261945	1.344195
N	1.749772	3.693068	0.620077
C	2.969518	3.170892	0.234757
C	3.928004	2.678822	1.186060
C	5.142937	2.136538	0.758905
H	5.855104	1.776528	1.506036
C	5.457078	2.012869	-0.595029
H	6.405585	1.573668	-0.911150
C	4.533904	2.469861	-1.532998
H	4.776195	2.397706	-2.597247
C	3.321636	3.060194	-1.153559
C	3.587005	2.661475	2.659809
H	2.793882	3.403057	2.805321
C	4.754927	3.038328	3.576484
H	4.386247	3.055513	4.614553
H	5.522981	2.243645	3.555618
C	5.390125	4.384528	3.258826
H	6.198826	4.620551	3.967373
H	4.648338	5.195442	3.317189
H	5.814916	4.403852	2.244117
C	2.965655	1.303151	3.032128
H	2.122985	1.113273	2.343774
H	3.706526	0.500474	2.853818
C	2.455156	1.208763	4.463410
H	3.273921	1.237291	5.197137
H	1.905352	0.270617	4.627225
H	1.774030	2.042867	4.693950
C	2.471487	3.701731	-2.233216
H	1.603435	4.143008	-1.719698
C	3.259889	4.861201	-2.872704
H	3.729595	5.436518	-2.059112
H	4.095823	4.443843	-3.461408
C	2.439039	5.802150	-3.744160
H	3.069070	6.610687	-4.145401
H	1.624736	6.269595	-3.171310
H	1.982268	5.289229	-4.604268
C	1.944929	2.715128	-3.283477
H	2.771756	2.082121	-3.649067
H	1.592948	3.280897	-4.160342
C	0.806540	1.839643	-2.788623
H	1.101155	1.190155	-1.944285
H	0.440460	1.157172	-3.566898
H	-0.067135	2.455316	-2.505250
C	0.034935	6.155114	0.715911
H	-0.855474	5.763468	1.231857
H	0.147088	7.185816	1.098020
C	1.364255	5.293106	3.234785
H	0.638047	4.532938	3.564107
H	0.966414	6.276186	3.535910
H	2.296578	5.132533	3.795639
C	3.065383	6.326818	0.876516
H	4.011604	5.782636	1.012669
H	3.106888	7.249616	1.476502
H	2.997005	6.614952	-0.184438
N	-0.630565	-0.005676	0.033613
K	-3.184294	-0.037263	-0.012227
Ca	0.028750	-2.362740	0.029464
Si	1.670075	-5.192912	-1.425362
N	1.814014	-3.652552	-0.639132
C	3.014295	-3.115104	-0.221034
C	3.995825	-2.613702	-1.145491
C	5.187940	-2.051403	-0.683002
H	5.918518	-1.683846	-1.407992
C	5.455954	-1.913572	0.679924
H	6.386522	-1.456781	1.023131

C	4.511615	-2.380728	1.590874
H	4.720789	-2.301403	2.661579
C	3.321636	-2.993118	1.177307
C	3.690464	-2.603363	-2.627578
H	2.965686	-3.405130	-2.805271
C	4.900673	-2.867745	-3.526462
H	4.559792	-2.856553	-4.573951
H	5.620378	-2.032577	-3.449049
C	5.601618	-4.191142	-3.254538
H	6.447462	-4.343729	-3.942299
H	4.909024	-5.036831	-3.384725
H	5.990193	-4.242234	-2.226369
C	2.966141	-1.294871	-2.993342
H	2.120296	-1.167437	-2.295067
H	3.650887	-0.441426	-2.825185
C	2.431047	-1.237377	-4.417345
H	3.238908	-1.226441	-5.163546
H	1.829868	-0.330651	-4.577861
H	1.791999	-2.107892	-4.631889
C	2.462463	-3.664135	2.233272
H	1.593172	-4.089105	1.706899
C	3.250765	-4.850910	2.822120
H	3.679349	-5.415101	1.978421
H	4.115904	-4.458274	3.384814
C	2.450941	-5.798379	3.705699
H	3.081971	-6.631408	4.051435
H	1.599252	-6.231578	3.161619
H	2.050005	-5.301855	4.602387
C	1.937865	-2.715569	3.318443
H	2.761999	-2.089653	3.700891
H	1.592578	-3.311084	4.177792
C	0.792388	-1.832124	2.854440
H	1.079917	-1.161542	2.023926
H	0.428863	-1.170366	3.651598
H	-0.081516	-2.444095	2.562605
C	0.156911	-6.143230	-0.804117
H	-0.742875	-5.769389	-1.317274
H	0.297571	-7.165386	-1.199471
C	1.410970	-5.164276	-3.311390
H	0.673168	-4.399240	-3.600473
H	1.008957	-6.140036	-3.630164
H	2.328105	-4.984088	-3.891502
C	3.191595	-6.235452	-1.030793
H	4.120060	-5.661609	-1.166809
H	3.243011	-7.133675	-1.666236
H	3.155727	-6.564639	0.019804
Si	-1.580188	-5.261945	1.344195
N	-1.749772	-3.693068	0.620077
C	-2.969518	-3.170892	0.234757
C	-3.928004	-2.678822	1.186060
C	-5.142937	-2.136538	0.758905
H	-5.855104	-1.776528	1.506036
C	-5.457078	-2.012869	-0.595029
H	-6.405585	-1.573668	-0.911150
C	-4.533904	-2.469861	-1.532998
H	-4.776195	-2.397706	-2.597247
C	-3.321636	-3.060194	-1.153559
C	-3.587005	-2.661475	2.659809
H	-2.793882	-3.403057	2.805321
C	-4.754927	-3.038328	3.576484
H	-4.386247	-3.055513	4.614553
H	-5.522981	-2.243645	3.555618
C	-5.390125	-4.384528	3.258826
H	-6.198826	-4.620551	3.967373
H	-4.648338	-5.195442	3.317189
H	-5.814916	-4.403852	2.244117
C	-2.965655	-1.303151	3.032128
H	-2.122985	-1.113273	2.343774
H	-3.706526	-0.500474	2.853818
C	-2.455156	-1.208763	4.463410
H	-3.273921	-1.237291	5.197137
H	-1.905352	-0.270617	4.627225
H	-1.774030	-2.042867	4.693950
C	-2.471487	-3.701731	-2.233216
H	-1.603435	-4.143008	-1.719698
C	-3.259889	-4.861201	-2.872704
H	-3.729595	-5.436518	-2.059112
H	-4.095823	-4.443843	-3.461408
C	-2.439039	-5.802150	-3.744160
H	-3.069070	-6.610687	-4.145401
H	-1.624736	-6.269595	-3.171310
H	-1.982268	-5.289229	-4.604268
C	-1.944929	-2.715128	-3.283477
H	-2.771756	-2.082121	-3.649067
H	-1.592948	-3.280897	-4.160342
C	-0.806540	-1.839643	-2.788623
H	-1.101155	-1.190155	-1.944285
H	-0.440460	-1.157172	-3.566898
H	0.067135	-2.455316	-2.505250
C	-0.034935	-6.155114	0.715911
H	0.855474	-5.763468	1.231857
H	-0.147088	-7.185816	1.098020
C	-1.364255	-5.293106	3.234785
H	-0.638047	-4.532938	3.564107
H	-0.966414	-6.276186	3.535910
H	-2.296578	-5.132533	3.795639
C	-3.065383	-6.326818	0.876516
H	-4.011604	-5.782636	1.012669
H	-3.106888	-7.249616	1.476502
H	-2.997005	-6.614952	-0.184438

222

[K(^{DIPeP}NN)Sr]$_2$(N$_2$) (6-Sr) TRIPLET

C	4.092923	6.297964	2.728824
C	5.276635	1.846999	3.007585
C	6.026123	-2.137122	3.568808
C	6.273386	3.555019	0.654069
C	2.860130	5.480162	3.089064
C	1.680849	2.804414	4.213471
C	5.037443	-3.066947	2.878828
C	6.023534	0.452953	0.528239
C	2.725828	4.184125	2.289760
C	1.561168	3.304070	2.780771
C	6.680087	-2.869657	-0.582848
C	5.971991	0.108646	-0.967606
C	2.873423	-1.798577	3.297652
C	2.592496	4.368733	0.793472
C	3.831150	-2.355579	2.235483
C	1.942791	5.489836	0.273007
C	3.032756	3.309418	-0.074684
C	5.268904	3.546788	-3.143157
C	1.950564	-0.696638	2.803129
C	3.180786	-3.285419	1.230204
C	1.669889	5.619649	-1.090861
C	2.722802	3.453658	-1.471732
C	2.562830	-4.447043	1.707550

C	5.589743	-1.798952	-3.238362
C	-1.059260	-3.348262	4.507017
C	2.057599	4.589036	-1.945188
C	3.979211	2.888795	-3.608039
C	3.320357	-3.060690	-0.184914
C	3.110366	2.370751	-2.455913
C	-1.949123	-0.078767	3.527579
C	-1.774428	1.292203	2.889468
C	-1.938506	5.431364	1.348422
C	-2.184686	4.285184	2.102639
C	2.085545	-5.435560	0.848541
C	-1.151777	-3.606956	3.009560
C	4.634494	-5.660631	-2.848295
C	2.802667	-4.082572	-1.051513
C	-3.962581	2.350814	3.662635
C	-3.080361	1.982782	2.462056
C	2.222019	-5.239838	-0.525937
C	-2.366038	5.444925	0.021029
C	-2.819281	3.167131	1.557039
C	1.875455	1.608122	-2.967931
C	2.814609	-3.881275	-2.550425
C	2.164967	0.270956	-3.637498
C	-5.296199	2.964551	3.263373
C	-3.001362	-5.348925	3.268530
C	3.296869	-5.112164	-3.326645
C	-2.521122	-4.103513	2.512116
C	-3.006300	4.350745	-0.569939
C	-3.245566	3.146422	0.179666
C	1.431345	-3.391839	-3.015529
C	-1.818710	-5.416954	0.499154
C	-2.480615	-4.297823	1.012470
C	-2.208806	4.281936	-2.962492
C	-4.309091	-5.929060	2.747738
C	-5.381767	-2.151979	3.188827
C	-1.751310	2.837396	-3.104554
C	-3.412796	4.465074	-2.022474
C	-4.171338	5.768964	-2.318518
C	-1.658310	-5.614115	-0.871894
C	1.312847	-3.106919	-4.506290
C	-3.056572	-3.316849	0.135351
C	-2.199188	-4.668607	-1.741799
C	-1.824511	-0.937841	-2.924028
C	-5.888492	-0.300748	0.906365
C	-2.895864	-3.547474	-1.277498
C	-5.032544	5.695396	-3.572243
C	-6.556282	3.094740	-0.621241
C	-5.931699	0.051152	-0.586909
C	-2.691550	-2.098066	-3.383983
C	-3.605315	-2.675828	-2.294765
C	-6.434533	-3.312348	0.545584
C	-5.309675	1.578013	-3.011631
C	-4.786605	-3.458680	-2.899648
C	-5.828687	-2.590785	-3.591742
H	4.156252	7.217672	3.330462
H	5.011945	5.718251	2.908846
H	6.269838	1.460848	3.290819
H	7.230738	3.554038	1.198869
H	6.859894	-2.704557	4.009699
H	5.108484	2.770298	3.581458
H	2.905354	5.222772	4.159344
H	5.561052	-1.556462	4.380412
H	6.454332	-1.416075	2.856087
H	4.092828	6.588429	1.667434
H	5.746602	4.493596	0.885793
H	4.533845	1.103187	3.338672
H	1.652511	3.625512	4.945140
H	7.065540	0.468669	0.891765
H	1.946518	6.091721	2.978964
H	2.624263	2.258054	4.362426
H	6.489713	3.556199	-0.424345
H	3.640506	3.603629	2.459565
H	4.665533	-3.823166	3.593102
H	0.856472	2.119047	4.461912
H	3.465826	-1.402980	4.137814
H	6.863653	-2.612639	0.471487
H	5.559311	-3.632489	2.091622
H	5.550413	-0.348095	1.123314
H	6.951386	0.275729	-1.446864
H	7.645737	-2.846301	-1.112486
H	4.236307	-1.511069	1.656334
H	0.617217	3.866948	2.663886
H	1.616781	6.279433	0.953729
H	5.062747	4.393838	-2.471854
H	2.529124	0.193325	2.493800
H	1.497375	2.429251	2.110734
H	6.296180	-3.901607	-0.614974
H	5.896262	2.831140	-2.590253
H	2.272612	-2.621987	3.719438
H	5.291535	0.789140	-1.510392
H	5.857658	3.920948	-3.994200
H	1.277640	-0.339553	3.595244
H	-0.109749	-2.853873	4.763867
H	6.580299	-1.423997	-3.545985
H	2.479486	-4.595197	2.788121
H	-1.239129	1.958565	3.586005
H	1.293844	-1.021142	1.974871
H	-1.437603	6.296873	1.786418
H	-0.975039	-0.525383	3.765872
H	3.753475	1.678674	-1.885660
H	-1.886265	4.261756	3.154724
H	1.161281	6.505771	-1.476096
H	-1.110032	-4.280845	5.087988
H	-1.878939	-2.698392	4.850196
H	-2.528991	-0.030894	4.460725
H	5.492475	-2.823894	-3.624953
H	3.389364	3.596472	-4.217312
H	-3.407370	3.042479	4.320855
H	4.831956	-1.177797	-3.741424
H	-1.089168	1.208432	2.021980
H	5.419184	-4.890371	-2.895834
H	4.219092	2.045868	-4.277150
H	1.846720	4.672512	-3.015356
H	4.580571	-6.003933	-1.804577
H	-0.370432	-4.332508	2.717889
H	1.622123	-6.342929	1.241242
H	-0.921319	-2.665897	2.479101
H	-4.142777	1.443396	4.262292
H	-2.476424	-0.790398	2.867133
H	4.962358	-6.509481	-3.467694
H	-2.186673	6.338025	-0.583573
H	3.514857	-3.060592	-2.748076
H	2.696828	-0.436179	-2.975008
H	1.154830	1.448168	-2.140569
H	-3.121634	-5.085496	4.331970
H	-2.215012	-6.125678	3.246609
H	-5.898857	3.222014	4.147333

H	1.330555	2.259127	-3.671864
H	2.794249	0.382626	-4.532379
H	1.845691	-6.002652	-1.213014
H	-3.689202	1.282720	1.864670
H	-5.150911	3.879831	2.670216
H	-3.235127	-3.292961	2.702699
H	-1.378610	4.919405	-2.606118
H	-1.398923	-6.148134	1.195638
H	3.379202	-4.841161	-4.391692
H	-4.659037	-1.474370	3.670400
H	2.530185	-5.907613	-3.286035
H	1.197096	-2.464959	-2.460671
H	-5.882833	2.263524	2.650370
H	1.232445	-0.220742	-3.945893
H	-1.575622	2.348037	-2.134284
H	-3.456000	6.605657	-2.407912
H	-4.629322	-6.792923	3.350161
H	-0.827805	2.759445	-3.695831
H	-5.218624	-3.160046	3.596450
H	2.119013	-2.439936	-4.849001
H	0.663787	-4.133462	-2.726483
H	-4.808784	6.012464	-1.452802
H	-1.131171	-6.490608	-1.254160
H	-6.390648	-1.830870	3.497871
H	-5.116793	-5.182652	2.780135
H	-1.165001	-1.198873	-2.076328
H	-5.261865	0.419360	1.462639
H	-2.464894	4.666254	-3.964018
H	-4.215191	-6.260851	1.703092
H	-4.100560	3.637988	-2.230220
H	1.368166	-4.027837	-5.105249
H	-2.522454	2.235662	-3.605108
H	0.352938	-2.622056	-4.740768
H	-6.192551	4.102364	-0.873519
H	-1.160581	-0.572968	-3.721153
H	-6.724357	3.068565	0.466175
H	-2.448257	-0.067469	-2.650844
H	-6.885455	-0.194001	1.366253
H	-5.526604	6.657389	-3.777889
H	-4.441726	5.433343	-4.464290
H	-2.102184	-4.821376	-2.820555
H	-2.052616	-2.899171	-3.791620
H	-4.047107	-1.839726	-1.729836
H	-5.332089	-0.673782	-1.167773
H	-5.820247	4.933183	-3.463863
H	-5.988735	-4.319263	0.553498
H	-7.524918	2.944691	-1.123900
H	-4.559509	0.829533	-3.316639
H	-7.388020	-3.354691	1.095698
H	-5.111464	2.499364	-3.577384
H	-6.950356	-0.074453	-0.992140
H	-3.312898	-1.756729	-4.227035
H	-6.656614	-3.052126	-0.500687
H	-5.268103	-4.028226	-2.090125
H	-6.292490	1.196308	-3.334795
H	-4.390925	-4.213624	-3.602411
H	-6.271325	-1.869214	-2.888194
H	-5.406203	-2.013819	-4.429016
H	-6.647043	-3.204216	-3.998605
K	-0.137489	3.278693	-0.094838
K	0.124253	-3.177105	0.008940
N	3.651847	2.178589	0.393497
N	3.868889	-1.896016	-0.669080
N	-0.029016	0.660761	-0.111673
N	0.015440	-0.601788	-0.058228
N	-3.786278	2.008432	-0.355815
N	-3.679351	-2.182646	0.602257
Si	5.216907	2.066541	1.117054
Si	5.441205	-1.671824	-1.347785
Si	-5.255282	-2.046418	1.295788
Si	-5.315388	1.766956	-1.116959
Sr	2.487486	0.087590	-0.205258
Sr	-2.496241	-0.082603	0.059239

Energy profile

109

(^{DIPeP}NN)Ca (5-Ca)

Ca	-0.000082	-0.000049	-0.765482
Si	1.916868	-0.992694	2.015773
Si	-1.916880	0.992979	2.015750
N	1.821568	-0.293186	0.438765
N	-1.821683	0.293274	0.438862
C	0.260256	-0.725444	2.888780
H	-0.470111	-1.416389	2.431280
H	0.389046	-1.101687	3.918559
C	-0.260123	0.726026	2.888586
H	0.470173	1.416853	2.430802
H	-0.388801	1.102587	3.918268
C	3.306632	-0.244284	3.048686
H	4.241407	-0.197571	2.467245
H	3.494535	-0.854680	3.946395
H	3.063252	0.775532	3.380215
C	2.211268	-2.863555	1.977148
H	1.448059	-3.383651	1.376864
H	2.177102	-3.285208	2.995330
H	3.196352	-3.104161	1.549014
C	-2.211511	2.863801	1.977057
H	-1.448208	3.384006	1.376981
H	-2.177652	3.285415	2.995265
H	-3.196515	3.104314	1.548688
C	-3.306330	0.244467	3.049013
H	-4.241412	0.198185	2.468031
H	-3.493649	0.854519	3.947079
H	-3.062989	-0.775543	3.379989
C	2.889160	0.182101	-0.318126
C	3.365933	1.523412	-0.166324
C	4.390618	2.000592	-0.985840
H	4.738668	3.029473	-0.856729
C	4.974327	1.202481	-1.967080
H	5.779781	1.591648	-2.594227
C	4.506466	-0.096085	-2.139264
H	4.950462	-0.725620	-2.916191
C	3.481045	-0.616647	-1.343807
C	2.726179	2.459953	0.835611
H	2.053836	1.841587	1.447064
C	1.857714	3.511349	0.126786
H	1.397736	4.175506	0.876005
H	2.504254	4.150370	-0.499573
C	0.758313	2.915379	-0.736842
H	1.205590	2.236804	-1.486576
H	0.195464	3.680957	-1.290308
H	0.019195	2.382743	-0.112642
C	3.748202	3.112619	1.776270
H	4.455356	2.336082	2.106403
H	4.351423	3.850309	1.218178

C	3.127237	3.780772	2.996624
H	3.902360	4.171381	3.673644
H	2.513383	3.067301	3.569864
H	2.476809	4.626473	2.724983
C	2.994151	-2.027514	-1.601216
H	2.170026	-2.204954	-0.891077
C	2.442911	-2.174449	-3.027600
H	3.258766	-2.002688	-3.749984
H	2.105466	-3.210067	-3.192937
C	1.296596	-1.225001	-3.343482
H	1.574702	-0.182946	-3.106973
H	0.385484	-1.515881	-2.791506
H	1.011275	-1.247268	-4.405236
C	4.084958	-3.063530	-1.292433
H	4.910867	-2.951305	-2.016929
H	4.517606	-2.811504	-0.310849
C	3.600305	-4.507305	-1.277120
H	3.248373	-4.840734	-2.265655
H	2.769873	-4.637068	-0.565100
H	4.406318	-5.192499	-0.972836
C	-2.889195	-0.182117	-0.318054
C	-3.481129	0.616555	-1.343776
C	-4.506469	0.095875	-2.139253
H	-4.950532	0.725368	-2.916179
C	-4.974163	-1.202758	-1.967100
H	-5.779526	-1.592030	-2.594297
C	-4.390388	-2.000801	-0.985848
H	-4.738288	-3.029736	-0.856774
C	-3.365827	-1.523484	-0.166256
C	-2.726093	-2.459854	0.835841
H	-2.053720	-1.841376	1.447152
C	-1.857691	-3.511455	0.127262
H	-1.397658	-4.175371	0.876659
H	-2.504244	-4.150687	-0.498870
C	-0.758365	-2.915691	-0.736603
H	-0.019193	-2.382891	-0.112620
H	-1.205718	-2.237322	-1.486495
H	-0.195564	-3.681388	-1.289955
C	-3.748195	-3.112181	1.776653
H	-4.455084	-2.335394	2.106771
H	-4.351693	-3.849731	1.218677
C	-3.127338	-3.780394	2.997029
H	-2.477312	-4.626424	2.725452
H	-3.902540	-4.170555	3.674217
H	-2.513109	-3.067083	3.570069
C	-2.994213	2.027383	-1.601325
H	-2.170108	2.204900	-0.891181
C	-2.442878	2.174056	-3.027719
H	-3.258735	2.002352	-3.750114
H	-2.105254	3.209602	-3.193149
C	-1.296727	1.224360	-3.343485
H	-0.385534	1.515108	-2.791584
H	-1.011453	1.246408	-4.405253
H	-1.575059	0.182384	-3.106870
C	-4.084982	3.063515	-1.292819
H	-4.910863	2.951164	-2.017329
H	-4.517689	2.811737	-0.311197
C	-3.600274	4.507273	-1.277822
H	-3.248304	4.840460	-2.266424
H	-2.769857	4.637173	-0.565809
H	-4.406274	5.192565	-0.973719

220

[K(^{DIPeP}NN)Ca]$_2$ diradical OPEN SINGLET

Ca	-0.000000	-0.000000	2.452253
K	0.000000	2.935150	0.000000
Si	-1.371514	-1.641738	5.322511
N	-0.634011	-1.769310	3.757055
C	-0.246267	-2.960019	3.172078
C	-1.193430	-3.882562	2.610534
C	-0.760690	-5.055972	1.987344
H	-1.504705	-5.741504	1.573288
C	0.594234	-5.362470	1.856390
H	0.913740	-6.278707	1.355777
C	1.528472	-4.476397	2.386370
H	2.592863	-4.716401	2.310889
C	1.144117	-3.303983	3.050270
C	-2.670127	-3.552193	2.612994
H	-2.814983	-2.776218	3.373181
C	-3.569102	-4.739132	2.975526
H	-4.610331	-4.381706	3.021025
H	-3.554079	-5.485662	2.160464
C	-3.219000	-5.406851	4.297544
H	-3.913559	-6.230187	4.524309
H	-3.270184	-4.688506	5.129657
H	-2.199238	-5.819848	4.285189
C	-3.061500	-2.910233	1.270780
H	-2.398516	-2.039815	1.101686
H	-2.857299	-3.623627	0.449608
C	-4.505663	-2.437910	1.182543
H	-5.216176	-3.277426	1.175340
H	-4.675431	-1.858761	0.262874
H	-4.762134	-1.793257	2.037822
C	2.216812	-2.508834	3.770219
H	1.713982	-1.634995	4.213127
C	2.759198	-3.355399	4.938638
H	1.896769	-3.802562	5.457271
H	3.331004	-4.205648	4.526478
C	3.614637	-2.598466	5.945808
H	3.935846	-3.263443	6.762066
H	3.057257	-1.764462	6.397674
H	4.525252	-2.177489	5.492844
C	3.340537	-1.996989	2.860168
H	3.712850	-2.818056	2.223951
H	4.195193	-1.696493	3.486493
C	2.942863	-0.814035	1.995751
H	2.125618	-1.040061	1.280533
H	3.772310	-0.454084	1.371209
H	2.639138	0.048251	2.617661
C	-0.759291	-0.103361	6.235482
H	-1.282933	0.781314	5.840266
H	-1.148198	-0.220569	7.262891
C	-3.262394	-1.433599	5.347319
H	-3.587043	-0.675098	4.617256
H	-3.568811	-1.077231	6.344842
H	-3.821880	-2.358303	5.143362
C	-0.903535	-3.142388	6.365847
H	-0.988830	-4.076338	5.790776
H	-1.540583	-3.227447	7.260427
H	0.141159	-3.051158	6.702456
Si	1.371514	1.641738	5.322511
N	0.634011	1.769310	3.757055
C	0.246267	2.960019	3.172078
C	1.193430	3.882562	2.610534
C	0.760690	5.055972	1.987344

H	1.504705	5.741504	1.573288
C	-0.594234	5.362470	1.856390
H	-0.913740	6.278707	1.355777
C	-1.528472	4.476397	2.386370
H	-2.592863	4.716401	2.310889
C	-1.144117	3.303983	3.050270
C	2.670127	3.552193	2.612994
H	2.814983	2.776218	3.373181
C	3.569102	4.739132	2.975526
H	4.610331	4.381706	3.021025
H	3.554079	5.485662	2.160464
C	3.219000	5.406851	4.297544
H	3.913559	6.230187	4.524309
H	3.270184	4.688506	5.129657
H	2.199238	5.819848	4.285189
C	3.061500	2.910233	1.270780
H	2.398516	2.039815	1.101686
H	2.857299	3.623627	0.449608
C	4.505663	2.437910	1.182543
H	5.216176	3.277426	1.175340
H	4.675431	1.858761	0.262874
H	4.762134	1.793257	2.037822
C	-2.216812	2.508834	3.770219
H	-1.713982	1.634995	4.213127
C	-2.759198	3.355399	4.938638
H	-1.896769	3.802562	5.457271
H	-3.331004	4.205648	4.526478
C	-3.614637	2.598466	5.945808
H	-3.935846	3.263443	6.762066
H	-3.057257	1.764462	6.397674
H	-4.525252	2.177489	5.492844
C	-3.340537	1.996989	2.860168
H	-3.712850	2.818056	2.223951
H	-4.195193	1.696493	3.486493
C	-2.942863	0.814035	1.995751
H	-2.125618	1.040061	1.280533
H	-3.772310	0.454084	1.371209
H	-2.639138	-0.048251	2.617661
C	0.759291	0.103361	6.235482
H	1.282933	-0.781314	5.840266
H	1.148198	0.220569	7.262891
C	3.262394	1.433599	5.347319
H	3.587043	0.675098	4.617256
H	3.568811	1.077231	6.344842
H	3.821880	2.358303	5.143362
C	0.903535	3.142388	6.365847
H	0.988830	4.076338	5.790776
H	1.540583	3.227447	7.260427
H	-0.141159	3.051158	6.702456
K	-0.000000	-2.935150	0.000000
Ca	0.000000	-0.000000	-2.452253
Si	-1.371514	1.641738	-5.322511
N	-0.634011	1.769310	-3.757055
C	-0.246267	2.960019	-3.172078
C	-1.193430	3.882562	-2.610534
C	-0.760690	5.055972	-1.987344
H	-1.504705	5.741504	-1.573288
C	0.594234	5.362470	-1.856390
H	0.913740	6.278707	-1.355777
C	1.528472	4.476397	-2.386370
H	2.592863	4.716401	-2.310889
C	1.144117	3.303983	-3.050270
C	-2.670127	3.552193	-2.612994
H	-2.814983	2.776218	-3.373181
C	-3.569102	4.739132	-2.975526
H	-4.610331	4.381706	-3.021025
H	-3.554079	5.485662	-2.160464
C	-3.219000	5.406851	-4.297544
H	-3.913559	6.230187	-4.524309
H	-3.270184	4.688506	-5.129657
H	-2.199238	5.819848	-4.285189
C	-3.061500	2.910233	-1.270780
H	-2.398516	2.039815	-1.101686
H	-2.857299	3.623627	-0.449608
C	-4.505663	2.437910	-1.182543
H	-5.216176	3.277426	-1.175340
H	-4.675431	1.858761	-0.262874
H	-4.762134	1.793257	-2.037822
C	2.216812	2.508834	-3.770219
H	1.713982	1.634995	-4.213127
C	2.759198	3.355399	-4.938638
H	1.896769	3.802562	-5.457271
H	3.331004	4.205648	-4.526478
C	3.614637	2.598466	-5.945808
H	3.935846	3.263443	-6.762066
H	3.057257	1.764462	-6.397674
H	4.525252	2.177489	-5.492844
C	3.340537	1.996989	-2.860168
H	3.712850	2.818056	-2.223951
H	4.195193	1.696493	-3.486493
C	2.942863	0.814035	-1.995751
H	2.125618	1.040061	-1.280533
H	3.772310	0.454084	-1.371209
H	2.639138	-0.048251	-2.617661
C	-0.759291	0.103361	-6.235482
H	-1.282933	-0.781314	-5.840266
H	-1.148198	0.220569	-7.262891
C	-3.262394	1.433599	-5.347319
H	-3.587043	0.675098	-4.617256
H	-3.568811	1.077231	-6.344842
H	-3.821880	2.358303	-5.143362
C	-0.903535	3.142388	-6.365847
H	-0.988830	4.076338	-5.790776
H	-1.540583	3.227447	-7.260427
H	0.141159	3.051158	-6.702456
Si	1.371514	-1.641738	-5.322511
N	0.634011	-1.769310	-3.757055
C	0.246267	-2.960019	-3.172078
C	1.193430	-3.882562	-2.610534
C	0.760690	-5.055972	-1.987344
H	1.504705	-5.741504	-1.573288
C	-0.594234	-5.362470	-1.856390
H	-0.913740	-6.278707	-1.355777
C	-1.528472	-4.476397	-2.386370
H	-2.592863	-4.716401	-2.310889
C	-1.144117	-3.303983	-3.050270
C	2.670127	-3.552193	-2.612994
H	2.814983	-2.776218	-3.373181
C	3.569102	-4.739132	-2.975526
H	4.610331	-4.381706	-3.021025
H	3.554079	-5.485662	-2.160464
C	3.219000	-5.406851	-4.297544
H	3.913559	-6.230187	-4.524309
H	3.270184	-4.688506	-5.129657
H	2.199238	-5.819848	-4.285189
C	3.061500	-2.910233	-1.270780

H	2.398516	-2.039815	-1.101686
H	2.857299	-3.623627	-0.449608
C	4.505663	-2.437910	-1.182543
H	5.216176	-3.277426	-1.175340
H	4.675431	-1.858761	-0.262874
H	4.762134	-1.793257	-2.037822
C	-2.216812	-2.508834	-3.770219
H	-1.713982	-1.634995	-4.213127
C	-2.759198	-3.355399	-4.938638
H	-1.896769	-3.802562	-5.457271
H	-3.331004	-4.205648	-4.526478
C	-3.614637	-2.598466	-5.945808
H	-3.935846	-3.263443	-6.762066
H	-3.057257	-1.764462	-6.397674
H	-4.525252	-2.177489	-5.492844
C	-3.340537	-1.996989	-2.860168
H	-3.712850	-2.818056	-2.223951
H	-4.195193	-1.696493	-3.486493
C	-2.942863	-0.814035	-1.995751
H	-2.125618	-1.040061	-1.280533
H	-3.772310	-0.454084	-1.371209
H	-2.639138	0.048251	-2.617661
C	0.759291	-0.103361	-6.235482
H	1.282933	0.781314	-5.840266
H	1.148198	-0.220569	-7.262891
C	3.262394	-1.433599	-5.347319
H	3.587043	-0.675098	-4.617256
H	3.568811	-1.077231	-6.344842
H	3.821880	-2.358303	-5.143362
C	0.903535	-3.142388	-6.365847
H	0.988830	-4.076338	-5.790776
H	1.540583	-3.227447	-7.260427
H	-0.141159	-3.051158	-6.702456

220

[K(^{DIPeP}NN)Ca]$_2$ diradical TRIPLET

Ca	2.194064	-0.000000	-0.000000
K	0.000000	3.370315	0.000000
Si	5.129963	-1.565867	1.412282
N	3.575416	-1.735423	0.647478
C	3.103365	-2.976681	0.257098
C	2.674114	-3.972394	1.205162
C	2.186900	-5.207177	0.770166
H	1.868920	-5.943703	1.512463
C	2.065012	-5.515417	-0.585838
H	1.670418	-6.481795	-0.906634
C	2.481700	-4.567586	-1.518706
H	2.420277	-4.807451	-2.584041
C	3.015551	-3.331543	-1.132515
C	2.650237	-3.644492	2.682311
H	3.404238	-2.867165	2.844262
C	2.992216	-4.820624	3.601451
H	2.970192	-4.459547	4.642022
H	2.196988	-5.586210	3.546370
C	4.348986	-5.456103	3.334308
H	4.548964	-6.278012	4.038782
H	5.158513	-4.718556	3.443751
H	4.414177	-5.863838	2.314489
C	1.301468	-3.000721	3.046562
H	1.109817	-2.182248	2.326248
H	0.492570	-3.742592	2.901633
C	1.225851	-2.435925	4.457999
H	1.275469	-3.224474	5.223140
H	0.285056	-1.889528	4.618935
H	2.057064	-1.738685	4.646332
C	3.639948	-2.463240	-2.209017
H	4.059880	-1.585385	-1.694269
C	4.817885	-3.231424	-2.841196
H	5.407572	-3.670450	-2.020602
H	4.419546	-4.089778	-3.409936
C	5.736391	-2.410683	-3.735721
H	6.553658	-3.035042	-4.128078
H	6.192327	-1.575892	-3.184746
H	5.210100	-1.983314	-4.602803
C	2.646832	-1.959073	-3.264203
H	2.018939	-2.794366	-3.618452
H	3.206341	-1.611062	-4.146698
C	1.764604	-0.820124	-2.782275
H	1.112033	-1.128654	-1.934852
H	1.076933	-0.466684	-3.561383
H	2.373622	0.058869	-2.500738
C	6.092606	-0.075017	0.761341
H	5.745839	0.846526	1.253253
H	7.114951	-0.233009	1.150560
C	5.128298	-1.292499	3.297338
H	4.359008	-0.564342	3.598351
H	6.104106	-0.875868	3.595717
H	4.972952	-2.208427	3.885949
C	6.178530	-3.084186	1.017630
H	5.621321	-4.019358	1.170128
H	7.087622	-3.116312	1.638788
H	6.490954	-3.053288	-0.038294
Si	5.129963	1.565867	-1.412282
N	3.575416	1.735423	-0.647478
C	3.103365	2.976681	-0.257098
C	2.674114	3.972394	-1.205162
C	2.186900	5.207177	-0.770166
H	1.868920	5.943703	-1.512463
C	2.065012	5.515417	0.585838
H	1.670418	6.481795	0.906634
C	2.481700	4.567586	1.518706
H	2.420277	4.807451	2.584041
C	3.015551	3.331543	1.132515
C	2.650237	3.644492	-2.682311
H	3.404238	2.867165	-2.844262
C	2.992216	4.820624	-3.601451
H	2.970192	4.459547	-4.642022
H	2.196988	5.586210	-3.546370
C	4.348986	5.456103	-3.334308
H	4.548964	6.278012	-4.038782
H	5.158513	4.718556	-3.443751
H	4.414177	5.863838	-2.314489
C	1.301468	3.000721	-3.046562
H	1.109817	2.182248	-2.326248
H	0.492570	3.742592	-2.901633
C	1.225851	2.435925	-4.457999
H	1.275469	3.224474	-5.223140
H	0.285056	1.889528	-4.618935
H	2.057064	1.738685	-4.646332
C	3.639948	2.463240	2.209017
H	4.059880	1.585385	1.694269
C	4.817885	3.231424	2.841196
H	5.407572	3.670450	2.020602
H	4.419546	4.089778	3.409936
C	5.736391	2.410683	3.735721
H	6.553658	3.035042	4.128078

H	6.192327	1.575892	3.184746
H	5.210100	1.983314	4.602803
C	2.646832	1.959073	3.264203
H	2.018939	2.794366	3.618452
H	3.206341	1.611062	4.146698
C	1.764604	0.820124	2.782275
H	1.112033	1.128654	1.934852
H	1.076933	0.466684	3.561383
H	2.373622	-0.058869	2.500738
C	6.092606	0.075017	-0.761341
H	5.745839	-0.846526	-1.253253
H	7.114951	0.233009	-1.150560
C	5.128298	1.292499	-3.297338
H	4.359008	0.564342	-3.598351
H	6.104106	0.875868	-3.595717
H	4.972952	2.208427	-3.885949
C	6.178530	3.084186	-1.017630
H	5.621321	4.019358	-1.170128
H	7.087622	3.116312	-1.638788
H	6.490954	3.053288	0.038294
K	-0.000000	-3.370315	0.000000
Ca	-2.194064	0.000000	-0.000000
Si	-5.129963	1.565867	1.412282
N	-3.575416	1.735423	0.647478
C	-3.103365	2.976681	0.257098
C	-2.674114	3.972394	1.205162
C	-2.186900	5.207177	0.770166
H	-1.868920	5.943703	1.512463
C	-2.065012	5.515417	-0.585838
H	-1.670418	6.481795	-0.906634
C	-2.481700	4.567586	-1.518706
H	-2.420277	4.807451	-2.584041
C	-3.015551	3.331543	-1.132515
C	-2.650237	3.644492	2.682311
H	-3.404238	2.867165	2.844262
C	-2.992216	4.820624	3.601451
H	-2.970192	4.459547	4.642022
H	-2.196988	5.586210	3.546370
C	-4.348986	5.456103	3.334308
H	-4.548964	6.278012	4.038782
H	-5.158513	4.718556	3.443751
H	-4.414177	5.863838	2.314489
C	-1.301468	3.000721	3.046562
H	-1.109817	2.182248	2.326248
H	-0.492570	3.742592	2.901633
C	-1.225851	2.435925	4.457999
H	-1.275469	3.224474	5.223140
H	-0.285056	1.889528	4.618935
H	-2.057064	1.738685	4.646332
C	-3.639948	2.463240	-2.209017
H	-4.059880	1.585385	-1.694269
C	-4.817885	3.231424	-2.841196
H	-5.407572	3.670450	-2.020602
H	-4.419546	4.089778	-3.409936
C	-5.736391	2.410683	-3.735721
H	-6.553658	3.035042	-4.128078
H	-6.192327	1.575892	-3.184746
H	-5.210100	1.983314	-4.602803
C	-2.646832	1.959073	-3.264203
H	-2.018939	2.794366	-3.618452
H	-3.206341	1.611062	-4.146698
C	-1.764604	0.820124	-2.782275
H	-1.112033	1.128654	-1.934852

H	-1.076933	0.466684	-3.561383
H	-2.373622	-0.058869	-2.500738
C	-6.092606	0.075017	0.761341
H	-5.745839	-0.846526	1.253253
H	-7.114951	0.233009	1.150560
C	-5.128298	1.292499	3.297338
H	-4.359008	0.564342	3.598351
H	-6.104106	0.875868	3.595717
H	-4.972952	2.208427	3.885949
C	-6.178530	3.084186	1.017630
H	-5.621321	4.019358	1.170128
H	-7.087622	3.116312	1.638788
H	-6.490954	3.053288	-0.038294
Si	-5.129963	-1.565867	-1.412282
N	-3.575416	-1.735423	-0.647478
C	-3.103365	-2.976681	-0.257098
C	-2.674114	-3.972394	-1.205162
C	-2.186900	-5.207177	-0.770166
H	-1.868920	-5.943703	-1.512463
C	-2.065012	-5.515417	0.585838
H	-1.670418	-6.481795	0.906634
C	-2.481700	-4.567586	1.518706
H	-2.420277	-4.807451	2.584041
C	-3.015551	-3.331543	1.132515
C	-2.650237	-3.644492	-2.682311
H	-3.404238	-2.867165	-2.844262
C	-2.992216	-4.820624	-3.601451
H	-2.970192	-4.459547	-4.642022
H	-2.196988	-5.586210	-3.546370
C	-4.348986	-5.456103	-3.334308
H	-4.548964	-6.278012	-4.038782
H	-5.158513	-4.718556	-3.443751
H	-4.414177	-5.863838	-2.314489
C	-1.301468	-3.000721	-3.046562
H	-1.109817	-2.182248	-2.326248
H	-0.492570	-3.742592	-2.901633
C	-1.225851	-2.435925	-4.457999
H	-1.275469	-3.224474	-5.223140
H	-0.285056	-1.889528	-4.618935
H	-2.057064	-1.738685	-4.646332
C	-3.639948	-2.463240	2.209017
H	-4.059880	-1.585385	1.694269
C	-4.817885	-3.231424	2.841196
H	-5.407572	-3.670450	2.020602
H	-4.419546	-4.089778	3.409936
C	-5.736391	-2.410683	3.735721
H	-6.553658	-3.035042	4.128078
H	-6.192327	-1.575892	3.184746
H	-5.210100	-1.983314	4.602803
C	-2.646832	-1.959073	3.264203
H	-2.018939	-2.794366	3.618452
H	-3.206341	-1.611062	4.146698
C	-1.764604	-0.820124	2.782275
H	-1.112033	-1.128654	1.934852
H	-1.076933	-0.466684	3.561383
H	-2.373622	0.058869	2.500738
C	-6.092606	-0.075017	-0.761341
H	-5.745839	0.846526	-1.253253
H	-7.114951	-0.233009	-1.150560
C	-5.128298	-1.292499	-3.297338
H	-4.359008	-0.564342	-3.598351
H	-6.104106	-0.875868	-3.595717
H	-4.972952	-2.208427	-3.885949

C	-6.178530	-3.084186	-1.017630
H	-5.621321	-4.019358	-1.170128
H	-7.087622	-3.116312	-1.638788
H	-6.490954	-3.053288	0.038294

109

$(^{DIPeP}NN)Sr$ (5-Sr)

Sr	0.000000	0.000000	0.889358
Si	0.591542	2.100983	-2.134321
N	0.036543	1.887824	-0.520007
C	0.659925	0.399668	-2.961268
H	1.025232	0.555734	-3.991077
H	1.469690	-0.161217	-2.461925
C	2.349597	2.804647	-2.222131
H	2.390708	3.848822	-1.875432
H	2.729727	2.782440	-3.256955
H	3.040727	2.217786	-1.596783
C	-0.520834	3.263767	-3.127062
H	-1.467702	2.781332	-3.413726
H	-0.020217	3.595263	-4.050916
H	-0.767008	4.159526	-2.533774
C	-0.672856	2.684418	0.359207
C	-2.109597	2.691559	0.368974
C	-2.795889	3.349613	1.394691
H	-3.888135	3.356404	1.385408
C	-2.123983	3.998209	2.429489
H	-2.681963	4.493705	3.227262
C	-0.732138	4.020584	2.418300
H	-0.197538	4.554518	3.209760
C	0.000000	3.404160	1.399064
C	1.507741	3.533748	1.371222
H	1.833005	3.107686	0.410217
C	1.953006	5.003957	1.402480
H	1.766047	5.433027	2.403811
H	3.045003	5.044089	1.259589
C	1.279602	5.861950	0.339814
H	1.672914	6.889955	0.348415
H	0.191557	5.912671	0.493694
H	1.446335	5.446866	-0.666287
C	2.161812	2.694457	2.480265
H	1.716273	2.957557	3.455596
H	1.889811	1.630414	2.317446
C	3.678534	2.795814	2.559620
H	4.096237	2.042165	3.244090
H	3.996775	3.783441	2.923798
H	4.137587	2.645574	1.570031
C	-2.864166	1.982140	-0.734616
H	-2.135686	1.830193	-1.541407
C	-3.996181	2.839164	-1.317450
H	-4.776666	3.026934	-0.560660
H	-3.570579	3.827953	-1.554321
C	-4.637566	2.250005	-2.566973
H	-5.195809	1.325445	-2.352918
H	-3.877836	2.006754	-3.327297
H	-5.346721	2.959405	-3.020129
C	-3.325800	0.568233	-0.334156
H	-3.765957	0.084000	-1.220352
H	-2.441205	-0.065289	-0.125957
C	-4.301775	0.482549	0.828232
H	-3.858442	0.852248	1.765674
H	-4.626079	-0.553731	0.994875
H	-5.205330	1.081416	0.636815
Si	-0.591542	-2.100983	-2.134321
N	-0.036543	-1.887824	-0.520007
C	-0.659925	-0.399668	-2.961268
H	-1.025232	-0.555734	-3.991077
H	-1.469690	0.161217	-2.461925
C	-2.349597	-2.804647	-2.222131
H	-2.390708	-3.848822	-1.875432
H	-2.729727	-2.782440	-3.256955
H	-3.040727	-2.217786	-1.596783
C	0.520834	-3.263767	-3.127062
H	1.467702	-2.781332	-3.413726
H	0.020217	-3.595263	-4.050916
H	0.767008	-4.159526	-2.533774
C	0.672856	-2.684418	0.359207
C	2.109597	-2.691559	0.368974
C	2.795889	-3.349613	1.394691
H	3.888135	-3.356404	1.385408
C	2.123983	-3.998209	2.429489
H	2.681963	-4.493705	3.227262
C	0.732138	-4.020584	2.418300
H	0.197538	-4.554518	3.209760
C	-0.000000	-3.404160	1.399064
C	-1.507741	-3.533748	1.371222
H	-1.833005	-3.107686	0.410217
C	-1.953006	-5.003957	1.402480
H	-1.766047	-5.433027	2.403811
H	-3.045003	-5.044089	1.259589
C	-1.279602	-5.861950	0.339814
H	-1.672914	-6.889955	0.348415
H	-0.191557	-5.912671	0.493694
H	-1.446335	-5.446866	-0.666287
C	-2.161812	-2.694457	2.480265
H	-1.716273	-2.957557	3.455596
H	-1.889811	-1.630414	2.317446
C	-3.678534	-2.795814	2.559620
H	-4.096237	-2.042165	3.244090
H	-3.996775	-3.783441	2.923798
H	-4.137587	-2.645574	1.570031
C	2.864166	-1.982140	-0.734616
H	2.135686	-1.830193	-1.541407
C	3.996181	-2.839164	-1.317450
H	4.776666	-3.026934	-0.560660
H	3.570579	-3.827953	-1.554321
C	4.637566	-2.250005	-2.566973
H	5.195809	-1.325445	-2.352918
H	3.877836	-2.006754	-3.327297
H	5.346721	-2.959405	-3.020129
C	3.325800	-0.568233	-0.334156
H	3.765957	-0.084000	-1.220352
H	2.441205	0.065289	-0.125957
C	4.301775	-0.482549	0.828232
H	3.858442	-0.852248	1.765674
H	4.626079	0.553731	0.994875
H	5.205330	-1.081416	0.636815

220

$[K(^{DIPeP}NN)Sr]_2$ diradical OPEN SINGLET

C	4.095934	6.239293	2.795950
C	5.238157	1.740206	3.009099
C	6.246329	-2.210296	3.384879
C	6.206301	3.569377	0.737539
C	2.857128	5.421740	3.134884
C	1.662638	2.742844	4.228753
C	5.228501	-3.097620	2.681568

C	6.070776	0.485210	0.478948
C	2.717549	4.135471	2.319950
C	1.545011	3.262771	2.803315
C	6.682849	-2.798894	-0.782470
C	5.990320	0.185927	-1.024680
C	3.089431	-1.914423	3.372538
C	2.589570	4.336454	0.824466
C	3.958650	-2.371014	2.194913
C	1.962344	5.476335	0.317145
C	3.016068	3.281288	-0.057694
C	5.291930	3.563247	-3.021840
C	2.043908	-0.873086	3.016791
C	3.250957	-3.258592	1.188566
C	1.698295	5.630389	-1.046317
C	2.707499	3.446774	-1.452993
C	2.645091	-4.431832	1.654595
C	5.480506	-1.628265	-3.340354
C	-0.950029	-3.274576	4.520050
C	2.067691	4.603762	-1.912361
C	4.025459	2.913200	-3.556183
C	3.351719	-2.999709	-0.225700
C	3.086844	2.383812	-2.462735
C	-1.968023	0.038636	3.754641
C	-1.809992	1.364021	3.028875
C	-1.992631	5.402110	1.290438
C	-2.220158	4.263082	2.059696
C	2.147340	-5.400873	0.785735
C	-1.078939	-3.512792	3.022086
C	4.679402	-5.521383	-2.930135
C	2.822612	-4.008378	-1.102190
C	-4.074786	2.372362	3.599183
C	-3.106935	1.970809	2.474534
C	2.255864	-5.177678	-0.587180
C	-2.417101	5.389245	-0.038377
C	-2.834621	3.124138	1.530852
C	1.844118	1.708831	-3.064365
C	2.815549	-3.791563	-2.599509
C	2.100260	0.407545	-3.807389
C	-5.379428	2.978771	3.105626
C	-2.917011	-5.257339	3.329880
C	3.323147	-5.004039	-3.389338
C	-2.453809	-4.019062	2.550811
C	-3.036961	4.275715	-0.614765
C	-3.261762	3.078199	0.153295
C	1.419163	-3.333333	-3.054561
C	-1.783431	-5.358304	0.545782
C	-2.441210	-4.233264	1.052617
C	-2.248808	4.203063	-3.015727
C	-4.233039	-5.847774	2.843205
C	-5.279608	-2.062080	3.272131
C	-1.780894	2.764115	-3.169292
C	-3.448919	4.371202	-2.067779
C	-4.230319	5.660434	-2.371051
C	-1.657075	-5.584150	-0.824618
C	1.280626	-3.052135	-4.544000
C	-3.046329	-3.274956	0.169171
C	-2.233600	-4.666472	-1.700680
C	-1.960599	-1.047665	-3.104118
C	-5.919816	-0.296470	0.959810
C	-2.929686	-3.541254	-1.243070
C	-5.100299	5.559131	-3.616644
C	-6.543483	3.054781	-0.675208
C	-6.003612	0.013909	-0.540601

C	-2.880796	-2.207175	-3.440170
C	-3.708035	-2.724576	-2.256717
C	-6.386716	-3.322237	0.707022
C	-5.318949	1.446158	-3.011015
C	-4.917761	-3.551748	-2.735509
C	-6.011443	-2.740722	-3.416856
H	4.153160	7.153556	3.406430
H	5.011576	5.656749	2.982516
H	6.243301	1.379220	3.283416
H	7.143426	3.582733	1.316401
H	7.127555	-2.793122	3.693606
H	5.033943	2.633276	3.617868
H	2.893445	5.149993	4.201867
H	5.837131	-1.734290	4.289232
H	6.596355	-1.405538	2.721013
H	4.109660	6.539745	1.737415
H	5.645432	4.487097	0.969800
H	4.522255	0.957364	3.306678
H	1.624058	3.552033	4.973126
H	7.120232	0.569254	0.811448
H	1.947202	6.039278	3.027480
H	2.610147	2.202310	4.372585
H	6.462276	3.601371	-0.331466
H	3.627156	3.546238	2.487246
H	4.925486	-3.927249	3.345062
H	0.843877	2.045433	4.460685
H	3.744989	-1.497378	4.153268
H	6.967316	-2.543348	0.249560
H	5.702959	-3.573044	1.810015
H	5.672558	-0.361992	1.062944
H	6.968285	0.330840	-1.514421
H	7.597473	-2.797034	-1.396553
H	4.294102	-1.477823	1.644074
H	0.605777	3.835488	2.693784
H	1.650675	6.263660	1.007072
H	5.056719	4.409430	-2.359216
H	2.514886	0.062636	2.663980
H	1.467071	2.393424	2.124688
H	6.276400	-3.822270	-0.776635
H	5.888512	2.843741	-2.441839
H	2.600015	-2.788341	3.834751
H	5.319281	0.895444	-1.537692
H	5.923468	3.937655	-3.841634
H	1.430110	-0.584884	3.882734
H	-0.003019	-2.765579	4.755988
H	6.453947	-1.228329	-3.670557
H	2.590220	-4.605975	2.732862
H	-1.326513	2.101320	3.690864
H	1.331694	-1.217444	2.243692
H	-1.509435	6.282992	1.717628
H	-0.989786	-0.345359	4.075641
H	3.668656	1.632433	-1.901536
H	-1.927146	4.262238	3.113362
H	1.210449	6.532409	-1.421524
H	-0.965927	-4.216404	5.088096
H	-1.771354	-2.645612	4.896205
H	-2.604858	0.119807	4.647410
H	5.382640	-2.639658	-3.761024
H	3.470819	3.630693	-4.187086
H	-3.560819	3.081699	4.272304
H	4.697045	-0.999614	-3.790781
H	-1.082914	1.214275	2.200199
H	5.445720	-4.733915	-2.990001

H	4.296509	2.076993	-4.221548
H	1.864779	4.706666	-2.982321
H	4.648200	-5.865665	-1.885691
H	-0.295170	-4.224659	2.702517
H	1.692570	-6.316472	1.169490
H	-0.871815	-2.560173	2.498577
H	-4.294216	1.481590	4.210311
H	-2.417571	-0.730355	3.103089
H	5.016963	-6.363188	-3.553931
H	-2.254363	6.277582	-0.654544
H	3.493609	-2.952569	-2.795256
H	2.578504	-0.347542	-3.159032
H	1.101747	1.498336	-2.262363
H	-3.017399	-4.981231	4.392054
H	-2.128815	-6.032066	3.302612
H	-6.028406	3.263023	3.947704
H	1.336764	2.428031	-3.729327
H	2.757625	0.542475	-4.678501
H	1.870928	-5.929618	-1.281518
H	-3.644736	1.208179	1.884884
H	-5.197065	3.877135	2.497214
H	-3.166795	-3.208756	2.744845
H	-1.421419	4.847919	-2.665411
H	-1.341728	-6.072302	1.246518
H	3.386915	-4.722931	-4.452986
H	-4.559225	-1.358352	3.716806
H	2.577662	-5.819288	-3.346988
H	1.169801	-2.407482	-2.500645
H	-5.935444	2.263615	2.481877
H	1.155895	-0.029017	-4.162246
H	-1.555047	2.281954	-2.203517
H	-3.528385	6.506623	-2.475910
H	-4.535064	-6.707798	3.460338
H	-0.881084	2.691061	-3.796208
H	-5.088356	-3.055495	3.702796
H	2.070340	-2.369816	-4.894562
H	0.667932	-4.088712	-2.756519
H	-4.864175	5.904948	-1.503076
H	-1.130646	-6.463687	-1.201027
H	-6.286740	-1.751122	3.597039
H	-5.043345	-5.105030	2.887783
H	-1.212093	-1.290414	-2.326083
H	-5.308716	0.457701	1.484165
H	-2.517014	4.588730	-4.013540
H	-4.161386	-6.188855	1.799726
H	-4.122938	3.531344	-2.267947
H	1.345775	-3.971793	-5.144029
H	-2.565077	2.149023	-3.631537
H	0.309783	-2.583896	-4.766441
H	-6.151293	4.052513	-0.921521
H	-1.382890	-0.703304	-3.975059
H	-6.750708	3.040816	0.405234
H	-2.539545	-0.168024	-2.769210
H	-6.911474	-0.225764	1.438063
H	-5.610724	6.511021	-3.829087
H	-4.512617	5.295976	-4.510438
H	-2.168598	-4.845994	-2.777558
H	-2.293364	-3.033434	-3.874511
H	-4.116740	-1.858468	-1.712387
H	-5.461436	-0.751710	-1.122935
H	-5.874878	4.786016	-3.492847
H	-5.907452	-4.313276	0.693030
H	-7.497753	2.921064	-1.209184
H	-4.586995	0.672681	-3.295750
H	-7.299231	-3.391647	1.320383
H	-5.101097	2.347095	-3.601967
H	-7.042402	-0.060360	-0.906106
H	-3.569573	-1.884684	-4.237033
H	-6.688953	-3.079528	-0.323075
H	-5.341782	-4.071516	-1.863452
H	-6.310339	1.077614	-3.323284
H	-4.556225	-4.347683	-3.410957
H	-6.415203	-1.973641	-2.738656
H	-5.651261	-2.223078	-4.319391
H	-6.847790	-3.387158	-3.723788
K	-0.146089	3.312894	-0.108264
K	0.153659	-3.136271	0.000892
N	3.621251	2.136425	0.398402
N	3.871989	-1.814629	-0.693957
N	-3.789463	1.925925	-0.367094
N	-3.656032	-2.127278	0.626197
Si	5.188953	2.032811	1.128328
Si	5.413384	-1.568160	-1.441131
Si	-5.213213	-2.003422	1.373449
Si	-5.327096	1.686080	-1.122267
Sr	2.384923	0.073778	-0.138960
Sr	-2.384013	-0.093906	0.020745

220

[K(^{DIPeP}NN)Sr]$_2$ diradical TRIPLET

C	4.209826	5.979573	3.195756
C	5.111494	1.443367	3.169704
C	6.014755	-2.329540	3.471941
C	6.151906	3.371611	1.009288
C	2.896418	5.234209	3.389999
C	1.549155	2.529870	4.257411
C	5.062263	-3.214170	2.679442
C	6.046145	0.339449	0.599796
C	2.736372	3.999592	2.501704
C	1.494061	3.173735	2.879205
C	6.461124	-2.876532	-0.901158
C	6.005151	0.140603	-0.919793
C	2.865797	-2.039722	3.177652
C	2.716178	4.276816	1.013169
C	3.840448	-2.488707	2.080346
C	2.255829	5.501672	0.526016
C	3.088925	3.220985	0.105630
C	5.483491	3.507047	-2.906317
C	1.906889	-0.939799	2.755708
C	3.216815	-3.367949	1.013156
C	2.113074	5.748421	-0.842310
C	2.912440	3.489453	-1.295666
C	2.719911	-4.616676	1.404960
C	5.388849	-1.408787	-3.374589
C	-0.908934	-3.103104	4.560606
C	2.437815	4.729073	-1.736115
C	4.189287	2.898499	-3.424339
C	3.268642	-3.004948	-0.378914
C	3.230190	2.424814	-2.324143
C	-1.910454	0.161970	3.621790
C	-1.801541	1.500099	2.907086
C	-2.196891	5.536860	1.113906
C	-2.366003	4.405713	1.911404
C	2.299505	-5.570044	0.478284
C	-1.093534	-3.462954	3.092926
C	4.668632	-5.327999	-3.327815

C	2.825090	-4.001695	-1.317016
C	-4.075162	2.460600	3.502338
C	-3.120376	2.070543	2.362656
C	2.374438	-5.249298	-0.877842
C	-2.574844	5.453308	-0.226851
C	-2.882735	3.213545	1.397791
C	1.932933	1.855245	-2.919327
C	2.768310	-3.683431	-2.794854
C	2.060063	0.544325	-3.680178
C	-5.395477	3.050258	3.029717
C	-2.999563	-5.084362	3.582319
C	3.282562	-4.814900	-3.692843
C	-2.509584	-3.924429	2.705890
C	-3.085518	4.279216	-0.790631
C	-3.251950	3.090607	0.008193
C	1.343644	-3.241969	-3.171785
C	-2.035823	-5.436245	0.769350
C	-2.556669	-4.221066	1.222999
C	-2.194169	4.124723	-3.154311
C	-4.358942	-5.645432	3.188828
C	-5.243898	-1.805886	3.316508
C	-1.659821	2.701648	-3.198283
C	-3.435690	4.299253	-2.263226
C	-4.239079	5.547255	-2.665685
C	-1.943008	-5.738843	-0.589272
C	1.160458	-2.828022	-4.625186
C	-3.054418	-3.239582	0.296526
C	-2.418443	-4.801149	-1.505383
C	-1.805643	-1.058785	-2.789612
C	-5.909653	-0.220681	0.898003
C	-2.982431	-3.586469	-1.099152
C	-5.063875	5.348684	-3.930246
C	-6.442698	3.002387	-0.902213
C	-5.970104	0.000552	-0.618178
C	-2.683684	-2.213415	-3.239697
C	-3.644249	-2.727234	-2.158265
C	-6.266407	-3.255808	0.803627
C	-5.158100	1.278897	-3.141250
C	-4.829031	-3.495661	-2.775658
C	-5.822667	-2.629809	-3.537997
H	4.274744	6.856504	3.858123
H	5.067018	5.326785	3.422615
H	6.113960	1.082407	3.452746
H	7.096247	3.326049	1.574610
H	6.850996	-2.919927	3.876654
H	4.879899	2.303343	3.814900
H	2.826945	4.911163	4.440946
H	5.521696	-1.836654	4.323888
H	6.442852	-1.537940	2.839608
H	4.332173	6.330930	2.160180
H	5.617900	4.286752	1.303247
H	4.400533	0.637927	3.411816
H	1.566018	3.274770	5.067044
H	7.083666	0.457066	0.959724
H	2.040686	5.917931	3.244664
H	2.449856	1.906896	4.360518
H	6.396127	3.463956	-0.059606
H	3.599961	3.350833	2.691878
H	4.705848	-4.042708	3.316907
H	0.677013	1.882302	4.430007
H	3.444192	-1.680332	4.043633
H	6.729633	-2.720259	0.155081
H	5.612341	-3.692043	1.854190

H	5.678077	-0.557758	1.122249
H	7.017656	0.184499	-1.357543
H	7.391859	-2.878918	-1.490216
H	4.210926	-1.591264	1.561894
H	0.598189	3.818344	2.801618
H	1.986174	6.288793	1.233815
H	5.285564	4.378479	-2.264773
H	2.455684	-0.021340	2.478434
H	1.374294	2.376994	2.122092
H	5.997293	-3.870953	-0.984638
H	6.052522	2.778800	-2.310018
H	2.293418	-2.909928	3.541640
H	5.453520	0.953277	-1.418504
H	6.127530	3.834065	-3.736569
H	1.229079	-0.646538	3.569320
H	0.068639	-2.627667	4.732575
H	6.393627	-1.045959	-3.648683
H	2.692996	-4.863500	2.470258
H	-1.353439	2.247408	3.581544
H	1.248631	-1.261159	1.919572
H	-1.801538	6.465898	1.529770
H	-0.921387	-0.192355	3.941735
H	3.755739	1.627712	-1.770534
H	-2.109593	4.456739	2.973569
H	1.764130	6.719201	-1.200925
H	-0.958039	-3.990142	5.209184
H	-1.687395	-2.402805	4.899799
H	-2.545126	0.223638	4.517566
H	5.232508	-2.364422	-3.895119
H	3.665388	3.625983	-4.070153
H	-3.561139	3.175943	4.168851
H	4.661228	-0.686107	-3.773887
H	-1.054106	1.423225	2.084704
H	5.412097	-4.517382	-3.351864
H	4.424497	2.039977	-4.075044
H	2.333330	4.905479	-2.810692
H	4.688337	-5.756348	-2.314848
H	-0.365955	-4.251066	2.820470
H	1.938412	-6.547885	0.804123
H	-0.845722	-2.580699	2.471625
H	-4.273641	1.567001	4.116619
H	-2.346801	-0.625203	2.981466
H	5.001399	-6.107115	-4.030681
H	-2.461265	6.335138	-0.863301
H	3.410406	-2.808282	-2.950546
H	2.495791	-0.261994	-3.063354
H	1.180261	1.728480	-2.108813
H	-3.045299	-4.732269	4.625652
H	-2.248283	-5.895490	3.582804
H	-6.034596	3.322921	3.883005
H	1.481601	2.622563	-3.570749
H	2.706197	0.639836	-4.564759
H	2.053848	-5.987621	-1.618258
H	-3.655591	1.295506	1.786188
H	-5.234444	3.952572	2.421371
H	-3.170788	-3.066076	2.875400
H	-1.409467	4.829604	-2.818791
H	-1.672707	-6.162158	1.502428
H	3.299053	-4.449369	-4.732340
H	-4.551635	-1.053307	3.724017
H	2.561165	-5.652701	-3.691621
H	1.070935	-2.391151	-2.517580
H	-5.952248	2.329868	2.412760

H	1.075661	0.196729	-4.024539
H	-1.412980	2.308237	-2.196437
H	-3.556086	6.404987	-2.798886
H	-4.673442	-6.442983	3.879347
H	-0.757718	2.626039	-3.822300
H	-5.035363	-2.760405	3.819996
H	1.917596	-2.087267	-4.924866
H	0.633542	-4.057052	-2.934092
H	-4.908427	5.825422	-1.835419
H	-1.527005	-6.691094	-0.925090
H	-6.264336	-1.499456	3.601421
H	-5.133501	-4.864559	3.206546
H	-1.162511	-1.339321	-1.927133
H	-5.356735	0.592569	1.396337
H	-2.441742	4.436893	-4.182884
H	-4.344663	-6.065248	2.172209
H	-4.074266	3.430837	-2.455397
H	1.247786	-3.684743	-5.309412
H	-2.414640	2.020176	-3.613441
H	0.168593	-2.380820	-4.789840
H	-6.050036	3.996926	-1.156684
H	-1.115984	-0.721331	-3.576279
H	-6.679763	3.009649	0.172837
H	-2.420898	-0.177625	-2.534206
H	-6.915854	-0.187575	1.349811
H	-5.590729	6.271967	-4.216175
H	-4.439989	5.048709	-4.787187
H	-2.379436	-5.034223	-2.573467
H	-2.051515	-3.043500	-3.598003
H	-4.061702	-1.857887	-1.628092
H	-5.472053	-0.824635	-1.152573
H	-5.821990	4.563286	-3.784262
H	-5.766514	-4.234406	0.862333
H	-7.382496	2.851780	-1.457032
H	-4.431409	0.480368	-3.362070
H	-7.198791	-3.308556	1.387720
H	-4.906529	2.144261	-3.770506
H	-7.010545	-0.026826	-0.986098
H	-3.268142	-1.884173	-4.113669
H	-6.535438	-3.080124	-0.249484
H	-5.353913	-4.022287	-1.963782
H	-6.144828	0.909424	-3.466239
H	-4.435741	-4.287108	-3.438144
H	-6.281581	-1.877999	-2.878882
H	-5.353873	-2.088797	-4.374544
H	-6.634205	-3.241995	-3.960184
K	-0.098088	3.694861	-0.158734
K	0.102904	-3.535714	-0.040662
N	3.563171	2.003968	0.540044
N	3.714533	-1.760535	-0.780239
N	-3.701496	1.892111	-0.483218
N	-3.566757	-2.023843	0.706756
Si	5.111629	1.827278	1.304814
Si	5.286576	-1.516708	-1.477383
Si	-5.144191	-1.869501	1.418024
Si	-5.220297	1.612484	-1.267945
Sr	2.176459	0.085306	-0.194802
Sr	-2.209472	-0.028851	0.134245

6. References

[S1] S. Kundu, S. Sinhababu, M. M. Siddiqui, A. V. Luebben, B. Dittrich, T. Yang, G. Frenking, H. W. Roesky, *J. Am. Chem. Soc.* **2018**, *140*, 9409–9412.

[S2] T. X. Gentner, B. Rösch, G. Ballmann, J. Langer, H. Elsen, S. Harder, *Angew. Chem. Int. Ed.* **2019**, *58*, 607–611.

[S3] J. Mai, M. Morasch, D. Jędrzkiewicz, J. Langer, B. Rösch, S. Harder, *Angew. Chem. Int. Ed.* **2023**, *62*, DOI 10.1002/anie.202212463.

[S4] B. Rösch, T. X. Gentner, J. Langer, C. Färber, J. Eyselein, L. Zhao, C. Ding, G. Frenking, S. Harder, *Science* **2021**, *371*, 1125–1128.

[S5] (a) Rigaku Oxford Diffraction, **2020**, CrysAlisPro Software system, version 1.171.40.84a, Rigaku Corporation, Wroclaw, Poland (compounds $[(BDI^*\text{-}H)Sr]_2(THF)_2$ and $(BDI^*)_2Sr(THF)_2$·*n*-pentane); b) Rigaku Oxford Diffraction, **2022**, CrysAlisPro Software system, version 1.171.42.72a, Rigaku Corporation, Wroclaw, Poland (compounds ($^{DIPeP}NN\text{-}H_2$), ($^{DIPeP}NN$)Sr(THF)$_2$ and ($^{DIPeP}NN$)Ca); c) Rigaku Oxford Diffraction, **2024**, CrysAlisPro Software system, version 1.171.43.106a, Rigaku Corporation, Wroclaw, Poland (all other compounds).

[S6] O. V. Dolomanov, L. J. Bourhis, R.J. Gildea, J. A. K. Howard, H. Puschmann, *J. Appl. Cryst.*, **2009**, *42*, 339–341.

[S7] G. M. Sheldrick, *Acta Crystallogr., Sect. A: Found. Adv.* **2015**, *71*, 3–8.

[S8] G. M. Sheldrick, *Acta Crystallogr., Sect. C: Struct. Chem.* **2015**, *71*, 3–8.

[S9] P. van der Sluis, A. L. Spek, *Acta Crystallogr., Sect. A: Found. Crystallogr.* **1990**, *46*, 194–201.

[S10] A. Thorn, B. Dittrich and G. M. Sheldrick, *Acta Crystallogr., Sect. A: Found. Crystallogr.* **2012**, *68*, 448–451.

[S11] J.-S. Jiang, A. T. Brünger, *J. Molec. Biol.,* **1994**, *243*, 100-115.

[S12] M. J. Frisch, G. W. Trucks, H. B. Schlegel, G. E. Scuseria, M. A. Robb, J. R. Cheeseman, G. Scalmani, V. Barone, G. A. Petersson, H. Nakatsuji, X. Li, M. Caricato, A. V. Marenich, J. Bloino, B. G. Janesko, R. Gomperts, B. Mennucci, H. P. Hratchian, J. V. Ortiz, A. F. Izmaylov, J. L. Sonnenberg, D. Williams-Young, F. Ding, F. Lipparini, F. Egidi, J. Goings, B. Peng, A. Petrone, T. Henderson, D. Ranasinghe, V. G. Zakrzewski, J. Gao, N. Rega, G. Zheng, W. Liang, M. Hada, M. Ehara, K. Toyota, R. Fukuda, J. Hasegawa, M. Ishida, T. Nakajima, Y. Honda, O. Kitao, H. Nakai, T. Vreven, K. Throssell, J. A. Montgomery, J. E. Peralta, F. Ogliaro, M. J. Bearpark, J. J. Heyd, E. N. Brothers,

K. N. Kudin, V. N. Staroverov, T. A. Keith, R. Kobayashi, J. Normand, K. Raghavachari, A. P. Rendell, J. C. Burant, S. S. Iyengar, J. Tomasi, M. Cossi, J. M. Millam, M. Klene, C. Adamo, R. Cammi, J. W. Ochterski, R. L. Martin, K. Morokuma, O. Farkas, J. B. Foresman, D. J. Fox, *Gaussian 16 Rev. A.03*, Wallingford CT, **2016**.

[S13] A. D. Becke, *J. Chem. Phys.* **1993**, *98*, 5648–5652.

[S14] J. P. Perdew, J. A. Chevary, S. H. Vosko, K. A. Jackson, M. R. Pederson, D. J. Singh, C. Fiolhais, *Phys. Rev. B* **1993**, *48*, 4978–4978.

[S15] F. Weigend, *Phys. Chem. Chem. Phys.* **2006**, *8*, 1057–1065.

[S16] F. Weigend, R. Ahlrichs, *Phys. Chem. Chem. Phys.* **2005**, *7*, 3297.

[S17] S. Grimme, S. Ehrlich, L. Goerigk, *J. Comput. Chem.* **2011**, *32*, 1456–1465.

[S18] E. D. Glendening, J. K. Badenhoop, A. E. Reed, J. E. Carpenter, J. A. Bohmann, C. M. Morales, P. Karafiloglou, C. R. Landis, F. Weinhold, *NBO 7.0*, Theoretical Chemistry Institute, University of Wisconsin, Madison, **2018**.

[S19] N. J. R. van Eikema Hommes, *Molecule*, Erlangen, **2018**.
